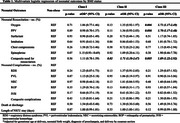# Poster Session 3: Thursday, February 12, 2026 10:30 AM – 12:00 PM

**DOI:** 10.1002/pmf2.70168

**Published:** 2026-02-09

**10:30 AM – 12:00 PM**

## 698 | Clinical Factors Associated With Hyponatremia in Preeclampsia With Severe Features: A Matched Case‐Control Study

A. U. Amanda Nwaba^1^; Meralis V. Lantigua‐Martinez^2^; Nikita A. Kakkad^1^; Xiaoying Zheng^1^; Kate Tolleson^3^; Cody Goldberger^4^; Linda Zambrano^5^; Steven Friedman^6^; Erinn M. Hade^6^; Ashley S. Roman^2^; Christina A. Penfield^7^

^1^NYU Grossman School of Medicine, New York, NY; ^2^Division of Maternal‐Fetal Medicine, Department of Obstetrics and Gynecology, NYU Grossman School of Medicine, New York, NY; ^3^New York University Grossman School of Medicine, NYU Grossman School of Medicine, NY; ^4^Department of Obstetrics and Gynecology, NYU Grossman School of Medicine, New York, NY; ^5^NYU Grossman School of Medicine, NYU Grossman School of Medicine, NY; ^6^Division of Biostatistics, Department of Population Health, NYU Grossman School of Medicine, New York, NY; ^7^Division of Maternal‐Fetal Medicine, Department of Obstetrics and Gynecology, NYU Grossman School of Medicine, New York City, NY

**Objective**: Hyponatremia is a rare but potentially life‐threatening condition associated with preeclampsia. We sought to characterize clinical factors associated with hyponatremia among patients with preeclampsia with severe features (PEC‐SF).

**Study Design**: We performed a multisite matched case‐control study of patients with PEC‐SF who delivered >34 weeks gestation and received magnesium sulfate between 1/2020–6/2024. Cases were defined as new‐onset moderate or severe hyponatremia (serum sodium <130 mEq/L) during the delivery admission. Controls were matched on a 1:1 basis by delivery date, delivery mode, and completed weeks gestation at delivery. We excluded patients with conditions or medications associated with hyponatremia. We evaluated demographics and clinical factors associated with hyponatremia. Logistic regression estimated the odds ratio (OR) and 95% confidence intervals (CI) for categorical covariates, and the Welch method was used to estimate mean difference (MD) and 95% CI assuming unequal variance for continuous characteristics.

**Results**: Of 3293 patients with PEC‐SF, 361 patients (11%) developed hyponatremia, and 292 patients met inclusion criteria. Compared to matched controls, cases more often had pregestational diabetes (6.2% vs 2%), lower BMI (32.0 kg/m2 vs. 34.4 kg/m2, 95% CI −3.5, −0.89), and higher maximum creatinine (0.94 mg/dL vs. 0.78 mg/dL, 95% CI 0.08, 0.24). Cases had longer magnesium exposure than controls (30.4 (14.5) vs. 33.5 (14.4) hours, 95% CI 0.69, 5.4). Hyponatremia was more common among those exposed to labor plus cesarean delivery (CD) compared to CD only (OR 1.44, 95% CI 0.92, 2.26) and less common among those diagnosed with PEC‐SF postpartum compared to antepartum (OR 0.42, 95% CI 0.26, 0.65). Magnesium diluent was not associated with risk of hyponatremia (Table 2).

**Conclusion**: Pregestational diabetes, antepartum PEC‐SF, longer magnesium exposure, and exposure to labor plus CD were associated with higher odds of hyponatremia. Thus, there may be a role for monitoring sodium levels during prolonged magnesium exposure, especially after an unsuccessful trial of labor.

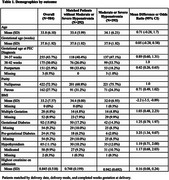

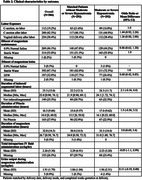

**10:30 AM – 12:00 PM**

## 699 | **STI** Coinfection **Does Not Increase Congenital Syphilis in Gravidas Effectively Treated for Syphilis in Pregnancy**

Abigail B. Clark^1^; Deepa Ravindra^2^; Jessica H. Wu^2^; Shreya Battu^2^; Heath Yancey^2^; Anastasia Kelley^2^; Jessica Sisco^2^; Jessica E. Pruszynski^2^; Emily H. Adhikari^2^

^1^University of Texas Southwestern Medical Center, University of Texas Southwestern Medical Center, TX; ^2^University of Texas Southwestern Medical Center, Dallas, TX

**Objective**: Evaluate the impact of sexually transmitted (STI) co‐infection pregnancy on maternal syphilis and congenital syphilis outcomes.

**Study Design**: In this retrospective cohort study, we identified pregnant patients with syphilis delivering at a large, public hospital. We excluded those with syphilis treatment prior to pregnancy, unless early infection was diagnosed during the current pregnancy. We reviewed medical records for coinfection with other STIs during the current pregnancy, including chlamydia, gonorrhea, HIV, and hepatitis B and C. We compared demographics, comorbidities, reported substance use, maternal syphilis characteristics and neonatal outcomes to determine the impact of STI coinfection on syphilis‐associated neonatal outcomes.

**Results**: From January 1, 2010 through June 30, 2025, 581 gravidas with syphilis were included, with 166 (28.6%) diagnosed with an STI co‐infection. Chlamydia was most frequent 117 (70%), followed by gonorrhea in 39 (23%), hepatitis C in 24 (15%), HIV in 19 (11%), and hepatitis B in 6 (4%). Patients with co‐infections were younger (24.4 v. 26.0, *p*  = 0.005) and more likely to have comorbid substance use (36% v. 26%, *p*  = 0.01). Patients with STI coinfection were diagnosed with syphilis later (21.5 vs 17.0 weeks, *p*  = 0.04), had fewer prenatal care visits, and were more likely to have early syphilis stage (66 [40%] vs 110 [27%], *p*  = 0.002). Frequency of benzathine penicillin G (BPG) administration before delivery was high in both groups. Syphilis‐exposed neonates born to individuals with co‐infection had lower birthweight after adjustment for the effect of maternal age and substance use. Infants born to individuals with STI co‐infections were not at increased risk for congenital syphilis after adjusting for maternal age, substance use and early syphilis stage.

**Conclusion**: STI co‐infection with maternal syphilis occurred in over 28% and was associated with maternal substance use and reduced prenatal care. While neonates had significantly lower birth weight, maternal STI coinfection was not independently associated with increased congenital syphilis.

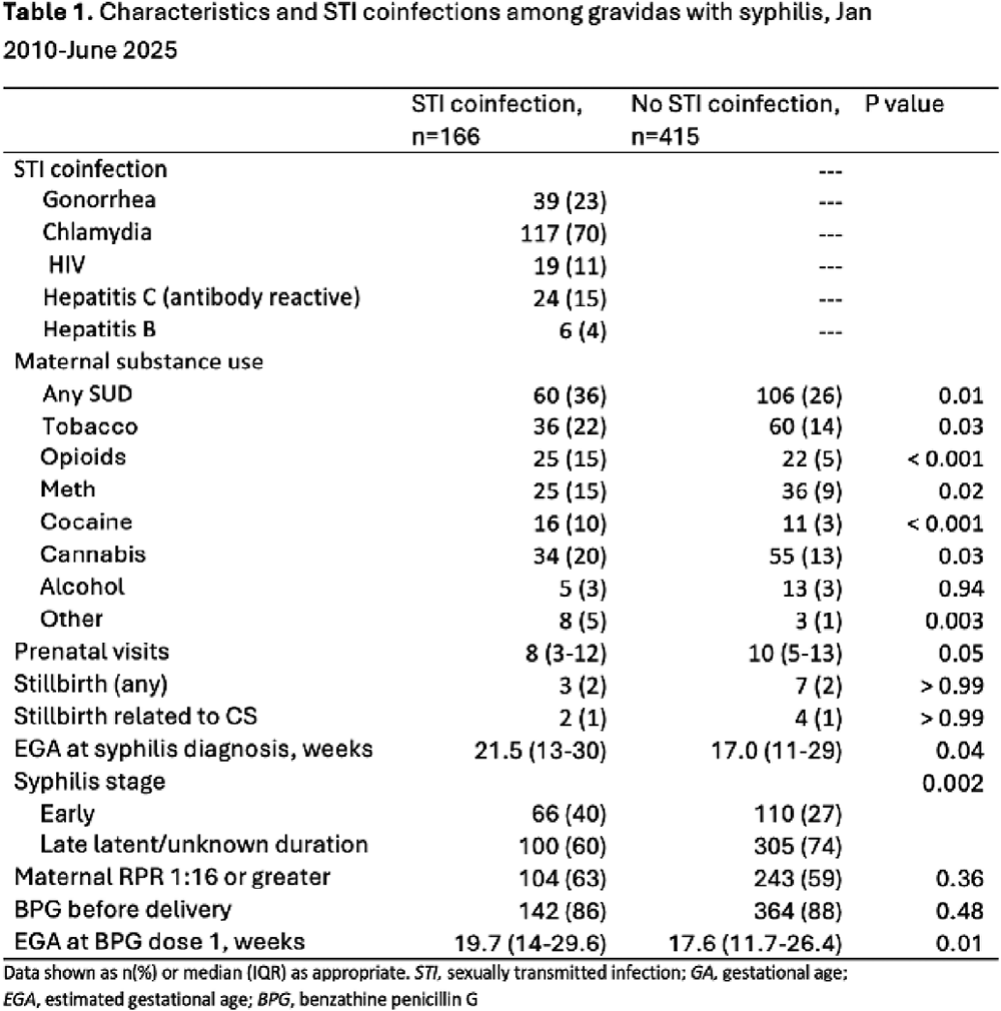

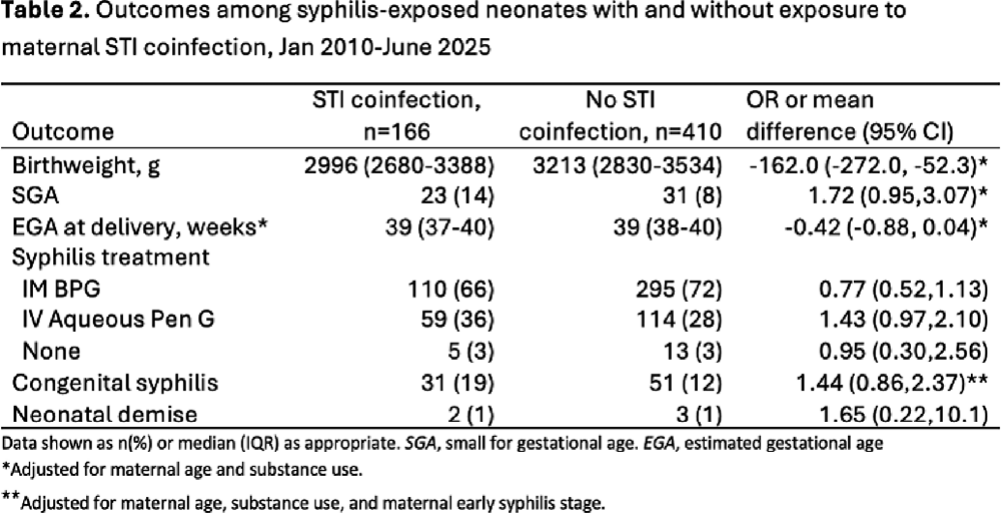

**10:30 AM – 12:00 PM**

## 700 | **Factors** Associated **With Completion of Tier 1 Carrier Screening**

Adams Mikaela^1^; Maura Jones Pullins^2^; Mia Hodges^3^; Emily Hardisty^1^; Madeline Dyke^1^; Neeta L. Vora^1^; Asha N. Talati^4^

^1^University of North Carolina at Chapel Hill, Chapel Hill, NC; ^2^University of North Carolina, Chapel Hill, Durham, NC; ^3^University of North Carolina at Chapel Hill, University of North Carolina, NC; ^4^University of North Carolina at Chapel Hill, University of North Carolina at chapel Hill, NC

**Objective**: To evaluate factors associated with completion of carrier screening (CS) during pregnancy at a quaternary care referral center.

**Study Design**: Chart review of G1 pregnancies at a single center from 01/2017–01/2024. Charts were manually reviewed for orders, documentation, and results of CS. Partner and neonatal charts were assessed for completion of CS and newborn screening (NBS). Exclusions included preconception or infertility care, transfer >20 weeks, unavailable prenatal records, or use of cell‐free DNA‐based CS. Complete Tier 1 CS was defined as screening for Cystic Fibrosis (CF), Spinal Muscular Atrophy (SMA), and Hemoglobinopathies (HB) (Tier 1). Compete fetal risk assessment was defined as screening in both partners if maternal carrier status was identified. Data were analyzed using descriptive statistics, t‐test, and chi‐square in Stata18.

**Results**: Of 1524 maternal charts reviewed, 522 were excluded with a final cohort of 1002 charts (Table 1). Overall, 646 (64.5%) underwent some form of CS; among those, 477 (73.8%) were screened for CF, 415 (64.2%) for SMA, and 617 (96%) for HB. Complete Tier 1 CS was documented in 372 (37.1%) patients. Patients who received genetic counseling were more likely to have complete Tier 1 CS compared to those who did not (53.4% vs. 46.6%, *p* < 0.001). 83 patients were identified as Tier 1 carriers. Of those, 27 (32.5%) had complete fetal risk assessment, which was significantly more likely in those who received genetic counseling (73.5% vs. 26.5%, *p* < 0.001). Patients seen in generalist or midwifery practices had higher rates of complete Tier 1 CS (*p* < 0.001) while insurance status was not associated (Figure 2). Among 81 neonates with a positive NBS, 52 (64%) screened positive for CF or HB. 1 neonate was confirmed positive for CF without documented parental screening. 1 neonate was confirmed positive for Sickle Cell Disease with complete fetal risk assessment.

**Conclusion**: Our findings underscore persistent gaps in the implementation of carrier screening guidelines. Enhancing access to genetic counseling may be a key strategy for improvement.

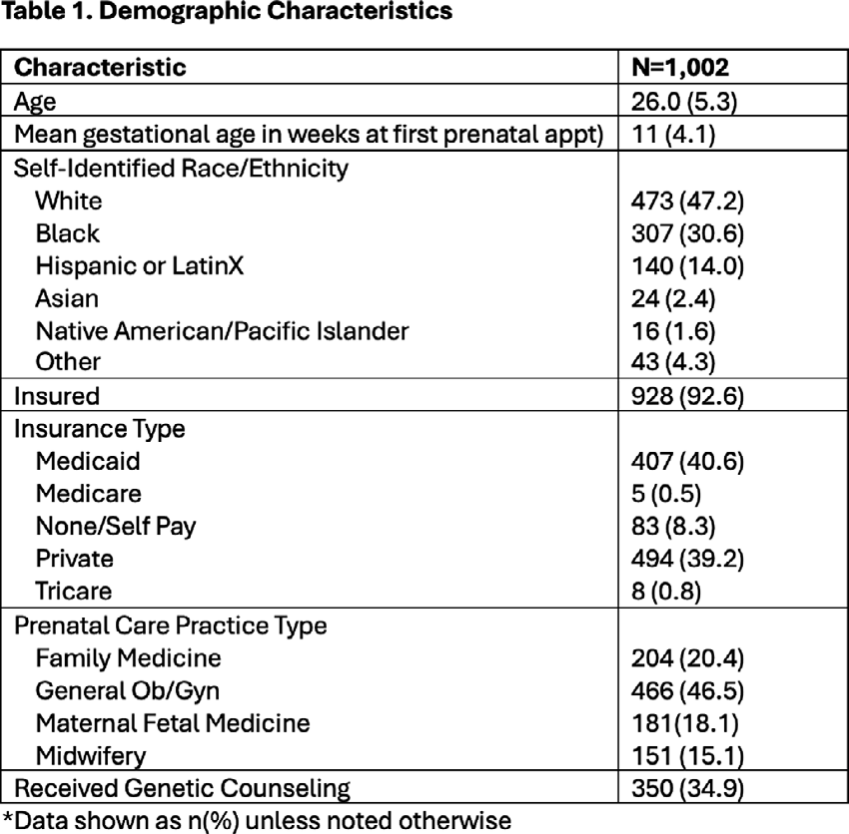

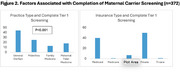

**10:30 AM – 12:00 PM**

## 701 | Evaluating **Indicators for Severe Maternal Morbidity From Pregnancy Onset through 1‐Year Postpartum**

Adina R. Kern‐Goldberger^1^; Mary Sefcik^2^; Meghana Sharma^2^; Anusha Kodi^2^; Oluwatosin Goje^2^; Justin R. Lappen^2^

^1^Cleveland Clinic Lerner College of Medicine, Cleveland, OH; ^2^Cleveland Clinic Foundation, Cleveland, OH

**Objective**: Severe maternal morbidity (SMM) is frequently evaluated exclusively during the delivery admission or immediate postpartum period. This may under‐ascertain true pregnancy risk as a substantial portion of maternal complications may occur in the extended postpartum period, and complications during pregnancy have more significant implications for the maternal‐fetal dyad. This study classifies an expanded SMM composite outcome by indication from week 0 of pregnancy through the extended postpartum period (1 year).

**Study Design**: This is a descriptive, retrospective cohort study in a multihospital health system including all deliveries from 1/1/2017–12/31/2024 (*n* = 57,092). SMM was defined by the CDC diagnosis codes as well as expanded codes for hemorrhage and puerperal infection (classified by Vizient), all occurring within 12 months of delivery, and ICU admission or hospital readmission within 12 weeks of delivery. Data were extracted from the electronic health record and plotted by pregnancy time point.

**Results**: Figure 1 demonstrates the distribution of SMM by indication starting from onset of pregnancy, and extending through “week 85.” The highest volume of SMM cases was the 33‐week time point, which likely represents patients who required preterm delivery due to SMM or SMM as a sequela of a pregnancy complication necessitating preterm delivery. Major drivers of this peak in SMM at this time point were readmission, ICU admission, and hemorrhage. Table 1 demonstrates the distribution of SMM indicators at the 10 time points with the highest incidence of SMM, defined relative to delivery (“week 0” = week of delivery). The highest incidence of SMM was the week of delivery, followed by the week after and the week before, respectively. The remainder of the SMM burden occurred largely between weeks 2–8 postpartum.

**Conclusion**: Trends in SMM indicators and volume over the expanded pregnancy course can inform focused prevention strategies and surveillance. Future institutional research and quality improvement efforts exploring targeted interventions can use this strategy for assessing SMM broadly as an outcome.

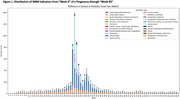

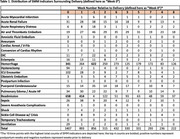

**10:30 AM – 12:00 PM**

## 702 | **Risk of** Subsequent **Preterm Birth in Patients With Failed Operative Vaginal Delivery Preceding Second‐Stage Cesarean**

Adwoa A. Baffoe‐Bonnie^1^; Hannah Kelly^1^; Lillian Boettcher^2^; Sarahn M. Wheeler^3^; Rachel L. Wood^4^

^1^Duke University School of Medicine, Durham, NC; ^2^Department of Obstetrics and Gynecology, Duke University School of Medicine, Duke University School of Medicine/Durham, NC; ^3^Division of Maternal Fetal Medicine, Department of Obstetrics and Gynecology, Duke University, Duke University/Durham, NC; ^4^Division of Maternal Fetal Medicine, Department of Obstetrics and Gynecology, Duke University, Durham, NC

**Objective**: Cesarean delivery in the second stage of labor is associated with an increased risk of spontaneous preterm birth (sPTB) in subsequent pregnancies. This risk may be further amplified by failed attempts at operative vaginal delivery (OVD) prior to cesarean. We sought to evaluate whether failed OVD preceding second‐stage cesarean increases the risk of sPTB in a subsequent pregnancy.

**Study Design**: This was a retrospective cohort study of patients who underwent term cesarean delivery during the second stage of labor at a single tertiary care center from March 2014–August 2024 and later delivered a subsequent pregnancy at ≥14 weeks’ gestation. The exposure was a documented failed vacuum or forceps attempt immediately preceding cesarean. The primary outcome was the incidence of sPTB (<37 weeks) in the subsequent pregnancy. Secondary outcomes included delivery characteristics of the index cesarean. Multivariable logistic regression was also performed. Statistical analyses were performed using Stata 18 SE.

**Results**: Of 275 patients who met inclusion criteria, 35 (12.7%) had a failed OVD preceding the index cesarean delivery; 14/35 (40.0%) with forceps and 21/35 (60.0%) with vacuum. Distribution of self‐reported race differed between the two groups; however, all other demographic characteristics were similar (Table 1). Patients who had a failed OVD prior to the index cesarean had an earlier mean gestational age at delivery (37.9 vs 38.6, *p* = 0.04) and a higher rate of subsequent preterm deliveries (17.1% vs 5.8%, *p* = 0.016) than those who did not have a failed OVD. Operative outcomes were similar among groups (Table 2). There was no difference in mode of delivery in subsequent pregnancies in failed OVD group (VBAC rate 17.1% vs 19.6%, *p* = 0.732).

In a multivariate logistic regression controlling for confounders, patients with a failed operative delivery prior to cesarean had significantly higher odds of subsequent sPTB (aOR 5.2, 95% CI 1.7–15.5).

**Conclusion**: Even after adjustment, patients who had a failed OVD prior to second‐stage cesarean had higher odds of preterm birth in the subsequent pregnancy.

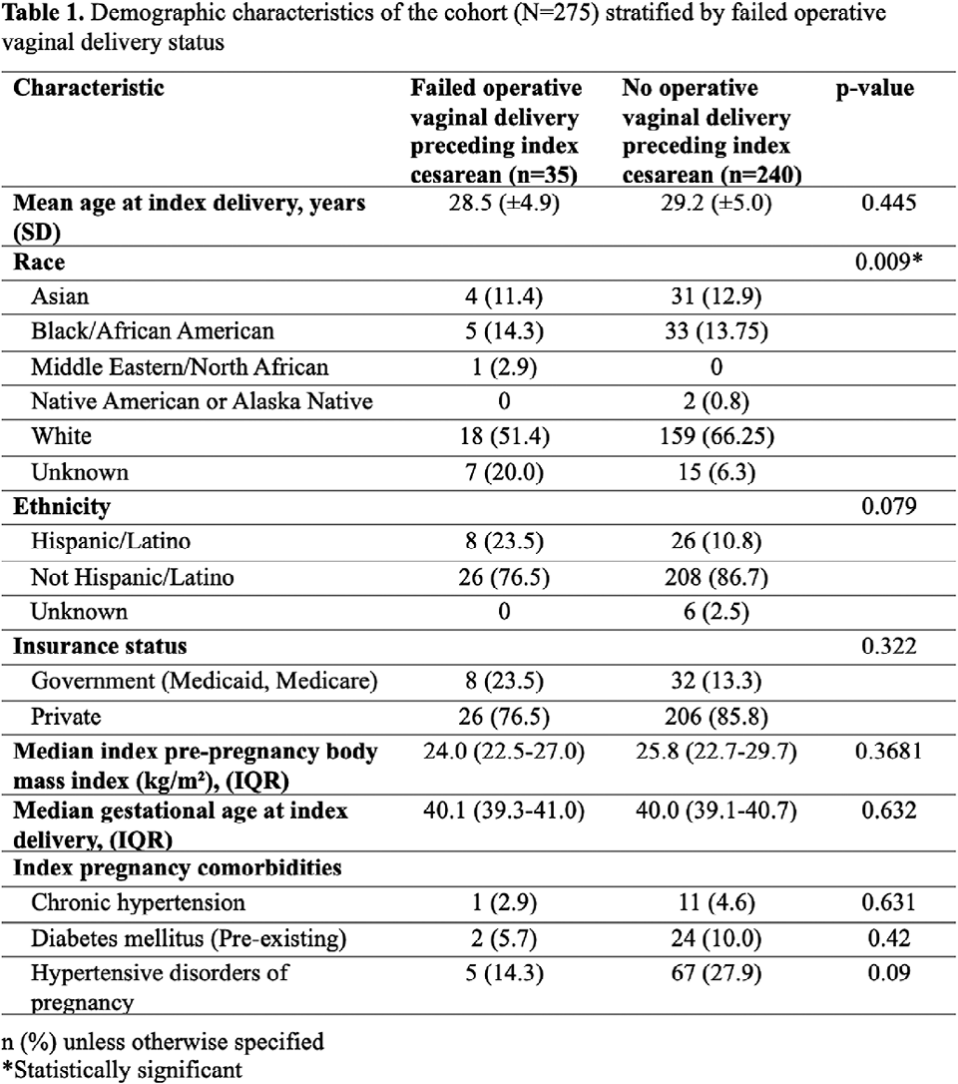

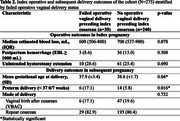

**10:30 AM – 12:00 PM**

## 703 | **Uterine** Rupture**: Comparison of Intact Versus Scarred Uterus**

Alesha M. White^1^; Isabelle Mulder^2^; Nicole Seyfried^2^; Lindsay Yeh^2^; Deepa Ravindra^2^; Donald D. McIntire^2^; F. Gary Cunningham^2^; C. Edward Wells^2^; Elaine L. Duryea^2^

^1^University of Texas Southwestern Medical Center, Grand Prairie, TX; ^2^University of Texas Southwestern Medical Center, Dallas, TX

**Objective**: Rupture of an unscarred uterus is uncommon with data limited to case reports and series. The objective of this study was to report on perinatal outcomes in cases of uterine rupture in an unscarred uterus when compared to a scarred uterus.

**Study Design**: This is a retrospective cohort study at a single institution including all patients with a diagnosis of uterine rupture between May 2009 and November 2024. Perinatal outcomes were compared between patients who experienced a uterine rupture with a history of prior uterine surgery to those with an unscarred uterus. Of note, at this institution during the study period trial of labor after cesarean is not recommended without a known history of one uncomplicated prior transverse cesarean, and oxytocin is not routinely administered in the setting of prior uterine scar and a non‐anomalous live fetus.

**Results**: Out of 187,352 deliveries, there were 95 (0.51 per 1000) uterine ruptures. The rate of uterine rupture in the setting of prior uterine surgery was 57/36,712 (1per 625). The rate of uterine rupture of an unscarred uterus was 38/150,640 (1 per 4000). Patients with an unscarred uterus were older, more likely to be Hispanic and nulliparous, delivered at later gestational ages, and more commonly underwent induction (all *p* < 0.05) (Table 1). The median Maximum cervical dilation of patients with an unscarred uterus was 8.5 (6–10) compared to 3.5 (1–6) in patients with a scarred uterus (*p* < 0.001). Their rupture was more likely to occur in the second stage of labor (39% vs 18%, *p* = 0.017) and in the posterior aspect of the uterus (21% vs 2%, *p* = 0.002). They more often required blood transfusion (79% vs 58%, *p* = 0.026) and a hysterectomy (48% vs 23%, *p* = 0.021). Fetal weight was higher in the unscarred cohort (3663 (3304–3993g) vs 3220 (2595–3688 g), *p* < 0.001) and base excess < −12 was more common (63% vs 35%, *p* = 0.006) (Table 2).

**Conclusion**: Uterine rupture with unscarred uterus, while rare, is more often associated with adverse maternal and neonatal outcomes including transfusion, hysterectomy, and elevated base excess.

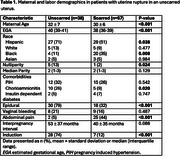

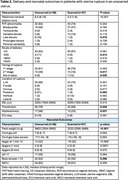

**10:30 AM – 12:00 PM**

## 704 | Correlation **of Point‐of‐Care Viscoelastic Testing and Standard Indices in an Obstetric Population**

Alesha M. White^1^; Donald D. McIntire^2^; Weike Tao^2^; Brenda Anyaehie^2^; Sean Yates^2^; David B. Nelson^2^

^1^University of Texas Southwestern Medical Center, Grand Prairie, TX; ^2^University of Texas Southwestern Medical Center, Dallas, TX

**Objective**: As coagulopathy is a major possible complication of postpartum hemorrhage (PPH), use of coagulation labs are recommended to guide transfusion. The use of viscoelastic point‐of‐care tests (POC) has not been validated in the setting of PPH but has been shown to help in the guidance of transfusion in other areas of medicine. The objective of this study was to demonstrate the correlation of standard coagulation labs with two different viscoelastic tests.

**Study Design**: This is a prospective observational study of patients who experienced PPH or who were at risk of experiencing PPH at the time of delivery. This data is a subgroup from a single‐center within a larger, multicenter industry sponsored study to validate a POC viscoelastic assay device, the Quantra^®^ sonorheometry (SEER) system. The correlation of hematologic indices with rotational thromboelastometry (ROTEM) and Quantra as well as the correlation of ROTEM with POC Quantra were compared with *p* < 0.05 considered significant.

**Results**: Of the total 40 samples, ROTEM assays were collected for 18 (45%) of patients and Quantra assays were collected for 40 (100%) patients. Conventional coagulation labs had a high correlation coefficient when compared to their ROTEM and Quantra equivalents, approaching 0.7 in all lab indices (all *p* < 0.05). Additionally, ROTEM and Quantra correlated well amongst themselves in the two examined equivalent parameters with correlation coefficients greater than 0.9 (all *p* < 0.05). Notably, the median total time for final result of Quantra was significantly less than ROTEM (*p* < 0.001) with Quantra having a first report projection of 2.2 [2.05,2.28] minutes.

**Conclusion**: Overall, the coagulation labs examined here correlate well with ROTEM and Quantra. POC Quantra results returned at significantly faster times compared to ROTEM. Further randomized controlled trials are needed to examine the impact of POC devices such as Quantra on guidance of transfusion in the setting of PPH.

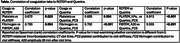

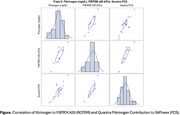

**10:30 AM – 12:00 PM**

## 705 | Understanding **Proteomics in Cases of Postpartum Hemorrhage**

Alesha M. White^1^; Andrea Woods^2^; Andrew Lemoff^2^; Ashley Solmonson^2^; Sean Yates^2^; David B. Nelson^2^

^1^University of Texas Southwestern Medical Center, Grand Prairie, TX; ^2^University of Texas Southwestern Medical Center, Dallas, TX

**Objective**: The objective of this study was to evaluate which proteins are upregulated and downregulated in patients who experience PPH when compared to patients who do not experience PPH.

**Study Design**: This is a prospective observational study of patients who were at‐risk for PPH with study cohorts defined as those who did and did not experience PPH. This data is a subgroup from a single‐center within a larger, multicenter industry sponsored study to validate a point‐of care viscoelastic assay device, the Quantra^®^ sonorheometry (SEER) system. Samples were analyzed for proteomics by LC‐MS/MS following abundant plasma protein depletion, disulfide bond reduction and alkylation, trypsin digestion, TMTpro labelling, and high‐pH fractionation. The peptide data was analyzed with Proteome Discoverer 3.0 against the human reviewed protein database from UniProt. Proteins with an abundance in 1/3 or more of the samples were analyzed. Benjamini‐Hochberg corrected *p*‐values were reported and a volcano plot created demonstrating fold change.

**Results**: A total of 41 patients were included, and of these, 33 (80%) experienced PPH with 121 proteins identified to be either significantly elevated or decreased in cases of PPH when compared to those without PPH (Figure). Myosin light chain kinase (3.84 fold increase, *p* < 0.001) and creatinine kinase B‐type (2.97 fold increase, *p* < 0.001) were the two most upregulated proteins in cases of PPH. The two most downregulated proteins in cases of PPH were chorionic somatomammotropin hormone 1 (3.90 fold decrease, *p* < 0.001) and glycoprotein hormone‐alpha subunit (1.93 fold decrease, *p* < 0.001).

**Conclusion**: Upregulated proteins associated with PPH were found to be proteins crucial to the control of PPH as it relates to uterine contractility. Downregulated proteins associated with PPH were found to be hormones with different functional properties in the postpartum state, not commonly thought to relate to uterine contractility. Further research is needed to understand if upregulation and downregulation of the respective proteins are predictive biomarkers for PPH risk.

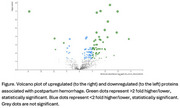

**10:30 AM – 12:00 PM**

## 706 | **Less Frequent Versus More Frequent Cervical Examinations During Labor: A Meta‐Analysis of Randomized Controlled Trials**

Alessandro Petrecca^1^; Chiara Tersigni^2^; Tullio Ghi^3^; Vincenzo Berghella^4^

^1^università cattolica del sacro cuore, Rome, Lazio; ^2^Fondazione Policlinico Gemelli IRCCS, Fondazione Policlinico Gemelli IRCCS, Lazio; ^3^Catholic University of Sacred Heart, Department of Women and Child Health, Fondazione Policlinico Gemelli IRCCS, Rome, Italy, Catholic University of Sacred Heart, Lazio; ^4^Thomas Jefferson University, Philadelphia, PA

**Objective**: To compare less frequent versus more frequent cervical examinations in labor in terms of obstetric, maternal, and neonatal outcomes

**Study Design**: Meta‐analysis of randomized controlled trials (RCTs). We included all RCTs written in English comparing less frequent versus more frequent cervical examinations in labor. The primary outcome was time from randomization to delivery. The secondary outcomes were duration of first and second stage of labor, number of vaginal examinations, incidence of chorioamnionitis, maternal fever, estimated blood loss (EBL) ≥500 mL, Apgar score <7 at 5 minutes, umbilical cord pH, admission to NICU.

 The summary measures were reported as relative risk (RR) or as mean difference (MD) with 95% of confidence interval (CI)

**Results**: Five RCTs, including 982 pregnancies, were analyzed. Three RCTs compared cervical exams in labor done every 8 vs 4 hours, one RCT ‘only if indicated’ vs 4 hours, and one RCT 4 vs 2 hours. The time from randomization to delivery was similar between the two groups (10.9 vs 9.79, *p* value = 0.78). Likewise, there were no significant difference between less frequent and more frequent cervical examinations during labor, with respect to the duration of first and second stage of labor, number of vaginal examinations, mode of delivery, chorioamnionitis rate, maternal fever, EBL ≥500 mL, maternal satisfaction, Apgar score <7 at 5 minutes, umbilical cord pH, admission to NICU (Table 1–2)

**Conclusion**: Less frequent compared to more frequent cervical examinations during labor have similar impact on time from randomization to delivery, and other obstetric, maternal, and neonatal outcomes. Based on these limited data, less frequent cervical examinations may be offered in labor, unless clinically indicated

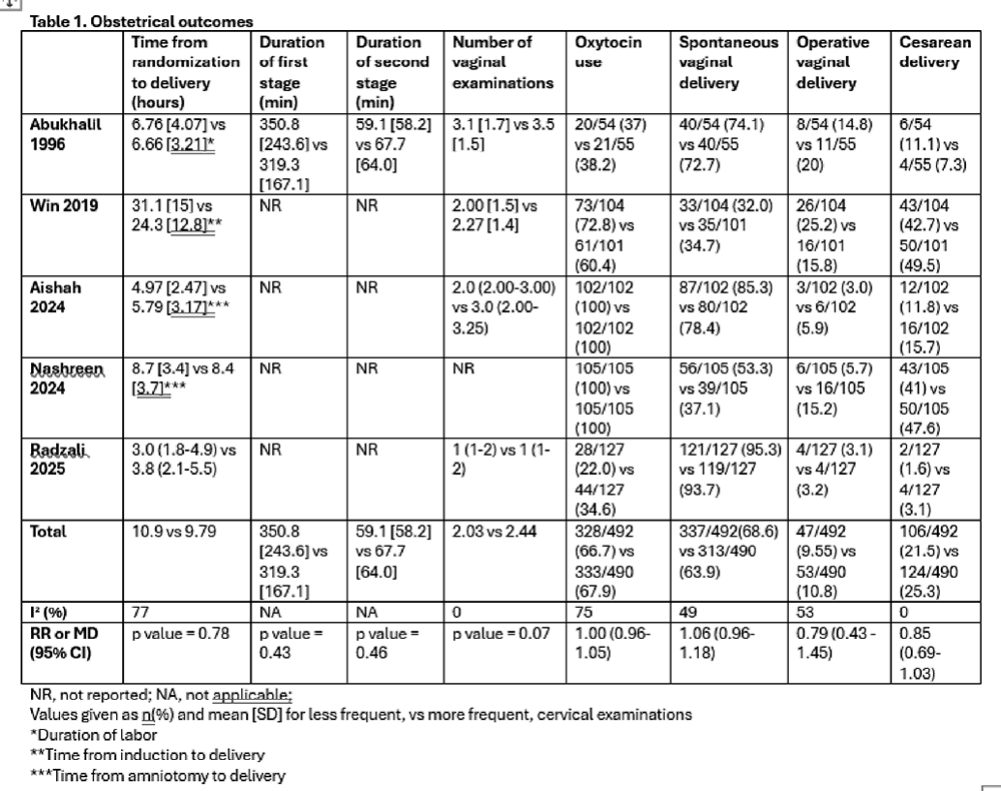

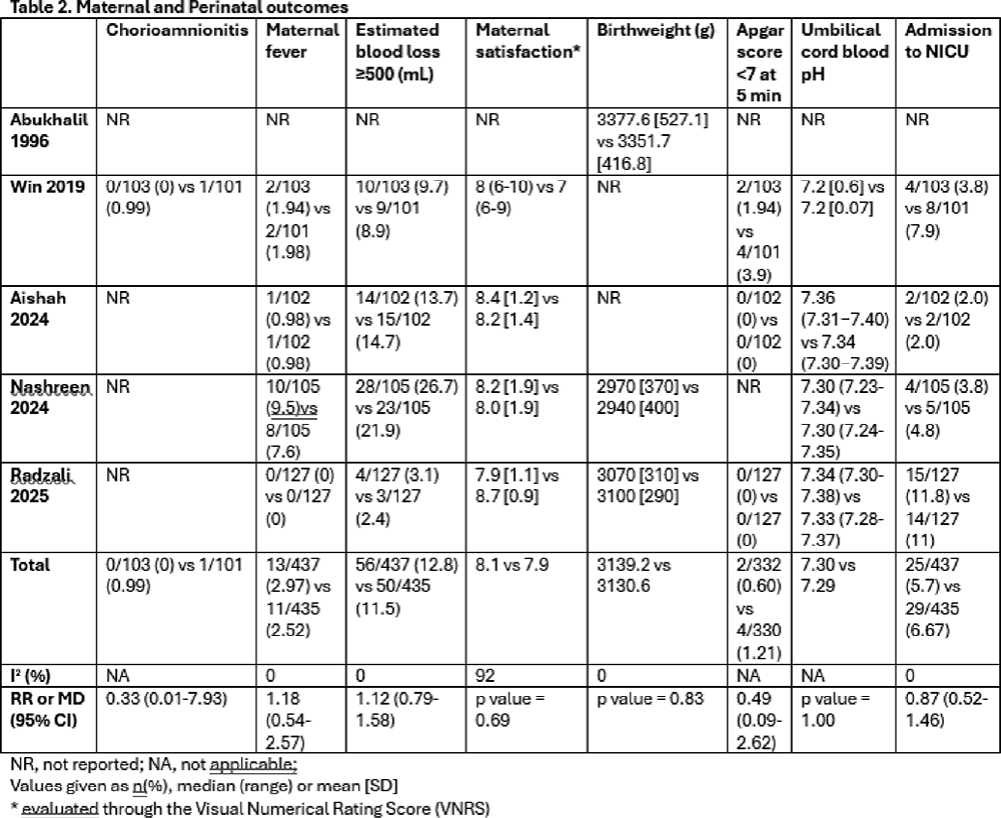

**10:30 AM – 12:00 PM**

## 707 | **Provider Perspectives of Socioeconomic Barriers and Facilitators of Tailored Prenatal Care in Academic Medical Systems**

Tanvi Sharma^1^; Elizabeth Krans^2^; David M. Haas^3^; Michelle Moniz^4^; Molly J. Stout^5^; Rachel A. Clark^6^; Sophia Fraga^1^; Kelsey Hoskin^1^; Julia Erickson^4^; Alex Peahl^7^

^1^University of Michigan Medical School, Ann Arbor, MI; ^2^UPMC Magee‐Womens Pregnancy and Women's Recovery Center, Pittsburgh, PA; ^3^Indiana University, Indiannapolis, IN; ^4^University of Michigan, University of Michigan, MI; ^5^University of Michigan, Ann Arbor, MI; ^6^Trinity Health – Ann Arbor Hospital, Ann Arbor, MI; ^7^University of Michigan Department of Obstetrics & Gynecology, Ann Arbor, MI

**Objective**: At the onset of the COVID‐19 pandemic, providers adapted prenatal care to incorporate telemedicine and targeted visit schedules. With these changes in clinical practice, the American College of Obstetricians and Gynecologists Clinical Consensus: Tailored Prenatal Care was published in May 2025. We describe providers’ perspectives of barriers and facilitators of adopting one component of tailored prenatal care models: addressing unmet social needs.

**Study Design**: We conducted semi‐structured interviews with 30 clinically active maternity care professionals at three academic sites. Interviews were transcribed, imported into MaxQDA, and iteratively coded using the Consolidated Framework for Implementation Research (CFIR), a structured framework for identifying barriers and facilitators of implementing evidence‐based interventions. Findings were organized by component of tailored care and then by CFIR domain to summarize drivers of tailored prenatal care adoption.

**Results**: Drivers of addressing unmet social needs were concentrated in the inner, outer, and individual settings. Inner setting facilitators were colocation of medical and social services and coordination of workflows, as well as a clinical culture of meeting social needs. Outer setting facilitators included support from community organizations and their integration with clinics. Outer setting barriers were maternity care deserts and varying insurance coverage for social needs (e.g., transportation, blood pressure monitors). Individual level facilitators included how tailored care could better accommodate work schedules, childcare, and transportation challenges. Barriers included lack of accommodations for specific populations (e.g., patients who do not primarily speak English).

**Conclusion**: Multilevel facilitators support clinics’ ability to address unmet social needs through routine prenatal care. Future work is needed to scale these interventions to the variety of locations where prenatal care is delivered, particularly safety net clinics where patients have higher rates of unmet social needs.

**10:30 AM – 12:00 PM**

## 708 | **Severe** Maternal **Morbidity in Pregnancy Among Individuals With Connective Tissue Disorders: A National Cohort Study**

Alexandra D. Forrest; Carrie Wolfson; Shriddha Nayak; Ryan King; Andreea Creanga; Marika Toscano

Johns Hopkins University, Baltimore, MD

**Objective**: Connective tissue disorders (CTDs) are characterized by systemic inflammation that increases risk for pregnancy complications, including preeclampsia, thromboembolism, and renal dysfunction. Given these known risks, we evaluated whether CTDs are associated with increased likelihood of severe maternal morbidity (SMM) during pregnancy and postpartum using a large real‐world electronic health record database.

**Study Design**: We conducted a retrospective analysis using TriNetX, a research platform of de‐identified electronic health records from over 155 million patients across 109 health systems. We identified pregnant individuals aged 12–55 years through July 2025 and categorized them based on presence or absence of CTD within the year prior to pregnancy, including antiphospholipid syndrome, rheumatoid arthritis, Sjögren's syndrome, systemic lupus erythematosus, and scleroderma. Propensity score matching balanced cohorts by age, race, ethnicity, obesity, asthma, hypertension, and hyperlipidemia. SMM was defined using the CDC's 21 indicators and ICD codes. We assessed odds of composite SMM and each individual condition occurring within one year of pregnancy onset.

**Results**: After matching, 37,365 individuals with CTD and an equal number of controls were analyzed (Table 1). Those with CTD had significantly higher odds of experiencing composite SMM (4.9% vs 2.0%, OR 2.8, 95% CI 2.8–3.4, *p* < 0.001). Nearly 2% of individuals with CTD experienced disseminated intravascular coagulation (DIC; OR 6.7, 95% CI 5.4–8.2; Table 2). Additional indicators with elevated risk included thromboembolic events –cerebrovascular complications (OR 4.5, 95% CI 3.4–6.0) and venous thromboembolism (OR 5.7, 95% CI 4.2–7.7).

**Conclusion**: Pregnant individuals with connective tissue disorders face significantly increased odds of SMM, with DIC and thromboembolism most strongly associated. This likely reflects immune‐mediated endothelial injury and proinflammatory cytokine activation. These findings underscore the need for risk stratification and multidisciplinary management to mitigate maternal risks.

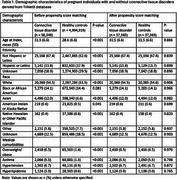

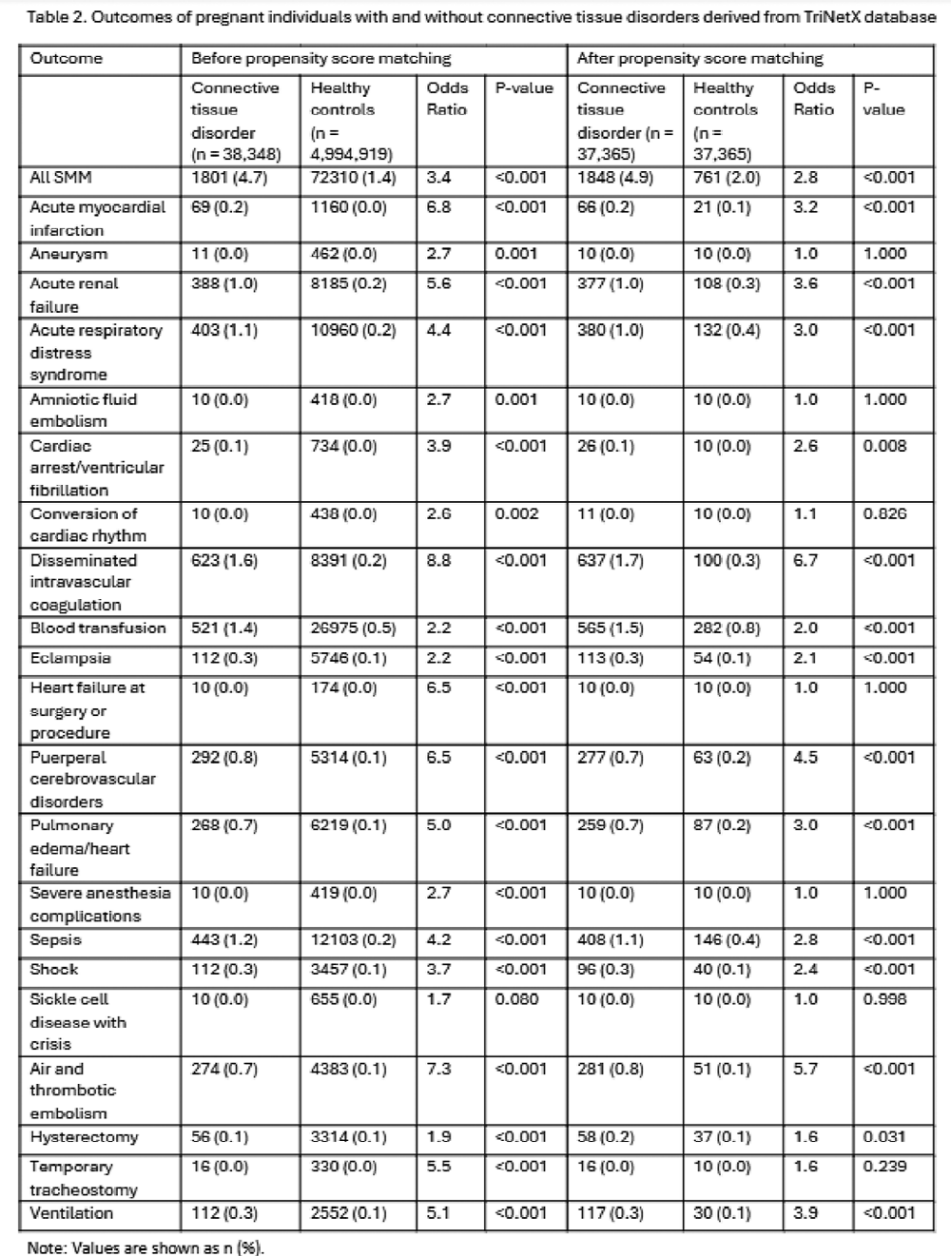

**10:30 AM – 12:00 PM**

## 709 | **Place,** not **Race? Social Vulnerability Index and Preterm Birth Risk**

Alexandria Williams^1^; David E. Cantonwine^2^; Thomas F. McElrath^3^

^1^Endeavor Health, Evanston, IL; ^2^Department of Obstetrics and Gynecology, MassGeneral Brigham; Division of Maternal‐Fetal Medicine; Harvard Medical School, Boston, MA; ^3^Brigham and Women's Hospital, Boston, MA

**Objective**: Social vulnerability index (SVI) is a CDC metric, ranging from 0 (least vulnerable) to 1 (most vulnerable), intended to identify areas that require urgent aid during natural disasters. SVI has been associated with a range of health outcomes, though only a few studies have focused on SVI and preterm birth (PTB). We aimed to investigate SVI, as an objective measure of structural racism or disadvantage, and PTB in a metropolitan area.

**Study Design**: LIFECODES participants in Boston, MA (2009–2017) were geocoded to census tract level and linked to SVI. Pregnancy losses and multiple gestations were excluded. Statistical analyses included: t‐test, ANOVA, chi square analysis, linear regression and logistic regression associated with SVI continuously and low, medium, and high SVI tertiles. Covariates in adjusted models were age, nulliparity, BMI, and chronic hypertension. Kaplan Meier survival analysis was performed for likelihood of remaining pregnant to 35 weeks. Models were evaluated using Akaike Information Criterion (AIC) to assess relative goodness of fit.

**Results**:*n* = 3273 were included in the analyses. The cohort represented the the entire range of SVI values. We found no significant relationship with SVI and risk of preterm birth <37 weeks (table 1). Risk of PTB <34 weeks in the highest vs lowest SVI tertiles were 4.3% vs 2.0% (< .01). Risk of PTB <28 weeks were 1.4% (high SVI) vs 0.5% (low SVI) (*p* < .01). In a model with SVI as a continuous predictor, adjusted odds ratio (OR) for PTB <37, <34, and <28 weeks were 1.4 (0.9, 2.0), 2.5 (1.1, 5.5), 6.7 (1.3, 40.0). See image 2 for Kaplan Meier curve. SVI had a lower AIC than a models with race as a predictor, indicating SVI alone has a better fit for predicting PTB.

**Conclusion**: Living in a higher SVI neighborhood is associated with increased OR for PTB <34 and <28 weeks among a diverse cohort in a Northeast metro area. In our cohort, SVI more accurately predicted PTB than race. Objective measures of structural disadvantage and/or racism may be a useful measure to identify groups at higher risk of early preterm birth compared to race alone.

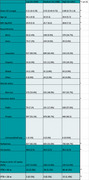

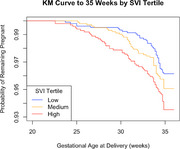

**10:30 AM – 12:00 PM**

## 710 | **Does** Second**‐Trimester Abdominal Wall Defect Size Predict Neonatal Outcomes in Gastroschisis?**

Nikan Zargarzadeh^1^; Ryne A. Didier^1^; Ali Javinani^2^; Giulia Bonanni^1^; Enaja Sambatur^3^; Shohra Qaderi^4^; Tom Jaksic^1^; Kjersti M. Aagaard^5^; Terry Buchmiller^1^; Alireza A. Shamshirsaz^6^

^1^Boston Children's Hospital, Boston, MA; ^2^Department of Obstetrics and Gynecology The George Washington University, Washington D.C., DC; ^3^Southeast Health Medical Center, Southeast Health Medical Center, AL; ^4^Baylor College of Medicine, Houston, TX; ^5^HCA, Houston, TX; ^6^Boston Children's Hospital, Harvard Medical School, Boston, MA

**Objective**: To determine whether abdominal wall defect size at 2nd trimester prenatal ultrasound predicts composite neonatal outcomes in fetuses with gastroschisis.

**Study Design**: This IRB‐approved retrospective cohort study included all pregnancies with a prenatal ultrasound diagnosis of gastroschisis at a tertiary center between January 2000 and December 2023. Cases were identified through the fetal imaging database, and abdominal wall defect size was measured in axial views using the umbilical cord insertion site as a landmark. We then applied a generalized additive model (GAM) to assess the association between abdominal wall defect size (measured at the second trimester ultrasound) and neonatal outcomes, adjusting for gestational age at delivery, external and intra‐abdominal bowel dilation (EBD and IBD), abdominal circumference (AC), and fetal growth restriction (FGR).

**Results**:*n* = 71 pregnancies met inclusion criteria. The mean gestational age at the time of the reference ultrasound was 21.3 weeks (SD ± 3.7). The model identified a “safe zone” between the 33rd and 55th percentiles of defect size (7.0–8.5 mm), where predicted neonatal outcomes were significantly better and the entire 85% confidence interval remained above zero. This middle range also represents the most reliable predictive zone, as model accuracy decreases at extremes. In adjusted analysis, IBD (β = 2.78, *p* = 0.002) and EBD (β = 2.15, *p* = 0.025) were independent predictors of worse outcomes.

**Conclusion**: In this single‐center, tertiary referral center study, mid‐range abdominal wall defect sizes—between the 33rd and 55th percentiles—were associated with significantly improved composite neonatal outcomes, while external and intra‐abdominal bowel dilation independently predicted worse outcomes. While it is well established that large defects are associated with extensive herniation and visceral injury, our study robustly estimates the thresholds for association of outcomes based on specific estimates of the size of the defect at the time of fetal anatomic survey. This should aid MFM clinicians in counseling patients reliably.

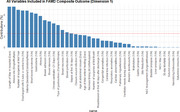

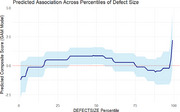

**10:30 AM – 12:00 PM**

## 711 | **The** Preeclampsia **Education Postpartum Study (PrEPS): A Randomized Controlled Trial**

Alice Sherman‐Brown^1^; Ann Nguyen Pham^2^; Shahnaz Vellani^3^; Megan C. Oakes^3^

^1^University of California, Irvine, Department of OBYGYN, Division of Maternal Fetal Medicine, Long Beach, CA; ^2^University of California, Irvine, Orange, CA; ^3^MemorialCare Miller Children's and Women's Hospital, Long Beach, CA

**Objective**: Preeclampsia is associated with short‐ and long‐term maternal morbidity. Therefore, it is critical for providers to educate patients regarding the nature of the disease, ongoing health risks, and warning signs that indicate worsening of the disease. We aimed to identify the optimal educational tool for short‐ and long‐term preeclampsia knowledge retention.

**Study Design**: A pilot randomized controlled trial of postpartum patients with preeclampsia with or without severe features delivered at a single tertiary care center. Participants were randomized 1:1 to standard text‐only education (TE) or a graphic version of the same information (GE) and completed a baseline preeclampsia knowledge assessment prior to receiving the educational intervention. All patients also enrolled in remote blood pressure monitoring (RBPM). The primary outcome was short‐term difference in preeclampsia knowledge score (PKS) from baseline. Secondary outcomes included differences in long‐term (>4 week) PKS, engagement in RBPM, postpartum follow‐up, and readmission. Anxiety was assessed as a balancing measure using GAD–7. To detect at 15% difference in short‐term PKS with 80% power and assuming a baseline PKS of 50% and drop‐out rate of 20%, the calculated sample size was 36 patients. Analyses were by intention‐to‐treat.

**Results**: Of the 36 recruited patients, 17 were randomized to the TE and 19 were randomized to GE arms. Those who received GE had significantly higher change in short‐term PKS (+3.7, 95% CI 1.45–5.97, *p* = 0.003) and were more likely to engage in RBPM. Patients receiving TE had significantly higher change in long‐term PKS (+1.8, 95% CI 0.12–3.47, *p* = 0.03). There were no differences in short or long‐term anxiety scores or readmission rates between groups.

**Conclusion**: Those randomized to GE demonstrated improved short‐term understanding about preeclampsia and were more engaged in RBPM compared to those receiving TE. However, this knowledge improvement was not sustained. Continued investigation is needed on educational strategies to maintain knowledge of the potential lifelong implications of preeclampsia.

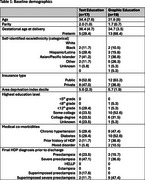

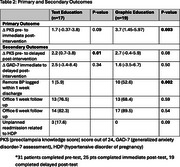

**10:30 AM – 12:00 PM**

## 712 | Prospective **Validation of the Obstetrical Transport Score (OTS) in Maternal‐Fetal Transport**

Alixandria F. Pfeiffer^1^; Mikaela Simmang^2^; Sarah A. T. Poor^2^; Michaela M. Kelly^2^; Leslie C. Omeire^3^; Arianna Braddom^2^; Hadley Ross^4^; Allison Moreno^5^; Patrick S. Ramsey^6^; John J. Byrne^7^

^1^University of Texas Health San Antonio, University of Texas health San Antonio, TX; ^2^University of Texas Health San Antonio, San Antonio, TX; ^3^University of Texas San Antonio, Long School of Medicine, San Antonio, TX; ^4^UT Health San Antonio, San Antonio, TX; ^5^University Health System, San Antonio, TX; ^6^Department of Obstetrics and Gynecology University of Texas Health Science Center in San Antonio, San Antonio, TX; ^7^University of Texas Health Science Center in San Antonio, San Antonio, TX

**Objective**: To prospectively evaluate the Obstetrical Transport Score (OTS), a novel triage tool designed to guide maternal‐fetal transport using maternal clinical, pharmacologic, physiologic, and fetal indicators.

**Study Design**: This is a prospective study of transported pregnant patients (April–July 2025) to a tertiary center. The Obstetrical Transport Score (OTS) is a 5‐level acuity tool (Levels 0–4) designed to guide transport triage. Receiving providers at the tertiary center assigned OTS scores based on clinical information provided by referring site and transport nurses assigned OTS scores at bedside evaluation at referring site. OTS level ≥3 indicates high acuity. The primary outcome was the overall predictive performance (threshold‐independent) of provider‐assigned OTS for maternal unit admission. The secondary outcome was prediction of a composite high‐acuity outcome: admission to L&D or ICU and/or urgent/emergent delivery within 24 hours. Statistical analyses included Chi‐Square testing and receiver operating characteristic (ROC) curve analysis.

**Results**: Of 74 transported patients, 71 (96%) had OTS scores from either provider, transport nurse, or both. Provider‐assigned OTS showed excellent discrimination for maternal unit admission (AUC 0.95, 95% CI 0.89–0.99, *p* < 0.001), with 100.0% sensitivity and 65.2% specificity at OTS ≥3. Transport nurse‐assigned OTS showed good discrimination (AUC 0.82, 95% CI 0.61–0.91, *p* < 0.001), with 93.9% sensitivity and 39.1% specificity. Both scores were significantly associated with high‐acuity outcomes (provider χ^2^ = 15.1, *p* < 0.001; RN χ^2^ = 7.4, *p*  = 0.006). In cases with dual scoring, transport nurse and provider scores matched in 43.3%.

**Conclusion**: The OTS demonstrated excellent performance in predicting maternal unit admission, regardless of assigning provider. This supports its use as a triage tool to guide obstetrical transport decisions and stratify maternal‐fetal risk.

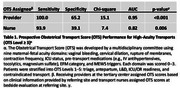

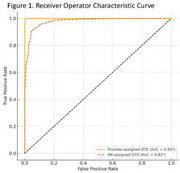

**10:30 AM – 12:00 PM**

## 713 | Trajectories **of Posttraumatic Stress Symptoms After a Vaginal Delivery: A Multicenter Prospective Study**

Alizee Froeliger^1^; Catherine Deneux‐Tharaux^2^; Lola Loussert^3^; Anne Laure Sutter‐Dallay^4^; Hugo Madar^5^; Loïc Sentilhes^5^

^1^Department of Obstetrics and Gynecology, Bordeaux University Hospital, Department of Obstetrics and Gynecology, Bordeaux University Hospital, France., Aquitaine; ^2^Université Paris Cité Institut Santé Des Femmes, Centre for Research in Epidemiology and Statistics (CRESS) U1153, Obstetrical Perinatal and Pediatric Epidemiology Research Team (Epopé), INSERM, INRAE, Paris, France., Paris, Ile‐de‐France; ^3^OPPaLE team, Centre for Research in Epidemiology and Statistics (CRESS) U1153, INSERM, Department of Obstetrics and Gynecology, Toulouse University Hospital, France., Languedoc‐Roussillon; ^4^Department of Perinatal Psychiatry, Charles Perrens Hospital, Bordeaux University Hospital, Department of Perinatal Psychiatry, Charles Perrens Hospital, Bordeaux University Hospital, France, Aquitaine; ^5^Bordeaux University Hospital, Bordeaux Universitary Hospital, Aquitaine

**Objective**: To characterize trajectories of posttraumatic stress symptoms between 2 days and 2 months after vaginal delivery at or near term and identify associated risk factors.

**Study Design**: Ancillary cohort study of the TRAAP randomized controlled trial in 15 French university hospitals (2015–2016), including women who delivered vaginally after 35 weeks. Endpoint was posttraumatic stress symptoms assessed at day 2 and 2 months postpartum using the validated Impact of Event Scale‐Revised (IES‐R). Four trajectories were defined: asymptomatic (IES‐R score<22 at both), recovered (≥22 at day 2, <22 at 2 months), emerging (< 22 at day 2, ≥22 at 2 months), and persistent (≥22 at both). Prevalences were corrected using inverse probability weighting to account for nonresponse. Associations with risk factors were examined using multivariate logistic regression.

**Results**: At both timepoints 2344/3891 women completed the IES‐R (60.2% response). Corrected prevalences were: asymptomatic 83.4% (95% CI, 81.8–85.0), recovered 11.0% (95% CI, 9.7–12.4), emerging 2.1% (95% CI, 1.6–2.8), and persistent 3.5% (95% CI, 2.7–4.4). In women without acute stress symptoms at day 2, emerging symptoms were associated with previous abortion (aOR 2.6; 95% CI, 1.6–3.9), pregnancy hospitalization (aOR 2.8; 95% CI, 1.4–4.7), labor induction (aOR 1.7; 95% CI, 1.1–2.8), bad delivery memories at day 2 (aOR 3.7; 95% CI, 1.4–5.6), intense fatigue at day 2 (aOR 2.2; 95% CI, 1.4–3.5), and was reduced by epidural (aOR 0.2; 95% CI, 0.1–0.7). In women with acute stress symptoms at day 2, persistent symptoms were associated with younger age (aOR 0.8; 95% CI, 0.6–0.9 per +5 years), non‐European origin (aOR 1.8; 95% CI, 1.2–2.6), psychiatric history (aOR 1.9; 95% CI, 1.2–2.4), bad delivery memories (aOR 2.4; 95% CI, 1.3–4.6), and hemoglobin < 9g/dL at day 2 (aOR 1.8; 95% CI, 1.1–2.7).

**Conclusion**: After term vaginal delivery, 5.6% of women had persistent or emerging posttraumatic stress trajectories up to 2 months postpartum. Persistent trajectories were mainly linked to preexisting factors, while emerging trajectories were more closely tied to obstetrical events.

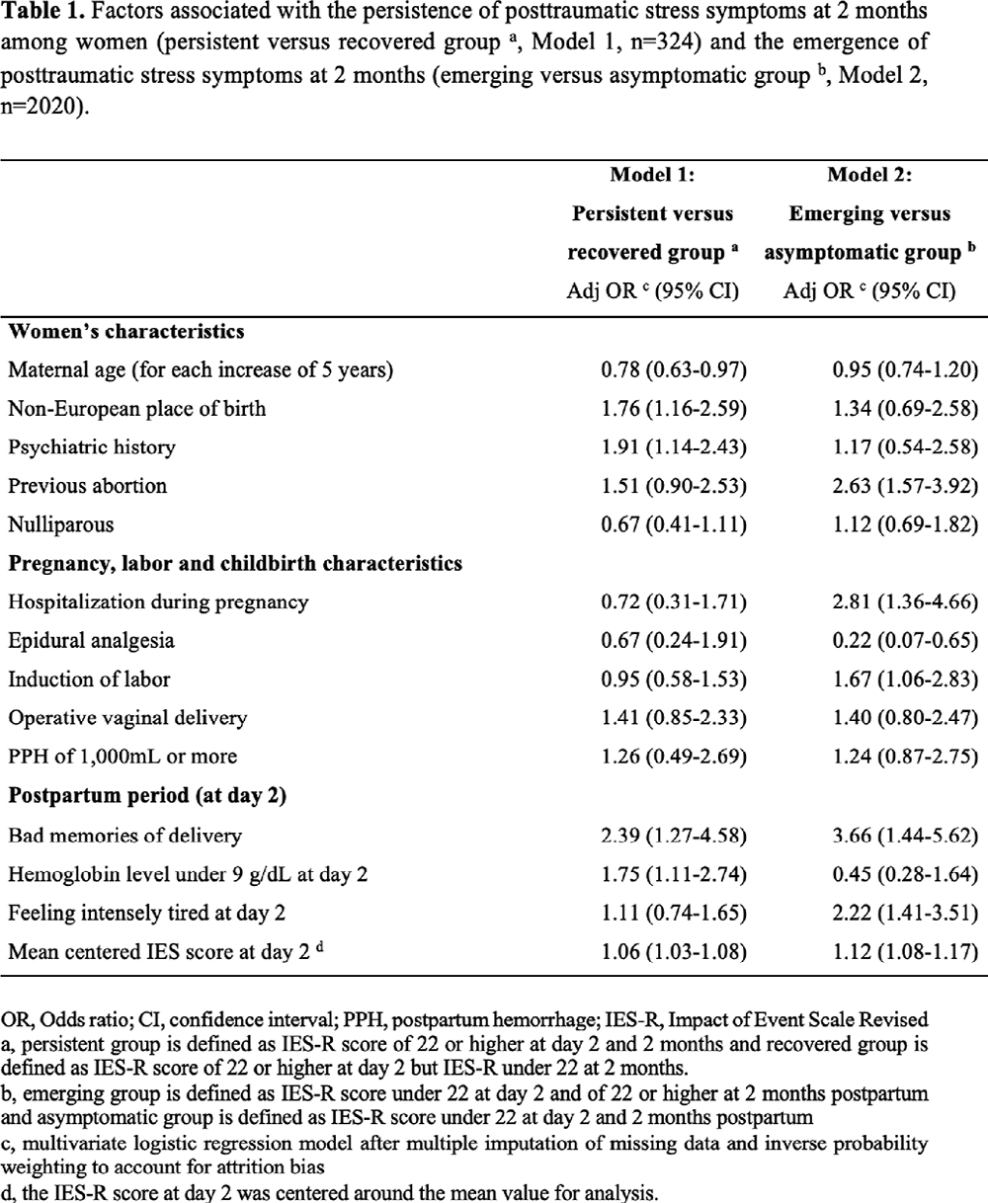

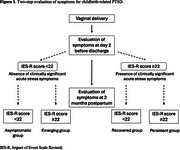

**10:30 AM – 12:00 PM**

## 714 | **Adverse miRNA and Protein Profiles Following Hypertensive Compared to Normotensive Pregnancy**

Allen Ghareeb^1^; James MacDonald^2^; Theo Bammler^2^; Shree Raj^2^

^1^University of Washington, University of Washington – Seattle, WA; ^2^University of Washington, University of Washington, WA

**Objective**: To identify differential microRNA (miRNA) expression in non‐pregnant individuals with and without history of a hypertensive disorder of pregnancy (HDP) and correlate this with previously measured inflammatory and cardiovascular disease (CVD)‐associated proteins in the same cohort.

**Study Design**: We conducted a case‐control study analyzing miRNA expression of 20 individuals with HDP history and 39 without HDP history. Peripheral blood was collected at a median of 4 years from the most recent pregnancy, and plasma was isolated using standard spin techniques. miRNA was extracted, libraries were prepared with unique molecular identifiers, and samples were sequenced in a single‐end mode, 75 bp reads. Transcripts were aligned to Homo Sapiens transcripts (miRBase v22). Data quality was assessed using multi‐dimensional scaling.

**Results**: There were no significant demographic differences between groups. Approximately 15 million reads per sample were generated with high quality scores. Using a false discovery rate of <0.1, we identified 26 differentially expressed miRNAs between the two cohorts (4 down‐regulated, 22 up‐regulated), many of which are implicated in hypertension or other CVDs (e.g., miR–200, miR–10a–5p, miR–3192–5p, let–7 family). Certain differentially expressed miRNAs relevant to cardiovascular (CV) conditions were significantly associated with CVD‐associated proteins or cytokines. For example, differentially expressed miR–3192–5p, implicated in angiogenesis and oxidative stress in epicardial tissue, is negatively correlated with three CVD‐associated proteins, fetuin‐A, fibrinogen, and L‐selectin (all estimates < –0.12, *p* < 0.001).

**Conclusion**: In our cohort, several miRNAs are differentially expressed in the few years after HDP compared to non‐HDP pregnancy, many of which are implicated in hypertension and other CVDs, and correlate with CVD‐associated proteins and inflammatory markers. This indicates that miRNAs, already well‐studied in the primary CVD literature, may present promising targets for CVD risk stratification in those with HDP history.

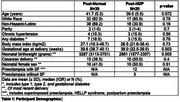

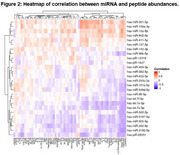

**10:30 AM – 12:00 PM**

## 715 | **The** Impact **of Selective Serotonin Inhibitor (SSRI) Treatment in Pregnancy on Long‐Term Cardiovascular Disease Outcomes**

Allison Carlisle^1^; Brandon Schickling^2^; Julie Vignato^1^; Serena Banu Gumusoglu^1^; Donna A. Santillan^1^; Mark K. Santillan^3^

^1^University of Iowa, Iowa City, IA; ^2^University of Iowa, University of Iowa, IA; ^3^University of Iowa, University of Iowa Health Care, IA

**Objective**: Preeclampsia is a hypertensive disorder of pregnancy associated with increased long‐term maternal cardiovascular disease (CVD) risk. Emerging evidence supports a shared pathophysiology between preeclampsia and depression involving serotonergic and vasopressin pathways. We aimed to evaluate whether SSRI use in pregnancy is associated with a reduced risk of future adverse CVD outcomes.

**Study Design**: This retrospective cohort study used data from the Intergenerational Health Knowledgebase (IIHK), comprising 11,437 pregnancies with documented PHQ‐9 scores. SSRI exposure was defined as an active prescription during pregnancy. The primary outcome was a composite of long‐term CVD diagnoses including atherosclerosis, arrhythmia, valvular disease, cardiomyopathy, and heart failure defined by ICD‐10 codes. Multivariable logistic regression models were constructed to evaluate the association between SSRI exposure and adverse CVD outcomes, to adjust for covariates.

**Results**: Of 11,437 pregnancies analyzed, a total of 944 individuals experienced adverse CVD. SSRI treatment during pregnancy was associated with a significant reduction in the odds of adverse CVD outcomes after controlling for depression severity, diabetes, age, BMI, and hypertensive disorders of pregnancy (adjusted OR 0.623; 95% CI: 0.538–0.722; *p* < 0.001). Independent risk factors for adverse cardiovascular outcomes included hypertensive disorders of pregnancy (aOR 1.68; 95% CI: 1.41–2.00), diabetes (aOR 1.31; 95% CI: 1.09–1.57), and White race (aOR 1.50; 95% CI: 1.27–1.79). Age, BMI, depression severity, and anxiety were not independently associated with CVD risk in adjusted models.

**Conclusion**: SSRI use during pregnancy was independently associated with reduced long‐term maternal cardiovascular disease risk. Our data demonstrates that treatment of perinatal psychiatric disease with SSRIs may contribute to decreased future CVD risk. Future studies should focus on identifying shared molecular mechanisms for perinatal psychiatric and cardiovascular disease.

**10:30 AM – 12:00 PM**

## 716 | Buprenorphine **Macrodose Inductions and Changes in Fetal Heart Rate Tracings**

Abigail B. Clark^1^; Allison Kurzeja^2^; Jessica Sisco^2^; Elaine L. Duryea^2^; Emily H. Adhikari^2^; Polly B. Cordova^3^; Jessica McNeil^3^; Amber Fisher^3^; Joshua Kern^2^; Nancy S. Onisko^2^; Aldo Andino^2^; Kurt Kleinschmidt^2^; C. Edward Wells^2^; Anne M. Ambia^2^

^1^University of Texas Southwestern Medical Center, University of Texas Southwestern Medical Center, TX; ^2^University of Texas Southwestern Medical Center, Dallas, TX; ^3^Parkland Health, Dallas, TX

**Objective**: To evaluate fetal heart rate tracing changes following induction of buprenorphine using a high‐dose protocol for medication‐assisted treatment (MAT) for opioid use disorder.

**Study Design**: This is a retrospective cohort study of patients admitted with opioid use disorder (OUD) in pregnancy. Patients were included if they underwent a buprenorphine induction in the antepartum setting and were greater than 24‐weeks' gestation. Fetal monitoring was reviewed for one hour following buprenorphine induction as well as any additional rescue doses. Tracings were independently reviewed and classified by three maternal fetal medicine physicians using the National Institute of Child Health and Human Development Criteria. Records were reviewed to assess subsequent antepartum and obstetrical management.

**Results**: From September 2023 through May 2025, 25 buprenorphine inductions occurred after 24 weeks' gestation. Of these, 19 (76%) had fetal monitoring obtained. Two (8%) patients had two buprenorphine inductions after 24 weeks’ gestation and thus had multiple tracings obtained. There were no cases of precipitated withdrawal. Most tracings remained reassuring, with 16 (84%) with a Category I tracing, 3 (16%) with a Category II tracing, and 0 with a Category III tracing. Of those with Category II tracings, the findings were transient, with no patients requiring intrauterine resuscitation or need for delivery due to non‐reassuring fetal status. Preterm contractions and arrested preterm labor complicated one induction at 36 weeks. Prelabor rupture of membranes occurred two days after one induction at 37 weeks, followed by repeat cesarean section. Two other patients underwent a scheduled delivery for usual obstetric indications during the same admission as the buprenorphine induction.

**Conclusion**: Our findings provide reassuring data that high‐dose buprenorphine inductions for medication‐assisted treatment in pregnancy does not result in acute non‐reassuring fetal heart rate patterns warranting emergent intervention. These preliminary findings support the acute safety of this induction protocol in the antepartum setting.

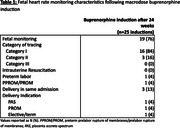

**10:30 AM – 12:00 PM**

## 717 | **Low‐Dose Versus High‐Dose Oxytocin for Induction and Augmentation of Labor: A Randomized Control Trial**

Amanda M. Wang^1^; Daphne Goncharov^2^; Sourabh Sharma^3^; Benjamin Bush^4^; George R. Saade^5^; Antonio Saad^6^

^1^HCA, League City, TX; ^2^Driscoll, Corpus Christi, TX; ^3^University of Texas Medical Branch, Galveston, TX; ^4^Emory University, Atlanta, GA; ^5^Department of Obstetrics and Gynecology, Eastern Virginia Medical School at Old Dominion University, Norfolk, VA; ^6^Inova Fairfax Hospital, Fairfax, VA

**Objective**: To compare the effect of low‐dose versus high‐dose oxytocin regimens on time to delivery in nulliparous individuals undergoing induction or augmentation of labor.

**Study Design**: Nulliparous individuals with singleton pregnancies at ≥37 weeks’ gestation who required oxytocin for induction or augmentation were randomized to receive a low‐dose (2 milli‐units/min initial dose, titrated by 2 milli‐units/min every 20 minutes) or high‐dose (6 milli‐units/min initial dose, titrated by 6 milli‐units/min every 20 minutes) regimen. Oxytocin was titrated to achieve adequate contractions; maximum rate of 40 milli‐units/min. The primary outcome was time from oxytocin initiation to delivery. Secondary outcomes included mode of delivery, uterine tachysystole, and maternal and neonatal complications. Data were analyzed using Mann‐Whitney Wilcoxon rank sum, Pearson chi‐square tests, and a Kaplan‐Meier curve with *p* < 0.05 considered significant. Sample size was calculated for superiority with a 25% difference rate and 80% power.

**Results**: 170 participants were randomized, 85 to the low‐dose (with 1 withdrawal) and 85 to the high‐dose group. Baseline characteristics were similar between groups. Most participants undergoing induction were admitted for elective labor and received mechanical cervical ripening with a Foley balloon. Median time from oxytocin initiation to delivery was 834 minutes in the low‐dose group and 726 minutes in the high‐dose group (*p* = 0.26). Participants in the high‐dose group received a higher maximum oxytocin dose (15.6 vs. 11.8 milli‐units/min, *p* = 0.004). No significant differences were found in mode of delivery or time to vaginal delivery (Figure 1, Table 1). The most common cesarean indications were failure to progress in the low‐dose group and non‐reassuring fetal heart rate in the high‐dose group. Estimated blood loss was significantly higher in the high‐dose group. No other significant differences were observed.

**Conclusion**: High‐dose oxytocin regimens did not significantly reduce time to delivery. While the high‐dose group received higher maximum oxytocin doses, safety profiles were comparable.

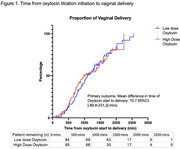

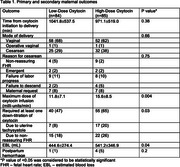

**10:30 AM – 12:00 PM**

## 718 | Symphysiolysis**: Investigating a Potential Contributory Association to Cesarean Delivery and Adverse Perinatal Outcomes**

Amir Snir^1^; Tamar Wainstock^2^; Eyal Sheiner^3^

^1^Soroka University Medical Center, omer, HaDarom; ^2^Department of Epidemiology, Biostatistics and Community Health Sciences, Faculty of Health Sciences, Ben‐Gurion University of the Negev, Beer Sheva, HaDarom; ^3^Soroka University Medical Center, Faculty of Health Sciences, Ben‐Gurion University of the Negev, beer sheva, HaDarom

**Objective**: Symphysiolysis is a bothering complication in pregnancy. The severity of symptoms varies from mild discomfort to severely debilitating pain. In this study we aimed to investigate whether beyond the discomfort, documented symphysiolysis is associated with adverse perinatal outcomes and whether it constitutes as an independent risk factor for cesarean delivery (CD) and labor induction.

**Study Design**: A population‐based cohort study was performed in a tertiary medical center including all singleton deliveries born between the years 1991–2021. Adverse perinatal outcomes were compared between pregnancies of women with and without documented symphysiolysis. The diagnoses were defined based on ICD‐9 codes as recorded in the hospital medical files. Multiple logistic regression models were used to control for confounders.

**Results**: Out of 232,476 patients, 227 (0.1%) were diagnosed with symphysiolysis during pregnancy. Perinatal outcomes such as low Apgar (<7) at 5 minutes (OR = 0.7, 95% CI 0.1–5.1, *p =*0.739) and perinatal mortality (OR = 0.54, 95% CI 0.07–3.8, *p =*0.53) were comparable between the groups. Higher rates of labor induction (OR 2.0, 95% CI1.5–2. 7, *p* < 0.001) and CD (OR = 4.3, 95% CI 3.3–5.65, *p* < 0.001, Table) were noted in pregnancies complicated by symphysiolysis. Using multiple logistic regression models, controlling for confounders such as maternal age, gestational diabetes mellitus, smoking, fertility treatments and mild pre‐eclampsia, symphysiolysis was noted as an independent risk factor for labor induction (adjusted OR = 1.8, 95% CI 1.3–2.4; *p* < 0.001) and CD (adjusted OR = 2.3, 95% CI 1.9–2.9; *p* < 0.001).

**Conclusion**: Symphysiolysis is an independent risk factor for cesarean delivery and labor induction. However, it is not associated with adverse perinatal outcomes.

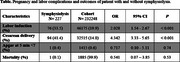

**10:30 AM – 12:00 PM**

## 719 | **National Consensus on Fetal Monitoring Protocols Following Maternal Trauma: Results From a Nationwide Survey**

Ana Collins‐Smith; Paloma Rodriguez Paramo; Aylia Rizvi; Celeste Traub

University of Texas Medical Branch, Galveston, TX

**Objective**: Trauma is the leading cause of nonobstetric death among pregnant women. However, management of obstetrical trauma varies significantly across U.S. institutions, largely due to the absence of standardized guidelines from the Society of Maternal‐Fetal Medicine or ACOG. This study aimed to describe current practices for fetal monitoring after maternal trauma among maternal‐fetal medicine (MFM) fellowship programs nationwide, highlighting variability and gaps.

**Study Design**: A cross‐sectional online survey was distributed via email to all current MFM fellows. The survey collected data on hospital characteristics and fetal and maternal evaluation practices after trauma. One response per fellowship program was analyzed using descriptive statistics.

**Results**: A 70% response rate was achieved. For previable fetuses (<24 weeks), no clear consensus emerged. Only 42.3% of respondents conducted comprehensive fetal assessments, including ultrasound or Doppler for fetal heart rate, vaginal or speculum exams, and labs.

By contrast, practices for viable gestations showed more consistency. Most programs reported a minimum of 4 hours of fetal monitoring, extended to 24 hours if contractions were present. Monitoring beyond 24 hours was reported by only ∼10% of institutions. Monitoring began at the time of trauma in 61.5% of responses, versus hospital presentation in 38.5%.

Most respondents (>90%) routinely obtained a standard lab panel for suspected placental abruption, including CBC, PT, PTT, fibrinogen, and type and screen. Additional labs such as Kleihauer‐Betke (KB) and urinalysis (UA) were rarely obtained unless the patient was Rh‐negative, in which case KB testing was routine.

**Conclusion**: This study shows significant variability in the evaluation and management of pregnant trauma patients, especially those under 24 weeks. While monitoring is more consistent in viable pregnancies, the lack of protocols for previable gestations highlights a critical gap. Findings support the need for evidence‐based guidelines to ensure consistent care and improve outcomes.

**10:30 AM – 12:00 PM**

## 720 | **Anemia** in **Pregnancy After use of GLP1 for Weight Management**

Andrea Neira Gesteira^1^; Mariella Gastanaduy^2^; Meena Mishra^3^; Sarah Olsen^4^; Frank B. Williams^5^

^1^Ochsner, ochsner/New Orleans, LA; ^2^Ochsner Center for Outcomes Research, New Orleans, LA; ^3^Ochsner Center of Outcomes & Health Services Research, New Orleans, LA; ^4^University of Queensland, University of Queensland, LA; ^5^Ochsner Clinic Foundation, Ochsner Clinic Foundation, LA

**Objective**: GLP1 agonist use for weight loss is increasingly common among reproductive age women, though the effects of preconception exposure remain to be determined. Accelerated weight loss can be associated with micronutrient deficiencies. We hypothesize there is a higher prevalence of early pregnancy anemia among those exposed to GLP1 agonists compared to matched controls.

**Study Design**: This single‐center, multi‐site retrospective cohort study included patients receiving prenatal and delivery care between Jan 2022 and Dec 2024 in a large regional health system. Patients with pregestational diabetes, bariatric surgery, hemoglobinopathy, and multifetal pregnancy were excluded, as were those presenting for prenatal care after the first trimester. The exposure group had documented GLP1 use for weight loss in the year prior to pregnancy. The control group was matched 1:2 by baseline pregnancy body mass index (BMI), maternal age, and payor status. Primary outcome was anemia, defined as either hemoglobin <11 g/dL or hematocrit <33%, at first trimester prenatal complete blood count. Secondary outcome was anemia at delivery admission. Baseline characteristics and outcomes were compared via chi square or Mann Whitney U test where appropriate. Outcome odds ratios were compared with 95% confidence intervals.

**Results**: Among 24,403 pregnancies, 195 GLP1‐exposed patients were matched to 330 unexposed controls by age, BMI and payor status. GLP1‐exposed patients were more likely nulliparous and have chronic hypertension while controls were more commonly Black (table). At initial assessment, no difference was observed in baseline anemia, with 9.2% of GLP1 patients and 11.8% of controls meeting criteria (aOR 0.80, 95% CI 0.4–1.4, figure). On delivery admission, anemia was observed among 32.7% of GLP1 patients compared to 42.1% of controls (OR 0.70, 95% CI 0.5–1.0).

**Conclusion**: In a multi‐site cohort, preconception GLP1 agonist exposure was not associated with increased anemia in pregnancy compared with age, BMI and insurer‐matched controls.

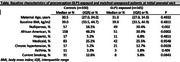

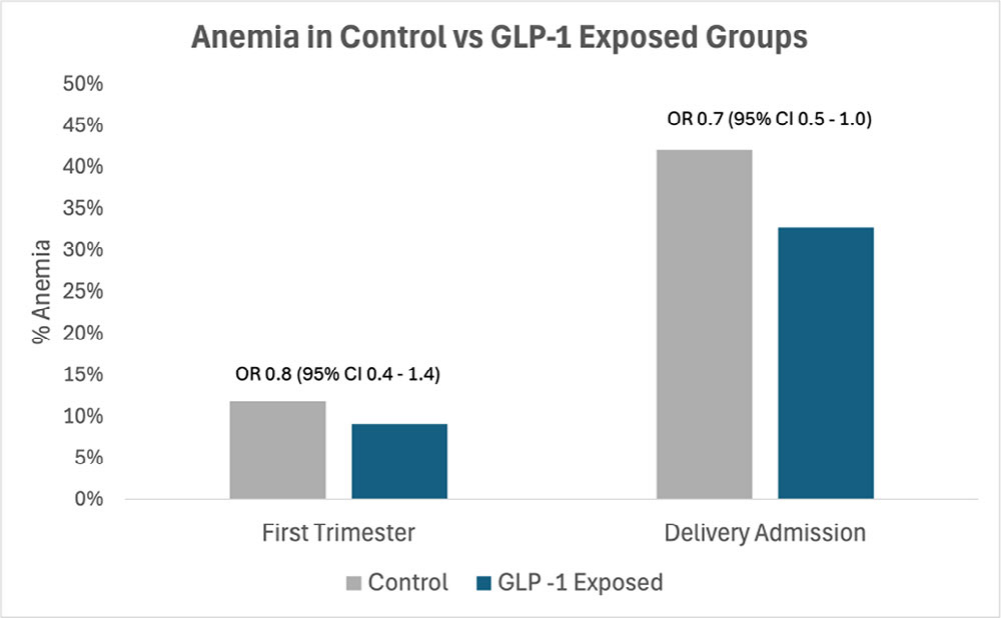

**10:30 AM – 12:00 PM**

## 721 | **Off‐label Use of Mirtazapine and Olanzapine for Hyperemesis Gravidarum: A Retrospective Cohort Study**

Andrew Housholder^1^; Marlena Fejzo^2^; Ellie Lewis^3^; Isabella Clements^4^

^1^The Morning Sickness Clinic, LLC, Birmingham, AL; ^2^University of Southern California, Keck School of Medicine, Los Angeles, CA; ^3^John Hopkins University, Baltimore, MD; ^4^University of Alabama at Birmingham, Birmingham, AL

**Objective**: To evaluate the effectiveness of off‐label mirtazapine and olanzapine for managing hyperemesis gravidarum (HG) unresponsive to standard therapies. Mirtazapine's 5‐HT3 and H1 antagonism reduces nausea, while alpha‐2 blockade increases appetite. Olanzapine's D2, 5‐HT3, H1, and M1 activity reduces nausea and vomiting, with evidence supporting its use in postoperative, chemotherapy‐related, and cyclic vomiting.

**Study Design**: A retrospective chart review was conducted at The Morning Sickness Clinic, which treated ∼1300 HG patients over 3 years. 100 patients who failed standard antiemetics were prescribed either mirtazapine (7.5–15 mg) or olanzapine (5 mg) based on physician clinical judgment, in addition to other meds and IV fluids. Outcomes were assessed using HELP and PUQE scores, vomiting frequency, and weight change at follow‐up 1–3 weeks post‐initiation. The study was deemed IRB exempt (BRANY IRB #24–12–337–1652)

**Results**: Among 62 patients prescribed mirtazapine, 46 had follow‐up visits (mean interval: 11 days; prior meds: 3.3; mean weight loss: 7.8 lbs; mean EGA: 12.8 weeks). At followup, HELP scores decreased by 10.0 points (SD ± 10.9; *p* < 0.001), PUQE scores decreased by 2.4 (SD ± 4.2; *p* < 0.001), vomiting decreased from 4.7x to 2.2x/day (Δ 2.5; SD ± 4.1; *p* < 0.001), and 83% of patients gained weight (mean: 2.9 lbs; SD ± 4.5; *p* < 0.001)

Among 38 patients prescribed olanzapine, 29 had follow‐up (mean interval: 10 days; prior meds: 3.9; mean weight loss: 7.9 lbs; mean EGA: 15.2 weeks). At followup, HELP scores decreased by 14.6 (SD ± 11.6; *p* < 0.001), PUQE scores decreased by 3.4 (SD ± 3.5; *p* < 0.001), vomiting decreased from 6.8x to 2.7x/day (Δ 4.1; SD ± 4.9; *p* < 0.001), and 90% of patients gained weight (mean: 6.2 lbs; SD ± 5.9; *p* < 0.001)

Subgroup analysis of patients treated before 12 weeks (mirtazapine 25; olanzapine 10; mean EGA 10.8) showed similar benefit.

**Conclusion**: In patients with refractory HG, mirtazapine and olanzapine were associated with reduced nausea and vomiting and improved weight stabilization. These findings support further study of these agents as adjunctive therapies for severe HG.

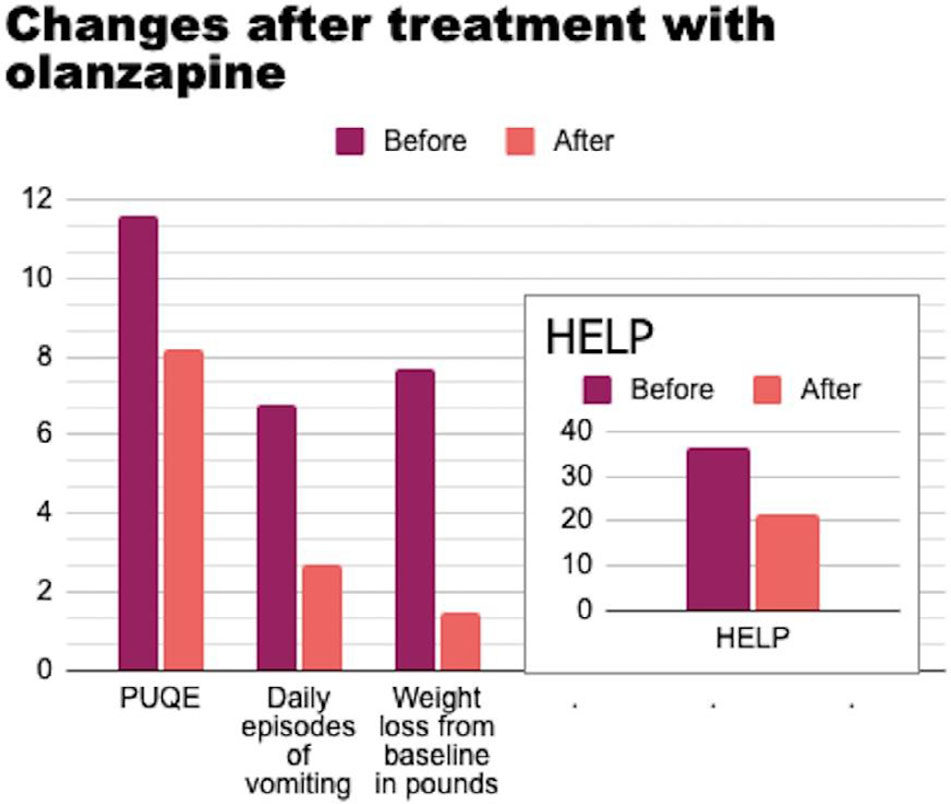

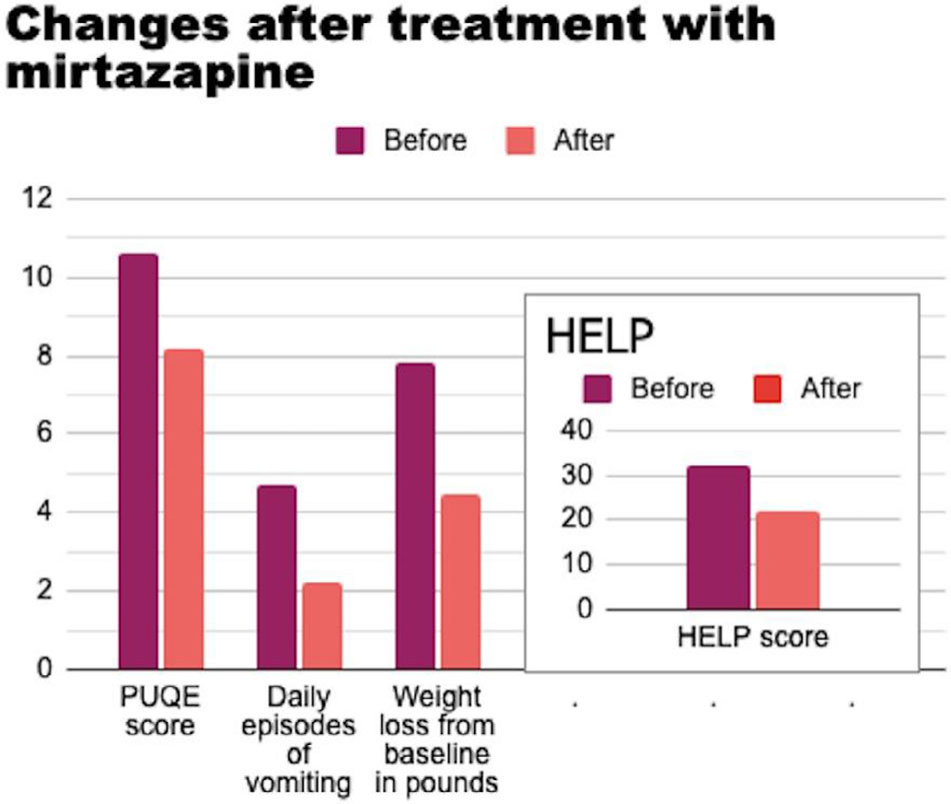

**10:30 AM – 12:00 PM**

## 722 | **Cerclage in PPROM: Should I Stay or Should I Go?**

Andrew Storm^1^; Gilad A. Gross^2^; Gary Dildy^1^; Tracy M. Tomlinson^1^

^1^Saint Louis University Division of Maternal Fetal Medicine, Saint Louis University, MO; ^2^SSM Health/St. Louis University School of Medicine, St. Louis, MO

**Objective**: The decision to remove or retain a cerclage in patients with preterm prelabor rupture of membranes (PPROM) without an active indication for delivery is controversial. We compared neonatal and maternal outcomes between patients with early (< = 24 hours) and deferred (> 24 hours) removal of a cerclage.

**Study Design**: Passive prospective cohort of 156 pregnancies with a cerclage in situ who presented between 24w0d and 34w0d with PPROM from 2009 to 2022, retrospectively analyzed. We assessed univariate associations of maternal and neonatal outcomes between those with early (< = 24 hours) and deferred (> 24 hours) cerclage removal after PPROM.

**Results**: 66 patients (42.3%) were in the early cohort and 90 (57.7%) were in the deferred cohort. Both had similar demographics, history, indications for cerclage, type of cerclage, and suture. Both had similar gestational age (GA) at placement (18.3w vs 18.9w, *p* = 0.27) and at the time of PPROM (28.9w vs 26.2w, *p* = 0.13). The early removal cohort had a higher GA at delivery (29.3w vs 28.0w, *p* = 0.029) and birthweight (1487g v 1202g, *p* = 0.001). The deferred removal cohort was associated with a higher rate of pregnancy prolongation ≥48 hours (44% vs 100%, *p* < 0.001) and ≥7 days (11% vs 62%, *p* < 0.001). The deferred cohort had a higher incidence of neonatal sepsis (16% vs 33%, *p* = 0.029) and bronchopulmonary dysplasia (16% vs 37%, *p* = 0.009). The rates of clinical chorioamnionitis (32 vs 43%, *p* = 0.18) and histopathologic chorioamnionitis (35% vs 52%, *p* = 0.60) were similar.

**Conclusion**: Management of PPROM with cerclage in situ is individualized depending on gestational age and specific patient factors with no consensus on optimal management. Our data suggest that while early removal was associated with a higher GA and birthweight at delivery, as well as a lower rate of neonatal sepsis and bronchopulmonary dysplasia, deferred removal was associated with a higher rate of pregnancy duration ≥ 48 hours and 7 days.

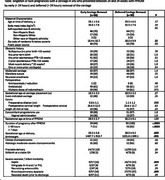

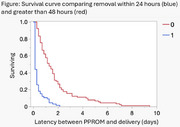

**10:30 AM – 12:00 PM**

## 723 | **Effect of** Low**‐Dose Aspirin Recommendations on Rates of Gestational Hypertension and Preterm Births**

Angela Kown^1^; Mariah Buzzell^2^; Ashley Zimmermann^3^; Gloria Khafif^4^; Jessica Gulati^4^; Sage Smith^1^; Yuzuru Anzai^3^

^1^University of Chicago, Chicago, IL; ^2^Northwell, Northwell Lenox Hill Hospital, NY; ^3^Northwell, New Hyde Park, NY; ^4^University of Chicago, University of Chicago, IL

**Objective**: To evaluate the impact of low‐dose aspirin recommendations on the rates of gestational hypertension and preterm birth, and to assess the association between gestational hypertension and preterm birth over time.

**Study Design**: A retrospective cohort analysis was conducted using CDC Natality data from 2011 to 2024. Multinomial logistic regression assessed the association between gestational hypertension and preterm birth rates over time. Adjusted odds ratios (aOR) with 95% confidence intervals (CIs) were calculated, with statistical significance set at *p* < 0.05. The analysis controlled for maternal BMI, age, in‐vitro fertilization (IVF), race, insurance type, gestational and pre‐gestational diabetes, hypertensive disorders of pregnancy, eclampsia, and inter‐pregnancy interval.

**Results**: Rates of gestational hypertension increased across low, moderate, and high‐risk groups during the study period. Additionally, rates of preterm birth in moderate and high‐risk groups decreased during the study period. However, adjusted odds ratios (aOR) for preterm birth among individuals with gestational hypertension demonstrated a steady decline from 2.824 in 2011 to 2.208 in 2024 (Table 1). Among high‐risk patients specifically, the decline was most pronounced and steadily decreased over time (Figure 1).

**Conclusion**: Following the USPSTF recommendation for aspirin administration to prevent early‐onset preeclampsia in 2014, preterm birth rates among patients with gestational hypertension have declined. This trend may indicate the efficacy of low dose aspirin on prevention of early onset gestational hypertension.

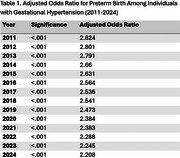

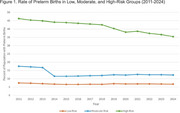

**10:30 AM – 12:00 PM**

## 724 | **Perinatal Outcomes in Patients With Pre‐eclampsia With Severe Features and Placental Abruption**

Anika Srinath^1^; Alesha M. White^2^; Deepa Ravindra^1^; Anastasia Kelley^1^; Jessica E. Pruszynski^1^; Elaine L. Duryea^1^

^1^University of Texas Southwestern Medical Center, Dallas, TX; ^2^University of Texas Southwestern Medical Center, Grand Prairie, TX

**Objective**: The objective of this study was to identify how maternal and neonatal outcomes differ amongst patients with pre‐eclampsia with severe features (SPE) depending on the presence or absence of placental abruption.

**Study Design**: This is a retrospective cohort study including patients diagnosed with SPE and placental abruption who delivered at a single institution between January 2019 and November 2024. Perinatal outcomes during delivery were compared between patients diagnosed with SPE who were also diagnosed with placental abruption to those with SPE alone. Patients diagnosed with SPE postpartum were excluded.

**Results**: Of 5056 deliveries complicated by SPE, 182 (4%) had both SPE and abruption. These patients were more likely to deliver via cesarean delivery (63% vs 42%, *p* < 0.001) and to experience a stillbirth (17% vs 1%, *p* < 0.001). Median estimated blood loss was increased (1250 (750–1500cc) vs 750 (500–1000cc), *p* < 0.001) and rate of transfusion (33% vs 14%, *p* < 0.001) higher in this cohort. If transfused, patients with SPE and abruption received fresh frozen plasma (FFP) and cryoprecipitate at higher rates (both *p* < 0.001). Neonates born to mothers with SPE and placental abruption had worse outcomes in all assessed neonatal outcomes including depressed Apgar scores, acidemia, NICU admission, and need for therapeutic hypothermia (all *p* < 0.05) (Table 2).

**Conclusion**: Patients with SPE and placental abruption have worse maternal and neonatal outcomes when compared to SPE alone. Providers should remain alert to the association of SPE with abruption and the subsequent possible perinatal outcomes.

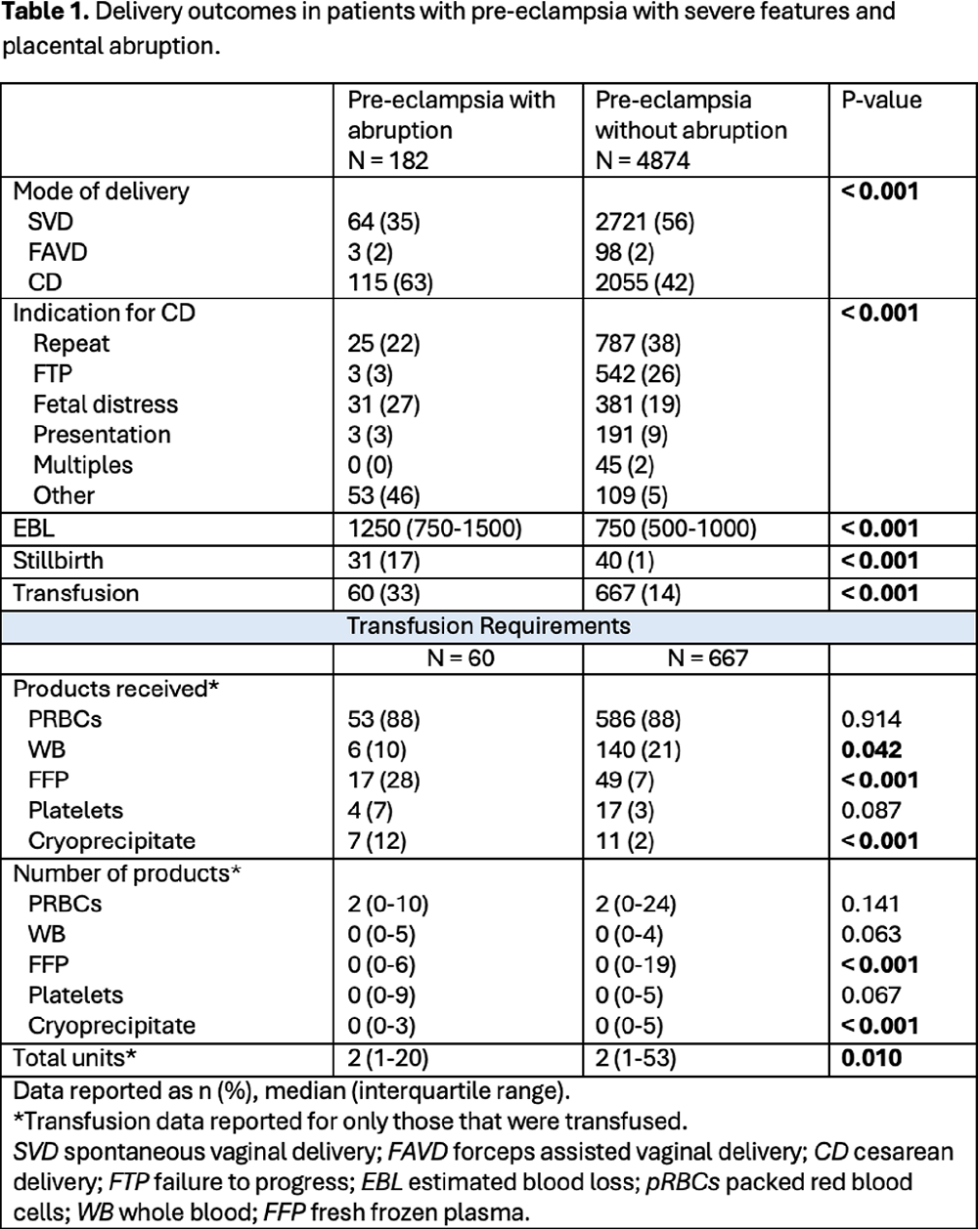

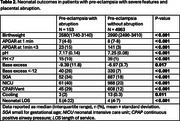

**10:30 AM – 12:00 PM**

## 725 | Psychosocial **Stressors and 30 Year Cardiovascular Risk Score for Individuals With Obstetric Risk Factors**

Lauren Keenan‐Devlin^1^; Chuhan Wu^1^; Alexa A. Freedman^2^; Linda M. Ernst^1^; Gregory E. Miller^3^; Ann EB Borders^4^

^1^Endeavor Health, Evanston, IL; ^2^Northwestern University, Chicago, IL; ^3^Northwestern University, Evanston, IL; ^4^Endeavor Health, Evanston Hospital, Evanston, IL

**Objective**: Obstetric (OB) risk factors including prior preterm birth and hypertension are linked to increased cardiovascular (CVD) risk, but it is unclear if stress compounds CVD risk associated with OB risk factors. We examined associations between psychosocial stress and projected 30‐year CVD risk for individuals with high and low OB risk.

**Study Design**: The prospective Stress, Pregnancy, and Health study collected stress measures during 2^nd^ and 3rd trimesters and documented clinical outcomes for 605 participants 2018–2023. High OB risk was defined by history of IUGR, preterm birth <37 wks, PPROM, or IUFD in a prior pregnancy, or current or prior hypertensive disorder in the prior or index pregnancy. Food insecurity, loneliness, financial hardship, and depressive symptoms were calculated from validated questionnaires. PREVENT 30‐year CVD risk score (percent probability of a first CVD event within 30yrs) was calculated with the AHA equation that uses age, systolic blood pressure, non‐HDL cholesterol, diabetes, smoking, and estimated glomerular filtration rate. Stress and PREVENT scores were z‐scored for comparability. Linear regressions within each OB‐risk group were adjusted for race/ethnicity and education/occupation.

**Results**: Among 469 participants, the high OB risk group (*n* = 148) had a higher proportion of Black participants (25.7 vs. 14.6%) and lower socioeconomic status. Mean PREVENT scores were significantly higher in the high‐risk group (0.40 ± 1.46 vs–0.18 ± 0.62). Within the high‐risk group, a 1‐SD increase in loneliness (β = 0.23, 0.003–0.45) and depressive symptoms (β = 0.23, 0.01–0.46) were significantly associated with corresponding increases in PREVENT scores. Food insecurity trended toward higher PREVENT scores (β = 0.19, −0.03–0.40). In the low‐risk group (*n* = 321), no stressors were associated with CVD risk score.

**Conclusion**: Loneliness and depressive symptoms were associated with increase in 30‐year CVD risk in high‐OB‐risk pregnancies. Psychosocial screening in prenatal care may identify opportunities to reduce long‐term health risk, particularly for individuals with obstetric risk factors.

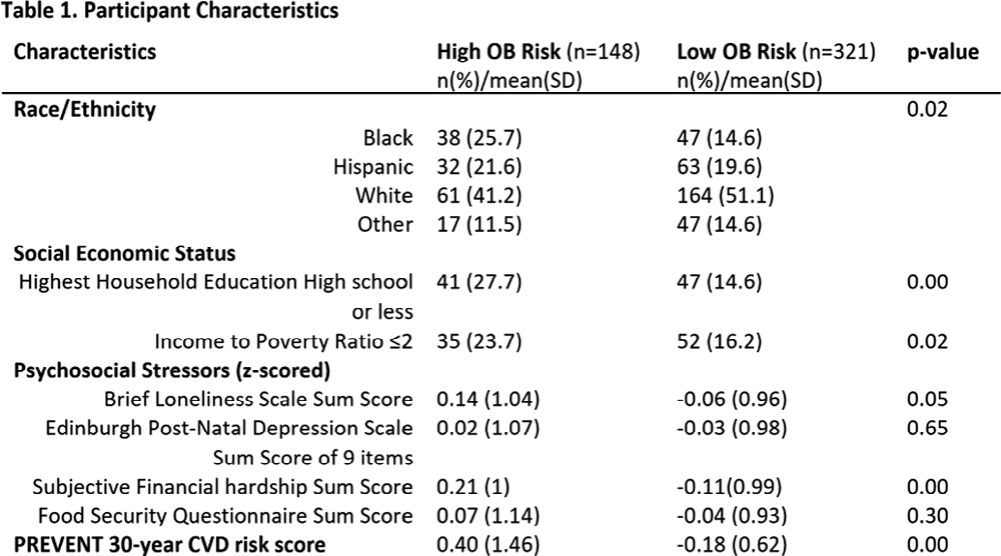

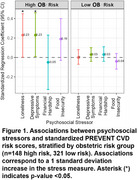

**10:30 AM – 12:00 PM**

## 726 | **Impact of Penicillin Allergy Labels on Maternal and Neonatal Healthcare Utilization and Clinical Outcomes**

Ann R. Tucker; Avery Bogart; Leena Choi; Elizabeth McNeer; Wei‐Qi Wei; Srushti Gangireddy; Jennifer L. Thompson; Cosby A. Stone

Vanderbilt University Medical Center, Nashville, TN

**Objective**: Penicillin allergy labels (PALs) are frequently reported in pregnancy but >90% are unverified. Unverified PALs lead to avoidance of first‐line antibiotics increasing the risk for adverse events. However, >95% of patients reporting PALs are penicillin tolerant when tested. We aimed to assess the impact of maternal PALs on maternal and neonatal healthcare utilization and clinical outcomes using a large population dataset.

**Study Design**: Retrospective cohort study included 75,236 maternal‐neonatal dyads at a major academic medical center with live births at 24 weeks or beyond from 2/1995–12/2024. Two groups were derived: Patients with PALs in the medical record and those without PALs documented in pregnancy. Clinical characteristics and outcomes were extracted. Using logistic regression, adjusted odds ratios (aOR) were calculated between cohorts and assessed for the following outcomes controlling for significant covariates as appropriate: maternal emergency (ER) visits during pregnancy or within 3 months postpartum, maternal hospital admissions excluding delivery during pregnancy or within 3 months postpartum, maternal ICU admission, maternal death, neonatal ER visits in the first 3 months of life, neonatal hospital readmission in the first 3 months of life, NICU admission and neonatal death. *p* < .05 was considered significant for all analyses.

**Results**: Maternal PALs significantly increased the odds of maternal ER admission (aOR 1.69), additional maternal hospital admission (aOR 1.66), maternal ICU admission (aOR 1.38) and maternal death (aOR 1.86) compared to those without PALs. For infants, maternal PALs significantly increased the odds of NICU admission (aOR 1.34) and neonatal death (aOR 1.38).

**Conclusion**: Maternal PALs increased maternal and neonatal healthcare utilization and adverse outcomes with higher odds of maternal ER admission, additional maternal hospital admission, maternal ICU admission, maternal death, NICU admission, and neonatal death. However, PALs are modifiable as >95% are penicillin tolerant when tested, suggesting a need for greater uptake of testing.

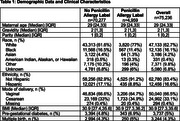

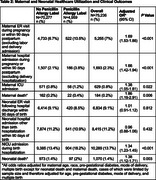

**10:30 AM – 12:00 PM**

## 727 | **Variation in Sink Water Temperature for Perineal Warm Compresses During Labor**

Anna E. Riegger^1^; Mariana Masteling^1^; Julia Erickson^2^; Jourdan E. Triebwasser^1^; Christopher X. Hong^1^

^1^University of Michigan, Ann Arbor, MI; ^2^University of Michigan, University of Michigan, MI

**Objective**: Perineal warm compresses during labor reduce the risk of obstetric anal sphincter injuries, yet clinical practice often deviates from standardized protocols. While prior studies used bedside water baths heated to ≥45°C, real‐world application typically involves warming washcloths using sink water, which may be inconsistent. This study aimed to characterize the maximum sink water temperature in labor rooms, time to reach maximum temperature, and variation across rooms to identify gaps in current practice and inform device design for standardization.

**Study Design**: We conducted a prospective observational study of 30 labor rooms within a tertiary obstetric hospital, each with an in‐room sink routinely used to prepare warm compresses. After a cold water washout, the hot water faucet was opened at maximal flow. Real‐time faucet water temperature was measured using a thermal camera (FLIR E54) until stabilization (Figure 1). Time–temperature profiles were analyzed to determine maximum steady‐state temperature (<1% further change), time to maximum temperature, and time to ≥45°C. Water volume was calculated using manufacturer‐reported flow rate.

**Results**: Significant variation in time–temperature profiles was observed across the 30 rooms (Figure 2). The median maximum steady‐state water temperature was 45.4°C (IQR 44.6–46.2), which required a median of 77 seconds to reach (IQR 66–84). Of the 30 rooms, 19 reached a maximum steady‐state temperature of ≥45°C, requiring a median of 62 seconds (IQR 51–73). Based on the time to reach either 45°C or steady‐state temperature, the median volume of water used was estimated at 5.8–7.3 liters, respectively.

**Conclusion**: While most rooms reached the 45°C threshold, substantial variability in time to temperature was observed. More than one minute of continuous flow was typically required, posing practical limitations in clinical settings. These findings highlight the need for accessible, consistent heat sources to enable standardized implementation of this evidence‐based practice.

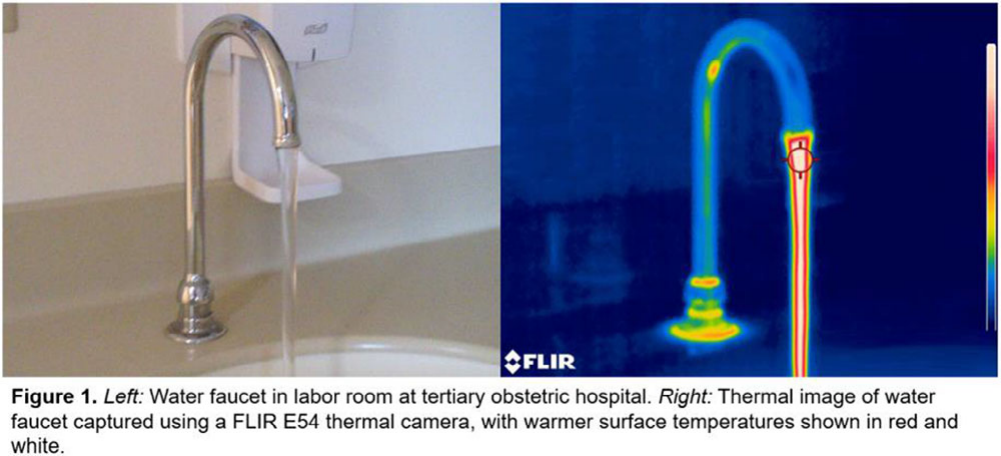

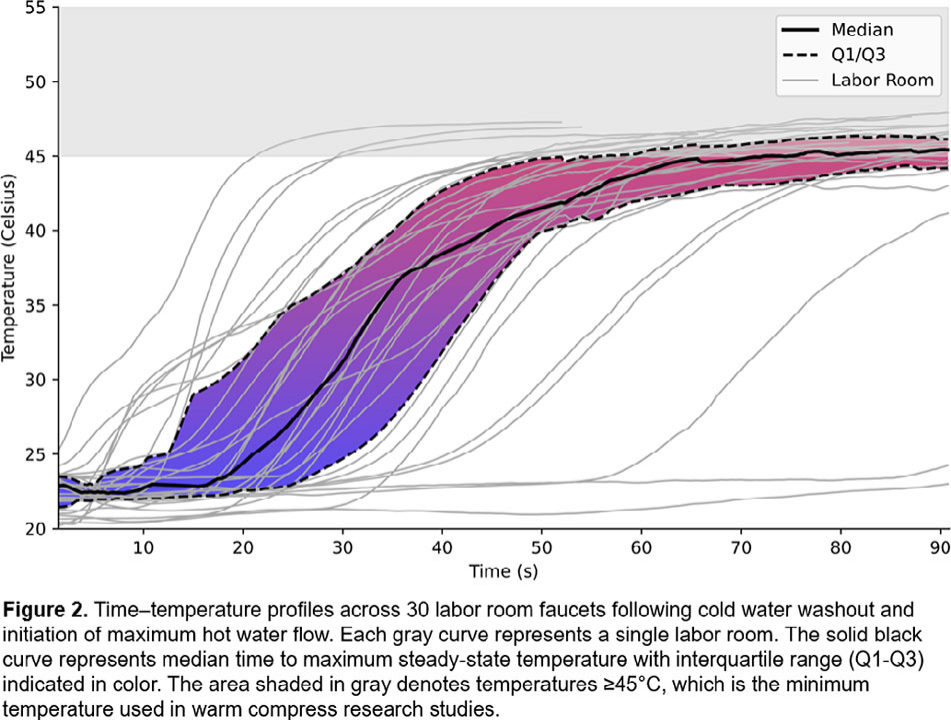

**10:30 AM – 12:00 PM**

## 728 | **Risk** Factors **and Perinatal Outcomes in Patients with Placenta Previa Delivered for Severe Antepartum Bleeding**

Anthony E. Melendez Torres^1^; Adwoa A. Baffoe‐Bonnie^2^; Kathleen Chang^2^; Joyce Liu^2^; Sara I. Jones^2^; Jennifer B. Gilner^2^; Sarah K. Dotters‐Katz^3^

^1^Division of Maternal Fetal Medicine, Department of Obstetrics and Gynecology, Duke University, Durham, NC; ^2^Duke University School of Medicine, Durham, NC; ^3^Division of Maternal Fetal Medicine, Department of Obstetrics and Gynecology, Duke University, Duke University/Durham, NC

**Objective**: Data on risk factors associated with delivery for severe bleeding and perinatal outcomes in patients with placenta previa remains lacking. We investigated potential risk factors associated with need for delivery due to severe bleeding and related perinatal outcomes.

**Study Design**: Retrospective cohort study of pregnant patients with ultrasound‐confirmed placenta previa, delivered at a single tertiary health system from 6/2013–3/2025. Resolved previa or low‐lying placenta were excluded. Primary outcome was risk factors associated with delivery for severe bleeding. Secondary analysis of obstetric and neonatal outcomes also conducted. Obstetric outcomes included composite of cesarean hysterectomy, ICU admission, postpartum hemorrhage (PPH), and blood transfusion, EBL >1.5 and 2 L, and preterm birth. Neonatal outcomes included composite of mechanical ventilation, respiratory distress syndrome (RDS), sepsis, seizure, intraventricular hemorrhage (IVH), and neonatal death, NICU admission and length of stay. Bivariate statistics used to analyze data. Regression models used to control for confounders.

**Results**: Of 247 included patients, 79 (32%) were delivered for severe bleeding. Demographics and clinical characteristics were similar between cohorts. No individual factors were identified to be independently associated with an increased risk of delivery for severe bleeding (Table 1). However, after controlling for confounders, those delivered for severe bleeding had higher odds of adverse composite delivery outcome, PPH, EBL >1.5 and >2 L, blood transfusion, and preterm birth (Table 2). No significant differences seen in neonatal outcomes (Table 2).

**Conclusion**: No independent risk factors associated with delivery for refractory bleeding were identified. Patients delivered for refractory bleeding had significantly higher odds of adverse maternal and delivery outcomes without differences in neonatal outcomes.

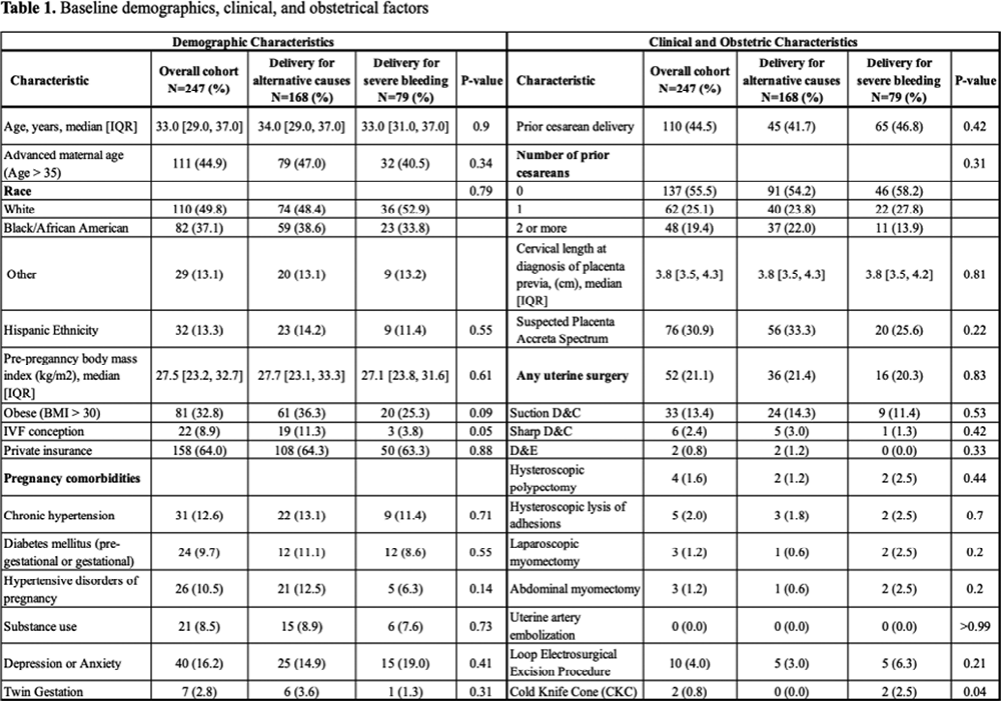

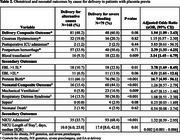

**10:30 AM – 12:00 PM**

## 729 | Assessment **of Gestational age Specific Severe Maternal Morbidity Among Patients With Pre‐Eclampsia With Severe Features**

Ariane C. Youssefzadeh^1^; Yadira L. Bribiesca Leon^2^; Zaira Chavez Jimenez^2^; Fay Pon^2^; Carolyn Rocha^2^; Brendan H. Grubbs^1^; Marc H. Incerpi^1^; Joseph G. Ouzounian^1^; Koji Matsuo^3^

^1^Division of Maternal‐Fetal Medicine, Department of Obstetrics and Gynecology, University of Southern California, Los Angeles, CA; ^2^Keck School of Medicine of USC, University of Southern California, Los Angeles, CA; ^3^Division of Gynecologic Oncology, Department of Obstetrics and Gynecology, University of Southern California, Los Angeles, CA

**Objective**: To *(i)* describe the temporal trend of preeclampsia with severe features (SPE) by gestational age at delivery (≥34 weeks, ≥28/<34 weeks, and <28 weeks) and *(ii)* compare severe maternal morbidity (SMM) among patients with SPE across these groups.

**Study Design**: This was a cross‐sectional study of Healthcare Cost and Utilization Project's National Inpatient Sample including 21,773,049 deliveries. All hospital deliveries with a diagnosis of SPE from 2016–2022 were included. Exposure was gestational age at delivery. The outcome measured was SMM per the Center for Disease Control and Prevention. A multivariable generalized linear model was created, adjusting for the following a priori selected factors: maternal age, race and ethnicity, maternal comorbidities (systemic lupus erythematosus, pregestational diabetes, obesity), prior uterine scar, placental abruption, and cesarean delivery.

**Results**: Among the 638,460 patients with SPE, 533,360 (83.5%) delivered at ≥34 weeks, 89,845 (14.1%) delivered at ≥28/<34 weeks, and 15,255 (2.4%) delivered at <28 weeks. The incidence of SPE increased during the study period among all three groups (Figure). In a multivariable analysis, compared to patients with SPE delivered at ≥34 weeks, patients with SPE who delivered at ≥28/<34 weeks had higher composite SMM, cardiopulmonary indicators of SMM, and multiple individual indicators including pulmonary edema, myocardial infarction, ventilation requirement, eclampsia, and puerperal cerebrovascular disorders (all, adjusted‐rate ratio >1.5; Table). Likewise, compared to patients with SPE delivered at ≥34 weeks, patients with SPE who delivered at ≤28 weeks also had higher composite SMM, cardiopulmonary indicators of SMM, and multiple individual indicators including pulmonary edema, renal failure, myocardial infarction, ventilation requirement, and eclampsia (all, adjusted‐rate ratio >2.5, Table).

**Conclusion**: This study demonstrates an on‐going rise in SPE deliveries including those remote from term. This finding is clinically compelling and warrants further investigation.

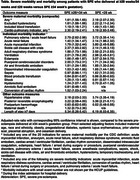

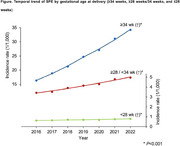

**10:30 AM – 12:00 PM**

## 730 | **Beyond** Blood **Loss: Hemoglobin‐Informed Thresholds to Optimize Peripartum Transfusion Decisions**

Arielle J. Higgs^1^; Sara Case^1^; Jenifer Fahey^1^; Courtney Townsel^2^

^1^University of Maryland, Baltimore, MD; ^2^University of Maryland School of Medicine, University of Maryland School of Medicine, MD

**Objective**: Postpartum hemorrhage (PPH) remains a leading cause of maternal mortality worldwide. The American College of Obstetricians and Gynecologists defines PPH as blood loss ≥1000 mL, a threshold that commonly prompts clinical intervention, including consideration of transfusion. However, up to 40% of pregnant patients are anemic at term due to hemodilution and increased iron demands. These patients have reduced oxygen‐carrying capacity and may require transfusion at lower blood loss volumes. Despite this, current protocols use universal blood loss thresholds, which underestimate transfusion need in vulnerable subgroups. This study evaluated diagnostic performance of pre‐delivery hemoglobin in predicting peripartum transfusion and aimed to identify optimal hemoglobin thresholds for clinical decision‐making.

**Study Design**: This retrospective cohort study analyzed delivery data from 2019–2024 at a single tertiary center. After data cleaning, 8379 patients with blood loss, pre‐delivery hemoglobin, and transfusion data were analyzed. Patients were stratified into two groups: Group 1 (Hgb < 11 g/dL, *n* = 3504) and Group 2 (Hgb ≥11 g/dL, *n* = 4875). ROC curves were generated for each group to evaluate EBL as a transfusion predictor. Optimal thresholds were identified using the Youden Index, and performance metrics were calculated at EBL cutoffs (500–2000 mL).

**Results**: Group 1 had an AUC of 0.814 for predicting transfusion, with an optimal blood loss threshold of 732 mL. Group 2 had an AUC of 0.877 and a threshold of 948 mL. Transfusion occurred in 14.6% of Group 1 and 3.8% of Group 2. At 1000 mL, Group 1 had lower sensitivity (0.55) but higher PPV (0.50) than Group 2 (sensitivity 0.73, PPV 0.23).

**Conclusion**: Stratifying transfusion thresholds by hemoglobin improves sensitivity without sacrificing specificity. These findings support individualized transfusion protocols as fixed blood loss thresholds may inadequately reflect transfusion need in anemic patients. Hemoglobin‐informed thresholds may optimize clinical response to peripartum blood loss before conventional PPH criteria are met.

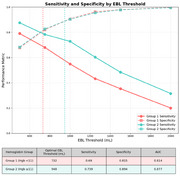

**10:30 AM – 12:00 PM**

## 731 | **Early** Gestational **Weight Gain and Risk of Hypertensive Disorders of Pregnancy**

Arlin Delgado^1^; Kaitlyn E. James^1^; Jacqueline Maya^2^; Camille Powe^1^; Lydia L. Shook^3^; Mark A. Clapp^4^

^1^Department of Obstetrics and Gynecology, MassGeneral Brigham; Division of Maternal‐Fetal Medicine; Harvard Medical School, Boston, MA; ^2^Department of Pediatrics, Massachusetts General Hospital, Boston, MA; ^3^Department of Obstetrics and Gynecology, MassGeneral Brigham; Division of Maternal‐Fetal Medicine; Harvard Medical School, Massachusetts General Hospital/Boston, MA; ^4^Department of Obstetrics and Gynecology, Mass General Brigham, Massachusetts General Hospital, MA

**Objective**: There is limited data on early gestational weight gain (GWG) as a modifiable risk factor to reduce the incidence of hypertensive disorders of pregnancy (HDP). We aimed to evaluate the association between GWG by 24 weeks and the development of HDP.

**Study Design**: This was a retrospective cohort study of singleton pregnancies that delivered at term between 2016 and 2024 at a large health system. The primary exposure was total GWG between preconception and 24 weeks of gestation. Alternately, we also examined the rate of GWG per week. The primary outcome, HDP, was a composite of gestational hypertension and preeclampsia with and without severe features. We used logistic regression to assess the association between GWG and HDP, adjusting for maternal age, race, insurance, pre‐pregnancy BMI, nulliparity, pre‐existing diabetes, and chronic hypertension.

**Results**: Among 29,989 eligible pregnancies, 2446 (8.1%) individuals were diagnosed with HDP. GWG by 24 weeks was significantly associated with increased odds of HDP (aOR 1.04; 95% CI 1.03, 1.05). Figure 1 shows GWG trends across gestational age intervals stratified by prepregnancy BMI, demonstrating that those who developed HDP gained more weight at each time interval than those who did not. Increasing rate of GWG in the 1st Trimester was associated with 90% increased odds of HDP (aOR 1.90 per kg/wk of GWG; 95% CI 1.53, 2.35), and exceeding the recommended rate of GWG by the Institute of Medicine in the 2nd Trimester was associated with 19% increased odds of HDP (aOR 1.19 per kg/wk of GWG; 95% CI: 1.08–1.31). Table 1 lists the adjusted and unadjusted odds ratios for GWG at trimester‐specific time points and rates of GWG in the two cohorts.

**Conclusion**: Higher GWG in the first and second trimesters was linked to increased odds of HDP, highlighting potential windows for early intervention.

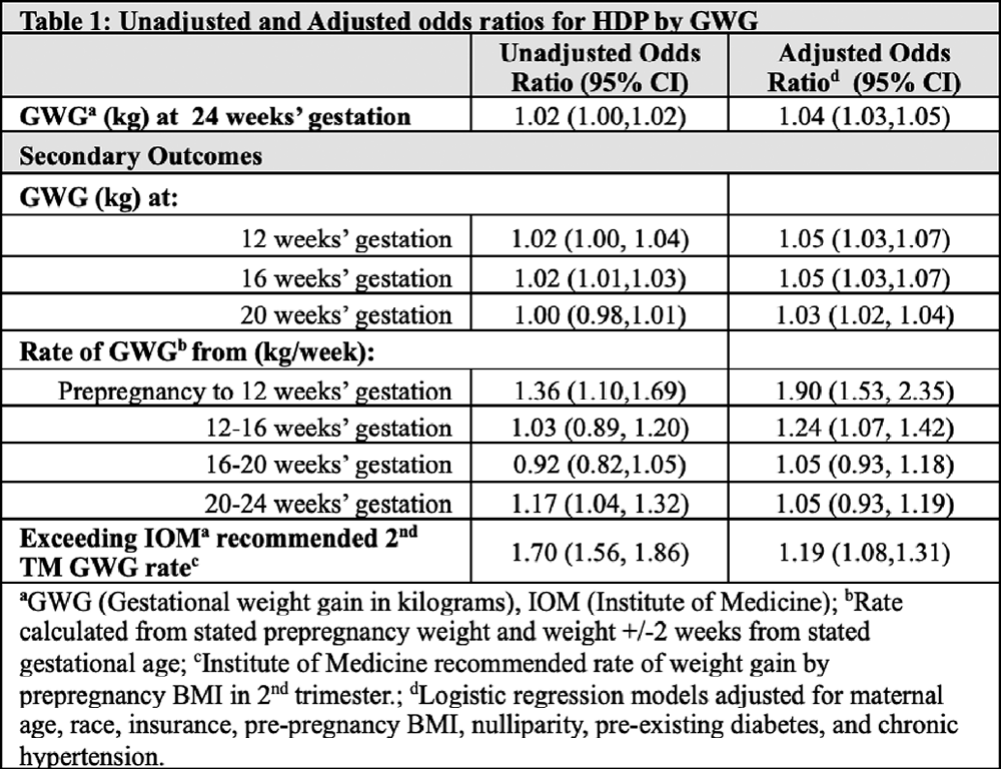

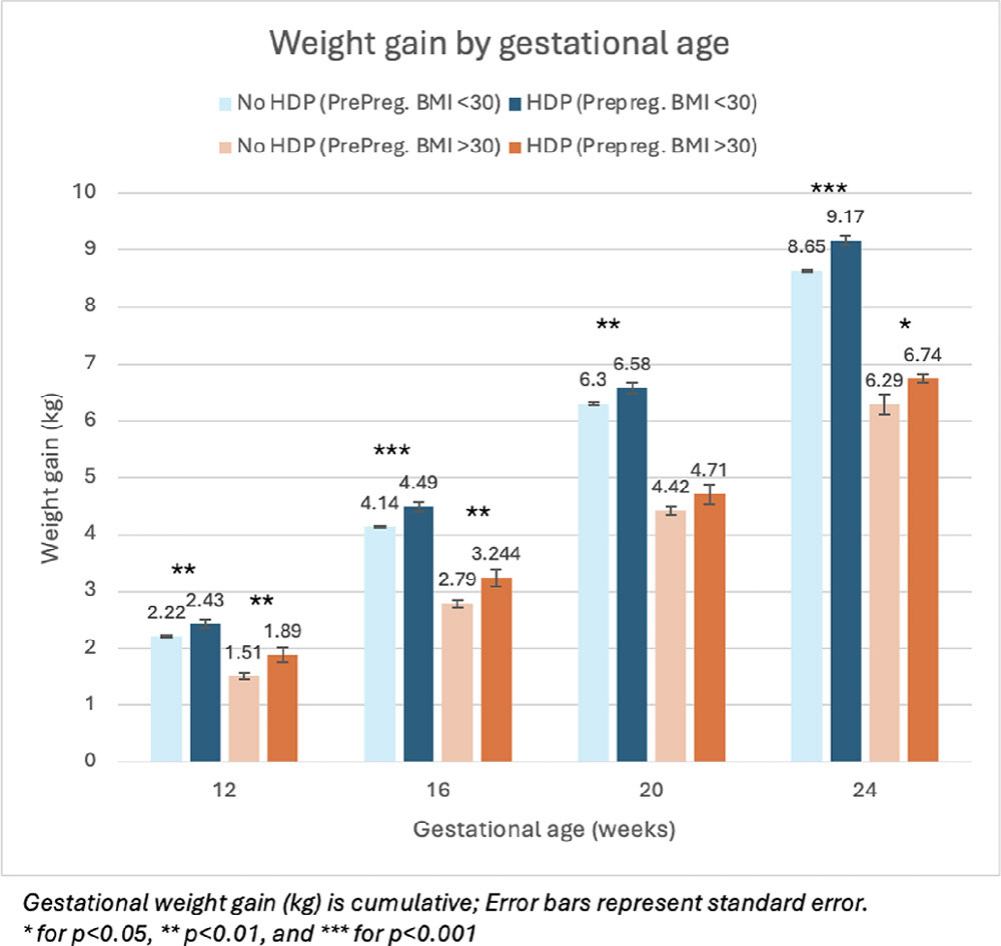

**10:30 AM – 12:00 PM**

## 732 | Therapeutic **Vasopressor Use for Postspinal Hypotension in High‐Risk Cesarean Delivery: A Systematic Review and Meta‐Analysis**

Arya Babul^1^; Sohi Ashraf^2^; Leanne Free^3^; Jyoti Desai^4^; Momina Hussain^5^; Najib Babul^6^

^1^West Career & Technical Academy, Las Vegas, NV; ^2^MountainView Hospital, Las Vegas, NV; ^3^Kirk Kerkorian School of Medicine at UNLV, Las Vegas, NV; ^4^Department of Gynecologic Surgery & Obstetrics, Kirk Kerkorian School of Medicine at UNLV, Las Vegas, NV; ^5^Chinese Academy of Tropical Agricultural Sciences, Sanya City, Hainan; ^6^Quadra Therapeutics, Las Vegas, NV

**Objective**: Postspinal hypotension (PSH) is a frequent complication of neuraxial anesthesia during cesarean delivery (CD), particularly in high‐risk pregnancies. Vasopressors (VPs) are commonly used to manage PSH, but their overall efficacy after the occurrence of PSH, particularly in high‐risk patients, remains uncertain. This study compared norepinephrine (NE), phenylephrine (PE), and ephedrine (EP) for the therapeutic management of PSH, excluding prophylactic VP use.

**Study Design**: After PROSPERO registration (CRD420251050321), we conducted a systematic review and meta‐analysis of randomized controlled trials (RCTs) comparing VPs for the treatment (not prevention) of PSH in high‐risk CD with PRISMA guidelines. The primary outcome was resolution of PSH, defined as restoration of SBP to ≥90–100% of baseline after VPs. Secondary outcomes included (i) comparison of multiple VPs vs. placebo/control, NE vs. PE, and EP vs. PE, and (ii) neonatal and other maternal effects. Pairwise meta‐analyses for direct comparison were done using a random‐effects model, expressed as relative risk (RR) with 95% CI. Heterogeneity, subgroup analysis, data quality, sensitivity analysis and publication bias were assessed.

**Results**: Nineteen RCTs comprising 1715 patients were included. Heterogeneity across studies was low (I^2^ = 6.03%). The pooled RR for PSH resolution with VPs was 1.18 (95% CI: 0.90–1.55), a non‐significant trend favoring treatment. Subgroup analysis showed a statistically significant benefit when multiple VPs were compared with placebo (RR = 1.24, 95% CI: 1.01–1.53), while direct comparisons—NE vs. PE and EP vs. PE—showed favorable trends without reaching significance.

**Conclusion**: Despite positive signals in specific subgroups, the overall pooled effect of VPs for therapeutic management of PSH was equivocal. This study purposely focused on direct drug comparisons rather than the broader and more controversial question of VP use vs. no treatment. The findings highlight clinical equipoise and the need for further high‐quality trials to identify optimal VP selection for the management of PSH after onset in high‐risk CD.

**10:30 AM – 12:00 PM**

## 733 | Development **of a Clinical Scoring System to Rule Out Pulmonary Embolism in Pregnancy**

Asaf Romano^1^; Chen Ben David^2^; Nitzan Dana Sela^3^; Hagit Itzhak Kedem^4^; Gil Zeevi^1^; Karin Edut^5^; Suzan Abd Elgani^6^; Bar Rosh^7^; Solt Ido^8^; Yaniv Zipori^9^

^1^Helen Schneider Hospital for Women, Rabin Medical Center, Petah Tikva, HaMerkaz; ^2^Obstetrics and Gynecology department, Rambam health care campus, Haifa, Israel, Haifa, HaZafon; ^3^Obstetrics and Gynecology department, Haemek medical center, Haemek medical center, Afula, HaZafon; ^4^Medical faculty, Technion University, Medical faculty, Technion University, Haifa, HaZafon; ^5^Department of Obstetrics and Gynecology, Mayaney Hayeshua Medical Center, Bnei Barak; Israel, Department of Obstetrics and Gynecology, Mayaney Hayeshua Medical Center, Bnei Barak; Israel, HaMerkaz; ^6^Obstetrics and Gynecology, Haemek medical center, Obstetrics and Gynecology, Haemek medical center, HaZafon; ^7^Department of Obstetric and Gynecology, Rambam Health Care Campus, Rambam Health Care Campus, HaZafon; ^8^Rambam Health Care Campus, haifa, Hefa; ^9^Rambam Health Care campus, Haifa, HaZafon

**Objective**: To identify clinical predictors of pulmonary embolism (PE) in pregnant and postpartum women and develop a simple clinical risk scoring tool to stratify PE probability and reduce unnecessary imaging.

**Study Design**: We conducted a retrospective multicenter cohort study across three university‐affiliated hospitals (2013–2023). All pregnant or postpartum women evaluated for suspected PE. Demographics, clinical data, and outcomes were collected. Univariate and multivariate logistic regression analyses identified independent predictors. A weighted scoring system was constructed based on adjusted odds ratios (ORs), with cutoffs optimized to prioritize negative predictive value (NPV). ROC analysis was used to assess model performance.

**Results**: Among 815 women evaluated for PE, 42 (5.2%) were diagnosed. Figure 1, demonstrates the multivariate regression model. The following variables were independently associated with PE: D‐dimer ≥1500 ng/mL (OR 21.6, 95% CI 2.7–170.6), heart rate >100 bpm (OR 3.51, 95% CI 1.63–7.58), diabetes in pregnancy (OR 4.16, 95% CI 1.65–10.5), thrombophilia (OR 4.56, 95% CI 1.64–12.7), and infectious state (OR 4.06, 95% CI 1.41–11.65). Based on these findings, the Pulmonary Embolism in Pregnancy Scoring Index (PEPSI) was created (range 0–10 points). A score of ≤2 identified low‐risk patients, with 82% specificity, 98.9% negative predictive value (NPV) and 16.6% positive predictive value (PPV). The the PPV for patients with a score of ≥6 reached 56.7%. The area under the ROC curve (AUC) was 0.887. As a result, we suggested to stratify patients into low (0–2), intermediate (3–5), and high (6–10) risk groups, with corresponding recommendations ranging from observation to immediate imaging and treatment initiation (table 1).

**Conclusion**: The *PEPSI* score provides a clinically useful tool to identify pregnant patients at low risk for PE who may avoid unnecessary imaging. Further validation in prospective cohorts is warranted.

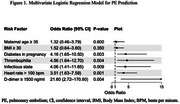

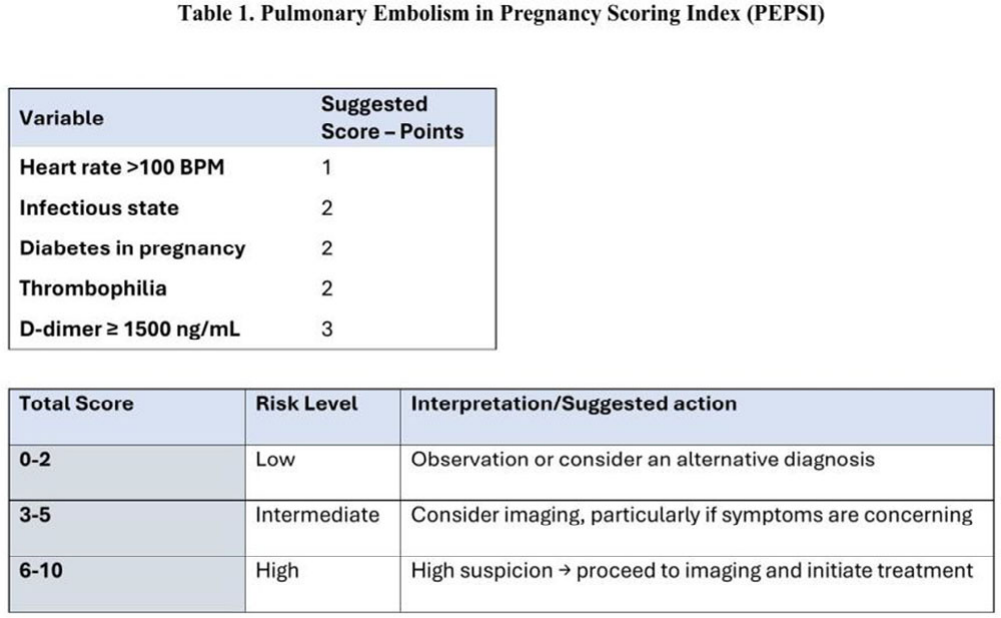

**10:30 AM – 12:00 PM**

## 734 | **Perinatal Autopsy Rates in a Contemporary U.S. Cohort**

Ashley Boerrigter^1^; Emily A. DeFranco^2^

^1^University of Kentucky, Lexington, KY; ^2^University of Kentucky, University of Kentucky/Lexington, KY

**Objective**: To assess the change in rate of perinatal autopsy after publication of a sentinel study in April 2017 by Page et al. which recommended inclusion of autopsy as a universal component of postmortem examination after stillbirth.

**Study Design**: National Vital Statistics System fetal death data was used to create a retrospective cohort of all fetal deaths ≥20 weeks’ gestation in the 50 United States and District of Columbia from 2014–2021. The primary outcome for analysis of this cohort was the percentage of stillbirths in which a perinatal autopsy was obtained. The exposure was time period, pre and post the study publication. Stillbirths during the year 2017 were excluded to allow time for adoption of recommendations.

**Results**: There were 180,520 stillbirths reported in the US during the study period, including stillbirths that occurred during 2017. Autopsy was obtained in 34,375 (19%) of stillbirths overall. The autopsy rate was 18.7% in the pre‐publication epoch, 2014–2016 (*n* = 13,361) and 19.1% in the later epoch 2018–2021 (*n* = 16,488), *p* = 0.019.

**Conclusion**: There was a persistently low rate of autopsy after stillbirth in the U.S. despite national society recommendations for universal perinatal autopsy. Although we identified a statistically significant increase from 18.7% to 19.1% after the guideline publication, this is likely not clinically significant. This study highlights a disconnect between national recommendations and clinical implementation, emphasizing the importance of continuing to identify and address barriers to autopsy consent and utilization.

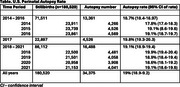

**10:30 AM – 12:00 PM**

## 735 | Performance **of Diagnostic Algorithms for Identification of Fetal Malformations Using Perinatal Claims Data**

Ashley Markowski^1^; Elisabeth Adkins^1^; Alexandra Sundermann^2^; Ashley A. Leech^2^; Andrew D. Wiese^2^; Margaret A. Adgent^2^; Amelie Pham^1^; Carlos G. Grijalva^2^; Sarah O. Osmundson^2^

^1^Vanderbilt University Medical Center, Nashville, TN; ^2^Vanderbilt University Medical Center, Vanderbilt University Medical Center, TN

**Objective**: Accurate identification of fetal anomalies (FAs) is essential for examining the effects of medication and environmental exposures in pregnancy. We aimed to determine the positive predictive value (PPV) of diagnostic algorithms using claims data for identifying births with common FAs.

**Study Design**: Among births >20 week's gestation occurring between 1/1/2018 and 1/31/2025 at a single medical center, we identified potential FAs using an algorithm based on ICD10 coded diagnoses (Table 1) in either the maternal or infant record and included both inpatient and outpatient encounters. Multiple gestations were excluded. Among all records identified with potential FAs, we randomly selected up to 100 records from the 8 most common malformations for structured review. Two researchers with expertise in neonatology and obstetrics reviewed both maternal and infant medical records to determine if FAs were present. Potential FA cases with discordant reviews were reviewed by a third reviewer who made final adjudications. Using the medical records review‐based decisions as reference, positive predictive values and 95% confidence intervals (CI) were calculated overall and for each malformation.

**Results**: During the study period, 29,324 women experienced 33,939 singleton births including 4426 (13.0%) infants with at least 1 potential FA based on the diagnostic algorithm. Of these infants, 1179 (26.7%) infants had multiple potential FAs. We reviewed records from 727 infants and confirmed 560 malformations for a PPV of 77.2% for the diagnostic algorithm for the presence of any malformation (95% CI 74.1–80.1). The most common FAs were septal cardiac defects (51.9%) and severe cardiac defects (15.4%) followed by microcephaly (3.0%). Individual malformations with a PPV over 80% for the diagnostic algorithm included severe heart defects, cleft lip and palate, abdominal wall defects, and spina bifida (Table 2).

**Conclusion**: Some major congenital malformations can be identified using diagnostic codes with reasonable accuracy. These estimates can inform subsequent studies of exposures or outcomes of FA.

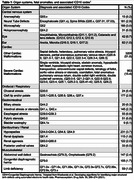

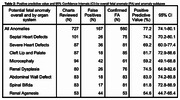

**10:30 AM – 12:00 PM**

## 736 | **The** Impact **of Implementation of a Universal Low‐Dose Aspirin Protocol**

Ashley Shea^1^; Christina T. Blanchard^1^; Annkay Alexander^2^; Sima Baalbaki^2^; Andi Farley^2^; Kaitlyn P. Casper^3^; Akila Subramaniam^1^

^1^Center for Research in Women's Health, University of Alabama at Birmingham, Birmingham, AL; ^2^Department of Obstetrics and Gynecology, University of Alabama at Birmingham, Birmingham, AL; ^3^University of Utah, Salt Lake City, UT

**Objective**: Low dose aspirin (LDA) is recommended for preeclampsia prophylaxis in high‐risk women, but uptake based on this risk‐based approach is poor nationally, despite attempted quality improvement (QI) and educational tools. We sought to improve LDA use through implementation of universal LDA.

**Study Design**: We performed a QI project transitioning from risk‐based LDA to universal LDA (LDA for all pregnant patients) receiving prenatal care from 4/2024–6/2025. Baseline data were collected from 4–6/2024 with transition to universal LDA on 7/1/2024. Starting at baseline, all patients were asked at each visit about LDA use, number of missed doses, and if LDA was recommended by their provider; all responses were documented in the electronic medical record (EMR). Providers were notified of these EMR changes and the transition to universal LDA; staff education was provided quarterly along with performance reports. The primary outcome was overall LDA use depicted by a monthly run chart. Secondary analysis was limited to those satisfying risk‐based LDA criteria. Quarterly outcomes in this group were compared using *p*‐values for trend.

**Results**: Between 4/2024–6/2025,13,756 individuals met inclusion. At baseline, 27.0% of patients reported LDA use despite 66.3% meeting risk‐based criteria; by the end of the QI study period, 64.9% reported LDA use with 57.1% meeting risk‐based criteria (*p* < 0.001) (Figure 1). Amongst those that met risk‐based criteria, LDA use increased (baseline 32.5% to Q4 62.3%, *p* < 0.001); both in those meeting criteria by high‐risk factors and moderate‐risk factors alone (*p* < 0.001) (Figure 2). In addition, there was an increase in LDA compliance (those reporting “zero” when asked how many doses were missed in the last 14 days, baseline 58.9% to Q4 66.2%, *p* < 0.001) and patient recall of provider recommendation if they were not taking LDA (baseline 4.3% to Q4 22.5%, *p* < 0.001).

**Conclusion**: A universal recommendation for LDA increased LDA use and compliance in our total population and in those meeting risk‐based criteria. Given the improved uptake of LDA, improvement in clinical outcomes should be evaluated.

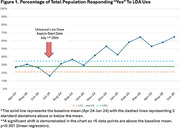

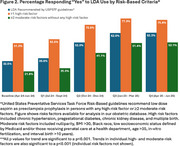

**10:30 AM – 12:00 PM**

## 737 | **Severe** Maternal **Morbidity Among Individuals with Cancer in Pregnancy and Radiation Treatment History**

Asmita Gathoo^1^; Yanling Dong^2^; Alexandra D. Forrest^2^; Marika Toscano^2^; Arthur Jason Vaught^2^; Shriddha Nayak^2^

^1^Johns Hopkins University School of Medicine, Johns Hopkins University/Baltimore, MD; ^2^Johns Hopkins University, Baltimore, MD

**Objective**: The study objective was to determine the odds of severe maternal morbidity (SMM) in pregnant individuals with cancer requiring radiation treatment pre‐pregnancy compared to those without radiation treatment history.

**Study Design**: This population‐based, retrospective cohort study queried TriNetX, a research network containing de‐identified electronic health data from over 155 million patients. Inclusion criteria were pregnant subjects 12–55 years diagnosed with cancer within one year prior to pregnancy, from 1/2015 to 8/2025. Two cohorts were derived: subjects with and without radiation treatment within one year prior to pregnancy. Cohorts were propensity score matched 1:1 on: age, race, ethnicity, diabetes, hypertension, hyperlipidemia, obesity, asthma, tobacco use, mental health disorder, chronic kidney disease, connective tissue disorder, cancer stage, surgery and chemotherapy. SMM was defined using the CDC's 21 indicators and corresponding codes. The primary outcome was adjusted odds ratio (aOR) of composite SMM occurring in antepartum or postpartum periods (up to one year from pregnancy diagnosis). Secondary outcomes were aOR of SMM individual indicators. All analyses were conducted 8/2025 using the TriNetX platform.

**Results**: After propensity score matching, subjects with cancer in pregnancy with and without radiation treatment pre‐pregnancy (*n* = 1065 for both) were analyzed (Table 1). Pregnant individuals with cancer and pre‐pregnancy radiation history had higher odds of composite SMM (prevalence 26.0% versus 11.6%, aOR 2.89 (95% confidence interval (CI) 2.25–3.73)) compared to those without radiation history (Table 2). Air and thrombotic embolism emerged as the indicator with the highest odds (aOR 3.13 (95% CI 1.82–5.38)) (Table 2).

**Conclusion**: A history of radiation oncology treatment within one year prior to pregnancy was associated with over 2.5 times increased odds of composite SMM. Further research is needed to explore the effect of radiation treatment history on the increased burden of maternal morbidity among patients with cancer in pregnancy.

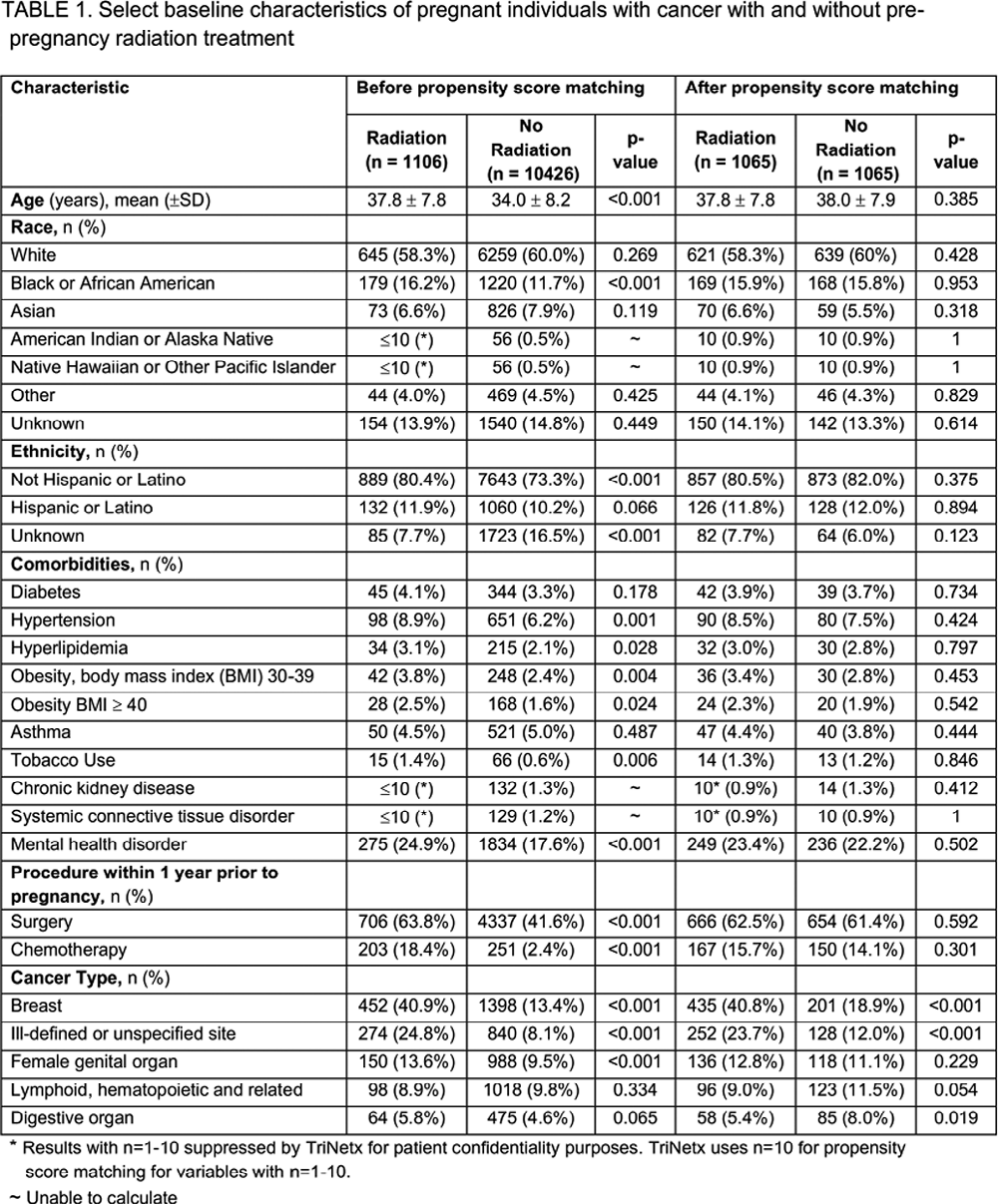

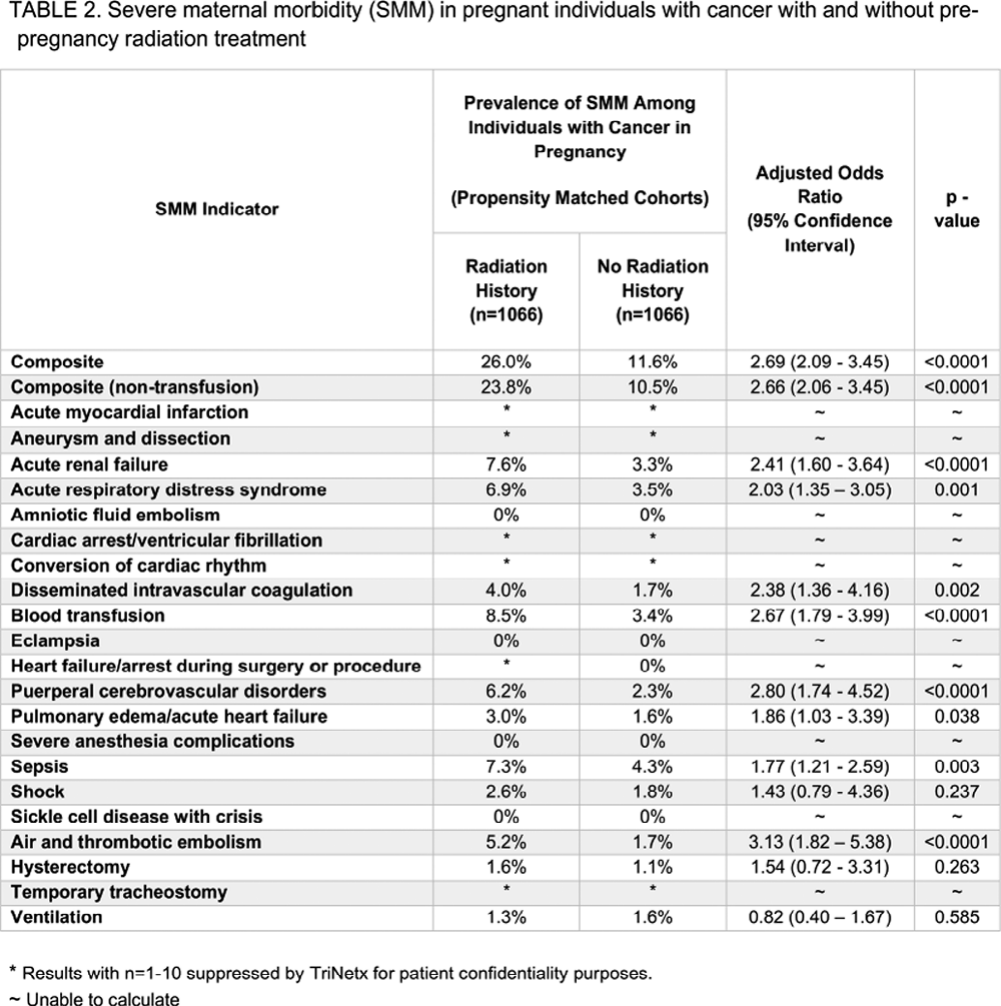

**10:30 AM – 12:00 PM**

## 738 | **Fetal** Scalp **pH Prediction: Human Expertise Still Beats Artificial Intelligence**

Juliette Vitrou^1^; Charles Garabedian^2^; Aude Girault^3^; Mathieu Hivert^4^

^1^Maternité Port‐Royal, groupe hospitalier Paris Centre, AP‐HP, Paris, France; Institut interdisciplinaire santé des femmes, iWISH, Université Paris cité, Paris, France;, Paris, Ile‐de‐France; ^2^CHU Lille, Lille, Nord‐Pas‐de‐Calais; ^3^Department of Obstetrics and Gynecology, Port‐Royal Maternity Hospital, AP‐HP, Cochin Hospital, FHU PREMA, Paris, France, Paris, Ile‐de‐France; ^4^CHU Lille, Department of Obstetrics, Lille, France; Univ Lille, ULR 2694‐METRICS, Lille, France., Lille, Nord‐Pas‐de‐Calais

**Objective**: To compare the accuracy of fetal scalp pH prediction by midwives, residents, and a large language model (ChatGPT) against actual measurements.

**Study Design**: Prospective monocentric study including term laboring women undergoing fetal scalp blood sampling for FHR II tracings. For each case, three pH predictions were independently obtained from a resident, a midwife, and ChatGPT, based on standardized clinical data and cardiotocographic tracings. Correlation with actual pH was assessed with Spearman's ρ, and accuracy using mean absolute error (MAE) and correct classification within predefined categories (<7.20; 7.20–7.24; >7.24). Based on prediction performance, we estimated the proportion of avoidable pH tests and potential clinical consequences, including avoidable cesarean deliveries and missed severe acidosis.

**Results**: A total of 95 fetal scalp pH measurements were analyzed. Correlation with actual pH values was weak for all predictors, with the highest for midwives (ρ = 0.26, *p*  = 0.011). MAE was lowest for midwives (0.042, 95% CI 0.036–0.050) and residents (0.047, 95% CI 0.038–0.056), compared with ChatGPT (0.098, 95% CI 0.087–0.110). Correct categorical prediction rates were 61.0% for midwives, 59.7% for residents, and 24.7% for ChatGPT. ChatGPT systematically underestimated fetal pH (71.4% of cases), whereas midwives and residents showed more balanced under‐ and overestimation. Compared to predictions, fetal scalp pH testing avoided between 1.3% (ChatGPT) and 5.2% (midwives and residents) of neonatal acidosis cases; and prevented unnecessary cesarean deliveries in 19.5% of cases when guided by midwife or resident predictions, but up to 62.3% when compared to ChatGPT‐based decisions.

**Conclusion**: Midwives and residents demonstrated comparable accuracy in predicting fetal scalp pH, both markedly outperforming ChatGPT. While professional clinical judgment can potentially reduce unnecessary fetal blood sampling and cesarean deliveries, reliance on large language models in their current state would increase misclassification risk and unnecessary interventions.

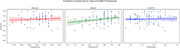

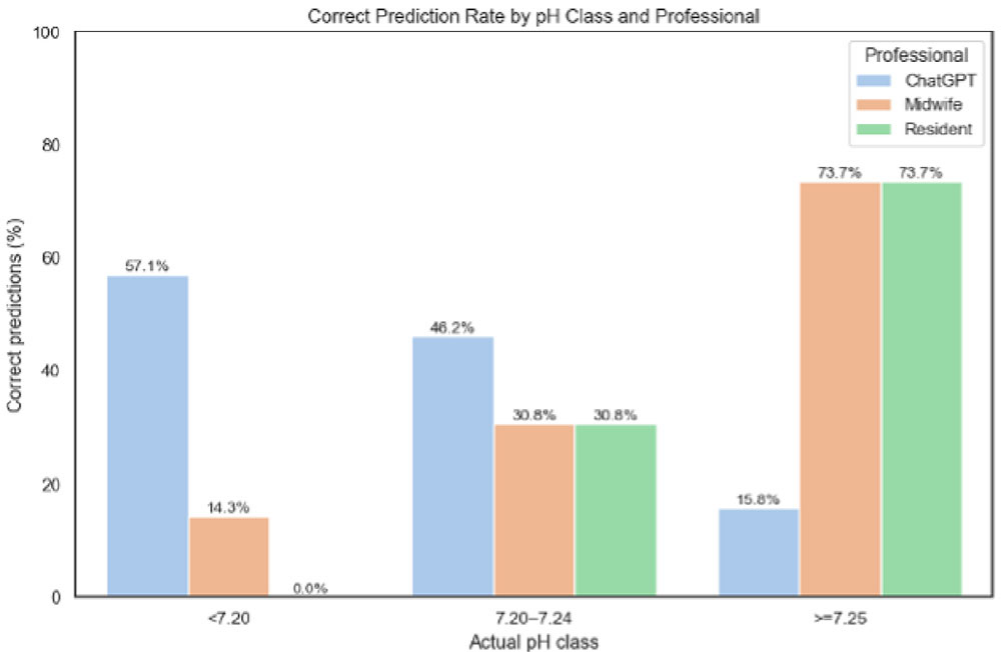

**10:30 AM – 12:00 PM**

## 739 | Standardized **Postpartum Blood Pressure Protocol and Lower Rates of Persistent Hypertension at 6 Weeks Postpartum**

Audra C. Fain^1^; Thalia Mok^2^; Jenny Y. Mei^3^

^1^Department of Obstetrics and Gynecology, David Geffen School of Medicine at University of California, Los Angeles (UCLA), Los Angeles, CA; ^2^David Geffen School of Medicine at University of California, Los Angeles (UCLA), Los Angeles, CA; ^3^Stanford University, Stanford University, CA

**Objective**: Hypertensive disorders of pregnancy (HDP) increase risk of chronic hypertension (CHTN) and cardiovascular disease. We evaluate the effect of a postpartum (PP) HTN standardized clinical assessment and management plan (SCAMP) on persistent HTN at 6 weeks PP.

**Study Design**: Secondary analysis of a prospective cohort study of patients with PP HTN at a quaternary care center for 6 months after enacting an institution‐wide SCAMP. Prospective group (P) was compared with historical controls (C) who delivered prior to initiation of the SCAMP. The SCAMP included 1) initiation or uptitration of medication for any blood pressure (BP) ≥150/100 or any 2 BPs ≥140/90 within a 24 hour period with goal of BP < 140/90 12 hours prior to discharge, 2) enrollment in a remote BP monitoring system upon discharge. Inclusion criteria were existing diagnosis of CHTN or HDP and a documented BP at a 6‐week PP visit. Primary outcome was persistent HTN, defined as SBP ≥ 140 or DBP ≥ 90 at 6 weeks PP. Secondary outcomes included mean BP values, BP ascertainment, and use of anti‐HTNs. For all analyses, *p*‐values were two‐way, and level of statistical significance was set at *p* < 0.05.

**Results**: Of 780 PP HTN patients in the prospective cohort study, 627 (80.4%) met inclusion criteria for the secondary analysis, 305 in (P) cohort and 322 historical controls (C). (P) cohort had lower rates of CHTN, more patients on anti‐HTNs, and higher rate of nifedipine use (Table 1). Logistic regression demonstrated that persistent HTN at 6 weeks PP was significantly lower in (P) cohort (adjusted odds ratio [aOR], 0.62, 95% confidence interval [CI] 0.38–0.99; *p* = 0.048) (Table 2). (P) cohort had lower mean systolic (*p* < 0.001) and diastolic BP (*p* < 0.001). (P) cohort had earlier BP assessment (*p* < 0.001) and higher rate of any BP ascertainment prior to 6‐weeks (aOR, 4.35; 95% CI, 2.86–6.62; *p* < 0.001).

**Conclusion**: Implementation of a SCAMP was associated with reduced persistent HTN at 6 weeks PP, with lower mean systolic and diastolic BP and improved BP ascertainment. More data is needed to evaluate how these findings mitigate long‐term HTN risk.

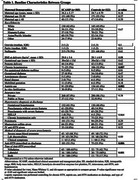

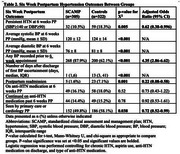

**10:30 AM – 12:00 PM**

## 740 | **A Risk‐**Based **Model for Cesarean Delivery Following Induction in Term Hypertensive Nulliparas**

Avihu Krieger^1^; Michal Axelrod^2^; Shiran Bookstein^3^; Baha M. Sibai^4^; Shalom Mazaki‐Tovi^5^; Michal Fishel Bartal^6^

^1^The Department of Obstetrics and Gynecology, The Chaim Sheba Medical Center, Ramat‐Gan, Israel, Ramat Gan, Tel Aviv; ^2^Sheba Medical Center, Sheba Medical Center, HaMerkaz; ^3^Sheba Medical Center, Ramat Gan, Israel, Ramat Gan, HaMerkaz; ^4^Division of Maternal‐Fetal Medicine, Department of Obstetrics, Gynecology and Reproductive Sciences, McGovern Medical School at UTHealth Houston, Houston, TX; ^5^The Department of Obstetrics and Gynecology, The Chaim Sheba Medical Center, Ramat‐Gan, Israel, Ramat Gan, HaMerkaz; ^6^Division of Maternal‐Fetal Medicine, Department of Obstetrics, Gynecology and Reproductive Sciences, Sheba Medical Center, Tel Aviv University, Israel, Ramat Gan, HaMerkaz

**Objective**: To identify predictors of successful induction of labor (IOL), defined as vaginal delivery, and evaluate unplanned cesarean delivery (CD) risk among nulliparous individuals with singleton pregnancies and hypertensive disorders of pregnancy (HDP) undergoing IOL at term (≥37 weeks).

**Study Design**: This retrospective cohort study included all nulliparous individuals with HDP who underwent IOL at term from 2010–2025. We excluded those with multiple gestations or planned CD. We evaluated risk factors for unplanned CD, and maternal and neonatal outcomes according to mode of delivery.

Risk stratification for unplanned CD was then performed using logistic regression by evaluating the cumulative impact of identified predictors including maternal age, Body mass index, severe HDP, thrombocytopenia and the need for cervical ripening.

**Results**: Of 6670 individuals with HDP during the study period, 2609 (38.6%) underwent IOL at term, and 1326 (50.8%) nulliparous individuals met inclusion criteria. Of these, 979 (73.8%) had a vaginal delivery, while 347 (26.2%) underwent unplanned CD.

Compared to those who delivered vaginally, individuals undergoing CD were older (32.1 vs 29.8 years, *p* < 0.001), had a higher body mass index (31.6 vs 29.4 kg/m^2^, *p* < 0.001), more frequent thrombocytopenia (3.5% vs 1.5%, *p*  = 0.03), higher incidence of severe HDP (16.7% vs 10.7%, *p* < 0.001) and were more likely to require cervical ripening (70.9% vs 59.5%, *p* < 0.001) (Table 1). Presence of ≥4 risk factors was associated with a 64.7% CD rate and yielded a positive likelihood ratio (LR) of 5.17 (95% CI 1.92–13.99). CD risk, stratified by cumulative number of identified risk factors, along with corresponding LR, is presented in Figure 2.

**Conclusion**: Approximately one in four nulliparous individuals with HDP undergoing IOL at term will end with an unplanned CD. Among those with four identified risk factors the risk rose to nearly two out of three. These findings offer a practical framework for individualized risk estimation and may support shared decision‐making regarding mode of delivery in this high‐risk population.

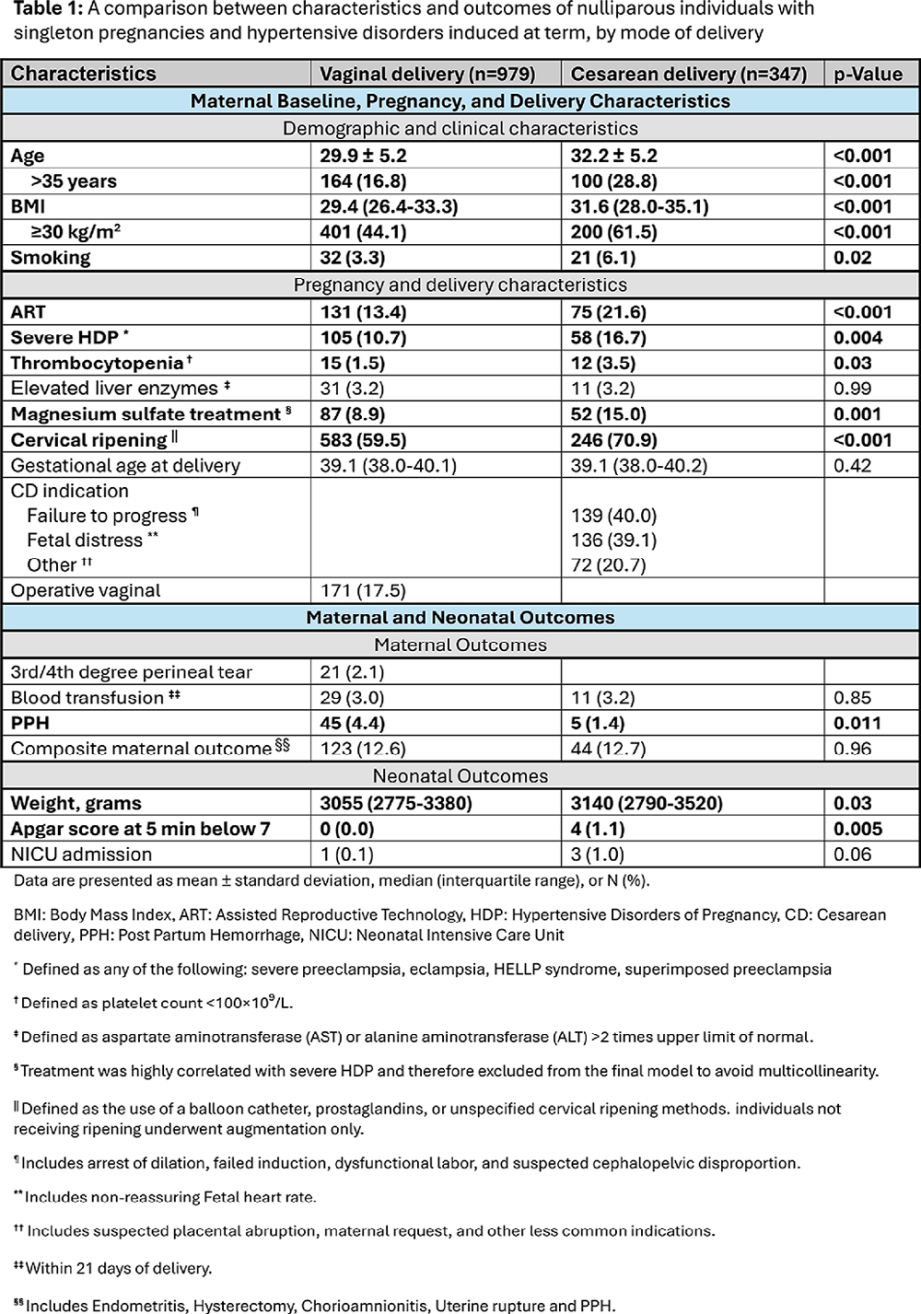

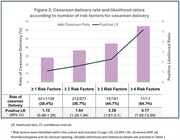

**10:30 AM – 12:00 PM**

## 741 | **Proactive AI Check‐Ins Rapidly Re‐Engage Patients With Gestational Diabetes: A Pilot Study**

Avish Arora^1^; Pe'er Dar^2^; Georgios Doulaveris^2^

^1^Montefiore/Albert Einstein College of Med., New York, NY; ^2^Montefiore Medical Center/Albert Einstein College of Medicine, Montefiore Medical Center/Albert Einstein College of Medicine, NY

**Objective**: To evaluate the effectiveness of automated assistant‐initiated check‐ins in eliciting timely patient responses and promoting active self‐management among newly diagnosed GDM patients.

**Study Design**: In this prospective pilot study conducted from June 1–July 30, 2025, we enrolled ten pregnant individuals recently diagnosed with diet‐controlled GDM. We developed a patient‐facing, agentic AI tool for GDM that received FDA approval as an investigational device to support self‐management. The WhatsApp‐based assistant logged patient‐reported glucose values, interpreted trends, offered tailored nutritional guidance, encouraged physical activity, and escalated concerning symptoms or readings. To enhance engagement, the assistant proactively delivered two types of automated check‐ins: daily morning summaries (sent at 09:00 am, summarizing the previous three days and requesting fasting glucose and breakfast details) and inactivity prompts (triggered after 22 hours of no interaction, excluding quiet hours). Primary outcomes were the proportion of check‐ins that received any patient response and those answered within 60 minutes.

**Results**: During the study, the virtual assistant issued 498 automated check‐ins, comprising 350 inactivity prompts (70%) and 148 morning summaries (30%). Patients replied to 318 (63.9%) of these automated messages. A total of 206 replies (39.7%) occurred within 60 minutes, with a median response time of 6.8 minutes (IQR 2.7–18.5). Five out of ten actively participating patients responded to over 90% of automated check‐ins. Two symptom‐related patient messages (one reporting dizziness, another nausea) were immediately escalated to clinicians within 15 minutes. No glucose‐related safety alerts were missed.

**Conclusion**: Automated conversational check‐ins from an AI‐powered GDM assistant effectively and rapidly re‐engage patients without added clinician burden. A randomized controlled trial comparing traditional nutritional counseling with agentic AI–based diabetes management is underway.

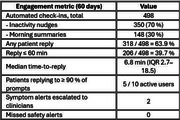

**10:30 AM – 12:00 PM**

## 742 | **Risk** Factors **for Unplanned Cesarean Delivery after Prelabor Rupture in Term Twin Pregnancies**

Avishag Abecassis^1^; Keren Zloto^2^; Hadeel Wattad^3^; Noa Gonen^4^; Shali Mazaki‐Tovi^5^; Michal Fishel Bartal^6^

^1^Department of Obstetrics and Gynecology, Sheba Medical Center, Ramat Gan, HaMerkaz; ^2^sheba medical center, Tel Hashomer, Tel Aviv; ^3^Sheba Medical center, Israel, Ramat Gan, HaMerkaz; ^4^Wolfson Medical Center, Holon, HaMerkaz; ^5^Department of Obstetrics and Gynecology, Sheba Medical Center, Ramat Gan, Tel Aviv; ^6^Division of Maternal‐Fetal Medicine, Department of Obstetrics, Gynecology and Reproductive Sciences, Sheba Medical Center, Tel Aviv University, Israel, Ramat Gan, HaMerkaz

**Objective**: Current literature offers limited data regarding the association between prelabor rupture of membranes (PROM) likelihood of unplanned cesarean delivery (CD) in twin pregnancies. Our study aims to determine risk factors associated with unplanned CD in individuals with twin pregnancies presenting with PROM.

**Study Design**: A retrospective cohort study conducted at a single tertiary medical center including all twin pregnancies with planned vaginal deliveries and PROM ≥36 weeks between 2011and 2025. Maternal and neonatal outcomes were compared between individuals who delivered vaginally and those who underwent unplanned CD.

**Results**: During study period 421 twins pregnancies presented with PROM >36 weeks of them 258 (61.2%) met inclusion criteria. The rate of unplanned CD was 28.3%. Patients in the unplanned CD group had a higher median maternal age (33.7 VS. 32.4 years, *p* = 0.04), higher BMI (29 vs. 27.1, *p* = 0.02), higher rate of nulliparity (60.3% vs. 38.9%, *p* < 0.01), cervical dilation < 2cm on admission (67.1% vs.36.8%, *p* < 0.01) and PROM duration >24 hours (32.9% vs. 14.6%, *p* < 0.01). Prior vaginal delivery was less common in the unplanned CD group (58.9% vs. 19.2%, *p* < 0.01). Intrapartum fever and oxytocin use were not associated with increased CD rates (Table 1). Postpartum hemorrhage occurred more frequently following vaginal delivery (9.7% vs. 1.4%, *p* < 0.01), whereas adverse neonatal outcomes including 1‐minute Apgar < 7 and NICU admission were more common after CD (6.8% vs. 3%, *p* = 0.04 and 8.2% vs. 2.4%, *p* < 0.01, respectively). (Table 2).

**Conclusion**: In term twin pregnancies with PROM, a trial of labor is associated with a high rate of vaginal delivery. Nulliparity, limited cervical dilation, and prolonged latency were associated with unplanned cesarean delivery. These factors may aid clinicians and patients alike in decision‐making during labor induction in this population.

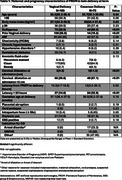

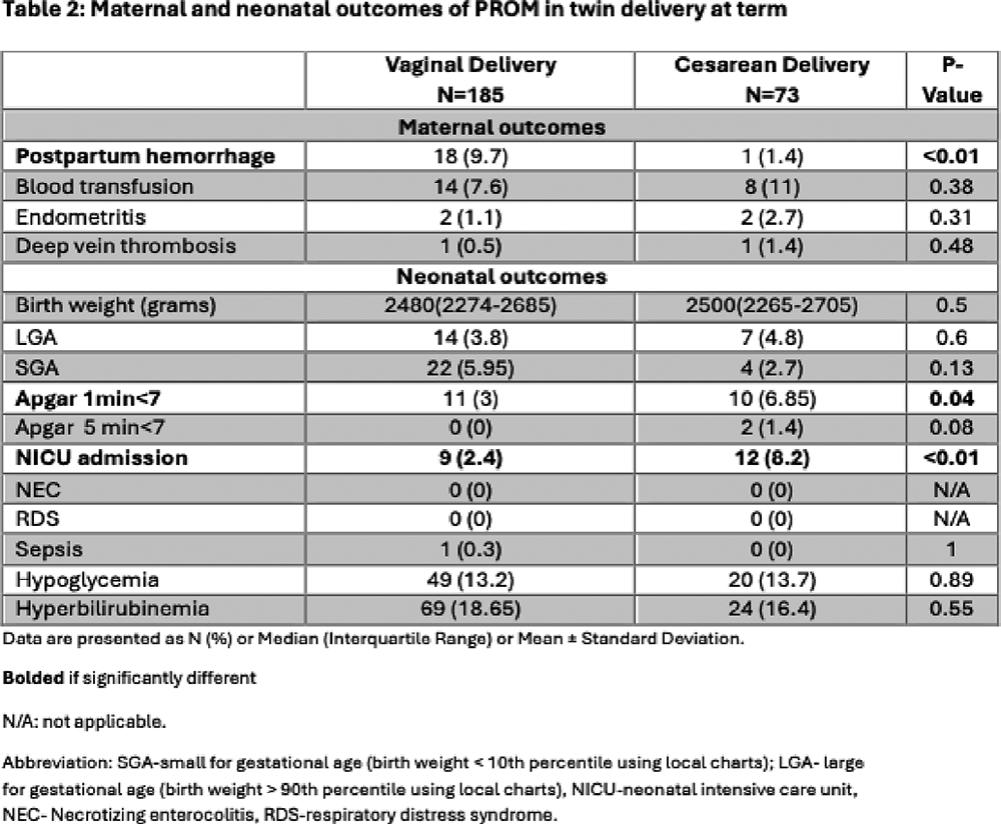

**10:30 AM – 12:00 PM**

## 743 | **Clinical** Implications **and Risk Factors of Increased Caput Succedaneum During Second Stage of Labor**

Bar Narkis; Yossi Geron; Gil Zeevi; Yinon Gilboa; Miriam Lopian; Or Lee Rak; Asaf Romano

Helen Schneider Hospital for Women, Rabin Medical Center, Petah Tikva, HaMerkaz

**Objective**: Caput succedaneum (CapS) is a common finding during labor, yet remains understudied as a diagnostic and prognostic marker of labor dystocia. We sought to determine risk factors for large CapS and assess its association with maternal and neonatal morbidity using a novel sonographic measurement technique.

**Study Design**: This single‐center prospective cohort study included 121 women in the second stage of labor who underwent transperineal ultrasound. CapS was measured in three dimensions (figure 1), with the Z‐axis (depth) chosen for analysis. Large CapS was defined as >2.5 cm. Maternal, fetal, and labor characteristics were collected. Head perineal distance (HPD), angle of progression (AoP), and fetal head position (OA, OP, OT) were also measured. Outcomes included mode of delivery, a composite neonatal morbidity index (Apgar < 7 at 5 minutes, umbilical artery pH < 7.1, NICU admission, or neonatal injury), and composite maternal morbidity.

**Results**: Large CapS (*n* = 28) was associated with prolonged second stage (>3 hours, OR 4.81, *p* < 0.01), shorter HPD (3.1 ± 1.0 vs. 3.9 ± 1.1 cm, OR 1.91, *p* < 0.01), and narrower AoP (138° vs. 145°, OR 0.94, *p* = 0.04). Fetal malposition was more frequent (non‐OA vs. OA: OR 3.25, 95% CI 1.28–8.25, *p* = 0.01). Rates of operative vaginal delivery (vacuum extraction: 64.3% vs. 35.5%, OR 4.91, *p* < 0.01) and cesarean section (14.3% vs. 6.5%, OR 6.00, *p* = 0.02) were both increased in the large CapS group. Composite neonatal morbidity was higher in the large CapS group (39.3% vs. 18.3%, OR 2.89, *p* = 0.02). No significant difference was found in maternal morbidity.

**Conclusion**: A Z‐axis CapS >2.5 cm is associated with prolonged labor, fetal malposition, increased neonatal complications, and higher rates of both vacuum‐assisted and cesarean deliveries. Ultrasound‐based CapS assessment may offer a valuable, underutilized tool for identifying labor dystocia and guiding timely obstetric decision‐making.

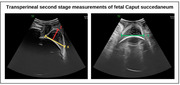

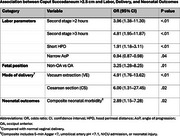

**10:30 AM – 12:00 PM**

## 744 | **Initial** Results **of Contingency Management to Treat Substance use Disorders in Postpartum Individuals**

Bridget M. Galati^1^; Anna Bay^1^; Brittaney Vaughn^2^; Michael Wenzinger^1^; Cynthia Rogers^1^; Jeannie C. Kelly^1^

^1^Washington University School of Medicine, Washington University School of Medicine, MO; ^2^Washington University School of Medicine, Barnes Jewish Hospital/Saint Louis, MO

**Objective**: The postpartum period is the highest risk time for individuals with substance use disorders (SUD), where treatment retention can be difficult. Contingency management (CM) is a behavioral treatment that provides positive rewards for targeted behaviors, such as treatment attendance or urine drug screen results. CM is one of the most powerful tools to improve outcomes, but is understudied in the obstetric population. We sought to evaluate the impact of a CM program targeting postpartum (PP) patients in a maternal SUD clinic.

**Study Design**: We conducted a pre‐post study in an urban maternal SUD clinic that provides treatment for SUD. A CM program was implemented and PP individuals received a grocery gift card of escalating amounts for attending a PP appointment each month, up to the one‐year PP mark (Table 1). If a participant missed an appointment and was unable to reschedule within 2 weeks, this was treated as a gap in treatment and the monetary amount was reset back to the baseline amount. We included all PP patients seen 6 months pre‐CM (August 1^st^ 2024 through January 31^st^ 2025) and compared to patients seen 6 months post‐CM implementation (February 1^st^ through July 31^st^ 2025). Our primary outcome was percent of attended visits. Our secondary outcome was average CM compensation per patient over 6 months.

**Results**: 43 patients were included (29 pre‐CM, 14 post‐CM). The majority (67%) had opioid use disorder and 49% had stimulant use disorder; 60% had multiple substance use disorders. 63% had a co‐occurring psychiatric condition, of which 44% were prescribed psychiatric medication. Pre‐CM, PP appointment attendance was 27% (30 of 110 appointments, *n* = 29 patients). Post‐CM, PP appointment attendance significantly increased to 70% (45 of 64 appointments, *n* = 14 patients; *p* = 0.0007). Average time PP at time of initial enrollment was 3 months but most often was 1 month (50%). Average total grocery gift card amount dispensed throughout the 6‐month period was $82.86 (± 55.89).

**Conclusion**: Contingency management may be a helpful strategy to improve treatment attendance and retention during the high‐risk PP period.

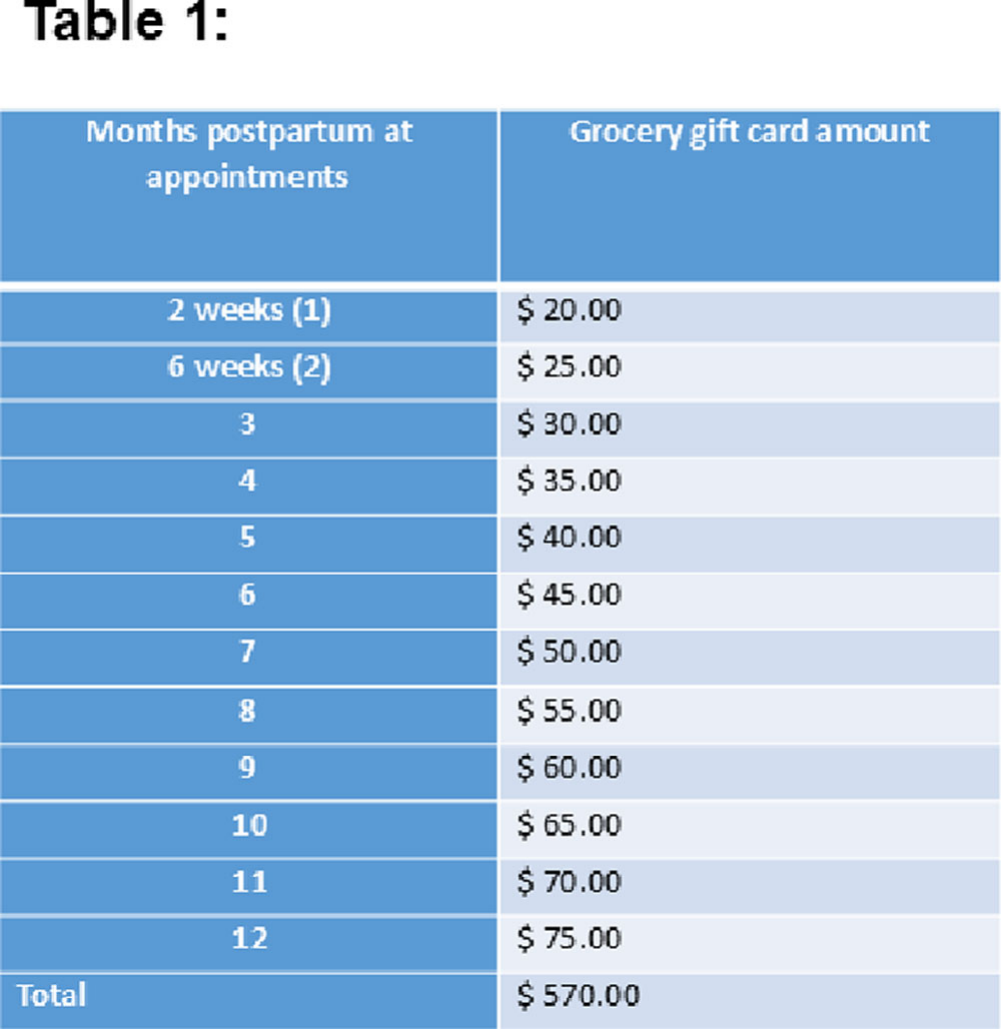

**10:30 AM – 12:00 PM**

## 745 | Experienced **Discrimination and Risk of Delivery Morbidity**

Bridget C. Huysman^1^; April Y. Lewis^2^; Mia Malcolm^2^; Harry A. Obeng^3^; Paige Wilder^4^; Maya Bussey^2^; Lori Atwood^1^; Adia Woodson^5^; Monique Gill^2^; Rachel Witt^6^; Eve Colson^2^; Ebony Carter^7^; Candice L. Woolfolk^1^

^1^Washington University School of Medicine, Washington University School of Medicine, MO; ^2^Washington University School of Medicine, St. Louis, MO; ^3^School of Public Health, Washington University in St. Louis, Washington University in St. Louis, MO; ^4^Washington University School of Medicine, Saint Louis, MO; ^5^Washington University in St. Louis, Saint Louis, MO; ^6^Children's Minnesota, Minneapolis, MN; ^7^University of North Carolina, University of North Carolina, NC

**Objective**: Racism and discrimination impact severe maternal morbidity (SMM) and obstetric (OB) complications. However, it is not known if a difference in discrimination correlates with morbidity risk. We sought to compare discrimination experienced by birthing persons who have an SMM by CDC criteria or unanticipated OB event (delivery morbidity) at delivery.

**Study Design**: A prospective survey of a convenience sample of birthing people presenting for delivery at a single center from 03/2024 to 06/2025 using the Discrimination in Medical Settings Scale (DMSS) administered prior to discharge. Birthing people admitted for an indication not related to delivery, delivered off L&D or precipitously, or unable to speak English were excluded. DMSS scores were compared to people who experienced delivery morbidity and those who did not. We analyzed by reports of any discrimination, and ≥90^th^ percentile (score 11). Survey responses were compared between groups using Chi‐squared or Mann Whitney U tests as appropriate and multivariable logistic regression was used to estimate adjusted odds ratios(aOR) and 95% confidence intervals (CI).

**Results**: Prior to discharge, 262 birthing people were approached to complete the survey, 83 declined, and 179 responded. Of those who participated, over 60% of the cohort identified as a person of color, of whom 15.1% experienced SMM, and 53.6% experienced an unanticipated OB complication. There was a statistically significant difference in reported total DMSS scores (8.24 ± 3.31 SMM vs. 7.78 ± 1.83, *p* = 0.02). DMSS scores ≥ 90^th^ percentile were significantly higher among those who experienced delivery morbidity compared to those who did not, with an almost 5 times higher risk of reporting discrimination (aOR 4.94 [95% CI 1.26, 19.37]) (Table). Less courtesy and less respect were reported by those experiencing delivery morbidity (courtesy *p* < 0.01; respect *p* = 0.04) (Table).

**Conclusion**: Birthing people who experience discrimination may be at higher risk of morbidity at the time of delivery. Strategies to mitigate discrimination experienced during pregnancy care could be promising to reduce morbidity.

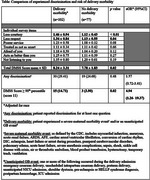

**10:30 AM – 12:00 PM**

## 746 | Development **and User Evaluation of LUNA: A Virtual Reality Program for Natural Delivery Training**

Byung Soo Kang^1^; Hyun Sun Ko^1^; Subeen Hong^2^; Elisa Gi Soo Um^2^; Seon Ui Lee^3^; Oyoung Kim^4^; Yeon Hee Kim^5^; Jiyoung Kwon^6^; Su Hwan Lee^7^; In Yang Park^2^

^1^Department of Obstetrics and Gynecology, Seoul St. Mary's Hospital, College of Medicine, The Catholic University of Korea, Seoul, Seoul‐t'ukpyolsi; ^2^Department of Obstetrics and Gynecology, Seoul St. Mary's Hospital, College of Medicine, The Catholic University of Korea, Seoul, Korea, Seoul, Seoul‐t'ukpyolsi; ^3^Department of Obstetrics and Gynecology, Incheon St. Mary's Hospital, College of Medicine, The Catholic University of Korea, Seoul, Korea, Incheon, Kyonggi‐do; ^4^Department of Obstetrics and Gynecology, Yeouido St. Mary's Hospital, College of Medicine, The Catholic University of Korea, Seoul, Korea, Seoul, Seoul‐t'ukpyolsi; ^5^Department of Obstetrics and Gynecology, Uijeongbu St. Mary's Hospital, College of Medicine, The Catholic University of Korea, Seoul, Korea, Uijeongbu, Kyonggi‐do; ^6^Department of Obstetrics and Gynecology, Eunpyeong St. Mary's Hospital, College of Medicine, The Catholic University of Korea, Seoul, Korea., Seoul, Seoul‐t'ukpyolsi; ^7^INPINI AI Co., Ltd, Seoul, Korea., Seoul, Seoul‐t'ukpyolsi

**Objective**: This study aimed to evaluate a newly developed virtual reality (VR) educational program for natural delivery, with a primary focus on its clinical realism and its ability to replicate key obstetric procedures. We also assessed user feedback on satisfaction, usability, and educational effectiveness to guide future improvements.

**Study Design**: We developed LUNA (Labor Unit for Newborn Arrival), an immersive VR training platform replicating core natural delivery processes, including spontaneous vaginal delivery, episiotomy, and perineal repair. The platform was specifically designed to replicate real clinical workflows and visual environments. Licensed physicians participated in a single VR session using LUNA, followed by a structured survey assessing six domains: demographics, immersion, learning satisfaction, system usability, learning effectiveness, and open‐ended feedback. All items were rated on a 5‐point Likert scale. A total of 14 physicians have completed the survey to date.

**Results**: Participants strongly endorsed the realism of the VR environment, with an average immersion score of 4.5 and specific praise for its likeness to actual clinical settings. Learning satisfaction scored an average of 4.4, especially for “structured content design” and “ease of goal achievement.” System usability received an average score of 4.3, with few reports of technical difficulties. In terms of learning effectiveness, participants rated 4.6 for “VR helped improve procedural skills in real practice” and 4.7 for “more effective than conventional methods.” In open‐ended responses, participants highlighted “the ability to repeat practice realistically and learn without pressure” as the most satisfactory aspect.

**Conclusion**: As clinical training environments face increasing limitations, this modality shows offers a valuable supplement to traditional education. By replicating real‐life obstetric procedures in an immersive, repeatable format, LUNA supports procedural skill development and may enhance the quality and accessibility of obstetric training.

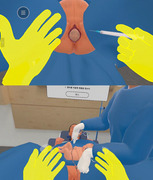

**10:30 AM – 12:00 PM**

## 747 | **Next** Steps **for Group Prenatal Care Research: Shifting From Intervention Design to Implementation Science**

Candice L. Woolfolk^1^; EleVATE Collaborative^2^; Julia Muller^3^; Traci Johnson^4^; Cheron Phillips^5^; Richelle Smith^5^; Annie Dude^3^; Julie Johnson^6^; Nia Plump^2^; Kelly McKay‐Gist^5^; Jeannie C. Kelly^1^; Nandini Raghuraman^7^; Melissa Tepe^8^; Shannon Lenze^9^; Ebony Carter^10^

^1^Washington University School of Medicine, Washington University School of Medicine, MO; ^2^EleVATE Collaborative, St. Louis Integrated Health Network, St. Louis, MO; ^3^University of North Carolina, Chapel Hill, NC; ^4^University of Missouri‐Kansas City, Kansas City, MO; ^5^St. Louis Integrated Health Network, St. Louis, MO; ^6^University of North Carolina – Chapel Hill, Chapel Hill, NC; ^7^Washington University School of Medicine, St. Louis, MO; ^8^Affinia Healthcare, St. Louis, MO; ^9^Washington University in St. Louis, School of Medicine, Department of Psychiatry, St. Louis, MO; ^10^University of North Carolina, University of North Carolina, NC

**Objective**: Despite evidence that group prenatal care (GPC) attendance ≥5 visits improves perinatal outcomes, recent negative trial results may reflect implementation challenges rather than intervention failure. This study analyzed the GPC engagement cascade to identify critical transition points where targeted implementation strategies may optimize intervention delivery.

**Study Design**: We conducted a cross‐sectional analysis of a multi‐site randomized controlled trial from 2021–2025. The study was conducted at 6 sites: 2 academic institutions and 4 community health centers. Associations between GPC receipt (1^st^ visit attendance) and retention (≥ 5 visit attendance) rates were assessed using Pearson's correlation coefficients. Sequential dropout rates were calculated as the proportion lost at each stage relative to the prior stage.

**Results**: Of 526 eligible patients, 307 (58.4%) were recruited into the study and 205 were randomized to GPC. Sequential dropout analysis showed: 41.6% (219/526) lost at recruitment, 26.8% (55/205) lost after randomization, and 34.0% (51/150) lost after initial attendance. First‐visit attendance was revealed as the critical bifurcation point: Probability[P](optimal engagement|receipt) = 66.0% overall versus P(optimal engagement|no receipt) = 0%. Site‐level correlation between receipt and retention was strong (*r* = 0.88, *p* < 0.05), with receipt explaining 77% of variance in retention.

**Conclusion**: Less than half of patients randomized to GPC achieve the 5‐session threshold associated with improved pregnancy outcomes. The strong correlation between receipt and retention across sites, combined with conversion rates among first‐time attendees, suggests that implementation quality—rather than intervention design—may determine trial outcomes. Future research should prioritize testing strategies to optimize initial patient engagement, as dismissing GPC based on implementation failures would abandon a potentially effective intervention before distinguishing implementation challenges from intervention ineffectiveness.

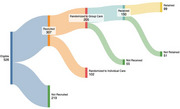

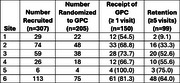

**10:30 AM – 12:00 PM**

## 748 | **Effect of Health Care Access on Post‐Cesarean Surgical Site Infection Severity (SSI)**

Hannah Kelly^1^; Carmen Rauh Garrido^2^; Emma Peek^3^; Kathleen Chang^4^; Joyce Liu^4^; Janice Wong^5^; Sarah K. Dotters‐Katz^6^; Rachel L. Wood^7^

^1^Duke University Hospital, Durham, NC; ^2^Stanford University Department of Obstetrics & Gynecology, Stanford University, CA; ^3^Duke University School of Medicine, Duke University Medical Center, NC; ^4^Duke University School of Medicine, Durham, NC; ^5^University of California San Diego – Kaiser Permanente, San Diego, CA; ^6^Division of Maternal Fetal Medicine, Department of Obstetrics and Gynecology, Duke University, Duke University/Durham, NC; ^7^Division of Maternal Fetal Medicine, Department of Obstetrics and Gynecology, Duke University, Durham, NC

**Objective**: Patients who have increased access to health care have been shown to have better health outcomes. Patients from marginalized communities in particular face healthcare access barriers compounding pre‐existing disparities. Our study sought to assess how healthcare access affects post‐cesarean surgical site infection (SSI).

**Study Design**: This was an IRB‐approved, retrospective cohort study of patients with post‐cesarean wound infections requiring an emergency department or OB‐ED wound‐related visit from a single health‐care system between 6/1/2013–7/31/2022, excluding patients with placenta accreta or severe preeclampsia. Primary exposure was appropriate access to postpartum care “touchpoints”, defined as having attended a postpartum appointment, called nurse line, or EHR messaged which led to an appointment prior to diagnosis of SSI. The outcomes of interest were the location of SSI diagnosis, severity of wound infection based upon the need for hospital admission or surgical intervention. Bivariate statistics were used to analyze data. Regression models were developed to control confounders.

**Results**: 528 patients were included in this analysis, 390 (73.9%) had accessed healthcare before their SSI diagnosis (“prior touchpoint” group) and 141 (26.7%) whose first postpartum healthcare access was at the time of SSI diagnosis (“no prior touchpoint” group). Patients with no prior touchpoint were younger and more likely to be Black with public insurance (Table 1). Patients with no prior touchpoint had an increased odds of being diagnosed in an emergency setting (4.3, 95% CI 2.8–6.5), requiring readmission for SSI (OR 1.9, 95% CI 1.3–3.0) and requiring return to the OR for surgical intervention (OR 1.9, 95% CI 1.1–3.2). Both emergency setting and readmission remained significant only after adjusting for confounders (maternal age, BMI, and insurance status) (Table 2).

**Conclusion**: Patients who accessed less medical care before SSI diagnosis were more likely to require readmission again highlighting the disparity faced by individuals lacking healthcare access.

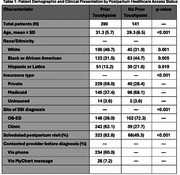

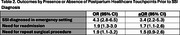

**10:30 AM – 12:00 PM**

## 749 | **Race and Ethnicity‐Specific Differences in Severe Preeclampsia Incidence: Assessment Per Socio‐Economic Status**

Carolyn N. Rocha^1^; Yadira L. Bribiesca Leon^1^; Zaira Chavez Jimenez^1^; Fay F. Pon^1^; Sawa Keymeulen^1^; Ariane C. Youssefzadeh^2^; Nicole M. Chadwick^1^; Joseph G. Ouzounian^2^; Koji Matsuo^1^

^1^Department of Obstetrics and Gynecology, Los Angeles General Medical Center, Los Angeles, CA; ^2^Division of Maternal‐Fetal Medicine, Department of Obstetrics and Gynecology, University of Southern California, Los Angeles, CA

**Objective**: To assess the incidence of preeclampsia with severe features (SPE) across race and ethnicity groups according to socio‐economic status.

**Study Design**: This cross‐sectional study queried the Agency for Health Healthcare Cost and Utilization Project's National Inpatient Sample. Study population included 19,907,373 hospital deliveries from 2016–2022. Exposure was race and ethnicity, grouped as White, Black, Hispanic, Asian, and Native American. The outcome measure was diagnosis of SPE. A multivariable generalized linear model was used to assess the exposure‐outcome association, adjusted for maternal age, year of delivery, clinical risk factors for preeclampsia (pregestational hypertension, diabetes mellitus, and obesity), and hospital parameters (location and teaching status, and region). The exposure‐outcome association was assessed according to socio‐economic status based on the combination of census‐level median household income (every quartile) and primary payer (private, Medicaid, and self‐pay).

**Results**: Across the census‐level median household income and primary payer strata, both Black and Native American people were more likely to have a diagnosis of SPE (Table). The effect sizes of developing SPE for Black people compared to White people were similar across the socio‐economic strata for private insurance (adjusted rate ratio, 1.76, 1.66, 1.58, and 1.61 for >75%ile, 51–75%ile, 26–50%ile, and ≤25%ile census‐level median household income groups, respectively) as well as for Medicaid insurance (adjusted rate ratio, 1.68, 1.55, 1.60, and 1.60 for >75%ile, 51–75%ile, 26–50%ile, and ≤25%ile census‐level median household income groups, respectively). Results were similar for Native American people (Table).

**Conclusion**: Race and ethnicity‐specific differences in rates of SPE incidence were similar across socio‐economic status. This suggests the development of SPE may be driven by a combination of social drivers of health, structural racism within the medical system, and biologic factors. Further research is needed to identify the drivers of SPE to reduce incidence and improve outcomes.

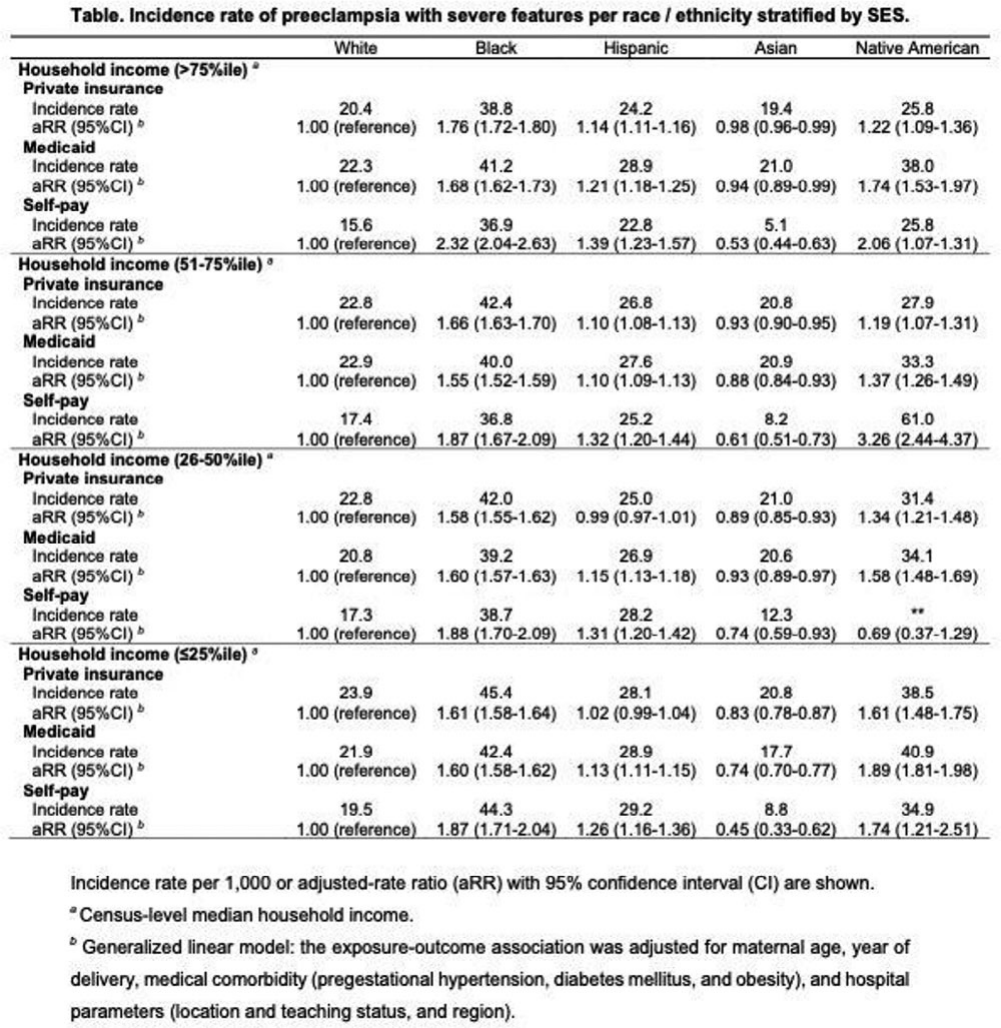

**10:30 AM – 12:00 PM**

## 750 | **Perinatal Patient Preferences Related to Medications for Opioid Use Disorder: A Discrete Choice Experiment**

Casey Tak^1^; Samantha West^1^; Valerie Jansen^2^; Karissa S. MacDonald^3^; Kailee Bunte^4^; Jasmin E. Charles^1^; Erin Johnson^1^; Torri D. Metz^1^; Marcela C. Smid^5^

^1^University of Utah, Salt Lake City, UT; ^2^USARA, Salt Lake City, UT; ^3^Pheonix Rising, West Valley City, UT; ^4^University of Missouri Kansas City‐ School of Medicine, Kansas City, MO; ^5^University of Utah Health, Salt Lake City, UT

**Objective**: Medications for opioid use disorder (MOUD) reduce mortality and morbidity risk for perinatal individuals with opioid use disorder (OUD), yet little is known about their preferences for treatment options. Our objective was to evaluate factors that perinatal individuals with OUD prioritize when considering MOUD options.

**Study Design**: We recruited perinatal individuals ages ≥18 with OUD receiving MOUD (buprenorphine, methadone, naltrexone) to participate in a discrete choice experiment (DCE). A power analysis suggested 40 individuals would be needed to detect statistical differences. MOUD attributes were selected through literature review, expert input, and iterative feedback from a panel of people with lived experience. The final DCE included six attributes: treatment effectiveness, risk of neonatal opioid withdrawal syndrome (NOWS), administration (oral vs injection), ease of access (travel time, virtual appointments), frequency of required visits, and pain management support. We collected demographics and participants completed 13 choice sets of two hypothetical treatments with varying characteristics via a web‐based application. We applied a mixed logit model to estimate utility scores, with positive and negative values indicating preferences toward and against, respectively, the attributes.

**Results**: From March to July 2025, we recruited 26 participants from our multi‐disciplinary specialty perinatal addiction clinic. Most identified as White (84%), had public insurance (72%), and were pregnant (56%) (Table 1). Utility estimates indicated that participants had strong preferences for MOUD that offered lower risk of NOWS (β = +0.40; *p* < 0.01) and were easily accessible (β = +0.32, *p* = 0.02) (Figure 1). Conversely, participants expressed a preference against required daily visits for MOUD (β = –0.22; *p* = 0.02).

**Conclusion**: Perinatal individuals with OUD prioritized reduced NOWS risk and accessible treatment, whereas daily clinical visits, such as those required for methadone, were viewed negatively. Incorporating preferences into clinical decision‐making may improve treatment engagement and outcomes.

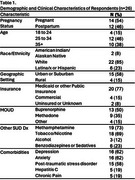

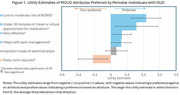

**10:30 AM – 12:00 PM**

## 751 | Pulmonary **Edema During Pregnancy: Experience From Upward of 70,000 Births**

Cassidy A. O'Sullivan^1^; Adam Wright^1^; Jessica C. Fields^2^; Matthew K. Hoffman^3^; Suneet P. Chauhan^4^; Anthony C. Sciscione^3^

^1^Christiana Care Health Services, Newark, DE; ^2^Delaware Center for Maternal Fetal Medicine, Newark, DE; ^3^Christiana Care Health System, Newark, DE; ^4^Delaware Center for Maternal‐Fetal Medicine of Christiana Care, Newark, DE

**Objective**: Pulmonary edema is a rare yet well‐known serious complication in pregnancy. The aim of this study was to describe the clinical features, and associated conditions among individuals with pulmonary edema during pregnancy.

**Study Design**: At a single tertiary care center, we identified deliveries with pulmonary edema between March 2014 and 2024 using ICD codes. Retrospective chart review was performed. Individuals were stratified by timing of diagnosis: before delivery (antepartum or intrapartum) versus after delivery (postpartum). Statistical comparisons were performed using Chi‐square and Fisher's exact tests as appropriate.

**Results**: During the study period, there were 70,465 deliveries, and ICD codes indicated 242 (0.3%) had pulmonary edema. After chart review, pulmonary edema was confirmed in 125 (0.2%), of which 51 (40.8%) were before delivery and 74 (59.2%) after. Majority were nulliparous (52.8%) and had BMI ≥ 30 kg/m^2^ (75.2%). Preeclampsia (76.0%), tocolytic use (61.6%), and infection (24%) were the three most common associated factors. Individuals diagnosed after delivery were significantly more likely to be at term (≥37 weeks: 67.6% vs 19.6%, *p* < 0.001), whereas those diagnosed before delivery were more likely to be preterm, particularly in the 28.0–36.6 weeks range (*p* < 0.001). Diabetes was more common in the antepartum group (25.5% vs 8.1%, *p* = 0.02), and preeclampsia was more prevalent before delivery (86.3% vs 69%, *p* = 0.04). The median day of postpartum diagnosis was 3.5 ± 3.6 days. ICU admission occurred in 12.8% of cases and did not differ significantly between the groups (*p* = 1.0). While there was one perimortem cesarean, there was no maternal death in the cohort.

**Conclusion**: Pulmonary edema remains an uncommon but clinically significant peripartum complication. Antepartum presentations were more often associated with pre‐eclampsia and prematurity, while postpartum cases tended to occur at term. Recognition of these patterns can inform antenatal counseling, and guide differential diagnosis in acute respiratory compromise with focus on risk reduction regarding ICU admission.

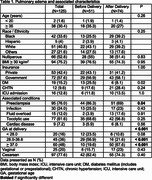

**10:30 AM – 12:00 PM**

## 752 | **Racial** Disparities **in Postpartum Hemorrhage Intervention Timing**

Catherine D. Yang; Sydney Seignious; Janeva Nicole Dimen; Sahel Hazrati; Mohammad Sunoqrot; Homa K. Ahmadzia

Inova Fairfax Hospital, Falls Church, VA

**Objective**: Black patients are at higher risk of morbidity and mortality from postpartum hemorrhage (PPH) but may also be less likely to receive escalations in care compared to White patients. This study examined whether delays in intervention beyond first‐line uterotonic medications in patients with PPH varied by patient race and insurance status. We hypothesized that Black patients and Medicaid patients experienced greater delays in receiving PPH interventions.

**Study Design**: A retrospective cohort study of patients with PPH during delivery in a large health system between 2019–2024. PPH was defined as total blood loss of 1000 mL or more. PPH interventions included balloon tamponade, uterine artery embolization (UAE), and hysterectomy. Time to intervention was defined as the time elapsed from the time of the PPH, estimated by the time of the 2nd medical intervention (aside from standard oxytocin). Patient demographics, obstetric characteristics, intervention times, and clinical outcomes were extracted from the electronic medical record. Time to each intervention was compared between Black and White patients and Medicaid and privately insured patients. Unadjusted analyses were performed using type 2, 2‐tailed t‐tests. A *p*‐value of 0.05 was considered significant.

**Results**: A total of 4317 patients were included, of which 13% were Black (*n* = 587) and 6.5% (*n* = 289) were publicly insured. Black patients received balloon tamponade later, on average, than White patients with a mean time of 229 minutes compared to 125 minutes (*p* = 0.03). Time to UAE or hysterectomy was not statistically different for Black compared to White patients (*p* = 0.34 and *p* = 0.41, respectively). There were no significant differences in time to balloon tamponade, UAE or hysterectomy between Medicaid and privately insured patients.

**Conclusion**: This study reveals potential disparities in postpartum hemorrhage care with respect to balloon tamponade, highlighting the need for monitoring of postpartum hemorrhage response time and its impact on patient outcomes.

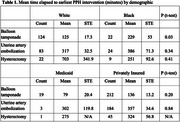

**10:30 AM – 12:00 PM**

## 753 | **Impact of Intramuscular Oxytocin on Postpartum Hemorrhage During a National Intravenous Fluid Shortage**

Catriona Lewis^1^; Amin Tavakoli^2^; Gabriela Dellapiana^3^

^1^Cedars‐Sinai Health Sciences University, Los Angeles, CA; ^2^Department of Obstetrics and Gynecology, Cedars‐Sinai Medical Center, Los Angeles, CA, CA; ^3^Cedars‐Sinai Medical Center, Los Angeles, CA

**Objective**: Oxytocin infusion requires dilution in intravenous (IV) fluids to reduce risk for toxicity. In response to the national IV fluid shortage in 2024, our hospital transitioned from a bolus of IV oxytocin (30 units in 500 mL lactated Ringer's) to intramuscular (IM) oxytocin (10 units) for active management of the third stage. We aimed to evaluate the impact of the IM oxytocin protocol on postpartum hemorrhage (PPH) rates.

**Study Design**: This is a retrospective cohort study of all vaginal deliveries ≥34 weeks at a quaternary hospital. We compared patients from the IM oxytocin period (10/2024–2/2025) to the same months during the two preceding years (IV oxytocin group; 10/2022–2/2023 and 10/2023–2/2024). The primary outcome was rate of PPH (blood loss ≥1000 mL). Secondary outcomes included total blood loss, additional uterotonic administration, and severe maternal morbidity. T‐test, chi‐square, and Wilcoxon rank‐sum were used as appropriate. Logistic regression was performed for potential confounders.

**Results**: Of 3963 patients, 1173 received IM oxytocin (30%) and 2790 received IV oxytocin (70%) postpartum. Patients who received IM oxytocin were more likely to have public insurance and receive prostaglandin labor augmentation; they were less likely to receive oxytocin augmentation or have admission thrombocytopenia (Table 1). Median total labor IV fluids were lower in the IM oxytocin group (661 mL vs 1816 mL, *p* < 0.01). PPH rates were lower in the IM oxytocin group (1% vs 2%, *p* = 0.04), but not after adjusting for admission thrombocytopenia (aOR 0.5, 95% CI 0.3–1.0). Use of additional uterotonics and tranexamic acid were more common in the IM oxytocin group; however, transfusion, hysterectomy, ICU admission, and postpartum length of stay were similar (Table 2).

**Conclusion**: Although adjusted analyses showed no significant difference in PPH, the IM oxytocin group required additional uterotonic administration. While IM oxytocin is a feasible alternative during an IV fluid shortage, its use may warrant closer clinical surveillance.

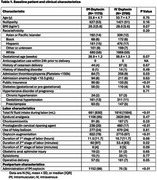

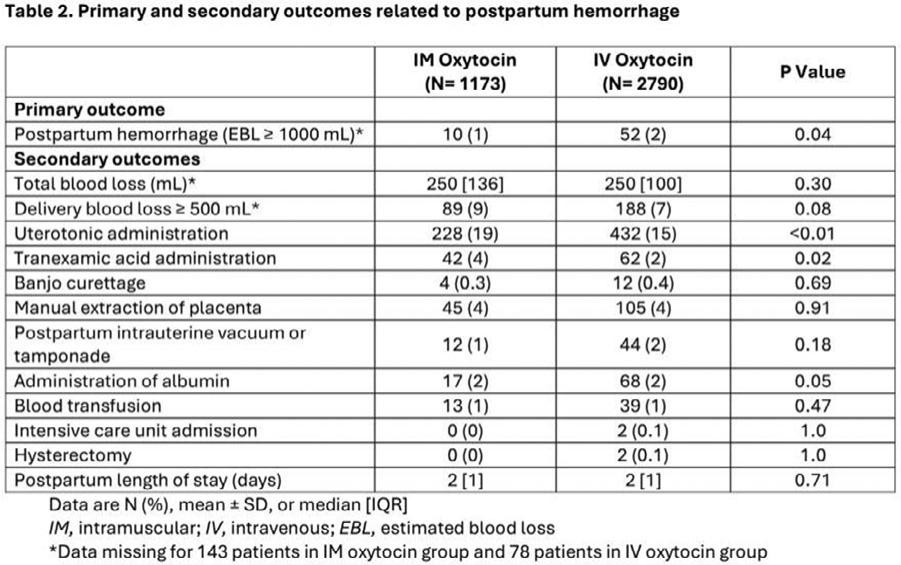

**10:30 AM – 12:00 PM**

## 754 | **Factors** Associated **With Performing a Cesarean Hysterectomy During Conservative Management Attempts for Placenta Accreta Spectrum**

Lena Segui^1^; Lola Loussert‐Chambre^2^; Maelys Nkobetchou^3^; Lise Couderc^2^; Aude Girault^4^; Paul Guerby^2^; Yoann Athiel^5^; Louise Ghesquiere^1^; Charles Garabedian^1^

^1^CHU Lille, Lille, Nord‐Pas‐de‐Calais; ^2^CHU Toulouse, Toulouse, Midi‐Pyrenees; ^3^Port‐Royal Maternity Hospital, AP‐HP, Cochin Hospital, Paris, France, Paris, Ile‐de‐France; ^4^Department of Obstetrics and Gynecology, Port‐Royal Maternity Hospital, AP‐HP, Cochin Hospital, FHU PREMA, Paris, France, Paris, Ile‐de‐France; ^5^APHP. Port Royal, Paris, Ile‐de‐France

**Objective**: The prevalence of placenta accreta spectrum (PAS) disorders has been increasing over recent decades. Some teams propose conservative management as an alternative to cesarean hysterectomy. Our aim was to examine factors associated with need of a per cesarean hysterectomy while conservative treatment was attempted in cases of PAS.

**Study Design**: This was a retrospective, multicenter study (3 centers in France), conducted between January 2013 and December 2023. Included were women who underwent an attempted conservative treatment for PAS. Women with a planned cesarean hysterectomy were excluded. Failure of expected conservative management was defined as the need for a hysterectomy during the same operative session as the cesarean delivery. Attempted conservative treatment were local resection or placenta left in situ partially or completely.

**Results**: Among the 101 women included in our study, 72 (71.3%) successfully underwent conservative treatment, while 29 (28.7%) experienced failure, requiring a cesarean hysterectomy. Maternal characteristics were similar between the two groups, except for a higher rate of prior uterine curettage in the failure group (51.7% vs. 25.0%, *p* = 0.010). Vaginal bleeding during pregnancy (69.0% vs. 38.9%, *p* = 0.006) and the need for antenatal corticosteroid therapy (69% vs. 43.7%, *p* = 0.02) were more frequent in the failure group. Intraoperative data showed a trend toward more frequent emergency cesareans in the failure group (41.4% vs. 23.6%, *p* = 0.074), and more intra operative identification of accreta compared to percreta (75.9% vs. 55.6%, *p* = 0.088).

**Conclusion**: The main risk factor for failure of attempted conservative treatment in cases of PAS is the presence of an history of currrettage and vaginal bleeding during pregnancy. Emergency delivery and intraoperative findings suggestive of accreta may also be associated with failure of conservative treatment.

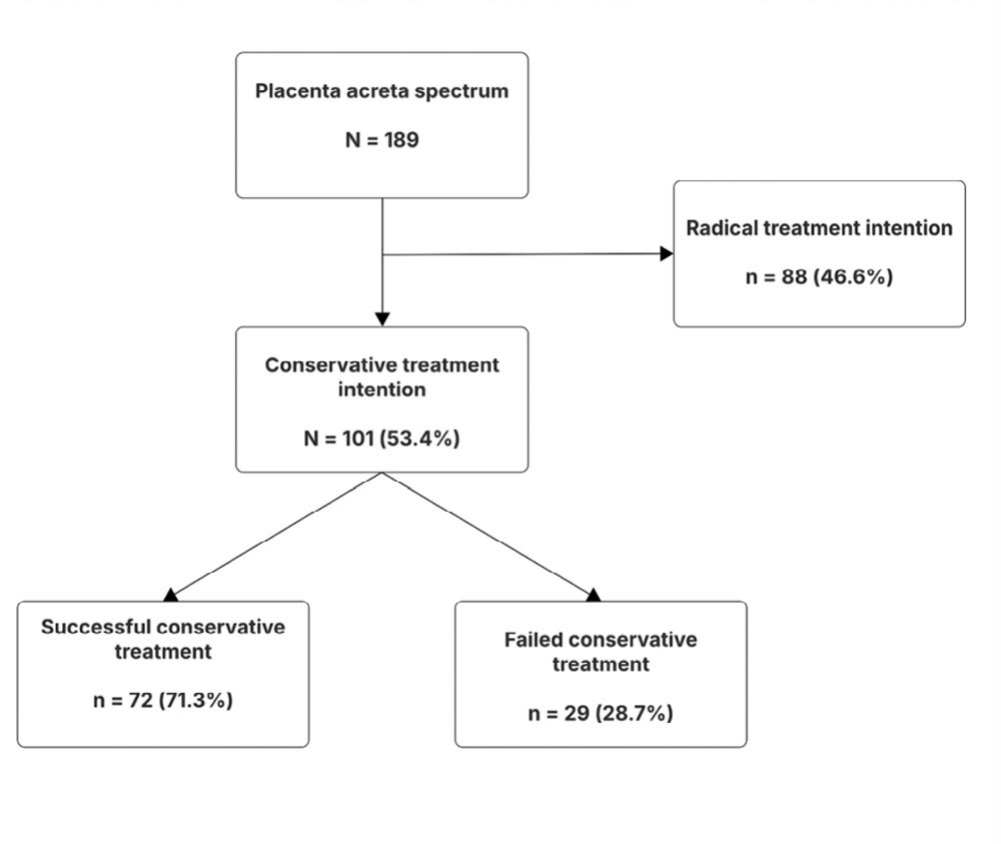

**10:30 AM – 12:00 PM**

## 755 | **Beyond** ICD **Codes and Quantitative Blood Loss: Evidence of Continued Increase in Postpartum Hemorrhage (2016–2025)**

Chloe Stanwyck^1^; Nan Guo^2^; Kay I. Daniels^2^; Kelly Fedoruk^2^; Brendan Carvalho^3^; Jessica Ansari^4^

^1^Stanford University, Menlo Park, CA; ^2^Stanford University School of Medicine, Stanford, CA; ^3^Stanford University Healthcare, Palo Alto, CA; ^4^Stanford University, Stanford University, CA

**Objective**: Epidemiologic studies of postpartum hemorrhage (PPH) rates are based on retrievable data elements (ICD codes, blood loss quantification, and transfusion records) that may be impacted by inaccurate documentation and coding errors. We aimed to assess an objective, laboratory‐based indicator of PPH – a ≥3 g/dL decline in hemoglobin – and compared trends in hemoglobin‐based PPH with PPH defined by blood loss, ICD codes, red blood cell (RBC) transfusion, or discharge summary mentions.

**Study Design**: We examined all deliveries at a tertiary care hospital from 1/1/2016 – 4/30/2025, excluding antenatally diagnosed placenta accreta. The primary outcome was ≥3 g/dL drop in hemoglobin. Secondary outcomes were quantitative or estimated blood loss (QBL or EBL) >1000 mL, PPH‐related ICD codes, RBC transfusion, and mention of clinically significant PPH in the discharge summary as identified by a large language model (GPT‐4.1). Yearly prevalence was plotted and annual change estimated with modified‐Poisson regression (log link, robust standard errors), treating calendar year as a continuous predictor.

**Results**: Among 42,373 deliveries, PPH incidence rose steadily by every definition over the study period (Figure 1). Fall in hemoglobin increased by 4% per year, corresponding to a 45% higher risk in 2025 vs. 2016 (RR 1.45, 95 % CI 1.33–1.57; Table 1). Secondary outcomes showed even larger increases (RR [95 % CI]): QBL > 1000 mL, 1.94 (1.77–2.13); transfusion, 2.70 (2.18–3.35); ICD‐coded PPH, 2.81 (2.58–3.06); and LLM‐identified PPH in discharge summaries, 2.24 (2.02–2.48).

**Conclusion**: This contemporary investigation revealed that PPH incidence steadily increased by every definition examined, including a ≥3 g/dL hemoglobin drop. The 45% relative increase in this laboratory‐based, objective outcome demonstrates that increases in PPH cannot be fully attributed to changes in measurement, coding, or transfusion practice. These findings underscore the need to define patient and system factors that underlie the continued rise in PPH. In the meantime, enhanced prevention and real‐time PPH surveillance are imperative.

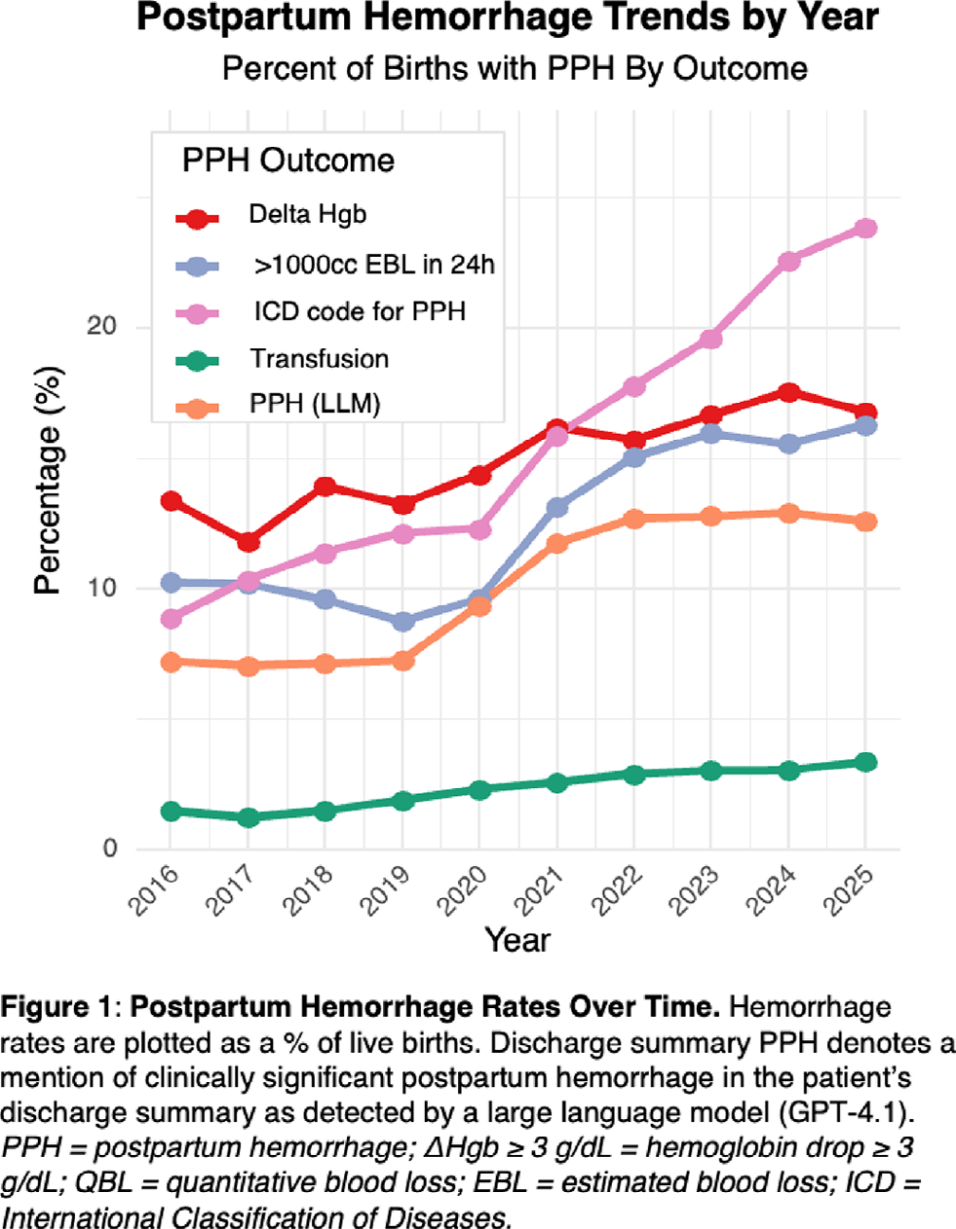

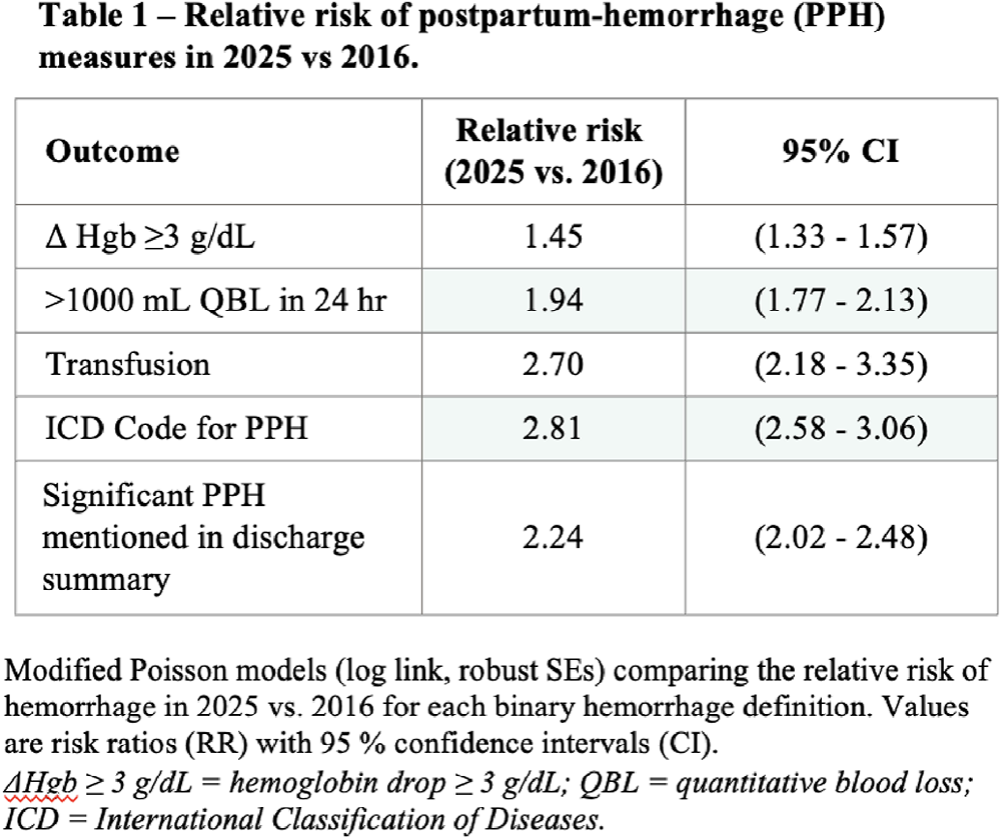

**10:30 AM – 12:00 PM**

## 756 | Postpartum **Lipid Screening in Patients With Pregestational Diabetes**

Christine E. Henricks; Caroline Magalhaes Juarez; Donald D. McIntire; Kristie R. Wilburn‐Wren; Elaine L. Duryea; David B. Nelson; Robert B. Martin

University of Texas Southwestern Medical Center, Dallas, TX

**Objective**: To characterize lipid profiles within the first year postpartum among individuals with diabetes mellitus (DM), a high‐risk, predominantly young population not captured in traditional 10‐year cardiovascular disease (CVD) risk calculators.

**Study Design**: This is a prospective observational study of postpartum patients with pregestational DM enrolled in a comprehensive postpartum care program. Individuals who delivered October 2020–October 2024 and had at least one lipid panel beyond 3 months postpartum were included. Hemoglobin A1c (HbA1c) levels were also measured at this time. Demographic and clinical data were abstracted from the medical record. Lipid levels were compared by stratified HbA1c values using Kruskal‐Wallis test.

**Results**: Of 151 eligible patients, 68 (45%) underwent lipid screening. The median age was 33 years, with 82% under age 40. Most had type 2 DM (91%) and/or hypertension (44.3%). Median lipid values were: total cholesterol 195 [162, 229]; LDL 107 [86, 137]; HDL 46 [38, 53]; and triglycerides 171 [117, 232] (Table 1). Overall, 46% had total cholesterol ≥200 mg/dL, 12% had LDL ≥ 160 mg/dL, and 59% had triglycerides ≥ 150 mg/dL. Notably, 90% of patients had an LDL ≥ 70 mg/dL, the recommended target value for primary prevention of atherosclerotic CVD in those with high‐risk conditions such as DM. Total cholesterol, LDL, HDL, and triglycerides did not vary significantly with worsening hemoglobin A1c values (Table 2).

**Conclusion**: Although increasing HbA1c was not significantly associated with changes in lipid levels, triglyceride levels were frequently elevated in this high‐risk cohort. While primary prevention guidelines are well established for patients aged 40 and older, there are limitations to current cardiovascular risk calculators in younger populations. This study highlights the need for targeted prevention strategies in at‐risk postpartum patients.

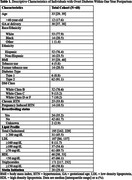

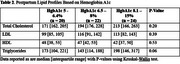

**10:30 AM – 12:00 PM**

## 757 | **What's** My **Method? ​A Randomized Controlled Trial for Contraceptive Self‐Efficacy Using a Contraceptive Education Game**

Clara Bertozzi‐Villa^1^; Tiffany Jordan^2^; Sonia Watson‐Miller^3^; Anderson Langdon^2^; Erin Sabato^4^; Elena Bertozzi^4^; Georgios Doulaveris^5^

^1^Montefiore‐Einstein, Bronx, NY; ^2^Barbados Family Planning Association, Bridgetown, Saint Michael; ^3^Barbados Community College, Bridgetown, Saint Michael; ^4^Quinnipiac University, Hamden, CT; ^5^Montefiore Medical Center/Albert Einstein College of Medicine, Montefiore Medical Center/Albert Einstein College of Medicine, NY

**Objective**: To evaluate whether a birth control education game called What's My Method? (WMM) would impact user scores on a metric of contraceptive self‐efficacy (CSE) as a reflection of reproductive empowerment.

**Study Design**: This study is a randomized controlled trial (RCT) conducted at the Barbados Family Planning Association clinic in Bridgetown using a modified version of the CSE sub‐Saharan Africa (CSESSA) scale developed by Whiting‐Collins et al. We hypothesized that those who play WMM before their contraceptive counseling session would score higher on the CSE survey than those who received standard counseling only. The primary outcome for our study is the difference in CSE score between those who did and did not play WMM. Our secondary outcomes are a comparison of method adoption between groups and a subjective assessment of the intervention. We conducted baseline population testing of the modified CSE score prior to the intervention. WMM was created by a team of game developers, experts in games for health, illustrators, and reproductive health specialists.

**Results**: The baseline testing for the modified CSE demonstrated universally high scores. For the RCT, 73 participants were enrolled. Three were excluded, and 70 records were analyzed; 34 (48.6%) in the control and 36 (51.4%) in the WMM group. Baseline characteristics were similar. There was not a difference in primary outcome of CSE score between groups (Table 1) or in the secondary outcome of method adoption. In the secondary outcome of subjective assessment, 94% of participants reported that WMM had a positive impact on their session, 89% said they'd play again if they could, and 72% were interested in sharing WMM with friends and family (Table 2).

**Conclusion**: Although there was not a difference in primary outcome, participants were overwhelmingly positive in their response to the game, and felt that it contributed to their counseling about contraception. Strengths of this study include its RCT design and deployment a population with high health literacy.

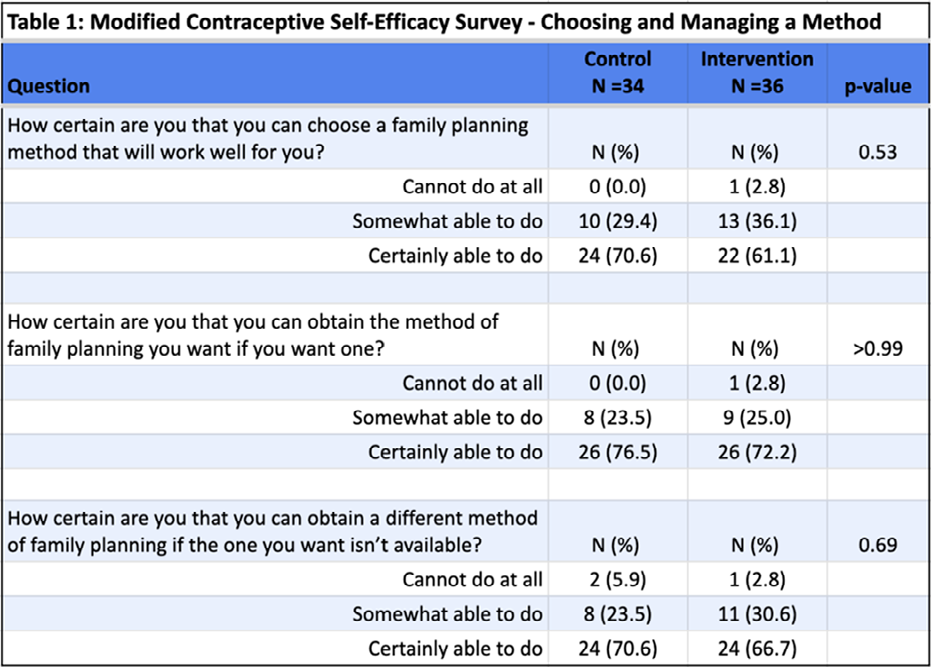

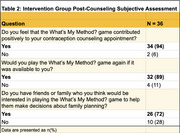

**10:30 AM – 12:00 PM**

## 758 | Respiratory **Outcomes in Preterm Neonates With Different Timing of Rescue Betamethasone Administration Before Delivery**

Claribel Solorio^1^; Brandon Ganjineh^1^; Ronald Clyman^1^; Katelin Kramer^1^; Cinthia Blat^2^; Melissa G. Rosenstein^1^

^1^University of California, San Francisco, University of California, San Francisco, CA; ^2^Department of Obstetrics, Gynecology and Reproductive Sciences, University of California, San Francisco, San Francisco, CA

**Objective**: At our institution, we prophylactically administer rescue betamethasone once eligible in patients at risk of delivery <28 weeks due to data that a rescue course decreases the risk of serious grade 3/4 interventricular hemorrhage (sIVH) in infants born <28 weeks. Since respiratory benefits of an initial betamethasone course wane with time, we examined whether respiratory effects of rescue betamethasone course also waned with time if infants delivered after 28 weeks.

**Study Design**: Patients who received a rescue betamethasone course and delivered a singleton between the gestational age of 28 0/7 through 33 6/7 at the University of California San Francisco between January 2015 and December 2024 were studied. All patients received an initial course at <28 weeks. Patients were retrospectively divided into two groups: (>10 dtd) received a rescue course > 10 days to delivery and (<10 dtd) received a rescue course < 10 days to delivery. Respiratory outcomes between groups were compared.

**Results**: Of the 74 studied patients, 33 (45%) had rescue betamethasone > 10 dtd and 41 (55%) had rescue betamethasone < 10 dtd. No difference in maternal baseline characteristics were seen. The group > 10 dtd were more likely to have received an initial betamethasone course and their rescue course at an earlier gestational age (24.6 vs. 26.7 weeks; *p* = <0.001) and (28.7 vs. 30.0 weeks; *p* = 0.002), respectively. The group > 10 dtd were more likely to deliver at a more advanced gestational age (32.1 vs. 30.0 weeks; *p* = 0.0002) and had higher birthweights (1,745 vs.1395 grams; *p* = 0.022). Logistic regression models adjusted for gestational age, birthweight and fetal sex showed that group > 10 dtd were more likely to have 5‐minute APGAR scores < 7 [aOR 8.46 (2.47–36.0), *p* = 0.0015] and were more likely to be intubated within 28 days of life [aOR 11.83 (3.09–60.00), *p* = 0.00091].

**Conclusion**: Although prophylactic rescue betamethasone decreases the risk of sIVH in infants delivering before 28 weeks, after 28 weeks a more judicious approach (waiting until delivery is more imminent) may improve neonatal respiratory outcomes.

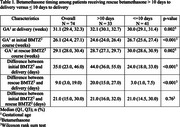

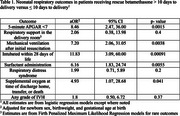

**10:30 AM – 12:00 PM**

## 759 | **Intra‐**Amniotic **Pressure Changes During Fetoscopic Laser Photocoagulation for Twin‐twin Transfusion Syndrome**

Claudio V. Schenone^1^; Ghadi Saikaly^2^; Alireza A. Shamshirsaz^1^; Eyal Krispin^1^

^1^Boston Children's Hospital, Harvard Medical School, Boston, MA; ^2^Boston Children's Hospital, Boston, MA

**Objective**: To describe intra‐amniotic pressure (IAP) changes during fetoscopic laser photocoagulation (FLP) and amnioreduction (AR) in pregnancies complicated by twin‐twin transfusion syndrome (TTTS).

**Study Design**: This was a retrospective analysis of monochorionic diamniotic twin pregnancies complicated by TTTS who underwent FLP and AR at our center between May 2024 and July 2025. We obtained IAP in mmHg before and after FLP and AR using a pressure‐gauge transducer (TruWave, Edwards Lifesciences Corp.) connected to the fetoscopic port. We estimated median and interquartile ranges (IQR) for opening and closing IAP. Paired t‐test assessed differences between the opening and closing IAP. Spearman rho (ρ) assessed the correlation between the amount of amniotic fluid volume removed and the difference between the opening and closing IAP.

**Results**: Seventeen patients were included. The opening and closing IAP were 25.0 (20.0–31.5) and 20.5 (17.5–26.0) mmHg, respectively. There was a 5.2 mmHg drop in IAP following FLP and AR (*p* < .001). The net volume of amniotic fluid removed was moderately correlated with the change in IAP (ρ = 0.515, *p* = 0.04) (Figure 1).

**Conclusion**: IAP decreases in pregnancies complicated by TTTS following FLP and AR. The higher the amount of amniotic fluid volume removed, the greater the degree to which the IAP drops. Larger studies are needed to validate our results and explore the relationship between changes in IAP during FLP and pregnancy outcomes.

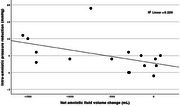

**10:30 AM – 12:00 PM**

## 760 | **Delivery Morbidity and Obstetric Outcomes Associated with Persistent Basal Plate Myometrial Fibers (BPMF)**

Cody Goldberger^1^; Juliana Lindberg^2^; Patricia Rekawek^3^; Tatyana Zinger^4^; Esther Adler^4^; Poonam Khullar^5^; Ashley S. Roman^6^; Rebecca H. Jessel^6^

^1^Department of Obstetrics and Gynecology, NYU Grossman School of Medicine, New York, NY; ^2^NYU Grossman School of Medicine, New York, NY; ^3^NYU Grossman Long Island School of Medicine, Mineola, NY; ^4^Department of Pathology, NYU Grossman School of Medicine, Long Island, NY; ^5^Department of Pathology, NYU Grossman School of Medicine, Mineola, NY; ^6^Division of Maternal‐Fetal Medicine, Department of Obstetrics and Gynecology, NYU Grossman School of Medicine, New York, NY

**Objective**: Placentas with evidence of basal plate myometrial fibers (BPMF) have been referred to as occult accreta or micro‐accreta. This histological finding of BPMF without intervening decidua is not infrequent and is often seen in clinically asymptomatic cases. There are conflicting data regarding the significance of these findings. The objective of this study was to evaluate obstetric morbidity at the time of the index delivery and in subsequent pregnancies for patients with evidence of BPMF.

**Study Design**: We performed an observational study of pregnancies complicated by pathologic evidence of BPMF at a large, urban health system between 9/3/2013 and 5/1/2025. Patients without pathologic specimens and with incomplete medical records were excluded. Charts were reviewed for demographic information, risk factors for placenta accreta spectrum (PAS), obstetrical morbidity during the index delivery including postpartum hemorrhage (PPH) (defined as QBL of >1L), uterine sparing surgical therapy, additional procedures, and/or ICU admission, and subsequent pregnancy outcomes.

**Results**: A total of 85 BPMF cases were identified. The median gestational age at delivery was 37 weeks. 88% of cases had at least one risk factor for PAS (Table 1). 27 deliveries (31.8%) were complicated by PPH, of which seven patients underwent peripartum hysterectomy. Of the 18 patients who had subsequent pregnancies, 14 carried at least one pregnancy to term, with no evidence of recurrent PAS at the time of delivery.

**Conclusion**: In our cohort, BPMF was appreciated in patients with risk factors for PAS, suggesting that BMPF exists on a continuum with more severe abnormal placentation. Further, pregnancies with evidence of BPMF on pathology were at increased risk of PPH but were not at increased risk of recurrent PAS in subsequent pregnancies. Ongoing research is needed to better understand the evolving and possibly morbid diagnosis of BPMF.

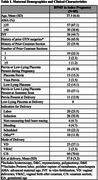

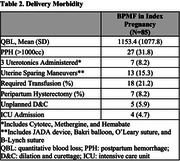

**10:30 AM – 12:00 PM**

## 761 | **Enhanced Group vs. Individual Prenatal Care in Preventing Preterm Birth—EMBRACE Randomized Comparative‐effectiveness Trial Exploratory Findings**

Daisy León‐Martínez^1^; Deborah Karasek^2^; Cinthia Blat^3^; Venise Curry^4^; Kristin Carraway^4^; Kimberly Coleman‐Phox^3^; Brittany D. Chambers Butcher^5^; Patience A. Afulani^6^; Bridgette Blebu^7^; Jennifer N. Felder^8^; Mary A. Garza^4^; Martha A. Tesfalul^3^; Christopher Downer^9^; Charles E. McCulloch^10^; Miriam Kuppermann^11^

^1^Division of Maternal‐Fetal Medicine at the University of California San Francisco, University of California San Francisco, CA; ^2^Oregon Health & Science University‐Portland State University School of Public Health, Portland, OR; ^3^Department of Obstetrics, Gynecology and Reproductive Sciences, University of California, San Francisco, San Francisco, CA; ^4^Central Valley Health Policy Institute at California State University, Fresno, Fresno, CA; ^5^Department of Human Ecology, College of Agricultural and Environmental Sciences, University of California, Davis, Davis, CA; ^6^Department of Obstetrics, Gynecology and Reproductive Sciences, University of California, San Francisco, Universiy of California San Francisco, CA; ^7^Lundquist Institute for Biomedical Innovation – Harbor‐UCLA, Los Angeles, CA; ^8^Department of Psychiatry and Behavioral Sciences, University of California, San Francisco, San Francisco, CA; ^9^Medical Education Program, University of California, San Francisco, Fresno, Fresno, CA; ^10^University of California, San Francisco, University of California San Francisco, CA; ^11^Department of Obstetrics, Gynecology and Reproductive Sciences, University of California, San Francisco, University of California San Francisco, CA

**Objective**: To compare the effect of two enhanced prenatal care models on preterm birth (PTB, < 37 weeks’ gestation).

**Study Design**: EMBRACE is a pragmatic, two‐arm, randomized comparative‐effectiveness trial evaluating depression (primary), care experiences (secondary), and PTB (exploratory) among Medicaid‐eligible pregnant people assigned to enhanced group prenatal care (eGPC) vs individual care enhanced (eIPC) by California's Comprehensive Perinatal Services Program. Primary and secondary outcomes of this analysis, which we prespecified as exploratory due to sample size constraints, are PTB (<37 weeks’ gestation) and mean gestational age at birth, respectively. Outcome data were obtained from hospital records and participant interviews. We conducted primary analyses by intention‐to‐treat and subgroup analyses by prenatal care visit attendance and self‐reported race and ethnicity (Black, Latine). To estimate the intervention effect, mixed logistic regression models and restricted maximum likelihood mixed linear regression models (adjusted for language of interview with random intercepts for provider) were performed.

**Results**: Among 652 participants (*n* = 281 eGPC, *n* = 371 eIPC), self‐identified race and ethnicity was 7.4% Black, 72.2% Latine, and 11.2% White. Participants assigned to eGPC were less likely to experience PTB compared to those assigned to eIPC, although the estimate did not reach statistical significance at the alpha 0.05 level (8.7% vs 12.2%; aOR 0.67, 95% CI [0.39, 1.14]). No difference was observed in gestational age at birth (38.7 ± 2.3 vs 38.7 ± 2.1 weeks; aMD 0.03, 95% CI [–0.32, 0.39]). Subgroup analyses suggested higher effectiveness with more eGPC attendance and Latine ethnicity, although differences were not statistically significant (Figure 1). Black subgroup analyses were limited by sample size.

**Conclusion**: This exploratory analysis suggests eGPC may reduce PTB rates among low‐income individuals. Larger studies are needed to definitively determine whether this model of care reduces preterm birth risk, particularly among low‐income Black and Latine populations.

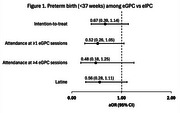

**10:30 AM – 12:00 PM**

## 762 | Respectful **Care and Safe Reduction of NTSV Cesarean Delivery Through Standardized QI Interventions**

Dajana Alku^1^; Delphina Maldonado^1^; Marie Russelman^1^; Rosanne Vertichio^1^; Kakita Smith^1^; Genevieve Sicuranza^1^; Melissa Peskin‐Stolze^2^; Anju Suhag^3^

^1^NYU Grossman Long Island School of Medicine, Mineola, NY; ^2^NYU Langone Health, Long Island, NY; ^3^NYU Langone Long Island, Mineola, NY

**Objective**: Racial disparities persist in New York, with Black birthing people facing nearly triple the mortality of White counterparts. In 2024, NTSV cesarean rates at NYU Langone—Long Island exceeded benchmarks: 47.1% in Black and 37.4% in Asian patients. This QI initiative aimed to reduce rates by 36% and 20%, targeting ≤30% overall.

**Study Design**: This single‐site, multidisciplinary QI project began in September 2023 with baseline data revealing elevated and racially disparate NTSV cesarean rates. In November 2024, provider education was launched via Grand Rounds, emphasizing evidence‐based labor management and documentation. Reinforcement included case reviews, education sessions, safety huddles, and high‐reliability organization rounds. A Race & Ethnicity Workgroup was added in March 2025. The intervention aligned with NYSPQC Respectful Care drivers (Figure 1). Outcomes were tracked monthly, including race‐stratified NTSV rates and balancing metrics (QBL >1000 mL and PC–06, unexpected newborn complications).

**Results**: By June 2025, the overall NTSV rate declined to 22.8%, with 6 of the last 7 months below 30%. Rates fell notably among Black (61.5% to 38.7%) and Asian (44.0% to 17.5%) patients, narrowing the Black–White gap from 34.3 to 14.2 points (Figure 2). Balancing metrics remained stable: QBL at 3.8–10.1%; PC–06 at 26.8–37.5 per 1000 births.

**Conclusion**: Equity‐focused, standardized labor interventions safely reduced overall and race‐stratified NTSV cesarean rates. The strategy, rooted in targeted universalism and respectful care, supports sustainable improvement. Future analysis will explore comorbidities and cesarean indications by race to identify ongoing opportunities for equitable care.

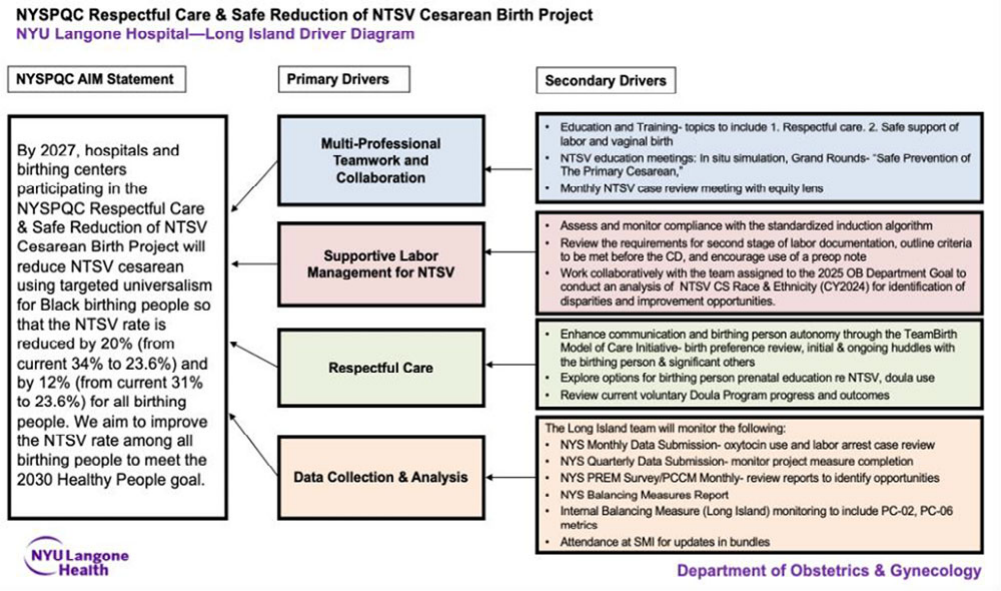

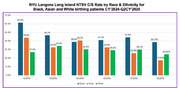

**10:30 AM – 12:00 PM**

## 763 | **Artificial Intelligence‐Based Prediction of Prolonged Post‐Cesarean Hospitalization: The Power of XGBoost**

Daniel Gabbai^1^; Itamar Gilboa^1^; Lee Reicher^2^; Yariv Yogev^3^; Emmanuel Attali^1^

^1^Lis Hospital for Women's Health, Tel Aviv Sourasky Medical Center, Lis Hospital for Women's Health, Tel Aviv Sourasky Medical Center, Tel Aviv; ^2^Lis Hospital for Women's Health, Tel Aviv Sourasky Medical Center, Tel Aviv, HaMerkaz; ^3^Lis Hospital for Women's Health, Tel Aviv Sourasky Medical Center Gray Faculty of Medicine, Tel Aviv University, Israel, Lis Hospital for Women's Health, Tel Aviv Sourasky Medical Center, Tel Aviv

**Objective**: To apply machine learning techniques for the prediction of prolonged hospitalization in women undergoing cesarean delivery (CD), facilitating early recognition of at‐risk patients and informed perioperative planning

**Study Design**: A retrospective cohort study was conducted at a tertiary medical center (2012–2024), including all women who underwent CD. Prolonged hospitalization was defined as length of stay ≥5 days (Normal stay – 72 hours). Demographic, clinical, obstetric, and neonatal variables were used as candidate predictors. Data preprocessing and model development were performed in R using the XGBoost package. The dataset was split into training (70%) and test (30%) sets. Model performance was evaluated by area under the ROC curve (AUC), and variable importance was assessed by model gain and SHAP (SHapley Additive exPlanations) value.

**Results**:

1. A total of 32,639 women underwent CD, with 1969 (6.0%) experiencing prolonged hospitalization.
2. The top predictors by model gain included gestational age at delivery (21%), maternal age (18%), neutrophil‐lymphocyte ratio (14%), antibiotic treatment for peripartum fever (13%), birth weight (11%), pre‐pregnancy BMI (10%), pre‐delivery platelet count (9%), and surgery duration (8%(.
3. SHAP analysis identified gestational age at delivery, antibiotic treatment for peripartum fever, birth weight, maternal age, and surgery duration as the leading contributors to prolonged hospitalization risk.
4. The final model incorporated 28 clinical variables and achieved an AUC of 0.83 on the test set, indicating good discrimination.

**Conclusion**: An XGBoost machine learning model accurately predicts prolonged hospitalization after CD. This approach can help clinicians identify women at increased risk for extended post‐cesarean stay, potentially guiding resource allocation and perioperative management.

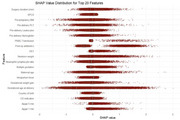

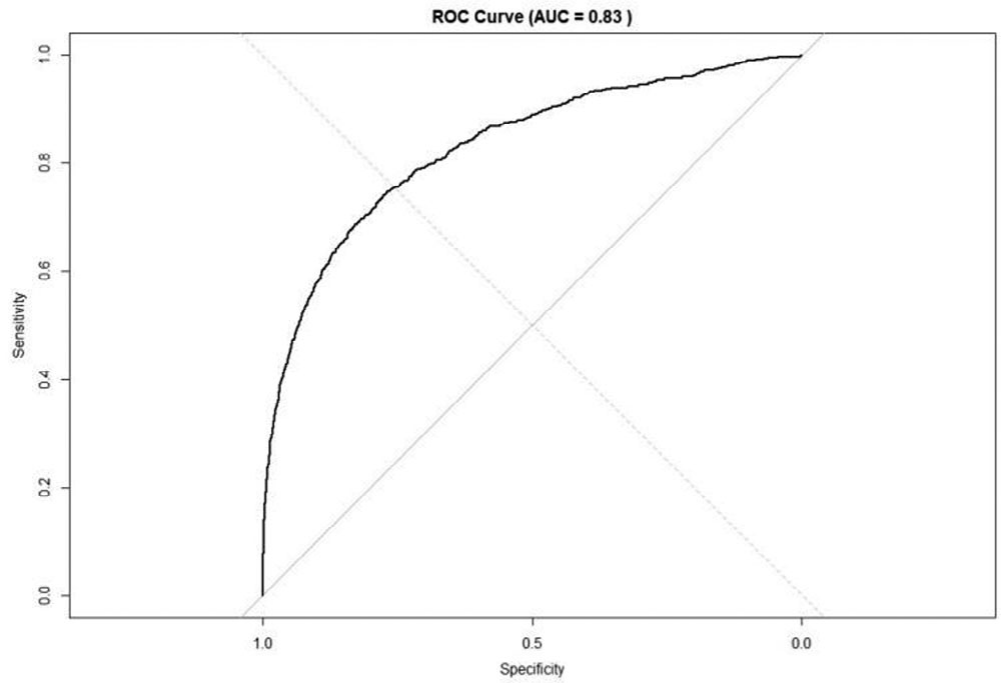

**10:30 AM – 12:00 PM**

## 764 | **Effect of** Improved **Uterotonic Availability on Postpartum Hemorrhage and Maternal Morbidity After Vaginal Delivery**

Danielle L. Chirumbole^1^; Towana Sims^2^; Stacie Denning^2^; Sheena Glover^2^; Lauren Shubert^2^; Courtney Thompson^2^; Adewunmi Babalola^2^; Tara Barrick^3^; Sharon Burks^4^; Manisha Gandhi^1^; Christina Davidson^1^

^1^Baylor College of Medicine, Houston, TX; ^2^Texas Children's, Houston, TX; ^3^Texas Children's Hospital, Texas Children's Hospital, TX; ^4^Texas Children's Hospital, Houston, TX

**Objective**: After implementation of a hemorrhage risk stratification system and management bundle in 2019, our hospital demonstrated a reduction in hemorrhage‐related severe maternal morbidity (SMM‐H). We then noted a plateau in PPH rates and a gradual increase in SMM‐H after 2020, with the majority of PPH after vaginal deliveries (VD) occurring in those deemed low‐risk. Consequently, we implemented a strategy to improve the availability of 2nd‐line uterotonics in the delivery room regardless of PPH risk. The objective of this study is to evaluate the effect of this intervention on rates of PPH and SMM‐H after VD.

**Study Design**: Prior to 7/2024, misoprostol was available in all delivery rooms. Methylergonovine and carboprost, considered more effective, were not in the room at delivery unless requested by the provider. After 7/2024, all 3 uterotonics were made available in the room at initiation of 2^nd^ stage labor. We reviewed all deliveries from the 10 months before and after this change (10/2023–7/2024, 9/2024–6/2025). Deliveries from 8/2024 were excluded to allow for washout. We investigated differences in PPH and SMM‐H rates in VD pre‐ and post‐intervention. Chi‐square was used to determine statistical significance.

**Results**: There was no difference in VD PPH pre‐ vs post‐intervention (6.09% vs 5.92%, *p* = 0.78). Similarly, there was no significant change in VD SMM‐H (22.35% vs 19.80%, *p* = 0.51). The rates of PPH and SMM‐H in low‐risk VD patients were 5.4% and 17.8% pre‐ compared to 5.4% and 15.7% post‐intervention. These differences were not statistically significant. Low‐risk patients comprised 57.31% of VD with PPH after the intervention. Of VD requiring 2nd‐line uterotonics, misoprostol was the most common first agent prior to the intervention (60%) vs methylergonovine after (46%) which was statistically different from prior (*p* < 0.0001).

**Conclusion**: Improving 2nd‐line uterotonic availability in the delivery room did not improve VD PPH rates or SMM‐H but did change 2nd‐line uterotonic choice. PPH risk stratification may not alter PPH rates or morbidity but may improve timeliness of blood product administration.

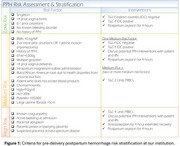

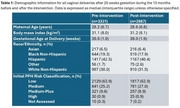

**10:30 AM – 12:00 PM**

## 765 | **Oral** Antihypertensives **for Pregnancy Prolongation in Preterm Preeclampsia: A Systematic Review and Meta‐Analysis**

David J. Rivera^1^; Insaf Kouba^2^; Ariane Chabanne^3^; Krista Howard^4^; Heisy B. Asusta^5^

^1^Orlando Health Bayfront Hospital, St Petersburg, FL; ^2^Johns Hopkins All Childrens, St. Petersburg, FL; ^3^Orlando Health Bayfront Hospital, St. Petersburg, FL; ^4^Texas State University, San Marcos, TX; ^5^Orlando Health, St Petersburg, FL

**Objective**: This study evaluated existing data on oral antihypertensives for prolonging pregnancies complicated by severe hypertension in preterm preeclampsia.

**Study Design**: An electronic literature search in the PubMed/ MEDLINE, Google Scholar, EMBASE databases and CENTRAL (the Cochrane Library) was performed. Observational and randomized controlled studies reporting pregnancy prolongation outcomes among women with preeclampsia who were treated with oral antihypertensive medications were included. Of the 62 records screened,19 were eligible for meta‐analysis using the PRISMA guidelines. The following secondary complication measures of interest were quantitatively synthesized: HELLP, eclampsia, placental abruption, and neonatal respiratory distress. Statistical meta‐analyses were performed using R 4.5.1 meta package. PROSPERO Registration: CRD420251090292.

**Results**: Two separate models were created for studies reporting mean days (*n* = 14; total*n* = 1119) and those reporting median days (*n* = 5; total*n* = 424). Random‐effects models were applied due to substantial heterogeneity (I^2^ = 97.0% and 96.3%, respectively). For the studies that reported means, the pooled estimate for prolongation of pregnancy was approximately 9.79 days (95% CI: 7.15, 12.42). For studies that reported medians, the pooled prolongation was estimated at 10.62 days (95% CI: 4.05, 17.20). Findings across both models were consistent. Meta‐analyses showed that the secondary complications were generally rare, but significant differences between the studies caused some variations in the results.

**Conclusion**: Oral antihypertensive medications are associated with an approximate 10‐day prolongation of pregnancy in preterm preeclampsia, with a low incidence of maternal and neonatal complications. These findings reinforce prior evidence and underscore the value of oral antihypertensives as a key component of expectant management, warranting further study to optimize pregnancy outcomes.

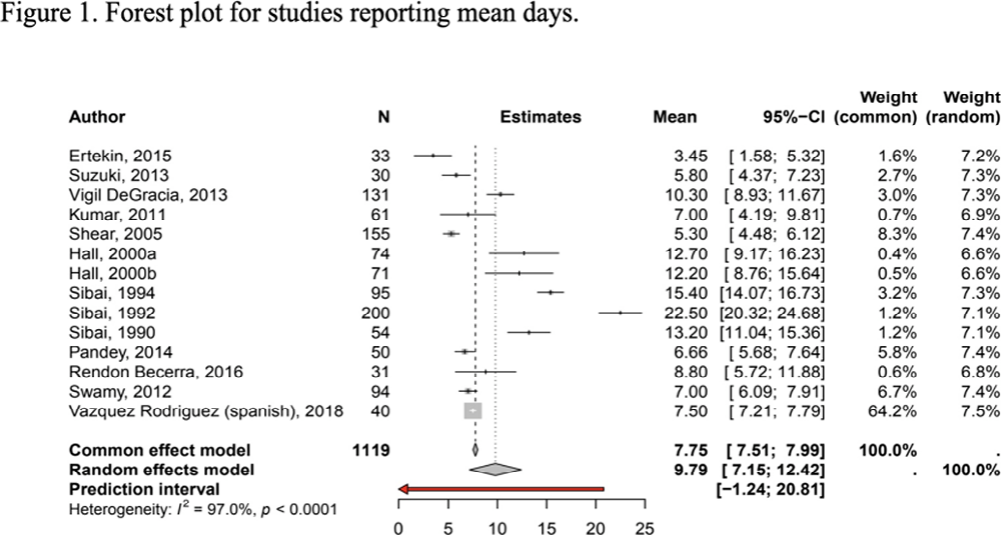

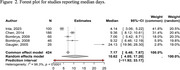

**10:30 AM – 12:00 PM**

## 766 | **Pediatric and Family Medicine Providers' Willingness and Barriers to Screen for Urgent Maternal Warning Signs**

Veronica Maria Pimentel^1^; Donna H. Kim^2^; Nicole Barreto^3^; Dorothy B. Wakefield^4^; Rebecca Crowell^5^

^1^Trinity Health Of New England / Hartford, Trinity Health Of New England / Hartford, CT; ^2^Frank H. Netter School of Medicine / Quinnipiac University; ^3^Bridgeport Hospital, Hamden, CT; ^4^Trinity Health Of New England, Trinity Health Of New England / Hartford, CT; ^5^Trinity Health Of New Engalnd, Trinity Health Of New England, CT

**Objective**: The CDC's Hear Her Campaign highlights urgent maternal warning signs to prevent pregnancy‐related deaths and tackle maternal health disparities. Yet, maternal mortality and disparities remain high, with 2/3 of deaths occurring post‐delivery, when postpartum patients have multiple contact with pediatric and family medicine providers. Our objectives were to assess pediatric and family medicine providers’ willingness to screen for urgent maternal warning signs during routine well‐child visits (WCV) and their perceived barriers to screening implementation.

**Study Design**: We developed and electronically distributed a questionnaire to pediatric (Peds) and family medicine (FMs) providers, assessing their demographics, willingness to screen postpartum patients for warning signs during routine WCV, and perceived barriers to screening implementation. Data was analyzed using chi‐square and t‐tests.

**Results**: 175 participants (69 Peds;106 FMs) completed the questionnaire. Peds and FMs were similar in age, sex, ethnicity, and training years (*p* >0.05). Peds were less likely to identify as White (*p* = 0.002) and more likely to work in academic centers (*p* < 0.001). Both groups saw equal percentages of Black and Indian/Native American patients (*p* >0.05). Peds saw fewer publicly insured patients (*p* = 0.021). While more Peds agreed that there are major racial/ethnic disparities in maternal mortality (89.9% vs. 62.3%, *p* < 0.001) and “almost always” screened for postpartum depression (85.5% vs. 56.6%, *p* < 0.001), they were less aware of the urgent maternal warning signs (24.6% vs. 79.3%, *p* < 0.001), less confident in recognizing these signs (7.7% vs. 41.5%, *p* < 0.001), and less willing to screen for physical warning signs (65.2% vs. 81.1%, *p*  = 0.049). The top three reported barriers to screening for warning signs were lack of time (62.3%), lack of provider training (43.4%), and lack of reimbursement (32.0%).

**Conclusion**: Peds and FMs should be trained to screen postpartum patients for urgent warning signs. Barriers to screening should be addressed so they can become vital partners in tackling the maternal mortality and disparity crisis.

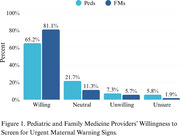

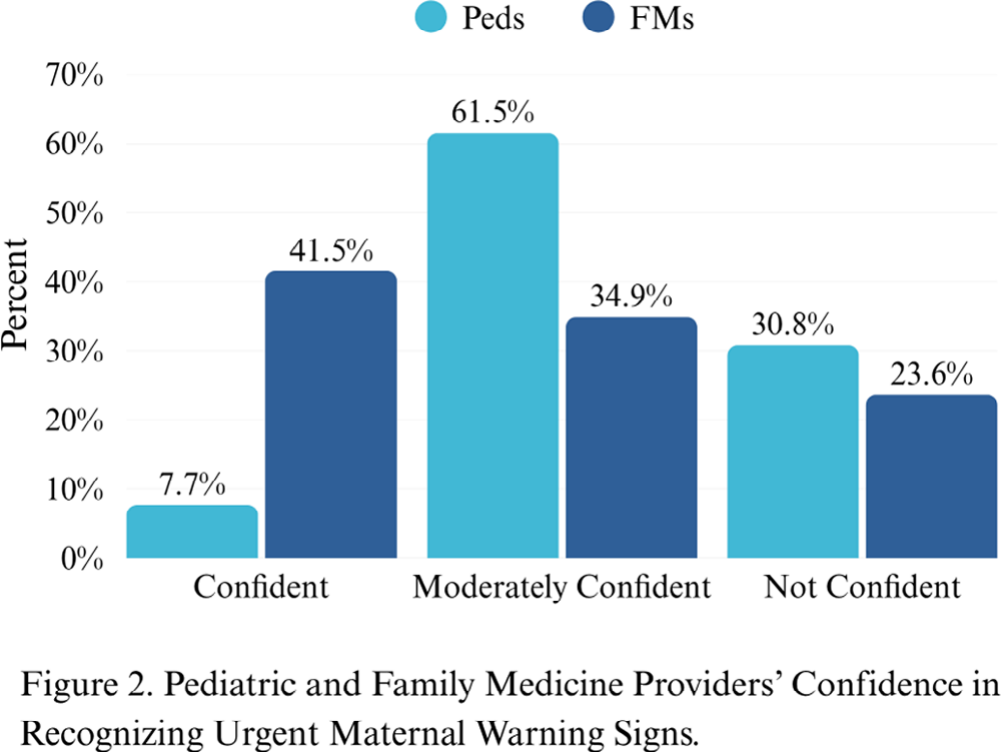

**10:30 AM – 12:00 PM**

## 767 | Fetoscopic **Spina Bifida Repair Offers Similar Benefits to Open Approach Based on Imaging Outcomes**

Duyhoang Q. Dinh^1^; Bree A. Goodman^2^; Jesse Vrecenak^1^; Jennifer M. Strahle^2^; Sasidhar Karuparti^1^; Jagruti Anadkat^1^; Nandini Raghuraman^2^; Jeannie C. Kelly^3^; Katherine H. Bligard^1^

^1^Washington University School of Medicine, Saint Louis, MO; ^2^Washington University School of Medicine, St. Louis, MO; ^3^Washington University School of Medicine, Washington University School of Medicine, MO

**Objective**: Fetoscopic repair of open neural tube defects (ONTDs) may reduce maternal and obstetrical risks compared to open repair via hysterotomy. However, it is unclear whether the fetoscopic approach confers similar fetal/neonatal benefits, specifically postoperative imaging and the need for cerebrospinal fluid (CSF) diversion.

**Study Design**: This retrospective cohort study included all in‐utero ONTD repairs performed between 2020 and 2024 at a single fetal care center. All repairs used an identical multi‐layer myofascial flap. The primary outcome was postoperative lateral ventricle (LV) growth rate (mm/week). Secondary outcomes included trajectories of additional biometric markers on pre‐ and post‐operative ultrasounds and incidence of CSF diversion within 12 months after delivery. Outcomes were compared between fetoscopic and open repairs using appropriate statistical methods.

**Results**: Among 36 patients, 24 (66.7%) underwent open and 12 (33.3%) underwent fetoscopic repair. Myeloschisis was more frequent in the fetoscopic group (58.3% vs. 16.7%, *p* = 0.02), and LV width at diagnosis trended toward significance (11.6 mm for fetoscopic vs. 9.8 mm for open, *p* = 0.06). All other preoperative characteristics were similar. Postoperative LV growth rates did not differ between groups (0.56 mm/week for fetoscopic vs. 0.21 mm/week for open, *p* = 0.14). Cerebellar and head growth rates were similar, and resolution of “banana” and “lemon” signs occurred at comparable rates. The mean gestational age at delivery was 35.7 weeks for fetoscopic and 36.3 weeks for open repair (*p*  = 0.89), and rates of postnatal CSF diversion were not significantly different.

**Conclusion**: In our cohort, fetoscopic and open ONTD repair resulted in similar postoperative prenatal imaging findings and rates of postnatal CSF diversion, despite a higher proportion of myeloschisis in the fetoscopic group. The findings suggest that fetoscopic repair offers comparable fetal/neonatal benefit to open repair. Given its potential for reduced maternal and obstetric risk, fetoscopic repair should be offered for all patients considering in‐utero ONTD repair.

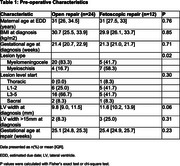

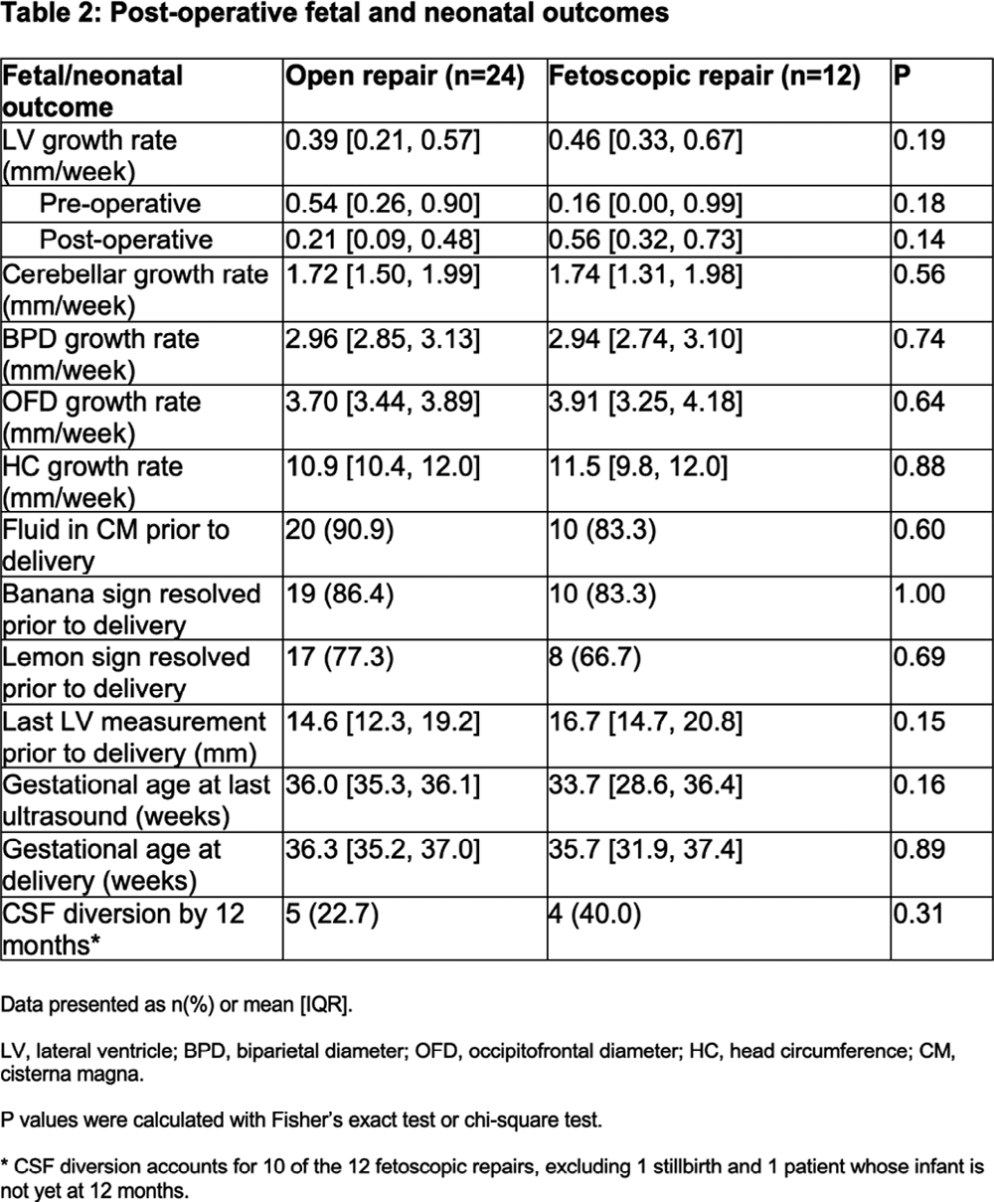

**10:30 AM – 12:00 PM**

## 768 | Multidimensional **Identity and Experiences of Racism and Discrimination Among Black Perinatal Women**

Sarah C. Haight^1^; Karen Sheffield‐Abdullah^2^; Aryana Daye^2^; Kayla Blades^3^; E. Nicole Nicole Teal^4^

^1^University of Georgia, Athens, GA; ^2^University of North Carolina at Chapel Hill, Chapel Hill, NC; ^3^The George Washington University, Washington, DC; ^4^Division of Maternal Fetal Medicine, Department of Obstetrics, Gynecology and Reproductive Sciences, University of California San Diego, San Diego, CA

**Objective**: Intersectionality theory posits how multiple social identities (e.g. race, gender, class) interact to create systems where people experience discrimination differently. Existing work among Black women has not explored intersections beyond race, pregnancy status, and gender. This study aims to compare experiences of racism and discrimination by multi‐dimensional identities in a sample of Black perinatal women.

**Study Design**: This study uses 2021 survey data of individuals who self‐identified as Black women >18 years old with a live birth at a university hospital system from 2019–2021. Surveys included demographic information, the Perceived Racism Scale, and the Perceived Discrimination Scale. Demographics included socioeconomic status (SES; low: < bachelor's degree or < $20,000 annual income), marital status, and age (young: <26). Latent class analysis identified four groups with additional marginalized identities beyond race and gender (Table 1). One‐way Kruskal‐Wallis tests determined whether scale medians differed by group. Modified Poisson regression calculated prevalence ratios (PR) and 95% confidence intervals (CI) for the likelihood of scoring in the top quartile on scales.

**Results**: Among 200 Black women 3–32 months post‐delivery, median perceived racism score was 99.5 (IQR 71–137.5; possible range 0–337) and did not differ by group (*p* = 0.3; Table 1). For experiences of discrimination, the median score was 29 (IQR 22–35; possible range 9–47) and did not differ by group (*p* = 0.1). The likelihood of experiencing the top quartile of racism did not differ by group (Table 2). However, compared to Group 1 (no additional marginalized identities), Group 3 (PR 2.4, 95% CI 1.2–4.5) and Group 4 (PR 2.3, 95% CI 1.1–4.9) were twice as likely to report top quartile discrimination.

**Conclusion**: In this sample of Black women, additional marginalized identities including low SES, being unmarried, and being younger heightened experiences of discrimination reported postpartum. Findings support the need for perinatal practices and policies to consider an intersectional lens.

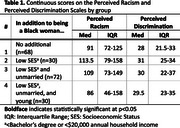

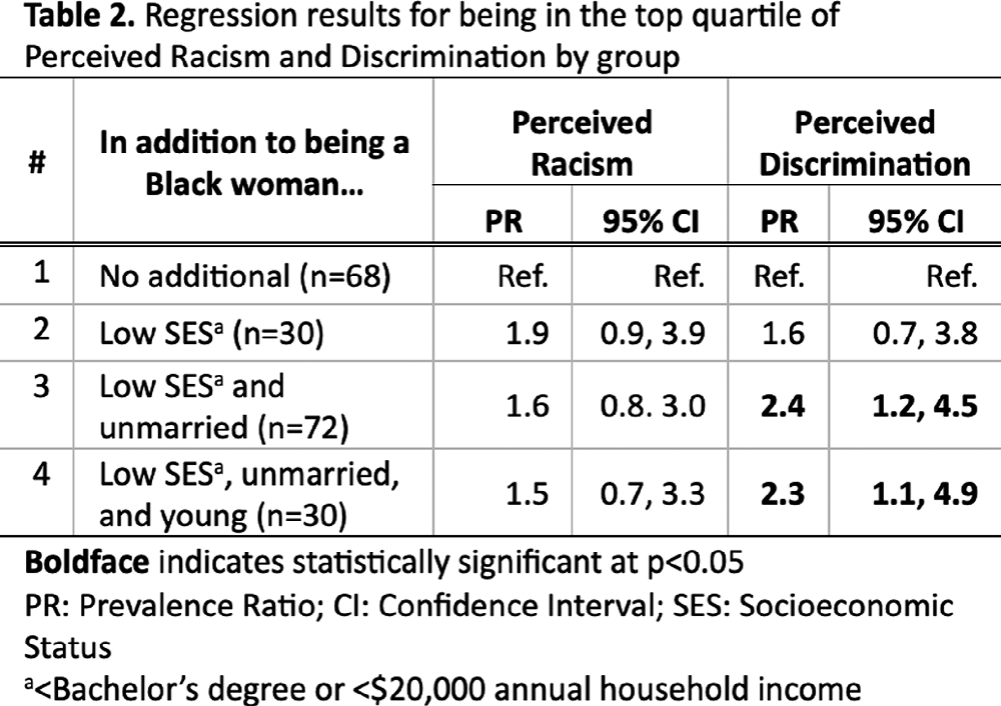

**10:30 AM – 12:00 PM**

## 769 | **Contrast‐Enhanced Ultrasound to Characterize Postpartum Uterine Circulation With Placenta in Situ for Placenta Accreta Spectrum**

Elias Kassir^1^; Edgar A. Hernandez‐Andrade^2^; Donatella Gerulewicz^2^; Farah H. Amro^2^; Eleazar E. Soto^1^; Ramesha Papanna^1^; Sean C. Blackwell^2^; Baha M. Sibai^2^

^1^University of Texas Health Science Center in Houston, McGovern Medical School, Houston, TX; ^2^Division of Maternal‐Fetal Medicine, Department of Obstetrics, Gynecology and Reproductive Sciences, McGovern Medical School at UTHealth Houston, Houston, TX

**Objective**: Alternate methods of management for placenta accreta spectrum (PAS) include leaving the placenta in situ postpartum until interval hysterectomy or placental resorption/expulsion; descriptions of placental vascular changes in this subset of patients are limited. Color Doppler mapping of the placenta is often used, but is suboptimal at characterizing slow‐moving vasculature. The use of ultrasound (US) contrast agents (UCA) may enhance visualization of placental flow. We used UCA to identify vascular changes over time in the uteroplacental interface in patients with placenta in situ after cesarean delivery (CD) for PAS.

**Study Design**: This was a case series of 5 patients suspected of having PAS based on US findings. Each patient underwent CD and retained the placenta with a plan for interval hysterectomy or awaiting placental resorption/expulsion. In patients opting for uterine preservation, contrast‐enhanced ultrasound (CEUS) was performed once the uterus appeared to have minimal vascularity on color Doppler sonography. In patients opting for interval hysterectomy, CEUS was performed at the final pre‐operative US. UCA (Lumason^®^ (sulfur hexafluoride lipid‐type A microspheres)) was administered intravenously and US images were obtained every 10 seconds using a combination of grayscale, color Doppler, and B‐mode techniques until UCA disappearance.

**Results**: CEUS was performed at 4.3, 5.9, 7.1, 9.1, and 14 weeks postpartum (PP). Uterine circulation enhancement was mainly seen using B‐mode technique within 1 minute of UCA administration. UCA remained stable for 8–10 minutes, followed by a signal reduction. Representative images of CEUS are shown in Figure 1; CEUS findings are summarized in Table 1.

Marked vascularization was seen in the uterus and placenta 4–5 weeks PP. After 7.1 weeks PP, a significant reduction in blood flow to the placenta can be observed, with absence of placental blood flow by 14 weeks PP.

**Conclusion**: UCA can be used to aid in visualization of uteroplacental perfusion. When the placenta is left in situ PP for PAS, vascularity begins to decrease at approximately PP week 7.

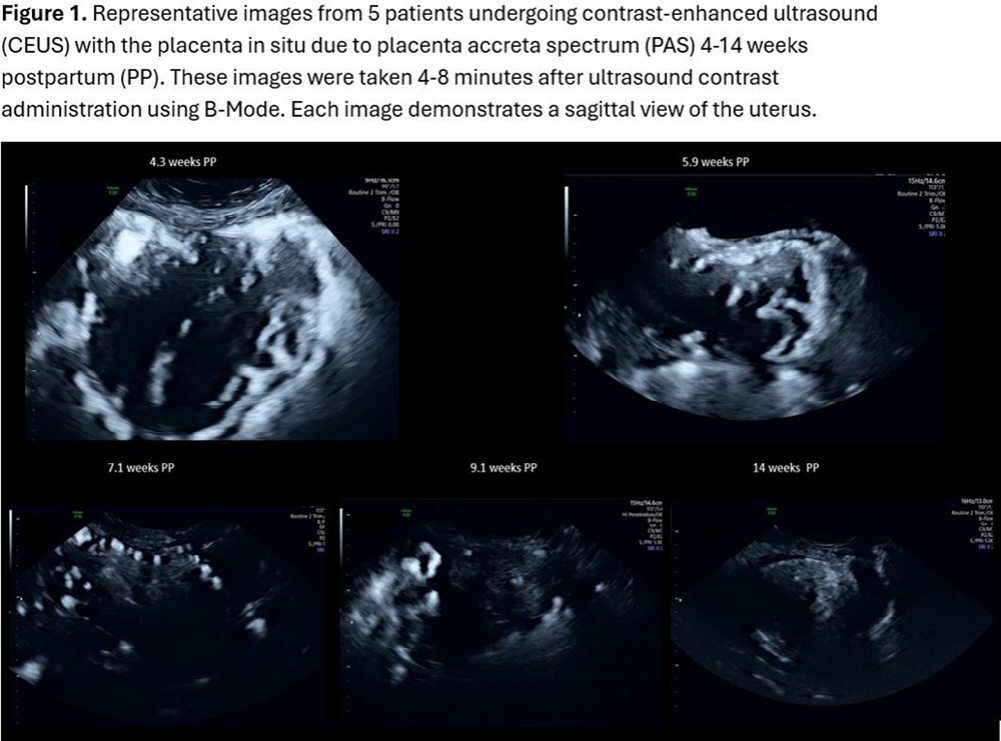

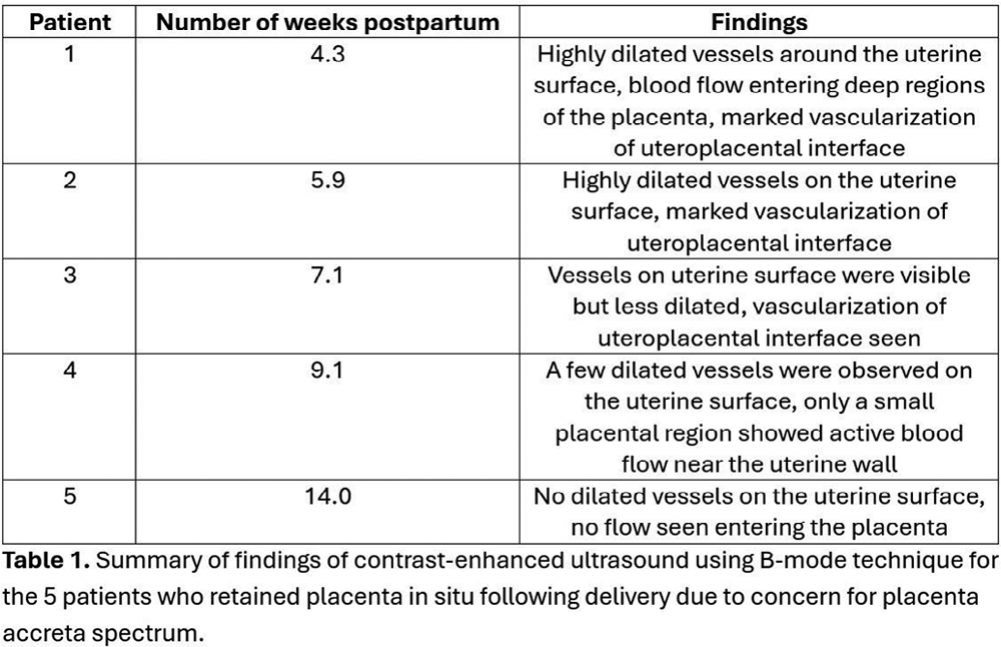

**10:30 AM – 12:00 PM**

## 770 | **The** Power **of Small: Abdominal Circumference <3% Is a High‐Yield Screen for Placental Pathology**

Eliel Kedar Sade^1^; Tamar Shyldkrot^2^; Ohad Mizrachi^2^; Letizia Schreiber^2^; Ilia Kleiner^3^; Eran Weiner^3^; Lior Kashani Ligumsky^2^

^1^Wolfson Medical Center, Department of Obstetrics and Gynecology, Wolfson Medical Center, Tel Aviv; ^2^Wolfson Medical Center, Department of Obstetrics and Gynecology, Wolfson Medical Center, HaMerkaz; ^3^Wolfson Medical Center, Department of Obstetrics and Gynecology, Holon, HaMerkaz

**Objective**: To evaluate sonographic and Doppler parameters as screening tools for placental vascular malperfusion in severe fetal growth restriction (FGR; BW< 3rd percentile) and moderate small‐for‐gestational‐age (SGA; BW 3–10th percentile).

**Study Design**: We retrospectively analyzed 346 singleton pregnancies delivered between 2016–2024 with BW< 10th percentile. Cases were stratified as BW< 3% (*n* = 243) or BW 3–10% (*n* = 103). Sonographic measures included abdominal circumference (AC) and estimated fetal weight (EFW) percentiles; Doppler indices included umbilical artery (UA), middle cerebral artery (MCA), and cerebroplacental ratio (CPR). Placental pathology was classified per Amsterdam Consensus (2016) as maternal (MVM) or fetal (FVM) vascular malperfusion.

**Results**: In BW < 3%, AC < 3% identified 90.6% of MVM and 93.4% of FVM lesions, while EFW< 3% detected 72.0% and 66.1%, respectively. Abnormal Doppler had lower sensitivity (MVM: 45.3% BW< 3%, 22.8% BW 3–10%) but higher specificity. Combining AC < 3% with EFW< 3% maintained high sensitivity for MVM (90.7% BW< 3%) and modestly improved specificity. In multivariable analysis for BW< 3% MVM, EFW< 3% remained significant after adjusting for maternal comorbidities and Doppler variables (OR 1.98, 95% CI 1.09–3.61, *p* = 0.026).

**Conclusion**: AC < 3% is the most effective screening parameter for detecting placental vascular malperfusion in growth‐restricted pregnancies. EFW < 3% adds independent predictive value, even after adjusting for Doppler findings. Doppler abnormalities, while less sensitive, increase specificity and are best applied as confirmatory tests in high‐risk cases. An approach using AC < 3% for screening, supplemented by EFW < 3% and targeted Dopplers, may reduce missed lesions and improve confidence in clinical management.

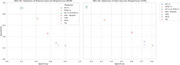

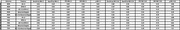

**10:30 AM – 12:00 PM**

## 771 | **A Natural Experiment: Impact of IV Fluid Restriction on Labor Outcomes**

Elizabeth Bloom^1^; Robert E. Jones^2^; Kathleen Clarke^2^; Sarah E. Little^3^

^1^Beth Israel Deaconess Medical Center, BOSTON, MA; ^2^Beth Israel Deaconess Medical Center, Boston, MA; ^3^Beth Israel Deaconess Medical Center, Newton, MA

**Objective**: Prior studies have found mixed results on the impact of IV fluid administration and labor outcomes. Following Hurricane Helene in September 2024, there was a nationwide IV fluid shortage due to flooding of a main production facility. As a result, our labor & delivery unit modified fluid protocols to use normal saline instead of lactated ringers and administer fluids at 50 cc/hr instead of 125 cc/hr. This provides a natural experiment to assess the impact of IV fluid use on labor outcomes.

**Study Design**: The fluid protocol was changed on October 16, 2024. This is a retrospective analysis examining fluid utilization and labor outcomes before (7/1/24–10/16/24) and after (10/17/24–1/31/25) the change in fluid protocol. All deliveries in our unit that labored (i.e. excluding scheduled cesareans) were included. Statistics were conducted in Stata.

**Results**: There was a significant decrease in fluid usage in the 3 months after the fluid shortage compared to the 3 months before (5.2 bags of 500 ml of fluid per delivery vs 3.7; *p* < 0.001) (Figure 1). There was no difference in labor duration (16.9 vs 16.3 hours, *p* = 0.92), cesarean delivery (23.0% vs 25.5%, *p* = 0.19), acute kidney injury (AKI) (1.4% vs 1.6%, *p* = 0.66) or postpartum hemorrhage (PPH, quantitative blood loss > 1 liter) (16.0% vs 18.0%, *p* = 0.21) in the before vs. after cohorts respectively (Table 1). There was a decrease in rate of intra‐amniotic infection (IAI) diagnosed after the shortage (10.8 % vs 8.2%, *p* = 0.04) but no difference in rates of NICU admission for term infants (3.7 % vs 4.0%, *p* = 0.52).

**Conclusion**: Despite a large and significant drop in fluid use during the IV fluid shortage, we found no difference in labor duration, cesarean delivery or other labor outcomes. The only significant change was a slight decrease in IAI, which is potentially spurious and unlikely to be related to decreased fluid administration. These results suggest that decreasing fluid utilization does not affect labor outcomes. This may encourage more conservative use of a critical medical resource and an opportunity for cost savings for labor & delivery units.

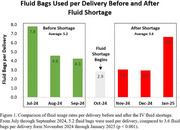

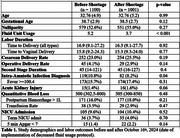

**10:30 AM – 12:00 PM**

## 772 | Geographic **and Racial Disparities in Placenta Accreta Spectrum: Does Referral Center Distance Impact Maternal Morbidity?**

Elizabeth Mangone^1^; Kathy Mostajeran^2^; Lelan McCann^2^; Pamela Garcia‐Filion^2^; Christopher Huls^2^

^1^University of Arizona College of Medicine Phoenix, Phoenix, AZ; ^2^University of Arizona College of Medicine, Phoenix, AZ

**Objective**: To assess whether distance to Arizona's sole regional referral center for placenta accreta spectrum (PAS) management is associated with differences in maternal morbidity in a histologically confirmed cohort.

**Study Design**: This single‐center retrospective study included patients with histologically confirmed PAS who delivered at ≥23 weeks of gestation at the only tertiary referral PAS center of excellence in Arizona between 2012 and 2023. Residential proximity was determined by global positioning system based mapping of distance from centroid of home zip code to centroid of hospital zip code: patients were categorized as local (≤50 miles) and remote (>50 miles). The primary outcome was composite maternal morbidity. A univariate analysis measured the association between maternal demographics [race, payor, body mass index, gestational age, and unscheduled delivery] and clinical outcomes by distance. A multivariate logistic regression measured the likelihood of morbidity by distance by adjusting for demographics and insurance status.

**Results**: Of 230 patients with PAS, 49 (21.3%) comprised the remote group. Baseline clinical factors were similar between groups. However, disparities in race and insurance status were observed: remote patients were more likely to identify as Native American (30.6% vs. 5.0%; *p* < 0.001) and be publicly insured (69.4% vs. 54.7%; *p* = 0.02).

Remote residence was more likely for patients identifying as a racial minority (OR 2.48, 95% CI 1.28–4.82) and use of public insurance (OR 2.01, 95% CI 1.01–4.00). Despite these differences, composite maternal morbidity did not differ by distance (adjusted OR 1.07; 95% CI 0.53–2.18).

**Conclusion**: Patients with PAS living remote from our referral center are disproportionately from marginalized populations. Despite this, once transferred to the state's only PAS center of excellence, maternal outcomes were equivalent, highlighting the resilience of regionalized PAS care models. These findings support expansion of coordinated transfer systems, early referral protocols, and prenatal outreach to optimize equitable PAS access and care.

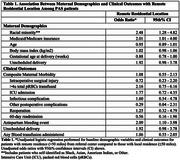

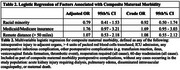

**10:30 AM – 12:00 PM**

## 773 | Association **of Placenta Accreta Spectrum Subtype with Severe Maternal Morbidity**

Elizabeth L. Mirsky; Emily A. DeFranco

University of Kentucky, University of Kentucky/Lexington, KY

**Objective**: Quantify differences in rate and risk of severe maternal morbidity (SMM) by severity of placenta accreta spectrum (PAS) subtype.

**Study Design**: Retrospective cohort study querying the National Inpatient Sample (NIS) database for delivery admissions containing an International Diagnosis Code (ICD) for PAS from 2016 to 2022. The study population was then weighted to approximate the US population according to predefined methods. The likelihood of individual SMM indicators and overall SMM, was stratified by PAS subtype. Risks were calculated for SMM as defined by two criteria – the Center for Disease Control and Prevention (CDC) and standardized PAS criteria (Einerson et al, 2025). Generalized linear modeling estimated relative risks after adjusting for significant confounding risk factors.

**Results**: 6370 (31,850 weighted) patients with PAS were included. 9.5% of accreta, 15.5% of increta, and 21.9% of percreta patients experienced SMM by CDC definition. 8.1% of accreta, 14.3% of increta, and 21.1% of percreta patients experienced SMM by standardized PAS criteria. Most individual SMM indicators were more likely to occur with worsening PAS subtype in a dose‐response type relationship (Table 1). SMM was increasingly more likely with placenta increta and percreta compared to accreta by both CDC and standardized PAS criteria, (CDC aRR [95% CI] 1.5 [1.2–1.9] and 2.1 [1.8–2.5]) and (standard PAS 1.5 [1.2–1.9] and 2.2 [1.8–2.6]), respectively (Figure 1). Also notable, maternal mortality was over 8‐fold increased with percreta.

**Conclusion**: These findings demonstrate a high rate of SMM associated with PAS, which increases progressively with increta and percreta, from 10% to 20%, respectively. This stepwise rise in morbidity highlights the increasing clinical complexity with deeper placental invasion. These results underscore the importance of meticulous preoperative planning, multidisciplinary coordination, and regionalization of care to optimize maternal outcomes in patients with suspected PAS disorders.

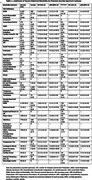

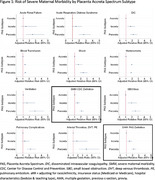

**10:30 AM – 12:00 PM**

## 774 | **The** Association **of Comorbid Gestational Diabetes and Preeclampsia With Adverse Pregnancy Outcomes in American Women**

Elli Y. Tian^1^; Ruth Escalera^1^; Pallavi Dubey^1^; Shakeel Ahmed^2^; Alok K. Dwivedi^2^; Sireesha Reddy^1^

^1^TTUHSC El Paso Paul L Foster School of Medicine, Department of Obstetrics and Gynecology, El Paso, TX; ^2^TTUHSC El Paso Paul L Foster School of Medicine, Department of Molecular and Translational Medicine, El Paso, TX

**Objective**: Gestational diabetes mellitus (GDM) and preeclampsia (PE) are independently associated with adverse pregnancy outcomes, but their combined impact is less understood. This study examines the association between comorbid GDM and PE with adverse pregnancy outcomes.

**Study Design**: We conducted a retrospective analysis using the 2017 National Inpatient Sample, including delivery hospitalizations among women aged 12–53 years. ICD–10 codes identified delivery type (vaginal, cesarean section [CS], instrumental), GDM, PE, and outcomes such as preterm prelabor rupture of membranes (PPROM), amniotic fluid disorders, placental disorders, preterm birth, and postpartum hemorrhage (PPH). Adjusted and propensity score‐matched logistic regression models were used to calculate odds ratios (ORs) with 95% confidence intervals (CIs).

**Results**: Among 592,904 patients, 6.3% had GDM (*n* = 45,016), 9.6% had PE (*n* = 68,740), and 1.3% had both (*n* = 9150). GDM prevalence was highest in Hispanic (7.5%) and Pacific Islander (9.2%) women, while PE was most common in Black women (13.2%). Independently, GDM was associated with increased odds of PPROM (OR  =  1.38; 95% CI = [1.31, 1.46]), amniotic fluid disorders (1.66; [1.58, 1.74]), placental disorders (1.12; [1.06, 1.20]), CS delivery (1.61; [1.58, 1.65]), preterm birth (1.16; [1.10, 1.21]), and PPH (1.06; [1.00, 1.12]). PE was associated with increased odds of CS delivery (1.71; [1.67, 1.74]), preterm birth (1.61; [1.54, 1.69]), and PPH (1.97; [1.89, 2.06]). Comorbid GDM and PE were more strongly associated with certain outcomes, including amniotic fluid disorders (1.92; [1.76, 2.10]), CS delivery (2.81; [2.70, 2.93]), preterm birth (2.20; [2.03, 2.39]), and PPH (1.76; [1.61, 1.94]).

**Conclusion**: Comorbid GDM and PE have a synergistic effect on many adverse pregnancy outcomes. These findings support the need for early identification and enhanced clinical monitoring in pregnancies affected by both conditions.

**10:30 AM – 12:00 PM**

## 775 | **Point‐of‐Care Ultrasound (POCUS) Exposure During Maternal Fetal Medicine (MFM) Fellowship: A National Survey**

Elsa Parra^1^; Sheree D. Carter^2^; Patricia Goedecke^1^; Michael VanDillen^3^; Angela Nakahara^4^; Kerri Brackney^1^

^1^University of Tennessee Health Science Center Memphis, Memphis, TN; ^2^Luminis Health Anne Arundel Medical Center, Upper Marlboro, MD; ^3^University of Tennessee Health Science Center, Memphis, TN; ^4^University of Tennessee Health Science Center, University of Tennessee Health Science Center, TN

**Objective**: Does self‐reported Obstetric Intensive Care Unit (OBICU) exposure impact rates of maternal POCUS (M‐POCUS) use by MFM fellows? We also assessed which educators provide M‐POCUS training, and fellow interest in this training.

**Study Design**: This was a cross‐sectional survey of United States MFM fellows. An anonymous 20‐item web‐based survey was designed to ascertain fellow exposure to and interest in M‐POCUS training. We defined M‐POCUS as maternal cardiac, lung, and/or abdominal ultrasound, and excluded feto‐placental assessment. We directly contacted 382 fellows and 112 program coordinators and directors via email (representing 106 of 107 programs). Surveys were distributed by email twice‐weekly and weekly by text message from March 5 to March 27, 2025.

Proportions were used to determine response rate, M‐POCUS training educator, rate of M‐POCUS use, and interest in M‐POCUS training. A Kruskal‐Wallis test was used to determine if OBICU exposure in fellowship impacts frequency of M‐POCUS use.

**Results**: The response rate was 56.2% (*n* = 215). Only 60% of respondents performed M‐POCUS at least once in fellowship, 53% of whom received M‐POCUS training during MFM fellowship. MFM fellows with OBICU exposure did not report a significant difference in M‐POCUS use (OR 1.63, 95% CI 0.80–3.36). Most M‐POCUS training was directed by an MFM or ICU attending (see graph). Most fellows (85%) would attend an M‐POCUS course, and 95% of respondents believe M‐POCUS would enhance their clinical management.

**Conclusion**: Although OBICU exposure during MFM fellowship did not alter use of M‐POCUS by MFM fellows, there are multidisciplinary educational opportunities available that could help to meet this valued training.

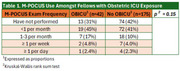

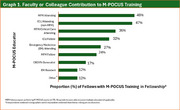

**10:30 AM – 12:00 PM**

## 776 | **Chitty** Abdominal **Circumference Reference Better Predicts Large for Gestational Age Than Hadlock AC Reference**

Emily Heideman^1^; Rebecca Nunge^1^; Angela J. Johnson^1^; Katherine Pressman^2^; Anthony O. Odibo^3^; Jose R. Duncan^1^

^1^University of South Florida, Tampa, FL; ^2^Department of Obstetrics and Gynecology, University of South Florida, Tampa, Florida, Tampa, FL; ^3^University of Missouri Kansas City, Kansas City, MO

**Objective**: To identify the fetal abdominal circumference reference that best predicts large for gestational age (LGA) neonates.

**Study Design**: This was a secondary analysis of a prospective cohort including singleton pregnancies that underwent fetal growth assessment between 26 and 36 weeks’ gestation. Exclusion criteria were fetuses with chromosomal or structural anomalies and cases without delivery data. Fetal biometry measurements were compared to the Chitty abdominal circumference (AC), Hadlock AC, and Hadlock estimated fetal weight (EFW) reference charts. Predictive ability for LGA was assessed by comparing the area under the receiver operating characteristic curve (AUC). Optimal cutoffs were determined using the Youden's index of the AUC coordinates.

**Results**: A total of 1054 neonates were included, of whom 111 (10.5%) were classified as LGA. The Chitty AC reference demonstrated superior predictive performance compared to the Hadlock AC reference (AUC 0.901 [95% CI 0.873–0.929] vs. 0.896 [95% CI 0.868–0.925], *p* = 0.045). The Hadlock EFW reference had an AUC of 0.906 [95% CI 0.878–0.933], which was not statistically different from the Chitty AC (*p* = 0.622) or Hadlock AC (*p* = 0.342) references. The optimal cutoffs were 0.895 for Chitty AC, 0.7450 for Hadlock AC, and 0.6575 for Hadlock EFW.

**Conclusion**: The Chitty AC reference may be a more effective predictor of LGA than the Hadlock AC reference. The Hadlock EFW reference does not significantly improve prediction of LGA compared to AC measurements alone. These findings support consideration of the Chitty AC reference when assessing risk for LGA in clinical practice.

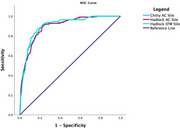

**10:30 AM – 12:00 PM**

## 777 | **The** Impact **of Denying Abortion Access to Individuals Who are Incarcerated: A Cost‐effectiveness Analysis**

Emily W. Modlin^1^; CeAnn Romanaggi^1^; Lily Ben‐Avi^2^; Aaron B. Caughey^2^

^1^Oregon Health & Science University, Portland, OR; ^2^Oregon Health & Science University, Oregon Health & Science University, OR

**Objective**: No mandatory standards exist for provision of reproductive healthcare in United States (US) jails and prisons, historically creating a system for inequitable access to abortion. The June 2022 *Dobbs v. Jackson Women's Health* Supreme Court decision that eliminated constitutional protections for abortion access further exacerbated this inequity in carceral settings. This study investigates the influence of abortion access on outcomes for pregnant individuals who are incarcerated with unintended pregnancies.

**Study Design**: The outcomes and cost‐effectiveness associated with providing abortion services in prisons and jails were evaluated using a decision‐analytic model. Clinical outcomes included mortality, intrauterine fetal demise, pre‐term birth, neonatal death, cesarean delivery, and re‐incarceration. The likelihood and financial burden of caring for an incarcerated pregnant person and the costs of abortion care were considered. The cost‐effectiveness threshold was set at $100,000/QALY and model inputs were derived from the literature.

**Results**: Our theoretical cohort included 6373 pregnant individuals who are incarcerated with unintended pregnancies. Access to abortion services led to 507 fewer preterm births, 2111 fewer cesarean sections, 35 fewer neonatal deaths, and 10 fewer episodes of re‐incarceration (Table 1). Access to abortion while incarcerated within jails and prisons resulted in a cost reduction of $136,129,893 and an increase in 161 QALYs, a dominant strategy.

**Conclusion**: Providing access to abortion for pregnant individuals who are incarcerated is a cost‐effective strategy, reducing adverse maternal and fetal outcomes and improving quality of life. Importantly, access to abortion also reduces re‐incarceration, an outcome that incurs significant individual and societal economic burden. With no mandatory standards for abortion care within US carceral settings and in the context of restrictive abortion laws, these findings may inform policies that improve outcomes for this vulnerable population.

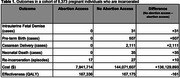

**10:30 AM – 12:00 PM**

## 778 | Interpregnancy **Interval and Weight Trajectories: Implication for Maternal Obesity**

Emma Altieri^1^; Julia Burd^2^; Candice L. Woolfolk^3^; Katherine H. Bligard^1^; Jeannie C. Kelly^3^; Nandini Raghuraman^4^; Alison G. G. Cahill^5^; Antonina I. Frolova^4^

^1^Washington University School of Medicine, Saint Louis, MO; ^2^Perelman School of Medicine, University of Pennsylvania, St. Louis, MO; ^3^Washington University School of Medicine, Washington University School of Medicine, MO; ^4^Washington University School of Medicine, St. Louis, MO; ^5^Department of Women's Health, Dell Medical School at the University of Texas at Austin, Department of Women's Health, Dell Medical School at the University of Texas at Austin, TX

**Objective**: Obesity is the most prevalent medical condition among reproductive‐age women and is associated with increased risk of adverse maternal and fetal outcomes. This study aimed to evaluate the relationship between interpregnancy interval (IPI) and maternal weight change between consecutive pregnancies.

**Study Design**: We conducted a secondary analysis of patients with one prior vaginal delivery and a subsequent singleton gestation delivered at ≥37 weeks between 2004 and 2015. The primary outcome was maternal delivery weight change between the first (G1) and second (G2) pregnancies, categorized by IPI < 2 years, 2–4 years, and >4 years. Secondary outcomes included change in BMI and incidence of new obesity (BMI ≥ 30 kg/m^2^) in G2. One‐way ANOVA and chi‐square tests were used for univariate analyses and linear regression was used to adjust for potential confounders.

**Results**: Of the 1435 patients, 478 (33%) had an IPI of < 2 years, 627 (44%) had an IPI of 2–4 years, and 330 (23%) had an IPI of >4 years. Maternal age and race were significantly different between groups, while the prevalence of chronic hypertension and pregestational diabetes was not. The mean delivery BMI in G1 pregnancies was approximately 30 kg/m2 and not significantly different between the groups (*p* = 0.38). Weight change between deliveries was –0.66 (SD = 8.3), 0.41 (9.4) and 4.01 (11.3) kg for IPI of <2 years, 2–4 years, and >4 years, respectively (*p* < 0.001). Linear regression showed that for every one year in IPI there was a 1.06 kg (95% CI 0.72–1.39) weight gain. Rates of new obesity in G2 differed significantly across groups, occurring in 12.1%, 16.2%, and 25.7% of women with IPI < 2, 2–4, and > 4 years, respectively.

**Conclusion**: There is a significant relationship between the length of IPI and maternal weight change between consecutive pregnancies. Longer IPI is associated with both notable weight gain and the development of obesity. Since IPI is a modifiable risk factor, patient education may reduce the risk of adverse pregnancy outcomes associated with maternal obesity in subsequent pregnancies.

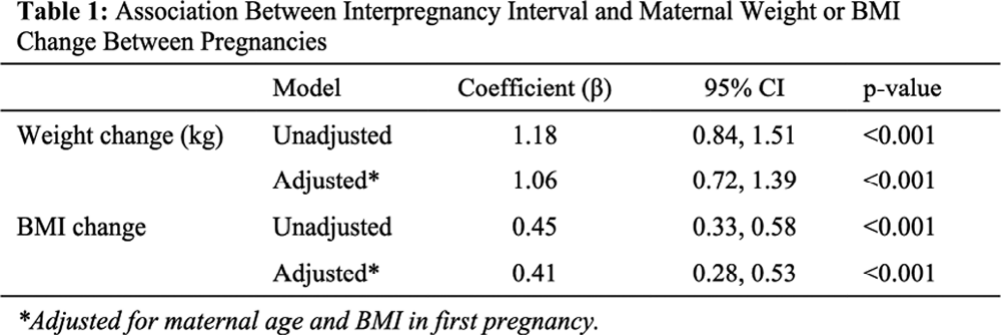

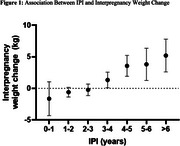

**10:30 AM – 12:00 PM**

## 779 | **Risk of** Recurrent **Severe Hypertensive Disorders of Pregnancy**

Emma Trawick Roberts^1^; Lauren Kucirka^2^; Matt Fuller^3^; Maura Jones Pullins^4^; Kim Boggess^5^; Marie‐Louise Meng^3^; Johanna Quist‐Nelson^1^

^1^University of North Carolina, Chapel Hill, Chapel Hill, NC; ^2^UNC Chapel Hill, Durham, NC; ^3^Duke University School of Medicine, Durham, NC; ^4^University of North Carolina, Chapel Hill, Durham, NC; ^5^University of North Carolina at Chapel Hill, Chapel Hill, NC

**Objective**: Prior studies have reported different recurrence rates for severe hypertensive disorders of pregnancy (HDP). We sought to assess the recurrence risk of severe HDP (sHDP) in a nationwide cohort.

**Study Design**: Retrospective cohort study using the Premier Inpatient database. We identified 560,295 individuals with ≥2 observed pregnancies between 10/2015 and 12/2020. The risk and relative risk (RR) of sHDP, defined as preeclampsia with severe features, HELLP syndrome, or eclampsia, in a subsequent pregnancy, was estimated based on HDP in index pregnancy. The risk of sHDP by gestational age and the risk of each subtype of sHDP (any severe features, HELLP, or eclampsia) was also estimated.

**Results**: Of those with ≥2 pregnancies, mean age was 27.2 ± 5.3 years, 14.1% were Black, and 3.3% had class III obesity. 57,584 (10.3%) had HDP and 11,433 individuals (2.0%) had sHDP in the index pregnancy; 1703 (14.9%) experienced recurrent sHDP in a subsequent pregnancy. Tables 1 and 2 show the risk of subsequent sHDP by timing and severity of HDP in the index pregnancy. Compared to those with no HDP in the index pregnancy, those with sHDP at any gestational age had an increased risk of sHDP in a subsequent pregnancy (14.9%, RR 16.0, 95% CI 15.2–17.0); those with a history of sHDP <28 weeks’ gestation had the highest risk (22.9%, RR 23.1, 95% CI 19.1–27.9). sHDP < 28 weeks conferred the highest risk of recurrence at < 28 weeks (RR 184.9, 95% CI 107.2–318.9), but the absolute risk was low (4.71%). Those with a history of HELLP or eclampsia had elevated risk of sHDP (11.5%, RR 9.7, 95% CI 8.3–11.3 and 11.0%, RR 9.2 95% CI 7.2–11.75 respectively), including recurrence of HELLP (4.6%, RR 61.0 95% CI 46.2–80.6) and eclampsia (1.1%, RR 40.1 95% CI 16.3–98.3), but these phenotypes occurred infrequently.

**Conclusion**: The risk of recurrent sHDP is lower than previously reported and is influenced by the severity and timing of HDP in the index pregnancy, with the highest recurrence seen among those with early onset sHDP. These results inform counseling about risk of future sHDP to aid patients in reproductive decision‐making.

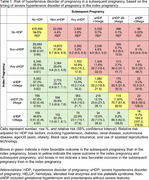

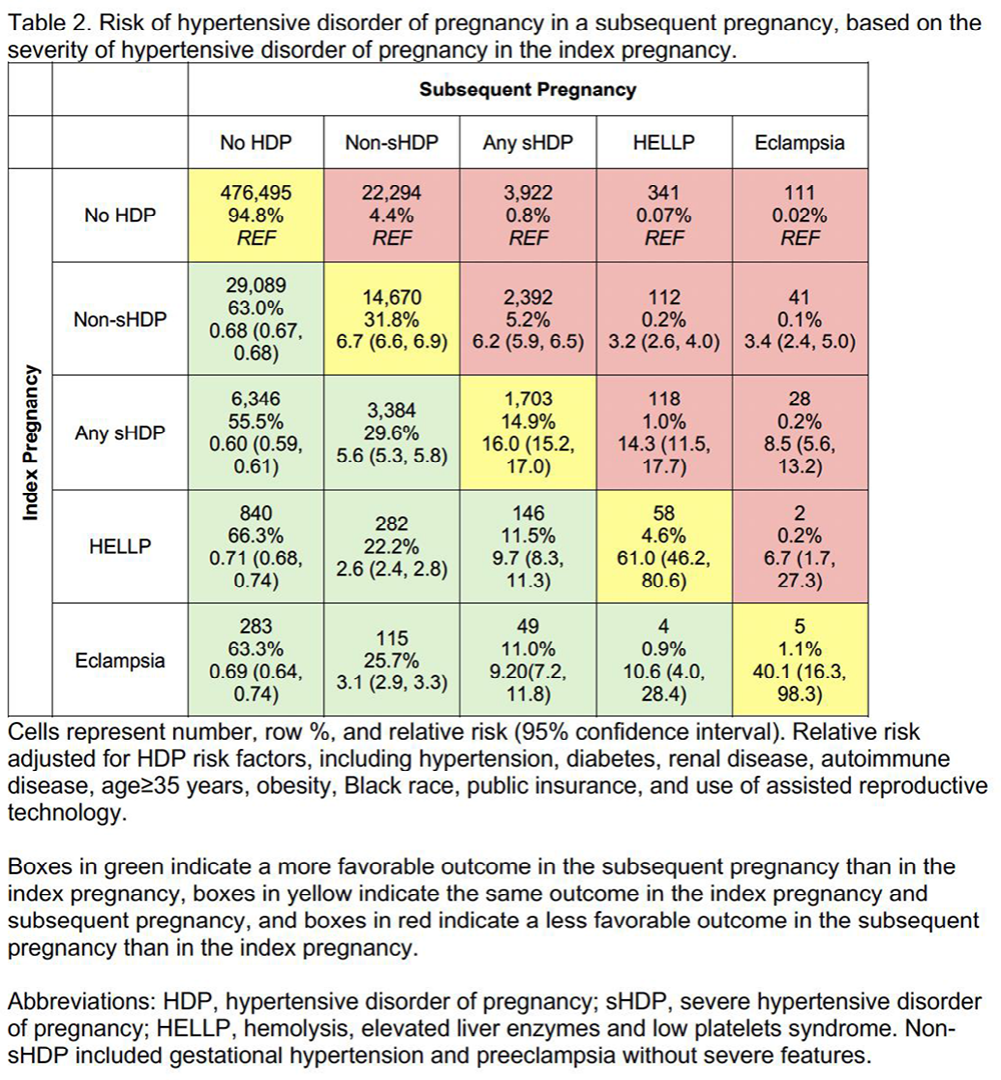

**10:30 AM – 12:00 PM**

## 780 | **Maternal Gestational Diabetes and the Risk of Polycystic Ovary and Metabolic Syndromes in Their Daughters**

Enav Yefet^1^; Manal Massalha^2^; Malak Watad^2^; Faten Yichye^2^; Zohar Nachum^2^

^1^Tzafon medical center, Poriya, HaZafon; ^2^Emek medical center, Afula, HaZafon

**Objective**: Gestational diabetes mellitus (GDM) is a known risk factor for adverse short‐ and long‐term outcomes in offspring, yet its independent contribution to polycystic ovary syndrome (PCOS) and metabolic syndrome remains uncertain. We aimed to examine whether intrauterine exposure to GDM is associated with increased risk of PCOS and metabolic syndrome in female offspring, independent of shared genetic and environmental factors.

**Study Design**: In this sibling‐controlled cohort study conducted between 2018 and 2022, we enrolled 28 pairs of sisters born to the same parents and raised in the same household; one following a GDM pregnancy and one following a non‐GDM pregnancy. Participants underwent clinical evaluation, endocrine and biochemical testing, and pelvic ultrasound. The primary outcomes were the rates of PCOS according to Rotterdam criteria; and metabolic syndrome using age‐specific National Cholesterol Education Program, Adult Treatment Panel III and International Diabetes Federation guidelines. The assessor was blinded to GDM status. Comparison between siblings was done using McNemar test for categorical variables and paired T test or Wilcoxon Signed Rank test of continuous variables.

**Results**: Sisters from GDM pregnancies exhibited significantly higher rates of PCOS, hirsutism, and elevated androgen levels (total testosterone and androstenedione) compared to their non‐GDM siblings. Although the overall prevalence of metabolic syndrome did not differ significantly, low HDL cholesterol was more common in the GDM‐exposed group. No significant associations were found between glycemic control during GDM pregnancy and the study outcomes.

**Conclusion**: In utero exposure to GDM is associated with long‐term metabolic and hormonal disturbances, particularly PCOS and low HDL cholesterol, in female offspring independent of genetic and shared environmental factors. These findings underscore the importance of intrauterine programming in the pathogenesis of PCOS and highlight a potential intergenerational impact of GDM.

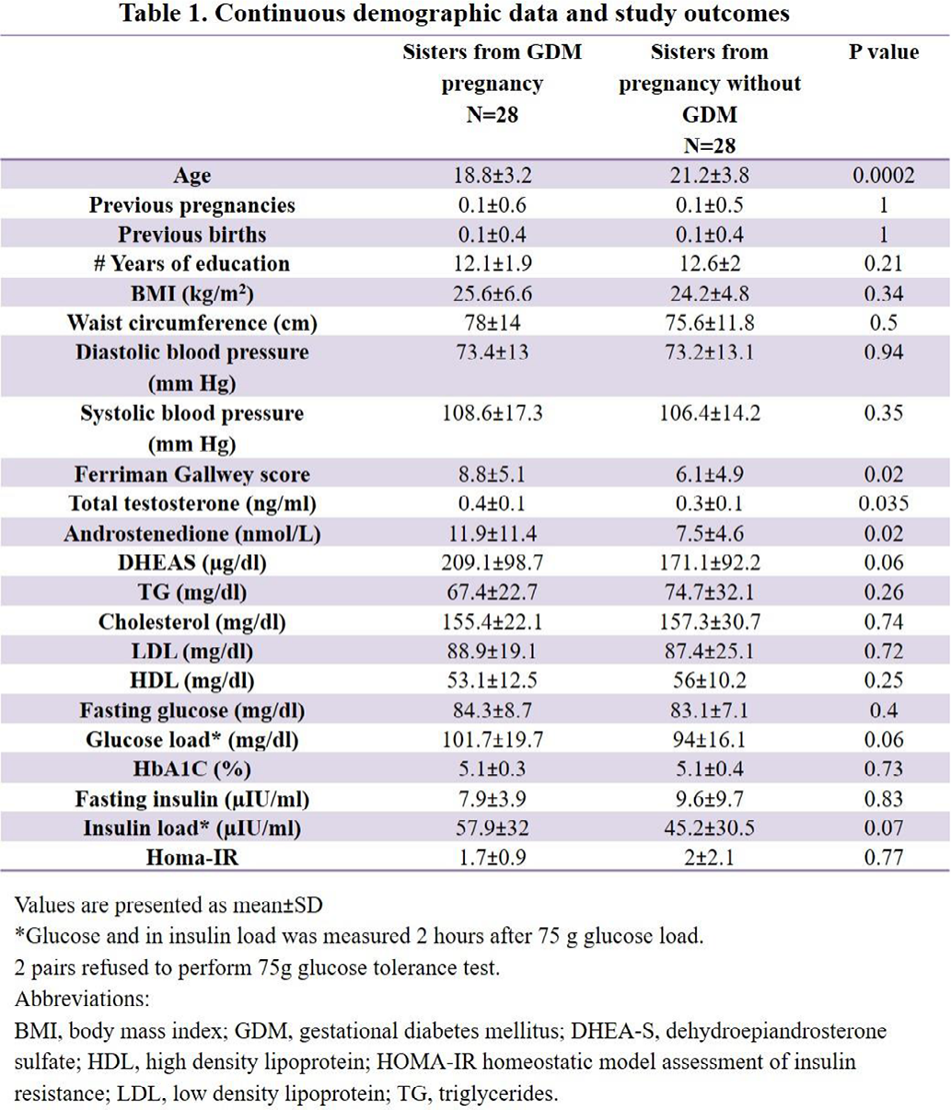

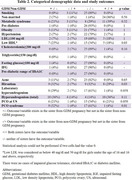

**10:30 AM – 12:00 PM**

## 781 | **A** Translational **Process Barrier: Recruitment and Retention of Black Birthing Women in Research**

Erica Marion^1^; Alenna Beroza^2^; Linetta Alexander Islam^3^; Rashonda Jones^4^; Michael Anello^2^; Anna Palatnik^5^

^1^Medical College of Wisconsin, Milwaukee, WI; ^2^Medical College of Wisconsin, Medical College of Wisconsin, WI; ^3^Ubuntu Research & Evaluation, Ubuntu Research & Evaluation, WI; ^4^The Parenting Network, The Parenting Network, WI; ^5^Medical College of Wisconsin – Milwaukee, WI, Milwaukee, WI

**Objective**: Black pregnant women are less likely to participate in research although they have the highest prevalence of adverse outcomes. Low recruitment and retention of Black women in research is a barrier for study generalizability and innovative interventions that could benefit the population at risk. The purpose of this study was to identify barriers of recruitment and retention of Black women in obstetric settings.

**Study Design**: Semi‐structured focus groups were conducted with Black women and their family members at two local community organizations in Milwaukee, Wisconsin. Audio was recorded, transcribed verbatim and analyzed using content thematic analysis. Focus group content included knowledge and attitudes towards research, research approaches, previous experiences with clinical research, clinical research interventions involving medication, research mistrust, barriers and facilitators to participating in clinical settings, and research education.

**Results**: There was a total of 32 Black women and 2 Black men participated in three focus group. To analyze barriers in retention and recruitment we identified four themes: 1) Some individuals perceive research methodologies as being inefficient and burdensome, leading to falsification of self‐reported data to expedite research activities. 2) Previous traumatic birth experiences and perceived side effects of medications. 3) Limited opportunities to participate in group settings to feel supported and “heard” instead of individual study participation. 4) Lack of dissemination to communities perceived as participation being insignificant and invaluable.

**Conclusion**: We identified key barriers to recruiting and retaining Black women in obstetric research, including inefficiencies, past negative experiences, limited group participation, and poor community dissemination. Addressing these issues is crucial to building trust, enhancing participation, and ensuring research benefits this high‐risk population.

**10:30 AM – 12:00 PM**

## 782 | Smartphone **Applications to Support Perinatal Mental Health: A Systematic Review**

Erin Chang^1^; Emily S. Miller^2^; Adam K. Lewkowitz^2^; Jennifer Unger^2^; Craig F. Garfield^3^; Jacqueline Gollan^3^; Young S. Lee^4^; Kathleen O'Sullivan^3^; Dinah Williams^5^

^1^The Warren Alpert Medical School of Brown University, Providence, RI; ^2^Women & Infants Hospital of Rhode Island / Warren Alpert Medical School of Brown University, Providence, RI; ^3^Northwestern University Feinberg School of Medicine, Evanston, IL; ^4^Northwestern University Feinberg School of Medicine, Chicago, IL; ^5^Women & Infants Hospital of Rhode Island, Providence, RI

**Objective**: Perinatal mental health conditions are common, yet many face barriers to accessing timely care. Smartphone apps can expand access to mental health support during the perinatal period and fill extant gaps in clinical care provision. However, many apps marketed directly to patients lack evidence of clinical effectiveness. Our objective was to identify which publicly available apps have been evaluated in clinical trials or observational studies to demonstrate efficacy in improving perinatal mental health outcomes.

**Study Design**: We conducted a systematic review of perinatal mental health smartphone apps available on the Apple App and Google Play Stores. Search terms included: “maternal mental health”, “perinatal mental health”, “postpartum health”, “pregnant mental health”, and “parent mental health”. Apps were included if they: 1) focused on perinatal individuals; 2) aimed to improve perinatal mental health; 3) were available in the US. For each app, we extracted key features and searched PubMed and ClinicalTrials.gov to identify published studies designed to evaluate efficacy. Publications were classified using the USPSTF grading system.

**Results**: Of 587 apps identified, 38 met the predefined inclusion criteria (Figure). Among these 38, only three (8%) had associated peer‐reviewed publications. One app had low‐level evidence, corresponding to a USPSTF Grade B recommendation; two were classified as USPSTF Grade I, indicating insufficient evidence. Six apps (16%) had ongoing RCTs registered with ClinicalTrials.gov. A summary of the apps’ corresponding USPSTF grades is provided in the Table.

**Conclusion**: Despite the proliferation of smartphone apps marketed directly to patients for perinatal mental health support, few have been rigorously evaluated. This lack of evidence raises concerns about efficacy, safety, accountability, and value‐based care. To ensure safe and effective mental health care delivery, efforts must prioritize the development of evidence‐based digital perinatal mental health interventions and apply greater caution in marketing unproven tools directly to patients.

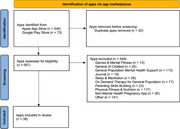

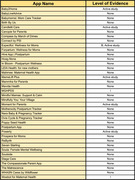

**10:30 AM – 12:00 PM**

## 783 | **Early Oral Feeding After Cesarean Delivery: A Systematic Review and Meta‐Analysis**

Fabrizio Zullo^1^; Moti Gulersen^2^; Sahithi Talasila^3^; Gabriele Saccone^4^; Antonio F. Saad^5^; Vincenzo Berghella^6^

^1^University of Rome La Sapienza, Rome, Lazio; ^2^Division of Maternal Fetal Medicine, Department of Obstetrics, Gynecology and Reproductive Science, Jefferson Health, Philadelphia, PA, Philadelphia, PA; ^3^Jefferson University, Philadelphia, PA; ^4^Università degli Studi di Napoli Federico II, Napoli, Campania; ^5^Inova Fairfax Hospital, Falls Church, VA; ^6^Thomas Jefferson University, Philadelphia, PA

**Objective**: To evaluate the effect of early oral feeding (EOF) after cesarean delivery (CD) on maternal and perinatal outcomes

**Study Design**: The search was conducted by a medical librarian using PubMed, Google Scholar, OVID, and Cochrane Library as electronic databases from their inception until May 2025, using specific keywords. Selection criteria included RCTs of patients with CD comparing EOF (intervention group) to delayed oral feeding (control group). EOF was defined as a liquid and/or solid diet < 24 hours of CD. Delayed oral feeding was defined as feeding > 24 hours or as defined by the original trial.

Analyses were done using an intention‐to‐treat approach. The primary outcome was hospital length of stay (LOS). Secondary outcomes included mean time intervals to first flatus and bowel sounds and presence of nausea, vomiting, and ileus symptoms. A subgroup analysis for EOF ≤ 6 hours was performed. The summary measures were reported as relative risk (RR) or mean difference (MD) with a 95% CI using the fixed‐effects model of DerSimonian and Laird. I‐squared (Higgins I2) was used to identify heterogeneity. (CRD42022314823)

**Results**: Data from 24 RCTs and 5760 participants were included (2884 vs 2876). Patients in the EOF group (usually < 12 hours after CD) had a significantly shorter postoperative LOS (MD −0.21 days, 95% CI −0.23 to −0.19), and shorter time interval to first flatus (MD −5.38 hours, 95% CI −5.73 to − 5.03), and first bowel sounds (MD −5.07 hours, 95% CI −5.38 to − 4.76) compared to controls. The rates of nausea, vomiting, and ileus symptoms were similar between the two groups. (Table 1). In the subgroup analysis, EOF <6 hours after CD was also associated with significantly shorter LOS, shorter time interval to first flatus, and to first bowel sounds, compared to delayed oral feeding

**Conclusion**: Early oral feeding – as early as <6 hours – after CD is associated with a reduced LOS and time interval to first flatus and bowel sounds compared to controls, without increasing GI symptoms. These findings support the safety and benefit of early postoperative feeding ≤6 hours in this population.

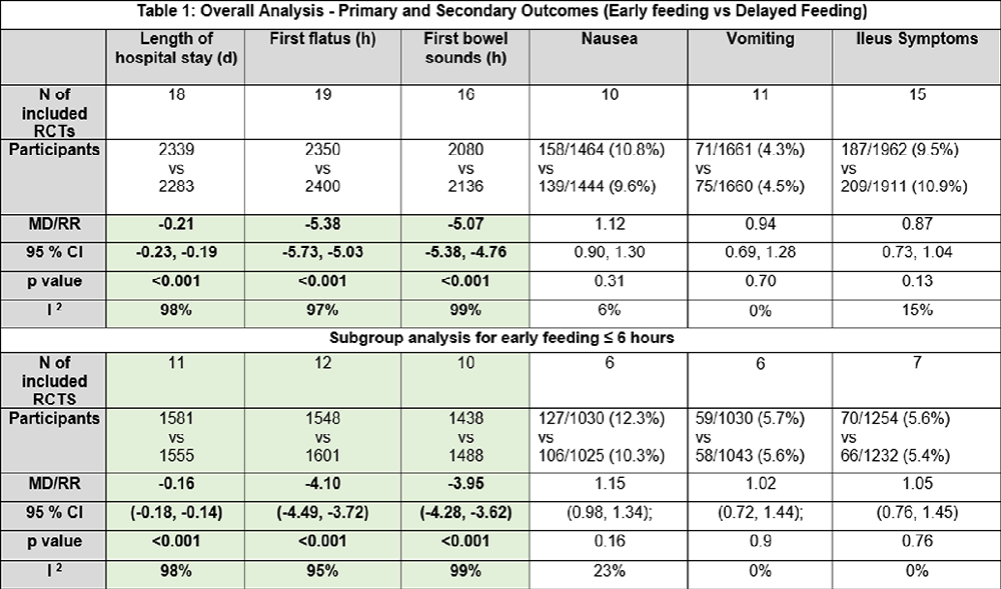

**10:30 AM – 12:00 PM**

## 784 | Sonographer **Workload Benchmarks Across Maternal Fetal Medicine Practice Settings in the United States**

Fadi Bsat^1^; Suwan Mehra^2^

^1^SMFM Practice Management Committee, West Springfield, MA; ^2^Advocate Children's Hospital, Advocate Childrens Hospital, IL

**Objective**: To establish national benchmarks for sonographer workload by quantifying the average number of ultrasound examinations performed per sonographer during an 8‐hour workday across various Maternal Fetal Medicine (MFM) practice settings in the United States.

**Study Design**: A cross‐sectional, web‐based benchmarking survey was emailed to all 2096 regular U.S.‐based members of the Society for Maternal Fetal Medicine (SMFM). Of these, 232 unique responses were included in the final analysis. Respondents were categorized by practice ownership—university/medical school, health system, hospital with academic affiliation, community hospital, and private practice—and by practice size, defined by the number of clinical full‐time equivalent (cFTE) MFM physicians (large: ≥ 5 cFTE; small: < 5 cFTE). Pearson's Chi‐squared test with simulated *p*‐value was used for statistical analysis.

**Results**: The median number of ultrasound examinations performed per sonographer during an 8‐hour workday was consistent across most practice settings, with a median of 8–9 scans per day. Community hospitals (*n* = 14) demonstrated a higher median daily volume of 10–11 scans, though this trend did not reach statistical significance (*p* = 0.053). When stratified by practice size, no significant difference was observed in sonographer workload between large and small practices, with both groups reporting a median of 10–11 scans per day (*p* = 0.379).

**Conclusion**: Sonographer workload in MFM practices demonstrates overall consistency across diverse practice types and sizes, with a typical volume of 8–9 ultrasound examinations per 8‐hour shift. The number of MFM physicians in a given practice did not significantly impact sonographer workload.

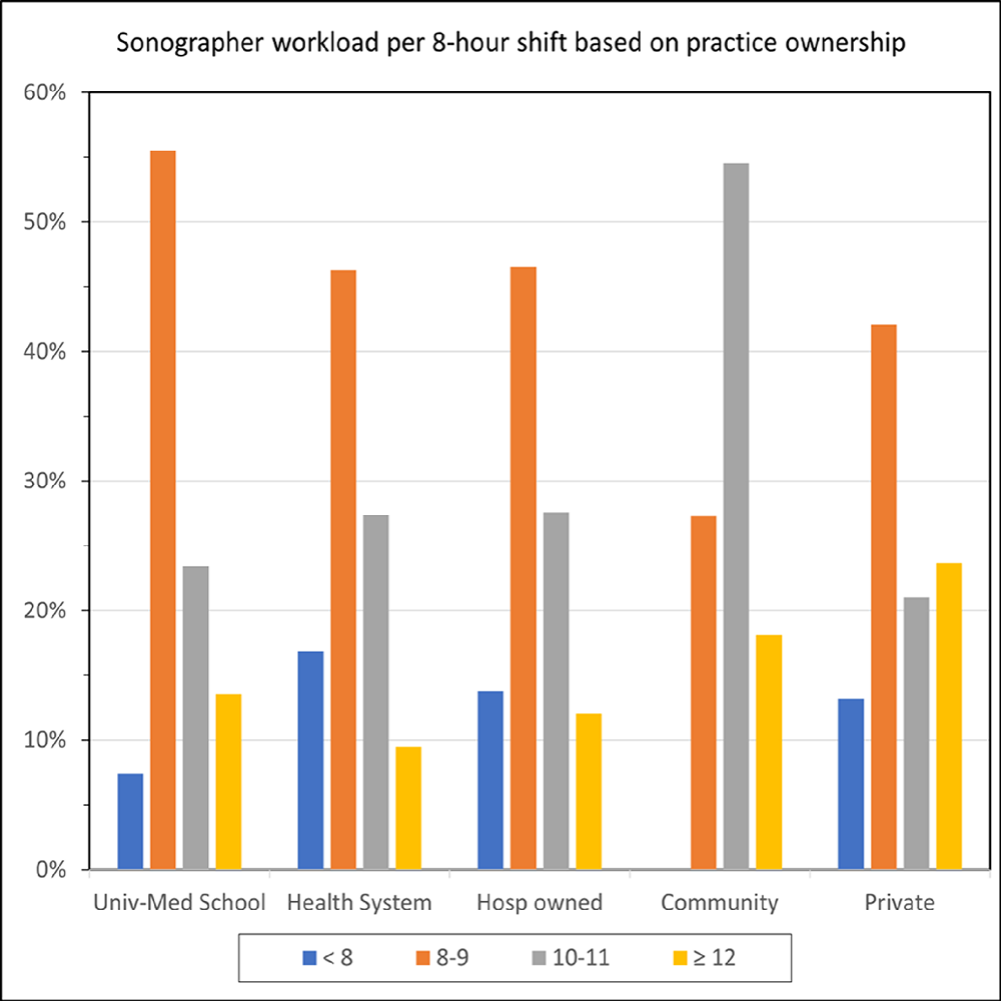

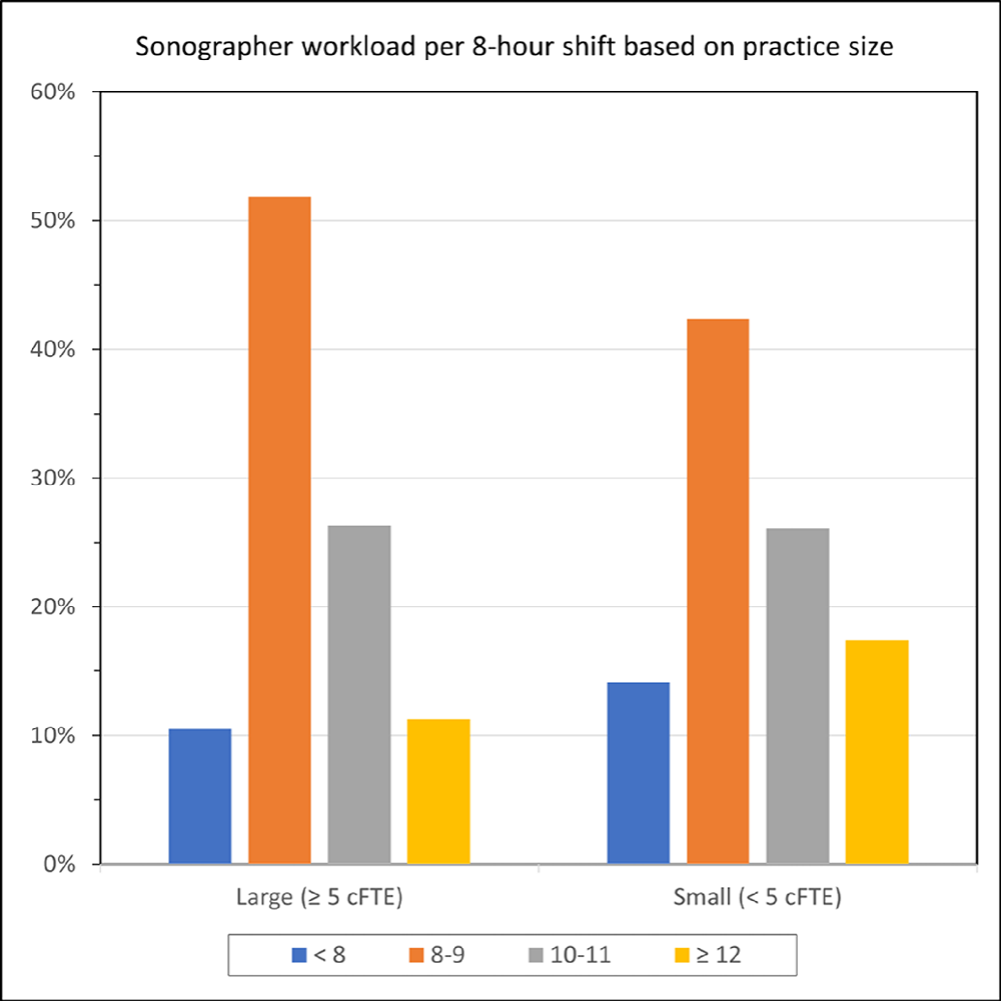

**10:30 AM – 12:00 PM**

## 785 | Association **Between HELLP Syndrome and Peripartum Cardiomyopathy**

Fay Pon^1^; Ariane C. Youssefzadeh^2^; Zaira Chavez Jimenez^1^; Yadira L. Bribiesca Leon^1^; Jennifer Yao^3^; Joseph G. Ouzounian^2^; Koji Matsuo^4^

^1^Keck School of Medicine of USC, University of Southern California, Los Angeles, CA; ^2^Division of Maternal‐Fetal Medicine, Department of Obstetrics and Gynecology, University of Southern California, Los Angeles, CA; ^3^University of Southern California, Keck School of Medicine, Los Angeles, CA; ^4^Division of Gynecologic Oncology, Department of Obstetrics and Gynecology, University of Southern California, Los Angeles, CA

**Objective**: To assess the association between HELLP syndrome and peripartum cardiomyopathy.

**Study Design**: This cross‐sectional study queried the Healthcare Cost and Utilization Project's National Inpatient Sample. The study population included 24,976,626 hospital deliveries from 2016–2022 and the exposure was a diagnosis of HELLP syndrome (*n* = 68,840 for HELLP and *n* = 24,907,786 for no HELLP syndrome). The main outcome measure was a diagnosis of peripartum cardiomyopathy. A multivariable generalized linear model was used to assess the exposure‐outcome association, adjusted for historical clinico‐obstetric characteristics.

**Results**: The incidence of peripartum cardiomyopathy (PPCM) was 24.0 and 2.2 per 10,000 deliveries for the HELLP syndrome and non‐HELLP syndrome group, respectively (*p* < 0.001). After controlling for maternal age, race and ethnicity, medical comorbidities (hypertensive disorder of pregnancy, diabetes mellitus, and obesity), socio‐economic status (primary payer and household income), substance use disorders, prior uterine scar, gestational age at delivery, cesarean delivery, and hospital parameters (location, teaching status, and region), pregnant people with HELLP were nearly three times more likely to have a diagnosis of PPCM compared to those without HELLP (adjusted‐incidence rate ratio [aIR] 1.26, 95% confidence interval [CI] 1.07–1.47). This association was larger for younger maternal age: aIR for < 25, 25–29, 30–34, and ≥35 years, 2.00 (95% CI 1.48–2.70), 1.65 (95% CI 1.24–2.18), 0.80 (95% CI 0.55–1.15), and 0.90 (95% CI 0.63–1.30), respectively. The association was also larger for delivery at later gestational age: aIR for ≥37, 34–36, 28–33, and <28 weeks gestation, 2.67 (95% CI 1.94–3.66), 1.30 (95% CI 0.93–1.83), 1.05 (95% CI 0.79–1.41), and 1.15 (95% CI 0.72–1.85), respectively.

**Conclusion**: This cross‐sectional study suggests that although rare, pregnant people with HELLP may have an increased risk of PPCM, particularly if delivery was at term. The role of PPCM as a complication of HELLP syndrome is clinically compelling and warrants further investigation in a prospective sample of patients.

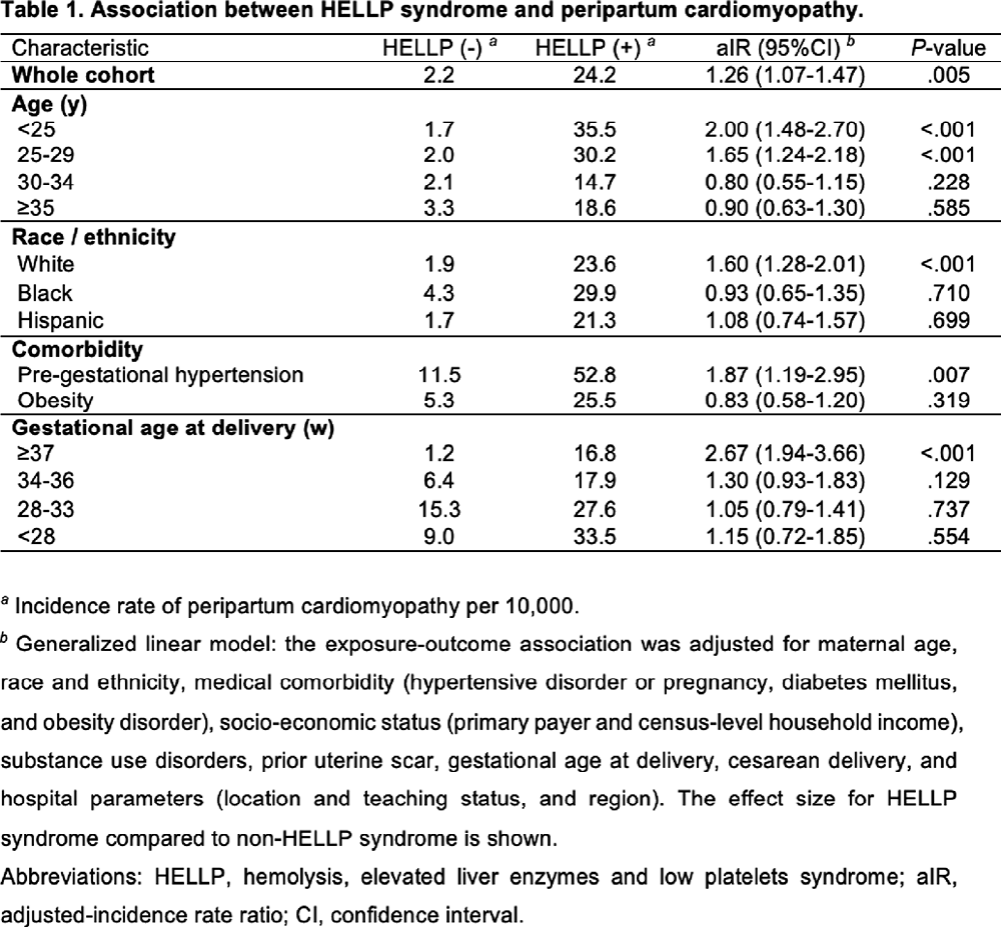

**10:30 AM – 12:00 PM**

## 786 | **Risk** Factors **for Preterm Prelabor Rupture of Membranes After Fetoscopic Spina Bifida Repair**

Felicia V. LeMoine^1^; Neha Agarwal^2^; Sen Zhu^3^; Sami Backley^1^; Elisa Garcia^3^; Stephanie Conaway^3^; Ashley Salazar^3^; Lovepreet Mann^3^; Stephen A. Fletcher^4^; Kuojen Tsao^5^; Mary Austin^5^; Eric Bergh^6^; Jimmy Espinoza^3^; Anthony Johnson^3^; Jerrie S. Refuerzo^1^; Suzanne Lopez^7^; David I. Sandberg^4^; Dejian Lai^8^; Ramesha Papanna^9^

^1^Division of Fetal Intervention, Department of Obstetrics, Gynecology and Reproductive Sciences, McGovern Medical School at UTHealth Houston, Houston, TX; ^2^University of Texas Health Science Center at Houston, UT Health Houston, TX; ^3^UTHealth Houston Fetal Institute; Division of Fetal Intervention, Department of Obstetrics, Gynecology and Reproductive Sciences, McGovern Medical School at UTHealth Houston, Houston, TX; ^4^Division of Pediatric Neurosurgery, Department of Pediatric Surgery, McGovern Medical School at UTHealth Houston, Houston, TX; ^5^Division of Pediatric General & Thoracic Surgery, Department of Pediatric Surgery, McGovern Medical School at UTHealth Houston, Houston, TX; ^6^Maternal Fetal Medicine, Nemours Children's Health, Wilmington, DE; ^7^Division of Neonatal‐Perinatal Medicine, Department of Pediatrics, McGovern Medical School at UTHealth Houston, Houston, TX; ^8^Department of Biostatistics, School of Public Health at UTHealth Houston, Houston, TX; ^9^University of Texas Health Science Center in Houston, McGovern Medical School, Houston, TX

**Objective**: Preterm prelabor rupture of membranes (PPROM) is a persistent complication of in‐utero spina bifida repair despite the transition to a minimally‐invasive, fetoscopic approach. We evaluated the relationship between maternal and perioperative characteristics and PPROM among pregnancies that underwent fetoscopic spina bifida repair.

**Study Design**: A secondary analysis of a prospective cohort study was conducted on cases of fetoscopic spina bifida repair completed between 2020 and 2025 (NCT#04243889 & #06042140). Maternal and perioperative factors were compared between cases with and without PPROM using appropriate two‐sample t tests, χ^2^ test, and Fisher's exact test, where applicable. Multiple logistic regression was performed to identify maternal and perioperative factors associated with PPROM.

**Results**: Twenty‐nine of 99 (29.3%) cases resulted in PPROM. The mean gestational age (GA) at PPROM was 32.4 ± 2.5 weeks. The PPROM group had earlier GA at delivery (33.4 ± 1.9 versus 35.7 ± 2.7 weeks, *p* < 0.001) and lower rates of gestational hypertension (0% versus 21.4%, *p* < 0.001) than the group without PPROM. Maternal and perioperative characteristics and obstetric outcomes were otherwise similar between groups. Multiple logistic regression demonstrated no significant relationship between maternal nor perioperative characteristics and PPROM.

**Conclusion**: No single clinical factor offers significant predictive value for PPROM. Further research is warranted to identify underlying biochemical mechanisms that may influence the risk of PPROM after fetoscopic intervention.

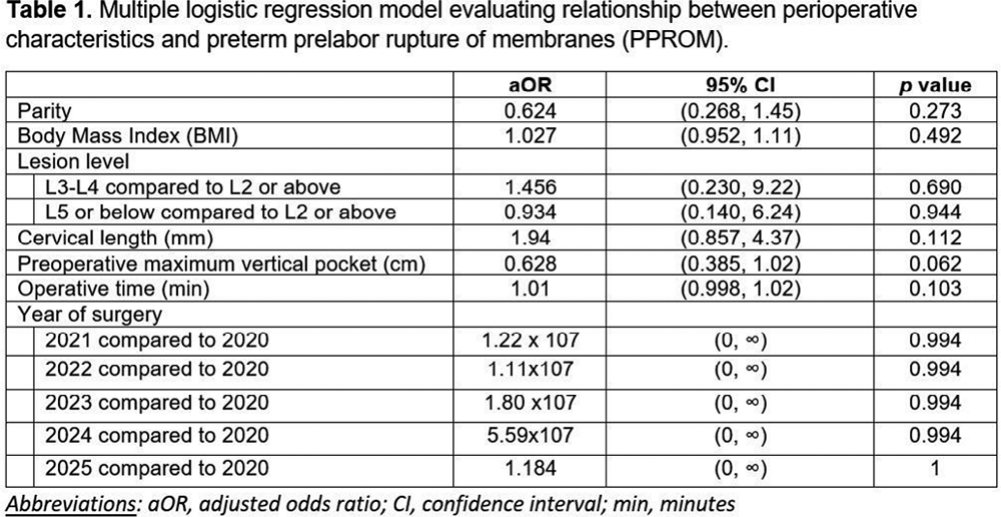

**10:30 AM – 12:00 PM**

## 787 | **Impact of Maternity Care Access on Term Delivery Management Following Prior Demise**

Frank I. Jackson; Isabella A. Molina; Sarah H. Abelman; Oladunni Ogundipe; Luis A. Bracero; Matthew J. Blitz

Northwell, New Hyde Park, NY

**Objective**: To examine whether living in a maternity care desert affects delivery timing and clinical decision‐making among term pregnancies complicated by prior fetal or neonatal death.

**Study Design**: Retrospective cohort study using 2023 U.S. natality data. We identified 40,109 term births among individuals with a prior child or fetal loss and linked their county of residence to the March of Dimes Maternity Care Access Codes. Multivariable logistic regression adjusted for race, Hispanic ethnicity, public insurance, and advanced maternal age. Outcomes included early term delivery (<39 weeks), induction of labor(IOL), betamethasone receipt, cesarean delivery (CD), and early term iatrogenic delivery (CD, and IOL).

**Results**: Compared to those in full‐access counties, individuals in maternity care deserts were more likely to have early term deliveries (40.8% vs 39.5% aOR 1.13; 95% CI: 1.02–1.25; *p* = 0.022). They were not significantly more likely to undergo IOL (28.5% vs 31.8%, *p* = 0.053), receive betamethasone (1.3% vs 2.0%, *p* = 0.668), or CD (35.8% vs 38.8%, *p* = 0.246). Among early term births, iatrogenic delivery rates were similar (64.4% vs 66.0% *p* = 0.46), suggesting non‐iatrogenic drivers. Black individuals were more likely to deliver early term than white individuals (44.5% vs 37.7% *p* < 0.001).

**Conclusion**: Despite fewer resources, patients in maternity care deserts received similar rates of obstetric interventions. However, they were more likely to deliver early without corresponding increases in iatrogenic birth, suggesting other contributors. Racial disparities persisted regardless of access level, highlighting that geographic provider availability alone is insufficient to address inequities. Targeted, equity‐driven interventions are needed to improve outcomes following prior loss.

**10:30 AM – 12:00 PM**

## 788 | **Early** Placental **Morphometric Parameters Predict Preeclampsia With Severe Features Over Clinical Risk Factors**

Gabriel A. Arenas^1^; Alison M. Pouch^2^; Baris Oguz^1^; Rebecca L. Linn^1^; Wensi Wu^1^; Xing Yao^3^; Claudia Tawil^1^; Jingyi Zhu^4^; Brett C. Byram^5^; Ipek Oguz^5^; Nadav Schwartz^1^

^1^University of Pennsylvania, Philadelphia, PA; ^2^Univeristy of Pennsylvania, Philadelphia, PA; ^3^Vanderbilt University, Nashville, TN; ^4^Vanderbilt University, Philadelphia, PA; ^5^Vanderbilt University, Vanderbilt University, TN

**Objective**: Gross placental development may reflect placental health, with novel automated tools now enabling extraction of early morphometric measures from 3D ultrasound. Our primary objective was to identify early placental morphometric differences associated with the development of hypertensive disorders of pregnancy (HDP). The secondary objective was to evaluate whether these parameters, combined with placental volume, improve prediction of HDP.

**Study Design**: This is secondary analysis of a prospective cohort of singleton pregnancies that had 3DUS volumes of the placenta obtained at 11–16 weeks. A validated convolutional neural network generated automated placental segmentations and placental volume measurements. Deformable medial modeling was used to extract standardized 3D morphologic parameters, including fetal and maternal surface areas, thickness, circumference, medial surface diameter, and placental volume. Parameters were normalized by gestational age and compared between normotensive and HDP pregnancies using Student's t‐tests or Mann‐Whitney U tests. Logistic regression and receiver operator curves were used to evaluate the predictive value of clinical risk factors, morphometric parameters, and placental volume for HDP.

**Results**: A total of 74 patients had analyzable placental segmentations that were obtained at an average gestational age of 14.0 ± 1.0 weeks. HDP developed in 19 (26.4%) patients (gestational hypertension (GHTN): 14; preeclampsia with severe features (PEC‐SF): 5). Morphometric parameters did not differ between normotensive and GHTN groups, but patients with PEC‐SF had significantly reduced placental surface areas, circumference, and medial surface diameter (Table 1). Placental thickness was stable across all groups. Placental volume and morphometric parameters outperformed clinical risk factors in predicting PEC‐SF only (Figure 1).

**Conclusion**: Reduced placental surface area, circumference, and mean diameter in early pregnancy may serve as an early indicator of PEC‐SF. These parameters show promise for PEC‐SF prediction but require validation in larger studies.

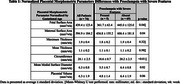

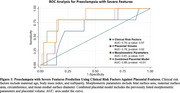

**10:30 AM – 12:00 PM**

## 789 | **The** Association **Between the Anesthesia Technique in Cesarean Delivery and Maternal Infant Bonding**

Aram Azbarga^1^; Tamar Wainstock^2^; Yair Binyamin^3^; Eyal Sheiner^4^; Inbal Reuveni^5^; Gali Pariente^6^

^1^Goldman Medical School at Ben‐Gurion University of the Negev, lehavim, HaDarom; ^2^Department of Epidemiology, Biostatistics and Community Health Sciences, Faculty of Health Sciences, Ben‐Gurion University of the Negev, Beer Sheva, HaDarom; ^3^Department of Anesthesiology, Soroka University Medical Center and the Faculty of Health Sciences, Ben‐Gurion University of the Negev, beer sheva, HaDarom; ^4^Department of Obstetrics and Gynecology, Soroka University Medical Center, Ben Gurion University of the Negev, beer sheva, HaDarom; ^5^Psychiatry Division, Tel Aviv Sourasky Medical Center, Faculty of Medicine, Tel Aviv University, tel aviv, Tel Aviv; ^6^Department of Obstetrics and Gynecology, Soroka University Medical Center, Ben‐Gurion University, beer sheva, HaDarom

**Objective**: Mother‐infant bonding(MIB) is a critical component of early child development and well‐being.Factors such as postpartum depression, anxiety, traumatic birth experiences, and delivery methods can contribute to impaired MIB.This study aimed to assess the association between the type of anesthesia used in cesarian delivery(CD) and MIB, and to evaluate its relationship with post‐partum depression and anxiety.

**Study Design**: A cross‐sectional study was conducted among women 1–3 days postpartum following CD at a single tertiary medical center.Eligible participants completed Postpartum Bonding Questionnaire(PBQ) to assess MIB.We used validated thresholds of a score ≥12 on the Impaired Bonding subscale, indicating impaired MIB.Post‐partum depression was assessed using the Edinburgh Postnatal Depression Scale(EPDS cut‐off ≥13).Anxiety was assessed using the State‐Trait Anxiety Inventory(STAI cut‐off above 75th percentile).Sociodemographic and clinical data were obtained via self‐report and medical records.Multivariable logistic regression model adjusted for confounders.

**Results**: A total of 366 women were included. 96(26.2%) underwent CD under general anesthesia and 270(73.8%) under neuraxial anesthesia. Emergent CD was more common in the general anesthesia group(68.8% vs.45.9%, *p* < 0.001). Maternal age, marital status, educational level, parity, birth weight, gestational age, and feeding practices were comparable between the groups. Women in the neuraxial group demonstrated better MIB scores, though not statistically significant.Impaired bonding was reported in 57.8% of the neuraxial group vs 68.8% in the general anesthesia group(*p* = 0.059).Postpartum depression and anxiety rates were low and comparable between the groups.General anesthesia was not independently associated with impaired bonding, in a multivariable model (OR 0.84, 95% CI 0.51–1.38, *p* = 0.498).

**Conclusion**: The type of anesthesia used for CD was not independently associated with impaired early MIB, suggesting that other factors, such as maternal recovery and neonatal status, may have a more significant impact on bonding during the immediate postpartum period.

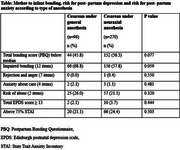

**10:30 AM – 12:00 PM**

## 790 | **Ketamine Infusion During Cesarean Deliveries and Association With Postpartum Depression**

Gary R. Kersbergen^1^; Matthew K. Hoffman^1^; Suneet P. Chauhan^2^; Jessica C. Fields^3^

^1^Christiana Care Health System, Newark, DE; ^2^Delaware Center for Maternal‐Fetal Medicine of Christiana Care, Newark, DE; ^3^Delaware Center for Maternal Fetal Medicine, Newark, DE

**Objective**: Postpartum depression (PPD) complicates 13% of pregnancies and has significant consequences for both mother and child. Recently, ketamine has been shown to have rapid anti‐depressant effects. However, there are few studies investigating the impact of ketamine on PPD. We aimed to assess PPD in patients who received ketamine as an additional analgesic during cesarean delivery.

**Study Design**: This single‐site retrospective study aimed to determine if administration of intravenous (IV) ketamine intra‐operatively at the time of cesarean delivery was associated with decreased PHQ‐9 scores postpartum, which was assessed routinely 24 hours post‐delivery. A PHQ‐9 score greater than 8 was used as an indicator of PPD. Psychiatry consults were a secondary outcome. Our analysis was stratified by patients that were already using anti‐depressants at the time of delivery.

**Results**: A total of 4897 cesareans were performed between 2014 and 2024 with 340 (6.9%) receiving IV ketamine intraoperatively (Table 1). Patients who received ketamine were more likely to have PHQ‐9 scores > 8 (6.18% vs. 3.4%; OR = 1.89, 95% CI 1.18, 3.03; *p* = 0.008). The average PHQ‐9 score for patients with ketamine was 2.28 compared to 1.88 in those without ketamine (*p* = 0.012). Similar results were noted among patients not previously taking psychiatric medications. Of patients who received ketamine, 8.24% had a psychiatry consult compared to 5.13% in patients who did not receive ketamine (*p* = 0.014). Among patients previously on psychiatric medications, patients who received ketamine had a rate of PHQ‐9 scores > 8 of 5.00%, compared to 13.4 % in patients who did receive ketamine (*p* = 0.28).

**Conclusion**: Ketamine administration during cesarean delivery is not associated with improved PHQ 9 scores in the immediate postpartum period. Administration of IV ketamine during cesarean delivery was associated with higher rates of PPD in these patients. Further investigation is warranted to determine the impact of ketamine on PPD beyond 24 hours post‐delivery.

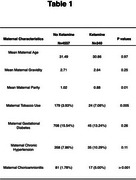

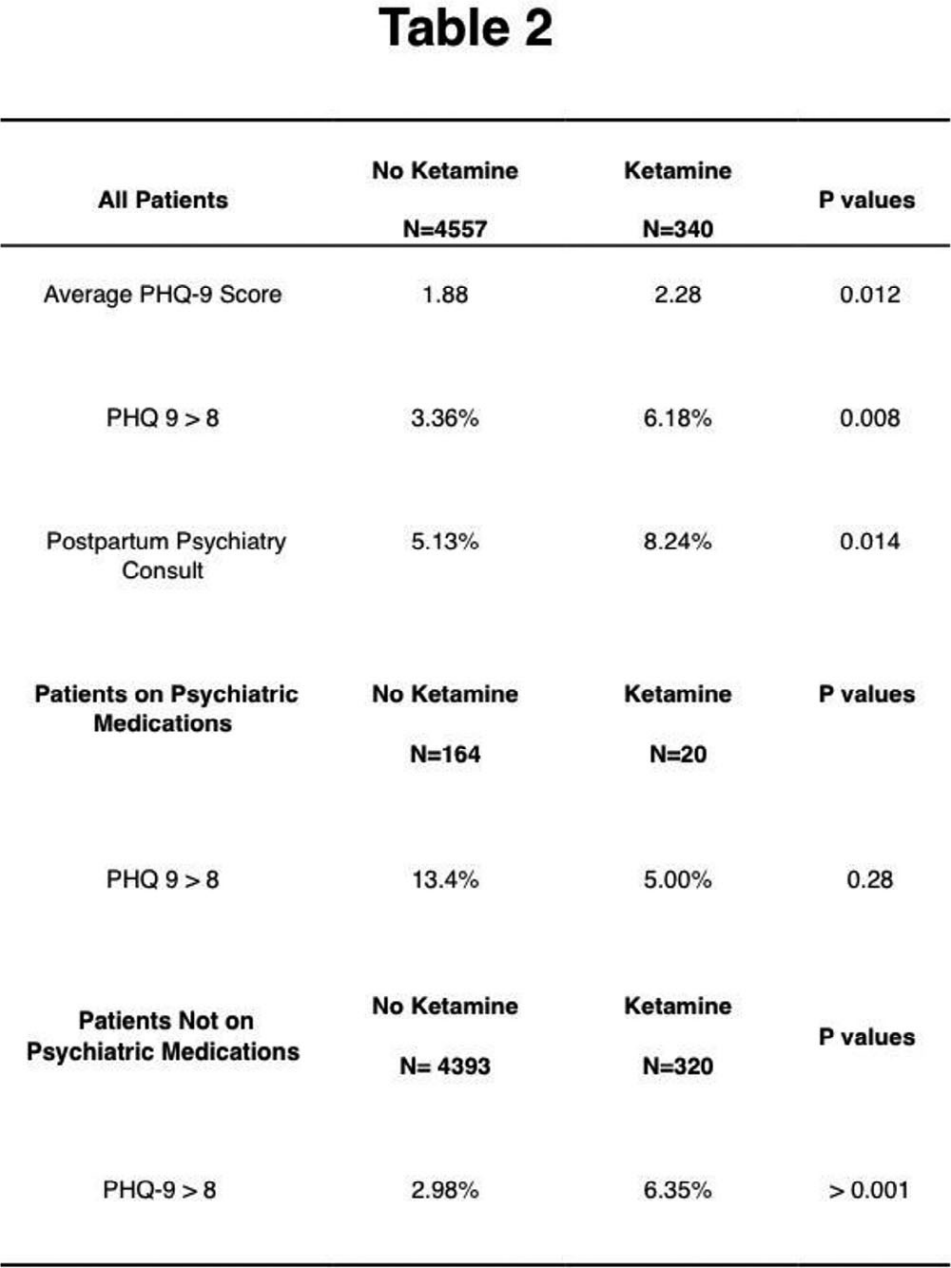

**10:30 AM – 12:00 PM**

## 791 | **Use of** Standing **Blood Pressures in Pregnant Patients with Chronic Hypertension**

Gayathri D. Vadlamudi^1^; Holt Garner^2^; April R. Gorman^1^; Anne M. Ambia^1^; C. Edward Wells^1^; Scott W. Roberts^1^; Donald D. McIntire^1^; David B. Nelson^1^

^1^University of Texas Southwestern Medical Center, Dallas, TX; ^2^UT Southwestern Medical Center/Parkland Health, Dallas, TX

**Objective**: Standing blood pressure (BP) measurements have been shown to improve detection of hypertension and cardiovascular disease in non‐pregnant individuals. This study aimed to characterize standing versus seated BP profiles across gestation in patients with chronic hypertension.

**Study Design**: This was a prospective observational study at a large academic medical center from May 2024‐June 2025 of pregnant individuals at a maternal‐fetal medicine clinic specializing in chronic hypertension. At each prenatal visit, BP was measured in seated and standing positions. Patients rested in a seated position before the first measurement, then stood for one minute before the standing measurement. Values from each visit were analyzed across gestation using random effects repeated measures models, reporting means and 95% confidence intervals. A sample size of 338 patients was estimated to detect a 3mmHg difference in seated vs standing BP with 80% power.

**Results**: There were 360 patients with chronic hypertension who met inclusion criteria, enrolled, and delivered during the study period. At entry to prenatal care, 106 (29.6%) were on antihypertensive therapy (Table 1). Seated systolic BP (SBP) was 3.5 mmHg higher than standing, with this difference increasing across gestation (Figure 1, *p* < 0.001). Early in pregnancy, seated diastolic BP (DBP) was 2 mmHg lower than standing (*p* < 0.001), with this difference diminishing with advancing gestation. Among 102 (30.2%) patients who developed superimposed severe preeclampsia, difference in SBP for seated vs standing was significantly higher than in those without severe preeclampsia (5.0 ± 6.1 vs 3.1 ± 7.3 mmHg, *p* = 0.012).

**Conclusion**: In a prospective longitudinal study of pregnant individuals with chronic hypertension, standing BP measurements differ significantly from seated values, with standing SBP lower throughout gestation. This difference appears more pronounced in those who go on to develop superimposed severe preeclampsia, suggesting potential prognostic value of using standing BPs for earlier risk stratification.

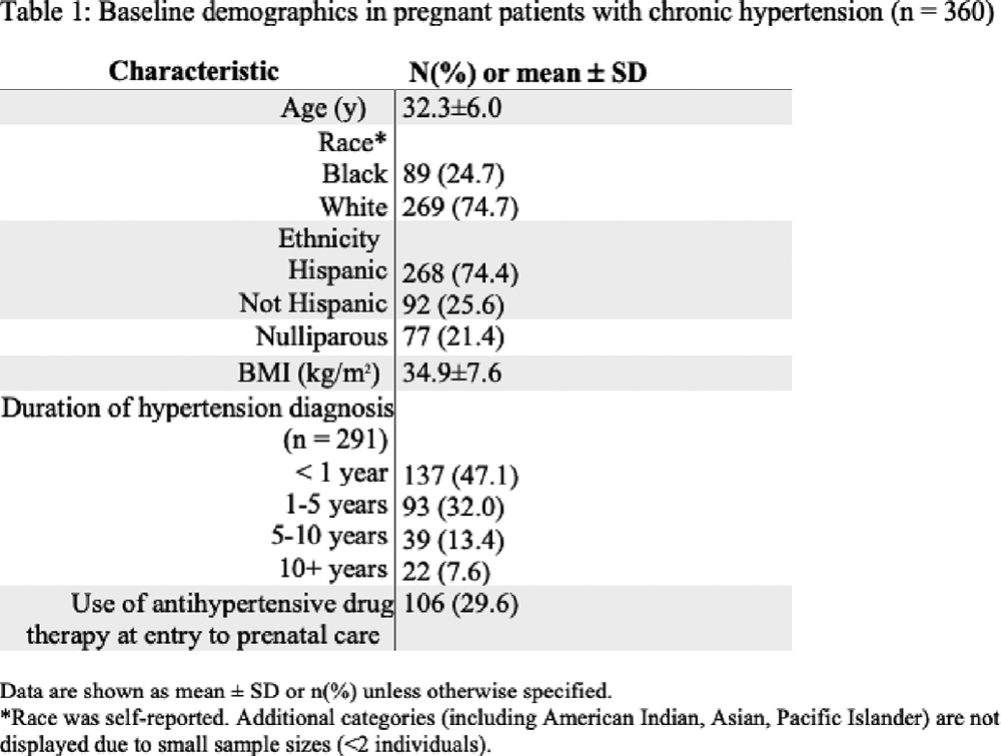

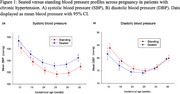

**10:30 AM – 12:00 PM**

## 792 | **Elevated Nuchal Translucency > 95%ile and Risk of Fetal Structural Abnormalities After Low Risk NIPT**

Genevieve Mazza^1^; Ellie Marlor^1^; Femitan Ajayi^1^; Jenny Chang^2^; Jonathan Steller^1^

^1^UC Irvine Medical Center, Orange, CA; ^2^University of California, Irvine, Irvine, CA

**Objective**: Ultrasound of the fetal nuchal translucency (NT) and maternal serum noninvasive prenatal testing (NIPT) are key components of first trimester genetic screening. While an elevated NT increases the risk for major fetal structural anomalies, genetic defects, and fetal loss, there is no consensus on an NT threshold that should trigger further evaluation, particularly after low risk NIPT. Recent practice patterns are de‐emphasizing the NT in favor of NIPT alone. The purpose of this study is to evaluate the utility of NT in the setting of low‐risk NIPT to prognosticate the risk for fetal structural anomalies in patients with an NT above the 95^th^ and 99^th^%iles.

**Study Design**: This was a retrospective cohort study of patients who received a first trimester NT ultrasound at our tertiary care center between 1/2021–12/2024. Those with an NT greater than the 95^th^ percentile and a low risk NIPT were included. The cohort was divided into two groups: NT between 95–98^th^ %ile and NT at or above the 99^th^ %ile to striate risk. Variables were compared using chi‐square, Fisher's exact, or Student's T‐tests as appropriate.

**Results**: 96 subjects (54 with NT 95–98%ile, 42 with NT > 99%ile) met inclusion criteria. There were no significant differences in baseline characteristics between groups. 26% of the entire cohort had > 1 abnormal finding on detailed anatomy ultrasound (16.7% of those with an NT between 95–98%ile, 38.1% of those with an NT > 99%ile; *p* = 0.007). Furthermore, 7.3% of the entire cohort had an abnormal fetal echocardiogram (1.9% of those with NT 95–98%, 14.3% of those with NT > 99%; *p* =  .0006).

**Conclusion**: In the setting of low‐risk NIPT, an elevated NT > 95%ile was associated with a 26% risk of abnormal anatomic survey and 7.3% risk of abnormal fetal echocardiogram. These rates are even higher for patients with an NT > 99%ile. The high overall rates of abnormalities in this cohort emphasizes the importance of performing both NT ultrasound and NIPT to empower counseling and patient‐centered decision‐making regarding further work‐up and management of early pregnancy.

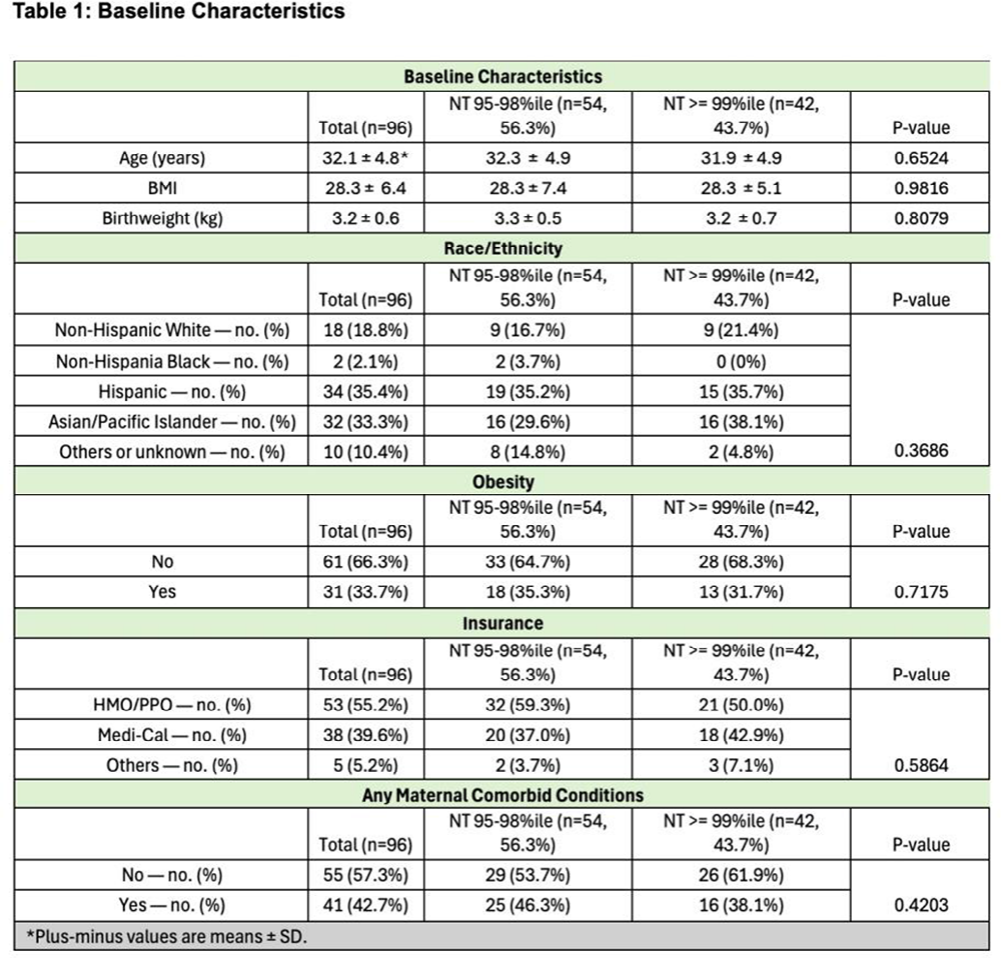

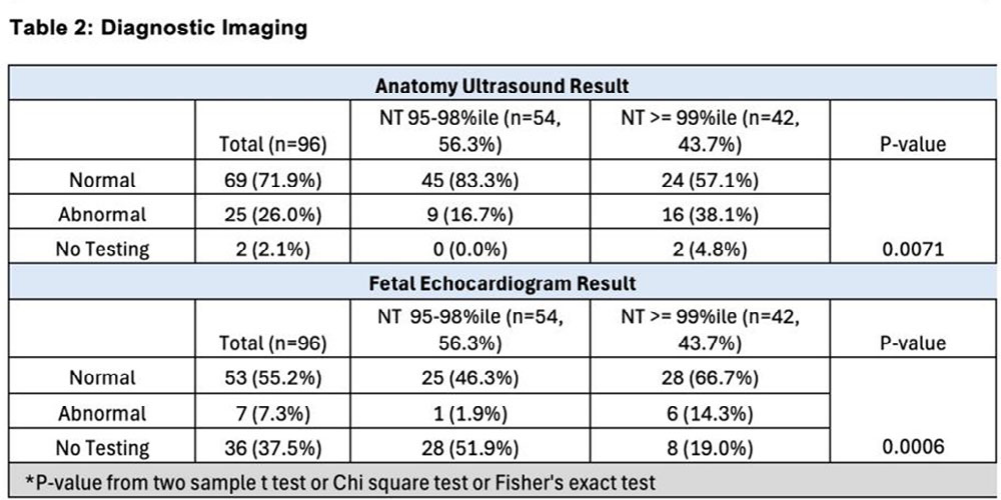

**10:30 AM – 12:00 PM**

## 793 | **Vaginal** Colonization **With Group B Streptococcus During Labor and Offspring Long‐Term Gastrointestinal Morbidity**

Gil Gutvirtz^1^; Tamar Wainstock^2^; Eyal Sheiner^3^

^1^Department of Obstetrics and Gynecology, Soroka University Medical Center, Ben‐Gurion University, Metar, HaDarom; ^2^Department of Epidemiology, Biostatistics and Community Health Sciences, Faculty of Health Sciences, Ben‐Gurion University of the Negev, Beer Sheva, HaDarom; ^3^Soroka University Medical Center, Faculty of Health Sciences, Ben‐Gurion University of the Negev, beer sheva, HaDarom

**Objective**: Vertical transmission of Group B Streptococcus (GBS) may occur during vaginal delivery when the neonate is exposed to maternal vaginal flora. To reduce early‐onset GBS disease, intrapartum antibiotic prophylaxis is routinely given to colonized mothers. Beyond infection prevention, both GBS exposure and peripartum antibiotics may influence the neonatal gut microbiome, which plays a key role in gastrointestinal (GI) development. This study aimed to evaluate whether maternal GBS colonization, despite antibiotic prophylaxis, is associated with long‐term GI morbidity in offspring.

**Study Design**: We conducted a population‐based retrospective study comparing offspring GI morbidity between those born to GBS‐positive mothers and those born to mothers with negative or unknown GBS status. The cohort included singleton vaginal deliveries between 2002–2021 at a tertiary center. Long‐term GI morbidity was assessed via outpatient and hospitalization records. A Kaplan‐Meier survival curve was constructed to compare the long‐term cumulative GI morbidity, and a Cox regression model was applied to adjust for confounders.

**Results**: Among 146, 103 singleton vaginal deliveries, 2225 (1.5%) mothers were GBS‐positive. Although overall GI morbidity rate was lower in the exposed group (Table), cumulative incidence over time was comparable between groups (Figure). Likewise, in the Cox regression model, adjusted for maternal and gestational age, diabetes mellitus, hypertensive disorders and ethnicity, GBS colonization of the maternal genital tract was not associated with long‐term GI morbidity of the offspring (adjusted HR = 0.98, 95% CI 0.89–1.08, *p* = 0.726).

**Conclusion**: In this large population‐based cohort, maternal vaginal GBS colonization during vaginal delivery, for which intrapartum antibiotic prophylaxis is routinely administered, was not associated with increased long‐term GI morbidity in offspring. These findings suggest that exposure to maternal GBS colonization and peripartum antibiotics does not adversely affect the risk of developing long‐term GI diseases in children.

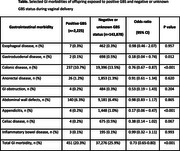

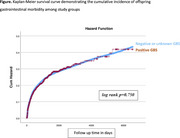

**10:30 AM – 12:00 PM**

## 794 | **Unveiling the Long‐Term Neurological Impact of Antenatal Corticosteroid Therapy on Preterm Infants**

Gil Gutvirtz^1^; Gali Pariente^2^; Tamar Wainstock^3^; Eyal Sheiner^4^

^1^Department of Obstetrics and Gynecology, Soroka University Medical Center, Ben‐Gurion University, Metar, HaDarom; ^2^Department of Obstetrics and Gynecology, Soroka University Medical Center, Ben‐Gurion University, beer sheva, HaDarom; ^3^Department of Epidemiology, Biostatistics and Community Health Sciences, Faculty of Health Sciences, Ben‐Gurion University of the Negev, Beer Sheva, HaDarom; ^4^Soroka University Medical Center, Faculty of Health Sciences, Ben‐Gurion University of the Negev, beer sheva, HaDarom

**Objective**: Administration of antenatal corticosteroid (ACS) in pregnancies at risk for preterm birth reduces neonatal morbidity and mortality. However, concerns have emerged regarding harmful long‐term neurological effects, especially in those born in the late preterm period. We investigated the long‐term neurological outcomes of preterm offspring (<37 weeks’ gestation) who were exposed to ACS prior to 34 weeks of gestation.

**Study Design**: A population‐based retrospective study of preterm singleton deliveries between 1998–2021 was conducted. We compared the long‐term neurological morbidity of preterm offspring who were exposed to ACS prior to 34 weeks of gestation with unexposed children, based on combined databases from both community and hospitalization records. Kaplan–Meier survival curves were used to compare the cumulative incidence of neurological morbidity and Cox regression models were constructed to control for confounders.

**Results**: 13, 580 preterm deliveries were included; 1, 538 (11.3%) children were exposed to ACS prior to 34 weeks. Neurological morbidity incidence per 1000 person‐years (Table) as well as the cumulative incidence over time (Figure 1a) were significantly higher in ACS exposed infants as compared to unexposed children. Similar results were found specifically among the late preterm group (34–36+6 weeks; Figure 1b). Cox regression models controlling for maternal and gestational age, ethnicity, child's birth year, diabetes mellitus, hypertensive disorders and cesarean delivery, found that ACS exposure before 34 weeks is an independent risk factor for long‐term neurological morbidity for all preterm infants (aHR 1.17, 95% CI 1.09–1.26, *p* < 0.001), as well as for the late preterm infants (aHR 1.19, 95% CI 1.08–1.31, *p* < 0.001).

**Conclusion**: While the importance of ACS in reducing morbidity and mortality of preterm offspring is well recognized, the long‐term neurological effects remain a concern. Cautious use of ACS is advised, particularly when preterm birth is uncertain, and further research is essential to establish the long‐term safety profile of ACS.

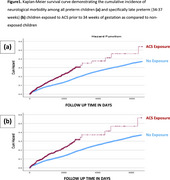

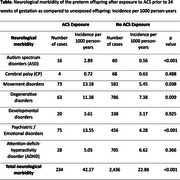

**10:30 AM – 12:00 PM**

## 795 | **Climate** as **a Potential Modifier of Aspirin's Dose–Response for Preeclampsia Prevention: Meta‐Analysis of RCTs**

Giulia Bonanni^1^; Faezeh Aghajani^2^; Samaneh Saraeian^3^; Kamyar Ebrahimi^4^; Izat Khawajah^5^; Blair J. Wylie^6^; Vincenzo Berghella^7^; Kjersti M. Aagaard^8^; Alireza A. Shamshirsaz^9^

^1^Division of Fetal Medicine and Surgery, Boston Children's Hospital Harvard Medical School, Boston Children's Hospital, MA; ^2^Boston Children's Hospital, Boston, MA; ^3^Independent Scholar, East Lansing, MI; ^4^Shiraz University of Medical Science, Shiraz, Fars; ^5^Tehran University of Medical Sciences, Tehran, Tehran; ^6^Beth Israel Deaconess Medical Center, Boston, MA; ^7^Thomas Jefferson University, Philadelphia, PA; ^8^HCA, Houston, TX; ^9^Division of Fetal Medicine and Surgery, Boston Children's Hospital Harvard Medical School, Boston, MA

**Objective**: Higher average daily temperatures have been linked to adverse outcomes such as cardiovascular disease; low dose aspirin is thought to mediate this effect. We aimed to evaluate the dose–response relationship between aspirin and preeclampsia and assess whether this is modified by climate and initiation timing.

**Study Design**: We conducted a one‐stage dose–response meta‐analysis of randomized controlled trials (RCTs) evaluating aspirin for preeclampsia prevention. Eligible RCTs enrolled moderate‐ or high‐risk pregnant individuals randomized to any daily aspirin dose versus placebo/no treatment. Random‐effects meta‐regression models with restricted cubic splines (REML) were stratified by climate (cool vs warm), defined using annual temperatures (CRU CY v4.09) averaged by trial and dichotomized at the median across included trials (11.9°C). Meta‐regression tested interactions between dose, climate and initiation timing (< 16 vs ≥16 weeks).

**Results**: Of 51 eligible RCTs, 34 with dose‐specific data were included. Aspirin reduced preeclampsia risk in a dose‐dependent manner. In warm climates, the dose–response curve was non‐linear (LRT *p* < 0.00003), with steepest risk reduction between 100–150 mg/day (Figure 1A). Predicted RRs versus placebo were 0.66 (95% CI: 0.55–0.79) at 81 mg, 0.51 (0.40–0.65) at 100 mg, and 0.26 (0.14–0.48) at 150 mg (Table 1A). In cooler climates, benefit was more modest and dose‐dependent effects less apparent. Three‐way meta‐regression confirmed modification by initiation timing (*p* = 0.0004) and by high‐dose × warm‐climate interaction (*p* = 0.0044), fully explaining between‐study heterogeneity (I^2^ = 0%) (Table 1B). Subgroup analyses showed strongest benefit in warm climates with initiation ≥16 weeks (RR = 0.63 [0.42–0.95]) (Figure 1B).

**Conclusion**: Aspirin's effectiveness varies by dose, timing of initiation, and climate. In warm climates, ≥150 mg/day started ≥16 weeks reduced risk by 74%. Climate may proxy other modifiers, but its strong interaction with dose suggests relevance for precision prevention and supports geographically informed prophylaxis and policies aimed at climate stabilization.

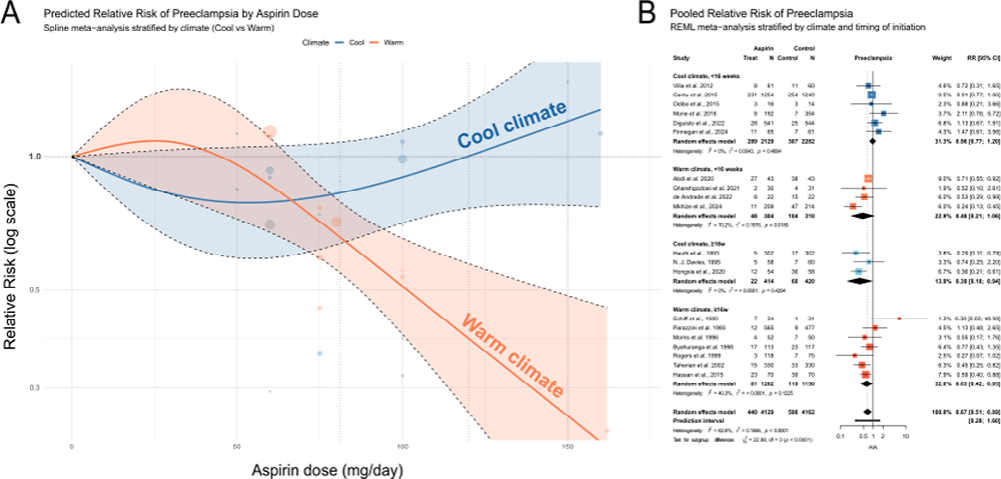

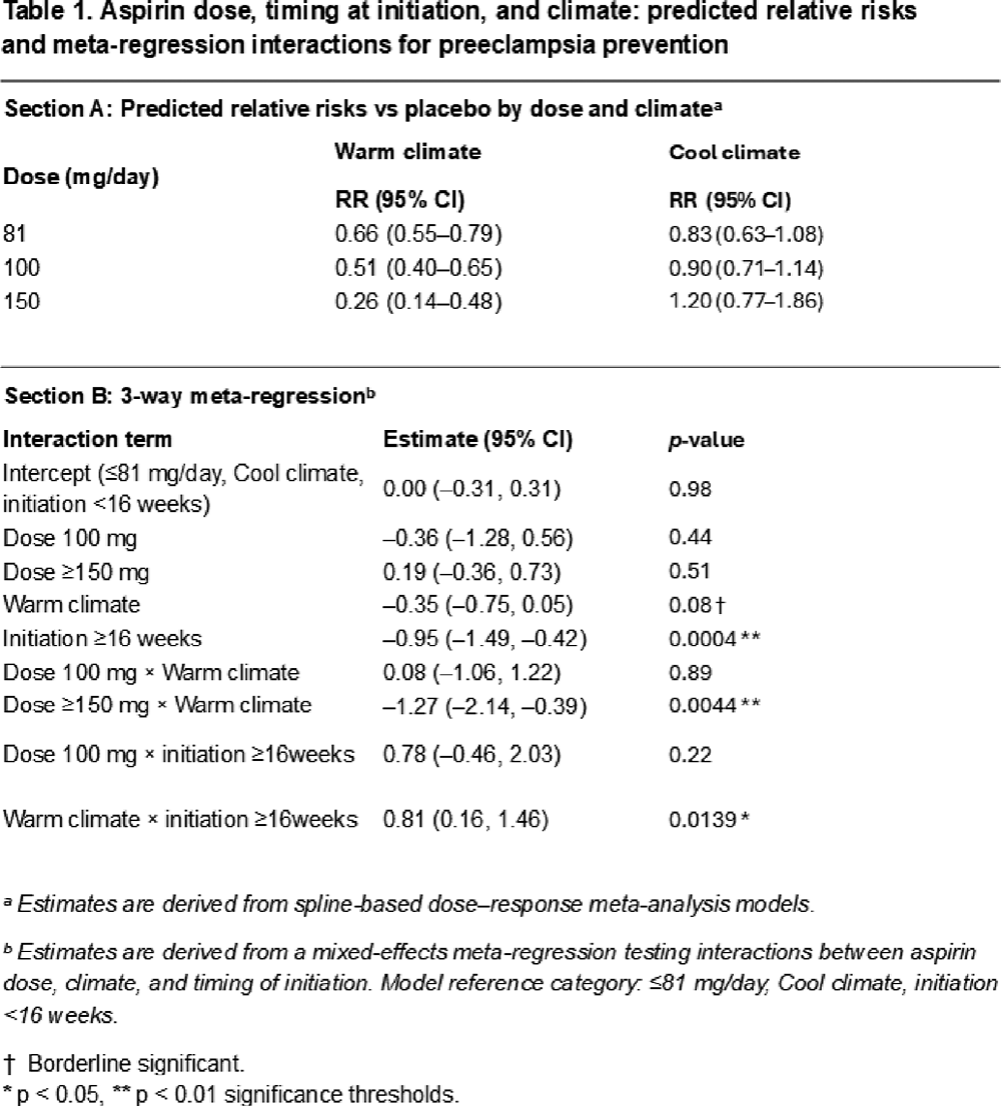

**10:30 AM – 12:00 PM**

## 796 | Gardnerella **Vaginalis Induces Lipid Peroxidation and Superoxide Dismutase Activity in the Cervicovaginal Space**

Gregory W. Kirschen^1^; Kristin M. Klohonatz^1^; Lauren Anton^1^; Kristin D. Gerson^2^

^1^University of Pennsylvania Perelman School of Medicine, Philadelphia, PA; ^2^University of Pennsylvania, Hospital of the university of Pennsylvania, PA

**Objective**: *Gardnerella vaginalis* (GV) colonization of the cervicovaginal (CV) space is associated with adverse perinatal outcomes including preterm birth (PTB). Mechanisms by which GV drives outcomes remain largely unknown. Oxidative stress promotes physiologic cervical ripening in term parturition. We hypothesized that GV induces oxidative imbalance in the CV epithelial barrier as a potential mechanism underlying premature cervical remodeling preceding PTB.

**Study Design**: We co‐cultured GV or *Lactobacillus crispatus* (LC), a healthy vaginal microbe, at 10^5^–10^7^ CFU/well with ectocervical (Ecto), endocervical (Endo), and vaginal (VK2) epithelial cells for 24h. We assessed two markers of oxidative stress: 1) thiobarbituric acid reactive substances (TBARS), a byproduct of lipid peroxidation, was measured in cell culture media; and 2) activity of superoxide dismutase (SOD), a key antioxidant enzyme, was measured in cell lysate. Data were analyzed by one‐way ANOVA with Tukey's test for multiple comparisons.

**Results**: GV induced TBARS for Ecto, Endo, and VK2 cells in a dose‐dependent manner across all cell lines compared to non‐treated (NT) and microbial control LC (*p* < 0.05 for all, Figure 1). GV similarly induced SOD activity in a dose‐dependent manner across all cell lines compared to NT and LC controls (*p* < 0.05 for all, Figure 2).

**Conclusion**: A vaginal anaerobe implicated in the pathophysiology of PTB induces oxidative stress in the CV epithelium. These molecular events likely contribute to the molecular pathophysiology of premature cervical remodeling. Antioxidant‐based therapeutics may serve as a novel strategy to reduce PTB among individuals with microbiota‐related risk. 1R01HD114611–01 (KDG)

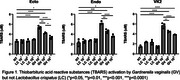

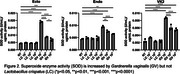

**10:30 AM – 12:00 PM**

## 797 | Integrative **Medicine Therapies in Hospitalized Antepartum Patients: Utilization Patterns and Impact on Discomfort Scores**

Hadley Ross^1^; Casandra Chouravong^1^; Ana Vera^1^; Adah Beck^1^; Ariana Braddom^1^; Alixandria F. Pfeiffer^2^; Jan Patterson^1^; Angela R. Boyd^1^

^1^UT Health San Antonio, San Antonio, TX; ^2^University of Texas Health San Antonio, University of Texas health San Antonio, TX

**Objective**: Integrative medicine (IM) therapies are increasingly used alongside conventional treatments to manage pain, stress, anxiety, and prolonged hospitalizations. However, IM remains understudied in obstetric populations. We evaluated the utilization of IM therapies and their impact on discomfort scores among hospitalized antepartum patients.

**Study Design**: We conducted a retrospective study of antepartum patients admitted to a tertiary care center who received at least one IM consultation between 1/2024 and 7/2025. IM therapies included aromatherapy, meditation and breathwork, sound, light, and relaxation therapy. Demographics, admission characteristics, IM modalities, and discomfort scores were abstracted from the medical record. Discomfort scores were measured on a generic 0–4 scale (0 = no discomfort, 4 = severe discomfort). Patients without both pre‐ and post‐intervention discomfort scores were excluded from analysis of discomfort scores. Paired scores were compared using the Wilcoxon signed‐rank test.

**Results**: Of 207 antepartum patients who received IM consultations, 174 (84%) had both pre‐ and post‐intervention discomfort scores available. Median gestational age at admission was 30 weeks, and median length of stay was 7 days. The most common admission indication was hypertensive disorders (31%). Most patients delivered during their hospitalization, with cesarean delivery more common than vaginal delivery (43% vs. 28%). Most patients received multiple IM modalities during a single visit, with aromatherapy (94%) and mind–body therapies such as meditation and breathwork (58%) being the most frequently used. Median discomfort scores decreased from 2 pre‐IM to 1 post‐IM (*p* < 0.0001).

**Conclusion**: In our hospitalized antepartum patients, IM therapies frequently involved multiple modalities and were associated with improvement in discomfort scores. These findings support the potential value of IM as a holistic intervention in the care of hospitalized antepartum patients. Prospective studies are needed to confirm and expand these results and evaluate effects on maternal and neonatal outcomes.

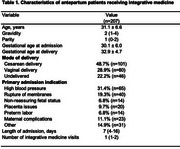

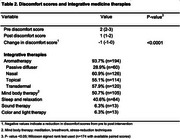

**10:30 AM – 12:00 PM**

## 798 | Identifying **Optimal Maternal Oxygen Saturation Targets Using Electronic Fetal Monitoring**

Haley E. Baker^1^; Michael Dombrowski^2^; Amanda C. Zofkie^2^; Heather Campbell^3^; Antonina I. Frolova^2^; Jeannie C. Kelly^4^; George A. A. Macones^5^; Alison G. G. Cahill^6^; Nandini Raghuraman^2^

^1^Washington University School of Medicine, Washington University School Of Medicine, MO; ^2^Washington University School of Medicine, St. Louis, MO; ^3^Washington University School of Medicine, St Louis, MO; ^4^Washington University School of Medicine, Washington University School of Medicine, MO; ^5^Department of Women's Health, Dell Medical School at the University of Texas at Austin, Austin, TX; ^6^Department of Women's Health, Dell Medical School at the University of Texas at Austin, Department of Women's Health, Dell Medical School at the University of Texas at Austin, TX

**Objective**: SMFM and ACOG recommend maintaining a maternal oxygen saturation goal of 95% to promote fetal oxygenation. However, there is a paucity of data supporting this target and some argue for permissive relative hypoxemia. We assessed the relationship between intrapartum maternal pulse oximetry and high‐risk electronic fetal monitoring (EFM) features.

**Study Design**: This is a secondary analysis of a prospective cohort of term, singleton pregnancies admitted for induction or spontaneous labor from 2010–2015. EFM data from the final 60 minutes (min) were abstracted by nurses blinded to outcome. Patients were included if they had at least one maternal oxygen saturation (O_2_ sat) measurement at the start of the 60 min window. Those with O_2_ sat < 90% were excluded due to concerns for artifact or underlying pathology. The primary outcome was a composite of high risk EFM features associated with acidemia (recurrent late or variable decelerations, >1 prolonged deceleration, minimal variability, tachycardia). Secondary outcome was umbilical artery (UA) pH. Outcomes were compared between patients with O_2_ sat 90–94% versus ≥ 95% using bivariate analyses and multivariable logistic regression. Sensitivity analyses included patients with multiple O_2_ sats < 95% in the last hour of labor.

**Results**: Of 6, 701 patients with O_2_ sat and EFM data, 1, 824 (27%) had O_2_ sat 90–94% and 4, 877 (73%) had O_2_ sat ≥ 95%. Composite high risk EFM features were not significantly different between those with O_2_ sat 90–94% vs ≥ 95% (aOR 0.94 (CI 0.84–1.05)). UA pH was similar between O_2_ sat groups. (Table). There was no trend toward improved EFM with higher O_2_ sat (Figure). Sensitivity analyses among those with multiple O_2_ sat < 95% showed similar results. Results were similar when stratified by stage of labor at time of delivery.

**Conclusion**: Maternal O_2_ sats 90–94% appear well tolerated by the fetus as indicated by EFM features and UA pH. These findings suggest that transient, relative maternal hypoxemia in this range may be clinically acceptable in labor.

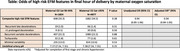

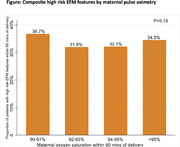

**10:30 AM – 12:00 PM**

## 799 | **Language Barriers and Obstetric Hemorrhage: Comparing Outcomes by Interpreter Requirement**

Hannah Caldwell^1^; Caitlyn Lee^1^; Vanessa Chin^2^; Hannah Tracy^1^; Heidi K. Leftwich^1^

^1^UMass Chan Medical School, Worcester, MA; ^2^UMass Chan Medical School, UMass Chan Medical School, MA

**Objective**: To evaluate whether interpreter requirement, as a proxy for limited English proficiency (LEP), is associated with differences in management and outcomes among patients with documented postpartum hemorrhage (PPH).

**Study Design**: We conducted a retrospective cohort study of 3, 199 patients with PPH (≥1000 mL blood loss) who delivered at a single tertiary care center from 2020–2023. Patients were stratified by interpreter requirement at admission. T‐tests and chi‐square analyses compared transfusion timing and volume, mode of delivery, and maternal and neonatal outcomes between groups.

**Results**: Of patients with PPH, 537 (16.8%) required an interpreter. Patients who required an interpreter were slightly younger (30.8 vs 31.7 years, *p* = 0.002), more likely to identify as Hispanic (63.5% vs 18.7%), and more racially diverse. Interpreter requirement was associated with lower average blood loss (1615 vs 1711 mL, *p* = 0.004) and lower NICU admission rates (16.6% vs 23.1%, *p* < 0.001). There were no significant differences in transfusion rate (10.8% vs 9.0%, *p* = 0.19), number of units transfused, or time to administration of uterotonic medications. ICU admission (0.9% vs 1.7%, *p* = 0.18) and cesarean delivery (81.6% vs 78.8%, *p* = 0.15) rates were also similar.

**Conclusion**: Among patients with PPH, interpreter requirement was not associated with clinically significant differences in blood loss, transfusion rates, or maternal ICU admission. These findings suggest that patients with LEP may receive comparable hemorrhage care once bleeding is identified. Further investigation is ongoing to evaluate whether language barriers impact timing of care escalation and length of stay after obstetric hemorrhage.

**10:30 AM – 12:00 PM**

## 800 | **Impact of Interprofessional Mentorship on Nurse Attitudes Towards Physician–Nurse Collaboration in Obstetrics**

Hannah Kelly^1^; Aya Bashi^1^; Melissa Katella^1^; Rachel L. Wood^2^; Anne West‐Honart^2^

^1^Duke University Hospital, Durham, NC; ^2^Division of Maternal Fetal Medicine, Department of Obstetrics and Gynecology, Duke University, Durham, NC

**Objective**: Interprofessional collaboration is essential for communication, patient safety, and psychological safety in high‐acuity obstetric settings. To improve teamwork on labor and delivery (L&D), our program launched “Adopt‐an‐Intern” (Figure 1), a mentorship program pairing L&D nurses with incoming OB/GYN interns. This study evaluated the program's impact on nurses’ attitudes toward nurse–physician collaboration.

**Study Design**: This prospective, cross‐sectional study used the Jefferson Scale of Attitudes Toward Physician–Nurse Collaboration (JSAPNC) to survey nurses who served as mentors and matched controls. Nursing background data were also collected. The JSAPNC measures attitudes in four domains: collaboration, nurse's role in care, nurse autonomy, and physician authority. The study was IRB‐exempt, and responses were collected in REDCap. Baseline characteristics were compared using t‐tests and chi‐square tests. Group differences in JSAPNC scores were analyzed using t‐tests or Mann–Whitney U tests, and effect sizes were calculated using Cohen's d.

**Results**: Fifty‐seven nurses completed the survey (15 mentors, 42 controls). Groups were similar at baseline with similar years of L&D experience (Mentor: 7.2 ± 5.9 vs. Control: 5.7 ± 8.0; *p* = 0.46) and day/night shift distribution (*p* = 0.73). Scores across all domains were consistently higher among mentors (Table 1). Although no differences reached statistical significance (all *p* >0.05), effect sizes were uniformly positive (Cohen's d≈0.10–0.25). When all domains were combined into a composite score, mentors had a higher mean (14.25 ± 1.00 vs. 14.05 ± 0.70; Cohen's d = 0.25), indicating a small effect favoring the intervention.

**Conclusion**: Participation in Adopt‐an‐Intern was associated with more favorable nurse attitudes toward team collaboration across all JSAPNC domains, though without statistical significance. While volunteer selection bias may play a role, results suggest interprofessional mentorship enhances perceptions of teamwork. Next steps include assessing the program's effect on physicians and patient‐centered outcomes.

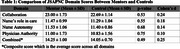

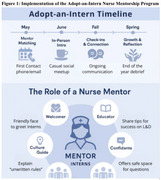

**10:30 AM – 12:00 PM**

## 801 | **The** Frequency **and Severity of Complications After Previable PROM in Colorado Following Dobbs Decision**

Hannah E. Vincent^1^; Bethany Waites^1^; Roopjit K. Sahi^2^; Neha Ali^3^; Virginia Lijewski^1^; Leilah Zahedi‐Spung^4^; Manesha Putra^3^

^1^University of Colorado, Aurora, CO; ^2^University of Colorado Anschutz Medical Campus, University of Colorado Anschutz Medical Campus, CO; ^3^university of Colorado Anschutz Medical Campus, Aurora, CO; ^4^University of Colorado School of Medicine, University of Colorado School of Medicine, CO

**Objective**: Previous studies have shown an increase in complications after a restrictive abortion law. In these studies, the delays in care were due to confusion around the laws preventing physicians from providing immediate intervention. We aim to compare the outcomes of pregnancies with previable PROM pre‐ and post‐Dobbs decision in a state with protected abortion care access.

**Study Design**: This is a retrospective cohort study of a tertiary care center from January 1, 2018‐December 31, 2024. Pre‐existing perinatal database (PARITY) was queried to identify singleton pregnancy with the diagnosis of previable PROM before 22 weeks. Baseline demographics, and clinical characteristics were abstracted by the authors. The study cohort is divided into pre‐ and post‐Dobbs group (June 24th, 2022) and clinical characteristics and outcomes were compared. Our primary outcome was composite adverse outcome including maternal ICU admission, transfusion, or sepsis. Multivariable logistic regression was used to adjust for confounding factors.

**Results**: A total of 237 pregnancies were included in this analysis, 134 pregnancies pre‐Dobbs and 103 pregnancies post‐Dobbs. There were no clinically significant differences in baseline characteristics in the study groups (Table 1). There was no difference in immediate delivery rate pre‐ 20.9% vs 21.6% post‐Dobbs (*p* < 0.9). There was also no difference in time from rupture to delivery pre‐ and post‐Dobbs (median [IQR]: 1 [1–8.75] days vs 2 [1–8] days, *p* 0.56). There was no significant difference in incidence of sepsis (5.2% pre‐Dobbs vs 3.9% post‐Dobbs, *p* 0.62). After adjusting for age, race and ethnicity, and gestational age at delivery there was no difference in the rate of composite adverse outcomes (12.7% pre‐Dobbs vs 9.7% post‐Dobbs, aOR = 0.69, CI 0.30, 1.63).

**Conclusion**: We did not see an increase in composite adverse outcomes pre‐ and post‐Dobbs in a state with protected abortion care access. The increase previously seen in abortion restrictive states likely is associated to state specific legislation that leads to delays in evidence‐based care.

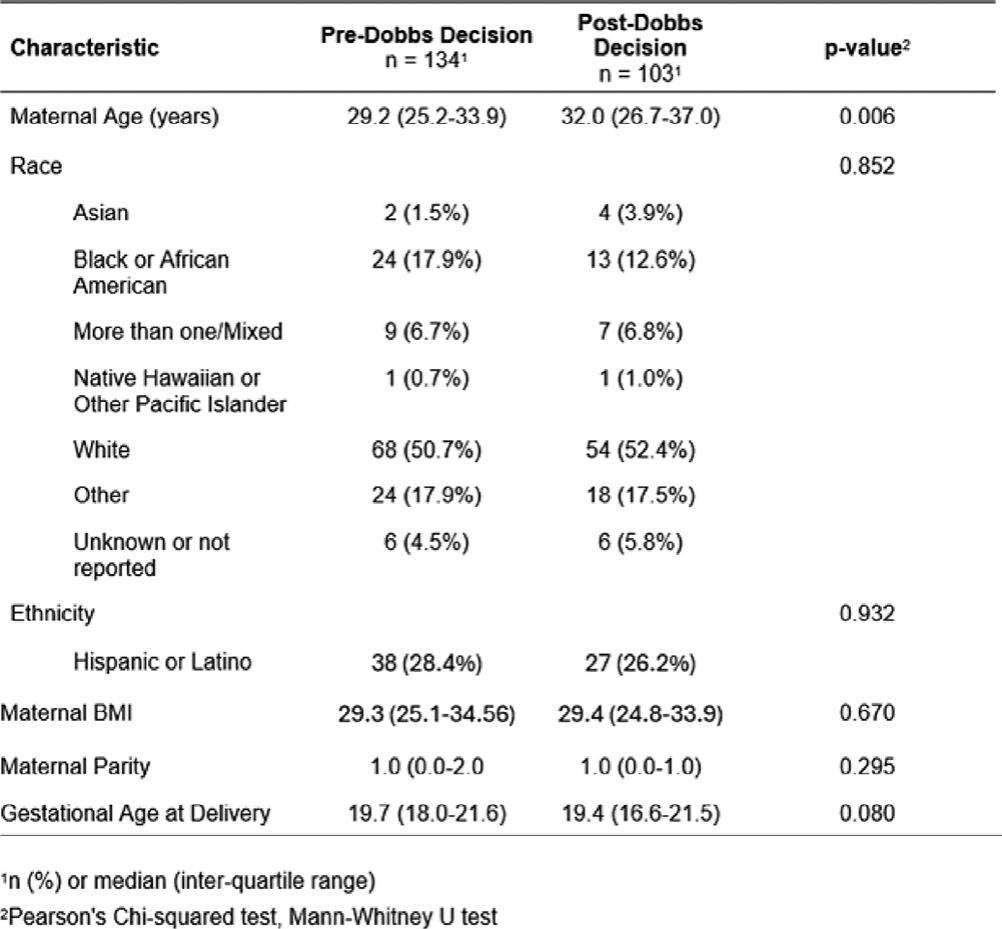

**10:30 AM – 12:00 PM**

## 802 | Improving **Perinatal Depression Screening Adherence via EMR Integration: A Phased Health System Implementation Study**

Hari Vedantam^1^; Melanie Danso^2^; Kim Brooks^3^; Jason Straub^4^; Jamie Swietlikowski^5^; Cybill Esguerra^6^; Marika Toscano^7^

^1^Johns Hopkins University Bloomberg School of Public Health, Johns Hopkins University Bloomberg School of Public Health, MD; ^2^Johns Hopkins University School of Medicine, Johns Hopkins University School of Medicine, MD; ^3^Johns Hopkins University, Johns Hopkins Hospital, MD; ^4^Johns Hopkins Hospital, Johns Hopkins Hospital, MD; ^5^John Hopkins Hospital, Johns Hopkins Hospital, MD; ^6^Johns Hopkins University, Johns Hopkins University, MD; ^7^Johns Hopkins University, Baltimore, MD

**Objective**: To enhance perinatal depression screening adherence by implementing a systematic, EMR‐integrated Edinburgh Postnatal Depression Scale (EPDS) workflow across outpatient prenatal clinics in a large health system.

**Study Design**: This quality improvement initiative was phased through 12 outpatient prenatal clinics in one health system from 7/2023–7/2025, divided into a pilot clinic (Cohort 1), four intermediate clinics (Cohort 2), and seven final clinics (Cohort 3). EPDS screening in English and Spanish was embedded into the EMR via patient portal check‐in before visits at three time points: initial prenatal, 26–30 weeks’ gestation, and postpartum. The primary outcome was EPDS screening adherence during two follow‐up periods—0–6 months and 7–12 months post‐implementation. A mixed‐effects logistic regression model with random intercepts for clinic sites and time periods nested within cohorts accounted for data hierarchy and clustering. Analyses were conducted in R.

**Results**: Among 9, 614 patients (Cohort 1 *n* = 2, 438; Cohort 2 *n* = 2, 848; Cohort 3 *n* = 4, 328), antepartum EPDS screening was strongly associated with higher adherence odds (aOR 11.9, 95% CI 8.8–16.2, *p* < 0.001). Compared to Cohort 1, patients in Cohort 3 had lower odds of adherence (aOR 0.26, 95% CI 0.14–0.48, *p* < 0.001), suggesting less sustained effect with phased implementation. Racial disparities persisted: Black (aOR 0.56, 95% CI 0.45–0.70, *p* < 0.001) and Other race (aOR 0.47, 95% CI 0.37–0.59, *p* < 0.001) groups had reduced adherence compared to White patients. Payor type, Hispanic ethnicity, and Asian race were not significantly associated.

**Conclusion**: The phased intervention improved antepartum screening adherence; however, sustained postpartum improvements were not observed. Persistent racial disparities highlight the need for targeted efforts to promote equity and address postpartum screening barriers, to sustain gains and improve perinatal mental health outcomes.

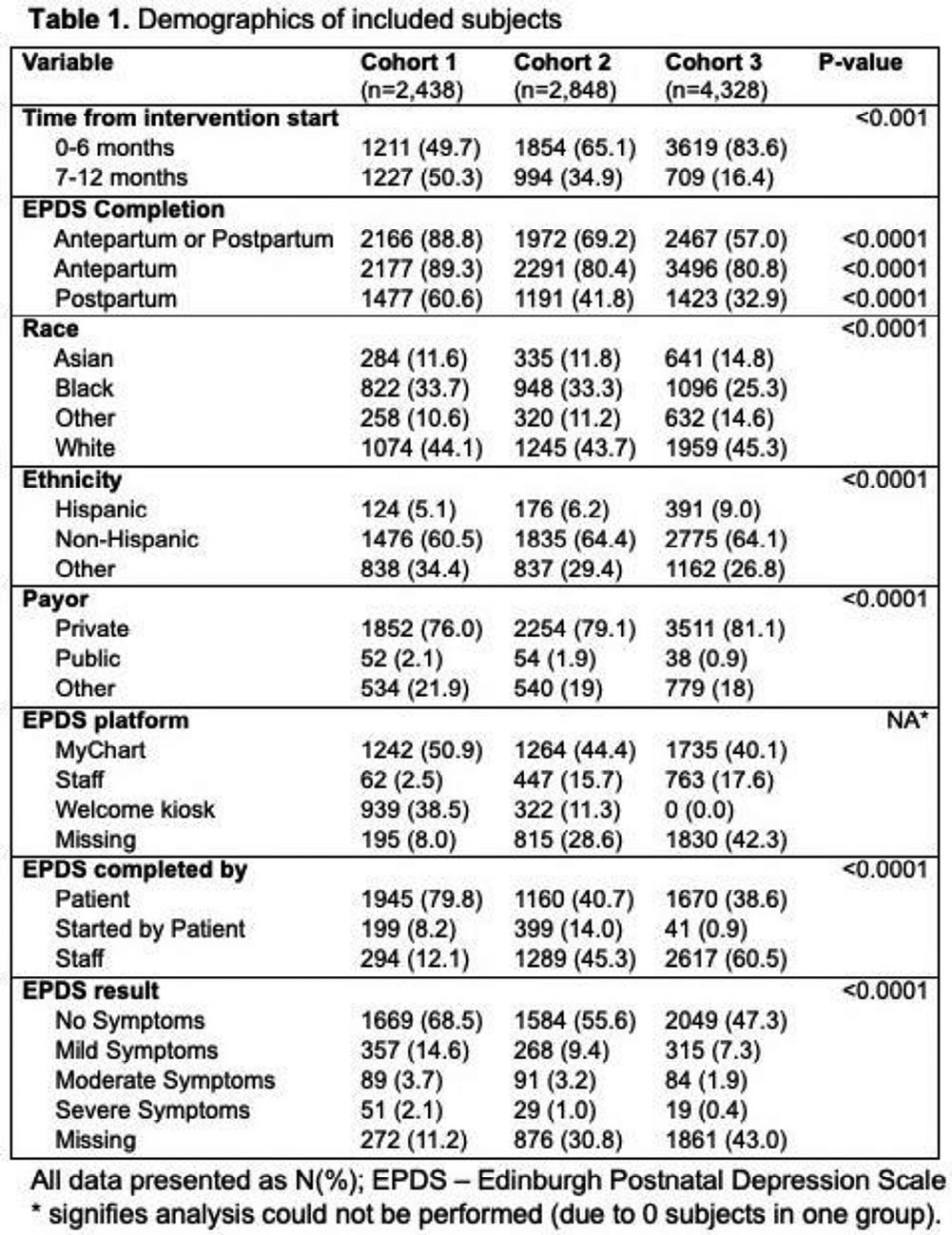

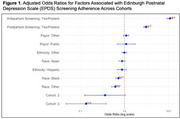

**10:30 AM – 12:00 PM**

## 803 | **Risk of** Hypertensive **Disorders of Pregnancy with Pre‐Pregnancy GLP‐1 Agonist Exposure**

Harsheen Chawla^1^; Andrea Neira Gesteira^2^; Maggie Ross^3^; Mariella Gastanaduy^4^; Meena Mishra^5^; Alexis Cates^3^; Frank B. Williams^3^

^1^Ochsner Clinic Foundation, New Olreans, LA; ^2^Ochsner, ochsner/New Orleans, LA; ^3^Ochsner Clinic Foundation, Ochsner Clinic Foundation, LA; ^4^Ochsner Center for Outcomes Research, New Orleans, LA; ^5^Ochsner Center of Outcomes & Health Services Research, New Orleans, LA

**Objective**: Use of glucagon‐like‐peptide‐1 (GLP1) agonists has dramatically increased, with 12% of US adults reporting use in 2024, including half for weight loss indications alone. Limited data exists on pre‐pregnancy GLP1 use with pregnancy outcomes. We aim to evaluate the association between preconception GLP1 use and subsequent pregnancy‐associated hypertension (PAH).

**Study Design**: We performed a retrospective cohort study of those receiving prenatal and delivery care in a large regional health system from 2022–2024. We included pregnancies with establishment of prenatal care before 20 weeks gestation that delivered after 20 weeks. Documented pregestational diabetes, prior bariatric surgery, and multifetal pregnancies were excluded. Baseline characteristics and comorbidities such as chronic hypertension (CHTN) were extracted from the electronic medical record. Recorded GLP1 use in the year prior to pregnancy defined exposure; controls had no documented use. Primary outcome was diagnosis of gestational hypertension or preeclampsia. Regression analyses controlling for age, BMI, and CHTN generated adjusted odds ratios with 95% confidence intervals.

**Results**: Of 24, 403 eligible pregnancies, 165 (0.7%) were exposed to GLP1 agonists in the twelve months prior to pregnancy while 24, 238 (99.3%) were classified as controls. As expected, the GLP1 group had higher baseline BMI. The GLP1 group likewise had higher age and nulliparity. Controls were more likely to be Black and use Medicaid insurance (Table 1). CHTN was more prevalent in the GLP1 group (13% vs 3%, *p* < 0.0001). PAH occurred in 31.5% of the GLP1 agonist group compared to 17.4% among controls, a difference which persisted even when controlling for age, BMI and CHTN (aOR 1.9, 95% CI 1.4–2.7, Figure 1). No difference was observed in PAH with severe features among GLP1 exposed (4.9%) vs controls (4.1%, aOR 1.3, 95% CI 0.6, 2.5).

**Conclusion**: Preconception GLP1 agonist use was associated with increased PAH even when controlling for well‐established risk factors associated.

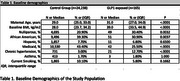

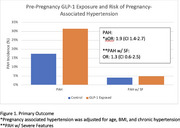

**10:30 AM – 12:00 PM**

## 804 | **Perinatal Health Perception Moderates Transcranial Direct Current Stimulation**

Hee Young Cho, N/A^1^; Sra Jung^2^; Min‐Kyoung Kim^3^

^1^Obstetrics and Gynecology, Seoul National University College of Medicine, Seoul, Kyonggi‐do; ^2^Department of Psychiatry, CHA University Ilsan CHA Hospital, Goyang‐si, Kyonggi‐do; ^3^Department of Psychiatry, CHA University Ilsan CHA Hospital, CHA University School of Medicine, Goyang‐si, Kyonggi‐do

**Objective**: Perinatal depression often remains undertreated due to concerns regarding antidepressant exposure during fertility treatment, pregnancy, or breastfeeding. Non‐pharmacological, home‐deliverable interventions such as transcranial direct current stimulation (tDCS) present a promising alternative; however, real‐world evidence in perinatal populations remains limited.

**Study Design**: This prospective observational study included 38 women who received infertility, pregnancy, or postpartum treatment at four x hospitals in South Korea. The participants self‐administered anodal tDCS targeting the left dorsolateral prefrontal cortex for 20–28 sessions over four weeks. Depressive symptoms were assessed using the Center for Epidemiologic Studies Depression Scale (CES‐D) at baseline and at weeks 2, 4, and 8. Subjective health perception was measured at baseline using a 5‐point Likert scale.

**Results**: Time had a significant effect on depressive symptoms (Wald χ^2^ = 90.75, *p* < 0.001), with the largest symptom reduction observed during the first two weeks. The CES‐D scores remained significantly lower than baseline at week 8, four weeks after treatment ended. Subjective health perception was significantly associated with baseline depression severity (Wald χ^2^ = 26.41, *p* < 0.001), and its interaction with time was also significant (Wald χ^2^ = 320.18, *p* < 0.001). Participants with poorer perceived health (scores 4–5) experienced greater reductions in depressive symptoms than those with more favorable perceptions (scores 1–2).

**Conclusion**: Home‐based tDCS was feasible and associated with clinically meaningful improvements in depressive symptoms among perinatal women. Those who initially perceived their health more negatively achieved a greater response, suggesting that subjective health perception may serve as a useful moderator and potential marker to inform personalized treatment strategies.

**10:30 AM – 12:00 PM**

## 805 | **Natural** History **and Outcomes of Prenatally Diagnosed Cephaloceles: A Systematic Review and Meta‐Analysis**

Hiba J. Mustafa^1^; Işıl Ayhan^2^; Faezeh Aghajani^3^; Ella Boardley^4^; Alireza A. Shamshirsaz^5^; Asma Khalil^6^

^1^Division of Maternal‐Fetal Medicine, Indiana University School of Medicine, Indianapolis, IN; ^2^Division of Maternal‐Fetal Medicine, Antalya City Hospital, Antalya, Antalya; ^3^Boston Children's Hospital, Boston, MA; ^4^Indiana University School of Medicine, Indiana University School of Medicine, IN; ^5^Boston Children's Hospital, Harvard Medical School, Boston, MA; ^6^Fetal Medicine Unit, St George's University Hospitals NHS Foundation Trust, LONDON, England

**Objective**: To systematically review and analyze the natural history and outcomes of prenatally diagnosed cephaloceles.

**Study Design**: PubMed, Cochrane, Scopus, and Web of Science were searched systematically from inception until March 2025. The primary outcome was survival, and secondary included cephalocele‐related morbidity.

**Results**: Thirty‐one studies comprising 340 prenatally diagnosed cephaloceles were included in the review. Studies reporting fewer than 3 cases were excluded from the meta‐analysis part. Pregnancy outcomes were as follows: termination in 36.5% (124/340), intrauterine fetal demise in 4.4% (15/340), and live birth in 47.9% (163/340). Meta‐analysis was conducted on 323 cases. The mean gestational age at diagnosis was 18.4 weeks, indicating most cases were detected in the second trimester. The majority of cephaloceles were occipital (89.3%), with few located in the parietal (3.5%), frontal (3.9%), or other regions. Most lesions were encephaloceles (95.9%), with meningoceles comprising only 4.1%. Genetic testing was performed in 96.2% of cases, revealing abnormalities in 7.8%. Associated structural anomalies were present in 24.4% of fetuses. Among liveborn infants, 96.7% survived until surgery, and 95.3% underwent surgical repair. Postnatal mortality was reported in 7.8% of cases. Neurodevelopmental impairment occurred in 44.5% of children (Figure 1), while 22.8% developed hydrocephalus requiring ventriculoperitoneal shunting. Epilepsy was observed in 9.2% of cases. 93.3% of the liveborn infants demonstrated some level of ambulation. Only 18 cases from two studies underwent fetal surgery.

**Conclusion**: Prenatally diagnosed cephaloceles are associated with frequent pregnancy termination and significant morbidity among survivors. While postnatal surgical repair is feasible for most liveborn and ambulatory potential is high, nearly half experience neurodevelopmental impairment, and many require further interventions such as ventriculoperitoneal shunting. Preliminary data on fetal surgery are promising but limited by small sample sizes. Further research is needed to guide counseling and management.

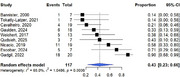

**10:30 AM – 12:00 PM**

## 806 | Tranexamic **Acid to Prevent Blood Loss in Women with Low‐Risk of Postpartum Hemorrhage**

Hugo Madar^1^; Catherine Deneux‐Tharaux^2^; Hanane Bouchghoul^1^; Aurelien Seco^2^; Alizee Froeliger^1^; Loïc Sentilhes^1^

^1^Bordeaux University Hospital, Bordeaux Universitary Hospital, Aquitaine; ^2^Université Paris Cité Institut Santé Des Femmes, Centre for Research in Epidemiology and Statistics (CRESS) U1153, Obstetrical Perinatal and Pediatric Epidemiology Research Team (Epopé), INSERM, INRAE, Paris, France., Paris, Ile‐de‐France

**Objective**: Prophylactic tranexamic acid (TXA) has been shown to reduce postpartum hemorrhage (PPH) in certain settings. However, its effectiveness may vary according to the baseline risk of PPH and remains underexplored in women with no identifiable risk factor for PPH. A recent meta‐analysis (Yunas et al., *Lancet* 2025) identified updated risk factors for PPH which could modify the effectiveness of TXA. Therefore, we reanalyzed data from the TRAAP trial to compare the effect of TXA versus placebo to prevent PPH in women with low risk of PPH according to these updated PPH risk factors.

**Study Design**: We included women from the modified intention‐to‐treat population of the multicenter, double‐blind, randomized controlled TRAAP trial who underwent vaginal delivery of a singleton fetus and received TXA or placebo in addition to prophylactic oxytocin. We excluded women with one or more risk factors for PPH, as identified in the published meta‐analysis: Black or Asian ethnicity, history of PPH, body mass index ≥25 kg/m^2^, grand multiparous, gestational diabetes, thrombocytopenia, anemia, induction of labor, instrumental delivery, neonatal birthweight ≥ 4000g, among others. The primary outcome was PPH, defined as blood loss ≥500 mL measured with a calibrated collector bag.

**Results**: Among the 3891 women randomized in the TRAAP trial, 1308 (33.6%) met the criteria for low‐risk of PPH and were included in this analysis (660 received TXA and 648 received placebo). Baseline characteristics were similar (Table 1). PPH occurred in 42 of 656 women (6.4%) in the TXA group and 40 of 640 (6.3%) in the placebo group (risk ratio, 1.02; 95% CI, 0.67–1.56) (Table 2). No significant differences were observed in secondary outcomes, including blood loss ≥1000 mL, clinically assessed significant hemorrhage, use of additional uterotonics, total estimated blood loss, postpartum transfusion, or hemoglobin/hematocrit changes (all *p* > .05) (Table 2).

**Conclusion**: Among women with low‐risk of PPH who received prophylactic oxytocin after vaginal delivery, prophylactic TXA did not reduce the incidence of PPH.

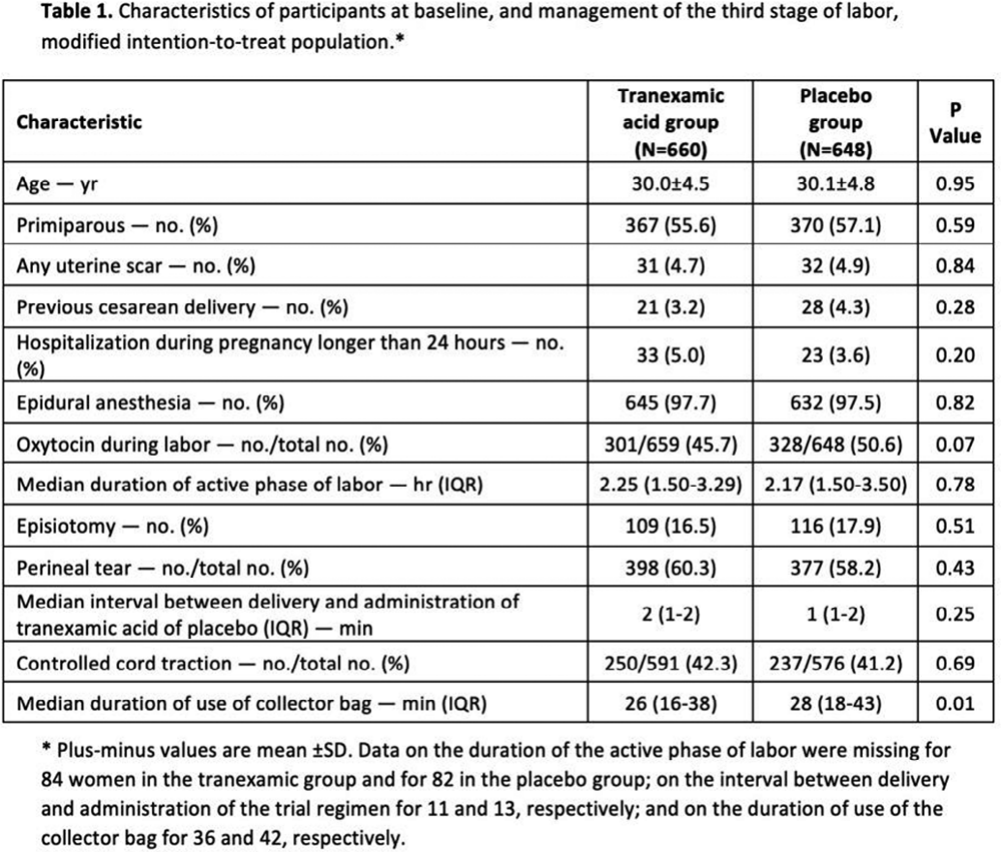

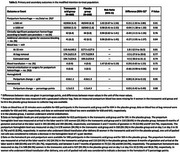

**10:30 AM – 12:00 PM**

## 807 | **Vaginal** Delivery **Outcomes in Nulliparous Women ≥40 Years With High Birth Weight Fetuses (≥3750 g)**

Ina Ryvkin^1^; Efrat Shekel^1^; Renana Dimant^1^; Tehilla Stamler‐Yakobi^1^; Snir Tamari^2^; Naama Lessans^2^; Doron Kabiri^1^

^1^Hadassah Medical Center and Hebrew University of Jerusalem, Jerusalem, Israel, Hadassah Medical Center and Hebrew University of Jerusalem, Jerusalem, Israel, Yerushalayim; ^2^Hadassah Medical Center and Hebrew University of Jerusalem, Jerusalem, Israel, Hadassah Medical Center and Hebrew University of Jerusalem, Jerusalem, Israel, Yerushalayim

**Objective**: To assess the probability of successful vaginal delivery and identify determinants of delivery mode in nulliparous women aged ≥40 years carrying fetuses with birth weight ≥3750 grams.

**Study Design**: Retrospective cohort study. From a cohort exceeding 250, 000 deliveries between 2003 and 2025, 12, 818 involved women aged ≥40 years. Of these, 76 were nulliparous with fetuses weighing ≥3750 grams. Thirty‐seven women underwent planned cesarean delivery, while 39 attempted vaginal delivery. Outcomes were compared between women with successful vaginal delivery (*n* = 19) and those requiring unplanned cesarean section (*n* = 20). Variables analyzed included maternal age, prolonged second stage of labor, use of neuraxial anesthesia, fetal sex, and neonatal complications. Statistical analysis employed Mann‐Whitney U test for continuous variables and Fisher's exact test or Chi‐square test for categorical variables.

**Results**: As shown in Figure 1 Spontaneous vaginal delivery occurred in 11 cases (28.2%), instrumental vaginal delivery in 8 cases (20.5%), and unplanned cesarean section in 20 cases (51.3%). The overall vaginal delivery success rate was 48.7% (19/39). The two groups were comparable in baseline characteristics (Table 1), with the only significant difference being the use of neuraxial anesthesia (*p* = 0.046), probably attributable to standard practice rather than a causal relationship. No definitive predictors of vaginal delivery success were identified. Neonatal outcomes were favorable in both groups, with low rates of NICU admission.

**Conclusion**: In nulliparous women aged ≥40 years carrying fetuses with birth weight ≥3750 grams, the vaginal delivery success rate was 48.7%. These findings suggest that a trial of labor may be a reasonable option under careful intrapartum monitoring.

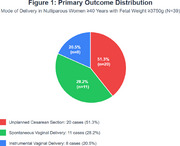

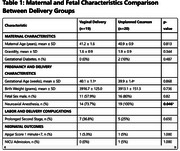

**10:30 AM – 12:00 PM**

## 808 | Prediction **Model for Intrapartum Cesarean Delivery in Pregnancies Complicated by Sonographic Small for Gestational Age**

Itamar Gilboa^1^; Daniel Gabbai^1^; Dana Englander^1^; Anat Lavie^1^; Yariv Yogev^2^; Emmanuel Attali^1^

^1^Lis Hospital for Women's Health, Tel Aviv Sourasky Medical Center, Lis Hospital for Women's Health, Tel Aviv Sourasky Medical Center, Tel Aviv; ^2^Lis Hospital for Women's Health, Tel Aviv Sourasky Medical Center Gray Faculty of Medicine, Tel Aviv University, Israel, Lis Hospital for Women's Health, Tel Aviv Sourasky Medical Center, Tel Aviv

**Objective**: To identify risk factors and develop a predictive model for intrapartum cesarean delivery (CD) in women carrying small for gestational age (SGA) fetuses.

**Study Design**: This retrospective cohort study included singleton pregnancies complicated by SGA defined as an estimated fetal weight <10th percentile at ≥36weeks’ gestation, delivered between 2013–2024 at a single tertiary center. Exclusion criteria were elective or prior CD and non‐viable fetuses. Only CDs performed during labor due to maternal or fetal indications were included. Maternal characteristics, ultrasound findings (including estimated fetal weight [EFW], abdominal circumference [AC] and Doppler), and labor variables were compared between women who delivered vaginally and those who underwent intrapartum CD. Univariate and multivariable logistic regression identified independent predictors, and a predictive model was developed. Model performance was assessed using a receiver operating characteristic (ROC) curve.

**Results**:

1. A total of 2, 035 women met the inclusion criteria, of whom 62 (8.0%) underwent intrapartum CD. Among intrapartum CDs, the most common indication was non‐reassuring fetal heart rate (73.0%).
2. Independent risk factors for intrapartum CD included induction of labor (OR 5.87, 95% CI 2.94–11.70), nulliparity (OR 5.50, 95% CI 2.62–11.54), preeclampsia (OR 3.54, 95% CI 1.41–8.93), maternal age >35 years (OR 2.91, 95% CI 1.64–5.16), gestational age between 36–37weeks (OR 2.81, 95% CI 1.26–6.25),
3. Epidural anesthesia (OR 0.25, 95% CI 0.14–0.45) was found to be protective for intrapartum CD
4. For each percentile increase in fetal abdominal circumference (AC) beyond the 1^st^ percentile, the risk of intrapartum CD decreased (OR 0.62, 95% CI 0.39–0.99, *p* = 0.049).
5. The predictive model demonstrated good performance with an AUC of 0.792 (95% CI 0.752–0.831; *p* < 0.001). A cutoff score of 6.5 yielded sensitivity of 74% and specificity of 67%.

**Conclusion**: A prediction model for intrapartum CD in SGA pregnancies may help clinicians identify high‐risk patients and support counseling and delivery planning.

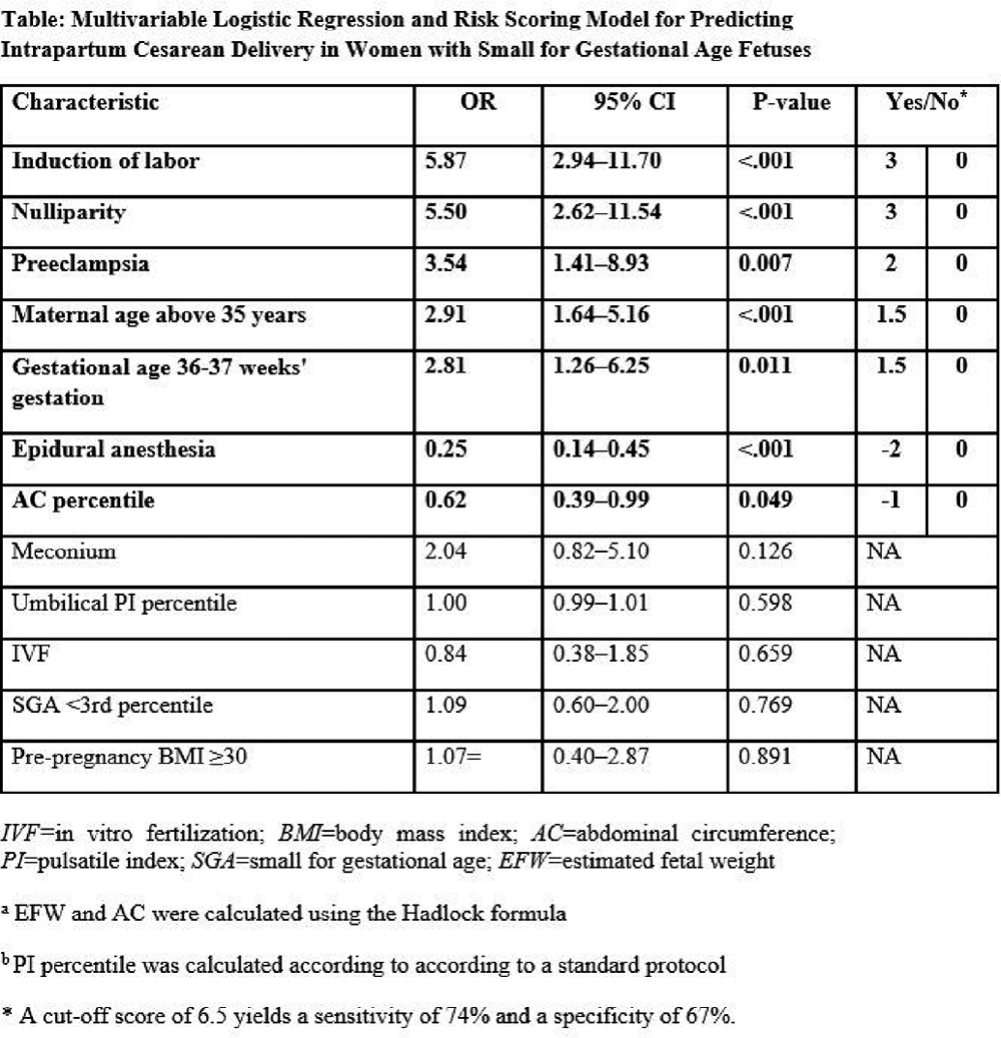

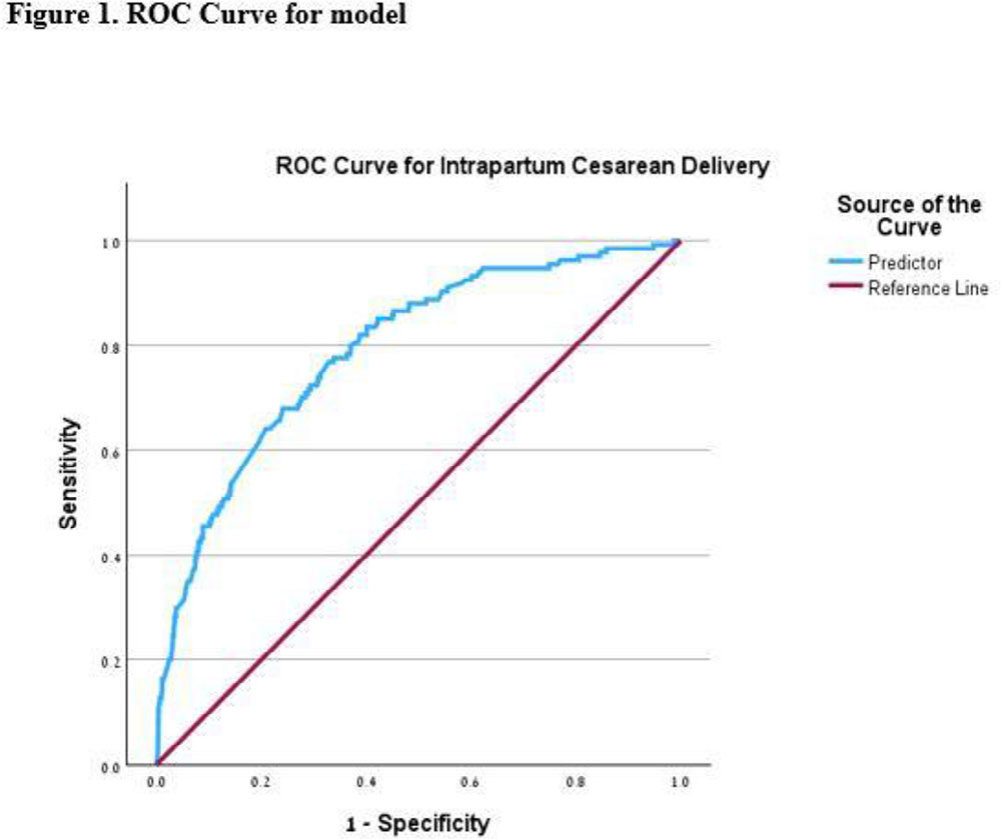

**10:30 AM – 12:00 PM**

## 809 | Pregnancy **Outcomes of Successive Higher‐Order Cerclages**

Ivie Odiase^1^; Andrei Rebarber^2^; Morgan Steelman^3^; Nathan S. Fox^1^

^1^Icahn School of Medicine, Mount Sinai West, New York, NY; ^2^Icahn School of Medicine, Mount Sinai West, Mount Sinai West, NY; ^3^Icahn School of Medicine at Mount Sinai Hospital, Icahn School of Medicine at Mount Sinai Hospital, NY

**Objective**: There are limited data on pregnant patients who undergo successive higher‐order cerclage with increasing parity. Our objective was to review pregnancy outcomes for patients who underwent higher‐order cerclages, and to estimate the association between increasing cerclage order and pregnancy outcomes.

**Study Design**: Retrospective cohort study of patients who underwent cerclage placement in a single maternal fetal medicine practice from 2005–2025. McDonald or Shirodkar cerclage were used at the surgeon's discretion, however, over 90% of cerclages performed were Shirodkar. 5mm Mersiline suture was used in all cases. We included women undergoing their third or higher order cerclage. We compared baseline characteristics and pregnancy outcomes for patients undergoing their 3^rd^, 4^th^, and 5^th^ or higher order cerclage. One way ANOVA and chi‐square for trend were used for analysis.

**Results**: 118 patients met inclusion criteria. Delivery data were available for 93% of patients (some are still pregnant, some delivered elsewhere). Data are shown in Table 1. Maternal age increased and gestational age decreased with successive cerclages, as expected. Otherwise, baseline characteristics were similar between the groups. There was a high incidence of cervical lacerations found, which increased with successive cerclages. Complication rates were low. There were very low rates of pregnancy loss prior to 24 weeks (2.1%, 3.4%, 2.6%, *p* = 0.873) and did not increase across the groups. The rates of delivery at 34+ weeks (91.3%, 89.3%, 86.8%, *p* = 0.513) and 37+ weeks (77.8%, 77.8%, 63.2%, *p* = 0.146) were high and did not decrease across the groups.

**Conclusion**: For patients undergoing higher order cerclages, aside from a high incidence of cervical lacerations noted, there is a very low rate of complications and pregnancy loss, and high rates of delivery at or near term. These results do not appear to change with increasing cerclage order. Patients should not be discouraged from pregnancy solely due to multiple prior cerclages. Our data may be limited to those undergoing Shirodkar cerclage by experienced surgeons.

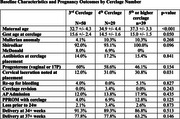

**10:30 AM – 12:00 PM**

## 810 | **The** PROMMO **(Prelabor Rupture of Membranes: Misoprostol vs Oxytocin) Study: A Randomized Control Trial**

Jacquelyn H. Adams^1^; Amy Godecker^2^; Kara K. Hoppe^2^

^1^University of Wisconsin School of Medicine and Public Health, Madison, WI; ^2^University of Wisconsin School of Medicine and Public Health, Department of Obstetrics and Gynecology, Madison, WI

**Objective**: Prelabor rupture of membranes (PROM) is common, yet optimal induction methods remain debated. We sought to compare intravenous oxytocin (OT) to oral misoprostol (MP) for induction of labor in PROM.

**Study Design**: This is a non‐blinded, block‐randomized control trial in a single institution from 08/2020 to 05/2024. Patients were randomized to either oral MP or intravenous OT. Inclusion criteria included confirmed PROM, an unfavorable modified Bishop score (<7), gestational age 34w0d and 41w6d, and ability to consent in Spanish or English. The primary outcome was time from first induction agent to vaginal delivery. Secondary outcomes included Cesarean delivery, IAI, and postpartum hemorrhage (PPH). Statistical analysis included chi‐square or Fisher's exact tests and t‐test or Wilcoxon‐Rank Sum as appropriate.

**Results**: We enrolled 134 of our planned 200 patients due to inability to recruit after the adoption of misoprostol as standard of care in our institution. MP patients received a median of 2 doses; 72% required oxytocin after ripening. Median time to oxytocin initiation in the MP group was 428 min [IQR 289, 560]. Study arms did not differ in baseline characteristics. The difference in time from first induction agent to vaginal delivery between oral MP 637 min [IQR 457, 1074.5] and OT 783 min [598, 1275] was not significant (*p* =  .208). There was, however, a significant difference in the length of the second stage (38 min [18.5, 91] MP vs 68 [29.5, 144.5] OT, *p* = 0.034). There was no difference in immediate PPH (12.3% v. 5.8% *p* = 0.233), Cesarean delivery (20% v. 24.6% *p* = 0.689), IAI (1.5% vs 7.3% *p* = 0.209), or NICU admission (24.6% v. 23.2% *p* = 0.846) for MP vs OT, respectively.

**Conclusion**: This study demonstrated a trend toward a shorter first stage and significantly shorter second stage of labor for patients with PROM treated with MP compared to OT. There was no difference in adverse outcomes such as PPH or IAI. Oral misoprostol should be considered for labor induction in patients presenting with an unfavorable cervix and PROM.

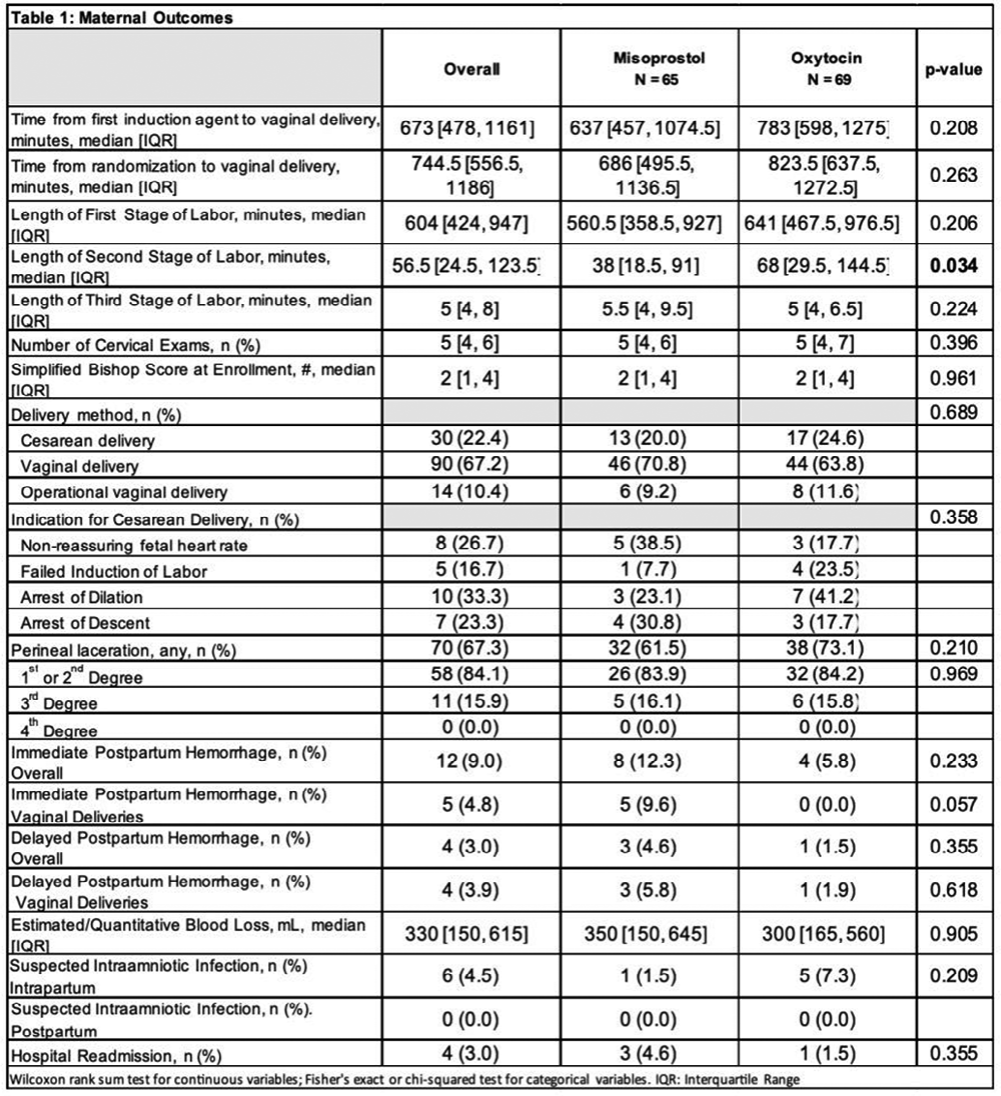

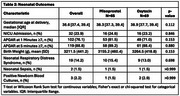

**10:30 AM – 12:00 PM**

## 811 | **Severe** Neonatal **Morbidity Among Jehovah's Witnesses and Others Declining Blood Products**

Jamie Green; Sarah H. Abelman; Frank I. Jackson; Oladunni Ogundipe; Luis A. Bracero; Matthew J. Blitz

Northwell, New Hyde Park, NY

**Objective**: To evaluate the incidence of severe neonatal morbidity (SNM) among Jehovah's Witnesses and other patients who decline blood products compared to the general obstetric population. These patients may be at increased risk for adverse neonatal outcomes due to deviations from routine obstetric management.

**Study Design**: This retrospective cross‐sectional study included all deliveries at two regional perinatal centers in a large New York health system from 2019–2024. Exclusion criteria were fetal demise and multiple gestation. The primary exposure was blood product refusal, documented at admission via a standardized intake question for all patients. The primary outcome was SNM, a composite neonatal adverse outcome indicator which includes diagnoses and procedures that signify life‐threatening neonatal complications. Multivariable logistic regression modeled the association between blood product refusal and SNM, adjusting for race and ethnicity group, public health insurance, multiparity, obstetric comorbidity index (OB‐CMI) score, gestational age at delivery, fetal growth restriction, and newborn sex. SNM is strongly influenced by gestational age; as nearly all deliveries before 32 weeks have some form of SNM, analysis was restricted to deliveries at ≥32 weeks with gestational age included as a covariate.

**Results**: A total of 78, 899 pregnancies were included. Blood product refusal was documented in 589 cases. Non‐Hispanic White patients constituted the largest race and ethnicity group (42.6%), followed by Asian or Pacific Islander (19.3%), Non‐Hispanic Black (14.0%), and Hispanic patients (12.6%). SNM occurred in 8.3% of patients declining blood products compared to 6.7% in the general obstetric population which was not a statistically significant difference (aOR 1.06; 95% CI 0.76–1.45; *p* = 0.71).

**Conclusion**: There was no difference in SNM between patients who declined blood products and the general obstetric population. Further research is needed to assess potential neonatal risks and inform management in this population.

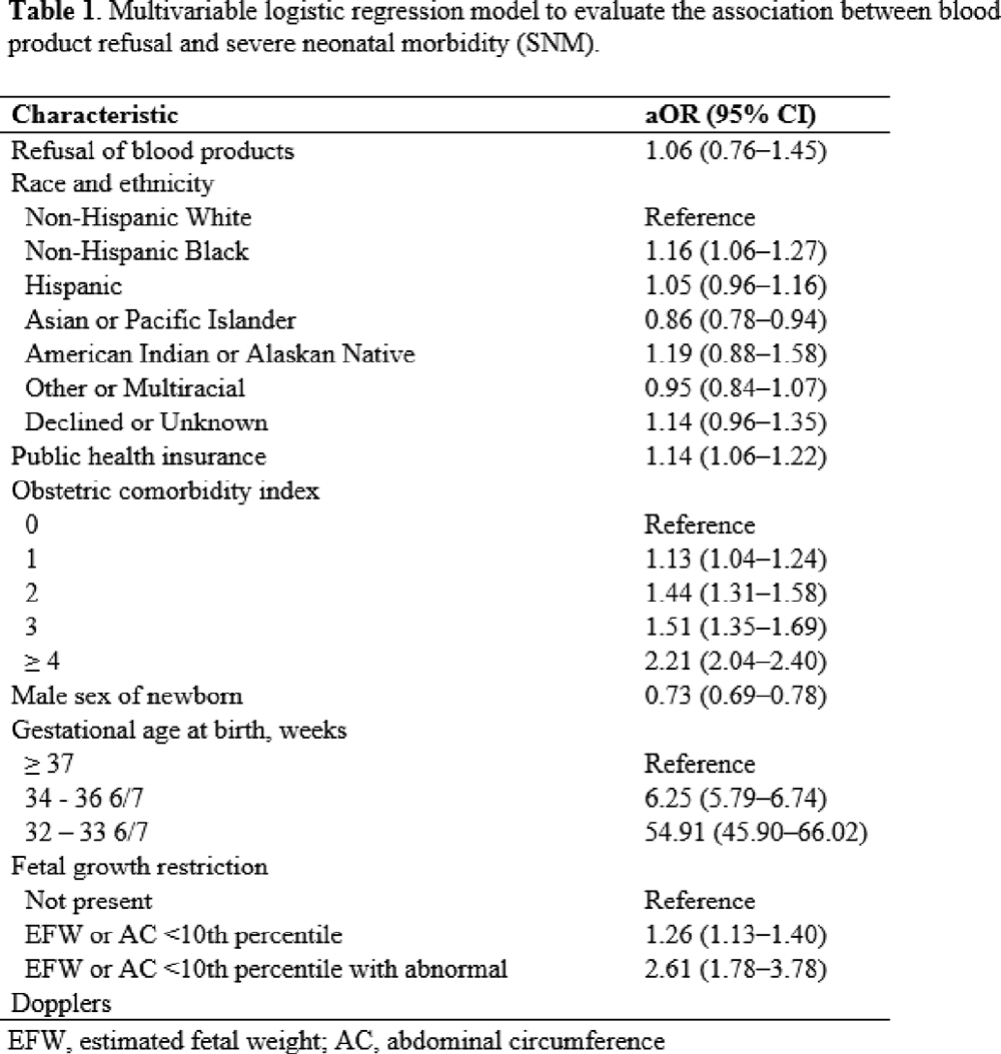

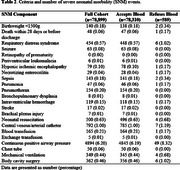

**10:30 AM – 12:00 PM**

## 812 | **Triplet‐**Specific **Birthweight Reference and its Utility in Predicting Neonatal Outcomes: A Retrospective Cohort Study**

Jeesun Lee^1^; Joon Hyung Lee^2^; Hee Young Cho, N/A^3^; Seung Mi Lee^4^; Chan‐Wook Park^5^; Joong Shin Park^5^; Jong Kwan Jun^6^

^1^Gyeongsang National University Hospital, Jinju‐si, Kyongsang‐namdo; ^2^The Nature Obstetrics and Gynecology, Pyeongtaek, Kyonggi‐do; ^3^Obstetrics and Gynecology, Seoul National University College of Medicine, Seoul, Kyonggi‐do; ^4^Department of Obstetrics and Gynecology, Seoul National University College of Medicine, Seoul, Seoul‐t'ukpyolsi; ^5^Department of Obstetrics and Gynecology, Seoul National University Hospital, Seoul National University College of Medicine, Seoul, Republic of Korea, Seoul, Seoul‐t'ukpyolsi; ^6^Department of Obstetrics and Gynecology, Ewha Womans University, Seoul, Seoul‐t'ukpyolsi

**Objective**: We tried to develop a triplet‐specific birthweight reference and evaluate its predictive accuracy for adverse neonatal outcomes compared with singleton and twin references.

**Study Design**: This retrospective cohort included 491 triplet trios delivered at a tertiary center (Nov 1997–Feb 2024). Birthweights of trichorionic and dichorionic triplets were used to construct gestational age–specific percentiles (3rd–97th) via the lambda‐mu‐sigma method. Small‐for‐gestational‐age (SGA) was defined < 10th percentile using singleton, twin, or triplet references. Unlike most growth references based on ultrasonographic estimated fetal weight (EFW), we used actual birthweight because EFW is less reliable in triplet pregnancies than in singleton and twin pregnancies due to technical limitations and labeling errors. Composite adverse neonatal outcome (neonatal death, respiratory distress syndrome, bronchopulmonary dysplasia, persistent ductus arteriosus, pulmonary hypertension, intraventricular hemorrhage, periventricular leukomalacia, retinopathy of prematurity, necrotizing enterocolitis, or early‐onset sepsis) was compared across references.

**Results**: Monochorionic triplets had lower birthweights than di‐ or trichorionic triplets (*p* =  .004). No significant birthweight difference was observed between male and female triplets (*p* =  .782). Triplet growth diverged from twins and singletons after 28 weeks. Using singleton, twin, and triplet references, 40.5%, 22.2%, and 8.3% of neonates were SGA, respectively. Neonates classified as SGA by singleton or twin reference, but non‐SGA by triplet reference had no increased risk of adverse outcomes compared with non‐SGA (singleton aOR 1.25, 95% CI 0.86–1.82; twin aOR 0.81, 95% CI 0.51–1.27). But, SGA by triplet reference showed significantly increased risk for adverse neonatal outcome (aOR 2.15, 95% CI 1.43–3.24).

**Conclusion**: The triplet‐specific birthweight reference reduces overdiagnosis of small but healthy neonates and more accurately identifies those at genuine risk for adverse outcomes, supporting its clinical utility in guiding optimal management of triplet pregnancies.

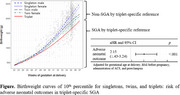

**10:30 AM – 12:00 PM**

## 813 | **Factors** Associated **With Providers’ Perspectives on Mode of Delivery in Rural Settings**

Heidi Preis^1^; Maria Fisher^2^; Emily Via^2^; Rakiya Watts^2^; Mayi Gnofam^3^; Jennifer Choi^4^

^1^Stony Brook University, Stony Brook, NY; ^2^Stony Brook University Hospital, Stony Brook, NY; ^3^Stony Brook University, New York, NY; ^4^Stony Brook University Hospital, Commack, NY

**Objective**: Epidemiological data suggest women traveling long distances to a delivery hospital are more likely to undergo nonmedically indicated induction of labor (IOL) and less likely to access trial of labor after cesarean (TOLAC), which may increase maternal morbidity and mortality. However, provider‐driven contributors to these practices remain unknown.

**Study Design**: Cross‐sectional online survey of obstetric providers in New York State (9/2024–3/2025). Outcomes were perspective about distance being an indication for IOL and the percentage of time they would recommend TOLAC (vs. repeat cesarean) for patients 50 miles from a hospital. Predictors were profession, litigation concerns, frequency of recommending TOLAC in medical scenarios, perspectives regarding medical scenarios being an indication for IOL, and institutional IOL or TOLAC rates. Analysis included bivariate, generalized linear models, and mediation analysis.

**Results**: Among the 107 participants, 61.3% were physicians and 32.7% were midwives. Physicians more often considered distance > 50 miles to be an indication for IOL in of itself (45.1%) compared to midwives (8.6%) (*p* < .001) and physicians less frequently recommended TOLAC compared to midwives (z = 3.49, *p* < .001). The IOL and TOLAC models were well‐fitting (Table 1). Perspectives on IOL in medical scenarios (aOR = 1.3) was the only significant predictor for perspectives on IOL in rural settings. Frequency of TOLAC recommendations in medical scenarios (aOR = 10.4) and litigation concerns (aOR = 1.8) were significant predictors of the TOLAC recommendation in rural settings. Medical scenario perspectives fully mediated the association between profession and TOLAC recommendations and partially mediated perspectives on IOL in rural settings (Figure 1).

**Conclusion**: Perspectives on delivery mode for rural patients are strongly influenced by profession, affecting overall views on IOL and TOLAC in medical scenarios. Litigation concerns also impacts TOLAC access. Shifting practice perspectives and expanding midwifery services could improve access to safe delivery options for rural patients.

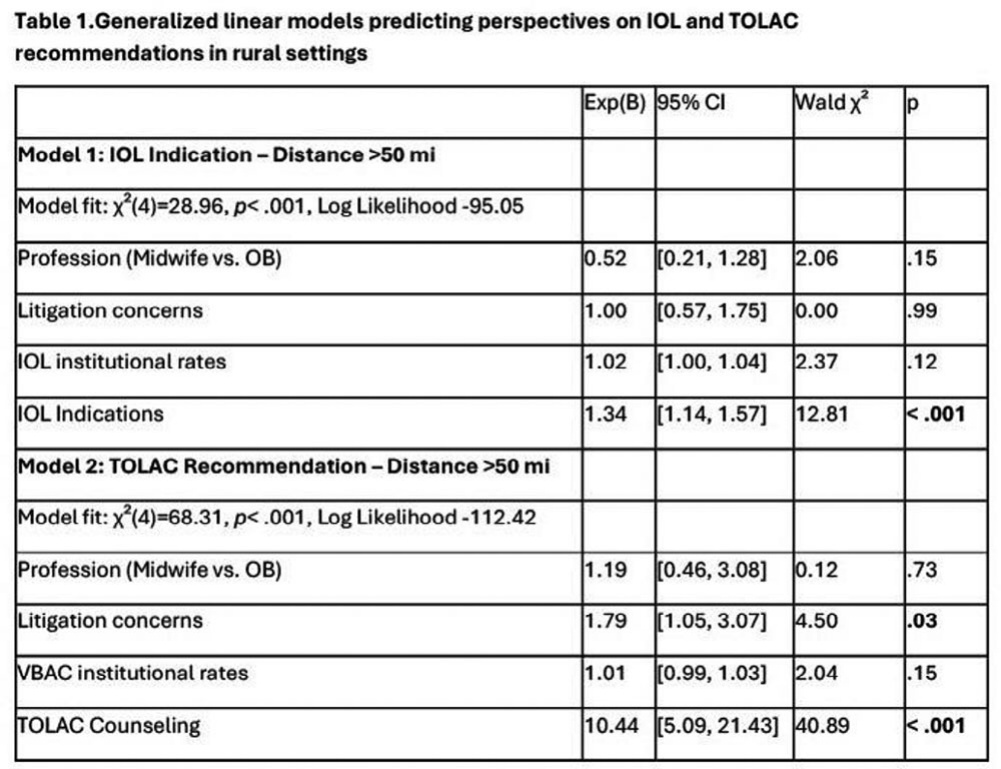

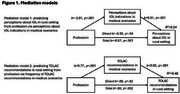

**10:30 AM – 12:00 PM**

## 814 | **Impact of AI Support on Completion of Fetal Cardiac Screening in an MFM Ultrasound Center**

Jennifer Lam‐Rachlin^1^; Andrei Rebarber^2^; Clementine Roth^3^; Luciana Vieira^4^; Simi Gupta^4^; Shari Gelber^1^; Jessica Spiegelman^4^; Steven R. Inglis^4^; Celia Muoser^4^; Xiangna Tang^4^; Samantha Do^4^; Nathan S. Fox^4^

^1^Icahn School of Medicine at Mount Sinai, Icahn School of Medicine at Mount Sinai, NY; ^2^Icahn School of Medicine, Mount Sinai West, Mount Sinai West, NY; ^3^Carnegie Imaging for Women PLLC, New York, NY; ^4^Icahn School of Medicine, Mount Sinai West, New York, NY

**Objective**: High quality fetal heart evaluations are essential for accurately identifying prenatal cases that may be at risk of severe congenital heart defects (CHDs).

We aim to evaluate the impact of an AI software developed by BrightHeart on the quality of prenatal ultrasound fetal cardiac screening by measuring its impact on view acquisition completeness and the rate of exams flagged for suspicious findings.

**Study Design**: A retrospective analysis was conducted on 1449 fetal anatomy exams performed at 18–24 weeks gestation at a single center across three phases: baseline (no AI), after providing sonographers with real time AI tool for assessing view completeness (defined as 2 seconds of 4CH, LVOT and RVOT views in grayscale ultrasound cines), and after additionally providing an enhanced AI tool that flags 8 findings suspicious for CHDs. Primary outcomes were the proportion of scans meeting standard cardiac view completeness criteria and the rate of exams without cardiac abnormalities flagged for suspicious findings.

**Results**: At baseline, 59.5% (95% CI: 54.4–64.3) of exams met view completeness criteria. Two months after implementation of the AI, this increased to 91.0% (95% CI: 88.5–93.1) (*p* < 0.01), indicating a significant improvement in image acquisition (Figure 1).

When AI for suspicious findings was not visible, 4.8% (95% CI: 3.5–6.5) of exams without cardiac abnormalities were flagged for suspicious findings. Following implementation of the AI tool, this rate declined to 1.5% (95% CI: 0.7–3.3) (Figure 2), corresponding to an alarm rate reduction of 68% (*p* < 0.01).

Mean scan duration was not significantly different between the three phases.

**Conclusion**: Access to real time AI software during image acquisition was associated with improved view completeness and reduced false‐positive screening results. This may reflect enhanced sonographic quality and diagnostic confidence by both sonographer and physician reviewers. These results support the integration of AI tools into prenatal ultrasound workflows as they may reduce unnecessary referrals, and elevate the quality and efficiency of fetal cardiac screening.

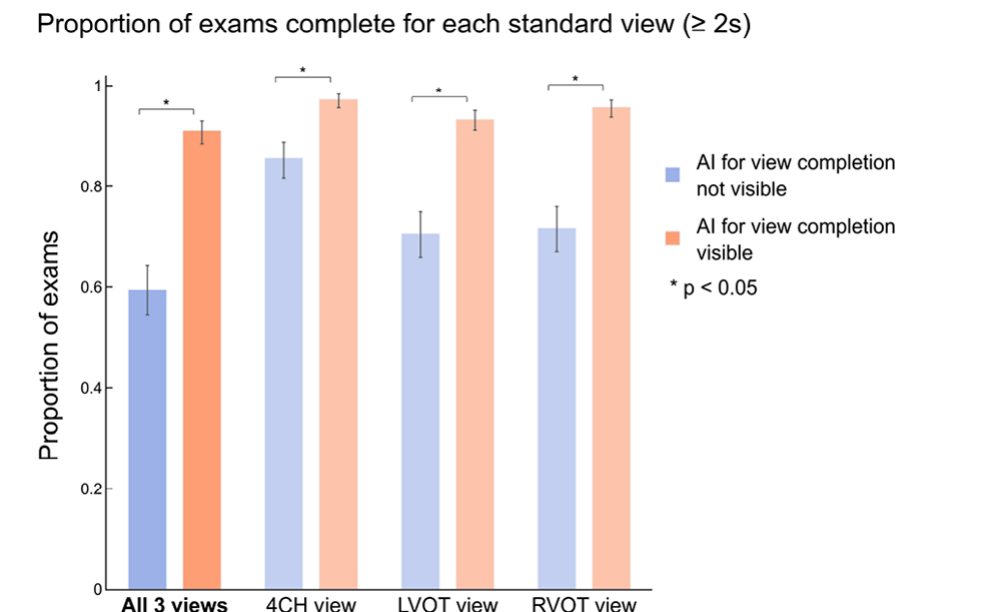

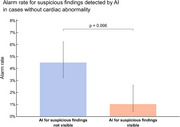

**10:30 AM – 12:00 PM**

## 815 | Development **of a Decision Aid on Infant Feeding for Birthing People Living With HIV**

Jennifer Qin^1^; Celeste Coler^2^; Faith Beers^1^; Isabelle L. Crary^3^; Ishita Kohli^4^; Lyndsey Benson^1^; Jane Hitti^1^

^1^University of Washington, Seattle, WA; ^2^University of Washington School of Medicine, Seattle, WA; ^3^University of Washington Department of OB/GYN, Seattle, WA; ^4^University of Washington, University of Washington, WA

**Objective**: With breastfeeding HIV transmission rates less than 1% for virally suppressed birthing people living with HIV (PLHIV), the United States Department of Health and Human Services now recommends patient‐centered counseling for PLHIV on infant feeding options. A prior qualitative study noted several barriers for birthing PLHIV including limited information, lack of regard for preferences, and a scarcity of research and clinical expertise. Thus, there is a need for effective, shared decision‐making regarding infant feeding for PLHIV.

The study objectives were to identify factors influencing infant feeding decisions and to design a patient‐facing decision aid for PLHIV.

**Study Design**: This qualitative cross‐sectional study engaged a multidisciplinary stakeholder team including birthing PLHIV, prenatal clinic staff, and community advocates. During 60–120 minute in‐depth semi structured interviews, seven participants shared their experiences with counseling and infant feeding decisions and provided input on a prototype decision aid for infant feeding. We used an inductive thematic analysis approach to generate a codebook and identify, analyze, and report themes in ATLAS.ti. Thematic saturation was achieved.

**Results**: Three key themes were identified: (1) how PLHIV interpret the <1% risk of HIV transmission varies considerably; (2) infant feeding decisions are shaped by complex, individualized values; and (3) while partners and family, community organizations, and multidisciplinary care teams can significantly impact the decision‐making process, PLHIV autonomy must be respected. Stakeholder input was incorporated into a prototype decision aid, which included an interactive values clarification tool with a summary of the participant's reflections.

**Conclusion**: Decision making around infant feeding for birthing PLHIV is a highly personal process that can be significantly impacted by care providers and support people. These results informed the design of a decision aid intended to support shared decision making on infant feeding choices for PLHIV. Next steps are to field test the decision aid.

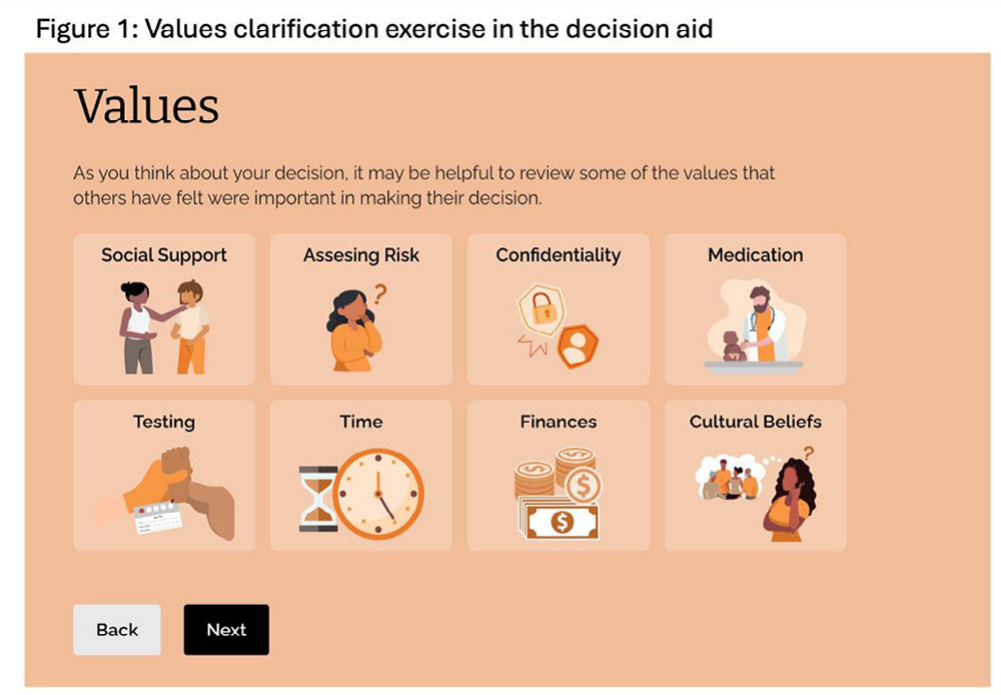

**10:30 AM – 12:00 PM**

## 816 | **AI** Enhanced **Prenatal Ultrasound Improves Detection of Congenital Heart Defects in Patients With High‐BMI**

Jessica Spiegelman^1^; Jennifer Lam‐Rachlin^2^; Rajesh Punn^3^; Sarina K. K. Behera^4^; Miwa Geiger^5^; Matthias Lachaud^6^; Sara Garmel^7^; Matthew K. Janssen^8^; Kendra R. Sylvester‐Armstrong^9^; Mia Heiligenstein^10^; Nathan S. Fox^1^; Andrei Rebarber^11^; Greggory R. R. Devore^12^; Carolyn M. Zelop^13^; Roger Bessis^14^; Marilyne Levy^15^; Bertrand Stos^15^; Alisa Arunamata^3^

^1^Icahn School of Medicine, Mount Sinai West, New York, NY; ^2^Icahn School of Medicine at Mount Sinai, Icahn School of Medicine at Mount Sinai, NY; ^3^Stanford University School of Medicine, Palo Alto, CA; ^4^Palo Alto Medical Foundation, Sutter Health, Palo Alto, CA; ^5^Icahn School of Medicine at Mount Sinai Hospital, New York, NY; ^6^Univ. Grenoble Alpes, CHU Grenoble Alpes, Grenoble, Rhone‐Alpes; ^7^Michigan Perinatal Associates and Corewell Health, Dearborn, MI; ^8^Perelman School of Medicine, University of Pennsylvania, Philadelphia, PA; ^9^Perinatal Specialists of the Palm Beaches, West Palm Beach, FL; ^10^Icahn School of Medicine, Mount Sinai West, New York City, NY; ^11^Icahn School of Medicine, Mount Sinai West, Mount Sinai West, NY; ^12^The Fetal Diagnostic Center of Pasadena, Pasadena, CA; ^13^Valley Health System, Paramus, NJ; ^14^Centre d'Echographie de l'Odéon, Paris, Ile‐de‐France; ^15^UE3C – Unité d'explorations cardiologiques – Cardiopathies Congénitales, Paris, Ile‐de‐France

**Objective**: Prenatal detection of congenital heart defects (CHDs) is critical for improving neonatal outcomes, yet remains challenging particularly in patients with high body mass index (BMI), where poor acoustic windows can hinder standard ultrasound assessments. We evaluate whether an AI‐aided approach to prenatal ultrasound improves CHD detection in this population.

**Study Design**: An AI software developed by BrightHeart analyzed grayscale 2D ultrasound cines from 200 fetal exams (18–24 weeks gestation) collected from 11 centers across 2 countries. The dataset included obstetric, detailed anatomic, and fetal echocardiograms from singleton pregnancies. The AI detects 8 morphological markers associated with severe CHDs and highlights frames for assessment. Ground truth was established by a panel of expert fetal cardiologists. Fourteen physicians (OBGYNs/MFMs, 1–30+ years’ experience) reviewed each case in randomized order, both with and without AI support. ROC AUC, sensitivity and specificity of readers in identifying the presence of any finding at the examination level were evaluated.

**Results**: Overall, AI assistance significantly improved diagnostic performance, increasing ROC AUC from 0.825 (95% CI: 0.741–0.908) to 0.974 (95% CI: 0.957–0.990). Importantly, AI‐aided performance was consistent across BMI categories: ROC AUC was 0.963 (95% CI: 0.926–0.999), 0.990 (95% CI: 0.977–1.000), 0.979 (95% CI: 0.957–1.000) and 0.996 (95% CI: 0.986–1.000) in the < 25kg/m^2^, 25–30 kg/m^2^, 30–35kg/m^2^ and ≥ 35 kg/m^2^ patient BMI subgroups, respectively. Physician reading performance improved similarly in all BMI subgroups when aided by AI (Figure 1), suggesting that the tool effectively mitigates the known imaging limitations associated with elevated maternal BMI.

**Conclusion**: AI‐aided analysis of prenatal ultrasound significantly enhances the detection of CHD‐associated findings in a challenging high‐BMI population. This tool may help reduce missed or late diagnoses and enable earlier referral for fetal echocardiography, particularly in cases where imaging quality is compromised.

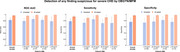

**10:30 AM – 12:00 PM**

## 817 | **Solomon Technique Increases Risk for Extreme Preterm Birth after Laser Surgery for Twin‐Twin Transfusion Syndrome**

Jimmy Espinoza^1^; Magdalena Litwińska^2^; Ramesha Papanna^3^; Anthony Johnson^1^; Sami Backley^4^; Felicia V. LeMoine^4^; Sen Zhu^1^; Dejian Lai^5^; Randy Mohtar^6^; Arlyn S. Llanes^7^; Ramen H. Chmait^8^

^1^UTHealth Houston Fetal Institute; Division of Fetal Intervention, Department of Obstetrics, Gynecology and Reproductive Sciences, McGovern Medical School at UTHealth Houston, Houston, TX; ^2^Department of Obstetrics and Gynecology, Medical University of Warsaw, Warsaw, Poland, Warsaw, Mazowieckie; ^3^University of Texas Health Science Center in Houston, McGovern Medical School, Houston, TX; ^4^Division of Fetal Intervention, Department of Obstetrics, Gynecology and Reproductive Sciences, McGovern Medical School at UTHealth Houston, Houston, TX; ^5^Department of Biostatistics, School of Public Health at UTHealth Houston, Houston, TX; ^6^Division of Maternal‐Fetal Medicine, Department of Obstetrics and Gynecology, Keck School of Medicine, University of Southern California, Los Angeles, Los Angeles, CA; ^7^Keck School of Medicine of USC, University of Southern California, Keck School of Medicine of USC, University Of Southern California, CA; ^8^UC, LA, MA

**Objective**: To explore associations between Solomonization during laser surgery and adverse pregnancy outcomes in monochorionic diamniotic (MCDA) pregnancies complicated by twin‐twin transfusion syndrome (TTTS).

**Study Design**: This retrospective cohort study included MCDA twin pregnancies complicated by TTTS that underwent laser surgery from 2006–2023 at two centers. Primary outcome was delivery <4 weeks after laser surgery. Secondary outcomes were delivery <28 weeks and placental abruption. Cases of dual fetal death and no laser technique or delivery data were excluded. Multivariable robust Poisson regression models were used to estimate relative risks (RR) for outcomes adjusted for TTTS stage, selective fetal growth restriction, cervical length < 25 mm, and premature prelabor rupture of membranes (PPROM), among other confounders. Cox regression was used to evaluate Solomonization's effect on surgery‐to‐delivery interval. A *p* < 0.0056 was used after Bonferroni correction.

**Results**: Of 1, 680 TTTS cases, 80 were excluded, with exclusions occurring equally between study groups. Adverse pregnancy outcomes were more frequent with Solomonization (Tables), which was independently associated with delivery < 4 weeks after surgery (RR: 1.98; 95% CI: 1.38–2.84; *p* < 0.001), delivery <28 weeks (RR: 1.60; 95% CI: 1.23–2.06; *p* < 0.001), placental abruption (RR: 1.91; 95% CI: 1.31–2.80; *p* = 0.001), and shorter surgery‐to‐delivery interval (hazard ratio: 1.42; 95% CI: 1.28–1.58; *p* < 0.001). The Solomon group exhibited increased laser time, higher laser energy consumption, elevated PPROM rates, earlier gestational age at delivery, and reduced 30‐day survival rate of at least one twin (Table 2). Anterior placenta, surgeon, entry technique, cannula size, fetoscope type, or amnioinfusion volume were not associated with the primary outcome.

**Conclusion**: The Solomon laser technique is associated with increased risk of delivery within four weeks after laser surgery, delivery < 28 weeks’ gestation, shorter surgery‐to‐delivery interval, and placental abruption in MCDA pregnancies complicated by TTTS.

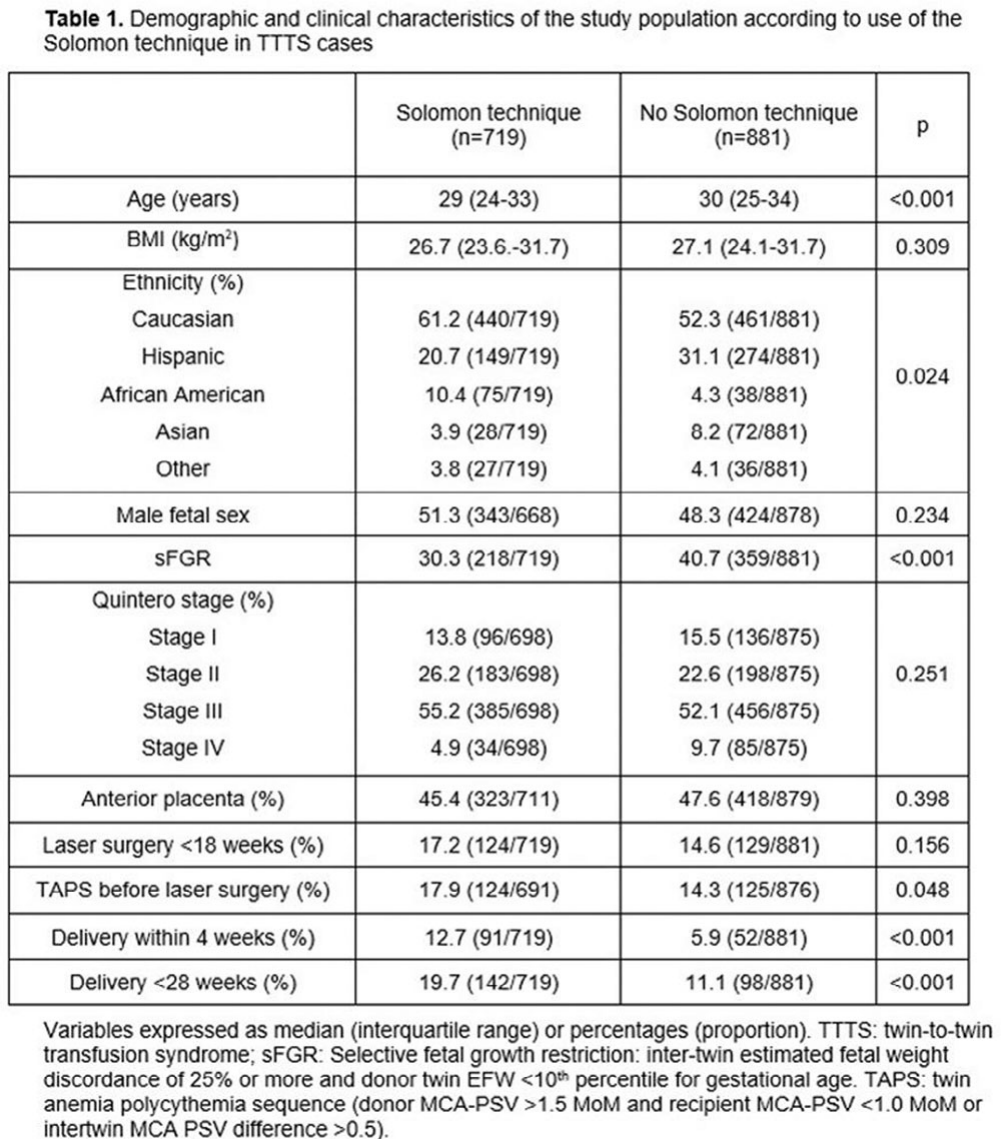

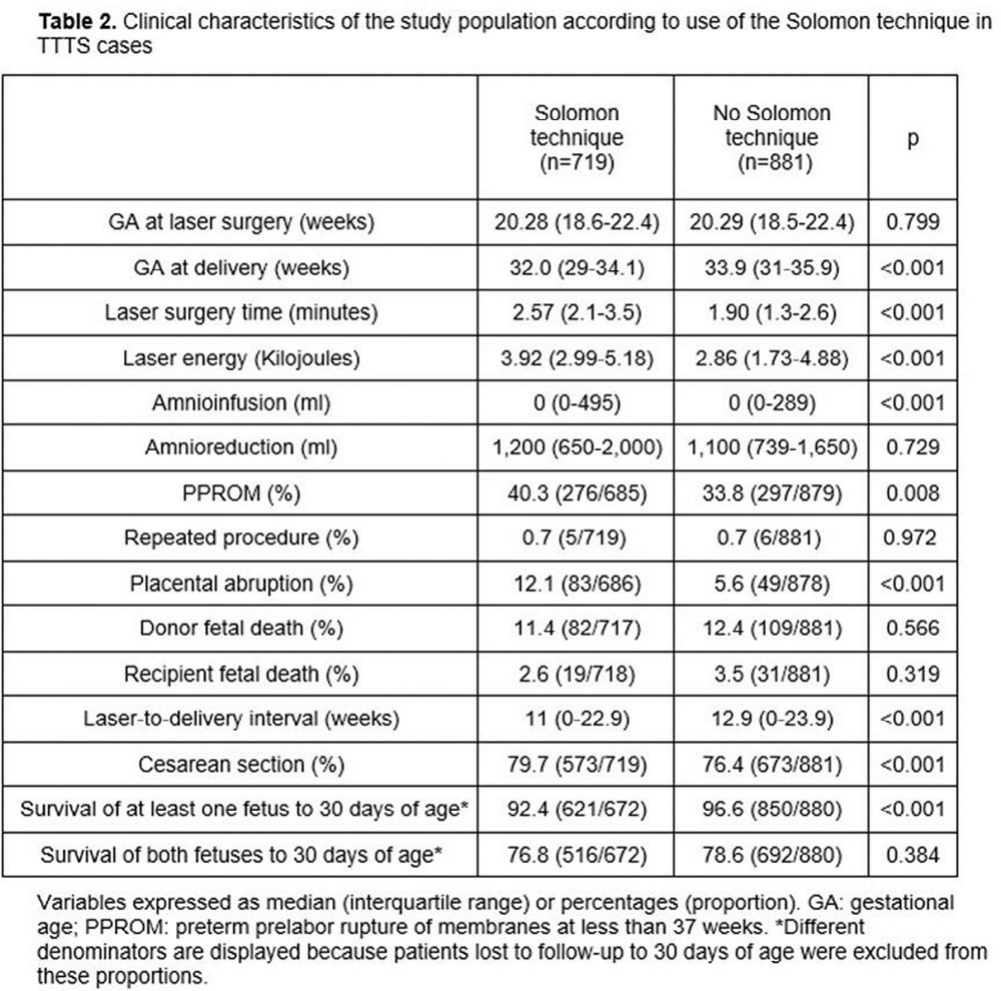

**10:30 AM – 12:00 PM**

## 818 | **Missed** Opportunities**: RPR Positivity at Delivery, Evaluating Perinatal Outcomes by Syphilis Treatment Adequacy**

JingJing Zhu^1^; Jeannie C. Kelly^2^; Amanda C. Zofkie^1^

^1^Washington University School of Medicine, St. Louis, MO; ^2^Washington University School of Medicine, Washington University School of Medicine, MO

**Objective**: The congenital syphilis rate in the United States has risen eight times within the last decade. We compared characteristics and perinatal outcomes among patients with a positive rapid plasma reagin (RPR) test at delivery by adequacy of syphilis treatment.

**Study Design**: We conducted a retrospective cohort study of all patients with a positive RPR at delivery at a tertiary care center (Dec 2019–Dec 2023). Deliveries were classified as adequately treated (completed stage‐appropriate penicillin during this pregnancy without reinfection) or inadequately treated. False‐positives and serofast results from infections treated previously were excluded from analysis. Data were abstracted from medical records. Primary outcomes were preterm birth and NICU admission; secondary outcomes included birthweight, neonatal death, infant RPR positivity, and infant treatment. Chi‐square, Fisher's exact, and Wilcoxon rank‐sum tests were used. Multivariable logistic regression was performed for adjusted analyses.

**Results**: Among 14, 405 deliveries, 162 had a positive RPR, with 109 meeting inclusion criteria. Of these, 76 (70%) were adequately treated; 63 (83%) were newly diagnosed during pregnancy and 13 (17%) were diagnosed prior to pregnancy and treated during prenatal care. Of 33 inadequately treated cases, 26 (79%) were diagnosed during pregnancy, including 18 (55%) newly identified at delivery. Inadequately treated patients were more likely to be older (31 vs 26 years, *p* < 0.001), have limited prenatal care (58% vs 4%, *p* < 0.001), comorbid Hepatitis C (36% vs 8%, *p* = 0.001), and illicit substance use (82% vs 38%, *p* < 0.001). Preterm birth rates did not differ by cohort, but NICU admissions were higher in inadequately treated cases (aOR 14.1, 95% CI 2.5–266.1). Inadequately treated cases also had lower birthweight (*p* < 0.001) and were more likely to require 10 days of neonatal treatment (*p* < 0.001).

**Conclusion**: Most inadequately treated syphilis cases at delivery were diagnoses made at time of delivery. Inadequate treatment is strongly associated with social vulnerability and adverse neonatal outcomes.

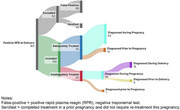

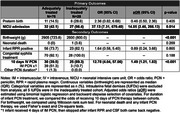

**10:30 AM – 12:00 PM**

## 819 | **Syphilis** Screening **to Follow‐Up: Role of Social Determinants in Pregnant Patients Visiting the ED**

Joe Haydamous^1^; Kayla M. Evans^2^; Laura Diab^3^; Analuisa C. Mosqueda^3^; Juan C. Padilla Ruiz^4^; Sabrina C. DaCosta^3^; Cassandra Igbe^5^; Mason Collie^6^; Irene A. Stafford^3^

^1^Department of Obstetrics, Gynecology and Reproductive Sciences, McGovern Medical School at UTHealth Houston, Department of Obstetrics and Gynecology, McGovern Medical School at UT Health, Houston, TX; ^2^McGovern Medical School – The University of Texas Health Science Center at Houston, Houston, TX; ^3^Division of Maternal‐Fetal Medicine, Department of Obstetrics, Gynecology and Reproductive Sciences, McGovern Medical School at UTHealth Houston, Houston, TX; ^4^UTHealth Houston, UTHealth Houston, TX; ^5^The University of Texas Health Science Center at Houston, The University of Texas Health Science Center at Houston, TX; ^6^McGovern Medical School at UTHealth Houston, Houston, TX

**Objective**: Emergency departments (EDs) offer a critical opportunity to identify syphilis during pregnancy. However, patient factors that influence test uptake and follow‐up remain understudied. This study evaluates the role of social determinants of health (SDOH) in syphilis test acceptance and linkage to prenatal care among pregnant ED patients.

**Study Design**: After Institutional Review Board approval, a prospective cohort study was conducted at the University of Texas Health Science Center in Houston, TX. Pregnant patients presenting to the ED between 11/05/24 and 06/01/25 were offered opt‐out syphilis testing and linkage to prenatal care. Key SDOH variables included race/ethnicity, primary language, insurance type, prenatal care status, partner presence, and substance use. Multivariable logistic regression identified predictors of test acceptance and prenatal care follow‐up.

**Results**: Of 230 patients approached, 178 (77%) accepted testing. Hispanic patients had significantly higher odds of accepting testing than non‐Hispanic White patients (OR = 13.6, 95% CI: 1.5–308.6, *p* = 0.036), while patients who identified Spanish as their primary language had lower odds (OR = 0.11, 95% CI: 0.006–0.68, *p* = 0.046). Patients without prenatal care were more likely to accept testing (OR = 0.31, 95% CI: 0.14–0.66, *p* = 0.003), and those with Medicaid (OR = 0.38, 95% CI: 0.16–0.87, *p* = 0.024) or self‐pay status (OR = 0.19, 95% CI: 0.07–0.50, *p* = 0.001) were less likely to accept testing compared to privately insured patients (Table 1). Among tested participants, 88 (49.4%) followed up with prenatal care. Patients who identified Spanish as their primary language (OR = 0.24, 95% CI: 0.08–0.69, *p* = 0.010) and self‐pay patients (OR = 0.13, 95% CI: 0.04–0.36, *p* < 0.001) had lower odds of follow‐up (Table 2). Prenatal care, partner presence, and substance use were not significant.

**Conclusion**: Preferred language, insurance status, and ethnicity impact test uptake and prenatal care follow‐up. Addressing these communication and financial barriers may help reduce disparities in perinatal syphilis care.

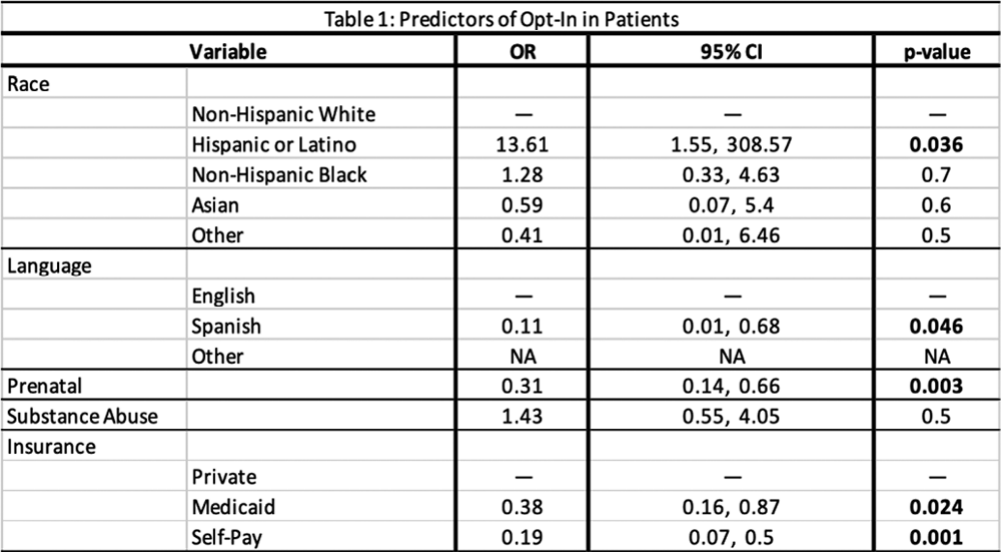

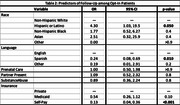

**10:30 AM – 12:00 PM**

## 820 | **Assessing the Accuracy and Safety of ChatGPT Responses to Common Questions About Preeclampsia in Pregnancy**

Joe Haydamous^1^; Laura Diab^2^; Farah H. Amro^2^

^1^Department of Obstetrics, Gynecology and Reproductive Sciences, McGovern Medical School at UTHealth Houston, Department of Obstetrics and Gynecology, McGovern Medical School at UT Health, Houston, TX; ^2^Division of Maternal‐Fetal Medicine, Department of Obstetrics, Gynecology and Reproductive Sciences, McGovern Medical School at UTHealth Houston, Houston, TX

**Objective**: To evaluate the accuracy, completeness, and safety of ChatGPT‐generated responses to common patient and provider questions about preeclampsia (preE) in pregnancy, assessing its potential role as an educational and triage‐support tool in obstetric care.

**Study Design**: Clinically relevant questions were developed from ACOG, SMFM, and WHO guidelines, as well as frequently discussed topics in patient forums and prenatal education resources. Responses were generated using GPT‐4 and independently evaluated by five maternal‐fetal medicine (MFM) specialists. Reviewers rated each response on a 5‐point Likert scale for accuracy, completeness, and safety. Two standardized prompts simulated patient‐ and provider‐directed queries. Questions were grouped into four categories: General Knowledge, Public Health/Prevention, Management & Treatment, and Diagnostic Interpretation.

**Results**: Across all domains, ChatGPT responses demonstrated high performance. Mean ratings (1–5 scale) were 4.60 for accuracy, 4.29 for completeness, and 4.43 for safety. The proportion of evaluations rated ≥4 was 92% for accuracy, 86% for completeness, and 85% for safety. Public Health/Prevention questions achieved the highest ratings across domains. Management & Treatment responses showed good alignment with guideline recommendations. Diagnostic Interpretation responses were generally accurate and safe but had slightly lower completeness, reflecting less detail in complex clinical decision‐making. No unsafe or potentially harmful content was identified.

**Conclusion**: ChatGPT generated safe, accurate, and generally complete responses to preE‐related questions. Its high accuracy and safety, particularly for public health and patient education topics, suggest potential for integration into prenatal counseling and triage support. Opportunities remain to enhance completeness in complex diagnostic contexts while maintaining its strong safety profile.

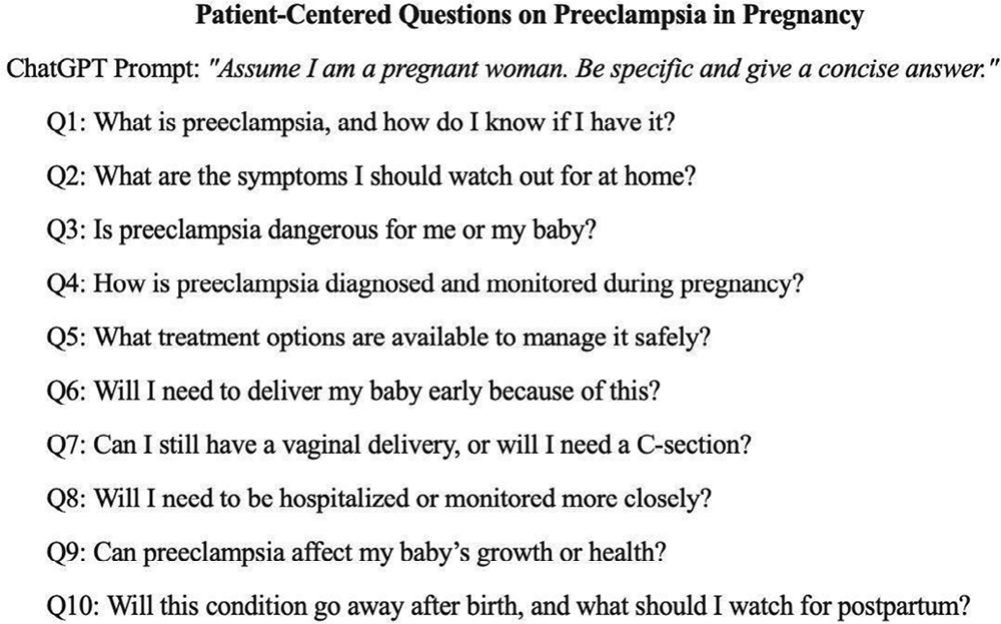

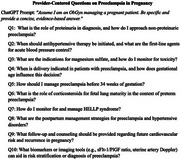

**10:30 AM – 12:00 PM**

## 821 | **The** Relationship **Between Antepartum Physical Activity Level and Postpartum Dyspareunia**

Jordan A. Gillenwater^1^; A. Dhanya Mackeen^2^; Victoria E. Boyd^1^; Cynthia Drazenovich^1^; Alicia Johns^1^; Hyagriv Simhan^3^; Richard Legro^4^; Douglas Teti^5^; Wenke Hwang^4^; Wadia Mulla^6^; Brenda Phillips^4^; Kristin K. Sznajder^4^

^1^Geisinger, Danville, PA; ^2^Geisinger Medical Center, Danville, PA; ^3^University of Pittsburgh Medical Center, Pittsburgh, PA; ^4^Pennsylvania State University, Hershey, PA; ^5^Pennsylvania State University, University Park, PA; ^6^Temple, Temple, PA

**Objective**: Few preventative strategies exist for postpartum (PP) dyspareunia which affects 41–83% of people. We examined whether there is an association between antepartum physical activity and PP dyspareunia.

**Study Design**: This was a secondary analysis of a prospective survey study (Pennsylvania (PA) Maternal and Infant Health in a Pandemic) of adult pregnancies at 4 PA health systems from 2023–2025. We compared 6‐month PP dyspareunia based on antepartum physical activity (sedentary vs. active) in those who had resumed intercourse PP. The active group was defined as performing ≥ 50% of aerobic activity recommended by the United States Department of Health and Human Servies (HHS) (Table 1). Chi‐squared and t‐tests evaluated the association between physical activity, participant characteristics, and the primary outcome (PP dyspareunia) for categorical and continuous variables, respectively. Multivariable logistic regression was used to adjust for the association between physical activity and PP dyspareunia using 2 models: 1 adjusting for all demographic variables and 1 adjusting only for variables with *p* < 0.10. *p*‐values of < 0.05 were considered significant.

**Results**: 954 patients had resumed intercourse: 412 (43%) were sedentary and 542 (57%) were active. 89% of active patients exceeded the minimum HHS recommendations and most engaged in a combination of moderate‐ and vigorous‐intensity activities. PP dyspareunia was reported by 116 (28%) and 151 (28%) of the sedentary and active groups, respectively. Physical activity was not associated with PP dyspareunia in the unadjusted (OR = 0.98; 95% CI 0.74, 1.31) or either adjusted (Tables 1 & 2) models. Higher parity was protective against PP dyspareunia (aOR 0.79; 95% CI 0.69, 0.92) (Table 1) when adjusting for all other demographics.

**Conclusion**: Antepartum physical activity was not associated with a reduction in PP dyspareunia. Additional investigation for postpartum dyspareunia prevention is warranted.

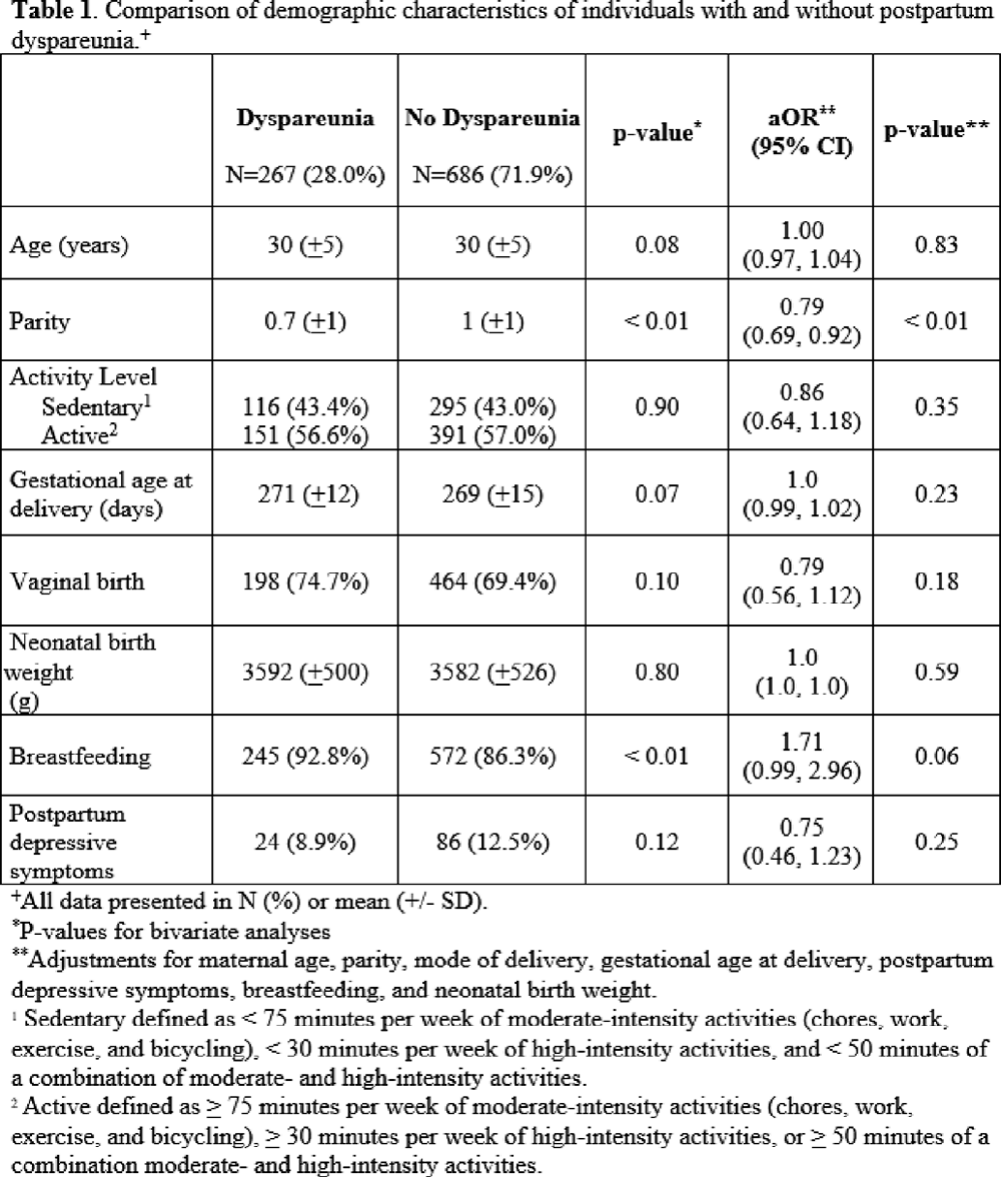

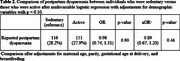

**10:30 AM – 12:00 PM**

## 822 | Postpartum **diabetes screening before hospital discharge in patients with gestational diabetes: a meta‐analysis**

Jordan A. McKinney^1^; Courtney Filliben^2^; Roxana Preis^2^; Luis Sanchez‐Ramos^2^

^1^University of Florida College of Medicine, Gainesville, FL; ^2^University of Florida College of Medicine, Jacksonville, FL

**Objective**: Individuals with gestational diabetes mellitus (GDM) are at increased risk of persistent glucose intolerance and progression to type 2 diabetes postpartum. Although guidelines recommend screening with a 75‐g oral glucose tolerance test (OGTT) at 4–12 weeks postpartum, adherence is low. In July 2024, ACOG suggested that performing the 75‐g OGTT on postpartum days 1–4, before hospital discharge, is a reasonable alternative. This systematic review and meta‐analysis evaluates the diagnostic accuracy and completion rates of inpatient OGTT performed on postpartum days 1–4, using the 4–12 week OGTT as the reference standard.

**Study Design**: We conducted a search of electronic databases and gray literature from January 1, 1985 to July 31, 2024. Eligible studies included individuals with GDM who underwent OGTT prior to hospital discharge and again at 4–12 weeks. Pooled sensitivity, specificity, likelihood ratios, diagnostic odds ratio (OR), and area under the hierarchical summary receiver operating characteristic (HSROC) curve were calculated using a bivariate random‐effects model. Screening adherence was compared using pooled proportions and ORs. Publication bias was assessed via Egger's test.

**Results**: This systematic review and meta‐analysis included 12 studies involving 1, 528 participants. Pooled sensitivity and specificity of OGTT prior to discharge were 88% (95% CI: 81–93%) and 84% (95% CI: 77–89%), respectively. The diagnostic OR was 1.32 (95% CI: 0.82–2.12), and the HSROC area was 0.91. Positive and negative likelihood ratios were 5.5 and 0.14, respectively. Screening completion was significantly higher prior to discharge (90.4%, 95% CI: 84.3–94.2%) compared to routine 4–12 week testing (57.1%, 95% CI: 46.5–67.1%), with a pooled OR of 5.91 (95% CI: 3.52–9.91). No small‐study effects were detected.

**Conclusion**: OGTT performed prior to hospital discharge demonstrates good diagnostic accuracy and substantially higher adherence than 4–12 week screening. These findings support prior‐to‐discharge testing as a practical and effective strategy for postpartum diabetes screening in individuals with GDM.

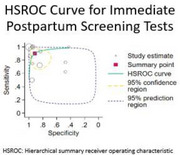

**10:30 AM – 12:00 PM**

## 823 | **Prenatal and Opioid Treatment Provider Practices, Knowledge, and Attitudes Regarding Perinatal Opioid Use Disorder Care**

Jose Reyes^1^; Abby Dolan^1^; Shoshana Aronowitz^1^; Max Jordan Nguemeni Tiako^2^

^1^University of Pennsylvania, Philadelphia, PA; ^2^University of California, Los Angeles (UCLA), Los Angeles, CA

**Objective**: Pregnant patients with opioid use disorder (OUD) face significant barriers to comprehensive care, despite the availability of effective treatments. We aimed to identify structural and clinical gaps that contribute to missed treatment opportunities during pregnancy.

**Study Design**: We administered a national cross‐sectional survey (Oct 2020‐Mar 2021) to clinicians providing obstetric and/or addiction care. The survey assessed practices, communication, attitudes, and training regarding OUD management during pregnancy. Descriptive statistics, chi‐square tests, and Mann‐Whitney tests were used to compare groups.

**Results**: Of the 113 respondents (80 [73%] female) from 28 states, 63 (56%) primarily provided prenatal care, and 50 (44%) primarily provided addiction care. Most practiced in the ambulatory setting (70 [62%]) and regularly (44 [39%]) or occasionally (50 [44%]) managed pregnant patients with OUD. Subgroup demographic and practice characteristics were similar. Despite broad endorsement of harm reduction strategies (78 [69%] strongly endorsed), 33 (30%) clinicians lacked harm reduction training. Only 60 (51%) were formally trained on treating OUD in pregnancy.

Among addiction providers, 48 (96%) offered pregnancy testing, though 5 (10.0%) only at intake and 34 (68%) only upon request or per symptoms. Most (43 [86%]) offered family planning services. Thirteen (26.0%) recommended but did not refer to prenatal care, and only 18 (36.0%) had protocols for patients unable to access prenatal care. Among referring providers, 7 (16%) did not communicate with the receiving provider post‐referral.

Among prenatal care providers, 25 (40%) managed OUD themselves. Most (59 [94%]) supported MOUD treatment during pregnancy, with 38 (60%) strongly agreeing. However, 16 (26%) viewed treating this population as challenging.

**Conclusion**: This study highlights critical gaps in care coordination, training, and protocols that leave pregnant people who use drugs vulnerable to delays in prenatal and addiction care access. Innovative care models – such as co‐located care – may help prevent avoidable harms due to delays in care.

**10:30 AM – 12:00 PM**

## 824 | Association **of Socioeconomic Deprivation With Maternal Vaccination Rates Before and After COVID Pandemic**

Judy Jiang^1^; Bhavana Janga^2^; Hannah Hill^3^; Kelly S. Gibson^4^; Hansika Kunduru^5^

^1^MetroHealth Medical Center/Case Western Reserve University, Cleveland, OH; ^2^MetroHealth Medical Center / Case Western Reserve University, Cleveland, OH; ^3^MetroHealth Medical Center, The MetroHealth System, OH; ^4^MetroHealth Medical Center/ Case Western Reserve University, Cleveland, OH; ^5^Case Western Reserve University, Case Western Reserve University, OH

**Objective**: To examine the trends in uptake of recommended vaccines for pregnant persons from 2016 to 2024 and to determine how these trends differ when looking at patients stratified by Social Vulnerability Index (SVI) and Area Deprivation Index (ADI) as measures of socioeconomic deprivation.

**Study Design**: Retrospective cohort study utilizing the Epic Cosmos aggregated data set, which was used to query diagnoses, vaccinations, and temporal data from Jan 2016 to Dec 2024 for patients pregnant at the time of the vaccination. Filter presets allowed us to see the number of patients who accepted the tetanus, diphtheria and pertussis (TDaP), influenza, and COVID‐19 vaccines during pregnancy. Trends were examined by fitting multivariable logistic regression models predicting vaccination on year and relevant independent variables.

**Results**: Data on 23, 118, 213 pregnant patients were queried. TDaP vaccination rates trend up from 43.1% in 2016 to a peak in 2020 at 52.2% and then trended back down to 43.4% in 2024. Influenza vaccination shifted from 19.8% in 2016 to a peak of 29% in 2020 followed by a drop back to 19% in 2024. COVID‐19 vaccinations peaked in 2021 at 14.4% of the pregnant population but then dropped to 0.04% in 2024. These trends were similar for all races and ethnicities. When examining patients stratified by quartiles of ADI and SVI, vaccination rates were found to inversely correlate with both measures of socioeconomic deprivation. Patients in the first SVI quartile (0–25), indicating the least social vulnerability, have the highest rates of vaccination and those in the last quartile have the lowest vaccination rates (*p* < 0.001). Results were unchanged when stratified by ADI.

**Conclusion**: After the COVID pandemic, vaccination rates are decreasing in pregnant patients. Patients with the highest levels of social vulnerability had the lowest rates of vaccination. These results highlight the importance of vaccine education and outreach to those most vulnerable to infection.

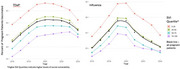

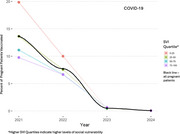

**10:30 AM – 12:00 PM**

## 825 | Implementation **of YEARS Criteria for Pulmonary Embolism Workup in Pregnancy in a Midwestern Hospital System**

Julia Burd^1^; Nicolette Pember^2^; Nandini Raghuraman^3^; Antonina I. Frolova^3^; Jeannie C. Kelly^4^; Sydney M. Thayer^4^

^1^Perelman School of Medicine, University of Pennsylvania, St. Louis, MO; ^2^Virginia Commonwealth University Health System, Richmond, VA; ^3^Washington University School of Medicine, St. Louis, MO; ^4^Washington University School of Medicine, Washington University School of Medicine, MO

**Objective**: In February 2024, our hospital system implemented the YEARS criteria, using D‐dimer to risk stratify need for chest imaging (CT pulmonary angiography [CTA] or V/Q scan) in cases of pregnant patients with pulmonary embolism (PE) symptoms. We hypothesized that this intervention would decrease patient radiation exposure without missed PE events.

**Study Design**: We conducted a retrospective cohort study of all pregnant patients evaluated for PE using D‐dimer, CTA, V/Q scan, or lower extremity (LE) Doppler ultrasound across 11 hospitals February 1, 2024‐January 31, 2025. The primary outcome was percent of cases where chest imaging was avoided through low‐risk D‐dimer and/or LE Dopplers. The process measure was YEARS criteria compliance. The balancing measure was protocol failure, defined as diagnosis of venous thromboembolism within three months of negative workup. Multivariable logistic regression was used to adjust for potential confounders of compliance.

**Results**: There were 85 PE evaluations with 2 PE events diagnosed (2.4% diagnostic yield). The YEARS algorithm was followed in 56 of 85 cases (65.9% compliance). For the primary outcome, 16 patients in the compliant cohort avoided chest imaging through low‐risk initial testing, yielding a theoretical 28.6% decrease in chest radiation events (Figure 1). Among patients ruled out for PE, no VTE events were diagnosed within the incorporated hospitals in the subsequent three months, demonstrating a 0% failure rate After adjusting for confounders, university affiliation, clinician type, hospital department, and pregnancy trimester were not associated with compliance differences (Table 1).

**Conclusion**: The YEARS criteria were acceptable to practitioners and demonstrated safety and efficacy in preventing radiation exposure in a pregnant population without missed VTE events when we assume all patients would have otherwise received chest imaging.

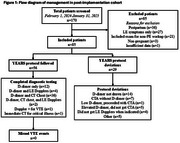

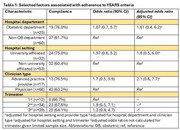

**10:30 AM – 12:00 PM**

## 826 | **Maternal and Neonatal Outcomes by Mode of Delivery in Botswana**

Júlia Sroda Agudogo^1^; Mercy Nassali^2^; Annliz Macharia^3^; Leah Savage^4^; Sarah Hanson^3^; Kago Ganagagabo^2^; David Tlhabano^5^; Francoise Rubgega^6^; Rebecca Zash^3^; Justus Hofmeyr^2^; Roger L. Shapiro^7^; Rebecca Luckett^3^

^1^Beth Israel Deaconess Medical Center, Beth Israel Deaconess Medical center, MA; ^2^University of Botswana, Gaborone, Central; ^3^Beth Israel Deaconess Medical Center, Boston, MA; ^4^Harvard Medical School, Boston, MA; ^5^Ministry of Health, Gaborone, Botswana, Gaborone, Central; ^6^University of Botswana, University of Botswana, Central; ^7^Botswana Harvard Health Partnership, Gaborone, Central

**Objective**: Cesarean births (CB) are associated with morbidity globally; however, risks of CB have not been well‐quantified in Botswana. This study aimed to investigate maternal and neonatal outcomes in a large cohort in Botswana by mode of delivery.

**Study Design**: Maternal and neonatal outcomes were abstracted from medical records at discharge for births at Princess Marina Hospital, the largest tertiary referral hospital in Botswana. We evaluated maternal and neonatal outcomes, impact of CB indications, and interventions by mode of delivery.

**Results**: From November 2021 to October 2024, 16, 419 births were recorded; 11, 753 (71.6%) vaginal births (VB) and 4, 666 (28.4%) CB. Of those with known CB indication, 241 (6.0%) were categorized as planned without known risk factors, 2235 (55.9%) were planned with risk factors, 566 (14.2%) had a failed VB attempt/labor dystocia, and 954 (23.9%) were for urgent/emergent indications. Demographic factors were comparable overall, with notably increased (median ±SD) age (30.4 ± 6.3 years versus 27.8 ± 7.0 years), weight (81.1 ± 18.6 kilograms versus 75.2 ± 16.8 kilograms) and parity (1.4 ± 1.2 births versus 1.2 ± 1.4 births) in CB versus VB groups. There were higher rates of obstetric haemorrhage, acute kidney injury, cardiopulmonary events, infection, hypertensive disorders, maternal death, additional uterotonics, tranexamic acid, transfusion, and hysterectomy (all *p* < 0.05) among those who underwent CB versus VB. Infants born via CB were more likely to experience neonatal intensive care unit (NICU) admission and neonatal death (all *p* < 0.05) compared to VB group (Table 1). Urgent/emergent CB were associated with higher rates of hypertension, hemorrhage, infection, cardiopulmonary events, transfusion, uterotonics, tranexamic acid, hysterectomy, NICU admission, Apgar < 4 min at 5 mins, and neonatal death (all *p* < 0.05) compared to planned/routine CB without risk factors (Table 2).

**Conclusion**: CB, especially for urgent/emergent indications, was associated with increased maternal and neonatal morbidity at a tertiary hospital in Botswana. Interventions to optimize CB outcomes are warranted.

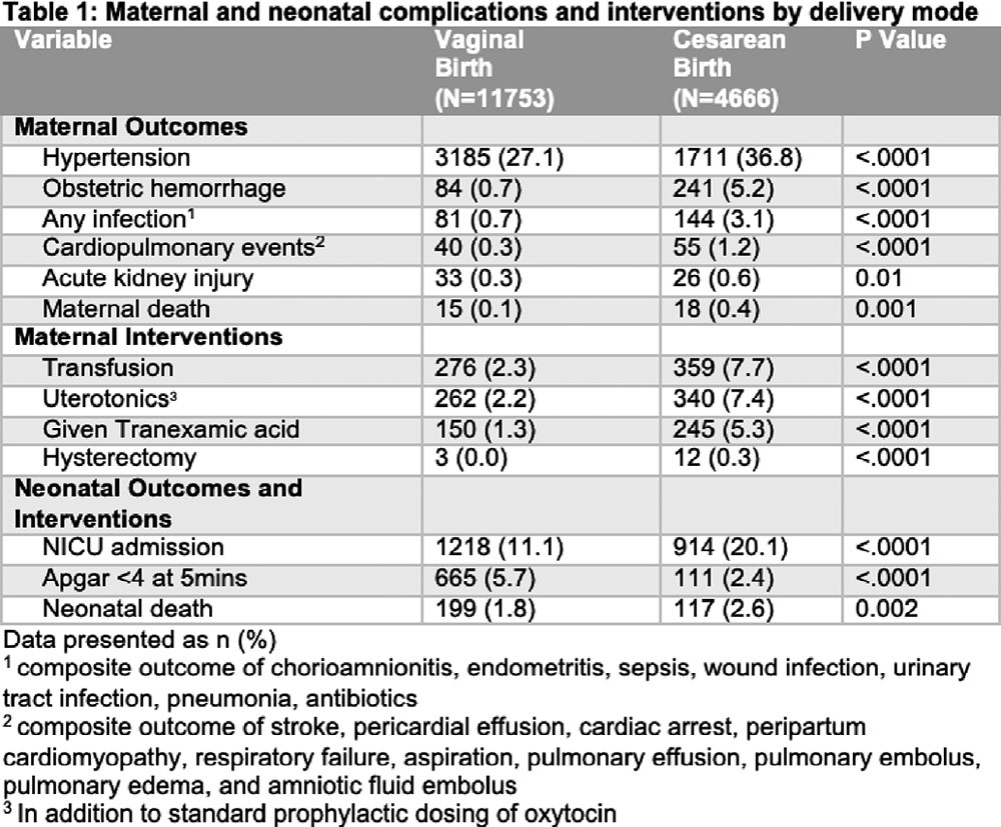

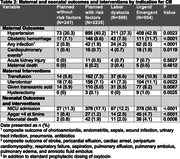

**10:30 AM – 12:00 PM**

## 827 | **Maternal Percentage Body Fat as a Possible Modulator of Fetal Adiposity: A Prospective Cohort Study**

Junko Tamai; Satoru Ikenoue; Keisuke Akita; Koki Shigematsu; Yushi Abe; Naotsugu Ishikawa; Yasuhiko Ogata; Yuka Fukuma; Yuya Tanaka; Toshimitsu Otani; Marie Fukutake; Yoshifumi Kasuga; Mamoru Tanaka

Department of Obstetrics and Gynecology, Keio University School of Medicine, Shinjuku‐ku, Tokyo

**Objective**: The intrauterine environment is known to influence short‐ and long‐ term health outcomes of the offsprings. Maternal obesity is associated with infant adiposity and early onset metabolic syndrome, however the association between maternal adiposity and fetal fat deposition in utero remains unclear. This study aimed to investigate the association between percentage body fat (%BF) and fetal adiposity across gestation.

**Study Design**: A prospective study was conducted in a cohort of 113 singleton pregnancies. Maternal %BF was assessed at 10, 24, 30 and 36 weeks’ gestation using bioelectrical impedance analysis. Fetal ultrasonography was performed at 24, 30 and 36 weeks. Estimated fetal adiposity (EFA) was calculated as the average of standardized z scores of arm percentage fat area, thigh percentage fat area and anterior abdominal wall thickness. Multiple linear regression was conducted to examine the association between maternal %BF and EFA adjusting for potential confounding factors including maternal age, parity, pre‐gravid BMI, gestational weight gain, and infant sex.

**Results**: Maternal %BF at 10, 24, 30 and 36 weeks were not associated with EFA at 24 and 30 weeks. Maternal %BF at 24, 30 and 36 weeks (but not at 10 weeks) were significantly and positively associated with EFA at 36 weeks. Even after controlling for the confounding factors, maternal %BF at 24 weeks (β = 0.043, *p* = 0.004), 30 weeks (β = 0.354, *p* = 0.002) and 36 weeks (β = 0.371, *p* = 0.002) significantly correlated with EFA at 36 weeks. Maternal %BF at all time points were not associated with estimated fetal weight across gestation.

**Conclusion**: Maternal %BF after 24 weeks were significantly correlated with EFA at 36 weeks. Maternal %BF in mid‐gestation could be an early marker for identifying increased fetal adiposity in late gestation, when fetal fat deposition rapidly accelerated.

**10:30 AM – 12:00 PM**

## 828 | Evaluating **Quality of Content on In Utero Myelomeningocele Repair Across YouTube, TikTok, and Instagram Reels**

Kai Holder^1^; Faith Kehinde^2^; Prottusha Sarkar^3^; Tiffany Deihl^4^

^1^UPMC Magee‐Womens Hospital, Pittsburgh, PA; ^2^University of Pittsburgh Medical Center, Pittsburgh, PA; ^3^University of New Mexico, Albuquerque, NM; ^4^UPMC Magee Womens Hospital, Pittsburgh, PA

**Objective**: Social media platforms are increasingly used by high‐risk obstetric patients seeking information on complex prenatal conditions. This study aimed to evaluate the quality of videos on YouTube, TikTok, and Instagram Reels as educational resources on in utero fetal surgery for myelomeningocele.

**Study Design**: A systematic search of YouTube, TikTok, and Instagram Reels was conducted using a public account and expert‐identified keywords related to fetal myelomeningocele repair. For each platform, the first 60 videos per keyword were screened, and unique videos were analyzed. Each video was independently evaluated using an expert‐derived content score and the DISCERN instrument, a validated tool for evaluating consumer health information. Videos scoring ≥5 (content) and ≥39 (DISCERN) were classified as high quality. Descriptive statistics and intergroup comparisons were performed using Bonferroni‐adjusted Mann‐Whitney U tests.

**Results**: A total of 467 videos were analyzed: YouTube (*n* = 179), TikTok (*n* = 220), and Instagram Reels (*n* = 68). Mean DISCERN scores were highest on YouTube (27.92 ± 10.47), followed by Instagram Reels (20.88 ± 8.27) and TikTok (19.96 ± 6.16); all *p* < 0.001. Mean content scores followed a similar pattern: YouTube (2.85 ± 2.64), Instagram Reels (0.94 ± 1.53), and TikTok (0.68 ± 0.96); all *p* < 0.001. Only 12 videos (2.57%) met high‐quality criteria: YouTube (*n* = 11; 6.15%), Instagram Reels (*n* = 1; 1.47%), and TikTok (*n* = 0; 0.00%).

**Conclusion**: While YouTube had the highest overall video quality, high quality content was nearly absent on TikTok and Instagram Reels. Given that in utero myelomeningocele repair is a complex intervention within the setting of high‐risk obstetric care, access to accurate, high‐quality educational content may significantly influence patient understanding and decision‐making. As short‐form video platforms such as TikTok and Instagram Reels are widely used by obstetric patients, targeted efforts to improve the quality of information on these platforms are essential to support informed counseling and timely decision‐making.

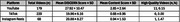

**10:30 AM – 12:00 PM**

## 829 | Predictors **of Placental Abruption in Fetoscopic Laser Photocoagulation for Twin‐to‐Twin Transfusion Syndrome**

Kamran Hessami^1^; Brian A. Burnett^1^; Rebecca M. Johnson^1^; Allie M. Sidwell^2^; Jessian L. Munoz^3^; Cara Buskmiller^4^; Roopali V. Donepudi^5^; Magdalena Sanz Cortes^5^; Michael A. Belfort^1^; Ahmed A. Nassr^5^

^1^Baylor College of Medicine, Houston, TX; ^2^University of Illinois College of Medicine, Chicago, IL; ^3^Texas Children's Hospital, Texas Children's Hospital, TX; ^4^Baylor College of Medicine, Austin, TX; ^5^Baylor College of Medicine, Baylor College of Medicine, TX

**Objective**: Twin‐to‐twin transfusion syndrome (TTTS) affects monochorionic twin pregnancies and is managed with fetoscopic laser photocoagulation (FLP), the standard of care. While FLP improves outcomes, it may be associated with complications, including placental abruption. This study aimed to identify clinical and operative predictors of placental abruption following FLP in cases of TTTS.

**Study Design**: This retrospective cohort study included monochorionic twin pregnancies that underwent FLP for TTTS at a single tertiary fetal center between 2012 and 2024. Patients were stratified by the clinical suspicion of placental abruption. Demographic, intraoperative, and postoperative characteristics were compared between study groups. Multivariate logistic regression was used to find the association between abruption and placental location, pre‐operative cervical length, gestational age at surgery, operative factors (cannula size, laser energy, laser time, and surgery duration), and postoperative complications (chorioamniotic membrane separation [CAS] and septostomy).

**Results**: Of 562 pregnancies with TTTS and undergoing FLP, 75 (13%) were delivered due to suspected placental abruption. Baseline demographic and operative characteristics were similar between groups. Abruption was associated with earlier gestational age at delivery (29.2 ± 3.4 vs 30.7 ± 4.4 weeks, *p* < 0.001). Anterior placentation was independently associated with abruption (OR 1.95, 95% CI 1.07–3.53; *p * = 0.03). No other intraoperative or postoperative variables were significantly associated with risk of abruption.

**Conclusion**: Anterior placentation is an independent risk factor for placental abruption following FLP for TTTS. Its identification may inform risk stratification, preoperative counseling, and postoperative surveillance.

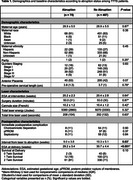

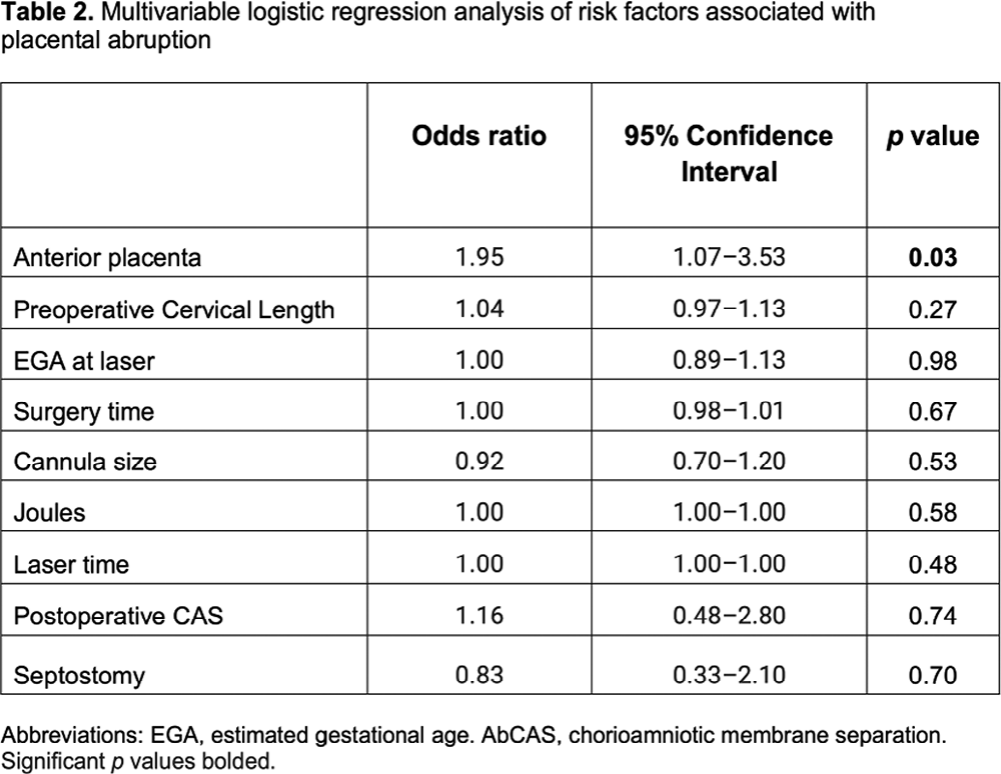

**10:30 AM – 12:00 PM**

## 830 | Phenotypic **Differences in Risk Factors and Pregnancy Outcomes of Gestational Diabetes in the Asian‐American Diaspora**

Kandace Fung^1^; Apekshya Nepal^2^; Kennedi Randolph^2^; Song Hyun Kim^2^; Nazow B. Tarakai^2^; Jiayue Chen^3^; Lorna Kwan^4^; Christina S. Han^5^

^1^Charles R. Drew University of Medicine and Science; David Geffen School of Medicine at University of California, Los Angeles (UCLA), Los Angeles, CA; ^2^David Geffen School of Medicine at University of California, Los Angeles (UCLA), Los Angeles, CA; ^3^Department of Urology, David Geffen School of Medicine at University of California, Los Angeles (UCLA), Los Angeles, CA; ^4^Department of Urology, David Geffen School of Medicine at University of California, Los Angeles (UCLA), Los Angeles, CA; ^5^Department of Obstetrics and Gynecology, David Geffen School of Medicine at University of California, Los Angeles (UCLA), Los Angeles, CA

**Objective**: Asian American (AsA) pregnant individuals are disproportionately affected by metabolic conditions, including gestational diabetes mellitus (GDM). This study aims to examine the risk factors, clinical course, and pregnancy outcomes associated with GDM among AsA populations. Our hypothesis was that risk factors and pregnancy outcomes for GDM differ between AsA and non‐AsA populations.

**Study Design**: We conducted a retrospective case‐control study of patients at an academic institution between 01/2022 to 12/2023. Inclusion criteria were age 16–49 years, singleton gestation, and a 1hr GTT completed within the institution. Exclusion criteria included aneuploidy, pregestational diabetes, multiple gestations, bariatric surgery, cystic fibrosis, or chronic systemic steroid use. Data were abstracted from electronic health records. Comparisons were made between AsA and non‐AsA, and between AsA with GDM and non‐AsA with GDM (AsA+GDM vs non‐AsA+GDM). Kruskal‐Wallis and Chi‐squared were used where appropriate.

**Results**: Table 1 compares the 1216 deliveries, AsA (17%) and non‐AsA (83%), with the AsA group having higher mean age (*p* = 0.0154), lower early pregnancy BMI (*p* < 0.0001), more private insurance (*p* = 0.0054), higher social vulnerability index (SVI) quartile (*p* = 0.0019), and lower gestational age at delivery (*p* < 0.0001), but no differences in GDM prevalence and type. Table 2 compares AsA+GDM and non‐AsA+GDM, with AsA+GDM having higher mean age (*p* = 0.0404), higher weight gain (*p* = 0.0317), and higher rates of perineal lacerations (*p* = 0.0381). No differences were seen in delivery timing, hypertensive disorders in pregnancy, and neonatal birth weight.

**Conclusion**: Despite the protective factors of lower BMI, private insurance, and higher SVI in the AsA group, the rates of GDM were the same in AsA and non‐AsA pregnant persons. AsA with GDM were more likely to sustain higher weight gain in pregnancy and perineal lacerations, even with no differences in birthweight. These findings support further investigation into culturally and physiologically responsive approaches to obstetric risk mitigation for Asian American populations.

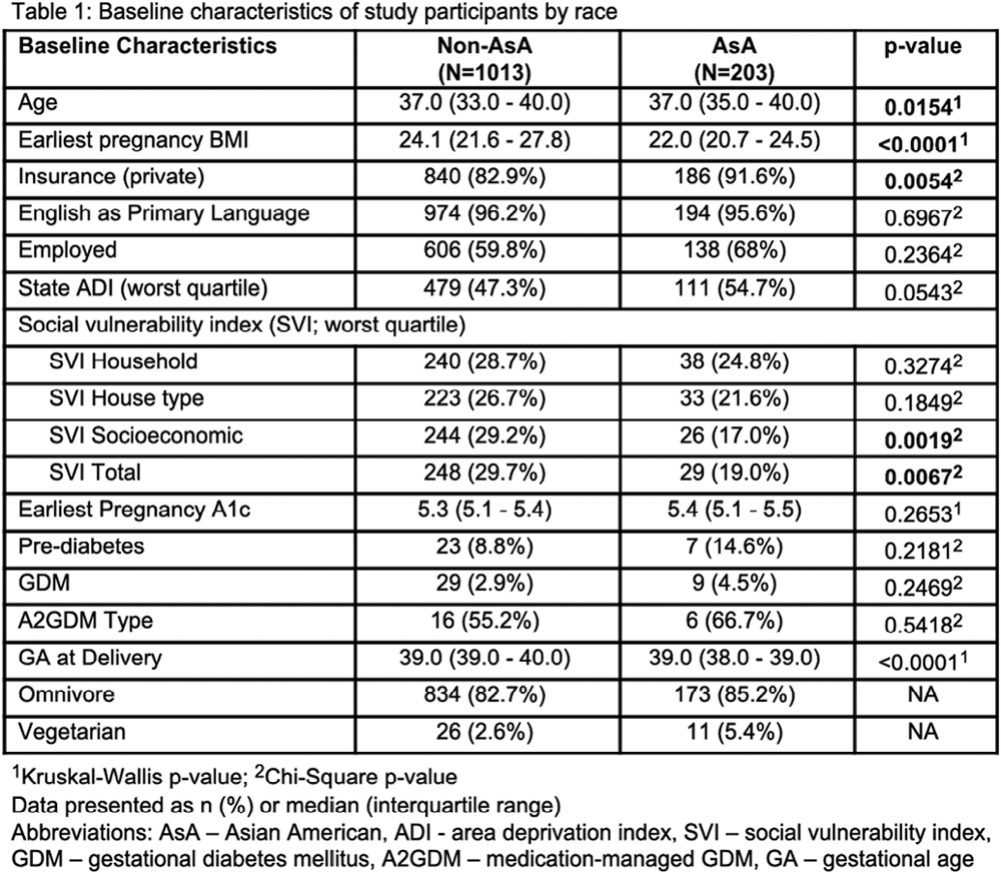

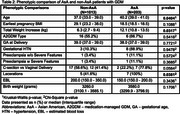

**10:30 AM – 12:00 PM**

## 831 | **Bridging Care Gaps: A Qualitative Study of Postpartum Health Coaching for Hypertensive Disorders of Pregnancy**

Elizabeth Albert^1^; Srishti Gupta^1^; Scott Hetzel^1^; Megan R. Knutson Sinaise^1^; Kristine Alaniz^1^; Kara K. Hoppe^2^

^1^University of Wisconsin‐Madison, Madison, WI; ^2^University of Wisconsin School of Medicine and Public Health, Department of Obstetrics and Gynecology, Madison, WI

**Objective**: To qualitatively assess participant experiences by study allocation in the STAC‐MyHEARTp randomized controlled trial: usual care vs. telephone health coaching for hypertensive disorders of pregnancy through 1‐year postpartum.

**Study Design**: This planned secondary qualitative study explored experiences of participants from the STAC‐MyHEARTp trial (NCT05685251), which tested the efficacy of health coaching vs. usual care in improving 12‐month postpartum hypertension follow‐up. A semi‐structured interview guide was developed using a deductive approach aligned with study aims. Participants were randomly selected for interviews, which were transcribed verbatim and independently coded by two team members in Dedoose, with a third resolving discrepancies. Codes were synthesized into broader themes reflecting postpartum and hypertension‐related care by study arm. Themes, definitions, and exemplary quotes were organized into a qualitative matrix and collaboratively refined.

**Results**: Thirty‐two of 140 participants completed interviews (16 per group). Table 1 shows participant characteristics. Five themes emerged (Table 2): (1) Motivation and accountability—coaching supported health behaviors via goal‐setting and check‐ins; (2) Power of the coaching relationship—the coach was trusted, supportive, and reduced isolation; (3) Self‐advocacy and empowerment—coaching encouraged active engagement with providers; (4) Hypertension awareness shaped by history—personal/family experiences influenced engagement; and (5) Unmet postpartum needs—participants cited gaps in mental health, support, and continuity of care.

**Conclusion**: Health coaching was perceived as highly valuable, filling gaps in routine postpartum care by providing education, support, promoting self‐advocacy and accountability. These findings support integrating health coaching into postpartum care for individuals with hypertensive disorders of pregnancy. There is early evidence from this cohort that there is interest in long‐term postpartum follow‐up to support their health, emphasizing the need for research on effective postpartum support strategies.

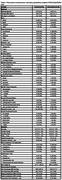

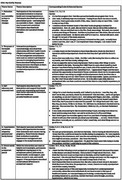

**10:30 AM – 12:00 PM**

## 832 | **Cost‐**Effectiveness **of Intramuscular Methylergonovine Prophylaxis at Cesarean Delivery for Twin Gestations: A Decision‐Analytic Model**

Kate Tolleson^1^; Amelia H. Gagliuso^2^; Aaron B. Caughey^3^

^1^New York University Grossman School of Medicine, NYU Grossman School of Medicine, NY; ^2^Oregon Health and Science University, Portland, OR; ^3^Oregon Health & Science University, Oregon Health & Science University, OR

**Objective**: Twin pregnancies are at increased risk for postpartum hemorrhage (PPH), transfusion, and maternal death. A recent randomized controlled trial (RCT) demonstrated that prophylactic administration of intramuscular (IM) methylergonovine after cord clamping significantly reduced blood loss during planned cesarean delivery (CD) in twin gestations. This study aimed to evaluate the cost‐effectiveness of IM methylergonovine for PPH prophylaxis at the time of CD in twin pregnancies.

**Study Design**: A decision‐analytic model was built using TreeAge software to compare the outcomes and cost‐effectiveness of prophylactic IM methylergonovine versus a placebo saline injection at the time of CD in twin gestations. The theoretical cohort included 85, 000 individuals, the estimated annual number of CD for twin gestations in the U.S. Maternal outcomes included postpartum hemorrhage, transfusion, ICU admission, hysterectomy, and maternal death. Probabilities, utilities, and costs were derived from the literature. A 50‐year time horizon and 3% annual discount rate were applied. The threshold for cost‐effectiveness was set at $100, 000 per QALY.

**Results**: In this cohort, prophylactic IM methylergonovine resulted in 31, 450 fewer PPH events, 5, 100 fewer transfusions, 326 fewer ICU admissions, three fewer hysterectomies, and the prevention of one maternal death, compared to placebo. IM methylergonovine was on average $100.48 less expensive per delivery and was the dominant strategy as it saved $8.5 million and led to an increase of 2, 550 QALYs (Table 1). Univariate sensitivity analysis demonstrated that IM methylergonovine is the dominant strategy until its cost exceeds $500, and remains cost‐effective until its cost exceeds $3, 107, which is far above the current price (Figure 1).

**Conclusion**: Routine administration of IM methylergonovine after cord clamping at the time of cesarean delivery for twin gestations is a cost‐effective strategy that reduces maternal morbidity and improves quality‐adjusted survival. These findings support the implementation of adjunctive uterotonic prophylaxis for high‐risk cesarean deliveries.

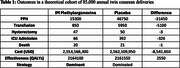

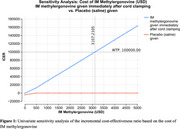

**10:30 AM – 12:00 PM**

## 833 | Simultaneous **Mifepristone and Misoprostol versus Misoprostol Alone in Second Trimester (MIST): A Randomized Controlled Trial**

Katherine H. Bligard^1^; Bridget C. Huysman^2^; Lori Atwood^2^; Nandini Raghuraman^3^; Antonina I. Frolova^3^; Jeannie C. Kelly^2^

^1^Washington University School of Medicine, Saint Louis, MO; ^2^Washington University School of Medicine, Washington University School of Medicine, MO; ^3^Washington University School of Medicine, St. Louis, MO

**Objective**: Mifepristone pretreatment shortens induction time and reduces retained placenta in nonviable second‐trimester pregnancies. However, the required 24‐to‐48‐hour delay may be impractical due to urgency, travel barriers, or limited access to specialized care. The MIST trial evaluated whether simultaneous mifepristone and misoprostol (mife+miso) shortens time to delivery versus misoprostol alone (miso).

**Study Design**: We conducted a randomized controlled trial of patients needing urgent induction between 14w0d–27w6d. Participants were randomized 1:1 to mife+miso or miso. The primary outcome was placental delivery within 12 hours. Secondary outcomes included delivery within 18 and 24 hours, use of additional medications/instruments, obstetric morbidity, and time to delivery using Kaplan‐Meier curves and Cox proportional hazard ratios. Analyses followed an intention‐to‐treat approach. A sample of 30 participants provided 80% power to detect an increase in 12‐hour delivery from 34% to 82% with an α of 0.05.

**Results**: Of 30 patients randomized, one in the miso group was withdrawn (15 mife+miso vs 14 miso in analysis). Baseline demographics, including gestational duration, parity, indication for care, and cervical dilation before induction were similar between groups. Simultaneous mifepristone use did not increase rate of delivery within 12 hours (33.3% mife+miso vs 35.7% miso, *p* = 0.89). Delivery within 18 and 24 hours were also similar, with most patients delivering within 18 hours. Overall time to delivery was also not altered by mifepristone (fetal HR 1.67 [95% CI 0.75–3.70], *p* = 0.20; placental HR 1.59 [95% CI 0.66–3.88], *p* = 0.30). There were no differences in use of adjuvant induction methods, obstetric composite morbidity, blood loss, or length of hospital stay. Retained placenta occurred in 27% (mife+miso) vs 29% (miso) of participants, *p* = 0.36.

**Conclusion**: In settings where mifepristone pretreatment is not feasible, simultaneous mifepristone and misoprostol administration did not reduce time to delivery or morbidity compared to misoprostol alone in non‐viable second trimester inductions.

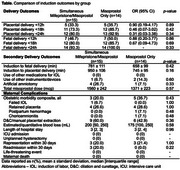

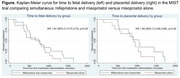

**10:30 AM – 12:00 PM**

## 834 | **Study of** Pregnancy **and Neonatal Health TIMing of dElivery (TIME) Randomized Trial for Gestational Diabetes**

Katherine L. Grantz^1^; Jessica Gleason^2^; Rajeshwari L. Sundaram^1^; Brenna L. Hughes^3^; Celeste Durnwald^4^; Jessica Page^5^; Alan TN Tita^6^; Robert M. Silver^7^; George Maxwell^8^; Joseph R. Biggio, Jr.^9^; John M. Thorp^10^; Daniel Molina^11^; Diane Putnick^1^; Fasil Tekola‐Ayele^1^; Edwina Yeung^1^

On behalf of the NICHD Study of Pregnancy and Neonatal Health (SPAN) TIMing of dElivery (TIME)

^1^Eunice Kennedy Shriver National Institute of Child Health and Human Development, National Institutes of Health, Bethesda, MD; ^2^NICHD, NIH, Bethesda, MD; ^3^Department of Obstetrics and Gynecology, Duke University School of Medicine, Duke, NC; ^4^University of Pennsylvania, Philadelphia, PA; ^5^Department of Maternal‐Fetal Medicine, Intermountain Healthcare. Department of Maternal‐Fetal Medicine, University of Utah Health, Salt Lake City, UT; ^6^Center for Research in Women's Health, University of Alabama at Birmingham, Birmingham, AL; ^7^Department of Obstetrics and Gynecology, University of Utah Health, Salt Lake City, UT; ^8^Inova Fairfax Medical Campus, Fairfax, VA; ^9^Ochsner Health, New Orleans, LA; ^10^University of North Carolina, Chapel Hill, Chapel Hill, NC; ^11^Technical Resources International, Inc., Bethesda, MD

**Objective**: To determine the optimal time between 37–39 weeks to initiate delivery for patients with gestational diabetes mellitus (GDM) to minimize perinatal morbidity and mortality.

**Study Design**: A multi‐site randomized trial, using an adaptive design, recruited pregnant women with poorly controlled GDM (elevated fasting or ≥3 post‐prandial blood glucoses after receiving diabetes education, large‐for‐gestational‐age (LGA), polyhydramnios or care nonadherence). Women were enrolled from 32 weeks (w) 0 days (d) to 36w6d and randomized at 35w0d–36w6d to one of seven 3‐day initiation of delivery arms between 37w0d to 39w6d (Table 1). Delivery mode was per usual obstetrical indications. Primary outcome was a composite of perinatal morbidity and mortality. The study was stopped by the DSMB due to low enrollment (target *N* = up to 3, 450).

**Results**: From 01/2023–11/2024, 2, 815 women with GDM were screened, 689 (24.5%) were eligible, 145 (5.2%) enrolled and 139 randomized. Of those randomized, 51.1% initiated delivery in the assigned window; 37.4% initiated delivery before the assigned window (44.2% due to medical indication; 25.0% spontaneous labor); and 5.8% after assigned window (62.5% due to patient or provider preference) with 5.8% unknown (e.g. trial closed). There was no significant difference in primary outcome among arms in intention‐to‐treat (ITT) or per‐protocol analyses. (Table 1) For individual primary neonatal outcomes (ITT), there was no pattern for rates of respiratory support, NICU > 1 day or birth trauma; and no other severe morbidity or death. For secondary outcomes, rates of LGA with advancing GA were 21.1%, 10.0%, 5.3%, 9.5%, 5.0%, 30.0%, 30.0%. Serious maternal morbidity was rare (Table 2). There was no pattern for delivery mode or shoulder dystocia.

**Conclusion**: Among women with poorly controlled GDM, there were high rates of co‐morbid conditions and medical indications for delivery prior to assigned windows. The trial provides valuable insight into delivery timing for this high‐risk population, highlighting the complexities of balancing varying outcomes associated with different gestational ages.

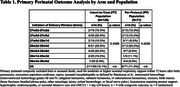

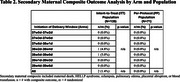

**10:30 AM – 12:00 PM**

## 835 | Postpartum **Follow‐Up During and After the COVID‐19 Pandemic in a Diverse Urban Population**

Rachel Nordlicht^1^; Jessica Atrio^2^; Kavita Vani^3^

^1^Albert Einstein College of Medicine, The Bronx, NY; ^2^Alice L. Walton School of Medicine, Bentonville, AR; ^3^Albert Einstein College of Medicine/Montefiore Medical Center, Bronx, NY

**Objective**: To evaluate postpartum follow‐up attendance before, during, and after the COVID‐19 pandemic in a large, urban, racially diverse obstetric population.

**Study Design**: We conducted a retrospective cohort study of 13, 874 patients who received prenatal care and delivered at Montefiore Medical Center between June 2018 – May 2022. Four periods were defined based on key COVID‐19 milestones: pre‐COVID (before 3/11/2020, pandemic declaration), peak COVID (3/11/2020–6/11/2020, elective procedures cancelled), late COVID (6/12/2020–6/15/2021, elective procedures resumed, no widespread vaccination), and post‐COVID (after 6/15/2021, vaccines widely available). The primary outcome was postpartum visit (PPV) attendance within 12 weeks of delivery. Chi‐square tests compared PPV attendance across periods. Logistic regression adjusted for demographics, prenatal care, comorbidities, delivery outcomes, and NICU admission. Secondary outcomes included emergency room (ER) visits and readmissions within 12 weeks.

**Results**: There were 6, 989 (50.4%) deliveries pre‐COVID, 944 (6.8%) during peak COVID, 3, 273 (23.6%) during late COVID and 2, 668 (19.2%) post‐COVID. Compared to pre‐COVID PPV attendance of 46.2%, PPV rates increased significantly during peak (73.5%), late (63.7%) and post‐COVID (59.8%), *p* < 0.01). After adjustment, compared to pre‐COVID, patients who delivered during peak COVID had a 3.5x higher odds of attending a PPV (aOR 3.50, 95% CI 2.97–4.11), 1.98x higher odds during late COVID (aOR 1.98, 95% CI 1.80–2.17) and 1.6x higher odds post‐COVID (aOR 1.61, 95% CI 1.45–1.78), all *p* < 0.01. Compared to pre‐COVID, ER visits were less likely during peak (aOR 0.56, 95% CI 0.44–0.69) and late COVID (aOR 0.83, 95% CI 0.73–0.93), *p* < 0.01. No significant differences in readmissions were found.

**Conclusion**: PPV attendance increased during the COVID‐19 pandemic and remained elevated post‐pandemic. This pattern may reflect forced changes in care delivery, such as expanded telehealth use, that may have helped reduce longstanding barriers to follow‐up. Sustained implementation of flexible care models may help preserve these gains.

**10:30 AM – 12:00 PM**

## 836 | **The** Postpartum **Cliff: Gaps in Postpartum Follow‐Up Among Individuals With Hypertensive Disorders**

Kelly F. Darmawan^1^; Elizabeth B. Sherwin^2^; Brian T. Bateman^2^; Danielle M. Panelli^3^; Jenny Y. Mei^4^; Ilona T. Goldfarb^2^; Kathleen C. Minor^4^; Sara Siadat^2^; Jena Pizula^4^; Stephanie A. Leonard^4^

^1^Stanford University, Palo Alto, CA; ^2^Stanford University, Stanford, CA; ^3^Stanford University Healthcare, Palo Alto, CA; ^4^Stanford University, Stanford University, CA

**Objective**: Individuals with hypertensive disorders of pregnancy (HDP) are at risk of short‐ and long‐term adverse sequelae. To mitigate risks, national guidelines recommend that patients with HDP have two postpartum follow‐up visits with obstetrics (within 3 weeks and between 3 to 12 weeks) and transition to primary care or cardiology within 1 year. We examined adherence to these guidelines and whether they differed by HDP groups in a nationwide cohort.

**Study Design**: We analyzed data from live births between 2014–2021 in the U.S. Merative^TM^ MarketScan^®^ Commercial Database. Hypertensive disorders were identified by diagnosis codes and categorized into mutually exclusive groups: severe HDP, mild HDP, and chronic hypertension (without preeclampsia). Provider visits were identified by provider specialty and outpatient visit MarketScan^®^ codes, and classified as Obstetrics (physicians and midwives), primary care (Internal, Family, or Preventative Medicine), and Cardiology. Multivariable modified Poisson regression models were conducted to estimate associations between HDP groups and presence of follow‐up visits.

**Results**: Among 922, 535 individuals, 167, 539 (18%) people had any HDP. 4% had severe HDP, 12% had mild HDP, and 2% had chronic hypertension without preeclampsia. Follow‐up rates at the 3–12 week obstetric visit ranged from 14–18% (Figure 1). Compared to individuals without HDP, rates of obstetric visits were higher across all HDP groups, most notable for the severe HDP group within 3 weeks (5% vs 15%, aRR: 2.62, 95% CI: 2.55–2.70) (Table 1). Similar results were seen for the 3–12 week obstetric visit and primary care and cardiology visits within 1 year postpartum. Follow‐up with primary care within 1 year was most notable in those with chronic hypertension compared to those without (60% vs 44%; aRR: 1.26, 95% CI: 1.25–1.28).

**Conclusion**: Follow‐up intervals across all groups, including patients with severe HDP, were much lower than recommended by national guidelines. Strategies to improve postpartum follow‐up and transition to primary care and cardiology are needed to mitigate risks for patients with HDP.

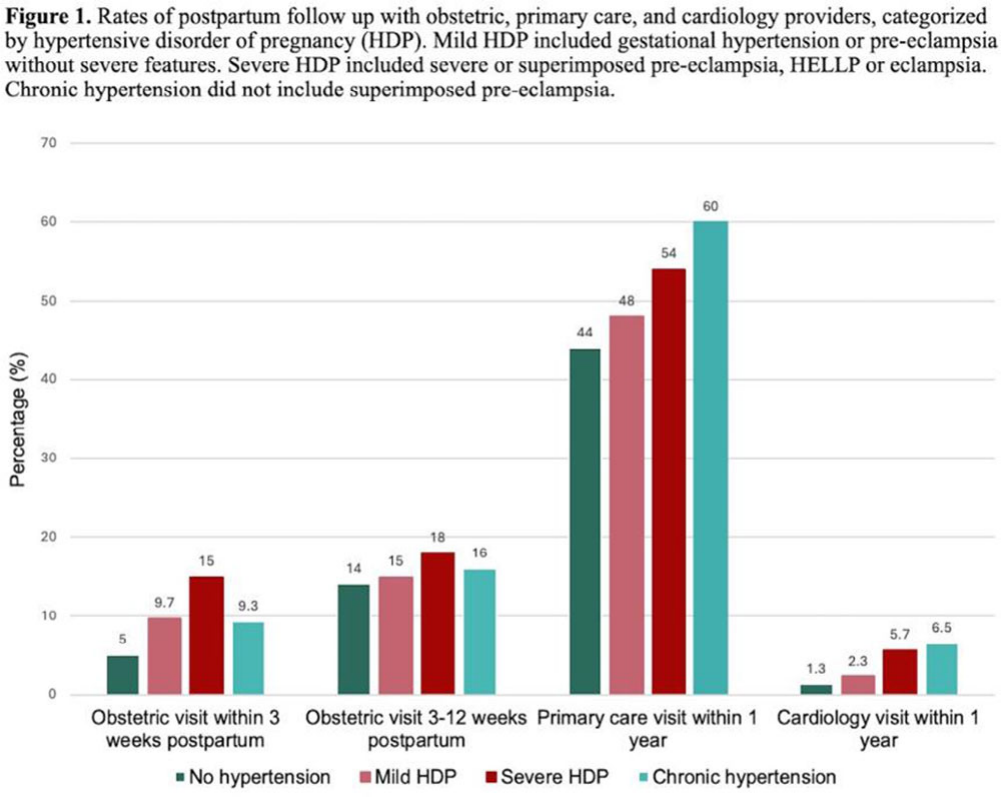

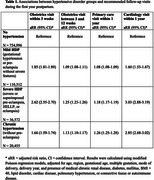

**10:30 AM – 12:00 PM**

## 837 | **Provider Remote Obstetrics‐Related Employment Education for Pregnant and Postpartum Patients: A Pilot Study**

Kelsey McNew^1^; Shakthi Unnithan^1^; Tracy Truong^2^; Thelma Fitzgerald^1^; Adwoa A. Baffoe‐Bonnie^1^; Barvina Toledo^1^; Danielle Lanpher^1^; Kelley E. C. Massengale^3^; Maya Jackson^4^; Sarahn M. Wheeler^5^

^1^Duke University School of Medicine, Durham, NC; ^2^Duke University Medical Center, Durham, NC; ^3^Diaper Bank of North Carolina, Durham, NC; ^4^MAAME, Inc., Durham, NC; ^5^Division of Maternal Fetal Medicine, Department of Obstetrics and Gynecology, Duke University, Duke University/Durham, NC

**Objective**: Obstetric providers are rarely trained on employment laws that impact pregnant workers. Thus, we developed the Provider Remote Obstetric‐Related Employment Education (PROMOTE) intervention, a 30‐minute online training and documentation toolkit. We pilot‐tested PROMOTE and evaluated the employment experiences, care adherence, and financial strain of patients.

**Study Design**: We conducted a pre/post intervention pilot at a single high‐risk OB clinic. Patients who self‐identified as Black or White race between 24 weeks gestation – 12 weeks postpartum with wage‐earning employment were surveyed at a single timepoint about their employment experiences using the NIH PhenX toolkit and a validated financial stain index. Outpatient appointment adherence was determined via chart review. PROMOTE was offered over three months to all clinical team members. The post‐intervention patient group was recruited using the same criteria. We compared employment experiences, financial strain, and outpatient visit adherence by pre‐ and post‐intervention, self‐identified race, and hourly vs salaried employment using descriptive statistics with SAS 9.4 (SAS Institute Inc., Cary, NC).

**Results**: 42 clinical team members completed the PROMOTE training. 97 patients (50 pre‐ and 47 post‐intervention) completed surveys. Demographics were similar between the two groups (Table 1). There were no differences in financial strain or adherence to outpatient visits between the pre‐ and post‐intervention groups (Table 2). Black participants had higher financial strain than their White counterparts (8.9 (3.7) vs 5.0 (2.6), *p* < 0.0001), as did hourly workers compared to salaried (8.5 (3.8) vs 4.9 (2.5), *p* < 0.0001). Hourly employed respondents were more likely to report a decrease in their work hours during pregnancy (30.8% vs 6.7% for salaried workers, *p* = 0.0058).

**Conclusion**: While we did not detect any pre/post PROMOTE differences in financial strain or adherence to care, we demonstrate the need for Black and hourly workers to be well represented in future studies on employment as a social driver of obstetric outcomes.

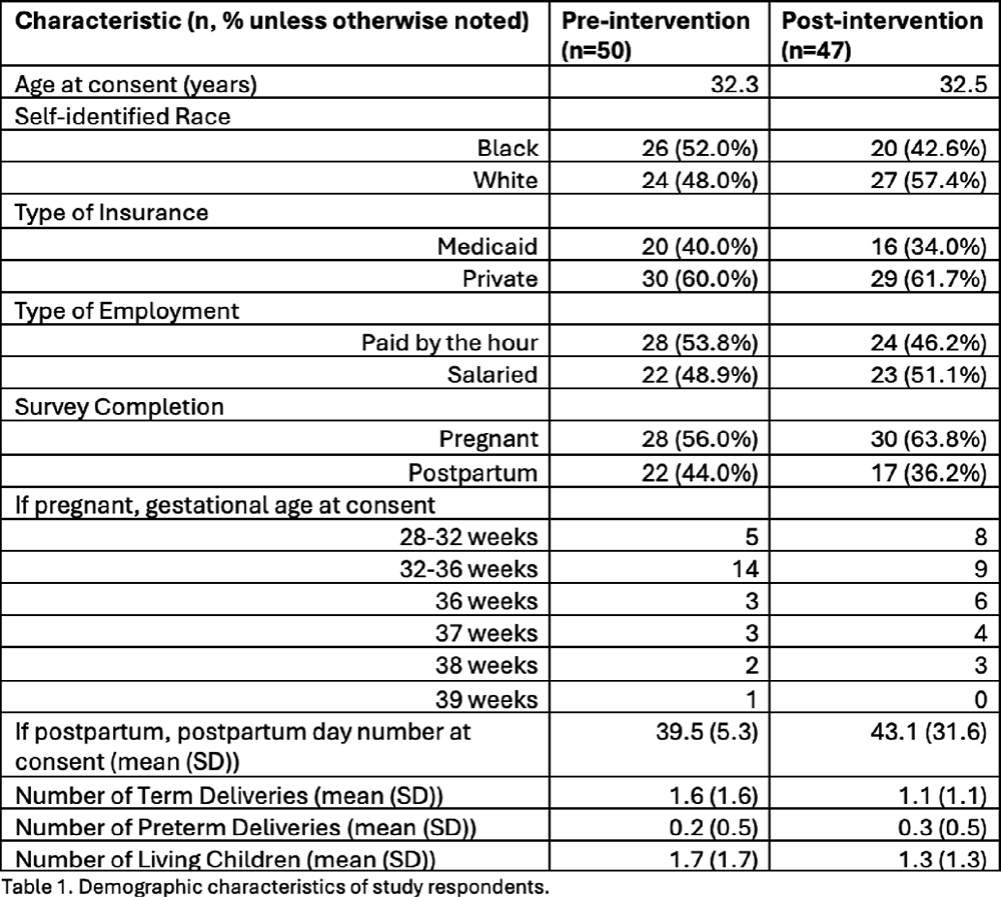

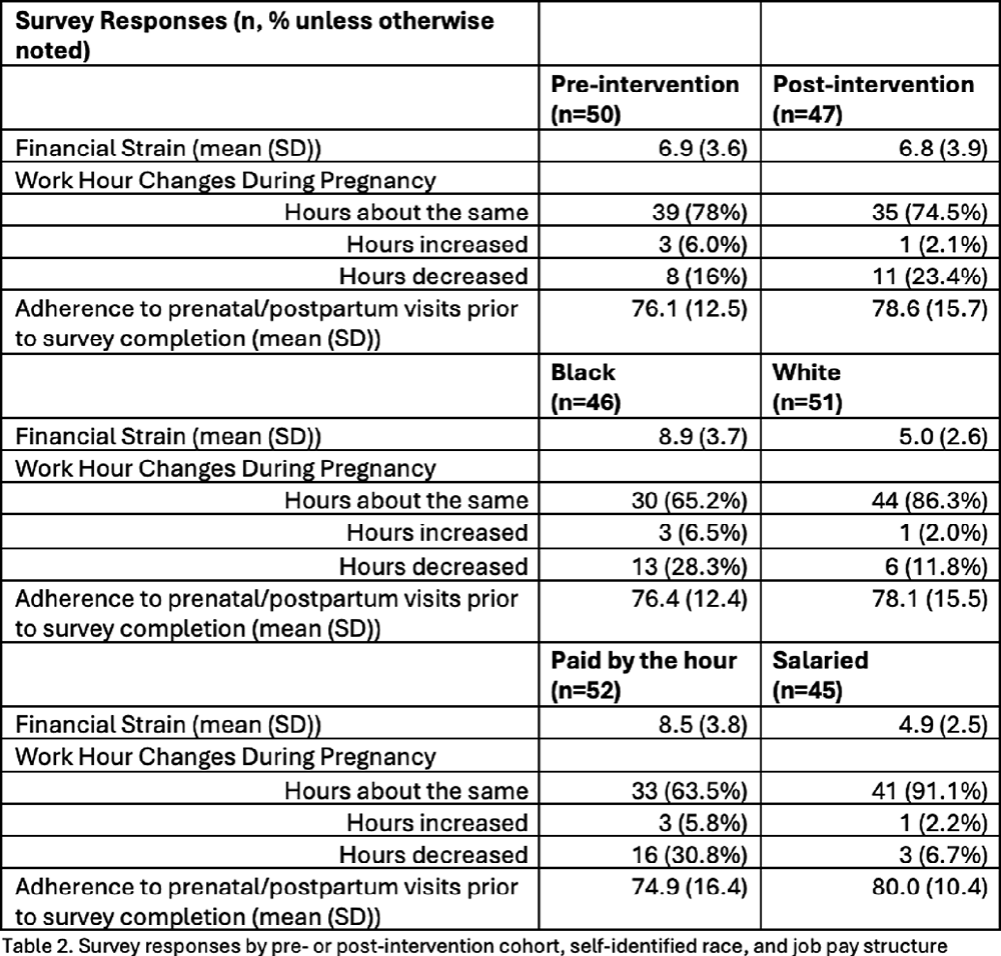

**10:30 AM – 12:00 PM**

## 838 | **A** Retrospective **Review of Fetal Imaging Findings in Confirmed or Suspected Septo‐Optic Dysplasia**

Kelsey C. Mumford^1^; Lauren Meiss^2^; Brooke Lawler^3^; Khyzer B. Aziz^1^; Angie C. Jelin^1^

^1^Johns Hopkins Hospital, Baltimore, MD; ^2^Johns Hopkins, Baltimore, MD; ^3^Johns Hopkins School of Medicine, Baltimore, IA

**Objective**: Prenatal ultrasound is limited as a screening tool for Septo‐Optic Dysplasia (SOD). We predict that prenatal screening ultrasounds have a low sensitivity in detecting SOD. Our objectives will be to evaluate the prevalence of an abnormal cavum septum pellucidum (CSP) and other sonographic findings on prenatal imaging in cases of postnatally diagnosed SOD.

**Study Design**: A retrospective chart review identified 30 patients born between 2016–2025 who have either confirmed or suspected SOD and whose mothers have accessible prenatal imaging. Fetal ultrasounds and MRI scans from maternal charts, and neonatology, endocrinology, neurology, and ophthalmology records in the corresponding pediatric charts were evaluated.

**Results**: Of 30 cases, 13 have clinically confirmed neonatal SOD and 17 have suspected neonatal SOD. Of the 13 confirmed cases, 7 (54%) were suspected to have SOD prenatally based on having an absent/partially absent CSP on fetal ultrasound and/or MRI. Two of those 7 cases had an absent CSP on fetal ultrasound but subsequently had a normal CSP on fetal MRI. The CSP was read as normal on fetal ultrasound for the other 6 (46%) patients. In 6 of the 8 cases with normal CSP readings on fetal ultrasound or MRI, the CSP on postnatal MRI was ultimately read as absent or partially absent. In the other 2 cases, SOD was clinically diagnosed via optic nerve hypoplasia and an empty sella turcica/pituitary abnormalities in one, and via alternate midline structure abnormalities and optic nerve hypoplasia in the other.

**Conclusion**: The postnatal diagnosis of SOD remains difficult to predict prenatally. In 46% of our cases, the CSP had been read as normal prenatally, but was subsequently found to be abnormal on postnatal imaging. In 2 cases, fetal MRI incorrectly read the CSP as present when it had previously been correctly identified as being absent on fetal ultrasound. This highlights the importance of re‐evaluating the CSP across multiple images and imaging modalities, as a single prenatal reading of a normal CSP does not necessarily rule out SOD.

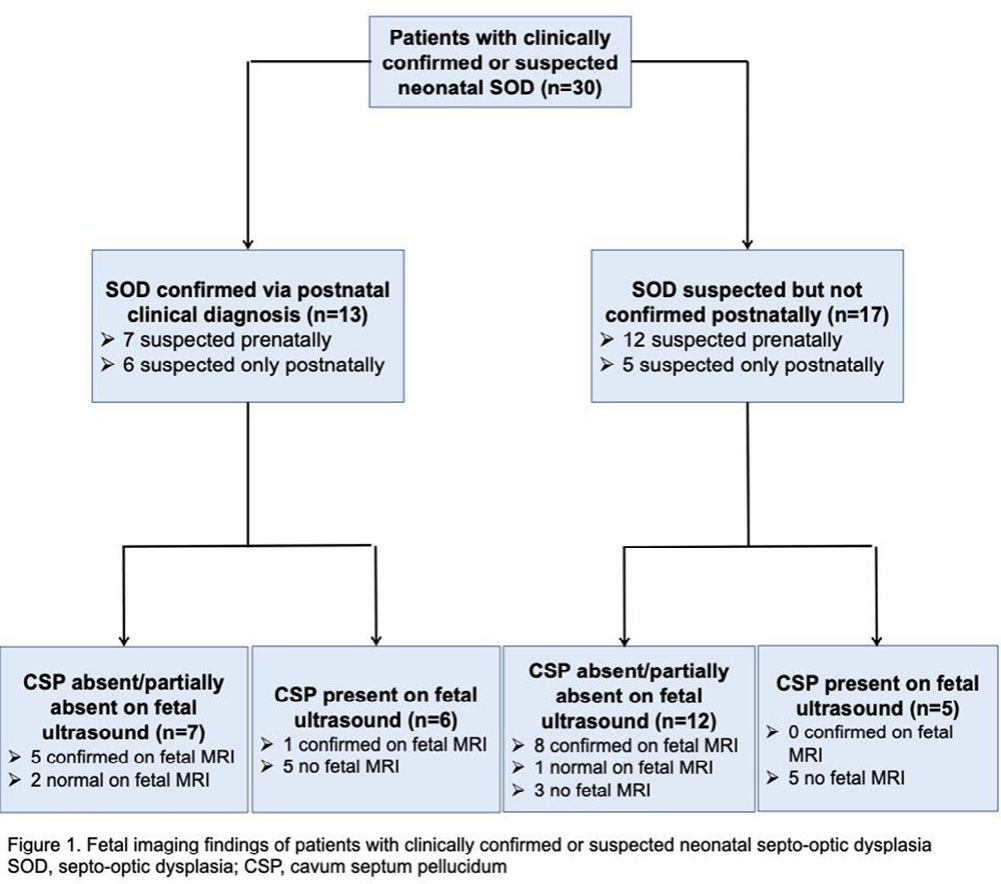

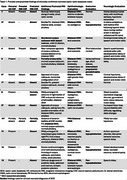

**10:30 AM – 12:00 PM**

## 839 | **Influence of Gestational Age and Maternal Obesity on Cesarean Delivery Rates in Term Inductions**

Khalil M. Chahine; Baha M. Sibai

Division of Maternal‐Fetal Medicine, Department of Obstetrics, Gynecology and Reproductive Sciences, McGovern Medical School at UTHealth Houston, Houston, TX

**Objective**: To compare the rate of cesarean delivery (CD) in low‐risk nulliparous women undergoing induction of labor (IOL) at different gestational ages and across BMI groups.

**Study Design**: This is a secondary, post hoc subgroup analysis of a multicenter randomized controlled trial of IOL at 39 weeks compared with expectant management in low‐risk nulliparous women (ARRIVE). Only participants who were admitted for IOL were included, irrespective of their group allocation in the parent study. Primary outcome was rate of CD. Secondary outcomes were maternal (duration from admission to delivery, operative vaginal delivery, OASIS, preeclampsia or gestational hypertension, chorioamnionitis, postpartum hemorrhage, postpartum infection, shoulder dystocia) and neonatal (birthweight, macrosomia, a composite of adverse outcomes). Modified Poisson and log‐linear regressions were performed to estimate adjusted relative risks; Chi‐square and Mann‐Whitney U tests were used for group comparisons.

**Results**: 3547 patients were included: 215 were induced at 38 weeks, 2551 at 39 weeks, and 781 at ≥40 weeks. The goup induced at 39 weeks was reference. Baseline demographics were similar, except for advanced maternal age (39 v 40), private insurance (39 v 38), tobacco use (39 v 40), previous pregnancy loss (39 v 40), and PROM (39 v 38 and 39 v 40). After adjustment for significant variables, rate of CD was significantly higher in those induced at ≥40 weeks than those induced at 39 weeks (33.7% v 20.9%, respectively, 95% CI 1.59 [1.40–1.80]), irrespective of BMI category (Figure). No significant difference in this rate was noted for IOL at 38 weeks. Furthermore, the rates of any of the secondary maternal and neonatal outcomes were not significantly different between those induced at 39 weeks versus 38 weeks or ≥40 weeks (Table).

**Conclusion**: IOL at ≥40 weeks is associated with a significantly higher cesarean rate across all BMI strata, most pronounced in BMI ≥40, without difference in neonatal outcomes.

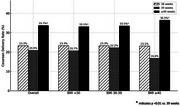

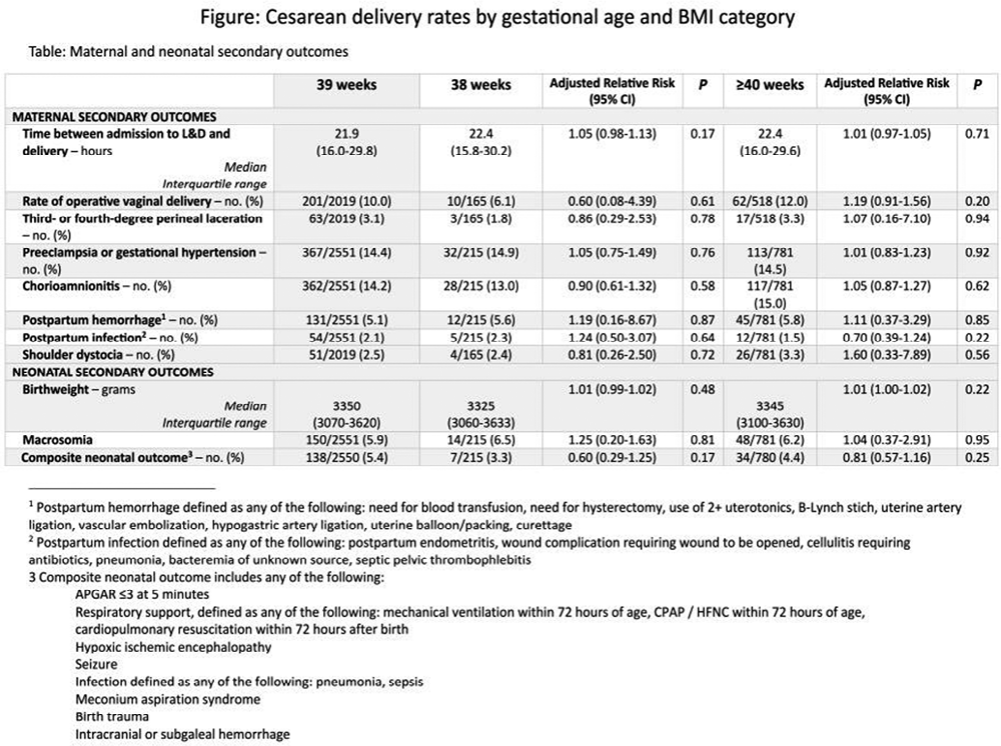

**10:30 AM – 12:00 PM**

## 840 | **Impact of Nonstress Test in Antenatal Surveillance of Fetal Growth Restriction**

Kristen A. Cagino^1^; Sloane A. Stabenow^2^; Beverly Red^2^; Eleazar E. Soto^3^; Angela Glaser^2^; Aaron W. Roberts^1^; Suneet P. Chauhan^4^; Hector Mendez‐Figueroa^5^

^1^Department of Obstetrics, Gynecology and Reproductive Sciences, McGovern Medical School at UTHealth Houston, Houston, TX; ^2^UT Houston, Houston, TX; ^3^University of Texas Health Science Center in Houston, McGovern Medical School, Houston, TX; ^4^Delaware Center for Maternal‐Fetal Medicine of Christiana Care, Newark, DE; ^5^Division of Maternal‐Fetal Medicine, Department of Obstetrics, Gynecology and Reproductive Sciences, McGovern Medical School at UTHealth Houston, Houston, TX

**Objective**: Antenatal surveillance of fetal growth restriction (FGR) includes a combination of Doppler velocimetry of various fetal vessels and nonstress testing (NST). However, due to time constraints and resource availability, some protocols may opt for an ultrasound‐only approach. Our study aimed to determine if the inclusion of NST to antenatal surveillance in FGR decreases the probability of composite neonatal adverse outcomes (CNAO).

**Study Design**: We performed a retrospective cohort study of FGR patients from 2022–2024. In 2023, the institutional protocol for FGR changed from as‐needed NST to NST required for all patient encounters after 32 weeks. Those managed after the change served as the study group; those prior were controls. Ultrasound surveillance for both groups included q3–4‐week growth evaluation and weekly biophysical profile with umbilical artery S/D ratio evaluation. Singleton pregnancies with FGR (EFW or AC < 10% for GA by Hadlock et al curve) who delivered ≥24 weeks were included. CNAO was a composite of Apgar score < 7 at 5 min, mechanical ventilation > 24 hours, neonatal seizure, culture‐proven neonatal sepsis, intracranial hemorrhage, and stillbirth or neonatal death. To assess the impact of the addition of routine NST, a 3:1 group allotment (study: control) was used. Bayesian analysis was used to calculate the posterior relative risk and the posterior probability of risk reduction.

**Results**: There were 903 individuals included for analysis, with 214 in the control and 689 in the study group. Baseline characteristics were similar between groups except for ethnicity, chronic hypertension, and gestational diabetes. CNAO occurred in 4.2% of the control group, and 5.5% in the study group (pRR 1.36, 95% CrI 0.72 – 2.91) with 20% posterior probability of any reduction in CNAO (80% probability of increasing). The number needed to harm in our cohort was 77.

**Conclusion**: In our cohort, the addition of NST to antenatal FGR surveillance did not significantly change CNAO. This information may help develop FGR surveillance protocols while balancing the risk of potential iatrogenic harm.

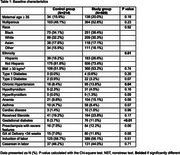

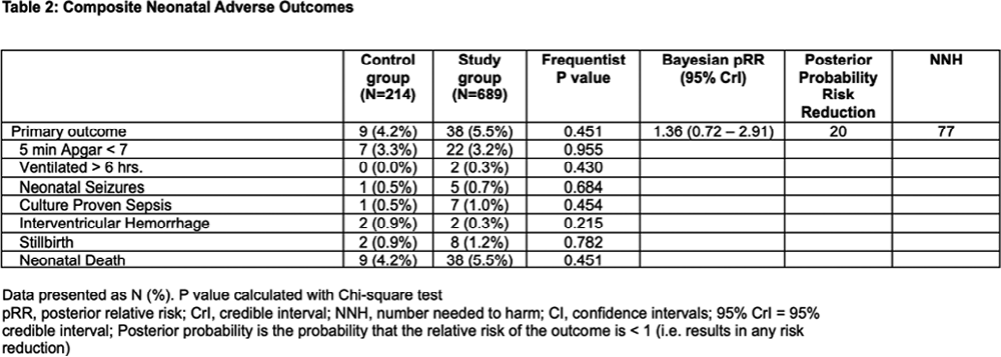

**10:30 AM – 12:00 PM**

## 841 | Gardnerella **Vaginalis Induces Biosynthesis of Higher Order Polyamines: Mechanisms of an Optimal Host Immune Response**

Olha Kholod^1^; Lauren Anton^2^; Kristin M. Klohonatz^2^; Briana Ferguson^2^; Britt A. Goods^1^; Kristin D. Gerson^3^

^1^Dartmouth College, Hanover, NH; ^2^University of Pennsylvania Perelman School of Medicine, Philadelphia, PA; ^3^University of Pennsylvania, Hospital of the university of Pennsylvania, PA

**Objective**: Distinct vaginal metabolomic signatures are associated with an anaerobe‐dominant microbiota, a risk factor for preterm birth (PTB). Among individuals with similar microbiota‐related risk, select metabolites can further risk stratify. Higher order polyamines, which function as anti‐inflammatory molecules in other contexts, are reduced in individuals with an anaerobe‐dominant microbiota who have PTB vs term birth. We hypothesize that an effective host immune response to vaginal anaerobes necessitates biosynthesis of polyamines.

**Study Design**: Vaginal, ectocervical, and endocervical epithelial cells were treated with 10^5^–10^7^ CFU of *Lactobacillus crispatus* (LC) or anaerobe *Gardnerella vaginalis* (GV) for 24h (*n* = 3/condition). Metabolomic profiling of cell culture media was performed and normalized data were analyzed using MetaboAnalyst. Dot plots of log‐transformed data were generated (ggplot2 R package). RNA sequencing was conducted on cells. Differential expression analysis of polyamine metabolism genes was performed using DESeq2.

**Results**: Metabolomic profiling revealed an increase in higher order polyamine spermidine with a concurrent decrease in the precursor arginine in response to GV compared to LC and NT (Figure 1). An increase in intermediates along the pathway was observed, with subtle differences across cell lines. To explore the molecular drivers of these metabolic shifts, we examined enzymes involved in polyamine metabolism. GV exposure upregulated *ODC1*, the rate limiting step in polyamine biosynthesis, and altered expression of additional enzymes including *ARG2*, *AMD1*, *SRM*, *SMOX1*, *PAOX*, and *SAT1* vs controls (Figure 2; all adjusted *p* < 0.05).

**Conclusion**: A vaginal anaerobe implicated in PTB induces biosynthesis of higher order polyamines, which may serve to constrain anaerobe‐induced inflammation. Modulating the host immune response to vaginal anaerobes, either through activation of enzymatic machinery involved in polyamine biosynthesis or vaginal polyamine repletion, may be leveraged as a future therapeutic to mitigate PTB risk in the setting of an anaerobe‐dominant microbiota. 1R01HD114611–01 (KDG)

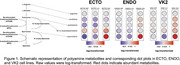

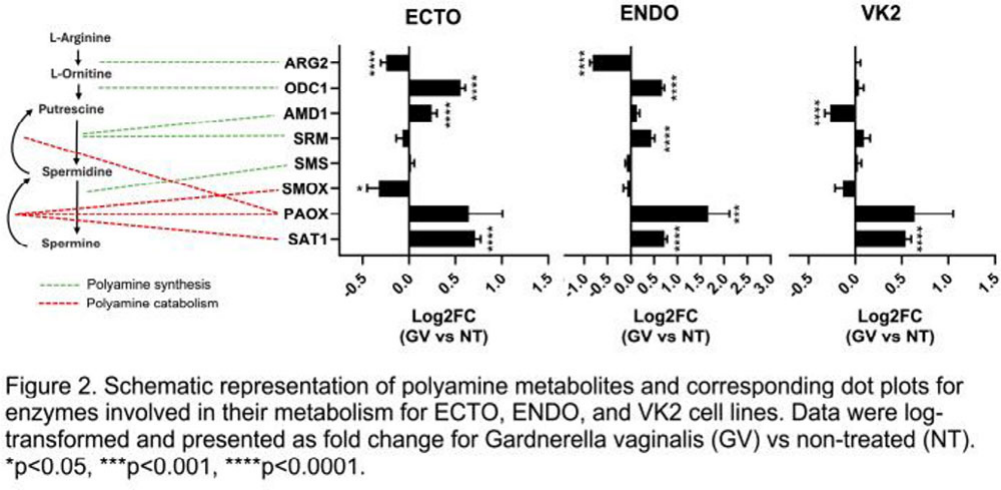

**10:30 AM – 12:00 PM**

## 842 | Thromboelastography **Values in Healthy Pregnant Individuals Compared to the Non‐Pregnant Population**

LaKeisha C. Majette^1^; Rebecca Purvis^2^; Kaitlin Warta^3^; Rachel Parker^4^; Geoffrey Slaughter^5^; William M. Johnstone, Jr.^5^

^1^Novant Health New Hanover Regional Medical Center, Wilmington, NC; ^2^University of Tennessee Health Science Center College of Medicine‐ Knoxville, Knoxville, TN; ^3^Novant Health NHRMC, Wilmington, NC; ^4^Lexington Medical Center, Columbia, SC; ^5^New Hanover Regional Medical Center, Wilmington, NC

**Objective**: Thromboelastography (TEG) is a method for assessing clotting and fibrinolysis that allows for real‐time assessment of a patient's coagulative status; based on the non‐obstetric population. The obstetric population faces unique risks of hemorrhage requiring blood product replacement in which a TEG would be helpful in guiding management. The objective of this study is to understand the pregnancy‐specific values for TEG parameters and directly compare these values to reference values in the non‐pregnant population.

**Study Design**: Data were synthesized from a prospective, cross‐sectional study of healthy pregnant individuals, in which TEG was performed at various gestational time points and postpartum. After collection, we calculated the mean and 95% confidence intervals for TEG values (Reaction time (R), Clot Kinetics (K), Clot Angle (angle), Maximum amplitude (MA), Percent lysis at 30 minutes (Ly30), and functional fibrinogen). We utilized the Wilcoxon Sign Rank Test to compare the calculated means to the established TEG reference values for non‐pregnant individuals.

**Results**: Compared to non‐pregnant individuals, pregnancy is associated with a marked hypercoagulable shift in TEG parameters. Specifically, R and K times are significantly lower (e.g., R: 4.69–5.05 min vs. 5.0–10.0 min, *p* = < 0.0001; K: 1.18–1.29 min vs. 1.0–3.0 min, *p* = < 0.0001). Angle and MA are increased (Angle: 70.53–72.38° vs. 53.00–72.00°, *p* = < 0.0001; MA: 68.90–70.65 mm vs. 50.00–70.00 mm, *p* = < 0.0001). Fibrinolysis (Ly30) is reduced in pregnancy. The pregnant individual's point estimates are consistently located at the extremes of the literature's confidence intervals.

**Conclusion**: Our findings suggest evidence of hypercoagulable state in pregnant individuals with statistical differences in TEG parameters R, K, Angle, MA, LY30, and functional fibrinogen compared to the non‐pregnant state. These findings suggest that pregnant individuals are afforded a hypercoagulable protective state, which should be considered in the setting of assessing coagulative status and blood product transfusion during pregnancy.

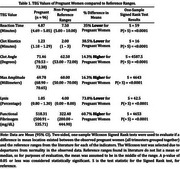

**10:30 AM – 12:00 PM**

## 843 | **Misdated or Just Small: Study of Fetal Growth Restriction Outcomes in Pregnancies With Suboptimal Dating**

Lama R. Noureddine^1^; Angela Hopf^2^; Lisa N. Gittens‐Williams^2^; Joseph J. Apuzzio^2^; Shauna F. Williams^2^

^1^Rutgers New Jersey Medical School, Newark, NJ; ^2^Rutgers NJMS, Rutgers NJMS, NJ

**Objective**: To assess abnormal umbilical artery Doppler (UAD) and neonatal outcomes in fetal growth restriction (FGR) when pregnancy dating is suboptimal.

**Study Design**: This was a retrospective cohort study of patients with FGR by ultrasound who delivered between January 2020 and March 2025 after 20 weeks. The cohorts were divided into optimally dated (Group 1: dating by first trimester ultrasound (1TU) or second trimester ultrasound (2TU) congruent with LMP) and suboptimally dated (Group 2: dating by third trimester ultrasound (3TU) or 2TU incongruent with LMP). The primary outcome, abnormal UAD was defined as elevated systolic to diastolic ratio (S/D), pulsatility index (PI), or resistance index (RI) greater than 95%, or absence or reversal in diastolic flow. Secondary outcomes included FGR resolution, neonatal weight, non‐congruent Ballard score, small for gestational age (SGA) neonates, NICU admission, and an adverse composite neonatal outcome (CNO). Fisher's exact, chi‐squared, and Mann‐Whitney U tests were used.

**Results**: Among 282 patients, 226 (80.1%) were optimally dated (Group 1) and 56 (19.9%) were suboptimally dated (Group 2). Within Group 1, 123 (54.4%) were dated by 1TU and 103 (45.6%)  by 2TU congruent with LMP. In Group 2, 36 (64.3%) were dated by 2TU incongruent with LMP, and 20 (35.7%) by 3TU. The rate of abnormal UAD was significantly lower in Group 1 (27 [11.9%] vs 16 [28.6%], *p* = 0.002). However, Group 1 was less likely to have resolution of FGR, (45 [19.9%] vs 44 [78.6%], *p* = < 0.0001). Neonatal weight (2600g [2295–2843] vs 2540g [2181–2871], *p* = 0.66) and Ballard scores (38 [38–39] vs 38 [38–38], *p* = 0.46) were similar. There were no significant differences in the rates of SGA neonates (112 [49.6%] vs 33 [58.9%], *p* = 0.21), NICU admission (62 [27.4%] vs 19 [33.9%], *p* = 0.49), or adverse CNO (62 [27.4%] vs 14 [25%], *p* = 0.49).

**Conclusion**: Given that the rate of abnormal UAD was significantly higher in pregnancies with suboptimal dating and that neonatal outcomes did not differ from well dated pregnancies, a diagnosis of FGR should be managed and surveilled similarly regardless of pregnancy dating.

**10:30 AM – 12:00 PM**

## 844 | **SMM** Rates **in PAS – Time to Revisit Our SMM Criteria**

Laura Diab^1^; Diego Aviles^2^; Edgar A. Hernandez‐Andrade^1^; Joe Haydamous^3^; Zakaria Doughan^4^; Sean C. Blackwell^1^; Baha M. Sibai^1^; Farah H. Amro^1^

^1^Division of Maternal‐Fetal Medicine, Department of Obstetrics, Gynecology and Reproductive Sciences, McGovern Medical School at UTHealth Houston, Houston, TX; ^2^Division of Gynecology Oncology, Department of Obstetrics, Gynecology and Reproductive Sciences, McGovern Medical School at UTHealth Houston, Houston, TX; ^3^Department of Obstetrics, Gynecology and Reproductive Sciences, McGovern Medical School at UTHealth Houston, Department of Obstetrics and Gynecology, McGovern Medical School at UT Health, Houston, TX; ^4^Department of Obstetrics and Gynecology, McGovern Medical School at UT Health, Houston, Houston, TX

**Objective**: Severe maternal morbidity (SMM) is used as a quality metric. Current ACOG‐SMFM criteria are not tailored to placenta accreta spectrum (PAS) since they include expected morbidity such as transfusion. Recently, PAS‐specific SMM criteria were proposed. Our objective is to compare SMM rates in PAS using newly proposed PAS‐specific versus ACOG‐SMFM criteria.

**Study Design**: A retrospective study at Level IV center (Jan 2015 to Jun 2025) of patients with ultrasound suspected PAS. Different PAS management approaches are utilized at our institution—cesarean hysterectomy (CH), delayed hysterectomy (DH) and leaving placenta in situ for uterine preservation (LPIS‐UP). Patients undergoing CH or DH were included with pathologic confirmation of PAS, while LPIS‐UP cases required intraoperative confirmation. Maternal outcomes were collected by chart review. SMM was compared between ACOG‐SMFM and PAS‐specific criteria.

**Results**: A total of 173 met inclusion criteria: 129 had CH, 30 DH, and 14 LPIS‐UP. Rate of SMM was higher using ACOG‐SMFM criteria (71%, 123/173) compared to PAS‐specific criteria (20%, 35/173; *p* < 0.05). The relative risk of SMM was 3.52 (95% CI: 2.6–4.8), indicating that ACOG‐SMFM definition identified over three times as many cases. SMM defined by ACOG‐SMFM was primarily driven by hemorrhage, while PAS‐specific SMM was driven by transfusion ≥8 units pRBCs (Table 1). When stratified by management approach, rates of SMM under ACOG‐SMFM vs. PAS‐specific criteria were CH – 71% vs. 22%; DH – 87% vs. 23%; LPIS‐UP – 43% vs. 0% (*p* = 0.02; Figure 1). Comparisons across management strategies showed significant differences using ACOG‐SMFM criteria (70% vs. 87% vs. 38%, *p* = 0.01), but not with PAS‐specific criteria (22% vs. 23% vs. 0%, *p* = 0.13).

**Conclusion**: Applying PAS‐specific severe maternal morbidity (SMM) criteria resulted in 51% reduction in reported SMM rate, redirecting focus toward complications uniquely associated with PAS. These findings support adoption of PAS‐specific criteria in place of ACOG‐SMFM definition, enabling more meaningful comparisons of outcomes across centers and management strategies.

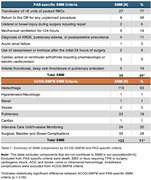

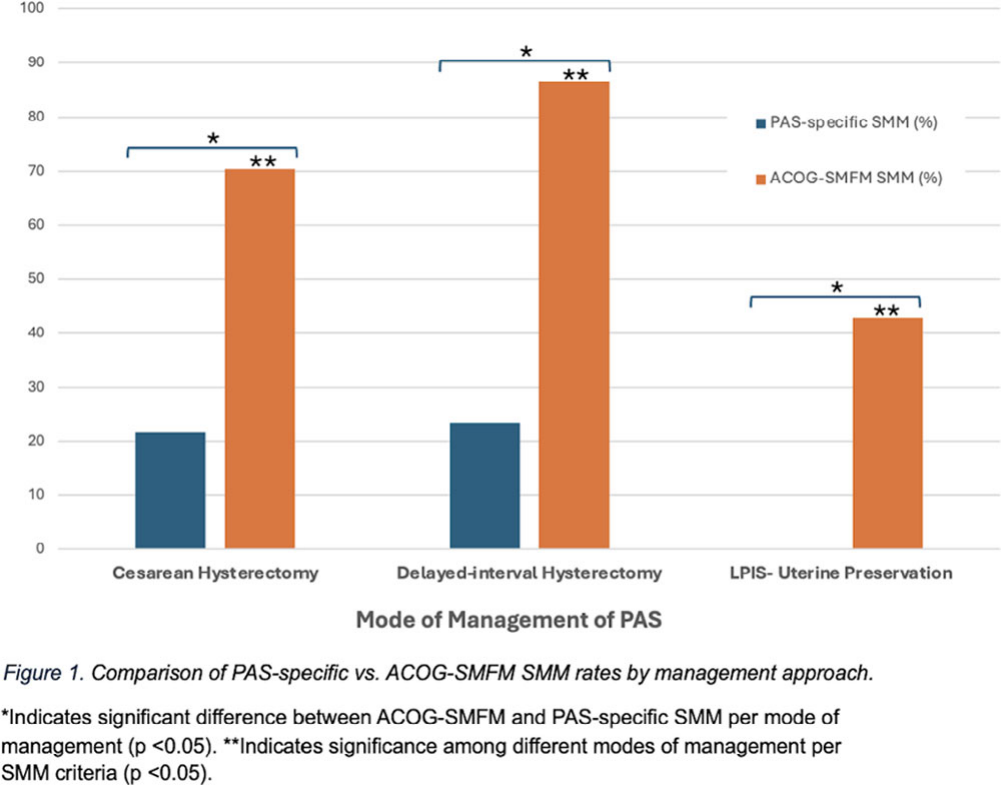

**10:30 AM – 12:00 PM**

## 845 | **Prenatal Care Utilization in a Novel Hybrid Prenatal Care Model: Implications in Chronic Disease**

Laura Peyton Ellis^1^; Elizabeth Krans^2^; David M. Haas^3^; Thuy Nguyen^4^; Basil Issac^5^; Chloe Qi^4^; Amy Jiao^4^; Alex Peahl^6^

^1^UConn Health Obstetrics and Gynecology, Farmington, CT; ^2^UPMC Magee‐Womens Pregnancy and Women's Recovery Center, Pittsburgh, PA; ^3^Indiana University, Indiannapolis, IN; ^4^University of Michigan School of Public Health, Ann Arbor, MI; ^5^University of Michigan School of Economics, Ann Arbor, MI; ^6^University of Michigan Department of Obstetrics & Gynecology, Ann Arbor, MI

**Objective**: The American College of Obstetricians and Gynecologists “Tailored Prenatal Care Delivery for Pregnant Individuals ” recommends personalized prenatal care. Yet, evidence for tailored care focuses on low‐risk patients. This project assessed the effect of tailored prenatal care on patients with chronic conditions.

**Study Design**: Retrospective cohort study of electronic health record data from three academic hospitals that implemented tailored prenatal care during the COVID‐19 pandemic: two sustained changes (“intervention”), and one did not (“control”). Chronic conditions (CC) included asthma, type 2 diabetes mellitus, substance use disorder, and chronic hypertension. Primary outcomes were planned (prenatal visits) and unplanned (ED/triage) utilization. Secondary outcomes were completion of recommended prenatal tests and timeliness of test completion. Difference‐in‐difference analysis compared pregnancies from pre‐COVID Jan ’17 to Feb ’20 to post‐COVID Jun ’20 to Dec ’23 excluding a washout period (Mar to May ’20). The difference in difference in difference (DDD) coefficient was reported for all outcomes.

**Results**: In the study cohort, chronic conditions were common (50% control, 27% intervention, *n* = 49, 264). Post intervention, both groups experienced a similar decrease in planned utilization (no CC: –1.49, CC: –1.31; DDD = 0.18, *p* = 0.57). The increase in unplanned utilization was not statistically significant, and was equivalent between groups (no CC: 0.51, CC: 0.66; DDD = 0.151, *p* = 0.348). Patients with chronic conditions had lower completion of services after the intervention (no CC: 0.15, CC: –0.15; DDD: –0.301, *p* = 0.35). Both groups had an increase in timely completion of services, but the increase was smaller for those with CC (no CC: 0.621, CC: 0.147; DDD: –0.475, *p* = 0.35).

**Conclusion**: For patients with CC, implementation of tailored prenatal care reduced planned care utilization, but did not increase in unplanned utilization. Patients with CC did not see the same benefit for timely completion of services. More work is needed to understand how tailored care models affect maternal and neonatal outcomes.

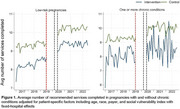

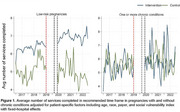

**10:30 AM – 12:00 PM**

## 846 | Postpartum **Diabetes Screening in Patients With Gestational Diabetes Enrolled in Remote Blood Pressure Monitoring**

Lauren Walheim^1^; Lisbet S. Lundsberg^2^; Jennifer F. Culhane^2^; Nicole Spaulding^3^; Christine Coffey^3^; Elizabeth Forbes^3^; Caitlin Partridge^2^; Katherine H. Campbell^4^; Anna Denoble^5^

^1^Yale School of Medicine, Yale School of Medicine – Yale University, CT; ^2^Department of Obstetrics, Gynecology, and Reproductive Sciences, Yale School of Medicine, New Haven, CT; ^3^Yale New Haven Hospital, New Haven, CT; ^4^Division of Maternal Fetal Medicine, Yale School of Medicine, New Haven, CT; ^5^Yale School of Medicine, New Haven, CT

**Objective**: Remote blood pressure monitoring (RBPM) in patients with hypertensive disorders of pregnancy (HDP) improves postpartum (PP) care engagement. We assessed whether RBPM participation improves PP screening (2‐hour glucose tolerance test [2h GTT] and/or hemoglobin A1C [A1C]) for non‐hypertensive disorders, such as gestational diabetes (GDM) and pre‐gestational diabetes mellitus (DM).

**Study Design**: Retrospective study of patients in PP RBPM program from 11/1/2022–6/30/2024. Patients with chronic hypertension or HDP are eligible for RBPM. Patients have telehealth visits for BP monitoring until they graduate (BP normalizes off medication or 12 weeks PP). Patients were grouped by RBPM engagement: 1) declined or enrolled but never attended; 2) attended ≥ 1 visit but did not graduate; 3) graduated. Patient characteristics and laboratory values were extracted from health records. Patient characteristics were compared by three groups; no diabetes, GDM and DM using chi‐square or Fisher's exact tests. We conducted sub‐analyses of patients with GDM and DM. Primary outcomes were completion of 2h GTT by 12 weeks PP for GDM and A1C by 1 year for GDM and DM. Secondary outcome was time to A1C. Logistic regression determined the odds of the primary outcomes and ANOVA for secondary outcome.

**Results**: 1, 160 patients were eligible for RBPM;165 had GDM and 120 had DM. Among GDM and DM patients, 121(42.5%) were Group 1, 60 (23.3%) Group 2, 104(36.5%) Group 3. Of the 165 with GDM, 37(22.4%) completed 2h GTT; 9(14.5%), 5(15.6%) and 23(32.4%) in Group 1, 2 and 3 respectively. Odds of completing 2h GTT were significantly higher for RBPM graduates (Group 3 OR 2.82; 95% CI 1.19–6.69). No significant difference between partial and no engagement (Group 2 OR 1.09; 95% CI 0.33–3.58). No significant difference in odds of A1C completion for GDM or DM and no difference in time to A1C (Table 2).

**Conclusion**: RBPM engagement improved DM screening within 12 weeks PP for GDM patients. While these patients may be more likely to complete recommended care, these findings suggest remote PP programs may improve PP care beyond HTN management.

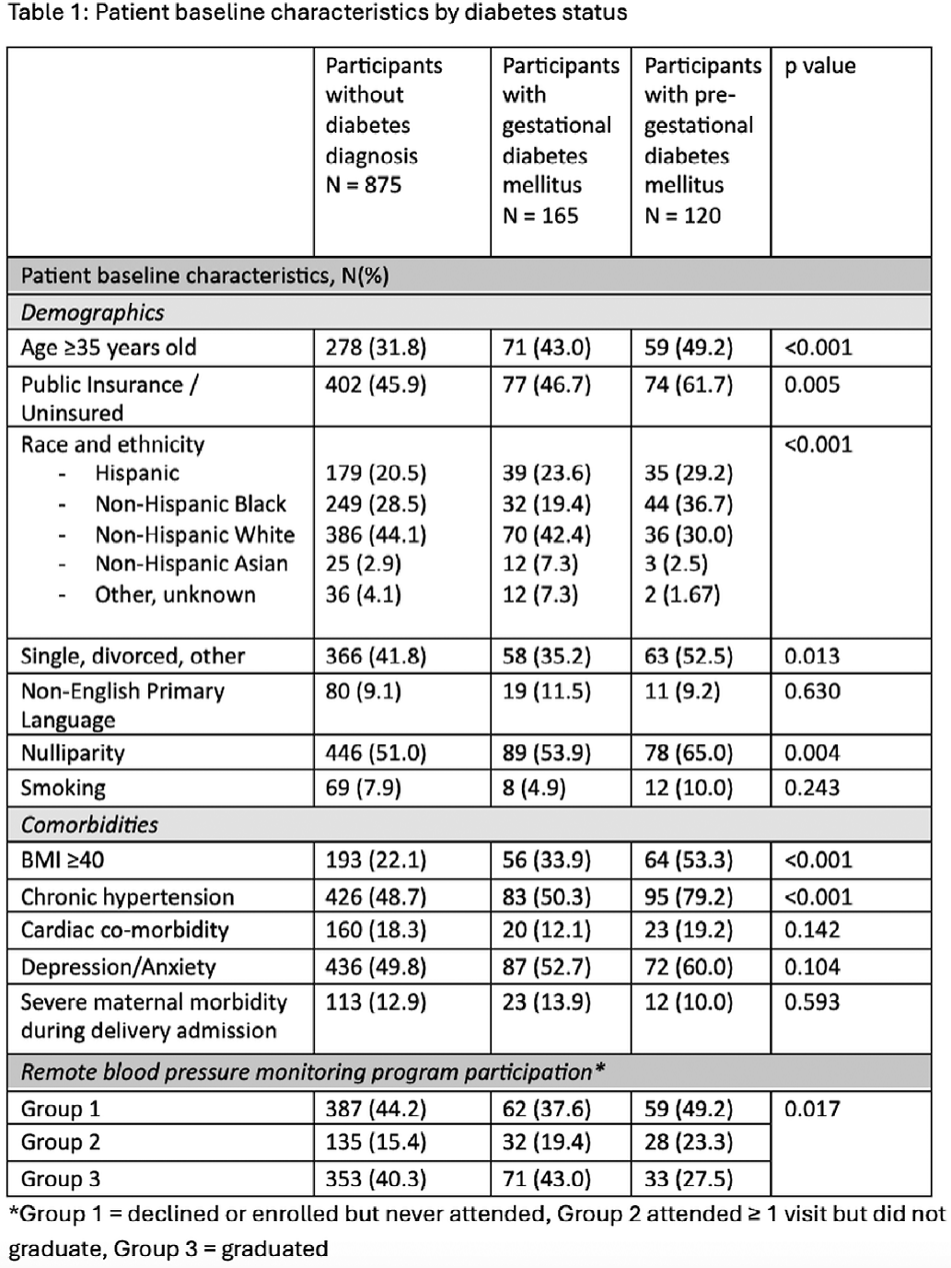

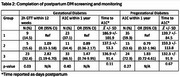

**10:30 AM – 12:00 PM**

## 847 | **Non‐**Invasive **Diagnosis of Symptomatic Congenital CMV Infection: Biomarkers from the MFMU Trial of Hyperimmune Globulin**

Lierni Ugartemendia^1^; Brenna L. Hughes^2^; Robert Suchting^3^; Gail Demmler‐Harrison^4^; Laura Goetzl^1^

^1^Division of Maternal‐Fetal Medicine, Department of Obstetrics, Gynecology and Reproductive Sciences, McGovern Medical School at UTHealth Houston, Houston, TX; ^2^Department of Obstetrics and Gynecology, Duke University School of Medicine, Duke, NC; ^3^Faillace Department of Psychiatry and Behavioral Sciences, McGovern Medical School at UTHealth Houston, Houston, TX; ^4^Department of Pediatrics and Pathology & Immunology, Baylor College of Medicine, Houston, TX

**Objective**: Prenatal diagnosis of congenital cytomegalovirus (CMV) infection requires delaying amniocentesis following primary infection. We hypothesized that fetal neural‐derived extracellular vesicles (fCNSEVs) may allow for earlier non‐invasive diagnosis of fetal infection as well as additional prognostic information.

**Study Design**: We purified fCNSEVs from the serum of pregnant women with primary CMV infection (GA< 28 weeks) enrolled in the MFMU‐CMV trial. 51 women received hyperimmune globulin (IG) and 47 placebo. Neonates were categorized into 3 groups: uninfected (UNI, *n* = 44), asymptomatic (ASY, *n* = 25), symptomatic (SYM, *n* = 29), and infection was confirmed after birth. We measured synaptopodin (SYNPO) and BCL2‐associated transcription factor 1 (BCLAF1) from fCNSEVs at baseline (GA< 27 weeks), and 8.3 ± 0.7 weeks later. Linear mixed‐effects models fit each log‐transformed biomarker as a function of time by symptom group interaction, adjusting for main effects. Interpretation relied on estimated marginal contrasts and slopes. Follow‐up models evaluated the effect of IG treatment.

**Results**: Baseline SYNPO levels were significantly higher in SYM relative to UNI (+24.7%, *p* < .001) and ASY (+15.8%, *p* = .043). But there were not differences between ASY and UNI (+7.7%, *p* = .354). Similarly, BCLAF1 were significantly higher in SYM relative to UNI (+20%, *p* = .025) and ASY (+27.2%, *p* = .012), without differences between ASY and UNI (+3.3%, *p* = .811). 8 weeks post enrollment, SYNPO decreased by ‐20.2% in SYM (*p* < .001, Fig 1A) but no changes in BCLAF1 were observed (*p* = .092, Fig 1B).

**Conclusion**: fCNSEV biomarkers may allow non‐invasive identification of those fetuses at highest risk for symptomatic infection, both for patient counseling and for potential novel anti‐viral treatments. Serial biomarker measurements are associated with neonatal outcome and may prove useful tracking response to therapy and/or fetal rebound from initial infectious injury through neuroplasticity.

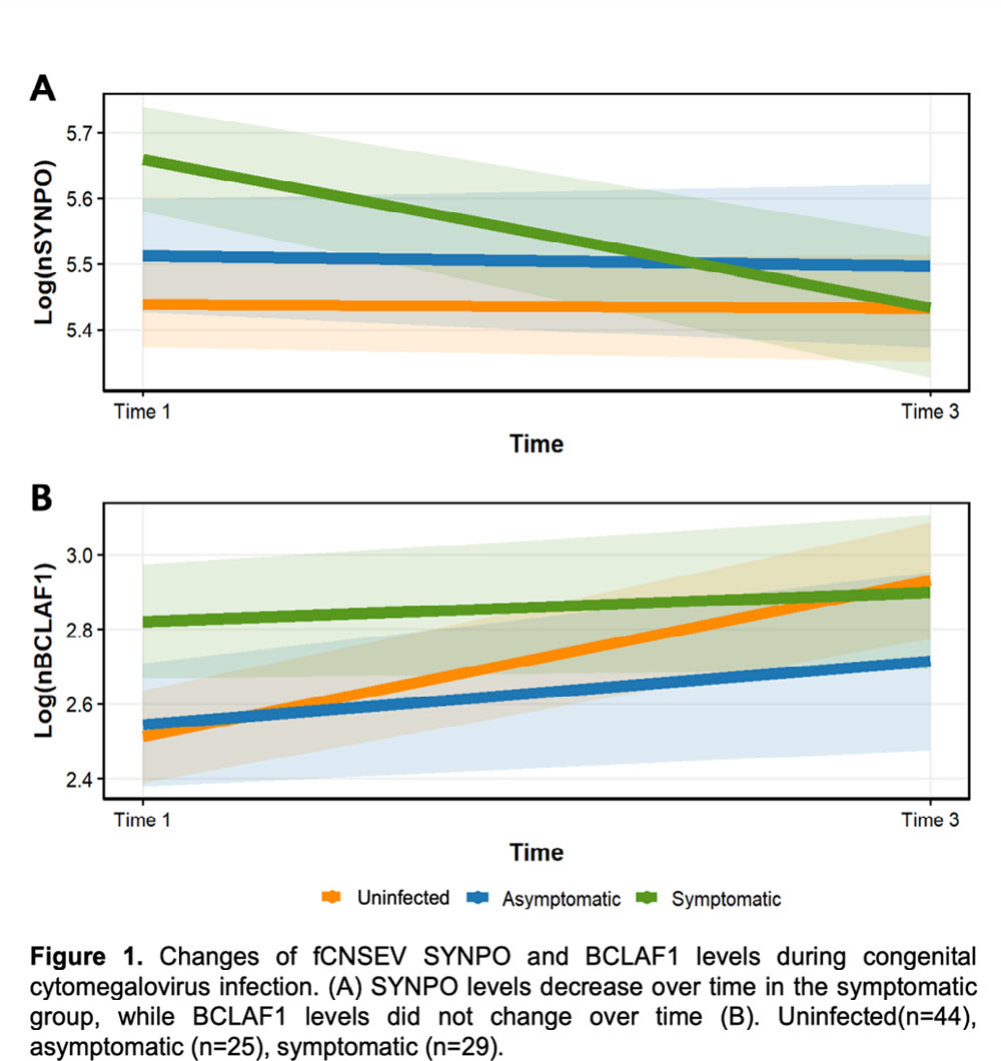

**10:30 AM – 12:00 PM**

## 848 | **Changes of Electrocardiogram Parameters in Pregnancies Affected and Unaffected by Cardiovascular Dysfunctions**

Lihong Mo^1^; Arielle Yoo^2^; Gizem Akildiz^3^; Imo Ebong^4^; Shantanu Milind Joshi^5^; Philip Strong^1^; Sonul Gupta^1^; Uma Srivasta^4^; Herman Hedriana^1^; Chen‐Nee Chuah^2^

^1^Department of Obstetrics and Gynecology, UC Davis, Sacramento, CA; ^2^Electrical and Computer Engineering, UC Davis, Davis, CA; ^3^School of Medicine, UC Davis, Sacramento, CA; ^4^Division of Cardiovascular Medicine, UC Davis, Sacramento, CA; ^5^Computer Science, UC Davis, Davis, CA

**Objective**: Pregnancy induces physiologic cardiovascular adaptations, but electrocardiogram (ECG) changes remain poorly characterized. We hypothesized that cardiac volume and compliance changes alter ECG parameters throughout pregnancy, with distinct patterns in pregnancies affected by cardiovascular dysfunction. This study aims to establish trends in four ECG parameters (heart rate, PR‐interval, QRS duration, and QTc) during pregnancy and compare these trends between pregnancies with and without cardiovascular complications.

**Study Design**: This is a retrospective case‐control study of a database that includes 26, 453 ECGs on 7, 637 pregnant individuals aged 18–50 (2011–2024) at a single tertiary center. After excluding patients with pre‐existing cardiac morbidity or hypertensive disorders of pregnancy, 3, 032 ECGs from 2, 407 individuals were included to establish normative trends. Ten individuals with heart failure (pre‐existing or peripartum cardiomyopathy) (Figure 1) and 13 patients with preeclampsia who had ECGs from ≥2 gestational periods (pre‐pregnancy, during pregnancy, or postpartum) were separately analyzed (Figure 2).

**Results**: In unaffected pregnancies, heart rate increased through the first and second trimesters, plateaued at 30–35 weeks, and declined postpartum to pre‐pregnancy levels. PR‐interval, QRS duration, and QTc remained stable. In contrast, patients with heart failure exhibited elevated QRS duration, QTc, and PR interval (Figure 1). Patients with preeclampsia only demonstrated elevated QTc and heart rate at 35 weeks gestational age (Figure 2).

**Conclusion**: Cardiovascular dysfunction, particularly heart failure, may disrupt physiologic ECG patterns in pregnancy, manifesting as prolonged QRS and QTc. Although sample size was limited in patients with cardiovascular dysfunction, these findings suggest ECG parameters may help identify pregnancies complicated by cardiac pathology, warranting further investigation into the prognostic utility of ECG beyond clinical parameters.

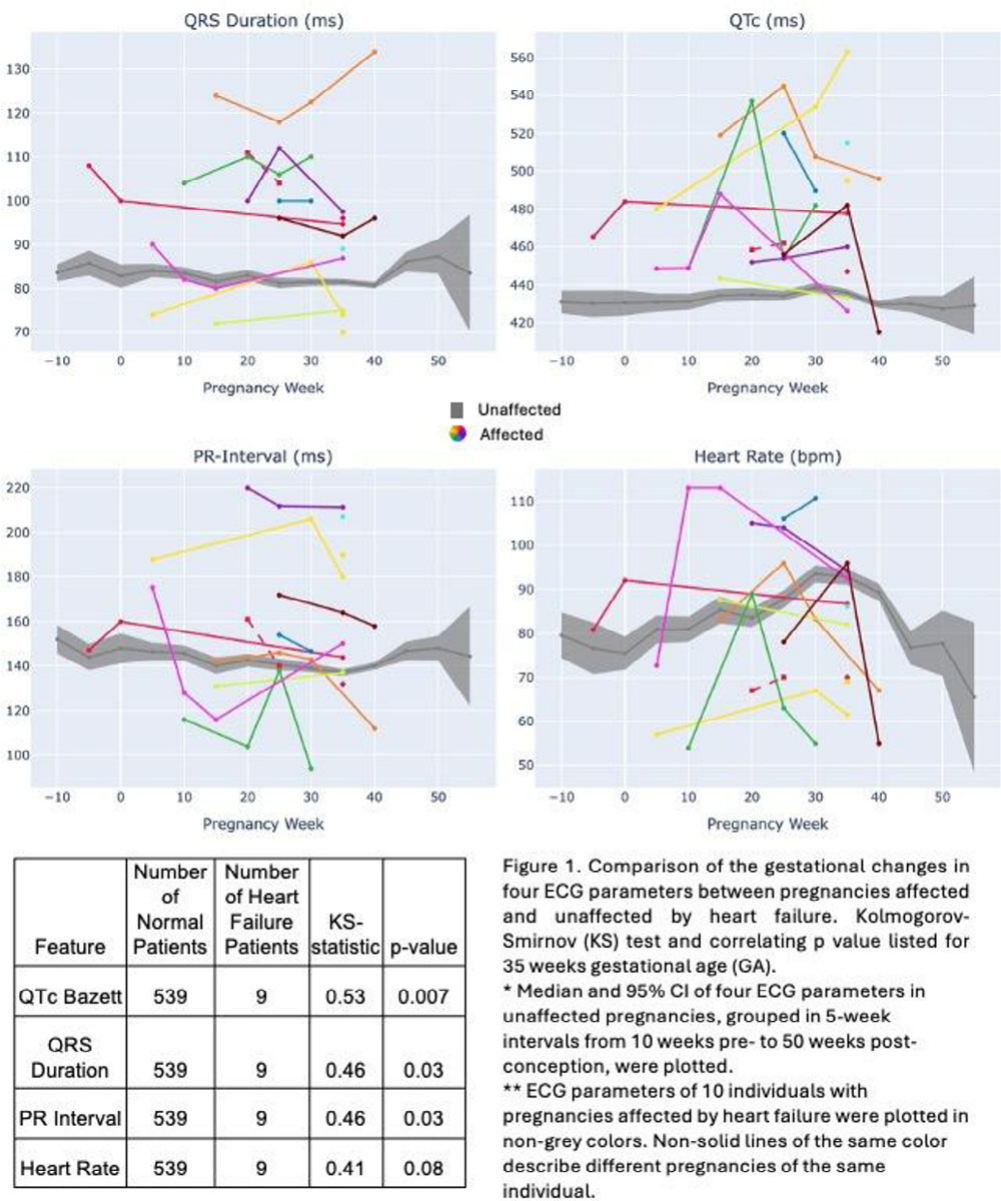

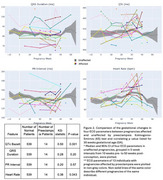

**10:30 AM – 12:00 PM**

## 849 | Hypertensive **Outcomes in Twin Pregnancies with Elevated Blood Pressure Defined by AHA 2017 Classification**

Lior Heresco^1^; Tzipi Hornik‐Lurie^2^; Or Touval^3^; Dorit Ravid^3^; Michal Kovo^4^; Tal Biron‐Shental^1^

^1^Meir Medical Center, Kfar Saba, HaMerkaz; ^2^Data research department at the Research Authority, Meir Medical Center, Kfar Saba, HaMerkaz; ^3^Department of Obstetrics and Gynecology, Meir Medical Center, Kfar Saba, HaMerkaz; ^4^Shamir Medical Center, Israel

**Objective**: Twin pregnancies are associated with an increased risk of hypertensive disorders of pregnancy (HDP). In 2017, the American Heart Association (AHA) redefined elevated blood pressure (BP) as systolic BP (SBP) of 120–129 mmHg with diastolic BP (DBP) < 80 mmHg. However, data on the implications of elevated BP within this range among twin gestations remain limited. This study aimed to evaluate the association between elevated BP‐ measured preconceptionally or in the first trimester—and the risk of HDP in twin pregnancies.

**Study Design**: This retrospective cohort study included all nulliparous women with twin pregnancies from 2010 to 2023 who had at least one BP measurement from one year before conception until 13 weeks of gestation. Chronic hypertension was an exclusion criterion. Patients were classified according to the AHA 2017 BP classification. Maternal and neonatal outcomes were compared between the normotensive and elevated BP groups. The composite HDP outcome included preeclampsia (with and without severe features), eclampsia and HELLP syndrome. A multivariable logistic regression model adjusted for maternal age, aspirin use, BMI, PGDM and chorionicity was utilized.

**Results**: A total of 1, 070 patients were classified as normotensive and 50 as having elevated BP. Baseline characteristics were similar between groups. The elevated BP group had significantly higher rates of preeclampsia without severe features (22% vs. 9.8%, *p* = 0.017) and composite HDP (36% vs. 16.8%, *p* = 0.002). In adjusted analysis, both elevated BP and PGDM were independently associated with increased odd of composite HDP outcome: aOR 2.89 (95% CI 1.57–5.31), aOR 1.96 (95% CI 1.17–3.28), respectively, *p* < 0.001 for both.

**Conclusion**: Elevated BP in the preconception and first trimester period is associated with a nearly threefold increased risk of HDP in twin pregnancies.

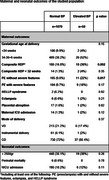

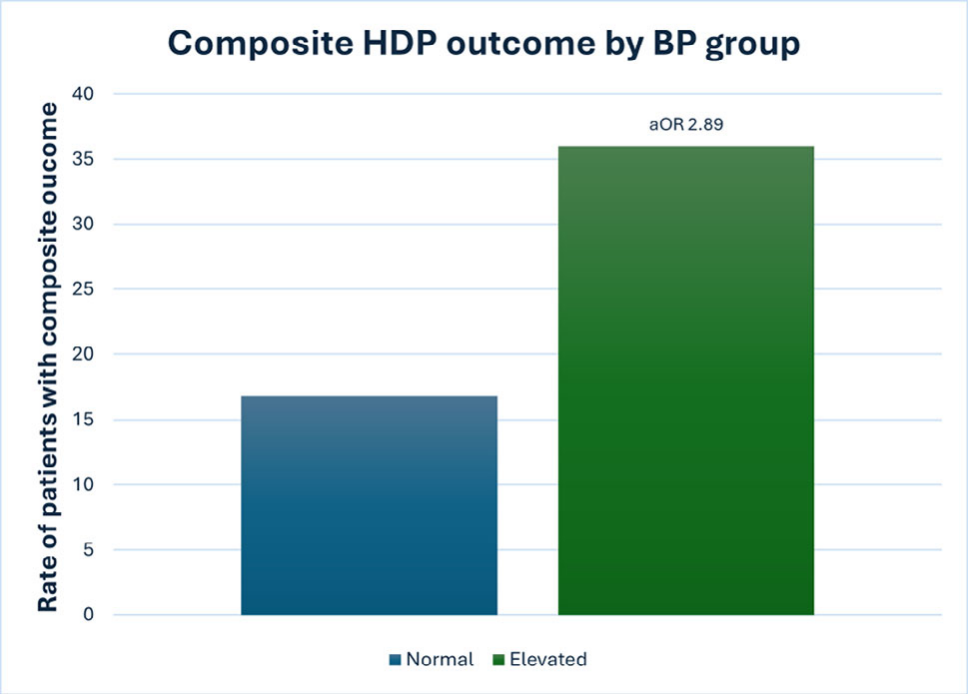

**10:30 AM – 12:00 PM**

## 850 | **Direct** Dispensation **of Folic Acid Supplements is Associated With Significant Reduction in Neural Tube Defects**

Lisa R. Thiele^1^; Anjali Nambiar^1^; Jessica E. Pruszynski^2^; Carrie Berge^3^; Elaine L. Duryea^2^; David B. Nelson^2^; Catherine Y. Spong^2^

^1^UT Southwestern, Dallas, TX; ^2^University of Texas Southwestern Medical Center, Dallas, TX; ^3^Parkland Health, Dallas, TX

**Objective**: To evaluate the impact of a public health effort to directly dispense prenatal vitamins to patients in a medically underserved community on the incidence of pregnancies complicated by neural tube defects (NTD) among those who established care in the first trimester.

**Study Design**: Beginning in 9/2019, a prenatal supplement containing 1000 µg of folic acid was provided to patients during routine prenatal appointments. This retrospective cohort study examined singleton pregnancies with established prenatal care in the first trimester before (1/2016–8/2019) and after (9/2019–12/2023) program implementation. Patients with an isolated NTD on ultrasound were identified from the electronic medical record, pregnancies complicated by genetic syndromes were excluded. Data regarding demographics, pregnancy outcomes, and NTD characteristics were examined. Analysis was performed using student t‐test, chi square and Fisher's Exact Test. Adjustment for demographic factors was conducted using logistic regression.

**Results**: Before direct dispensation, 36 out of 25, 591 patients were diagnosed with NTD (1.4/1000) compared to 13 out of 26, 336 patients post‐dispensation (0.5/1000) (Figure). Anencephaly and spinal myelomeningocele were the most common NTD, rates 0.09% and 0.04% pre‐dispensation and 0.02% and 0.03% post‐dispensation respectively. Rate of NTD after direct dispensation was significantly lower (0.14% vs 0.05%, *p* value < .001) with an adjusted odds ratio of 0.35 (95% CI 0.17–0.66) indicating a 65% reduction in likelihood of NTD.

**Conclusion**: Direct dispensation of folate containing prenatal supplements in the first trimester was associated with reduction in the incidence of NTDs among patients delivering at a single safety net hospital. Direct dispensation of prenatal vitamins is a low‐cost intervention that was associated with reduced incidence of NTD in this population, prompting further investigation of direct dispensation of folic acid as a method of reducing NTD.

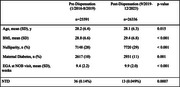

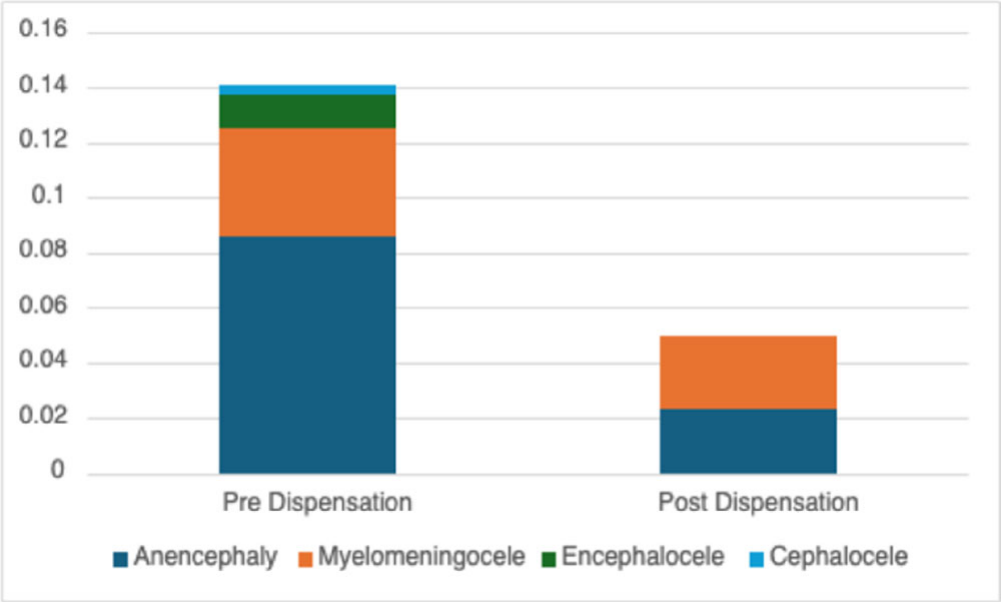

**10:30 AM – 12:00 PM**

## 851 | **Breaking the Cycle of Preterm Birth: Prospective Cohort Study on Maternal Stress and Recurrent PTB**

Maayan Hagbi Bal^1^; Eyal Sheiner^2^; Israel Yoles^3^; Iris Shoam^4^; Shimrit Yaniv Salem^5^; Noa Dina Israel‐Tov^4^; Tamar Wainstock^6^

^1^Icahn School of Medicine at Mount Sinai, Beer sheva, HaDarom; ^2^Soroka University Medical Center, Faculty of Health Sciences, Ben‐Gurion University of the Negev, beer sheva, HaDarom; ^3^Clalit Health Services, The Central District, Rishon Le Tzion, HaMerkaz; ^4^Ben‐Gurion University of the Negev, Ben Gurion, HaDarom; ^5^Faculty of Health Sciences, Joyce & Irving Goldman Medical School at Ben‐Gurion University, Beer sheva, HaDarom; ^6^Ben‐Gurion University of the Negev, Beer sheva, HaDarom

**Objective**: To investigate the association between prenatal maternal perceived stress, salivary cortisol, and the risk of recurrent preterm birth (rPTB).

**Study Design**: This prospective cohort study was conducted at a tertiary medical center (IRB#0390–20‐SOR). The study population included pregnant women with a history of spontaneous singleton preterm birth (PTB). Participants provided saliva samples and completed validated stress questionnaires twice during pregnancy. The incidence of rPTB was compared between women with high versus low cortisol levels and perceived stress, defined as greater than the 75^th^ percentile for the cortisol levels and the questionnaire score. Multivariable logistic regression models were used to assess the association between elevated stress markers and the risk of rPTB.

**Results**: Among 245 women with a history of PTB included in the study, 55 (22.4%) experienced rPTB. No differences were observed between women with rPTB versus women without in terms of demographic, health, or lifestyle characteristics. No association was observed between cortisol levels, high perceived stress in the first trimester, and rPTB in either of the study groups (Figure 1). High perceived stress levels later in second/third trimesters, were associated with a higher incidence of rPTB compared to the first trimester (38.9% vs. 19.2%; *p* = 0.011, adj. OR = 2.44, 95% CI: 1.053–5.639; *p* = 0.038), after adjusting for maternal age, number of prior PTBs, and parity. Women with consistently high stress levels had the highest incidence of rPTB (40%), followed by women with increased stress in the second and third trimesters only (37.5%). Multivariable regression analyses identified consistently high stress (adj. OR = 3.82, 95% CI: 1.37–10.64; *p* = 0.010) and high stress in the second/third trimester of pregnancy (adj. OR = 3.18, 95% CI: 1.04–9.73; *p* = 0.042) as significant risk factors of rPTB (Table 1).

**Conclusion**: High perceived stress levels during late pregnancy are significant risk factors for PTB. However, saliva cortisol was not statistically significant in relation to the risk of recurrence.

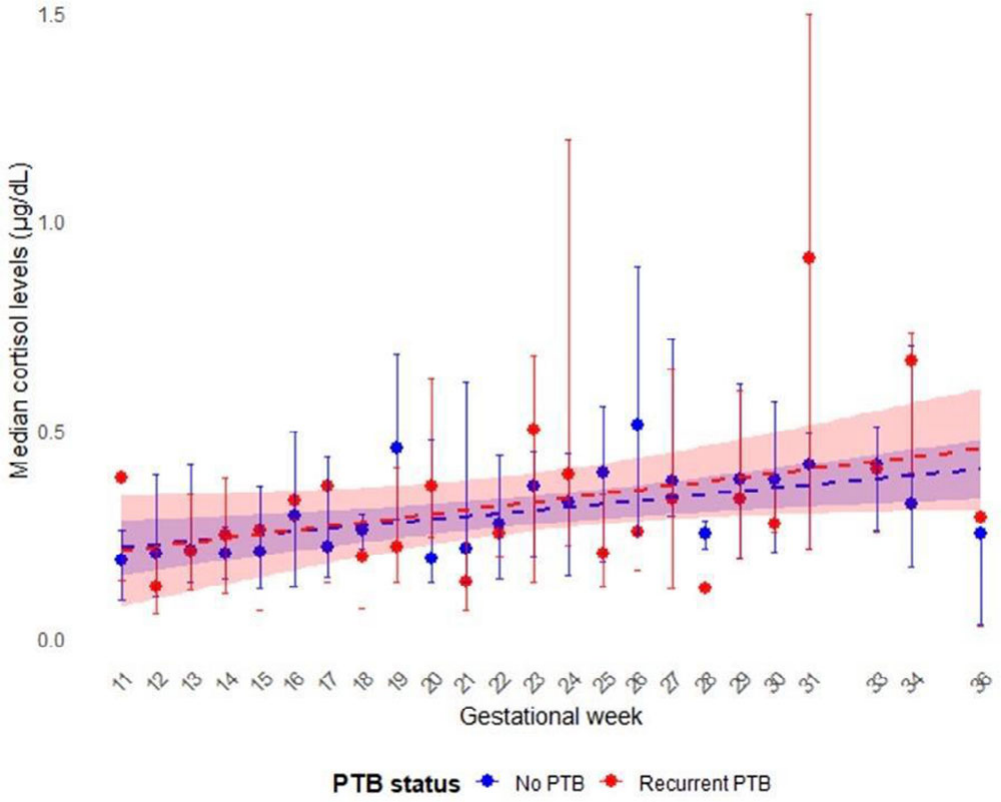

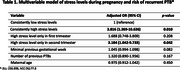

**10:30 AM – 12:00 PM**

## 852 | **What Can cfDNA Tell Us About Normal Pregnancy? Insights from cfChIP‐seq**

Maayan Ormianer^1^; Joshua Isaac Rosenbloom^1^; Esty Harpenas^2^; Nir Friedman^2^; Tal Levi^2^; Hadar Rosen^3^

^1^Hadassah Medical Center and Hebrew University of Jerusalem, Jerusalem, Israel, Jerusalem, Yerushalayim; ^2^Hebrew University of Jerusalem Israel, Jerusalem, Yerushalayim; ^3^Hadassah Medical Center and Hebrew University of Jerusalem, Jerusalem, Israel, Hadassah Medical Center Ein Kerem Campus, Jerusalem, Israel, Yerushalayim

**Objective**: Placental cell‐free DNA (cfDNA) circulating in maternal blood is widely used for non‐invasive prenatal testing. Recent evidence shows that most cfDNA exists as cell‐free chromatin, retaining epigenetic marks from the source cells. Analyzing these fragments via cfChIP‐seq allows identification of active genes and regulatory pathways in the tissue of origin. The release of placental cell‐free chromatin into maternal blood offers a non‐invasive window into placental molecular states. We hypothesized that physiologic and molecular changes during normal pregnancy are reflected in maternal cfChIP‐seq profiles, even early in gestation.

**Study Design**: This is a prospective longitudinal study. Sampling began as early as gestational week 9 and included all three trimesters, the peripartum period, and the immediate postpartum. For each participant, last menstrual period and clinical course were documented. cfChIP‐seq was used to profile promoter‐associated chromatin in plasma cfDNA, reflecting gene activity in contributing tissues.

**Results**: A total of 96 blood samples were collected from 36 pregnant women across gestation, with 2 to 7 samples per participant (mean 2.6). Genes were filtered for low signal in non‐pregnant controls and for correlation with gestational age (Kendall tau > 0.5). We identified 126 genes with low expression in non‐pregnant controls that showed increasing cfChIP signal across gestation. Ontology analysis (TISSUES Curated 2025) revealed enrichment in placenta, embryonic structures, and organism morphogenesis. Cell‐type enrichment (CellMarker 2024) confirmed placental cell predominance, supporting the placental origin of the signal and its relation to normal gestational physiology

**Conclusion**: cfChIP‐seq enables non‐invasive monitoring of transcriptional regulation during pregnancy and reveals dynamic gene expression patterns reflective of normal placental and embryonic physiology. These findings provide a foundational physiological reference for future studies exploring cfChIP in pregnancy complications such as IUGR, preeclampsia, gestational diabetes, and placenta accreta spectrum

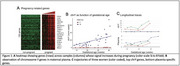

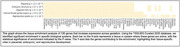

**10:30 AM – 12:00 PM**

## 853 | **Trends in Antepartum Anemia Prevalence Among Commercially Insured Individuals in the United States, 2018–2023**

Maciel E. Duverge‐Corporan^1^; Irogue I. Igbinosa^2^; Anna Booman^2^; Deirdre J. Lyell^3^; Brian T. Bateman^2^; Stephanie A. Leonard^4^

^1^Charles R. Drew University of Medicine and Science, College of Medicine, Inglewood, CA; ^2^Stanford University, Stanford, CA; ^3^Division of Maternal‐Fetal Medicine, Department of Obstetrics and Gynecology, Stanford School of Medicine, Stanford, CA; ^4^Stanford University, Stanford University, CA

**Objective**: Anemia during pregnancy increases the risk of adverse perinatal outcomes, yet recent data on the prevalence of anemia in pregnancy in the U.S. are lacking. We assessed the prevalence of anemia during pregnancy by analyzing hemoglobin and hematocrit laboratory values in a national cohort of commercially insured individuals from 2018 to 2023.

**Study Design**: We conducted a retrospective cohort study using the Merative^TM^ Marketscan^®^ Commercial Database of commercial insurance claims and laboratory data in the U.S. between 2018–2023. We included pregnant individuals without hereditary anemia, and with a live‐ or stillbirth at ≥28 weeks’ gestation. We defined anemia as hemoglobin < 11.0 g/dL or hematocrit < 33% in the first and third trimesters and hemoglobin < 10.5 g/dL or hematocrit < 32% in the second trimester, following ACOG and WHO guidelines. We calculated anemia prevalence overall and by age, obesity status, and region. We then assessed prevalence of anemia over time.

**Results**: Among 134, 163 individuals, 34, 314 (25.6%) of individuals had anemia during pregnancy based on hemoglobin and hematocrit measurements (Figure 1). The prevalence was higher in individuals with younger age, obesity, and those who lived in the South and Northeast. The prevalence of anemia remained consistent from 25.9% in 2018 to 25.7% in 2023 (Figure 2).

**Conclusion**: One‐quarter of individuals had anemia during pregnancy in this national study of commercially insured individuals and this prevalence remained steady over time with variation by patient characteristics. The prevalence of anemia was higher than previous estimates and is likely to be even higher in an economically disadvantaged population.

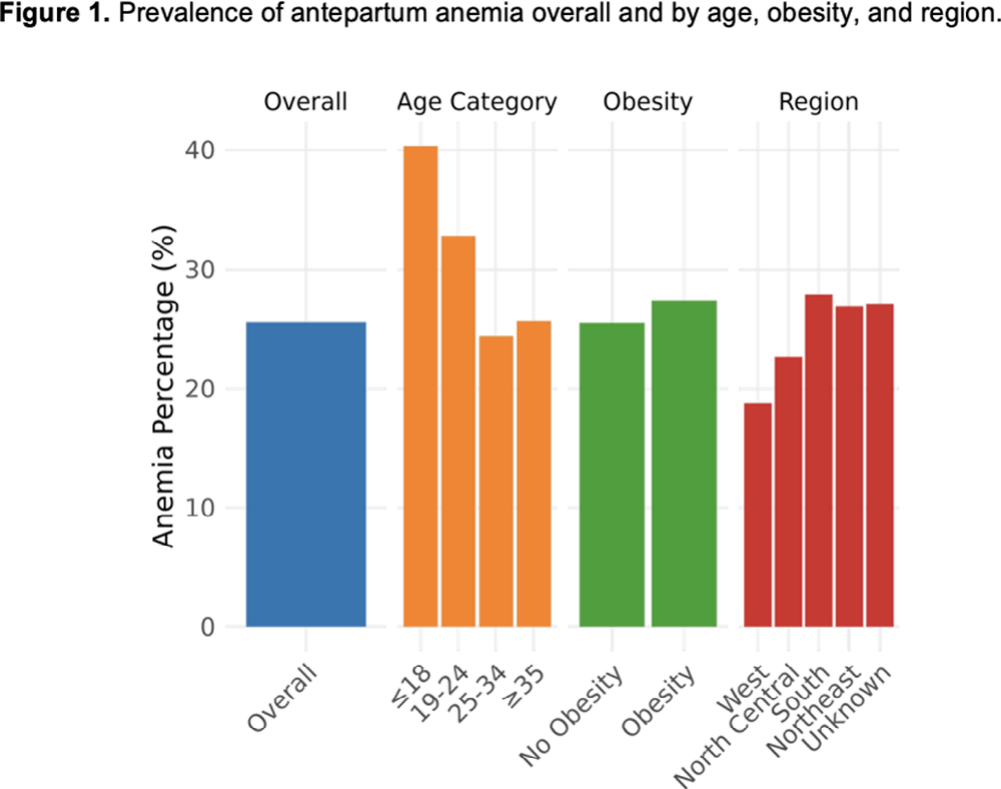

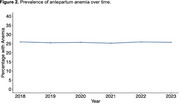

**10:30 AM – 12:00 PM**

## 854 | **Do** Patient **Characteristics Influence Cervical Length Screening?**

Alicia Mandujano^1^; Mackenzie Cronin^2^; Jim A. Shelton^1^; Michelle Lauria^1^

^1^University at Buffalo, Buffalo, NY; ^2^Christiana Care Health Services, Kennett Square, PA

**Objective**: To evaluate if unconscious bias influenced cervical length (CL) screening within a single health system.

**Study Design**: This was a retrospective cohort study of ultrasound (US) performed in high risk units (HRU) within a multi‐site health system. US were performed during 2023 in singleton gestations between 16w0d–24w0d.

**Results**: 5150 cases met inclusion criteria. For pregnant patients with multiple studies, only the first study was used. Cases with missing data or cases performed in a non‐HRU were excluded leaving 1294 cases. Earlier gestational age was less likely to have CL screening performed (19.4 weeks [range 16.7–22.1] vs 20.0 weeks [range 18.4–20.9], *p*‐value 0.02). Though there was a trend to variation in maternal age and Medicaid coverage, these differences were not significant. Women with “other” for race were less likely to undergo CL screening when compared to white women (OR 0.49; 95% CI 0.26–0.95; *p* 0.029). BMI, African American and Hispanic race, median income, and preferred language were not found to influence CL performance.

**Conclusion**: These data did not demonstrate significant impact of unconscious bias in CL screening within a HRU. Further investigation as to why patients of “other” race/ethnicity did not have CL screening performed is recommended to confirm consistent and equitable quality of care.

**10:30 AM – 12:00 PM**

## 855 | **Diagnosis of Chronic Hypertension During Prenatal Care Through Remote Blood Pressure Monitoring**

Madalyn Myers^1^; Serenity Budd^2^; Stephen Fernandez^2^; Diyang Lyu^2^; Loral Patchen^3^

^1^Medstar Washington Hospital Center/Georgetown University Obstetrics & Gynecology, Washington DC, DC; ^2^MedStar Health Research Institute, District of Columbia, DC; ^3^MedStar Washington Hospital Center, District of Columbia, DC

**Objective**: Chronic hypertension (cHTN) in pregnancy is associated with adverse maternal and neonatal outcomes and necessitates specific management. Early detection and treatment of cHTN provides the opportunity to protect cardiovascular health during and after pregnancy. A distributed care network implemented a remote blood pressure monitoring (RBPM) intervention into routine prenatal care starting at the first visit for all patients at one of its urban obstetrical sites. We compared new diagnoses of cHTN among RBPM users at the intervention site to patients receiving usual care.

**Study Design**: This is a retrospective cohort study using data from patients enrolled versus not enrolled in the RBPM intervention between 01/2020 and 09/2023. The primary outcome was new diagnosis of cHTN within the first 20 weeks of prenatal care. Age at delivery, race, ethnicity, insurance type, prior diabetes diagnosis, prior myocardial infarction, tobacco use during pregnancy, and BMI were covariates. A logistic regression model was fit to model the association between cHTN diagnosis and covariates. Average predicted probabilities of cHTN with 95% confidence intervals were displayed, and a significance level of 0.05 was used for all analyses.

**Results**: 7, 866 patients were included in this study, with *n* = 670 in the intervention group and *n* = 7, 196 in the comparison group. Patients in the intervention group were 2.24 times more likely to have a diagnosis of cHTN compared to those receiving usual care after adjusting for all covariates (*p* < 0.001). The predicted probability of a diagnosis of cHTN in the intervention group was 3.26% [2.29%, 4.63%] compared to 1.48% [1.20%, 1.82%] in the usual care group.

**Conclusion**: Patients enrolled in the prenatal RBPM intervention group had significantly more diagnoses of CHTN in the first 20 weeks of pregnancy after adjusting for demographic variables and comorbidities.

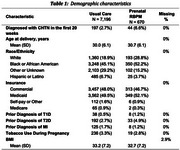

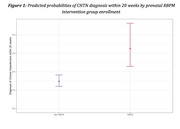

**10:30 AM – 12:00 PM**

## 856 | **Utility of Ultrasound Surveillance of Fetal Growth in Patients with Velamentous Cord Insertion**

Madeline J. Pence^1^; Anna Madden‐Rusnak^1^; Jermiah Crowder^2^; Madeline Petrikas^2^; Kobina Ghartey^3^; Alison G. G. Cahill^3^; Lorie M. Harper^1^

^1^Department of Women's Health, Dell Medical School at the University of Texas at Austin, Austin, TX; ^2^Dell Medical School at the University of Texas at Austin, Austin, TX; ^3^Department of Women's Health, Dell Medical School at the University of Texas at Austin, Department of Women's Health, Dell Medical School at the University of Texas at Austin, TX

**Objective**: Velamentous cord insertion (VCI) is associated with fetal growth restriction (FGR); ACOG recommends antenatal testing starting at 36 weeks (w) but does not make recommendations on growth surveillance. The purpose of this study was to evaluate the timing of ultrasound surveillance in patients with VCI.

**Study Design**: Retrospective cohort of singleton pregnancies diagnosed with VCI < 24w at a single center from 9/2021–5/2025. Pregnancies complicated by chronic hypertension, diabetes, BMI≥ 35 kg/m2, or age ≤ 16 or ≥ 40 years were excluded. Subsequent growth ultrasounds were excluded after patients developed gestational diabetes or pregnancy‐induced hypertension. Outcomes considered were diagnosis of FGR (estimated fetal weight (EFW) or abdominal circumference (AC) < 10%ile) or oligohydramnios (amniotic fluid index (AFI) ≤ 5cm). We calculated the number needed to screen (NNS) and 95% confidence interval (CI) to identify one abnormality for scans that were performed at a gestational age of 24–31w6d and ≥ 32w.

**Results**: Among 65 patients with isolated VCI meeting inclusion criteria, 151 additional ultrasounds were performed, and the majority received >1 ultrasound for growth surveillance (38% received two, 38% received ≥3, Figure 1). Two cases of FGR were diagnosed in total, both ≤ 32w (Table 1). Chart review demonstrated both cases of FGR were diagnosed at 30–31w and persisted through delivery. The NNS to diagnose one case from 24–31w6d was 26.5 (95% CI: 7.7–217.2). No cases of oligohydramnios were diagnosed. No new cases of FGR were diagnosed after 32w, suggesting that the risk of FGR is not higher than 5%, based on the calculation (1‐Maximum Risk)^n^ = 0.05.

**Conclusion**: Among patients with isolated VCI, few cases of FGR were diagnosed, and were diagnosed at 30–32w. Therefore, if an ultrasound surveillance screening protocol for patients with VCI is used, a single ultrasound at 30–32w to evaluate growth is likely sufficient, followed by antenatal testing beginning at 36w per ACOG recommendations.

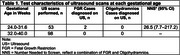

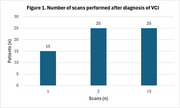

**10:30 AM – 12:00 PM**

## 857 | **Use of** Medicaid **Covered Doula Services and Associated Pregnancy Outcomes**

Madeline F. Perry^1^; Diana Montoya‐Williams^2^; Sindhu K. Srinivas^3^; David Grande^4^; Scott A. Lorch^2^

^1^Hospital of the University of Pennsylvania, Philadelphia, PA; ^2^Children's Hospital of Philadelphia, Philadelphia, PA; ^3^Department of Obstetrics and Gynecology, Perelman School of Medicine, Maternal and Child Health Research Center, Philadelphia, PA; ^4^Perelman School of Medicine, Philadelphia, PA

**Objective**: Doulas are non‐medical professionals who provide support during pregnancy and postpartum and are a potential resource to mitigate perinatal disparities. State Medicaid programs have increasingly provided coverage for doula services with limited data on their effects on outcomes. We aim to describe the sociodemographic characteristics of birthing people who use doula services covered by Medicaid and pregnancy outcomes.

**Study Design**: This cross‐sectional analysis utilized T‐MSIS Analytic File (TAF) data from 2016–2019 in Minnesota and Oregon (the earliest adopters of Medicaid covered doula services). Exposure was use of doula services during pregnancy through 6 weeks postpartum, where patients using doula services were matched 1:3 to patients not using doula service for sociodemographic and medical factors associated with doula use. Outcomes included cesarean delivery (CD), preterm birth (PTB) < 37 weeks, postpartum hemorrhage, postpartum anxiety or depression, severe maternal morbidity, and any ED visit or inpatient admission in the first 60 days postpartum. Conditional logistic regression determined the association of doula use on outcomes controlling for sociodemographic and medical factors.

**Results**: Of 121, 268 births, 0.6% (*n* = 799) used doula services covered by Medicaid. Doula use was more common among Black patients and people living in zip codes with a higher median income (Table 1). In the matched cohort, PTB was 49% less likely (aOR 0.51, 95% CI 0.35, 0.74). Doula use was associated with lower odds of cesarean delivery (aOR 0.84, 95% CI 0.70–1.00). Other clinical outcomes were not associated with doula services (Table 2).

**Conclusion**: Use of Medicaid‐covered doula services was associated with a 49% reduction in PTB even after accounting for SES and health differences in patients using doula services. However, these services are only used by < 1% of the eligible population and more common among Medicaid recipients living in higher median income zip codes. Expansion of such services may improve outcomes and address disparities in maternal child health.

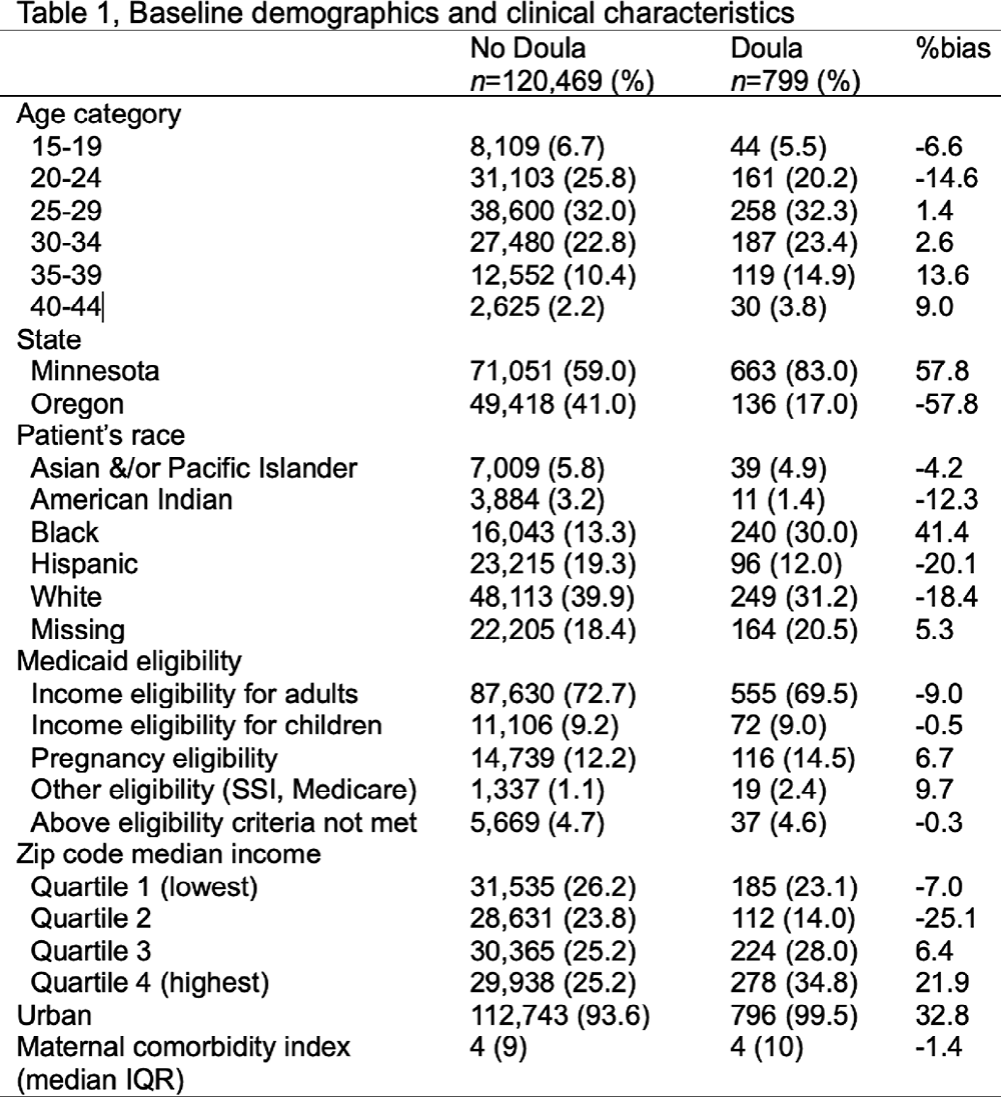

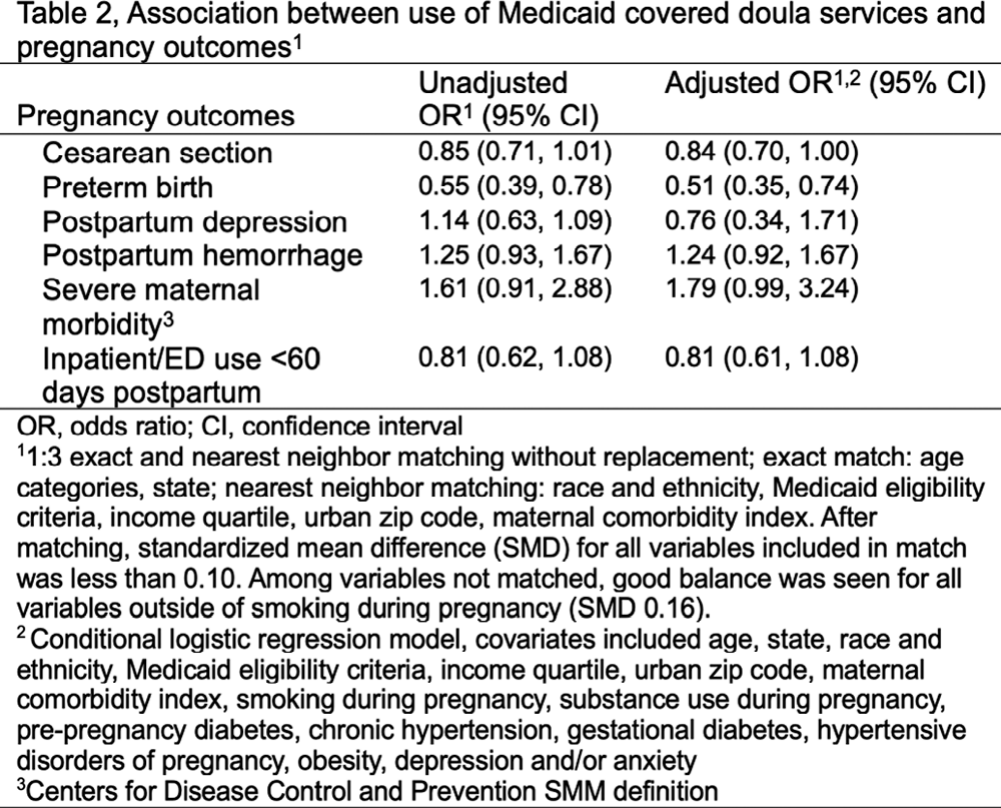

**10:30 AM – 12:00 PM**

## 858 | Postpartum **Psychiatric Morbidity Following Pregnancy‐Associated Intimate Partner Violence and Sexual Assault**

Manasa G. Rao^1^; Timothy Wen^2^; Emily Hansman^3^; Mary E. D'Alton^4^; Alexander M. Friedman^4^

^1^Columbia University, New York, NY; ^2^UCSD, Irvine, CA; ^3^Columbia University Medical Center, New York, NY; ^4^Division of Maternal‐Fetal Medicine, Department of Obstetrics and Gynecology, Columbia University Irving Medical Center, New York, NY

**Objective**: Intimate partner violence (IPV) and sexual assault (SA) during pregnancy are associated with higher rates of depression, anxiety, substance use disorders (SUD) and maternal mortality, yet national data on short term risks of psychiatric and SUD morbidity remains limited. This study aims to evaluate the association of IPV/SA in pregnancy and postpartum readmissions with de novo psychiatric or SUD at the national level.

**Study Design**: The 2016–2021 Nationwide Readmissions Database was used to identify delivery hospitalizations with an IPV/SA diagnosis, excluding preexisting psychiatric or SUD diagnoses. Outcomes were 180‐day readmissions with a new (1)psychiatric or (2)SUD diagnosis, identified using ICD–10 codes. Joinpoint regression assessed temporal trends in IPV/SA and de novo psychiatric and SUD diagnoses, expressed as average annual percent change (AAPC). Multivariable logistic regression models assessed the association between IPV/SA in pregnancy and postpartum readmissions for de novo psychiatric and SUD diagnoses, adjusting for demographic, clinical, and delivery complications.

**Results**: Among 10.4 million delivery hospitalizations, 22, 515 (22 per 10, 000 births) were complicated by IPV/SA, increasing from 14 to 31 per 10, 000 births during the study period (AAPC 16.8%, 95% CI:14.6%–19.6%) with no significant changes in de novo psychiatric or substance use readmissions. Postpartum readmissions with de novo psychiatric (1.1% vs. 0.3%) and substance use (1.0% vs. 0.2%) disorders were higher among those with IPV/SA compared to those without (Table 1). Adjusted analysis showed nearly three times higher likelihood of readmissions with de novo psychiatric (aOR 2.53, 95% CI: 1.94, 3.31) and SUD (aOR 2.61, 95% CI: 2.02, 3.39) among those with IPV/SA (Table 2).

**Conclusion**: Despite low overall readmission, rates of IPV/SA in pregnancy have increased over time. IPV/SA during pregnancy is significantly associated with increased rates of de novo psychiatric and substance use disorders. This data may underscore the need tailored perinatal screening to mitigate postpartum psychiatric morbidity.

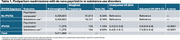

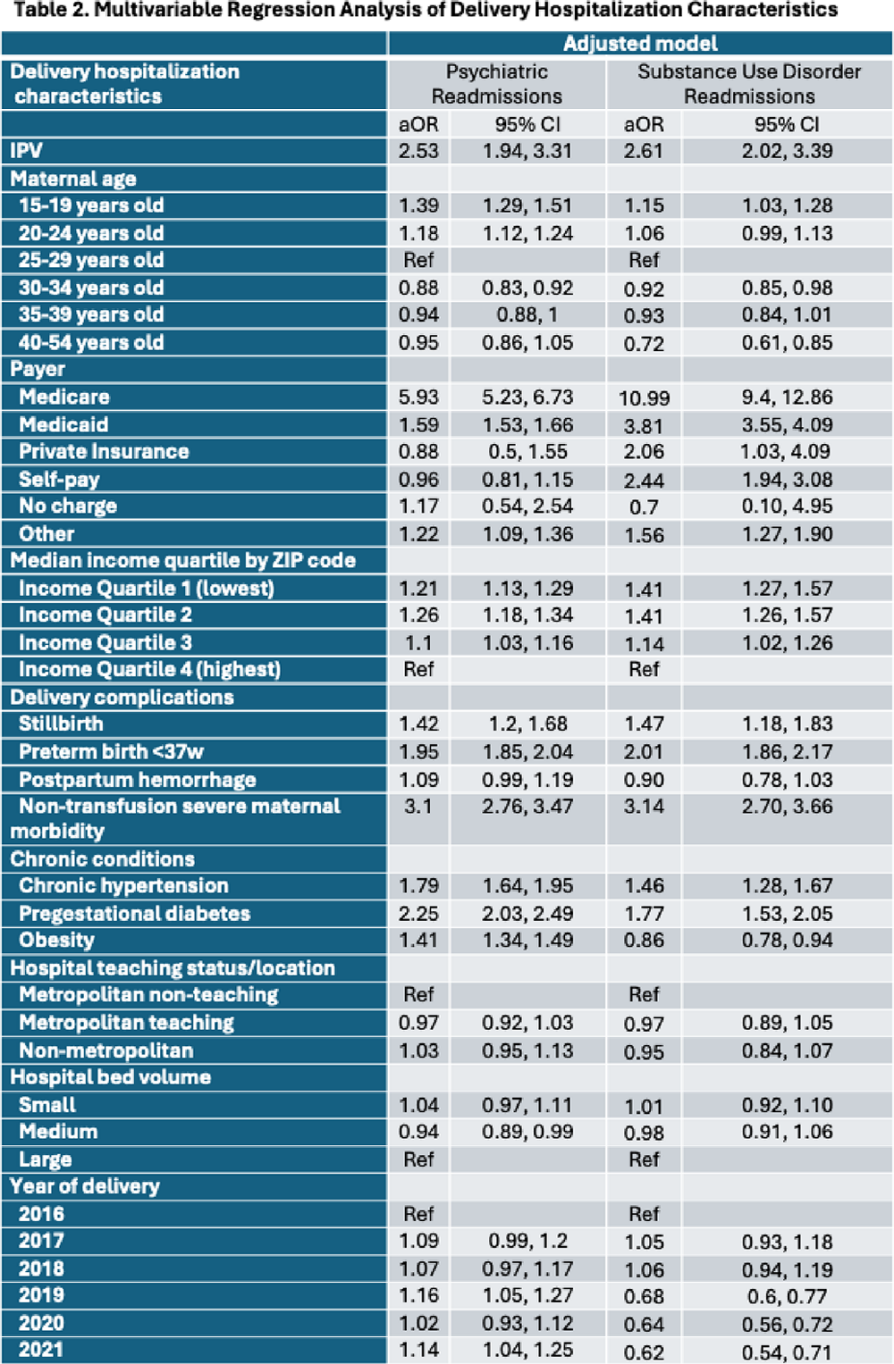

**10:30 AM – 12:00 PM**

## 859 | **Factors** that **Account for Cesarean Delivery in the Chronic Hypertension Population**

Marcia Des Jardin; Justin M. Leach

On behalf of the CHAP Consortium

Center for Research in Women's Health, University of Alabama at Birmingham, Birmingham, AL

**Objective**: The cesarean delivery rate in patients with chronic hypertension (CHTN) is high (up to 50%). We aim to identify risk factors for cesarean delivery in patients with CHTN to explain the increased rate.

**Study Design**: This is a secondary analysis of CHAP, a multicenter treatment RCT of patients with mild CHTN enrolled prior to 23 weeks’ gestation. Logistic regression was performed for both unadjusted and multivariable models of cesarean section using predefined characteristics (Table). The multivariable models were (1) full model, (2) full model excluding labor type. The area under the ROC curve was used to evaluate multivariable models for predictive performance.

**Results**: Of 2292 patients who were included, 1174 had a cesarean delivery and 1118 had a successful vaginal delivery, for a cesarean rate of 49%. In the full model, increasing BMI, pregestational diabetes, preeclampsia, nulliparity, no prior preeclampsia, and “no labor” were associated with increased CD. When excluding labor type, nulliparity and no prior preeclampsia were similarly associated, but preeclampsia and prior preeclampsia were not statistically significant with cesarean delivery; additionally increasing age, and decreasing gestational age were associated with cesarean delivery.

Predictive performance as estimated by AUC was 0.87 (95% CI: [0.86, 0.89]) for the full model, 0.68 (95% CI: [0.66, 0.70]) excluding labor type (Figure 1).

**Conclusion**: Risk factors for CD in a CHTN cohort are similar to reported risk factors for all US patients. In a model that includes all labor types (none, spontaneous or induced labor), a prediction model for cesarean deliveries was superior to one that excluded labor or evaluated baseline characteristics only.

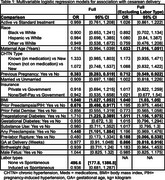

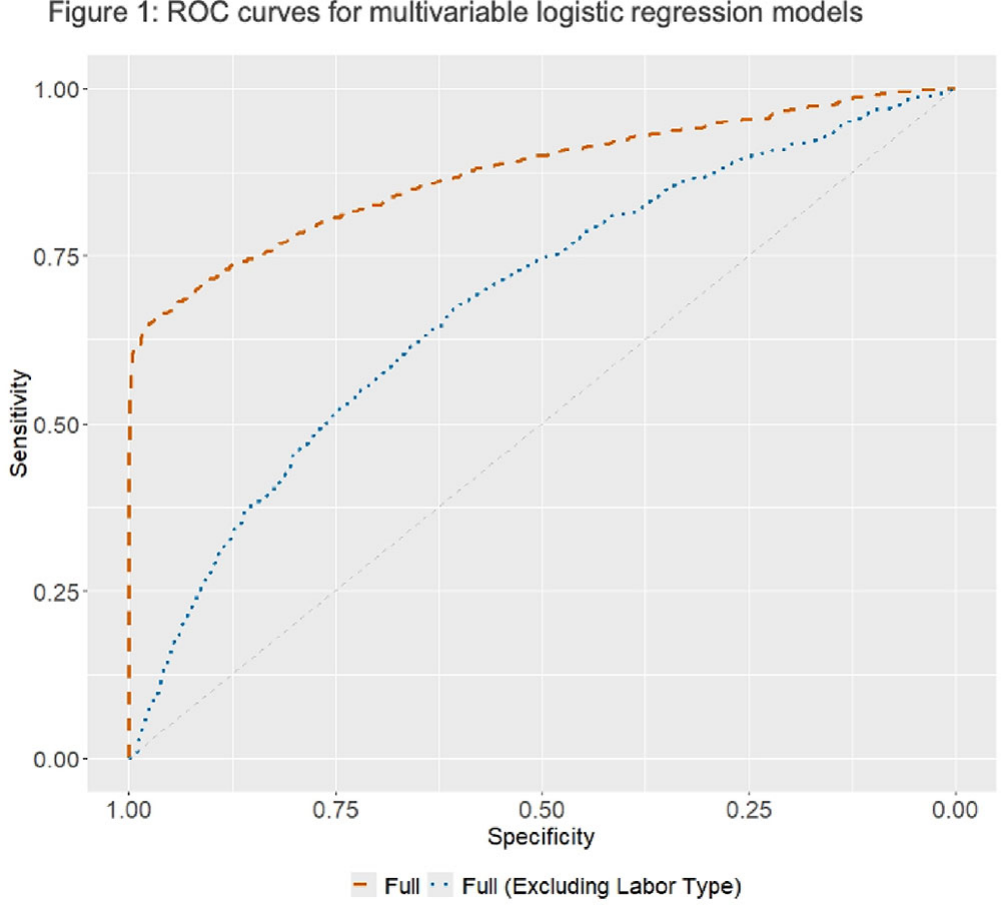

**10:30 AM – 12:00 PM**

## 860 | **Maternal Postpartum Blood Pressure Prior to Delivery Discharge and Risk of Hospital Readmission**

Margaret R. Page; Justin M. Leach; Hui‐Chien Kuo; Samantha L. Martin; Alan TN Tita

On behalf of the CHAP Consortium

Center for Research in Women's Health, University of Alabama at Birmingham, Birmingham, AL

**Objective**: Postpartum hypertension (HTN) is a risk factor for hospital readmission and is associated with increased risk of maternal morbidity and death. We examined the association between adequate BP control (mean < 140/90 mmHg) based on the 3 measures before delivery discharge and hospital readmission in patients with chronic hypertension (CHTN).

**Study Design**: Secondary analysis of all patients enrolled in the Chronic Hypertension and Pregnancy trial. Patients who had a miscarriage, were lost to follow‐up, or were missing BP measurements during delivery hospitalization were excluded. The primary outcome was postpartum readmission. Characteristics and outcomes were compared between those who had a mean BP < 140/90 prior to discharge versus those with mean BP ≥140/90. Logistic regression was used to control for significant covariates.

**Results**: Of 2, 223 eligible patients, 1, 554 (70%) had mean pre‐discharge BPs < 140/90 and 669 (30%) had mean BP ≥ 140/90. Patients who had mean BP ≥140/90 were more likely to be Black, have had a cesarean and delivered earlier, use antihypertensive medications at discharge, and experience longer delivery hospitalization (Table 1). Patients with controlled BPs had 104 total readmissions (6.7%) with average length of stay (LOS) 3.7 days, whereas those with uncontrolled BPs had 52 readmissions (7.8%) with average LOS 3.3 days, though the difference in readmission rate was not statistically significant (Table 2). Further, there were no differences in rates of severe maternal morbidity or readmission length between the two groups.

**Conclusion**: Higher postpartum BPs just prior to discharge were not associated with significantly increased rates of hospital readmission in patients with mild CHTN, even though these patients did have worse delivery outcomes. Higher use of antihypertensives in the those with higher BPs likely mitigated the risk of hospitalizations.

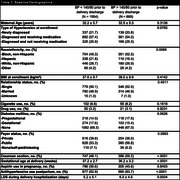

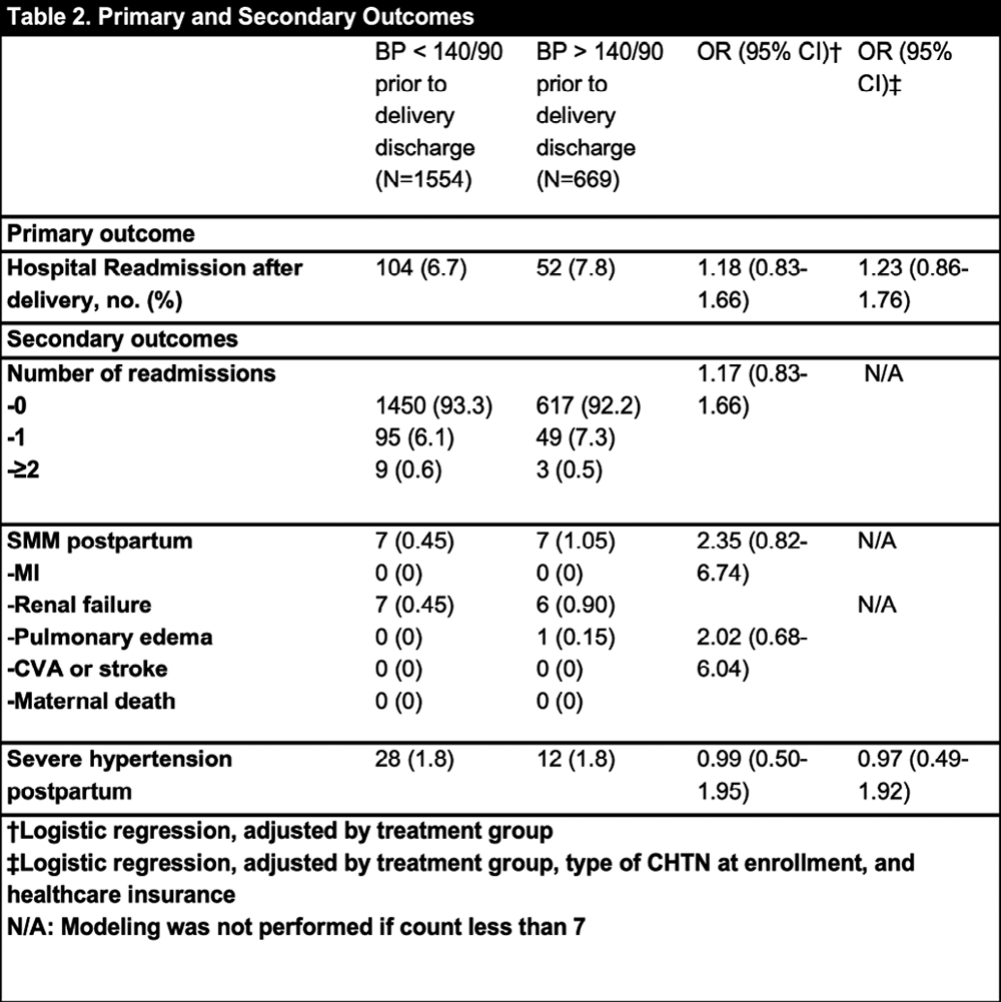

**10:30 AM – 12:00 PM**

## 861 | **Opioid‐**Sparing **Protocol and Post‐Cesarean Opioid Prescription among Individuals With Substance Use Disorders**

Marina Kern^1^; Aidyn Eldredge^1^; Casey Tak^2^; Theresa Kurtz^3^; Christine M. Warrick^2^; Mark D. Rollins^1^; Marcela C. Smid^1^

^1^University of Utah Health, Salt Lake City, UT; ^2^University of Utah, Salt Lake City, UT; ^3^Intermountain Healthcare, Salt Lake City, UT

**Objective**: In response to rising deliveries complicated by substance use disorder (SUD), particularly opioid and stimulant use disorders, our institution implemented an opioid‐sparing (OS) postpartum pain protocol in 2018, incorporating epidural and/or low‐dose ketamine infusions with multimodal analgesia (scheduled acetaminophen, ibuprofen or ketorolac, and pregabalin). We aimed to evaluate the association between the OS protocol and opioid prescriptions at discharge following cesarean birth in individuals with SUD.

**Study Design**: We conducted a retrospective cohort study of individuals who underwent cesarean birth at a single academic hospital from Jan 2018 to Dec 2023. Three groups were compared: (1) individuals with SUD who received the OS protocol, (2) individuals with SUD who received standard pain management (scheduled acetaminophen, ibuprofen, and as‐needed oxycodone), and (3) a control group without SUD who received standard management (one individual randomly selected per month of study period). The primary outcome was opioid prescription at discharge. Logistic regression estimated the association, adjusted for confounders.

**Results**: We included 273 individuals: 189 with SUD who received OS, 33 with SUD and standard management, and 51 controls. Demographic and clinical characteristics differed between groups (Table 1). Among OS recipients, 63.5% received both ketamine and epidural, 18.5% ketamine only, and 18.0% epidural only. Compared to controls, the OS group had over 90% lower odds of opioid prescription at discharge (88.2% vs. 18.5%; aOR 0.08, 95% CI 0.02–0.30) (Figure 1). There was no significant difference between the control group and the SUD group with standard management (88.2% vs. 66.7%; aOR 0.42, 95% CI 0.11–3.43).

**Conclusion**: At a single institution, the OS pain management protocol significantly reduced post‐cesarean opioid prescriptions among individuals with SUD.

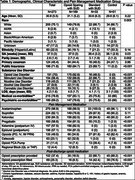

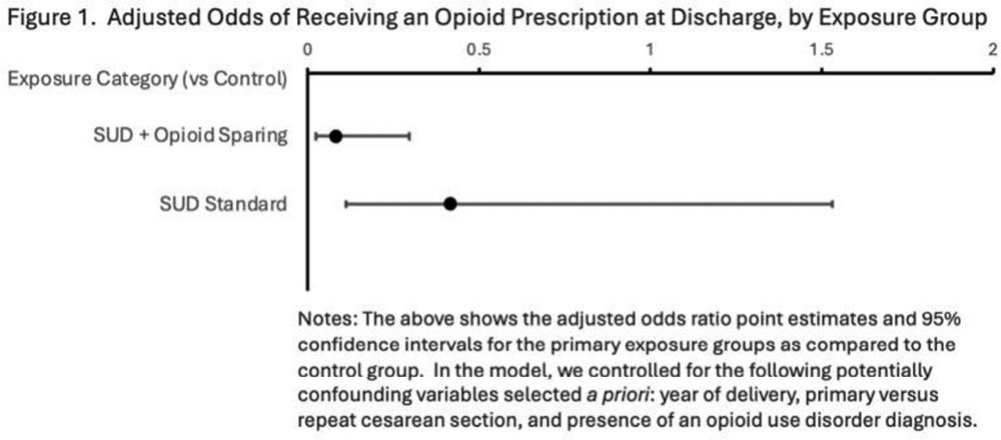

**10:30 AM – 12:00 PM**

## 862 | Improving **Placenta Accreta Spectrum Prediction: Integrating Conventional Statistics and Machine Learning**

Mario I. Lumbreras‐Marquez; Maria Diaz‐Diaz; Yubia Amaya‐Guel; Maria Rodríguez‐Sibaja; Yazmin Copado‐Mendoza; Dulce Camarena‐Cabrera; Berenice Velazquez‐Torres; Manuel Gallardo‐Gaona; Diana Diaz‐Perez; Mario Rodriguez‐Bosch; Sandra Acevedo‐Gallegos; Jose Ramirez‐Calvo

Instituto Nacional de Perinatologia, Instituto Nacional De Perinatologia/Mexico City, Distrito Federal

**Objective**: To evaluate the predictive performance of logistic regression and a machine learning (ML) algorithm in identifying placenta accreta spectrum (PAS) using ultrasonographic signs assessed before delivery. Early and accurate PAS prediction is critical for surgical planning and improving maternal outcomes. However, access to expert interpretation of PAS‐related ultrasound findings is often limited, particularly in low‐resource settings.

**Study Design**: Retrospective study conducted at a quaternary referral center. We included patients with suspected PAS who underwent prenatal ultrasound assessment and had definitive intraoperative or histopathological confirmation. Ultrasonographic signs and clinical variables were extracted and labeled (yes/no) using the ISUOG pro forma by maternal‐fetal medicine specialists. Grayscale ultrasound parameters included loss of the retroplacental hypoechoic zone, myometrial thinning, placental lacunae, bladder wall interruption, placental bulge, and focal exophytic mass. Color Doppler parameters included bridging vessels, uterovesical hypervascularity, subplacental hypervascularity, and turbulent lacunar flow. A multivariable logistic regression and a random forest model were developed. Model performance was evaluated for discrimination (using the area under the curve [AUC]) and calibration.

**Results**: We included 233 patients, of whom 157 (67.4%) had confirmed PAS. The logistic regression model achieved an AUC of 0.89, while the ML model achieved an AUC of 0.88, indicating similar discrimination. Calibration showed good agreement between predicted and observed risks for both models (Hosmer‐Lemeshow *p* = 0.523; Brier Score = 0.121 for random forest). Key predictors included loss of the “clear zone”, myometrial thinning, and bridging vessels.

**Conclusion**: Both models demonstrated strong performance in predicting PAS. ML offered no improvement in discrimination. With further validation, such models could help standardize PAS risk assessment and guide referral or surgical planning, especially in settings with limited access to high‐risk obstetric ultrasound expertise.

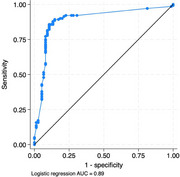

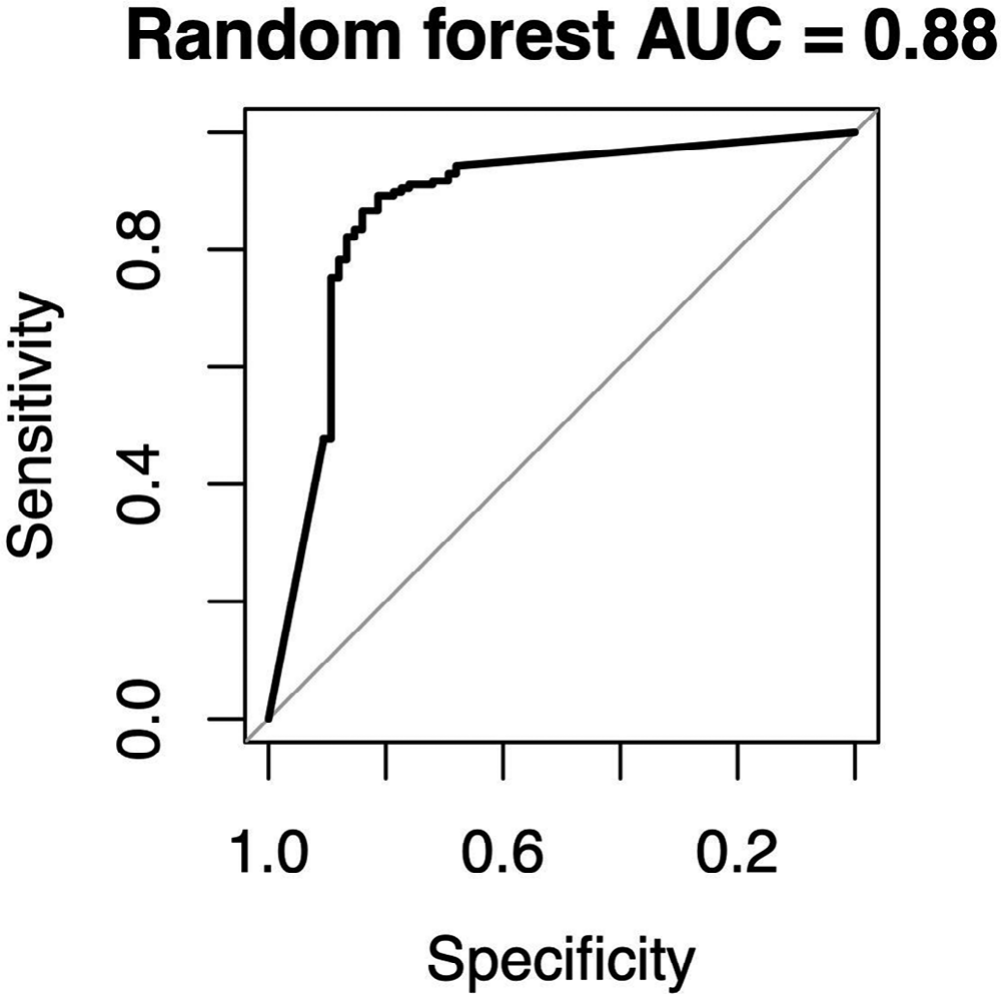

**10:30 AM – 12:00 PM**

## 863 | **Timing of Mid‐Trimester Loss in Index Pregnancy Predicts Recurrent Adverse Outcomes After Previable PPROM**

Marissa A. Hand^1^; Hannah Sinks^2^; Amrit Dhanushkoti^3^; Christine Chien^4^; Caroline Pennacchio^5^; Catherine Klammer^6^; Adriana Huelmo^3^; Meng Yao^7^; Carol Wang^3^; Lisa Gray^8^; Dzhamala Gilmandyar^2^; Ahmed Ahmed^7^; Amol Malshe^7^; Craig Pennell^3^

^1^Cleveland Clinic Foundation, Cleveland Clinic Foundation, Cleveland, OH; ^2^Hofstra Northwell School of Medicine, Hofstra Northwell School of Medicine, NY; ^3^University of Newcastle, University of Newcastle, New South Wales; ^4^Carle Foundation Hospital, Urbana, IL; ^5^University of Pittsburgh Medical Center, University of Pittsburgh Medical Center, PA; ^6^University of Pennsylvania, University of Pennsylvania, PA; ^7^Cleveland Clinic Foundation, Cleveland Clinic Foundation, OH; ^8^Carle Foundation Hospital, Carle Foundation Hospital, IL

**Objective**: Previable rupture of membranes is associated with high rates of fetal loss, neonatal morbidity, and maternal complications. The timing of rupture may influence both immediate and future outcomes. Early mid‐trimester loss (14–20 weeks) may represent a distinct clinical phenotype compared to later losses (20–24 weeks), potentially altering recurrence risk. However, data on how gestational age at rupture impacts subsequent pregnancy outcomes remain limited.

**Study Design**: This multinational retrospective cohort study included women with previable PPROM (14w0d–23w6d) from U.S. and Australian sites (2010–2023) who had a subsequent pregnancy reaching ≥14 weeks. Exclusions included intrauterine fetal demise prior to rupture or iatrogenic rupture. Subsequent pregnancies were categorized as term (≥37w), preterm birth (24w0d–36w6d), or mid‐trimester loss (14w0d–23w6d). Group comparisons were performed using Chi‐square and ANOVA tests; *p* < 0.05 was considered significant.

**Results**: Among 140 patients with prior previable PPROM, 48 (34.3%) had MTL or PTB in a subsequent pregnancy, while 92 (65.7%) delivered at term. Early mid‐trimester loss in the index pregnancy was strongly associated with mid‐trimester loss in the subsequent pregnancy (71.4% vs. 28.6%, *p* < 0.001). Progesterone use in the index pregnancy was more common among those with adverse outcomes (52.2% vs. 20.7%, *p* < 0.001), whereas aspirin or progesterone use in the subsequent pregnancy did not differ. Cerclage was more frequent in those with recurrent MTL or PTB (64.6% vs. 43.5%, *p* = 0.028). Rates of hypertensive disorders and recurrence did not differ by country.

**Conclusion**: One‐third of patients with previable PPROM had recurrent MTL or PTB, highlighting substantial risk in future pregnancies. Early mid‐trimester loss appears to mark a high‐risk phenotype for recurrent adverse outcomes, warranting tailored counseling and intensified surveillance in future pregnancies.

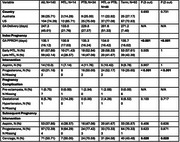

**10:30 AM – 12:00 PM**

## 864 | **Trauma in Pregnancy: Perinatal Outcomes at a Level I Trauma Center**

Marissa Platner^1^; Jessica Preslar^1^; Natalie Poliektov^1^; Sheree Boulet^2^

^1^Emory University School of Medicine, Atlanta, GA; ^2^Boston Medical Center, Boston, MA

**Objective**: Traumatic injury during pregnancy is a leading cause of non‐obstetric maternal and fetal morbidity and mortality. However, the association between the most severe traumatic injuries and adverse pregnancy outcomes (APOs) remains understudied. The aim of this study was to investigate the effects of severe traumatic injury during pregnancy on maternal and fetal outcomes in a high‐risk patient population.

**Study Design**: This retrospective cohort study examined all pregnancies with admission encounters at an urban safety net hospital between 2016 – 2022. Traumatic injury during pregnancy was defined as a pregnancy with an ICD–10 diagnosis and procedure code denoting trauma during pregnancy as well as an associated imaging encounter, in order to more accurately identify the most severe traumas. APOs were identified using ICD–10 diagnosis and procedure codes. Multivariable logistic regression was performed adjusting for maternal age, body mass index, parity, and race/ethnicity.

**Results**: Of *N* = 11, 181 pregnancies included in this analysis, *n* = 130 (1.2%) experienced a severe traumatic injury. 9% of the pregnancies with traumatic injury required hospitalization at the time of the event. Patients who experienced trauma had an increased odds of cesarean delivery (aOR 1.92 (1.18, 3.15)), preterm labor (aOR 2.26 (1.25, 4.09)), preterm birth (aOR 2.52 (1.48, 4.29)), placental abruption (aOR 4.47 (1.57, 12.74)), and Neonatal Intensive Care Unit (NICU) stay >3 days (aOR 1.86 (1.09, 3.19)) as compared to patients who did not experience severe trauma. (Table)

**Conclusion**: Traumatic injury during pregnancy severe enough to warrant diagnostic imaging was associated with an increased odds of APOs. These data support the need for early identification and multidisciplinary management of trauma in pregnant individuals.

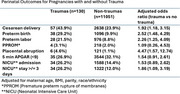

**10:30 AM – 12:00 PM**

## 865 | **Changes** in **Congenital Malformations in the Post‐Dobbs US: A State‐Level Public Health Assessment**

Marlee Hirsch^1^; Erez Lenchner^2^; Ashley Zimmermann^3^; Moti Gulersen^4^; Itai Hazan^5^; Eran Bornstein^3^

^1^Northwell, New York, NY; ^2^New York University Rory Meyers College of Nursing, New York, NY; ^3^Northwell, New Hyde Park, NY; ^4^Division of Maternal Fetal Medicine, Department of Obstetrics, Gynecology and Reproductive Science, Jefferson Health, Philadelphia, PA, Philadelphia, PA; ^5^Ben‐Gurion University of the Negev, Beer Sheva, HaDarom

**Objective**: To examine whether state‐level rates of live births with congenital malformations changed following the Dobbs supreme court decision.

**Study Design**: Retrospective analysis of the Centers for Disease Control and Prevention's Natality live birth database, with detailed geographical data available (2018–2023). We compared rates of live births with major congenital and/or chromosomal malformations in each state before and after the Dobbs ruling on June 24, 2022. The period from July‐December 2022 was considered transitional and excluded from the analysis. Pearson Chi Square testing was used with statistical significance set at *p* < 0.05.

**Results**: The prevalence of live births with congenital malformations varies significantly among states in both the pre and post Dobbs decision periods (pre‐Dobbs 0.11%–1.29%, and post‐Dobbs 0.13%–1.40%) (Figure 1). In addition, significant variations were noted in the change in prevalence of congenital anomalies between these two time periods (odds ratios (OR) ranging from 0.49–2.42). While a substantial decrease in congenital anomalies was noted in Georgia, Kentucky, Mississippi, Oregon, Rhode Island, Tennessee, Texas, and Wisconsin (OR range 0.49–0.79), a notable increase was noted in California, Delaware, Florida, Hawaii, Maine, Michigan, and Washington (OR range 1.21–2.42) (Figure 2). *p*‐value of 0.01 was calculated.

**Conclusion**: Significant state level variations in the prevalence of live births with congenital anomalies were observed during both the pre and post Dobbs periods. Moreover, the magnitude and direction of rate changes post‐Dobbs varied markedly by state, with some experiencing notable increases and others substantial decreases. Further state‐level investigation is warranted to elucidate the root causes of these divergent trends. Nevertheless, underlying factors such as disparities in access to prenatal care, availability of legal pregnancy termination, cultural and demographic differences, nutritional status, and environmental exposures may be contributing to this disparity.

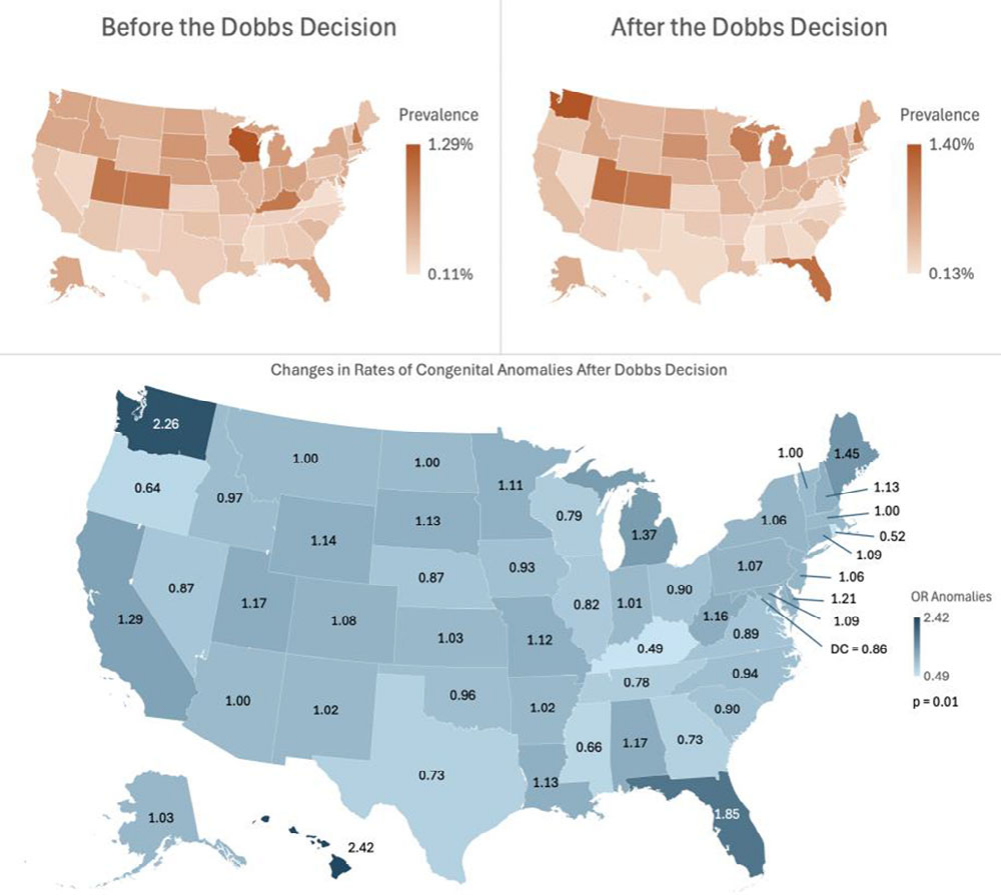

**10:30 AM – 12:00 PM**

## 866 | Mitigating **Pregnancy‐Associated Atherosclerotic Cardiovascular Risk: The Relationship Between Weight, Adverse Pregnancy Outcomes, and Cardiometabolic Conditions**

Mary Falstin; Gabriella Braun; Bryan L. Aaron; Levi J. Anderson; Adam Baruch; Samantha Schon; Lindsay Admon; Ashley M. Hesson

University of Michigan, Ann Arbor, MI

**Objective**: Atherosclerotic cardiovascular disease (ASCVD) is a leading cause of death for women known to correlate with antecedent adverse pregnancy outcomes (APOs). Elevated BMI is also a risk factor for ASCVD, but the relationship between pregnancy‐specific weight gain, APOs, and ASCVD risk is incompletely characterized.

**Study Design**: We modeled BMI trajectories for first singleton births occurring from 2014–2015 at an academic medical center. Linear models were fit to the BMI course of each participant within the index pregnancy, subsequent singleton pregnancies, and last documented weight. “High ASCVD risk” individuals were identified using variables from the American College of Cardiology (ACC) ASCVD risk calculator (diabetes, hypertension, or hyperlipidemia developed before the index pregnancy and/or non‐White race); those without these risk factors were coded as “Low ASCVD Risk”. Within each risk stratum, development of major cardiometabolic morbidity (MCM) after the index pregnancy was modeled with age, APO history, and geolocated poverty index.

**Results**: A total of 467 subjects (872 pregnancies) were analyzed. Mean age at first pregnancy was 29.6 ± 5.3. See Table 1 AVSC and APO status. The distribution of intra‐pregnancy BMI slopes (Figure 1) shows the development of de novo risk‐augmenting MCMs is associated with higher weight slope (more intra/ inter‐pregnancy weight gain) in those without APOs and low preexisting ASCVD risk. APOs significantly predicted the future development of MCMs in the low baseline risk model (*p* < 0.01), interacting significantly with BMI slope (*p* = 0.01). There were no significant predictors of new MCMs in the high‐risk model. Poverty index was not significant in the high or low risk group (*p* = 0.19 vs *p* = 0.42).

**Conclusion**: Without an APO, low ASCVD risk women are more likely to develop MCMs that contribute to lifetime ASCVD if they gain and retain pregnancy weight. Interventions targeting postpartum ASCVD risk reduction should include both those with APOs and excessive weight gain in/ between pregnancies to decrease the lifelong burden of women's cardiovascular morbidity and mortality.

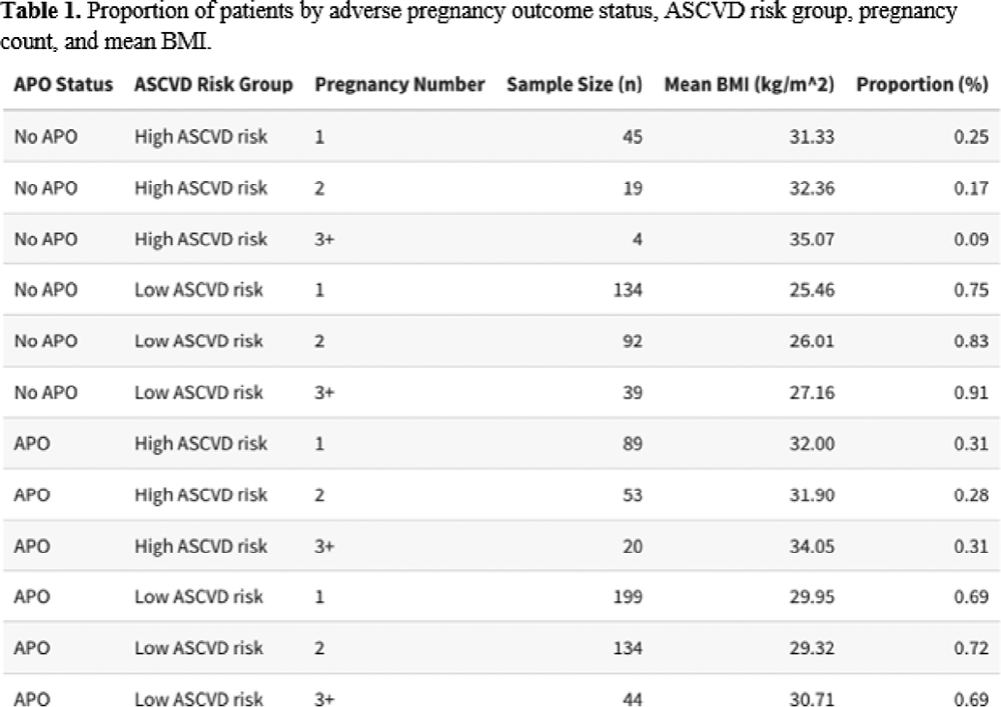

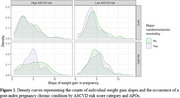

**10:30 AM – 12:00 PM**

## 867 | Comparative **Analysis of Short‐Interval Birth Rates Among Adolescents in Tennessee: Impact of IPP LARC Utilization**

Mary G. Herndon^1^; Kaylee Ramage^2^; Megan L. Young^3^; Alicia Mastronardi^1^; Sam Havron^1^; Jill M. Maples^4^; Jordan E. Lewis^1^; Katherine B. Kaak^3^; Shelley M. Stiltner^1^; Nikki B. Zite^1^

^1^University of Tennessee Graduate School of Medicine, Knoxville, TN; ^2^University of Tennessee, Knoxville, Knoxville, TN; ^3^University of Tennessee Medical Center, Knoxville, TN; ^4^University of Tennessee Health Science Center COM Knoxville, Knoxville, TN

**Objective**: Adolescent pregnancy impacts social determinants of health and is associated with adverse pregnancy outcomes. We aimed to describe the association of immediate postpartum long‐acting reversible contraception (IPP LARC) and short interval birth (SIB) among adolescents in Tennessee (TN).

**Study Design**: This retrospective cohort study linked TN hospital billing data with birth certificate data from Jan 2018 to Dec 2021. Adolescent was defined as < 20 years of age at the index birth event, (Jan 2018‐Dec 2019), exposure status was defined as IPP LARC receipt, and outcome status was defined as a SIB (subsequent birth 18‐ or 24‐months from index birth). We assessed adolescent socio‐demographic characteristics during index birth, stratifying exposure status; differences between groups were assessed using chi‐squared tests. Unadjusted logistic regression assessed the association between IPP LARC receipt and SIB.

**Results**: From Jan 2018 to Dec 2019, 9, 873 TN adolescents gave birth; 122 (1.2%) received IPP LARC. Demographics are presented in the Table. Adolescents who received IPP LARC were 0.28 (95% CI: 0.14–0.58) times less likely to experience a SIB within 24 months of their index birth and 0.13 (95% CI: 0.03–0.54) times less likely to experience a SIB within 18 months of their index birth compared to those that did not receive IPP LARC. Of those receiving IPP LARC, 32.8% (*n* = 40) received an IUD and 67.2% (*n* = 82) received an implant; no differences in IPP LARC type were seen when stratified by maternal age (Figure).

**Conclusion**: Despite the 2017 policy that allowed reimbursement for IPP LARC among patients receiving Tennessee Medicaid, there was low overall uptake (1.2%) among adolescents. Nonetheless, TN adolescent IPP LARC use was associated with significantly lower rates of SIB within 18‐ and 24‐months, compared to adolescents who did not receive IPP LARC. These findings highlight the impact of immediate postpartum contraception in reducing rapid repeat pregnancies and support the need for broader access to both implants and IUDs immediately postpartum.

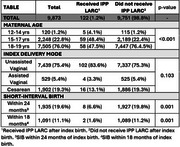

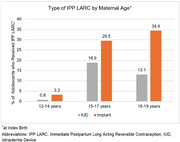

**10:30 AM – 12:00 PM**

## 868 | Prospective **Comparison of two Cost‐Effective Cervical Cerclage Simulation Models for OBGYN Resident Training**

Mary Lou^1^; Danielle Sawka^1^; Theo Addo^1^; Anthony Barisano^1^; Venkatsai Bellala^1^; Regan Cavin^1^; Martha Kole‐White^2^; Alexis C. Gimovsky^2^

^1^Warren Alpert Medical School of Brown University, Providence, RI; ^2^Women & Infants Hospital of Rhode Island / Warren Alpert Medical School of Brown University, Providence, RI

**Objective**: Vaginal suturing techniques needed for proper cerclage placement in management of cervical insufficiency are challenging to teach and learn due to limited operative field visualization. The objective of this study was to compare the use and efficacy of two novel, low‐cost models for cervical cerclage placement in resident training

**Study Design**: A low fidelity plastic and woodmodel and a higher complexity 3‐D printed silicone model of the vaginal canal and cervix were developed by the research team. The plastic model was made out of a 1 liter bottle and OBGYN residents were recruited for a 2‐hour simulation where they practiced cerclage placement on both models. They were assigned randomly to order of model use to reduce performance bias. Residents completed pre‐ and post‐simulation surveys assessing comfort and need for further training using a Likert scale (1 = strongly disagree, 5 = strongly agree). Responses were analyzed using descriptive statistics and paired t‐tests.

**Results**: Twenty‐one OBGYN residents with an average of 2.6 (SD 1.2) years of training participated in the simulation session. Pre‐ and post‐simulation surveys revealed a significant increase in confidence in suturing ability (3.0 vs. 4.3, *p* < 0.001) and decrease in perceived need for further training in cerclage placement (4.3 vs 3.7, *p* = 0.04) (Figure 1). In comparing silicone and plastic

models, residents had a strong preference for the silicone model for future training (4.7 vs 2.5, *p* < 0.001), noting that it had better texture (4.3 vs 1.8, *p* < 0.001) and anatomic realism (4.5 vs 2.3, *p* < 0.001) (Figure 2)

**Conclusion**: Usage of simulation models, particularly the silicone model with improved anatomic realism, for cerclage training increased resident comfort levels with the procedure and enhanced overall resident satisfaction and educational experience.

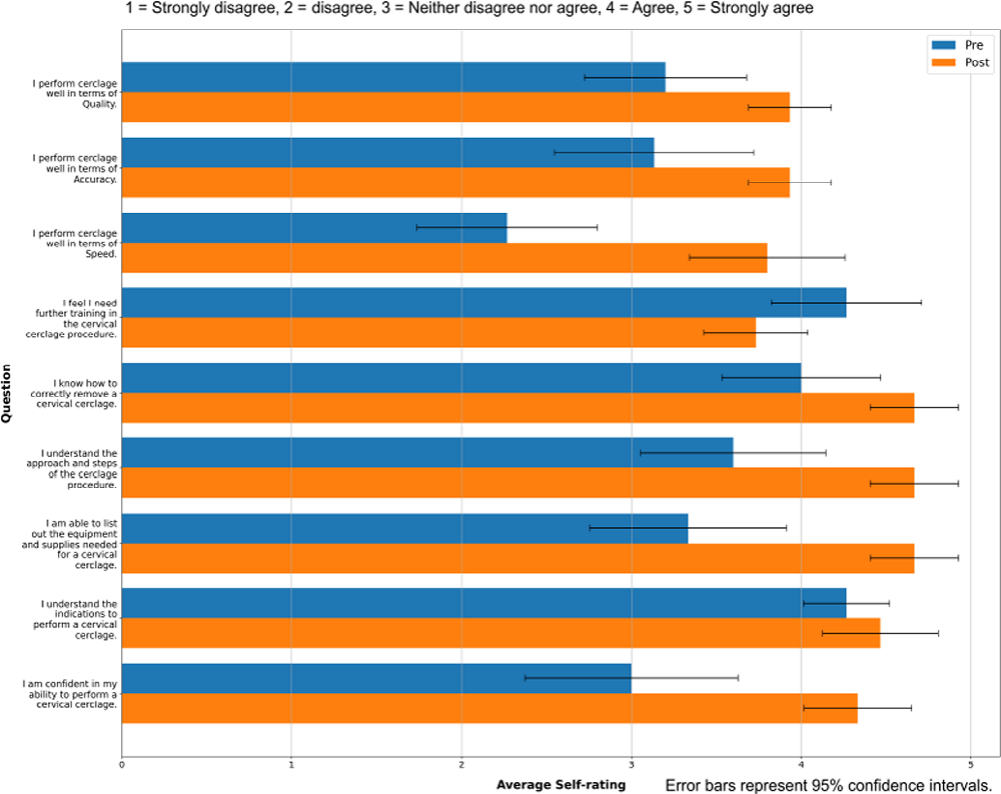

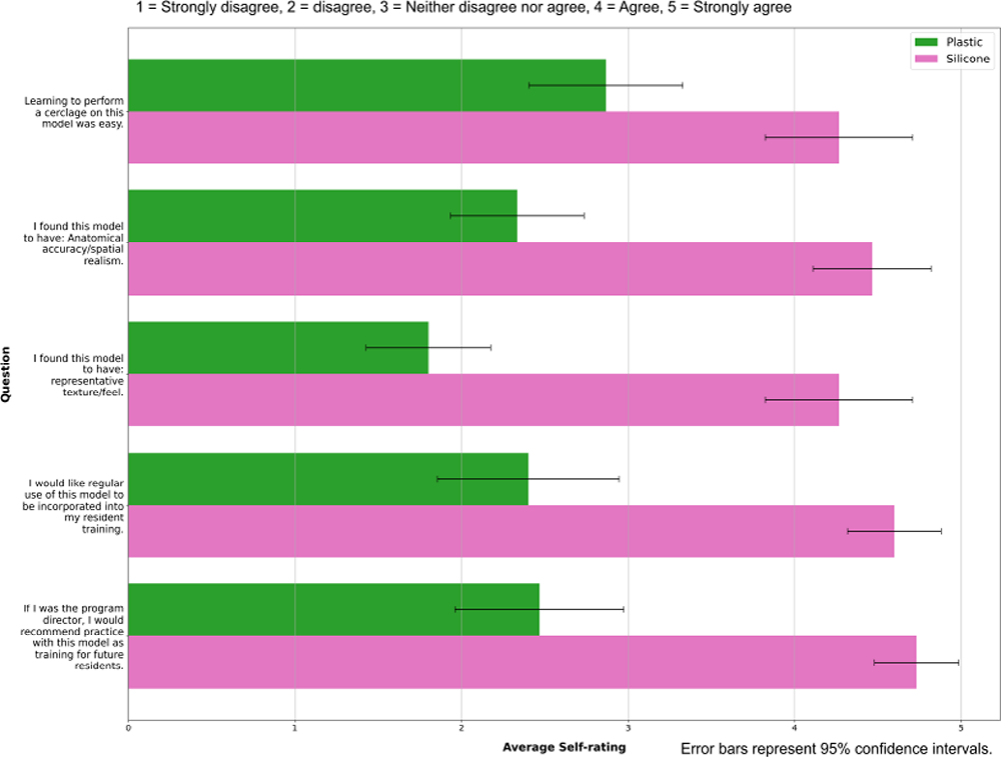

**10:30 AM – 12:00 PM**

## 869 | **Vigorous Exercise is Associated With Reduced Insulin Resistance in Pregnancies at Risk for Gestational Diabetes**

Mary Sedlak^1^; Diana C. Soria‐Contreras^2^; Kaitlyn E. James^3^; Chloe Michalopoulos^4^; Emily Rosenberg^5^; Lydia L. Shook^6^; Tanayott Thaweethai^2^; Camille E. Powe^4^

^1^Harvard Medical School, Boston, MA; ^2^Massachusetts General Hospital, Massachusetts General Hospital/Boston, MA; ^3^Department of Obstetrics and Gynecology, MassGeneral Brigham; Division of Maternal‐Fetal Medicine; Harvard Medical School, Boston, MA; ^4^Massachusetts General Hospital, Boston, MA; ^5^Medical University of South Carolina, Charleston, SC; ^6^Department of Obstetrics and Gynecology, MassGeneral Brigham; Division of Maternal‐Fetal Medicine; Harvard Medical School, Massachusetts General Hospital/Boston, MA

**Objective**: To determine if intensity of physical activity is associated with insulin resistance in pregnant people at risk for gestational diabetes (GDM).

**Study Design**: In a cohort of pregnant people at high risk of GDM (*n* = 102), we used the International Physical Activity Questionnaire‐Short Form (IPAQ‐SF) to estimate hours/week spent sitting, walking, in moderate exercise, or in vigorous exercise at 10–15 and 24–32 weeks' gestation. We used a 75g oral glucose tolerance test (GTT) to calculate the Matsuda index, a measure of insulin sensitivity (opposite of insulin resistance) at 24–32 weeks’ gestation. Our primary hypothesis was that more time spent sitting at 10–15 weeks’ would be associated with greater insulin resistance (lower Matsuda index, log transformed) at 24–32 weeks’ in linear regression models adjusted for age and BMI. Secondary analyses evaluated the relationship between time spent sitting, walking, in moderate physical activity, or in vigorous physical activity at both timepoints and insulin resistance at 24–32 weeks’ gestation. We replicated findings from secondary analyses in a distinct cohort (*n* = 104) using the Pregnancy Physical Activity Questionnaire (PPAQ) for physical activity and Matsuda index calculated using a 100g GTT at 24–32 weeks' gestation.

**Results**: There was no association between hours spent sitting per day at 10–15 weeks’ gestation and insulin resistance at 24–32 weeks’ (β = 0.01, 95% CI [–0.03, 0.05], *p* = 0.67, Figure 1). There was no association of walking or moderate physical activity at either timepoint with insulin resistance at 24–32 weeks’. Vigorous activity at 24–32 weeks’ gestation was associated with lower insulin resistance (β = 0.12, 95% CI [0.04, 0.20]) at the same timepoint. This was replicated in an independent cohort, with increased hours of vigorous physical activity per week at 24–32 weeks' gestation associated with lower insulin resistance cross‐sectionally at 24–32 weeks' in an adjusted model (β = 0.13, 95% CI [0.02, 0.24]).

**Conclusion**: Vigorous activity at 24–32 weeks’ gestation appears protective against insulin resistance in pregnant people with GDM risk factors.

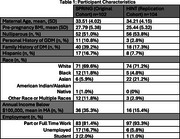

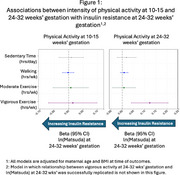

**10:30 AM – 12:00 PM**

## 870 | **New** Insights **of the Starling Law Shows that Preload Determines UA Diastolic Flow**

Masami Yamamoto^1^; Horacio Figueroa^2^; Ignacio J. Valenzuela^2^; Yves Ville^3^

^1^Clinica Universidad de los Andes, santiago, Region Metropolitana; ^2^Clinica Universidad de los Andes, Santiago, Region Metropolitana; ^3^Hôpital Necker Enfants Malades, Hopital Necker Enfants Malades, Ile‐de‐France

**Objective**: Counterintuitive is the finding that, in monochorionic pregnancies affected by twin‐to‐twin transfusion syndrome (TTTS), donors with absent end diastolic flow in the umbilical artery (UA) recover following laser ablation of the anastomosis, which theoretically increases the resistance. The hypothesis is that preload determines cardiac output, explained by the Starling law, and therefore, the UA diastolic flow increases with higher venous flow.

**Study Design**: TTTS cases treated in Clinica Universidad de los Andes. Retrospective analysis of Doppler findings in cases with umbilical vein flow (UVF) measurement before and after laser therapy for TTTS. Linear correlation was calculated for Donor and Recipient UVF with UA‐PI, MCA‐PI, MCA‐PSV and DV‐PI.

**Results**: 46 cases of TTTS were included. There was an inverse correlation between UVF and UA‐PI in donors (R: ‐0, 4763 *p* < 0.001) and recipients (R: ‐0, 3677 *p* < 0.05). This was not found with MCA‐PI or MCA‐PSV. There was an inverse correlation between UVF and DV‐PI in donors (R: ‐0, 3072 *p* < 0.05), but not in recipients.

Following laser, there was no correlation between UA‐PI and venous flow, although only 21 cases had measurements. There was a significant venous flow increase in the donors (median 28 to 72ml/min, *p* = 0.023)< ![if !supportAnnotations] >[IJVS1]< ![endif] > , and no change in the recipients (*p* = 0.73).

Of the 15 Stage 3 cases, 8 had donor demise before discharge, and two more after discharge. Of the 7 donors alive at discharge, all recovered diastolic flow and the mean UVF increase was 46ml/min (4 cases available measurements, 31–60ml/min increase).

**Conclusion**: There was a correlation between UVF and UA end diastolic flow (UA‐PI), supporting that lower preload results in reduced end diastolic flow. In stage 3 donors who survived laser therapy, UVF increased. These findings support the hypothesis that low UVF, rather than placental resistance, is the main determinant of end diastolic flow in TTTS.

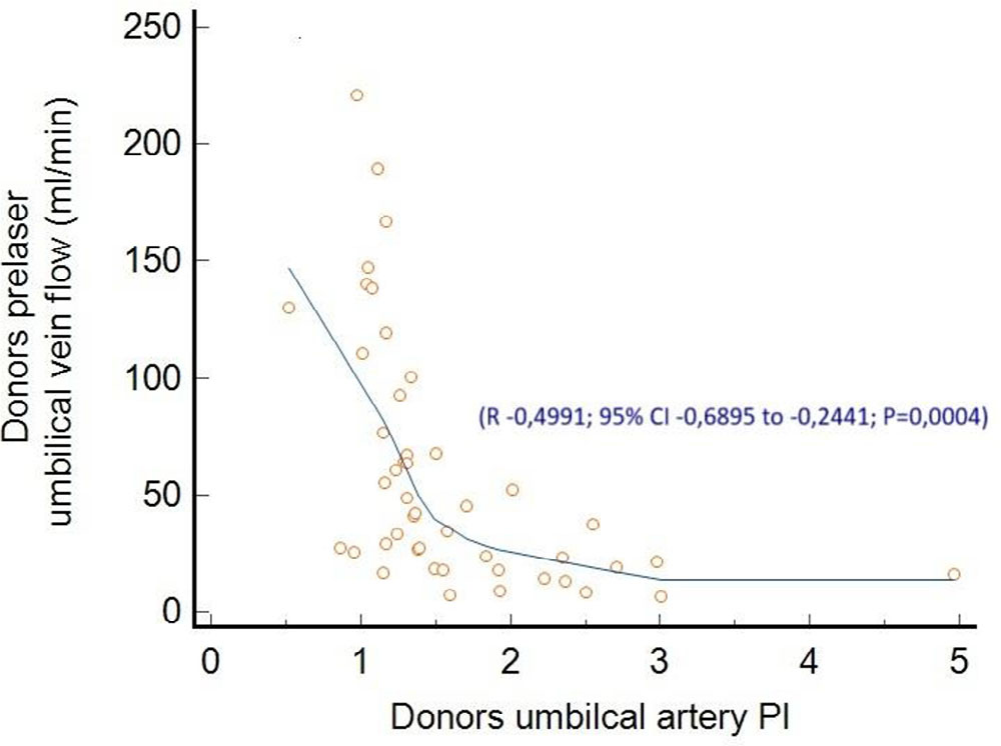

**10:30 AM – 12:00 PM**

## 871 | **Hepatitis B Triple‐Panel Screening in Pregnancy: A Quality Improvement Project**

Matthew H. Mossayebi; Kaitlyn E. James; Malavika Prabhu

Department of Obstetrics and Gynecology, MassGeneral Brigham; Division of Maternal‐Fetal Medicine; Harvard Medical School, Boston, MA

**Objective**: Recent ACOG/SMFM guidelines recommend universal Hepatitis B virus triple‐panel screening once per adult lifetime, with vaccination as indicated. We describe the results of triple panel screening and vaccination after implementation of a quality improvement (QI) initiative in our obstetric population.

**Study Design**: Retrospective cohort study evaluating the implementation of hepatitis B triple panel screening (hepatitis B surface antigen, hepatitis B surface antibody, total hepatitis B core antibody) among all patients establishing prenatal care at a large academic medical center between January and September 2024. The primary outcome was completion of triple panel screening. Process measures included the proportion of hepatitis B susceptible patients with documentation of hepatitis B vaccination recommendations. Triple‐panel results were interpreted per CDC guidance. For patients without testing, charts were reviewed for prior results.

**Results**: 1, 996 patients were included, with a mean age of 33.2 (± 4.8 years), the majority being English‐speaking (88.1%), White (61.7%), and non‐Hispanic (87.1%). The monthly rate of incomplete testing decreased during the study period, ranging from 45% in January 2024 to 4% in August 2024 (Figure 1). 1, 085 (55.2%) were immune to hepatitis B due to prior vaccination, 620 (31.5%) were susceptible to hepatitis B infection, 19 (1%) were immune to hepatitis B due to previous infection, 6 (0.3%) had acute or chronic hepatitis B infection, and 3 (0.2%) had uncertain testing (Table 1). Of the 620 patients susceptible to hepatitis B, 121 (19.5%) had documentation of prior complete hepatitis B vaccination.

**Conclusion**: Rates of susceptibility to the hepatitis B virus in our population were higher than expected. Pregnancy is a time of increased access to care, and given recent FDA approval of a 2‐dose vaccine (Heplisav‐B), vaccination during pregnancy and postpartum is feasible. Our findings demonstrate that triple‐panel screening is feasible across an obstetric population and can enable vaccination of susceptible persons during pregnancy and postpartum.

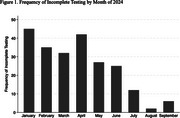

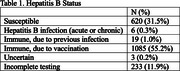

**10:30 AM – 12:00 PM**

## 872 | Examining **Barriers to in‐Office Prenatal Vaccination for Non‐Birthing Partners**

Maxine Slater^1^; Natalie Passarelli^2^; John Soehl^3^; Erica Hardy^1^; Melissa A. Clark^4^; Adam K. Lewkowitz^5^; Laurie B. Griffin^6^

^1^Alpert Medical School of Brown University / Women & Infants Hospital of Rhode Island, Providence, RI; ^2^Beth Israel Deaconess Medical Center, Boston, MA; ^3^University of Wisconsin‐Madison, Madison, WI; ^4^Brown University School of Public Health, Providence, RI; ^5^Women & Infants Hospital of Rhode Island / Warren Alpert Medical School of Brown University, Providence, RI; ^6^University of Utah, Cottonwood Heights, UT

**Objective**: Pediatric guidelines recommend both parents receive Tdap vaccination prior to delivery of their neonate. Despite this, many non‐birthing parents are not up to date on Tdap vaccination, potentially due to lack of education on vaccine importance or access to the vaccination itself. In our randomized control trial that offered in‐office vaccination to non‐birthing partners, many non‐birthing partners did not receive a Tdap. We sought to determine what barriers existed for participation in the prenatal in‐office vaccination program for non‐birthing partners.

**Study Design**: This is a mixed methods study of participants in a RCT in which non‐birthing partners were counseled either in‐person or via telephone on the importance of Tdap vaccination for themselves and protection of their neonate and some were offered in‐office Tdap vaccine administration in the prenatal care office at their convenience. Those who were offered in‐office Tdap vaccination but did not receive this vaccine completed a postpartum survey to elicit barriers to receiving Tdap.

**Results**: Twenty‐two (47.8%) non‐birthing partners who were offered prenatal in‐office vaccination did not receive their Tdap in pregnancy and were included in the current study. The most common self‐reported reason for not receiving vaccination was inability to attend a prenatal care appointment (*n* = 8, 36.3%,) after telephone counseling. Additionally, vaccine hesitancy concerns were also articulated including: belief that vaccination was not needed given personal health status (*n* = 3, 14%), concerns regarding side effects (*n* = 3, 14%), and opposition to vaccination (*n* = 1, 5%).

**Conclusion**: Offering in‐office vaccination increases Tdap vaccination rates for non‐birthing partners, but this solution does not result in universal vaccination due to vaccine hesitancy and barriers to attending prenatal care appointments. Future studies should focus on identifying specific barriers to attending appointments to inform implementation of effective care delivery model in pursuit of encouraging sustainability and optimizing vaccination opportunities.

**10:30 AM – 12:00 PM**

## 873 | Automated **AI‐Based Assessment of Fetal Head Station Through Intrapartum Ultrasound: A Proof of Concept**

Maya Oberman^1^; Amir Kandel^2^; Galor Raviv‐Sharabi^1^; Omri Kempler‐Zarka^1^; Inbal Avrahami^1^; Oren Barak^3^; Edi Vaisbuch^1^; Roni Levy^1^

^1^Kaplan Medical Center, Kaplan Medical Center, HaMerkaz; ^2^Kaplan Medical Center, Kaplan Medical Center, HaZafon; ^3^Kaplan Medical Center, Rehovot, HaMerkaz

**Objective**: This study aimed to develop and evaluate an AI‐based algorithm for automatically assessed fetal head station from intrapartum transperineal ultrasound videos, focusing on the angle of progression (AOP) and head‐perineum distance (HPD), which have not yet been analyzed together by a single automated system.

**Study Design**: A deep learning algorithm was trained on 9, 314 expert‐classified ultrasound frames extracted from 314 ultrasound videos recorded during all stages of labor. Frames were manually categorized into four quality groups: “Good”, “So‐so”, “Bad”, or “Uninteresting”. Frames classified as good or so‐so were further annotated for either AOP or HPD. The model was trained to detect optimal frames and automatically measure AOP, and a second module was developed to perform the same task for HPD. All data were randomly divided into training (80%) and validation (20%) sets using expert annotations as references.

**Results**: The model achieved a mean absolute error of 1.1° (SD 1.5°) for AOP measurement across 4, 025 good or so‐so frames (Figures 1 and 2). For HPD, analysis of 546 annotated images demonstrated a mean absolute error of 1.7 mm (SD 3.5 mm) compared to expert measurements (Figure 1).

**Conclusion**: This proof‐of‐concept AI algorithm facilitates objective and automated evaluation of fetal head descent via intrapartum ultrasound. The approach demonstrates considerable potential for minimizing operator variability and supporting real‐time clinical decision‐making during labor. Current efforts include expanding validation cohorts, increasing and diversifying the training dataset, adding fetal head position analysis, and preparing for clinical implementation.

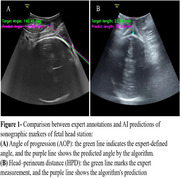

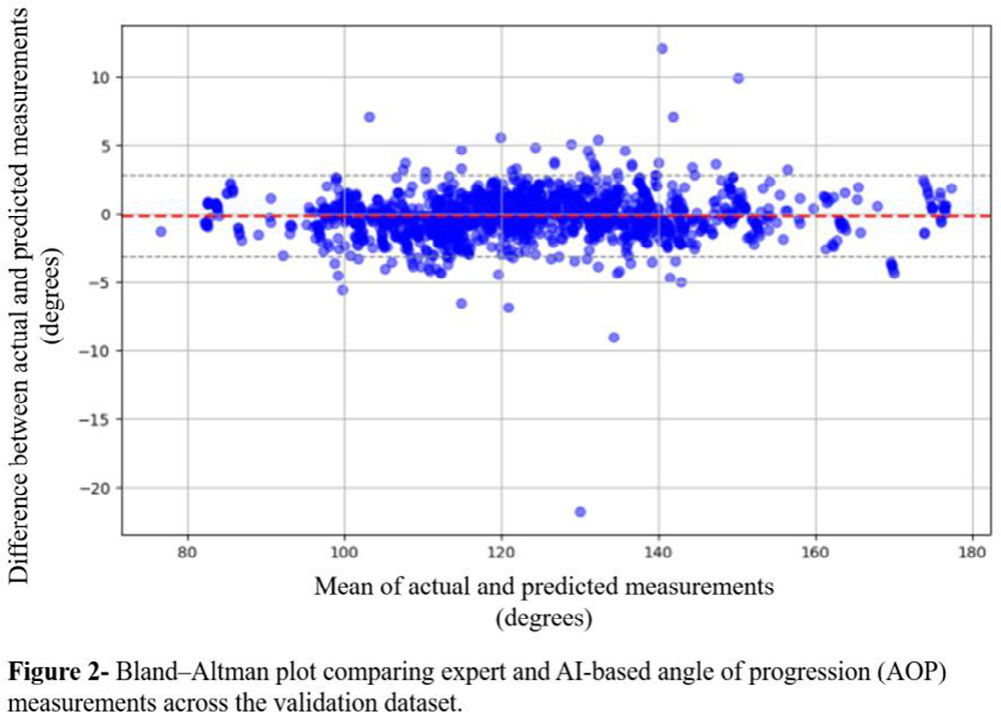

**10:30 AM – 12:00 PM**

## 874 | **Utilizing a Cascade Approach to Identify Gaps in Care Transitions for Hypertensive Disorders of Pregnancy**

Mayra Alejandra Shafique^1^; Bryan L. Aaron^2^; Jonathan Troost^3^; Ashley Williams^2^; Jourdan E. Triebwasser^2^; Molly J. Stout^2^; Natasha R. Kumar^4^

^1^University of Michigan, OBGYN Department, Ann Arbor, MI; ^2^University of Michigan, Ann Arbor, MI; ^3^Michigan Institute for Clinical and Health Research, University of Michigan, MI; ^4^University of Michigan, Department of OBGYN, Ann arbor, MI

**Objective**: Hypertensive disorders of pregnancy (HDP) affect one in 10 pregnancies in the United States, increasing long‐term cardiovascular disease (CVD) risk. However, the minority of individuals with HDP attend a primary care provider visit (PCV) in the first year postpartum. Cascade analysis has been used in other populations to identify gaps in care transitions.

**Study Design**: A stakeholder‐engaged process defined key operational steps in primary care transition, including correct identification of primary care provider (PCP), PCV scheduling and attendance, and CVD counseling at PCV. A cascade analysis identified steps with significant attrition among a retrospective cohort of individuals with HDP who delivered at a single academic center from January to June 2023. Multinomial logistic regression identified predictors of completion of each step.

**Results**: 389 patients were included. Steps with highest levels of attrition were PCV scheduling (–14.1%, Figure 1) and CVD counseling at PCV (−36.0%). In adjusted analysis, public insurance, non‐English language preference (NELP), gestational age at prenatal care initiation and NICU admission predicted attrition (Table 1). Publicly insured individuals were less likely to attend PCV (aOR 0.40, 95% CI 0.22–0.73). Individuals with NELP were more likely to have a correctly identified PCP (aOR 5.66, 95% CI 1.22–26.22) and CVD counseling at PCV (aOR 11.12, 95% CI 1.64–75.25). Individuals who initiated prenatal care later were less likely to have a correctly identified PCP (aOR 0.91, 95% CI 0.85–0.98) and attend PCV (aOR 0.94, 95% CI 0.90–0.99). Individuals with children admitted to NICU were more likely to schedule PCV (aOR 16.13, 95% CI 2.58–100.84).

**Conclusion**: Utilizing a cascade approach to primary care transitions for individuals with HDP identifies operational steps which can be targeted by tailored quality improvement interventions. The most impactful opportunities to mitigate long‐term CVD risk include correct identification of PCP and effective handoffs between OB providers and PCPs communicating this risk profile.

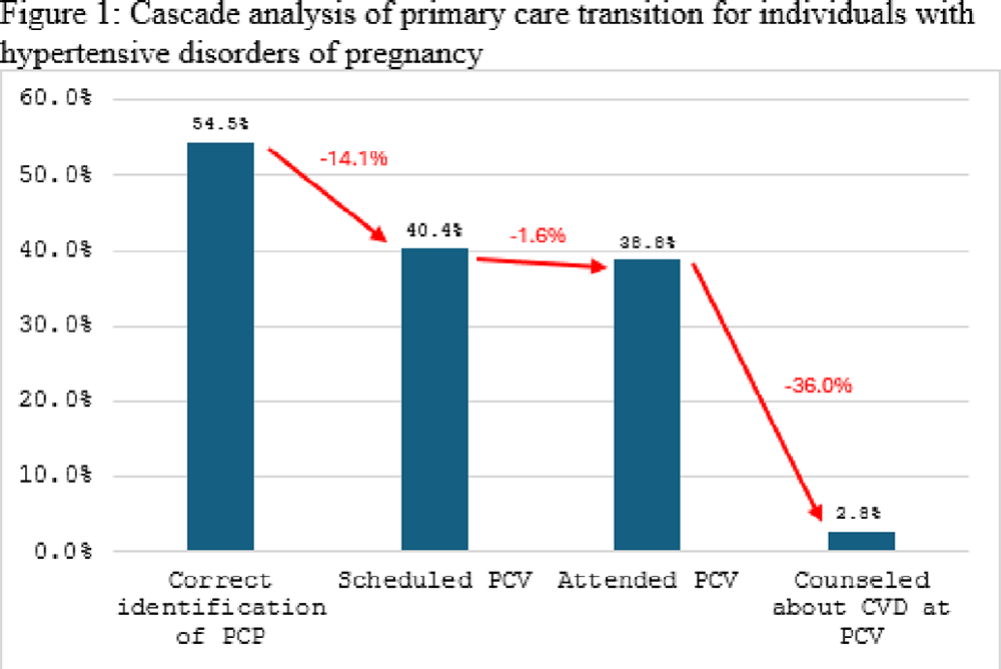

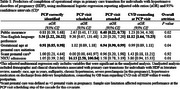

**10:30 AM – 12:00 PM**

## 875 | **The** Impact **of Time of Day on Operative Vaginal Delivery Rate**

Megan Stevenson^1^; Rachel D. Seaman^1^; Lisbet S. Lundsberg^2^; Jennifer F. Culhane^2^

^1^Yale School of Medicine, New Haven, CT; ^2^Department of Obstetrics, Gynecology, and Reproductive Sciences, Yale School of Medicine, New Haven, CT

**Objective**: Despite a decrease in the rate of operative vaginal delivery (OVD), it remains a key obstetric skill and it is valuable to understand patterns of OVD utilization. We sought to identify if time of day at delivery impacts the likelihood of OVD.

**Study Design**: A cohort of 132, 203 deliveries from an academic health system from 2013–2024 was queried for delivery admissions with vaginal mode of delivery. Vaginal deliveries were classified as OVD, using forceps or vacuum, or spontaneous (SVD). Inclusion criteria were vaginal delivery and full data regarding mode of delivery and gestational age at delivery. Time of day at delivery was defined as day shift (0700–1859) and night shift (1900–0659). Bivariate tests of association were conducted to compare patient attributes by time of day at delivery using chi square tests. The primary outcome was the rate of OVD. Crude and adjusted odds ratios were calculated using logistic regression to assess the association between time of day and likelihood of OVD, adjusting for significant confounders.

**Results**: In total, 85, 556 vaginal deliveries were identified: 42, 999 (50.3%) at night and 42, 557 (49.7%) during the day. Bivariate tests showed patients delivered at night were more likely to have a hypertensive disorder of pregnancy and admit BMI >40 but were less likely to have OVD compared to those in the day (5.5% vs 6.5%; *p* < 0.001) (Table 1). Crude odds ratios showed that compared to SVD patients, OVD patients are less likely to have BMI >40, chronic HTN, preterm birth, public insurance, language barrier, or be non‐married. OVD patients were more likely to have singleton gestations. After adjusting for confounders, delivery at night remained associated with a lower likelihood of OVD (aOR 0.83; CI 0.78–0.88). Interestingly, in the multivariate model, a language barrier was associated with increased likelihood of OVD.

**Conclusion**: Among vaginal deliveries, the rate of OVD varied by delivery time. Differences in OVD rates was also seen based on insurance status, marital status, language, BMI, parity, and preterm birth.

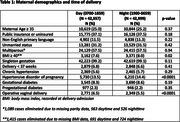

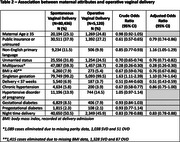

**10:30 AM – 12:00 PM**

## 876 | **Early** Postpartum **Muscle Index Predicts Glycemic Outcomes and β‐Cell Function After Gestational Diabetes**

Megumi Koga^1^; Katsuhito Hayashi^2^; Ichiro Yasuhi^1^

^1^NHO Nagasaki Medical Center, Omura, Nagasaki; ^2^NHO Ureshino Medical Center, Ureshino, Saga

**Objective**: Women with a history of gestational diabetes (GDM) face a high risk of developing type 2 diabetes postpartum. Skeletal muscle is a major site of insulin‐mediated glucose uptake, and low muscle mass may impair glucose regulation. This study examined whether appendicular skeletal muscle index (ASM‐I) in early postpartum is associated with β‐cell compensation and fasting glucose status during two‐year follow‐up in women with prior GDM.

**Study Design**: This secondary analysis used data from a prospective observational study at a tertiary perinatal center in Japan. Singleton women with prior GDM underwent postpartum metabolic assessments including a 75g oral glucose tolerance test (OGTT) at 6–9 weeks, and follow‐up OGTTs at 6 months, 1 year, and 2 years. Appendicular skeletal muscle mass was measured using bioelectrical impedance analysis at 4 weeks postpartum. ASM‐I was calculated as muscle mass divided by body weight. The primary outcome was disposition index (DI) from the first follow‐up OGTT. Secondary outcomes included fasting plasma glucose (FPG) and insulin levels across follow‐up points. Associations were analyzed using multivariable linear regression models.

**Results**: Among 139 women (mean age 34.0 ± 5.0 years; BMI 24.5 ± 5.2 kg/m^2^), higher ASM‐I at 4 weeks postpartum was significantly associated with greater DI at the first follow‐up OGTT (β = 0.28, *p* < 0.005), indicating better β‐cell compensation. ASM‐I was also inversely associated with FPG and insulin levels at 6 months, 1 year, and 2 years (all *p* < 0.05). Women in the lowest ASM‐I quartile had persistently elevated FPG and insulin, suggestive of insulin resistance and β‐cell dysfunction.

**Conclusion**: n women with prior GDM, lower ASM‐I in early postpartum was linked to impaired β‐cell function and sustained fasting hyperglycemia over two years. ASM‐I may serve as a practical, modifiable marker to identify high‐risk women for targeted interventions to prevent type 2 diabetes.

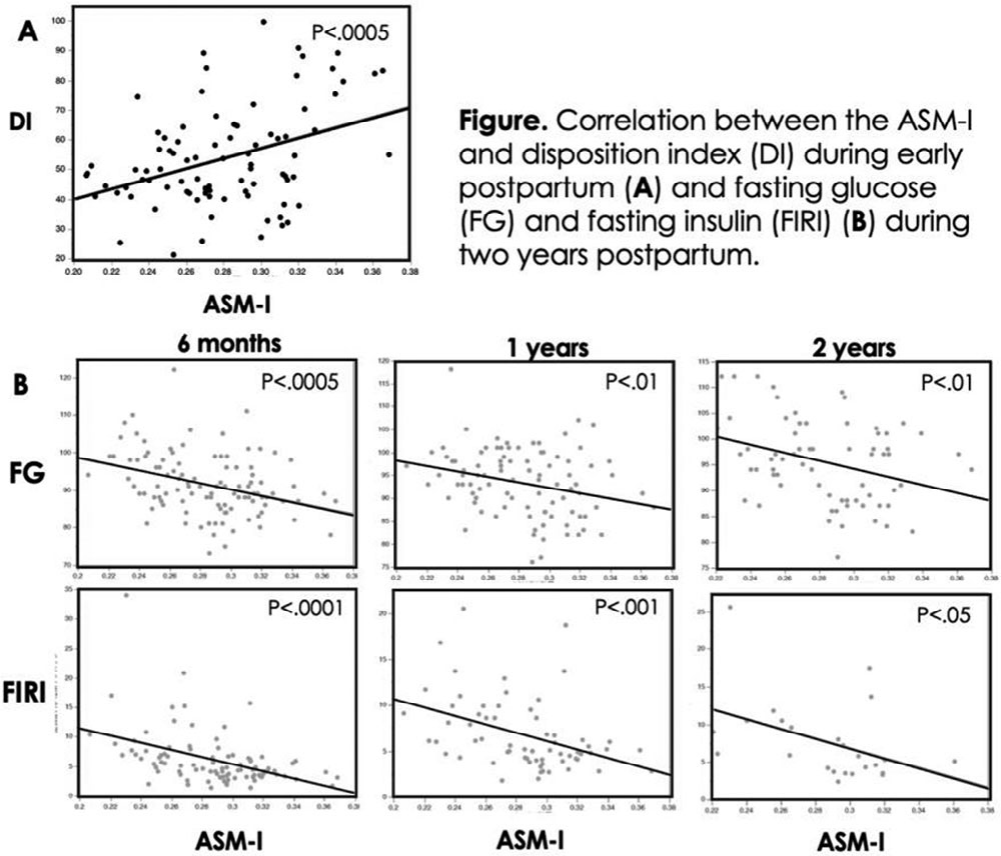

**10:30 AM – 12:00 PM**

## 877 | Management **of Pregnancies With Chronic Hypertension Where Calcium Channel Blockers and Beta‐Adrenergic Blockers are Contraindicated**

Daniel J. Martingano^1^; Melody Boafo^2^; Andrea Rivera^3^; Crystal Awad^4^; Marwah Al‐Dulaimi^5^; Samantha Giler^1^; Jaina Diaz‐Kelly^2^; Kelly Mondey^2^; Kristen Henry^2^; Brenna M. Regan^6^; Kristen Cohen^7^; Iris Gomez‐Brito^8^; Gloria Otoo^2^; Eddie Santana^9^; Jacqueline Marecheau^2^

^1^St. John's Episcopal Hospital‐South Shore / Lake Erie College of Osteopathic Medicine, St. John's Episcopal Hospital‐South Shore / Far Rockaway, NY; ^2^St. John's Episcopal Hospital‐South Shore, St. John's Episcopal Hospital‐South Shore / Far Rockaway, NY; ^3^Wyckoff Heights Medical Center, Wyckoff Heights Medical Center / Brooklyn, NY; ^4^Jamaica Hospital Medical Center, Jamaica Hospital Medical Center / Queens, NY; ^5^St. John's Episcopal Hospital‐South Shore, St. John's Episcopal Hospital‐South Shore, NY; ^6^William Carey University College of Osteopathic Medicine, William Carey University / Hattiesburg, MS; ^7^RWJBarnabas Health Trinitas Regional Medical Center, RWJBarnabas Trinitas Regional Medical Center / Elizabeth, NJ; ^8^RWJBarnabas Trinitas Regional Medical Center, RWJBarnabas Trinitas Regional Medical Center / Elizabeth, NJ; ^9^Good Samaritan University Hospital / NYIT, Good Samaritan University Hospital / West Islip, NY

**Objective**: Calcium channel blockers (CCB) and Beta‐adrenergic blockers (BB) remain essential for pregnancies complicated by chronic hypertension (CHTN), yet optimal alternatives where both CCB and BB are contraindicated remains undefined. This study sought to evaluate the efficacy of alternative antihypertensives for CHTN where both CBB and BB are contraindicated.

**Study Design**: We conducted a multi‐center, prospective observational study from 7/2022 to 7/2025 and included all pregnant women diagnosed with CHTN from 28 0/7 to 41 0/7 weeks‐gestation.

Monotherapy medications included oral hydralazine 50 mg thrice‐daily (HZ), clonidine 0.1 mg/24‐hours weekly patch (CLD), prazosin 5 mg twice‐daily (PRZ), and methyldopa 500 mg thrice‐daily (MDA). Medication choice was based on physician preference. Each regimen was compared to the total patients receiving a different regimen. All patients received aspirin 81 mg daily. Primary outcomes included events of blood pressure >160/110 mm Hg (HE), superimposed preeclampsia (PEC), patient‐reported missed doses (MD), and patient‐reported symptomatic improvement (SI), as discrete events. Patients with cardiac or psychiatric disorders, or multi‐fetal gestations were excluded.

**Results**: The study included 707 patients, with 141 receiving HZ, 133 receiving CLD, 113 receiving PRZ, and 187 receiving MDA. Baseline demographic factors were not significantly different. Patients receiving CLD had lower rates of MD (3.0% v. 29.9% *p* < 0.001) and HE (19.5% v. 28.7%, *p* = 0.003), with a 91% (RR = 0.09, 95% CI 0.01–0.22, *p* < 0.001) and 42% (RR = 0.58 95% CI 0.42–0.71, *p* = 0.040) decreased risk in confounder‐adjusted models, respectively. Patients receiving PRZ had greater rates of SI (69.9% v. 50.7% *p* = 0.002), with a 71% (RR = 1.71, 95% CI 1.63–1.84, *p* = 0.001) increased likelihood in adjusted models. Patients receiving HZ had lower rates of PEC (14.8% v. 20.0%, *p* = 0.040), with a 22% (RR = 0.79, 95% CI 0.46–0.89, *p* = 0.050) decreased risk in adjusted models.

**Conclusion**: PRZ, HZ, and CLD are safe and effective alternatives for pregnancies complicated by CHTN where CCB and BB are contraindicated.

**10:30 AM – 12:00 PM**

## 878 | Development **of Novel Machine Learning Model for the Prediction of Postpartum Readmission for Hypertension Management**

Meralis V. Lantigua‐Martinez^1^; Wenke Liu^2^; Steven Friedman^3^; Ashley S. Roman^1^; David Fenyo^2^; Christina A. Penfield^4^

^1^Division of Maternal‐Fetal Medicine, Department of Obstetrics and Gynecology, NYU Grossman School of Medicine, New York, NY; ^2^Institute for Systems Genetics, NYU Grossman School of Medicine, New York, NY; ^3^Division of Biostatistics, Department of Population Health, NYU Grossman School of Medicine, New York, NY; ^4^Division of Maternal‐Fetal Medicine, Department of Obstetrics and Gynecology, NYU Grossman School of Medicine, New York City, NY

**Objective**: Postpartum hypertension remains a leading cause of emergency department visits and readmissions in the US. Identifying patients at high risk for postpartum readmission may help identify candidates for closer outpatient follow‐up. Machine learning may allow the evaluation of multiple variables to predict high risk patients with increased precision compared to traditional readmission risk models. We developed a machine learning algorithm that predicts patients at risk for postpartum readmission for hypertension management (PRHM).

**Study Design**: We performed an observational cohort study of all births in an academic health system between 1/1/20 and 6/30/24. Predictive models for PRHM were developed using random forest and XGBoost. 54 variables associated with PRHM were considered in the model. Hyperparameters were tuned with a grid search over 5‐fold cross‐validation and model performance was evaluated with area under the curve for precision‐recall (AUCPR) and receiver operating characteristic (AUCROC). The best hyperparameter set was selected as the combination that yields the highest mean AUCPR across folds. Feature importance was retrieved from the best model.

**Results**: Of 56, 147 deliveries among 50, 467 patients, 730 (1.3%) resulted in readmission for hypertension management. Both random forest and XGBoost showed high AUCROC (random forest: 0.923 ± 0.005; XGBoost: 0.928 ± 0.004, mean ± standard error), but low AUCPR (random forest: 0.123 ± 0.007; XGBoost: 0.162 ± 0.010). At the optimal F1 score, specificity was around 98%, sensitivity ranged from 28.4%–43%, negative predictive value (NPV) was 97–98%, and positive predictive value (PPV) 15%–16% (Table 1). For both algorithms, a history of pregnancy related hypertension contributed the most to the final prediction (Figure 1).

**Conclusion**: Despite high overall accuracy and specificity, both models had poor positive predictive value in identifying patients at risk for PRHM, limiting clinical applicability. Our findings highlight the challenges of applying machine learning to rare outcomes like postpartum readmission.

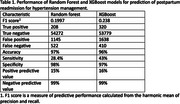

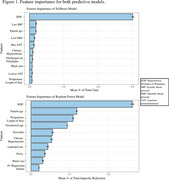

**10:30 AM – 12:00 PM**

## 879 | **Obstetric Outcomes After Cervical Conization: A Retrospective Cohort from a Large Tertiary Center**

Michal Axelrod^1^; Chen Amichay^2^; Avihu Krieger^3^; Shlomi Toussia‐Cohen^4^; Limor Helpman^2^

^1^Sheba Medical Center, Sheba Medical Center, HaMerkaz; ^2^Sheba Medical Center, Ramat Gan, HaMerkaz; ^3^The Department of Obstetrics and Gynecology, The Chaim Sheba Medical Center, Ramat‐Gan, Israel, Ramat Gan, Tel Aviv; ^4^The Sheba Medical Center, The Sheba Medical Center, HaMerkaz

**Objective**: To evaluate the association between prior cervical conization and obstetric outcomes, particularly preterm birth, compared to the general obstetric population.

**Study Design**: This retrospective cohort study was conducted at a large tertiary center between 2011 and May 2025. Women who delivered following cervical conization (*n* = 61) were compared to all other deliveries during the same period (*n* = 150, 276). The primary outcome was preterm birth < 37 weeks. Secondary outcomes included preterm birth < 34 weeks, mode of delivery, and neonatal outcomes. Multivariable logistic regression adjusted for maternal age, BMI, nulliparity, multifetal gestation, history of preterm labor, and smoking.

**Results**: Preterm birth < 37 weeks occurred more frequently in the conization group (14.8% vs. 2.9%, aOR 5.54, 95% CI 2.69–11.39, *p* < 0.001). However, the median gestational age remained within term (38.4 vs. 39.1 weeks, *p* = 0.026), and birth < 34 weeks was comparable. There was no significant association between time from conization to delivery and risk of preterm birth (*p* = 0.656). Cesarean delivery was more common after conization (42.6% vs. 28.3%, *p* = 0.013), with a trend toward unplanned CD. Rates of placental abruption (3.3% vs. 0.5%, *p* = 0.037) and NICU admission (5.0% vs. 0.6%, *p* = 0.005) were higher. No differences were observed in birthweight, Apgar scores, or neonatal mortality.

**Conclusion**: Cervical conization is associated with increased risk of preterm birth and selected maternal and neonatal complications. Still, most women deliver at term with reassuring neonatal outcomes. These findings support individualized prenatal counseling and enhanced monitoring in pregnancies following cervical excisional procedures.

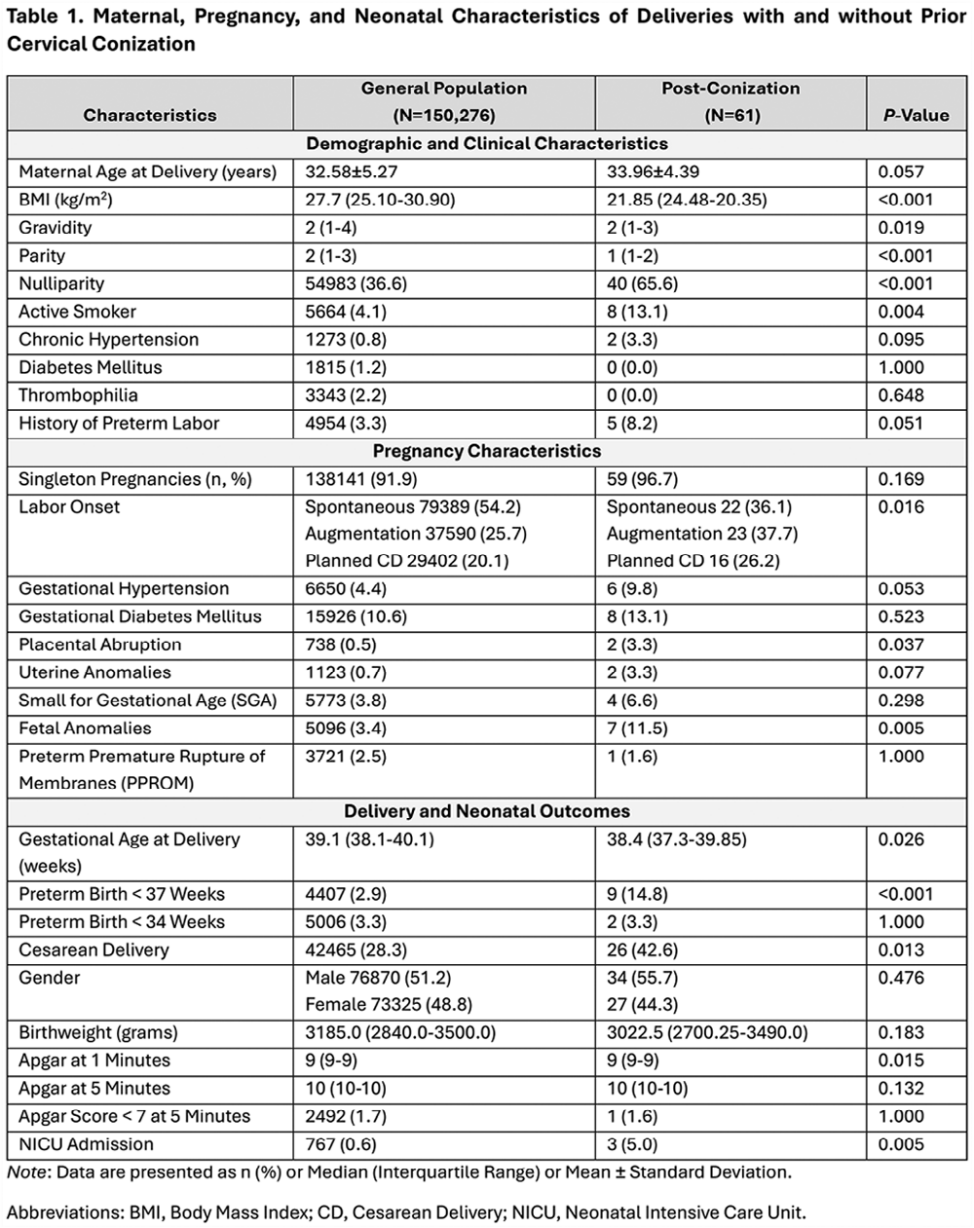

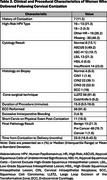

**10:30 AM – 12:00 PM**

## 880 | Improvement **in Labor Dystocia Adherence Varies Widely in a Statewide Quality Collaborative**

Michelle Moniz^1^; Daniel Almiral^2^; Rebecca Wardrop^2^; Jourdan E. Triebwasser^2^; Lisa Kane Low^2^; Brian Torok^3^; Molly J. Stout^2^

^1^University of Michigan, University of Michigan, MI; ^2^University of Michigan, Ann Arbor, MI; ^3^Corewell Health, Troy, MI

**Objective**: To characterize real‐world patterns of adoption of ACOG/SMFM criteria for dystocia as the primary indication for unplanned cesarean birth.

**Study Design**: Retrospective analysis of nulliparous, term, singleton, vertex births in the registry of the Obstetrics Initiative (OBI), a statewide quality collaborative funded by Blue Cross Blue Shield of Michigan and Blue Care Network as part of the BCBSM Value Partnerships program. During the eight quarter study period (4/2021–3/2023), all hospitals received BASE, OBI's standard model of QI support. BASE includes 1) clinician education in dystocia criteria, 2) monthly hospital performance reports, 3) online toolkit, and 4) small financial incentives for hospital participation. Hospitals with any quarterly dystocia case count < 1 were excluded. We categorized each hospital's performance by: 1) *dystocia adherence rate* in quarters 1, 5, and 8 (defined as high if >quarter 1 75th percentile; all others = low); and 2) *dystocia adherence rate trend* (increasing vs. no change, using the time variable's coefficient in logistic regression models estimated for each site, for each year). Using a matrix, we grouped sites with similar adherence rate/trend combinations to identify distinct adoption phenotypes.

**Results**: Of 70 OBI hospitals, 50 were eligible for analysis. Five performance phenotypes emerged (Table 1; Figure 1): High Adherence (*n* = 10, yellow line‐starts high, ends high); Rapid Adopters (*n* = 11, teal line‐starts low, rises rapidly, ends high); Regressors (*n* = 9, light blue line‐starts low, rises, then falls); Slow Adopters (*n* = 11, burgundy line‐starts low, rises steadily); and Non‐Adopters (*n* = 9, pink line‐starts low, ends low).

**Conclusion**: In a statewide dystocia initiative, hospital response to BASE QI support varied in timing, magnitude, and duration. An adaptive QI strategy that starts with BASE and “steps up” to more intensive QI support for hospitals that do not rapidly respond, and minimizes QI support resources allocated to high‐performing sites, is critically needed to effectively and efficiently address heterogeneous QI needs on inpatient maternity units.

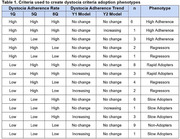

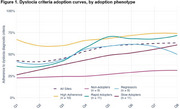

**10:30 AM – 12:00 PM**

## 881 | **Parity‐**Specific **Predictors of Obstetric Anal Sphincter Injury: Real‐Time Insights for Clinical Decision‐Making**

Eiman Shalabna^1^; Rawia Hussein‐Aro^1^; Milana Gelman^2^; Basel Habib Nasser^3^; Noa Haggiag^1^; Moran Gawie‐Rotman^1^; Nashwa Fadaos^1^; Rawan Daher^1^; Baraa Darawshe^1^; Adi Malkoff Rabin^4^; Amir Naeh^1^; Rinat Gabbay‐Benziv^5^

^1^Hillel Yaffe Medical center, Hadera, HaZafon; ^2^Department of Obstetrics and Gynecology, Hillel Yaffe Medical Center, Netanya, HaMerkaz; ^3^Hillel Yaffe Medical center, Hadera, Israel; Rappaport Faculty of Medicine, Technion – Israel Institute of Technology, Haifa, Israel, Hadera, HaZafon; ^4^Hillel Yaffe Medical center, Binyamina, HaZafon; ^5^Hillel Yaffe Medical Center, Hillel Yaffe Medical Center, HaMerkaz

**Objective**: To identify maternal and intrapartum predictors of obstetric anal sphincter injury (OASIS) using clinically available data at term, stratified by parity, and to challenge the predictive value of traditional markers such as estimated fetal weight (EFW) and second‐stage duration.

**Study Design**: We conducted a retrospective cohort study of 22, 220 singleton, term vaginal deliveries (2018–2024) at a single tertiary center. Exclusion criteria included multiple gestations and caesarean delivery before full dilation. Data included maternal demographics, pregnancy characteristics, EFW (assessed ≤7 days before delivery), and intrapartum factors. Logistic regression was used to identify independent risk factors, stratified by parity. Birthweight was excluded from models to ensure real‐time clinical applicability.

**Results**: OASIS occurred in 0.46% of births (102/22, 220), with higher rates among nulliparas (0.92%) vs. multiparas (0.23%). In multivariable analysis of the full cohort, nulliparity (aOR 3.00, 95% CI: 1.67–5.39), vacuum‐assisted delivery (aOR 2.49, 95% CI: 1.43–4.32), EFW >3500g (aOR 1.67, 95% CI: 1.01–2.76), and meconium‐stained fluid (aOR 1.75, 95% CI: 1.00–3.05) were associated with increased risk. In parity‐stratified models, only vacuum extraction predicted OASIS among nulliparas (aOR 2.31, 95% CI: 1.25–4.28), while prior cesarean predicted OASIS among multiparas (aOR 3.62, 95% CI: 1.31–10.00). Neither EFW nor second‐stage duration remained significant in stratified models.

**Conclusion**: This is one of the largest single‐center OASIS cohorts to date. The study uniquely models risk using only variables available before or during labor, enhancing clinical relevance. Findings highlight that OASIS is largely unpredictable; EFW and second‐stage duration have limited utility, while first vaginal birth—whether in nulliparas or VBACs—and vacuum delivery are key risks. These results support universal perineal protection strategies rather than selective risk‐based approaches.

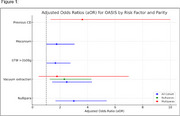

**10:30 AM – 12:00 PM**

## 882 | **Maternal Body Mass Index Class and Rates of Successful Preterm Induction of Labor**

Minhazur R. Sarker^1^; Grace K. Noonan^2^; Rachel L. Wiley^1^; Daphne Yvette Lacoursiere^2^; Daniella Rogerson^2^; Timothy Wen^1^; Cynthia Gyamfi‐Bannerman^1^; Ukachi N. Emeruwa^3^

^1^Division of Maternal Fetal Medicine, Department of Obstetrics, Gynecology and Reproductive Sciences, University of California San Diego, San Diego, CA; ^2^Department of Obstetrics, Gynecology and Reproductive Science, University of California San Diego, San Diego, CA; ^3^University of California San Diego, San Diego, CA

**Objective**: Preterm induction of labor (IOL) is associated with a high perceived baseline rate of failure especially among patients with higher morbidity such as maternal obesity. We aimed to characterize cesarean delivery (CD) rates among preterm IOL stratified by maternal BMI class.

**Study Design**: This is a cross‐sectional study of United States National Vital Statistics System birth data from 2016–2023 to identify singleton, vertex, non‐anomalous pregnancies undergoing preterm IOL between 28w0d–36w6d. The primary exposure and outcome were maternal BMI class at delivery and CD, respectively. Multivariable logistic regression assessed the association between maternal BMI class and CD using normal weight (BMI 18.5–24.9) as the reference. Results were adjusted for maternal age, self‐reported maternal race and ethnicity, payer status, gestational age at delivery, diabetes and hypertensive disorders, and macrosomia (birthweight > 4000 g).

**Results**: 499, 685 individuals with mean gestational age of 34.8 ± 1.6 weeks met our inclusion criteria: 14, 704 (2.8%) underweight, 164, 444 (32.9%) normal, 132, 516 (26.5%) overweight, 92, 683 (18.5%) class I, 52, 304 (10.5%) class II, 36, 846 (7.4%) class III, 5, 896 (1.2%) class IV, and 742 (0.2%) class V. Increasing BMI class was associated with increasing CD rate: 11.4% underweight, 14.2% normal, 17.6% overweight, 21.0% class I, 24.9% class II, 30.7% class III, 36.7% class IV, and 44.2% class V (Figure 1a, *p* < 0.01). In the adjusted regression, using normal weight as the reference, increasing BMI class was associated with CD (aOR; 95% CI): underweight (0.78; 0.74–0.83), overweight (1.31; 1.29–1.34), class I (1.61; 1.57–1.64), class II (1.94; 1.89–1.99), class III (2.47; 2.40–2.54), class IV (3.00; 2.83–3.19), and class V (3.85; 3.29–4.51) (Figure 2a). When sub‐analyzing only nulliparous patients undergoing preterm IOL, higher CD rates were appreciated with stronger associations with CD (Figure 1b and 2b).

**Conclusion**: Our findings provide quantitative preterm IOL success rates and highlight that many patients undergoing preterm IOL delivery vaginally.

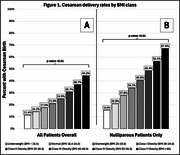

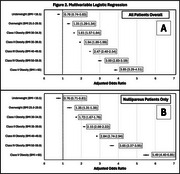

**10:30 AM – 12:00 PM**

## 883 | **Maternal Respiratory Syncytial Virus Vaccination Reduces Infant Risk of Any Respiratory Infection**

Minhazur R. Sarker^1^; Gregory W. Poorman^2^; Timothy Wen^1^; Rachel L. Wiley^1^; Barbara Levy^2^; Patrick Edmundson^2^; Ukachi N. Emeruwa^3^; Leah M. Lamale‐Smith^1^; Cynthia Gyamfi‐Bannerman^1^

^1^Division of Maternal Fetal Medicine, Department of Obstetrics, Gynecology and Reproductive Sciences, University of California San Diego, San Diego, CA; ^2^Dorsata, Inc., Arlington, VA; ^3^University of California San Diego, San Diego, CA

**Objective**: Respiratory syncytial virus (RSV) is a major contributor to infant morbidity and mortality. Recently, an antenatal RSV vaccine delivered between 32–36 weeks' gestation during RSV season was recommended to mitigate newborn complications. We aimed to assess the real‐world efficacy of RSV vaccination on birth outcomes and newborn respiratory illnesses.

**Study Design**: This retrospective cohort study utilized the Dorsata database, a structured prenatal electronic medical record with linked infant outcomes. Pregnant individuals eligible for RSV vaccination (defined as having at least two gestational weeks between 32–36 week) during the 2023–24 RSV season with live births >34 weeks’ gestation were included. Infant outcomes (RSV and other respiratory illness diagnoses within the first year of life) and birth safety outcomes (preterm birth and preeclampsia) were compared by vaccination status as primary exposures. Adjusted logistic regression models were fit to assess the relationship between maternal vaccination status and infant outcomes, adjusting for clinical and demographic differences.

**Results**: Of the 2, 404 eligible patients, 1, 955 (81.3%) did not receive the RSV vaccine. Among those vaccinated, there were lower rates of NICU admission (4.7% vs. 7.9%, *p* = 0.03), and infant RSV diagnosis (0.2% vs. 1.4%, *p* = 0.04) and any infant respiratory illness (8.7% vs. 16.1%, *p* < 0.01) within the first year of life (Table 1). Adjusted regression analysis noted a 47% decreased likelihood of having any infant respiratory illness (aOR 0.53, 95% CI 0.37–0.76, Table 2) among infants born to vaccinated individuals. There was no significant difference in infant RSV illness in the adjusted analysis, likely limited by small sample size. There were no differences in maternal safety outcomes by vaccination status (Table 1).

**Conclusion**: Using real‐world data, we found that antenatal vaccination with RSV decreased the incidence of any infant respiratory illnesses within the first year of life and was not associated with higher maternal risk. Our findings support the ongoing recommendation for antenatal RSV vaccination.

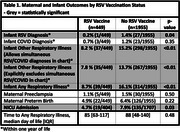

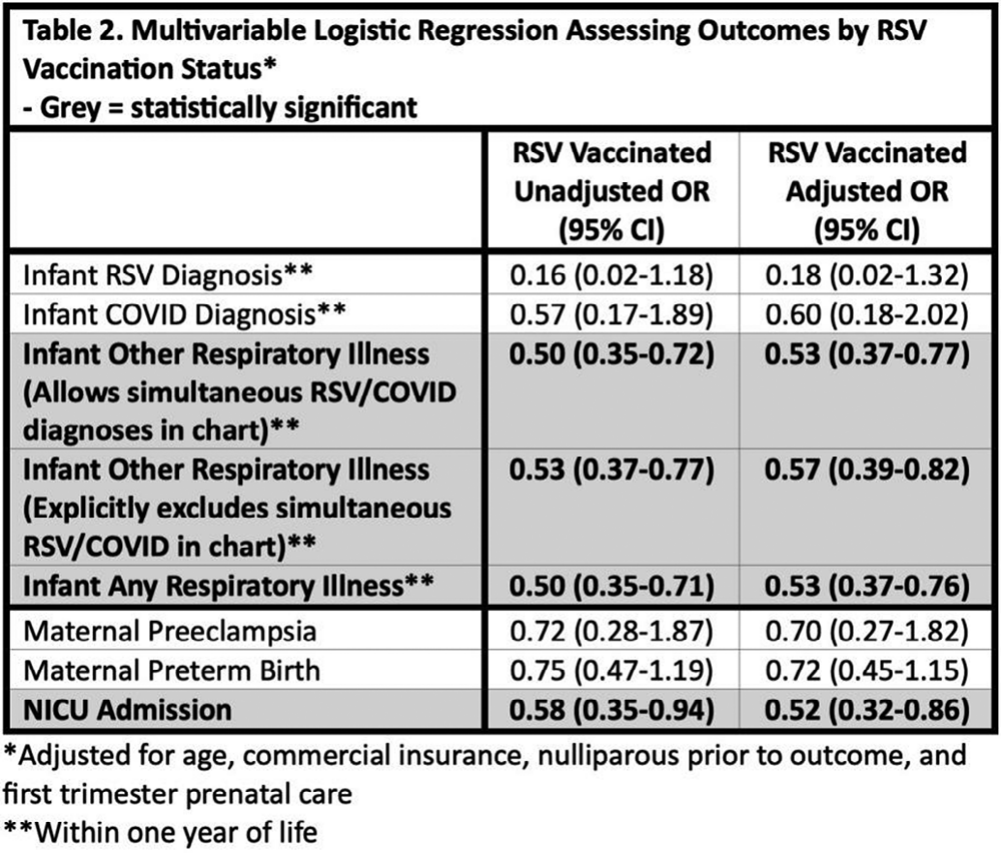

**10:30 AM – 12:00 PM**

## 884 | **Mean** OGTT **Deviation as a Predictor of GDMA2 and Subsequent Glycemic Control**

Moran Weiss^1^; Shaked Yarza^2^; Shaked Zeevi^3^; Hadar Gluska^1^; Ella Pardo^4^; Tal Biron‐Shental^1^; Omer Weitzner^1^

^1^Meir Medical Center, Kfar Saba, HaMerkaz; ^2^Meir Medical Center, kefar saba, HaMerkaz; ^3^Meir Medical Center, Kefar Saba, HaMerkaz; ^4^Meir Medical Center, Meir Medical Center, Kfar Saba, Israel, HaMerkaz

**Objective**: The oral glucose tolerance test (OGTT) is a key diagnostic tool for identifying gestational diabetes mellitus (GDM), and the number of abnormal values correlates with adverse obstetric outcomes. However, the clinical significance of the average degree of glycemic deviation above the normal ranges of OGTT has yet to be investigated. We aimed to evaluate whether an average OGTT deviation exceeding 10% is associated with an increased risk of requiring medical therapy for glycemic control (GDMA2) and the degree of glycemic control among treated patients.

**Study Design**: This retrospective cohort study included 1, 038 women diagnosed with GDM who had at least two abnormal values on the OGTT over 8 years. For each participant, we calculated the mean percentage deviation above the OGTT diagnostic thresholds. Patients were classified according to their average OGTT deviation: >10% vs. ≤10%. GDMA2 rates were assessed and compared between the two groups. A subgroup analysis was performed to evaluate glycemic control among women with GDMA2.

**Results**: Among the 1, 038 women included, 532 (51.3%) had an average OGTT deviation >10%, and 506 (48.7%) had a deviation ≤10%. Women in the >10% group had a significantly higher rate of GDMA2 (adjusted OR 1.39; 95% CI 1.06–1.82, *p* = 0.02). When modeled as a continuous variable, increasing OGTT deviation was also associated with greater odds of GDMA2 (*p* = 0.04). Among those with GDMA2, A mean OGTT deviation greater than 10% was significantly associated with a higher proportion of unbalanced gestational diabetes (73.4% vs 62%, *p* = 0.042). No significant differences were observed between the groups in maternal or neonatal composite outcomes.

**Conclusion**: Among women with GDM and ≥2 abnormal OGTT values, a mean glycemic deviation >10% is associated with increased likelihood of requiring pharmacologic treatment and poorer glycemic control at delivery. These findings suggest that quantifying the extent of glycemic abnormality at diagnosis may serve as an early risk stratification tool in GDM management.

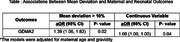

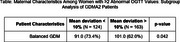

**10:30 AM – 12:00 PM**

## 885 | **Planned mode of Delivery in Preterm Births Complicated by Hypertension: The Impact of Clinical/Sociodemographic Characteristics**

Moti Gulersen^1^; Itai Hazan^2^; Erez Lenchner^3^; Vincenzo Berghella^4^; Eran Bornstein^5^

^1^Thomas Jefferson University, PHILADELPHIA, PA; ^2^Ben‐Gurion University of the Negev, Beer Sheva, HaDarom; ^3^New York University Rory Meyers College of Nursing, New York, NY; ^4^Thomas Jefferson University, Philadelphia, PA; ^5^Northwell, New Hyde Park, NY

**Objective**: To evaluate the planned mode of delivery in preterm births (PTBs) complicated by hypertensive disorders of pregnancy (HDP) and whether clinical and sociodemographic factors influence it.

**Study Design**: Retrospective analysis of the Centers for Disease Control and Prevention Natality Live Birth database (2016–2023). All nulliparous, singleton, in‐hospital, live births with HDP (i.e. gestational hypertension or preeclampsia) from 23 0/7–36 6/7 weeks of gestation were eligible for inclusion. Births with congenital anomalies, non‐cephalic presentation, or missing data were excluded. Preterm births were stratified into 3 gestational age (GA) groups: 23 0/7–27 6/7, 28 0/7–33 6/7, and 34 0/7–36 6/7 weeks. Several clinical and sociodemographic factors were compared between patients undergoing a trial of labor (TOL) and those who had a planned cesarean delivery (PCD) within each GA group. Multivariable logistic regression was performed to evaluate the association of each factor with odds of TOL. Data are presented as adjusted odds ratios (aOR) with 95% confidence intervals (CI).

**Results**: Of the 147, 944 nulliparous, singleton, HDP patients at 23–36 weeks included, 66.1% underwent a TOL and 33.9% PCD. Rates of TOL were lowest in the earliest GA group and increased with advancing GA (23 0/7–27 6/7 weeks: 19.0% [1, 016/5, 355], 28 0/7–33 6/7 weeks: 39.4% [13, 816/35, 101], 34 0/7–36 6/7 weeks: 77.2% [83, 001/107, 488] (Figure, *p* < 0.01). Clinical factors such as older maternal age and higher body mass index were associated with increased odds of PCD (Table). Sociodemographic factors such as maternal education, race and ethnicity, and insurance type had minimal to no impact on the mode of delivery (Table).

**Conclusion**: Mode of delivery in nulliparous PTBs complicated by HDP is GA dependent, with PCD much more common at early GAs. Clinical factors such as older maternal age and higher body mass index were associated with increased odds of PCD. These findings suggest that delivery planning in nulliparous PTBs complicated by HDP is primarily influenced by GA, maternal age, and BMI.

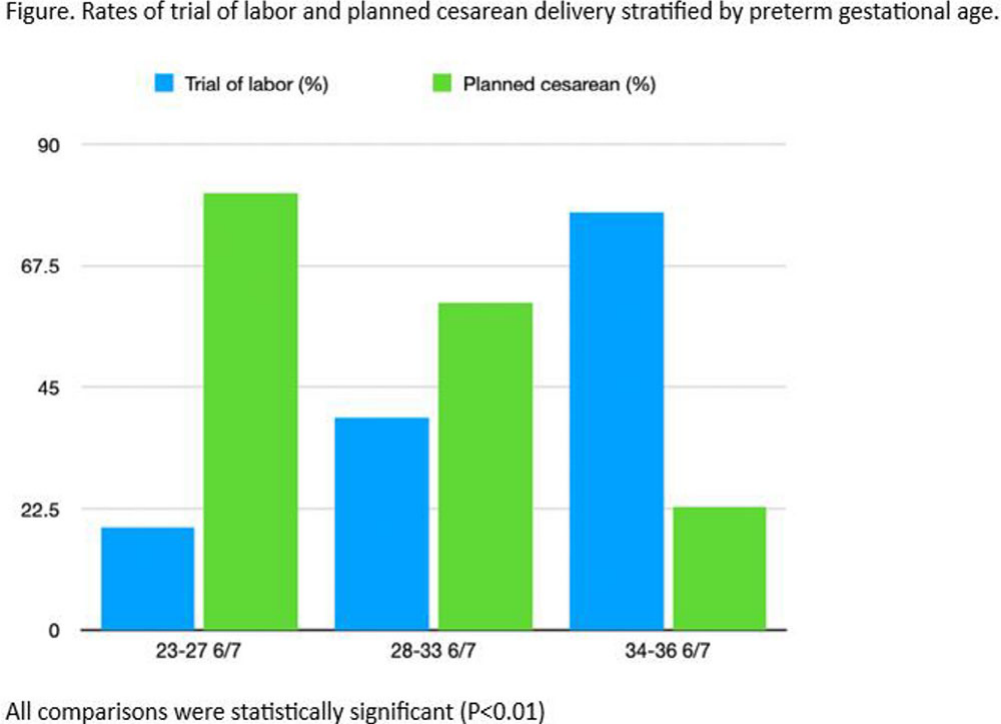

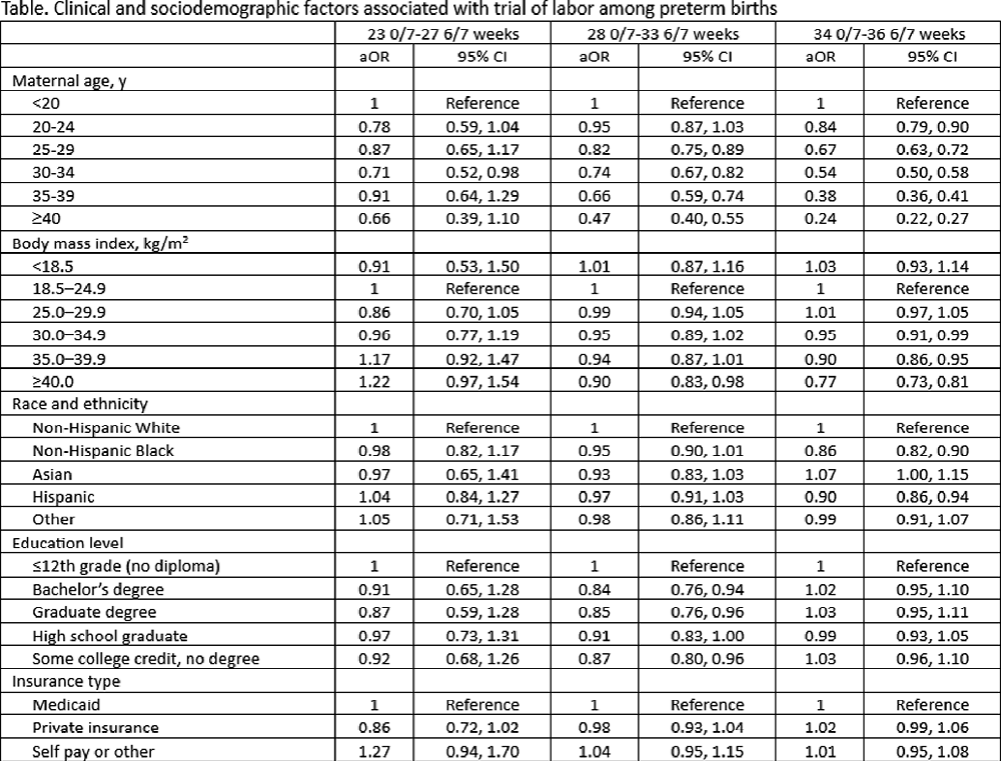

**10:30 AM – 12:00 PM**

## 886 | **Umbilical Vein Spectral Analysis to Predict Hypertensive Disorders of Pregnancy and Maternal Vascular Malperfusion**

Jingyi Zhu^1^; Claudia Tawil^2^; Xing Yao^3^; Baris Oguz^2^; Gabriel A. Arenas^2^; Rebecca L. Linn^2^; Ipek Oguz^4^; Brett C. Byram^4^; Nadav Schwartz^2^

^1^Vanderbilt University, Philadelphia, PA; ^2^University of Pennsylvania, Philadelphia, PA; ^3^Vanderbilt University, Nashville, TN; ^4^Vanderbilt University, Vanderbilt University, TN

**Objective**: While the umbilical vein (UV) carries nutrient‐rich blood to the fetus, conventional UV Doppler techniques have not provided an effective tool for assessing placental function. We present a UV Doppler‐based workflow to predict placental dysfunction, including hypertensive disorders of pregnancy (HDP), small‐for‐gestational‐age (SGA), maternal vascular malperfusion (MVM), and fetal vascular malperfusion (FVM), by quantifying maternal‐to‐fetal spectral power.

**Study Design**: UV Doppler waveforms were acquired at 18–24 weeks gestation in a prospective cohort study of singleton pregnancies. Sonographers were instructed to obtain 1–3 extended‐duration cine clips of UV Doppler near the placental cord insertion. Frames of cine clips were stitched into long sequences using OpenCV's feature‐based pipeline to improve spectral resolution. UV Doppler envelopes were extracted using Rubin's method (Fig 1a). Welch's method was applied for spectral estimation due to its robustness to envelope variation caused by fetal motion or maternal breathing. The log ratio of the spectral peaks at the maternal and fetal heart rates (LRSP) was assessed as a predictor of placental dysfunction.

**Results**: 70 subjects were included in the analysis, with a mean of 1.77 cine clips (SD = ±0.59) per subject, each lasting an average of 6.89 seconds (SD = ±1.81). Cohort details are provided in Table 1. LRSP was significantly higher in pregnancies that later developed HDP (*p* = 0.0181, AUC = 0.7) and in those with MVM (*p* = 0.0436, AUC = 0.8) (Fig 1bc). This indicates a greater relative contribution of maternal power to the UV Doppler in pathological cases compared to healthy subjects. No significant association was found with SGA (*p* = 0.17) or FVM (*p* = 0.78).

**Conclusion**: UV Doppler spectral estimation can be used to assess the relative maternal and fetal contributions to UV flow patterns and may serve as a non‐invasive early marker of  placental dysfunction. Current validation includes 4 confirmed MVM cases; ongoing pathology follow‐up and larger cohorts are expected to further establish the method's predictive value.

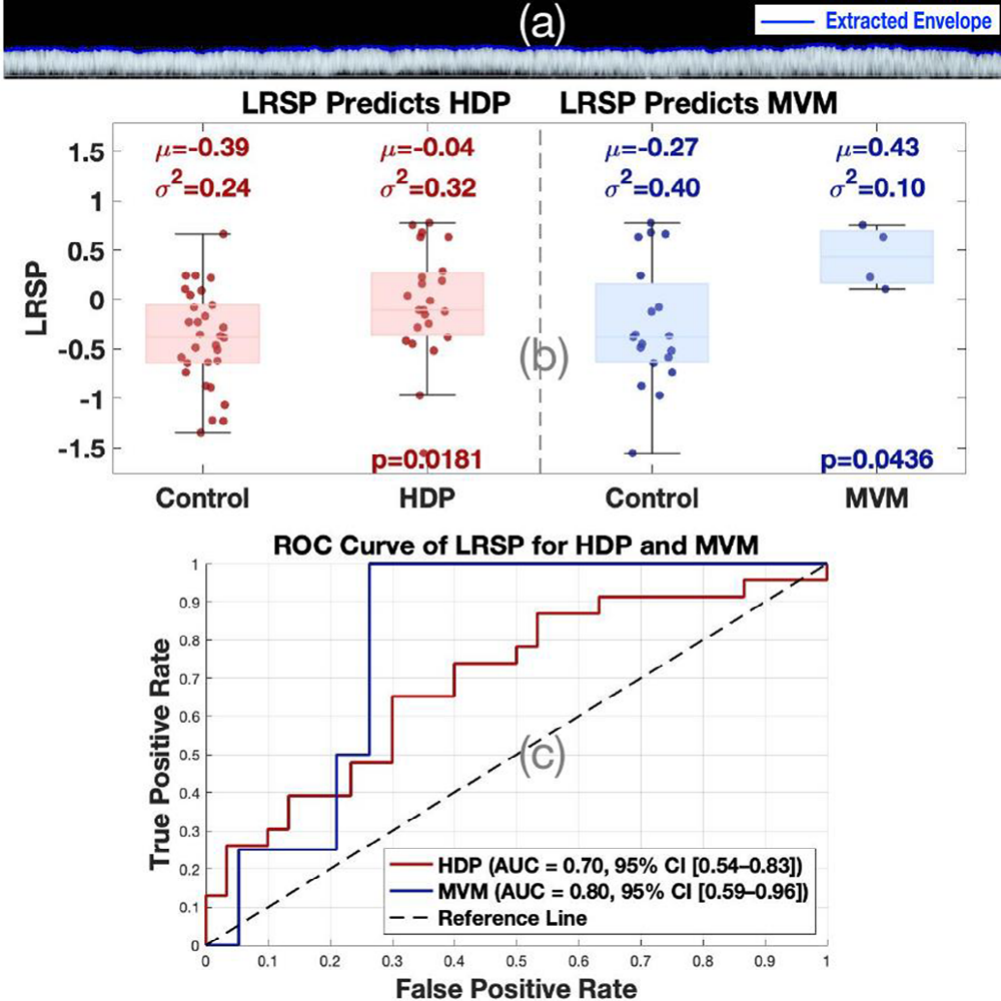

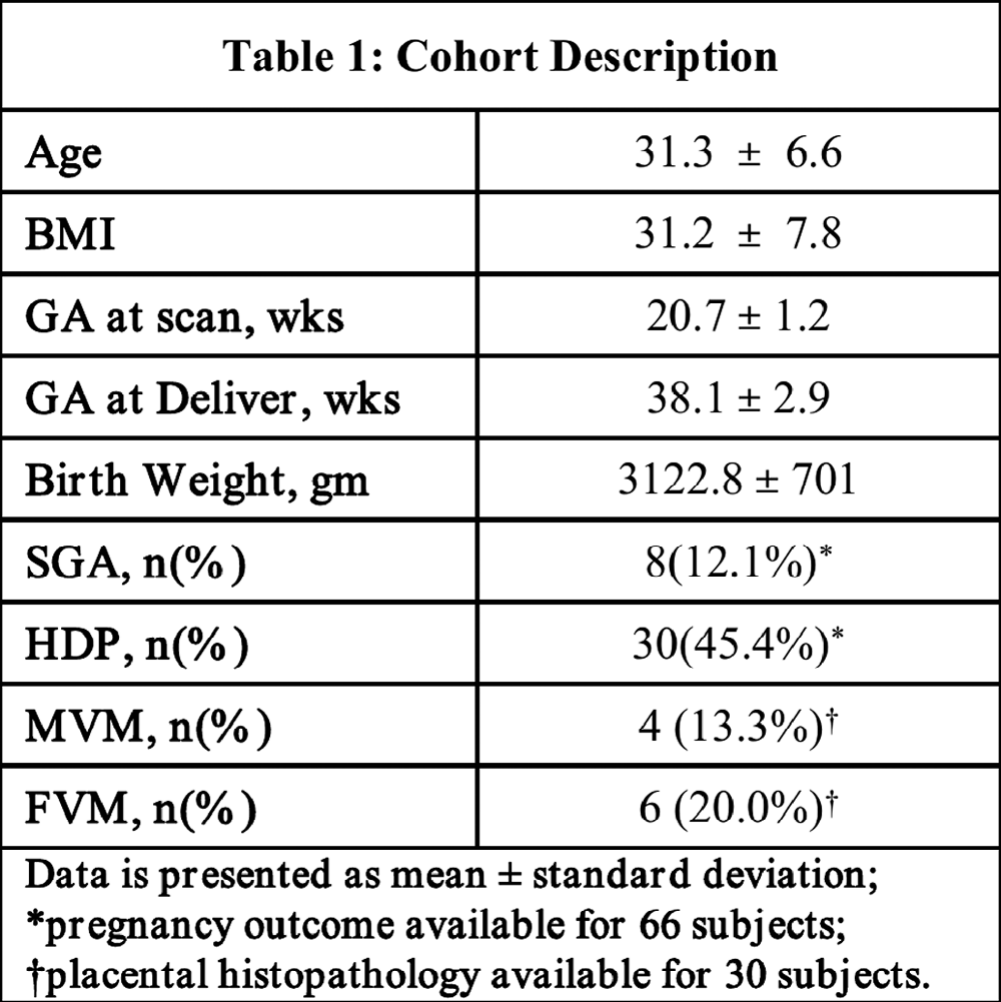

**10:30 AM – 12:00 PM**

## 887 | Moderate**/Severe Insomnia and Risk of Ischemic Placental Disease and Preterm Delivery: A Longitudinal Evaluation**

Naima E. Ross^1^; Steven Friedman^2^; Rashmi N. Aurora^3^; Erinn M. Hade^2^; Justin S. Brandt^4^

^1^NYU Langone, NYU Langone, NY; ^2^Division of Biostatistics, Department of Population Health, NYU Grossman School of Medicine, New York, NY; ^3^Department of Medicine, NYU Grossman School of Medicine, New York, NY; ^4^Division of Maternal‐Fetal Medicine, Department of Obstetrics and Gynecology, NYU Grossman School of Medicine, New York, NY

**Objective**: To assess whether persistent moderate/severe insomnia during pregnancy or its development in the third trimester is associated with ischemic placental disease (IPD)—including preeclampsia (PEC), abruption, and fetal growth restriction (FGR)—and with preterm delivery (PTD).

**Study Design**: We conducted a secondary analysis of the Nulliparous Pregnancy Outcomes Study: Monitoring New Mothers‐to‐be, a prospective cohort of nulliparous singletons. We included those who underwent insomnia screening with the Women's Health Initiative Insomnia Rating Scale (WHIRS) in the first and second/third trimesters (visit 1: 6–14 weeks; visit 3: 22–30 weeks). We compared those with persistent insomnia, defined as moderate/severe insomnia (WHIRS scores of 15–28) at both visits 1 and 3 and those with incident insomnia, defined as none/mild insomnia at visit 1 and moderate/severe insomnia at visit 3. The primary outcome was IPD, defined as any PEC, abruption, or FGR. Secondary outcomes included individual IPD components and PTD < 37 weeks. Log‐binomial regression models estimated the relative risk between groups and corresponding 95% confidence intervals (CI).

**Results**: Among 7, 273 individuals with WHIRS measured at both study visits, 1596 (22.0%) had persistent moderate/severe insomnia and 1240 (17%) had incident moderate/severe insomnia. Those with persistent moderate/severe insomnia had more gestational diabetes (6.5%) and pre‐pregnancy depression (17.9%) (Table 1). Persistent moderate/severe insomnia was associated with 29% increased risk of PTD < 37 weeks (Table 2). Compared to those with none/mild insomnia at both visits, the adjusted risk of IPD was 1.02 (95% CI 0.83, 1.26) for the persistent moderate/severe insomnia group versus 0.85 (95% CI 0.66, 1.09) for the incident insomnia group.

**Conclusion**: Persistent moderate/severe insomnia was associated with increased risk for PTD, while exposure to neither persistent nor incident insomnia was associated with IPD. These findings provide mechanistic insights that suggest the link between insomnia and pregnancy complications such as PTD are not driven by placental ischemia.

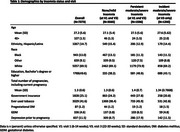

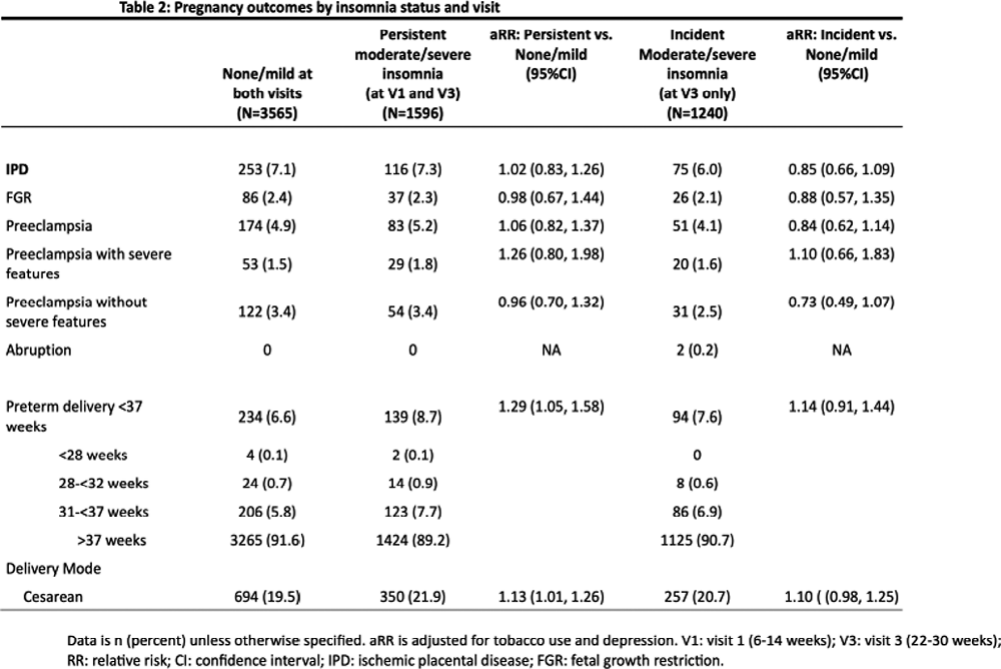

**10:30 AM – 12:00 PM**

## 888 | Antepartum **Low Dose Aspirin Use and De Novo Postpartum Hypertension**

Natalie Cooley^1^; Natalie Creekmur^2^; Sallie Canumay^3^; Christopher Genobaga^4^; Leonard Soloniuk^5^; Bryan Oshiro^6^

^1^School of Medicine, University of California, Riverside, Riverside, CA; ^2^Department of Obstetrics and Gynecology, Loma Linda University School of Medicine, Loma Linda, CA; ^3^Department of Obstetrics and Gynecology, Riverside University Health System, Moreno Valley, CA; ^4^Department of Obstetrics and Gynecology, Riverside University Health System, Loma Linda, CA; ^5^Department of Gynecology and Obstetrics, Riverside University Health System, Moreno Valley, CA; ^6^Department of Obstetrics and Gynecology, Riverside University Health System, Moreno Valley, CA, CA

**Objective**: This study investigates whether antepartum low‐dose aspirin (LDA) use in high‐risk pregnancies reduces the incidence of de novo postpartum hypertension (dn‐PPHTN) between 2 and 42 days postpartum.

**Study Design**: We conducted a large scale retrospective cohort study using TriNetX, a U.S. collaborative network of 71 healthcare organizations and over 126 million patients. Patients aged 18 to 45 with a term uncomplicated delivery and at least one ACOG‐defined high‐risk factor for hypertensive disorders of pregnancy (HDP) were included. Patients with prior diagnoses of HDP or history of immunosuppressant use were excluded. Our study cohort consisted of patients prescribed 81 mg aspirin (ASA) at some instance within 189 days of delivery; the control cohort had no aspirin exposure during that time period. Propensity score matching (PSM) was used to balance age, race, ethnicity, and comorbidities between the cohorts. Outcomes were identified using ICD‐10 codes related to postpartum hypertensive conditions during the 2–42 day postpartum period. Statistical analyses consisted of a 2‐tailed T test and 95% confidence intervals with statistical significance defined as a *p*‐value < 0.05.

**Results**: After PSM, 2, 304 patients remained in each cohort. The incidence of HTN without proteinuria shows a 0.006% lower risk (risk ratio [RR] = 0.46, *p* = 0.028, CI 0.226, 0.934) while pre‐eclampsia shows a 0.008% lower risk (RR = 0.62, *p* = 0.039, CI 0.392, 0.981) in the non‐ASA cohort compared to the ASA cohort. There was no difference between cohorts in the risk ratio of unspecified maternal hypertension (HTN), elevated blood pressure (systolic >140 mmHg or diastolic >90 mmHg), essential HTN, acute kidney disease, eclampsia, and overall postpartum morbidity.

**Conclusion**: These findings may suggest that antepartum LDA offers, at most, limited postpartum vascular protection in high‐risk pregnancies without prior hypertension, as it may not be associated with reduced risk of dn‐PPHTN. This warrants future investigation into the pathophysiology and prevention of dn‐PPHTN beyond 48 hours postpartum.

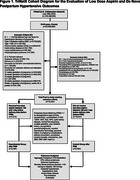

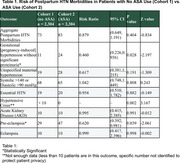

**10:30 AM – 12:00 PM**

## 889 | **Optimal** Timing **of Cervical Length Screening Before 24 Weeks in Twin Pregnancies**

Natalie Kongkham^1^; Arietta Vayenas^1^; Liran Hiersch^2^; Jon F. Barrett^3^; Nir Melamed^4^

^1^Sunnybrook Research Institute, Toronto, ON; ^2^Lis Hospital for Women's Health, Tel Aviv Sourasky Medical Center Gray Faculty of Medicine, Tel Aviv University, Israel, ISRAEL, Tel Aviv; ^3^McMaster University, Hamilton, ON; ^4^Sunnybrook Health Sciences Center, Toronto, ON

**Objective**: Sonographic cervical length (CL) is a valuable screening test for preterm birth (PTB). In twin pregnancies, vaginal progesterone and cervical cerclage may reduce PTB risk in patients with CL≤25mm and ≤15mm, respectively, if identified before 24 weeks. However, the optimal timing for CL measurement remains uncertain. While later measurement increases the overall detection of short cervix, it may delay the diagnosis and timely intervention for those whose cervix shortened earlier. This study aimed to determine the gestational week at which CL measurement provides the optimal balance between overall detection and timely diagnosis of a short cervix.

**Study Design**: A retrospective study of patients with twin pregnancies followed at a single center (2012–2024). Patients underwent routine serial CL measurements every 2–3 weeks from 16–18 weeks until 28–30 weeks. For each week between 16 and 23 weeks, we calculated: (1) the cumulative proportion of patients with CL ≤25 mm and ≤15 mm detected at that week; and (2) the proportion whose diagnosis of a short cervix would have been delayed by ≥2 weeks (who might have missed the opportunity for a timtely intervention). The optimal week was defined as the one achieving the best trade‐off between overall detection and delayed diagnosis.

**Results**: A total of 1, 571 patients underwent 4, 828 CL measurements between 16–24 weeks. The cumulative proportion with CL≤25 mm increased from 1% at 16 weeks to 11% at 24 weeks, while the corresponding increase for CL≤15 mm was from 0% to 6% (Figure 1). CL measurement at 23 weeks offered the optimal balance between overall detection and delayed diagnosis (Figure 2). When considering a strategy of two CL measurements, the best combinations were 19 and 23 weeks for CL≤25mm, and 21 and 23 weeks for CL≤15mm.

**Conclusion**: In twin pregnancies, a single CL measurement at 23 weeks optimizes the detection of a short cervix while minimizing delayed diagnosis. A two‐step approach with an earlier scan at 19 or 21 weeks may further improve the timely diagnosis of a short cervix and the opportunity for intervention.

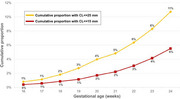

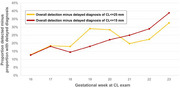

**10:30 AM – 12:00 PM**

## 890 | **Clinical** Risk **Score for Predicting Relaparotomy After Cesarean Delivery: A Multicenter Study**

Natav Hendin^1^; Liron Seidman^2^; Shalhevet Frank^3^; Amit Yavor^4^; Alina Weissmann‐Brenner^5^; Raanan Meyer^6^; Lital Shacham^7^; Liat Mor^8^; Ron Sagiv^8^; Asnat Walfisch^9^; Roy Kessous^10^; Noa Gohar^4^; Eran Hadar^9^; Ohad Houri^11^

^1^Rabin Medical Center, Petah Tikva, HaMerkaz; ^2^Lis Hospital for Women's Health, Tel Aviv Sourasky Medical Center Gray Faculty of Medicine, Tel Aviv University, Israel, Ramat Gan, HaMerkaz; ^3^Rabin medical center, Petah Tikva, HaMerkaz; ^4^Soroka University Medical Center, Beer Sheva, HaDarom; ^5^Sheba Medical Center, Sheba Medical Center, HaMerkaz; ^6^Sheba Medical Center, Ramat Gan, Tel Aviv; ^7^The Chaim Sheba Medical Center, Ramat Gan, HaMerkaz; ^8^Edith Wolfson Medical Center, Holon, HaMerkaz; ^9^Rabin Medical Center, Petach Tikva, HaMerkaz; ^10^Soroka, BEER SHEVA, HaDarom; ^11^BCNatal Fetal Medicine Research Center, BARCELONA, Catalonia

**Objective**: To develop and validate a clinical risk score for predicting relaparotomy within 30 days after cesarean delivery (CD), based on a large multicenter cohort.

**Study Design**: We conducted a retrospective cohort study including 79, 578 women who underwent CD at four tertiary centers between January 2012 and December 2022. Of these, 228 cases of relaparotomy were identified (0.3%). A predictive risk model was derived from a subset of 11, 693 women (228 cases and 11, 465 controls). We developed a clinical risk score by assigning weighted points to variables based on scaled β‐coefficients from a multivariable logistic regression model. Eight variables were selected based on predictive strength and practical applicability: maternal age, BMI at delivery, parity, preterm delivery (< 37 weeks), multifetal gestation, number of prior CDs, emergency CD, and APGAR score at 1 minute. Points were assigned to each variable based on relative risk weights, resulting in a score ranging from 0 to 140. Patients were stratified into three risk groups: low (< 25), medium (25–45), and high (>45).

**Results**: Among the 11, 693 patients included, 44.0% were classified as low risk with a relaparotomy rate of 0.84% (43/5, 145), 34.0% as medium risk with a rate of 2.54% (101/3, 980), and 22.0% as high risk with a rate of 3.27% (84/2, 568). Compared to the low‐risk group, medium‐risk patients had 3.02‐fold higher odds of relaparotomy (2.54% vs. 0.84%), and high‐risk patients had 3.89‐fold higher odds (3.27% vs. 0.84%, *p* < 0.001).

Figure 1 summarizes variable weights and maximum contributions. Parity, emergency CD, and preterm delivery showed the strongest individual discrimination (AUC = 0.600, 0.590, and 0.560, respectively).

**Conclusion**: The proposed score demonstrated meaningful clinical stratification, with relaparotomy rates rising progressively across risk categories. Medium and high‐risk groups showed significantly higher odds or reoperation. Given the severity of relaparotomy as a rare but potentially life‐threatening complication, this proposed tool offers valuable insight for early risk identification and proactive management.

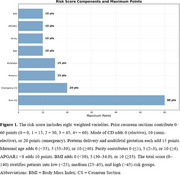

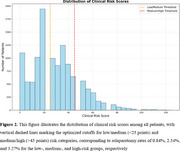

**10:30 AM – 12:00 PM**

## 891 | **Beyond** Biometry**: AI‐Driven Delivery‐Date Prediction Outperforms Hadlock‐derived Estimates in Singletons**

Neil B. Patel^1^; Robert Bunn^2^; Garrett Lam^3^; Brandon Schanbacher^3^; John A. Bauer^3^; John O'Brien^3^

^1^Ascension Sacred Heart Pensacola, Pensacola, FL; ^2^Ultrasound AI, Ultrasound AI, CO; ^3^University of Kentucky, Lexington, KY

**Objective**: Ultrasound‐derived EDD estimation early in the 1st trimester attempts to define an optimal duration of pregnancy; however, additional data to fetal biometry can be utilized to predict pregnancy duration. Our aim was to determine if a Delivery Date AI model can predict actual days‐until‐delivery with non‐inferiority (≤7 days margin) versus the gold‐standard Hadlock method after the 1st trimester and assess superiority.

**Study Design**: Retrospective multi‐site analysis of singleton pregnancies (≥14 weeks) treated at University of Kentucky and 12 satellite clinics from 2017–2021. Individual patient ultrasound studies with ≥3 de‐identified 2‐D grayscale ultrasound images were eligible. AI generated delivery dates; whereas Hadlock GA was obtained from US machine text. Actual date of delivery came from delivery records. Absolute error (AE) was calculated as the difference between actual delivery date and the estimate. Mean AE was the primary endpoint. Median AE and R^2^ were calculated. McNemar and two‐sided paired t‐tests were used.

**Results**: 2350 studies met inclusion criteria, 1061(45.1%) 2^nd^ and 1289(54.9%) 3^rd^ trimester. Delivery Date AI mean AE was 11.1 days (95 % CI 10.7–11.5) vs Hadlock 15.7 days (14.9–16.5); Δ = −4.6 days (95 % CI –5.3 to –3.9); non‐inferiority met; superiority was identified (*p* < 0.0001). R^2^ improved with AI‐incorporation into the estimate: 0.900 vs 0.728. Median AE 8.3 vs 9.0 days was similar; however, data was skewed with AI demonstrating superiority at the upper bounds. Both predictions within 14 days (73.0% vs 66.3%) and within 21 days (88.3% vs 79%) were improved (*p* < 0.0001); but no difference was identified within 7 days (Figure 1). Performance advantage was observed across trimesters; most BMI subgroups; differing ages and ethnicities; and across ultrasound platforms.

**Conclusion**: Relative to the Hadlock standard, Delivery Date AI produced a 4–5 day improvement in predicting date of delivery. Consistent gains across patient subgroups in differing practice locations and across ultrasound platforms supports generalizability of this observation.

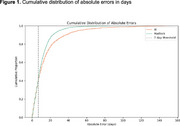

**10:30 AM – 12:00 PM**

## 892 | **Influence of Area Deprivation Index on Diet Quality in Pregnancy**

Ngantu Le^1^; Dong Wang^1^; Megan L. Lawlor^1^; Jeannie C. Kelly^2^; Peinan Zhao^2^; Sarah K. England^1^; Antonina I. Frolova^1^; Nandini Raghuraman^1^

^1^Washington University School of Medicine, St. Louis, MO; ^2^Washington University School of Medicine, Washington University School of Medicine, MO

**Objective**: Area Deprivation Index (ADI) is a measure of socioeconomic disadvantage at the neighborhood level. Pregnant patients living in disadvantaged areas are at higher risk for adverse pregnancy outcomes due to various community‐level factors including limited access to high quality and nutritious food. We studied the relationship between ADI, diet quality, and dietary components in pregnancy.

**Study Design**: This is a secondary analysis of a prospective cohort study of singleton pregnancies who completed the NIH Dietary Health Questionnaire II (DHQ‐II) in the third trimester or within 3 months of delivery. The primary outcome was the Healthy Eating Index (HEI)‐2015 score, a measure of diet quality. Secondary outcomes were dietary components of the HEI score, high HEI 2015 score (>70), and other DHQ‐II components. These outcomes were compared between patients with low ADI < 9 decile (less deprived; < 75^th^ percentile) and high ADI ≥ 9 (more deprived; ≥ 75^th^ percentile). Multivariable linear regression was used to assess the association between ADI and each of the above‐mentioned outcomes.

**Results**: Of the 650 patients included, 445 (68%) had low ADI and 205 (32%) had high ADI. The mean ADI among all participants is 5.8 ± 3.1. Although not statistically significant, patients in higher ADI areas showed lower HEI‐2015 total scores compared to those in lower ADI areas (56.0 ± 8.8 vs 61.1 ± 10.3, *p* = 0.68). High ADI was significantly associated with lower dairy intake (*p* = 0.01) and greater water intake (*p* = 0.03) compared to low ADI. All other components were similar between groups.

**Conclusion**: Overall diet quality did not differ significantly between ADI groups; however, patients in more deprived neighborhoods had significantly lower dairy and higher water intake. Future work can investigate the link between dairy and water intake and adverse pregnancy outcomes.

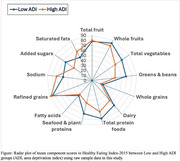

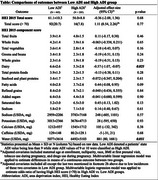

**10:30 AM – 12:00 PM**

## 893 | Predicting **Persistent Postpartum Hypertension after Hypertensive Disorders of Pregnancy: A Meta‐Analysis of Prevalence and Predictors**

Nguyen Thi Huyen Anh^1^; Nguyen Dinh Ky^2^; Nguyen Quynh Anh^2^; Tran Ngoc Tam Phuc^3^; Pham Ngoc Ha^3^; Nguyen Dang Linh Chi^3^; Tran Trung Kien^3^; Nguyen Han Phong^3^; Truong Thanh Huong^4^; Nguyen Manh Thang^5^

^1^National Hospital of Obstetrics and Gynecology, Cua Nam, Ha Noi; ^2^Hanoi Medical University, Kim Lien, Ha Noi; ^3^Hanoi Medical University, Hanoi, Ha Noi; ^4^Phenikaa University, Kim Lien, Ha Noi; ^5^National Hospital of Obstetrics and Gynecology, Hoan Kiem, Ha Noi

**Objective**: While hypertensive disorders of pregnancy (HDP) are known to increase long‐term cardiovascular risk, predicting which women will develop persistent postpartum hypertension (PPPH) remains a clinical challenge. This study aimed to determine the pooled prevalence of PPPH after HDP and to evaluate key maternal predictors.

**Study Design**: A systematic review and meta‐analysis was conducted by searching Embase, PubMed, Scopus, and grey literature in July 2025. PPPH was defined as the persistence of hypertension (blood pressure ≥ 140/90 mmHg) for at least 6 weeks after delivery in women previously diagnosed with a HDP, excluding those with pre‐existing chronic hypertension and cases due to medication withdrawal. Random‐effects models were used to pool the prevalence overall, as well as for early (6 weeks to 3 months) and late (4 to 12 months) follow‐up periods. Standardized mean differences (SMD) were synthesized for continuous predictors.

**Results**: Ten studies involving 5750 participants were included. The overall pooled prevalence of PPPH was 12.5% (95% CI, 11.7–13.4%; I^2^ = 97.3%). Prevalence peaked at 24.4% (95% CI, 21.5–27.2%; I^2^ = 92.7%) during the initial 6 weeks to 3 months postpartum, declining to 11.4% (95% CI, 10.6–12.3%; I^2^ = 98.1%) by 4 to 12 months postpartum. In predictor analysis, women with PPPH were significantly older (SMD 0.34; 95% CI 0.06 – 0.62; I^2^ = 91.2%) and had a higher pre‐pregnancy BMI (SMD 0.57; 95% CI 0.35 – 0.80; I^2^ = 83.8%). Gestational age at delivery did not differ significantly (*p* = 0.15).

**Conclusion**: The finding that nearly 1 in 4 women with prior HDP remain hypertensive at three months postpartum challenges the adequacy of the traditional 6‐week visit and confirms the high‐risk period extends well beyond the conventional puerperium. Advanced maternal age and elevated pre‐pregnancy BMI are powerful predictors that must guide the development of risk‐stratified, extended surveillance and cardiometabolic counseling.

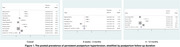

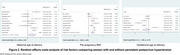

**10:30 AM – 12:00 PM**

## 894 | Associations **of a Composite Social Vulnerability Index with Adverse Pregnancy Outcomes**

Nicola F. Tavella^1^; Sara E. Edwards^2^; Barbara Pereira Vera^3^; Raina Kishan^4^; Nina G. Faynshtayn^3^; Calvin E. Lambert, Jr.^2^; Kimberly Glazer^3^

^1^Division of Maternal‐Fetal Medicine, Icahn School of Medicine, Icahn School of Medicine at Mount Sinai Hospital, New York, NY; ^2^Division of Maternal‐Fetal Medicine, Icahn School of Medicine at Mount Sinai, New York, NY; ^3^Icahn School of Medicine at Mount Sinai, New York, NY; ^4^Mount Sinai Hospital, New York, NY

**Objective**: Exposure to socioenvironmental stress worsens the risk of adverse pregnancy outcomes. Indices measuring social vulnerability cannot individually capture the complex dynamics of the social environment. This study examined whether a composite social vulnerability index (CSVI) was independently associated with adverse pregnancy outcomes.

**Study Design**: This was a retrospective study of all pregnant patients giving birth at a large urban medical center between January 2013—December 2022. We excluded patients outside New York State. Individual‐level data were abstracted from electronic medical records. Addresses were geocoded to 2020 US Census tracts and merged with three measures: Social Vulnerability Index (SVI), Index of Concentrations at the Extremes (ICE), and Childhood Opportunity Index (COI). Primary Component Analysis synthesized a composite index to maximize validity and minimize collinearity. Multivariable multilevel and Firth's regression models tested associations of CSVI with adverse pregnancy outcomes, adjusting for individual‐ and area‐level covariates.

**Results**: This study included 44, 697 pregnant patients. CSVI values ranged from ‐2.75 (least vulnerable) to 3.79 (most vulnerable). Three quarters (33, 524) of patients lived in areas in the lowest three quartiles of CSVI; one quarter (11, 173) of patients lived in areas in highest quartile. Patients in areas of greater CSV were disproportionately more likely to be younger, multiparous, non‐White, and have public insurance and obesity (Table 1). These patients had greater rates of preterm birth (PTB) (*p* = 0.01), as well as hypertensive disorders of pregnancy (HDP) and small‐for‐gestational‐age (SGA) neonates (*p* < 0.001; Table 1). Models estimated that patients in areas with the highest CSVI had greater risk of PTB (1.23 [1.06, 1.43]), HDP (1.18 [1.01, 1.37]), and neonates classified as SGA (1.45 [1.21, 1.74]) or LGA (1.95 [1.22, 3.16]).

**Conclusion**: A tract‐level CSVI was associated with adverse pregnancy outcomes. This suggests value in further refinement of sociostructural indices.

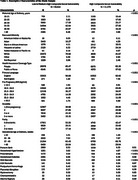

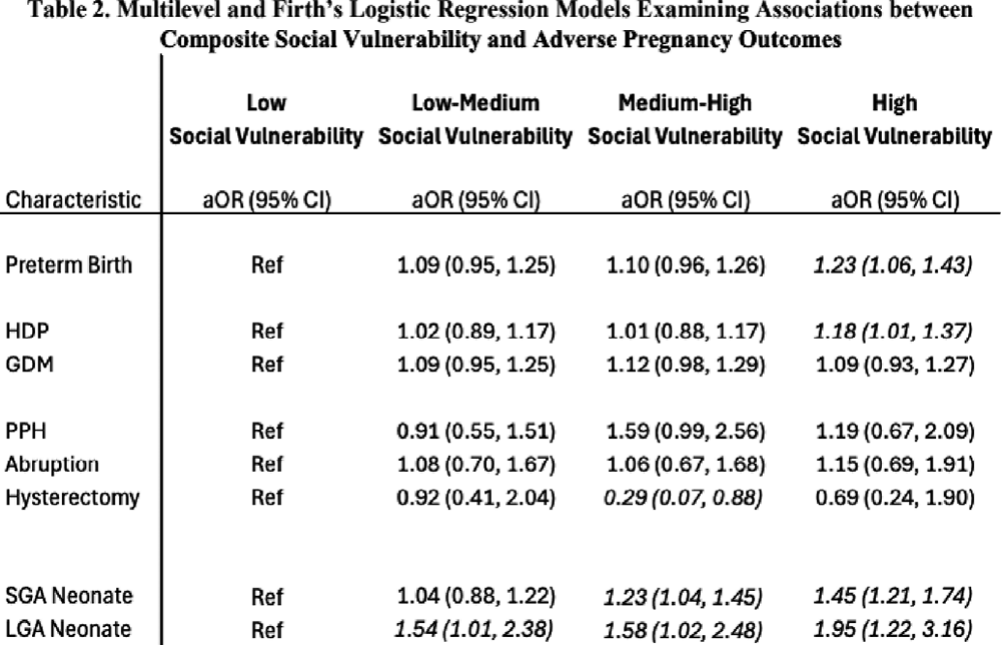

**10:30 AM – 12:00 PM**

## 895 | Obstetricians' **and Midwives' Attitudes and Perceived Barriers to the Implementation of Immediate Postpartum Diabetes Testing**

Veronica Maria Pimentel^1^; Nicole Barreto^2^; Charissa Okang^3^; Dorothy B. Wakefield^3^; Rebecca Crowell^4^

^1^Trinity Health Of New England / Hartford, Trinity Health Of New England / Hartford, CT; ^2^Bridgeport Hospital, Hamden, CT; ^3^Trinity Health Of New England, Trinity Health Of New England / Hartford, CT; ^4^Trinity Health Of New Engalnd, Trinity Health Of New England, CT

**Objective**: The American College of Obstetricians and Gynecologists (ACOG) endorses immediate postpartum diabetes (IPD) testing, performed on day 1 or 2. Compared to traditional testing (4–12 weeks postpartum), IPD has a similar diagnostic value and a higher testing completion rate. Yet, most institutions have not implemented IPD testing. Our study aimed to assess obstetricians' and midwives' attitudes, perceived barriers, and support needed to implement IPD testing.

**Study Design**: We conducted a cross‐sectional online survey of obstetricians (OBs) and midwives practicing in the United States. We evaluated familiarity with the test, willingness to test, barriers to IPD testing, and ways to overcome barriers. Descriptive statistics were performed.

**Results**: We surveyed 213 OBs and midwives from September 2024 to July 2025. Of 180 OBs, 46 (25.6%) were MFMs. Half of all respondents worked in academic centers (49.3%), and 33.9% in community hospitals. Overall, 131 (61.5%) were unfamiliar/slightly familiar with IPD testing, and 82 (38.5%) were moderately/extremely familiar. Only 49 (23.0%) knew that ACOG had endorsed IPD, 34 (15.9%) reported that their institution had IPD guidelines, and 10 (4.7%) participants typically performed IPD. Yet, 174 (81.7%) were willing to perform IPD testing, and 148 (69.5%) believed IPD can improve postpartum care. Participants identified perceived barriers to IPD testing and the support needed to overcome barriers. The top three barriers were lack of familiarity with the test (70.0%), expectations that patients would not fast before testing (46.5%), and perceived lack of ACOG endorsement of IPD (40.4%). The top three desired supports were ACOG endorsement (76.1%), hospital protocol (67.6%), and hospital order set (57.3%).

**Conclusion**: Most OBs and midwives were willing to perform immediate postpartum diabetes testing. Familiarity with the test, knowledge of ACOG's endorsement, and institutional protocol and order sets could help with test implementation.

**10:30 AM – 12:00 PM**

## 896 | **Effects of Healthy Dietary Interventions on Maternal Biomarkers: A Pilot Randomized Clinical Trial**

Julio Mateus Nino; Nicole Elhelou; Anna Buhle; Silvia Triana; Leah Carnick Ledford; Sequoia Finney; Tasha L. Gill

Wake Forest School of Medicine, Charlotte Campus, Charlotte, NC

**Objective**: During pregnancy, physiological increases in lipids, C‐reactive protein (CRP), and hemoglobin A1c (HbA1c) are more pronounced in complications like gestational diabetes (GDM) and preeclampsia. We examined how a healthy Western‐style diet (HWS‐Diet) compares to a Mediterranean‐style diet (Med‐Diet) in influencing these biomarkers in overweight and obese pregnant women.

**Study Design**: Pilot randomized clinical trial of overweight (body mass index [BMI]25.0–29.9 kg/m2) and obese (BMI ≥30.0 kg/m2) pregnant patients at ≤16 weeks of gestation, randomly assigned 1:1 to either an HWS‐Diet or Med‐Diet. Participants received comprehensive dietary counseling and pre‐selected meals and snacks delivered to their homes. Maternal blood levels of CRP, lipids (total cholesterol, LDL, HDL, and triglycerides), and HbA1c were measured at ≤16 weeks and 26–30 weeks, and compared between dietary groups using independent sample t‐tests and Mann‐Whitney U tests.

**Results**: Of 28 pregnant patients, 15 were assigned to the HWS‐Diet and 13 to the Med‐Diet. No significant differences in biomarkers were observed between the groups at any testing point (Table 1). Within each group, CRP and Hb1Ac trended toward a decrease, while total cholesterol, HDL, and LDL trended toward an increase, but these changes were not statistically significant (Table 2). Conversely, triglyceride levels increased significantly in both groups (HWS‐Diet:168.60 ± 12.50 to 217.66 ± 14.21 (*p* = 0.01); Med‐Diet: 159.23 ± 23.50 to 261.23 ± 36.27 (*p* = 0.004)), with a smaller increase in the HWS‐Diet group (29.1%) compared to the Med‐Diet group (64.0%). The rates of GDM (13.33% vs. 23.08%; *p* = 0.64) and preeclampsia (6.67% vs. 7.69%; *p* = 1.0) were not significantly different between groups.

**Conclusion**: In this pilot study, findings suggest that starting a healthy dietary intervention early in pregnancy may help prevent increases in CRP and HbA1c levels, while also lowering the rise in lipid levels, with the HWS‐diet being favored over Med‐Diet. The impact of these changes on clinical outcomes should be evaluated in a larger trial.

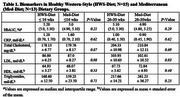

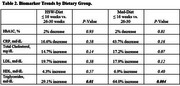

**10:30 AM – 12:00 PM**

## 897 | **Uterine** Synechiae **and the Risk of Adverse Pregnancy Outcomes: A Systematic Review and Meta‐Analysis**

Tomi Kanninen^1^; Nicole Feigenblum^2^; Abdulla Al‐Khan^3^; Manuel Alvarez^3^; Jesus Alvarez‐Perez^3^

^1^Hackensack University Medical Center, Hackensack, NJ; ^2^Hackensack University Medical Center, Englewood, NJ; ^3^Hackensack University Medical Center, Hackensack University Medical Center, NJ

**Objective**: Uterine synechiae are frequently found during pregnancy. Evidence in regard to adverse Outcomes in pregnancy associated with this finding is conflicting. The aim of our study Was to systematically review the currently available evidence on the rate of adverse Outcomes in pregnancy with uterine synechiae.

**Study Design**: PubMed, ScienceDirect, Cochrane Library, and CINAHL were reviewed from their Inception to July 2025. We included all studies comparing adverse outcomes in Pregnancy in patients with and without a uterine synechiae. Bias was evaluated using The Newcastle‐Ottawa Scale.

**Results**: We identified 4 studies with 628 in the uterine synechiae group, and 65, 959 in the Control. In patients with uterine synechiae, the risk of preterm delivery at less than 37 Weeks (OR 1.80, 95%, CI 1.35, 2.39), preterm premature rupture of membranes (OR 3.58, 95%, CI 1.13, 11.36), placental abruption (OR 3.34, 95%, CI 1.57, 7.13), lower Birth weight (MD ‐154.29, 95%, CI ‐259.20, ‐49.38), and presence of placenta previa (OR 3.19, 95%, CI 1.07, 9.57) were significantly increased as compared to the control Group. The risk of preterm birth at less than 34 weeks (OR 1.88, 95%, CI 0.92, 3.85), Cesarean section (OR 1.06, 95%, CI 0.51, 2.21), fetal growth restriction (OR 1.14, 95%, CI 0.54, 2.44), stillbirth (OR 1.37, 95%, CI 0.50, 3.76), and NICU admission (OR 10.98, 95%, CI 0.29, 414.01) and were not significantly different in patients with uterine Synechiae as compared to the control group. Sub‐group analysis for cesarean section For fetal malpresentation was significantly higher in patients with uterine synechiae as Compared to those without (OR 1.97, 95%, CI 1.23, 3.13).

**Conclusion**: The presence of uterine synechiae during pregnancy was associated with a significantly Increased risk of preterm birth at less than 37 weeks, preterm premature rupture of Membranes, placental abruption, decreased birth weight, presence of placenta previa, Gestational age of delivery and cesarean section for fetal malpresentation. These data Support an increased risk of adverse events in pregnancy in the setting of uterine Synechiae.

**10:30 AM – 12:00 PM**

## 898 | Comparative **Patient Outcomes Based on Utilization of Coordinated Care Network for Maternal Opioid Use Disorder**

Nidhi Bhide^1^; Chinyere N. Reid^2^; Meera Ratani^3^; Tanner G. Wright^4^; Emily Coughlin^5^; Jennifer Marshall^2^; Anthony M. Kendle^6^

^1^University of South Florida, Morsani College of Medicine, Tampa, FL; ^2^University of South Florida College of Public Health, Tampa, FL; ^3^Department of Pediatrics, University of South Florida, Tampa, FL; ^4^Department of Pediatrics, The University of South Florida, Tampa, FL; ^5^University of South Florida Morsani College of Medicine, Tampa, FL; ^6^Department of Obstetrics and Gynecology, University of South Florida, Tampa, FL **Objective**: Opioid use disorder (OUD) during pregnancy is a leading cause of morbidity and mortality. The CADENCE (Continuous and Data‐Driven Care) Program integrates perinatal, pediatric, behavioral health, and addiction medicine for patients with OUD. We hypothesized that utilization of the CADENCE program would result in higher rates of medication for OUD (MOUD) use at time of delivery.

**Study Design**: We conducted a retrospective cohort study of pregnancies with OUD from 09/01/2023–01/31/2025 at a single tertiary care center. Electronic medical records were reviewed for demographic and clinical data. The primary exposure was participation in the CADENCE Program based on patient attendance at more than one of the coordinated clinics prior to date of delivery. The primary outcome was MOUD utilization at the time of delivery. Secondary outcomes included completion of prenatal and postpartum visits, hepatitis C screening, obstetric complications, and postpartum depression screening. Data were compared using chi‐square/Fisher's exact test for categorical variables and Mann‐Whitney U test for nonparametric variables. A *p* < 0.05 was considered significant.

**Results**: A total of 135 dyads met inclusion criteria. Sixty‐six (48.9%) participated in the CADENCE Program. Patients in the CADENCE group were more likely to have MOUD enrollment at delivery compared to the non‐CADENCE group (71.8% vs. 28.2%, *p* < 0.001). Of the 71 patients who had MOUD enrollment at delivery, 33 (46.5%) used methadone, 21(29.6%) used buprenorphine, and 17 (23.9%) used buprenorphine‐naloxone. Distribution of MOUD was similar between groups. Patients in the CADENCE group were also significantly more likely to be screened for Hepatitis C (83% v 64%, *p* = 0.010), attend a postpartum visit (64% v 45%, *p* = 0.029), and have any prenatal care (86% v 67%, *p* = 0.007). There was no statistically significant difference in obstetric complications at delivery or postpartum depression scores between groups.

**Conclusion**: Care coordination and evidence‐based treatment through the CADENCE program increases gold‐standard treatment with MOUD in pregnant individuals.

**10:30 AM – 12:00 PM**

## 899 | **Impact of Prenatal Surgical‐to‐Delivery Interval on Early Urologic Outcomes after Fetal Myelomeningocele Repair**

Nikan Zargarzadeh^1^; Claudio V. Schenone^2^; Eyal Krispin^2^; Alireza A. Shamshirsaz^2^; Ashish Premkumar^3^

^1^Boston Children's Hospital, Boston, MA; ^2^Boston Children's Hospital, Harvard Medical School, Boston, MA; ^3^University of Chicago, Chicago, IL

**Objective**: To evaluate the association between the interval from prenatal fetal myelomeningocele (fMMC) repair to delivery and early childhood urologic outcomes in the Management of Myelomeningocele Study (MOMS) cohort.

**Study Design**: This was an unplanned secondary analysis of the prenatal fMMC repair arm of the MOMS using de‐identified data provided by the National Institutes of Health (NIH). The primary exposure was surgery‐to‐delivery interval, dichotomized as ≤58.2 days (lowest quartile) vs. >58.2–110 days. Primary outcomes included urologic surgery, clean intermittent catheterization (CIC), vesicoureteral reflux (VUR), and hydronephrosis by 30 months. Bivariate analyses used Fisher's exact and Wilcoxon tests. Linear regression assessed associations between interval and urologic outcomes, adjusting for covariates significant at *p* < 0.05.

**Results**: 90 participants were eligible for inclusion, 23 (25.5%) in the shortest quartile and 67 (74.5%) in the longer quartiles. Preoperative ventricular size was larger in the shorter surgery‐to‐delivery interval (14.1 v. 10.8 mm, *p* < 0.001). Rates of urologic surgery, CIC use at 30 months, and CIC use at the latest follow‐up were similar between groups. However, vesicoureteral reflux development or worsening demonstrated a trend toward increased risk in the shortest interval group (20% vs. 2.6%, *p* = 0.06). Hydronephrosis at 12 months was notably more frequent in the shortest interval group (relative risk: 0.38, 95% CI: 0.15–0.98), approaching statistical significance (*p* = 0.10). Hydronephrosis at 12 months was significantly associated with shorter prenatal surgical‐to‐delivery interval in adjusted analyses accounting for collinearity between the surgery‐to‐delivery interval and gestational age at delivery through residual analysis and preoperative ventricular size (adjusted OR: 0.94 [95% CI: 0.89, 0.99], *p* = 0.04).

**Conclusion**: A shorter interval between prenatal fMMC repair and delivery is associated with an increased risk of hydronephrosis in early childhood. This finding suggests that prolonging pregnancy post‐surgery may independently improve early urologic outcomes.

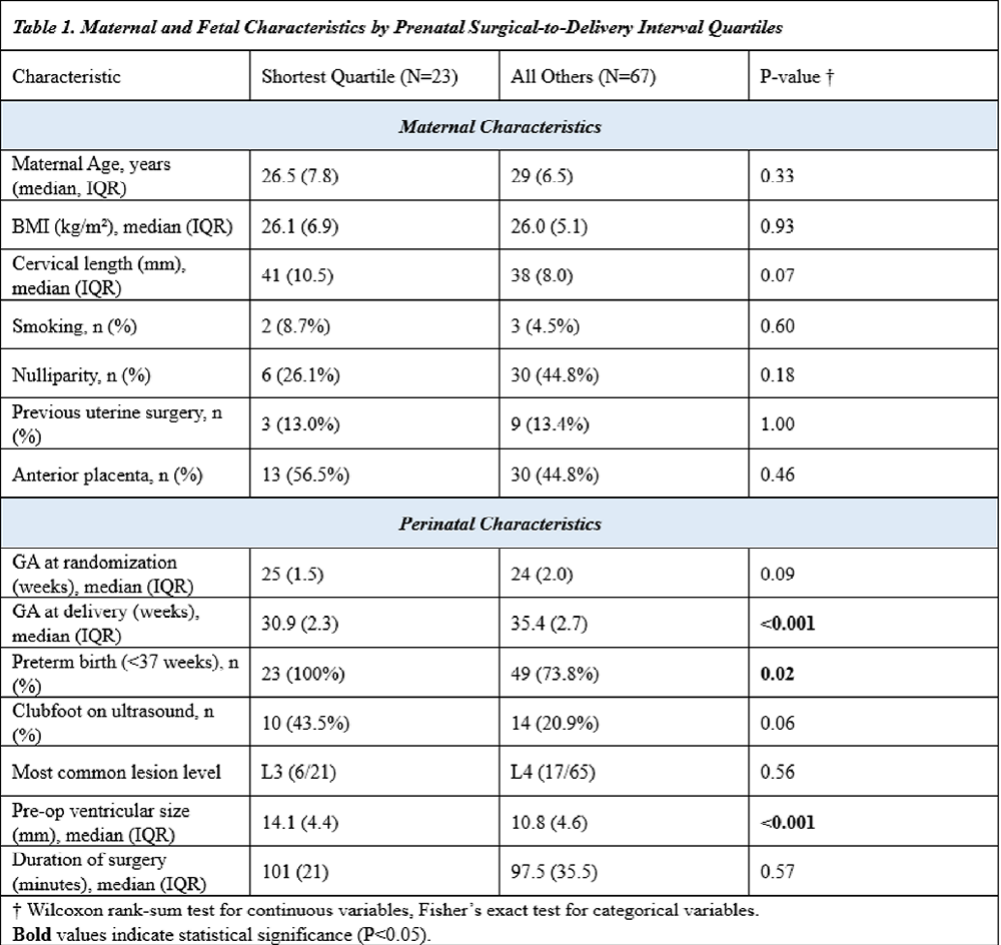

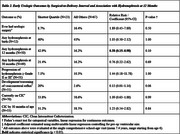

**10:30 AM – 12:00 PM**

## 900 | Preliminary **Proteomics Analysis of Placental Immunological Profiles in Two Congenital Diaphragmatic Hernia Rat Models**

Nilhan Torlak^1^; Fernando Ferrer‐Marquez^2^; Josephine Kemp^2^; Marc Oria^2^; Marco Klein^3^; Ronny Schmidt^3^; Christoph Schroeder^3^; Braxton Forde^4^; Emrah Aydin^2^; Jose L. Peiro^1^

^1^New York University Langone Health Fetal Research Lab, New York, NY; ^2^Center for Fetal and Placental Research, Cincinnati Children's Hospital Medical Center, Cincinnati, OH; ^3^Sciomics, Heidelberg, Baden‐Wurttemberg; ^4^Division of Maternal Fetal Medicine, Department of Obstetrics and Gynecology, College of Medicine, University of Cincinnati, Cincinnati, OH

**Objective**: Congenital diaphragmatic hernia (CDH) leads to pulmonary hypoplasia, hypertension, and abnormal placental pathology and development. The drivers of said placental abnormalities remain entirely unknown. Given the importance of inflammation and maternal‐feto tolerance in proper placental development, thus sought to characterize placental immune profiles in nitrofen‐ and surgically‐induced CDH rat models.

**Study Design**: Pregnant Sprague Dawley rats were either treated with nitrofen (via gavage feeding) at embryonic day 9.5 (E9.5) or underwent surgical creation of CDH at E18.5. Placentas were harvested at E21.5. Fetuses were assigned to the groups according to CDH presence in both models. Rat fetuses that were not exposed to nitrofen or surgery were used as controls (CNT). Histological evaluation was performed on the lungs and placentas. Additionally, placentas were used for proteomics analysis.

**Results**: Macroscopic inspection of the diaphragm and lung histology confirmed CDH presence and lung hypoplasia. Preliminary proteomics analysis revealed 21 immune system‐related proteins as differentially abundant in nitrofen groups and 30 in surgical groups. Four proteins were differentially abundant in both nitrofen and surgical groups relative to controls (Table 1). ARHG2 and CCN2 were elevated in nitrofen groups, and decreased in the surgical groups. CCL4 was decreased in the nitrofen groups, and it was elevated in the surgical groups. In the nitrofen groups, IL1A was increased, while in the surgical groups, it was decreased in abundance.

**Conclusion**: This study is the first to identify placental cytokine profiles in nitrofen and surgical CDH rat models. The findings showed distinct immunological responses according to the method of CDH induction, and highlighted key immunological factors linking placental disruptions in CDH, which had not been previously described. Future research is needed to investigate these differences to establish a deeper association between placental disruptions and CDH.

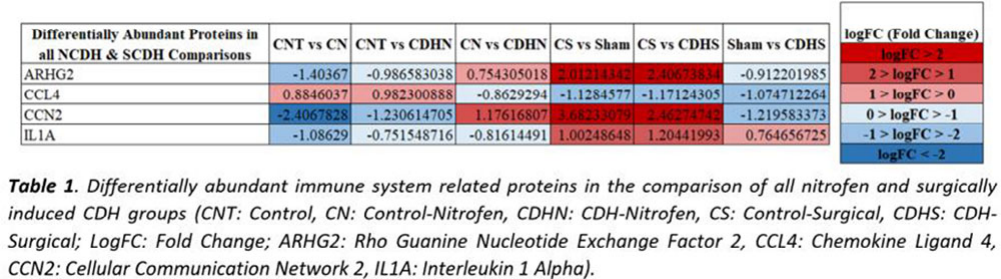

**10:30 AM – 12:00 PM**

## 901 | **Maternal Adverse Outcome in Placental Abruption: Impact of Preterm vs. Term Delivery**

Nirit Maliyanker^1^; Michal Fishel Bartal^2^; Guy Dumanis^3^; Noa Gonen^4^; Avishag Abecassis^5^; Lital Shaham^6^

^1^Sheba medical center, GIVATAYIM, Tel Aviv; ^2^Division of Maternal‐Fetal Medicine, Department of Obstetrics, Gynecology and Reproductive Sciences, Sheba Medical Center, Tel Aviv University, Israel, Ramat Gan, HaMerkaz; ^3^Education Authority, Chaim Sheba Medical Center, Sheba Medical Center, HaMerkaz; ^4^Wolfson Medical Center, Holon, HaMerkaz; ^5^Department of Obstetrics and Gynecology, Sheba Medical Center, Ramat Gan, HaMerkaz; ^6^Department of Obstetrics and Gynecology, Sheba Medical Center, Sheba Medical Center, HaMerkaz

**Objective**: To evaluate maternal morbidity in individuals with placental abruption, comparing outcomes between those delivering preterm (< 37 weeks) and at term (≥37 weeks).

**Study Design**: We conducted a retrospective cohort study of individuals presenting with placental abruption at a tertiary medical center between 2011 and 2025. We excluded those with placenta previa, placenta accreta spectrum, or multifetal gestation. The primary outcome was a composite of severe maternal adverse outcomes including any of the following maternal death, disseminated intravascular coagulation (DIC), intensive care unit (ICU) admission, postpartum hemorrhage, blood transfusion, re‐laparotomy or re‐admission.

**Results**: Of 144, 601 deliveries during the study period 1, 360 (1%) presented with placental abruption, of them 450 (33.1%) delivered preterm. The preterm group had lower BMI) 25.95 vs 27.1 *p* < 0.001), and nulliparity (33.8% vs 42.6%, *p* = 0.002), higher rate of chronic hypertension (2.9% vs. 1.2%, *p* = 0.05), thrombophilia (6% vs. 3%, *p* = 0.011), antiphospholipid syndrome (3.1% vs. 1.2%, *p* = 0.025), past preterm delivery (2.7 vs 0.8, *p* = 0.01), preeclampsia (8% vs. 3%, *p* < 0.001), severe preeclampsia (2.2% vs. 0.2%, *p* < 0.001), premature contraction (18% vs.11% *p* < 0.001) and blood products administration during pregnancy (8.2% vs. 0.7% *p* < 0.001) compared to term abruption. The rate of composite severe maternal morbidity was higher in the preterm group compared to term abruption (10.7 vs 5.4%, *p* = 0.001). Significant differences were found in various components of the composite outcome such as higher rate of ICU admission (1.1% vs. 0.1% *p* = 0.017), higher rate of DIC (2.4% vs.0.2% *p* < 0.001), re‐laparotomy (2.9% vs.0.9%, *p* = 0.009) and re‐admission (2.2% vs. 0.1% *p* < 0.001).

**Conclusion**: The composite outcome was significantly more prevalent in the preterm delivery group compared to term deliveries. Various component of the composite outcome was also observed at higher rates among the preterm group. These findings underscore the importance of strategic planning and preparedness in managing preterm deliveries associated with placental abruption.

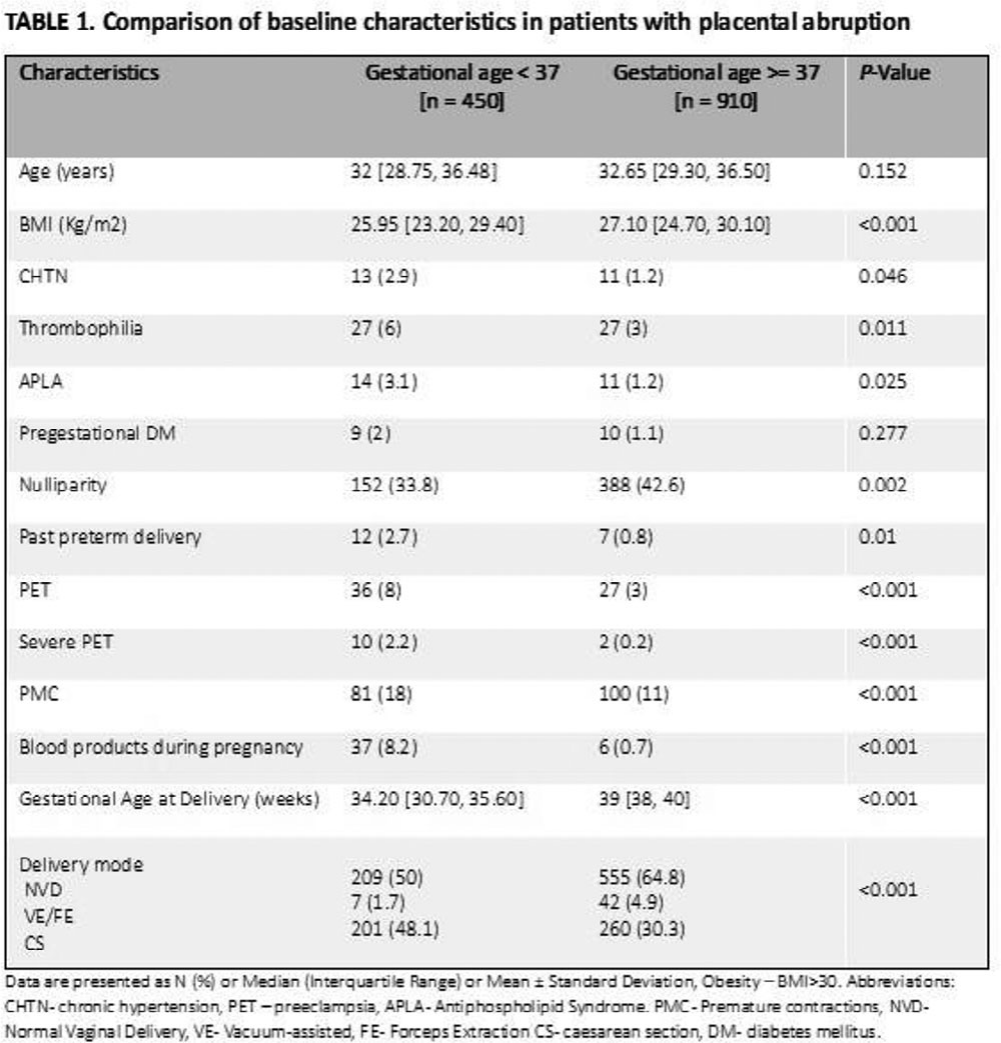

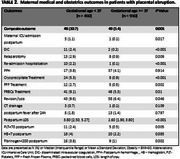

**10:30 AM – 12:00 PM**

## 902 | **The** Relationship **Between Household Income and Maternal Psychosocial Outcomes Following Prenatal Myelomeningocele Repair**

Nkechinyelum Ogu^1^; Abhinav Reddy^2^; Henry David^2^; Bahktiar Yamini^2^; Jessica T. Fry^1^; Aimen Shaaban^3^; Ashish Premkumar^2^

^1^Northwestern University Feinberg School of Medicine, Chicago, IL; ^2^University of Chicago, Chicago, IL; ^3^The Chicago Institute for Fetal Health (CIFH), Chicago, IL

**Objective**: To evaluate the relationship between household income and maternal psychosocial outcomes at 12 and 30 months following delivery in patients who underwent prenatal myelomeningocele (MMC) repair.

**Study Design**: This was an unplanned secondary analysis of individuals undergoing prenatal MMC repair in the Management of Myelomeningocele Study (MOMS) who completed maternal psychosocial surveys by 30 months after delivery. Bivariate analyses were performed comparing families with household income above versus below $50, 000 at the time of randomization, which corresponded to the median U.S. household income during the study period. Outcomes included scores from the Family Resource Scale (FRS), Family Support Scale (FSS), Impact on Family Scale, Parenting Stress Index, and Beck Depression Inventory. Wilcoxon rank‐sum tests were used for continuous variables and Fisher's exact or chi‐squared tests for categorical variables. A *p*‐value < 0.05 indicated statistical significance.

**Results**: A total 83 individuals were assessed at 12 months, and 86 at 30 months. Of these, 64% and 70%, respectively, reported a household income above $50, 000. While significant sociodemographic differences were noted between groups, there was no difference in lesion level, lateral ventricular size, clubfoot, operative duration, and gestational age at delivery (Table 1). Participants with lower household income had significantly lower FRS scores at 12 (133.5 v. 120, *p* = 0.002) and 30 months (133.1 v. 121.6, *p* = 0.02), as well as lower FSS scores at 12 months (49.8 v. 42.8, *p* = 0.009) indicating more challenges. Participants with lower income reported greater personal and financial strain at 12 months; personal strain persisted at 30 months. No statistically significant differences were observed in other outcomes.

**Conclusion**: Lower household income at the time of prenatal intervention is associated with persistent disparities in maternal psychosocial and financial well‐being. These findings highlight the importance of assessing maternal psychosocial well‐being and addressing socioeconomic factors during counseling and post‐operative care.

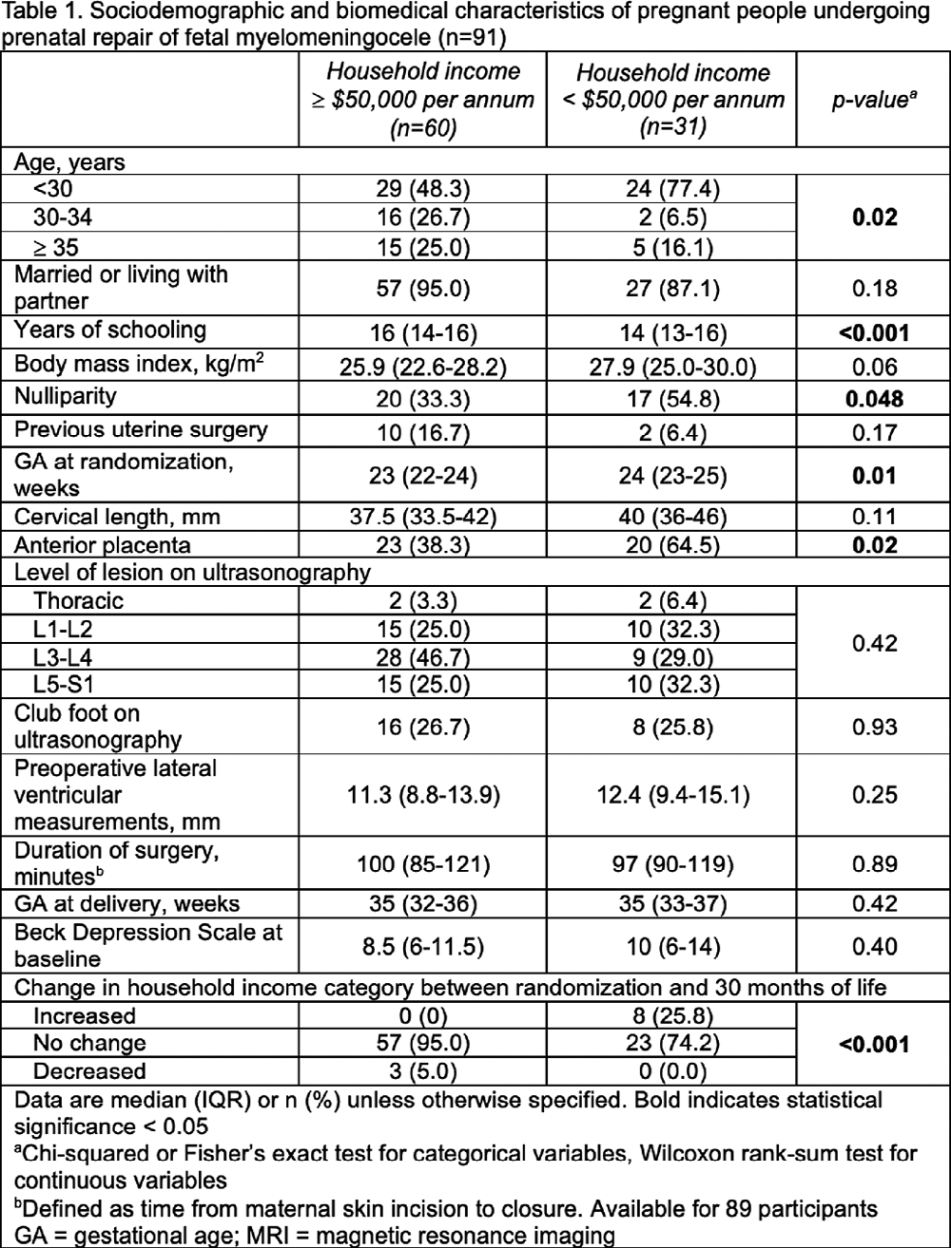

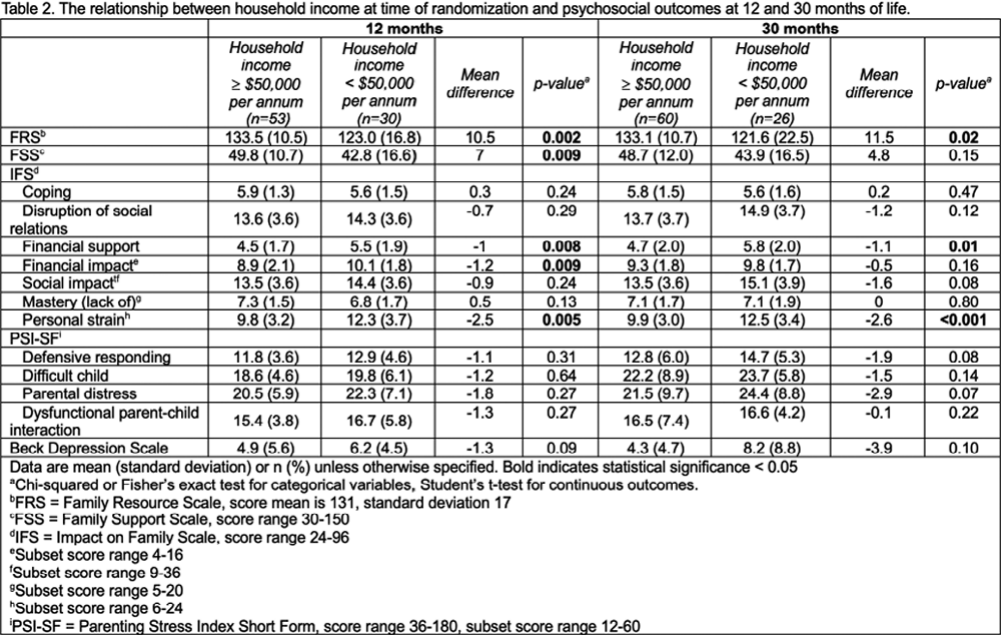

**10:30 AM – 12:00 PM**

## 903 | **Maternal Asthma and Long‐Term Gastrointestinal Morbidity in the Offspring: A Population‐Based Cohort Study**

Noam Shema^1^; Tamar Wainstock^2^; Eyal Sheiner^3^

^1^Department of Obstetrics and Gynecology, Soroka University Medical Center, Ben‐Gurion University, Beer sheva, HaDarom; ^2^Department of Epidemiology, Biostatistics and Community Health Sciences, Faculty of Health Sciences, Ben‐Gurion University of the Negev, Beer Sheva, HaDarom; ^3^Soroka University Medical Center, Faculty of Health Sciences, Ben‐Gurion University of the Negev, beer sheva, HaDarom

**Objective**: Maternal asthma is a prevalent inflammatory condition during pregnancy. Emerging evidence suggests that maternal immune activation may influence fetal gastrointestinal development and function. We aimed to investigate whether maternal asthma is associated with increased long‐term gastrointestinal morbidity in [אשפ1] the offspring.

 [אשפ1]תברר עם שיר או מישהי אם לא אומרים of the offspring

**Study Design**: A population‐based cohort analysis was conducted, comparing the incidence of gastrointestinal‐related pediatric morbidity (obtained from both hospital and community‐based diagnoses) among offspring of mothers with and without asthma. The cohort included all singleton live births between 1991 and 2021 at a tertiary medical center. Infants with congenital malformations, multiple gestations, or perinatal mortality were excluded. A Kaplan‐Meier survival curve compared cumulative incidence of gastrointestinal morbidity. A Cox proportional hazards model was used to control for relevant confounders.

**Results**: The final cohort included 232, 476 births, of which 2, 875 (1.2%) were to mothers with asthma. Offspring of asthmatic mothers had significantly higher rates of gastrointestinal‐related morbidity compared to those without maternal asthma (32.6% vs. 26.4%, OR = 1.3, 95% CI 1.24–1.45; *p* < 0.001; Table). Likewise, Kaplan–Meier analysis demonstrated a significantly increased cumulative incidence of gastrointestinal morbidity in the exposed group (log‐rank *p* < 0.001; Figure). In a Cox regression model, maternal asthma remained an independent risk factor for gastrointestinal morbidity in the offspring (adjusted HR = 1.95%3, CI 1.20–1.30; *p* < 0.001), after adjusting for confounders such as maternal age, gestational age at birth, gestational diabetes, hypertensive disorders, and cesarean delivery.

**Conclusion**: Maternal asthma is independently associated with an increased long‐term gastrointestinal morbidity of the offspring. Maternal inflammatory and immune conditions during pregnancy may have long‐lasting effects on fetal organ development.

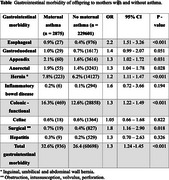

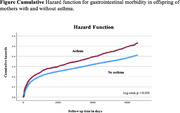

**10:30 AM – 12:00 PM**

## 904 | **Late** Preterm **Steroids and Neonatal Outcomes in Fetal Growth Restriction: A Retrospective Cohort Analysis**

Nofar Bar Noy‐Traub^1^; Hadar‐ Yaacov Omer^1^; Liel Mharian^1^; Batel Yosef^1^; Eynit Grinblatt^1^; Moran Weiss^1^; Dorit Ravid^2^; Tal Biron Shental^3^; Sivan Farladansky Gershnabel^2^

^1^Meir Medical Center, Kfar Saba, HaMerkaz; ^2^Department of Obstetrics and Gynecology, Meir Medical Center, Kfar Saba, HaMerkaz; ^3^Meir Medical Center, Meir Medical Center, HaMerkaz

**Objective**: Antenatal corticosteroids (ACS) before 34 weeks improve neonatal outcomes. Their use has extended into the late preterm period (34.0–36.6 weeks) for pregnancies at risk of early delivery. However, the effect of late preterm ACS in pregnancies complicated by fetal growth restriction (FGR) is unclear. This study evaluated the association between late preterm ACS and early neonatal outcomes in FGR pregnancies.

**Study Design**: A retrospective cohort study included singleton pregnancies with FGR, defined as an estimated fetal weight (EFW) below the 3rd percentile, delivered during nine years. This strict definition focused on significantly growth‐restricted cases. Patients were categorized by ACS exposure: none, <34 weeks, or ≥34 weeks. Neonatal outcomes included NICU admission, hypoglycemia, phototherapy, respiratory support, and a composite adverse outcome (≥1 of the above). Multivariable logistic regression was adjusted for gestational age, birthweight, and maternal/obstetric factors. Major congenital anomalies, viral infections, and genetic syndromes were excluded.

**Results**: Of 3, 492 FGR pregnancies, 3, 241 did not receive ACS, 184 received ACS < 34 weeks, and 67 received ACS ≥34 weeks. The late preterm ACS group delivered earlier (36.2 vs. 37.3 vs. 38.6 weeks, *p* < 0.001) and had lower birthweights (1989g vs. 2159g vs. 2443g, *p* < 0.001) as expected. NICU admission (34.3%), hypoglycemia (20.8%), and phototherapy (20.8%) were significantly higher in the late ACS group (all *p* < 0.001). Respiratory support was similar across groups (*p* = 0.284). Despite higher baseline risk, multivariable analysis showed no association between late preterm ACS and improved outcomes (aOR 0.97, *p* = 0.955).

**Conclusion**: In FGR pregnancies < 3rd percentile, late preterm ACS administration was not associated with improved early neonatal outcomes. The higher complication rates in the late ACS group were attenuated after adjustment. These findings emphasize the need for individualized clinical decision‐making when considering late preterm corticosteroids in FGR cases.

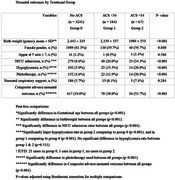

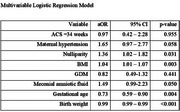

**10:30 AM – 12:00 PM**

## 905 | **Beyond** Gestational **Weight Gain: Food Deserts Linked to Higher Early Pregnancy Weight**

Noor K. Al‐Shibli^1^; Elizabeth C. Rhodes^2^; Erin Ferranti^3^; Anne L. Dunlop^4^; Suchitra Chandrasekaran^5^

^1^Emory University School of Medicine, Emory University School of Medicine, GA; ^2^Rollins School of Public Health, Emory University, Atlanta, GA; ^3^Nell Hodgson Woodruff School of Nursing, Dept of Gynecology & Obstetrics, Emory University, Nell Hodgson Woodruff School of Nursing, Dept of Gynecology & Obstetrics, Emory University/ Atlanta, GA; ^4^Department of Gynecology and Obstetrics, Division of Research, Emory University School of Medicine, Dept of Gynecology & Obstetrics, Division of Research, Emory University School of Medicine/ Atlanta, GA; ^5^Department of Gynecology and Obstetrics, Emory University School of Medicine, Atlanta, GA

**Objective**: Obesity, increased gestational weight gain (GWG), & postpartum weight retention (PPWR) are well‐established risk factors for adverse perinatal outcomes. While poor diet quality contributes to maternal obesity and metabolic dysfunction, the impact of the local food environment remains unexplored. Food deserts (FD), areas with limited access to nutritious/affordable food, exemplify structural drivers of health disparities. Therefore, we evaluated the association between FD residence during pregnancy and maternal body mass index (BMI), GWG & PPWR.

**Study Design**: Using our institutional obstetric database, we performed a retrospective cohort study of all deliveries with documented zip‐code & clinical data. FD status was assigned by zip‐code of primary residence according to the U.S. Department of Agriculture (USDA) definitions. Maternal BMI was documented at 1^st^ prenatal & delivery visits. Maternal weight was documented in 1^st^ trimester (TRI) & postpartum visits. GWG was estimated by calculating change between *(Delivery BMI‐1^st^ TRI BMI)*. PPWR was estimated calculating change between *postpartum weight (PPW)‐initial prenatal weight (IPW)*. Kruskal‐Wallis & Chi‐square tests were performed to compare continuous & categorical variables, respectively.

**Results**: Among *N* = 12, 968 pregnancies, 33% (*n* = 4, 298) lived in a FD. 1^st^ TRI BMI (30.2 ± 7.9 vs 29.5 ± 7.2, *p* < 0.01), IPW (177 ± 49.3 vs 171.3 ± 45.2, *p* < 0.01) & PPW (178.2 ± 48.9 vs 171.1 ± 45.2, *p* < 0.01) were significantly higher among those who lived in a FD vs those who did not. However, GWG estimated by change in BMI (3.02 ± 3.1 vs 3.07 ± 3.2, *p* = 0.66)) & PPWR (2.5 ± 14.9 vs 2.85 ± 14.4, *p* = 0.62) did not significantly differ between groups.

**Conclusion**: While GWG & PPWR did not differ significantly by FD status, those living in FDs began pregnancy at a higher weight and retained a higher weight postpartum. Our data demonstrate residence in a FD is associated with higher maternal weight throughout the perinatal period, which can increase long term cardiometabolic risk. Future studies should investigate underlying sociodemographic factors driving elevated maternal weight in FD populations.

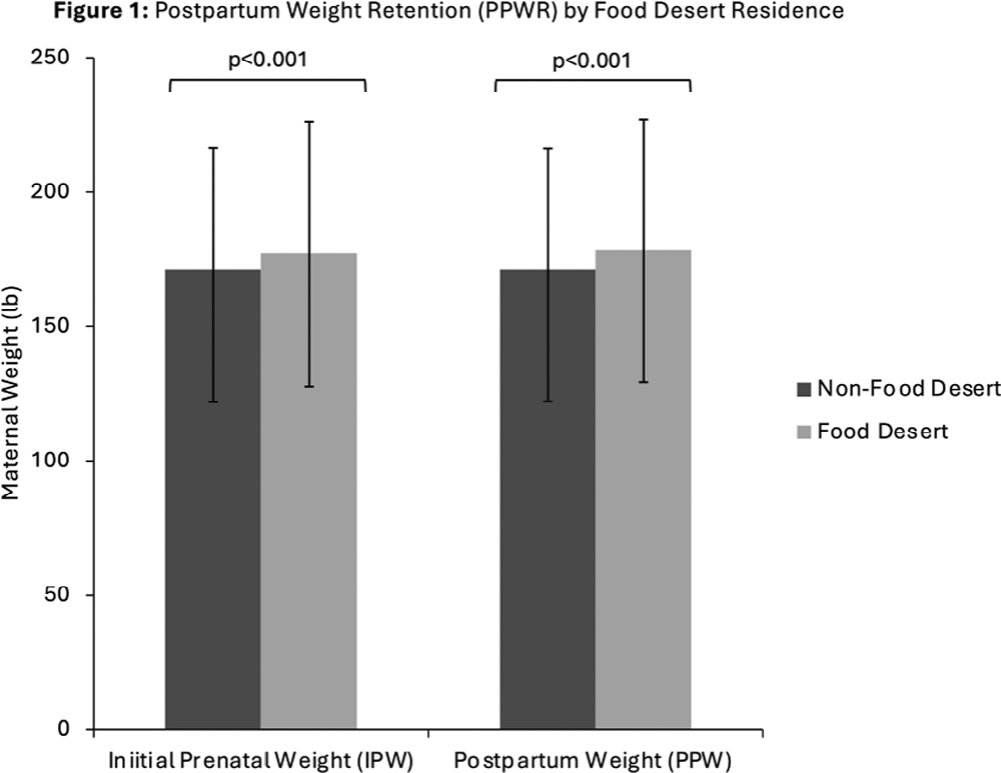

**10:30 AM – 12:00 PM**

## 906 | Qualitative **Analysis of Community Doula Integration into Hospital‐Based Perinatal Care for Black Birthing Patients**

Noria McCarther^1^; Angela Martin^2^; Sharla M. Smith^2^; Joanna Brooks^2^; Oluoma Obi^2^; Sharon A. Fitzgerald Wolff^3^; Annabel Mancillas^2^; Carrie L. Wieneke^4^

^1^University of Kansas Medical Center, Kansas City, MO; ^2^University of Kansas Medical Center, Kansas City, KS; ^3^The University of Kansas, Kansas City, KS; ^4^University of Kansas Medical Center, Fairway, KS

**Objective**: To explore experiences of community‐based doulas supporting Black birthing patients in an academic medical center and to identify barriers and facilitators to effective hospital‐community partnership.

**Study Design**: This qualitative study was embedded within a randomized controlled trial comparing routine versus doula‐enhanced perinatal care. Study participants were doulas from Uzazi Village, a Black‐led community organization. Doulas participated in a structured focus group addressing hospital navigation, collaboration with clinical teams, and perceived impact on patient experience. Discussions were audio‐recorded, transcribed, and coded using a combined deductive–inductive framework. Secondary analysis with patient surveys and perinatal outcome data is ongoing.

**Results**: Doulas reported being welcomed during prenatal visits but often disregarded upon arrival to Labor & Delivery. Barriers included lack of formal introduction to the unit, navigation/parking challenges, no badge or role identification, and limited access to patient resources. Staff knowledge of doula roles varied; collaboration ranged from positive engagement to exclusion from care discussions. Facilitators included respectful acknowledgment from clinicians and advocacy alongside patients. Five themes emerged (Table 1).

**Conclusion**: Community‐based doulas face structural, logistical, and cultural barriers to inpatient integration. Mixed‐methods analysis will further elucidate relational dynamics and identify strategies to foster respectful, bidirectional partnerships between hospitals and community doula organizations, critical to advancing perinatal equity for Black birthing people.

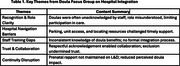

**10:30 AM – 12:00 PM**

## 907 | **Mouse** Model **of Cesarean Section Wound: Model Validation and Basic Characteristics of the Scar**

Olga Grechukhina^1^; Viktoriia Kolesnik^2^; Samantha Novo^2^; Qing Zuo^2^; Colleen Sinnott^1^; Yang Yang‐Hartwich^3^

^1^Division of Maternal Fetal Medicine, Yale School of Medicine, New Haven, CT; ^2^Yale University, New Haven, CT; ^3^Department of Obstetrics, Gynecology, and Reproductive Sciences, Yale School of Medicine, New Haven, CT

**Objective**: Placenta accreta spectrum (PAS) is a highly morbid pregnancy complication that results from abnormal adherence of the placenta to the uterine wall, usually in the area of prior cesarean section or other uterine injury. Molecular mechanisms driving the abnormal placental implantation and trophoblast invasion are still unclear. The aim of this study was to improve and better characterize the mouse model of cesarean section wound and to further analyze the cellular and molecular features of resultant scar tissues.

**Study Design**: On postpartum day 1 C57BL6 mice underwent a unilateral longitudinal full thickness injury (Figure1A). Mice were sacrificed 4–6 weeks later, and uteri (injured and uninjured) were fixed to prepare tissue sections. H&E staining was performed to assess histological architecture. Immunofluorescent (IF) staining for alpha smooth muscle actin and COL1A2 was performed to characterize the myometrial structure and collagen composure of the scar.

**Results**: Gross appearance of the injured uterine horn varies from unremarkable to dilated with some uterine distortion and adhesions (Fig 1B). Injured uteri consistently demonstrated a defect in the circumferential and longitudinal myometrial layers of various width (Figure 1C, D). The scar was consistently covered by endometrial epithelium, but the thickness of underlying stroma varied. IF confirmed consistent incomplete healing of both myometrial layers with areas of disorganized myometrial fibers and abnormal collagen deposition in the scar (Figure2).

**Conclusion**: We characterized a mouse model of uterine injury which recapitulates cesarean section injury in humans. Full thickness longitudinal uterine injury results in permanent disruption of both layers of myometrium and development of the scar, characterized by various amount of type I collage deposition. This model can be used to further understand post‐cesarean section healing process and the role of the scar in PAS formation.

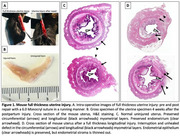

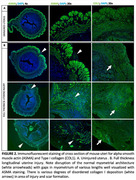

**10:30 AM – 12:00 PM**

## 908 | **Artificial Intelligence as a Prenatal Support Tool: How Does AI Compare to Standard Clinical Guidance?**

Olivet Martinez^1^; T Morgan Moody^1^; April Bleich^2^; Kollier Hinkle^1^

^1^Baylor Scott & White All Saints Medical Center, Fort Worth, TX; ^2^Baylor Scott & White All Saints Medical Center, Baylor Scott & White All Saints Medical Center, TX

**Objective**: Artificial intelligence (AI) has gained popularity in healthcare and conversational AI chatbots have become widely accessible in recent years. Pregnant individuals often seek health information online and this trend is especially prevalent in patients with difficulty accessing health care services. The purpose of this study was to assess the accuracy, readability, and empathy of AI‐generated responses to common pregnancy questions.

**Study Design**: This cross‐sectional study analyzed AI chatbot responses to 10 pregnancy‐related questions. Questions were input into four AI chatbots: ChatGPT, Meta AI, Google Gemini, and Grok. Each response was evaluated across three metrics: accuracy, determined by comparing responses to guidelines from the American College of Obstetricians and Gynecologists; readability, assessed using the Flesch Reading Ease Score; and empathy, measured using a scale adapted from the Empathetic Communication Coding System.

**Results**: Accuracy of chatbot responses was high with all responses assessed as completely accurate (40% of responses) or partially accurate (60% of responses). Accuracy did not differ significantly across chatbots (*p* = 0.337). However, statistically significant differences were observed in readability (*p* = 0.0099) and empathy (*p* = 0.035). ChatGPT had significantly higher readability scores than Meta AI (*p* = 0.028) and Grok (*p* =  .008). ChatGPT also demonstrated significantly higher empathy scores than Google Gemini (*p* = 0.019).

**Conclusion**: Although all chatbots provided similarly accurate information, ChatGPT ranked highest in delivering responses that were more readable and emotionally supportive compared to Google Gemini, Meta AI, and Grok. This highlights the potential of AI to serve as a prenatal support tool, delivering accurate and accessible information to meet the informational and emotional needs of expectant patients during pregnancy.

**10:30 AM – 12:00 PM**

## 909 | **Health** Literacy **and Maternal Mental Health and Psychosocial Support**

Onyinye Ohamadike^1^; Xiaoning Huang^1^; Uma M. Reddy^2^; Robert M. Silver^3^; Rebecca B. McNeil^4^; George R. Saade^5^; Hyagriv Simhan^6^; William A. Grobman^7^; Lynn M. Yee^8^

^1^Northwestern University, Chicago, IL; ^2^Columbia University Irving Medical Center, New York, NY; ^3^Department of Obstetrics and Gynecology, University of Utah Health, Salt Lake City, UT; ^4^RTI International, Triangle Park, NC; ^5^Eastern Virginia Medical School at Old Dominion University, Norfolk, VA; ^6^University of Pittsburgh Medical Center, Pittsburgh, PA; ^7^Brown University, providence, RI; ^8^Northwestern University Feinberg School of Medicine, Chicago, IL

**Objective**: Maternal health literacy (HL) has been linked to perinatal and long‐term health outcomes, yet its relationship with psychosocial well‐being in pregnancy is not well understood. We aimed to evaluate whether HL is associated with patient‐reported mental health (MH) outcomes and psychosocial support in pregnancy.

**Study Design**: Secondary analysis of data from a prospective multi‐site cohort of nulliparous participants enrolled at 8 US medical centers from 2010–2013. Participants completed the Rapid Estimate of Adult Literacy in Medicine–Short Form at 16–21 weeks’ gestation, which is an appropriate proxy for pre‐pregnancy HL as it is stable across pregnancy. Scores < 7 (≤8^th^ grade reading level) were categorized as inadequate. MH and psychosocial support outcomes, assessed at study visits between 6–29 weeks’ gestation, included: moderate‐to‐high anxiety (State‐Trait Anxiety Inventory–Trait >19), depressive symptoms (Edinburgh Postnatal Depression Scale >9), moderate‐to‐high stress (Perceived Stress Scale >13), high social support (Multidimensional Scale of Perceived Social Support >60), and good resilience (Connor‐Davidson Resilience Scale >59). Multivariable logistic regression estimated adjusted odds ratios (aORs) with confidence intervals (CIs), adjusting for confounding sociodemographic factors.

**Results**: Among 9, 878 participants with available data, 2, 175 (22%) had inadequate HL. Participants with adequate HL were older, more likely to be non‐Hispanic white, privately insured, and had greater education and income. Adequate HL was associated with lower odds of anxiety (aOR 0.86, 95% CI 0.76–0.98), depressive symptoms (aOR 0.82, 95% CI 0.74–0.90), and stress (aOR 0.74, 95% CI 0.68–0.81); and higher odds of reporting high social support (aOR 1.16, 95% CI 1.00–1.34) and good resilience (aOR 1.63, 95% CI 1.28–2.07) (Table).

**Conclusion**: Adequate HL was associated with better patient‐reported MH and psychosocial support in pregnancy. These relationships should be further explored as HL may be a modifiable factor to test in future interventions to improve maternal MH and support in the perinatal period.

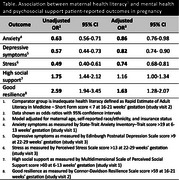

**10:30 AM – 12:00 PM**

## 910 | Nomogram **for Predicting Postpartum Hemorrhage After Term Vaginal Delivery**

Or Bercovich^1^; Ophir Blickstein^1^; Lior Friedrich^2^; Karolin Sokolik^3^; Yossi Geron^4^; Ohad Houri^5^; Daniela Chen^3^

^1^Helen Schneider Hospital for Women, Rabin Medical Center, Petach Tikva, Israel, Helen Schneider Hospital for Women, Rabin Medical Center, Petach Tikva, HaMerkaz; ^2^Helen Schneider Hospital for Women, Rabin Medical Center, Petah Tikva, Israel, Petah Tikva, HaMerkaz; ^3^Helen Schneider Hospital for Women, Rabin Medical Center‐Beilinson Hospital, Petach Tikva, Helen Schneider Hospital for Women, Rabin Medical Center‐Beilinson Hospital, Petach Tikva, HaMerkaz; ^4^Helen Schneider Hospital for Women, Rabin Medical Center, Petah Tikva, HaMerkaz; ^5^BCNatal Fetal Medicine Research Center, BARCELONA, Catalonia

**Objective**: To develop a predictive model and nomogram for postpartum hemorrhage (PPH) following term vaginal delivery.

**Study Design**: This retrospective study was conducted at a single tertiary care center. We included all vaginal deliveries at ≥37 gestational weeks (either normal vaginal delivery [NVD] or vacuum extraction [VE]) recorded between July 2012 to December 2023. The primary outcome was the occurrence of PPH, defined as bleeding that, in the clinician's judgment, exceeded physiological norms and posed a threat to maternal stability, thereby necessitating medical or surgical intervention. The full multivariate logistic regression included variables such as age, BMI, parity, diabetes, hypertensive disorders, chorioamnionitis, polyhydramnios, mode of delivery, gestational age, birthweight, oxytocin use, second‐stage duration, and neuraxial analgesia. The stepwise model, selected based on the Akaike Information Criterion, retained the variables gestational age (GA), clinical weight estimation at delivery, mode of delivery (NVD or VE), use of intrapartum oxytocin, and the duration of the second stage of labor. A nomogram was constructed based on the final logistic regression model. Internal validation and calibration were performed.

**Results**: A total of 29, 094 vaginal deliveries met the inclusion criteria, of which 978 cases (3.4%) were complicated by PPH. The final logistic regression model showed that higher gestational age (*p* < 0.001), higher clinical fetal weight estimation (*p* = 0.008), vacuum‐assisted delivery (*p* < 0.001), use of oxytocin (*p* = 0.022), and longer second stage (*p* < 0.001) were all independently associated with increased odds of PPH. Their respective predicted probabilities are represented in the nomogram (Figure 1).

**Conclusion**: The developed nomogram provides a clinically useful tool for individualized risk prediction of PPH after term vaginal birth. This may assist clinicians in identifying patients at higher risk and optimizing postpartum management strategies accordingly.

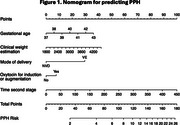

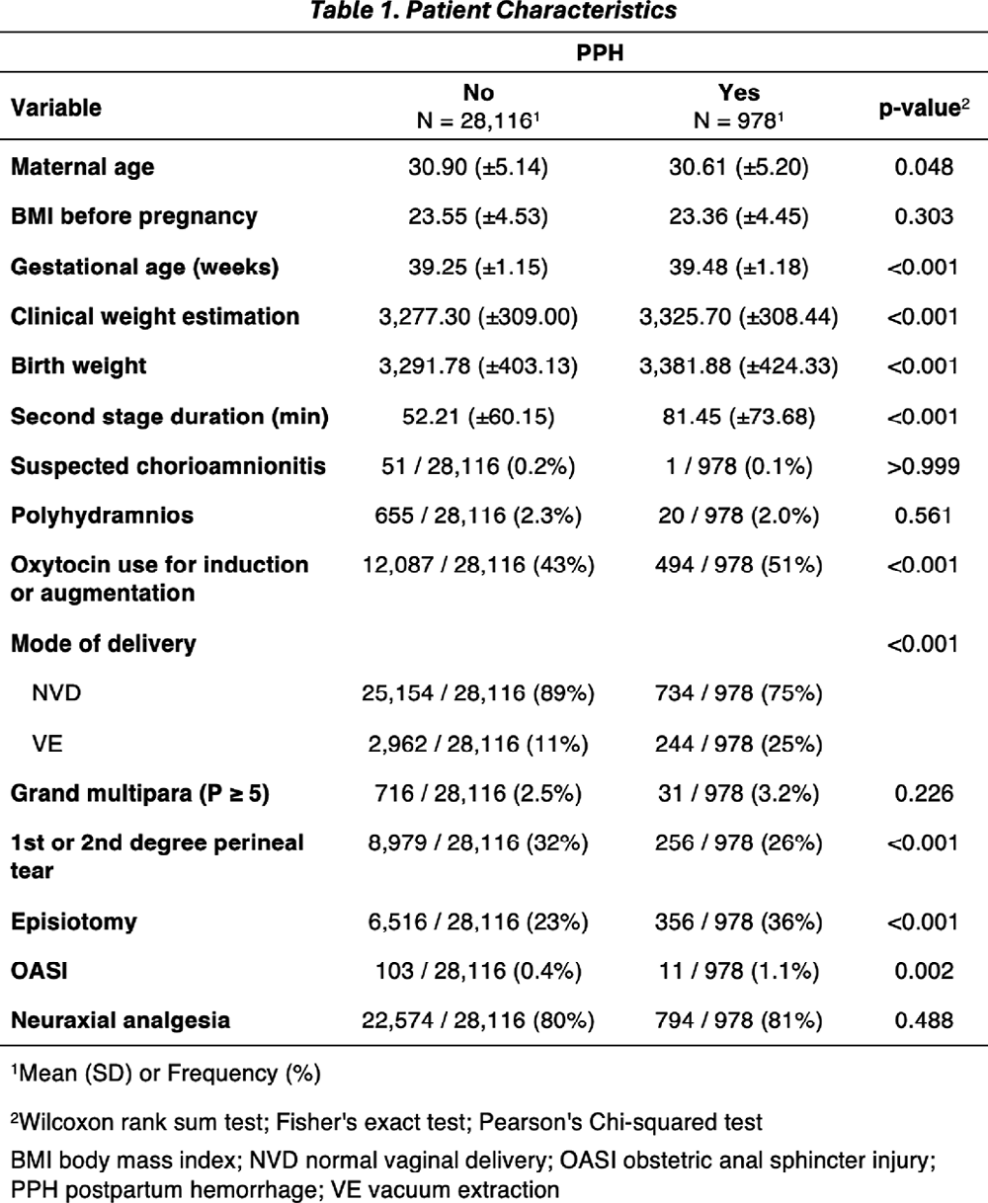

**10:30 AM – 12:00 PM**

## 912 | **Neonatal Respiratory Morbidity in Pregnancies with Great Obstetrical Syndromes at Risk for Preterm Delivery**

Pascalle F. Magdelijns^1^; Rachel L. Wiley^1^; Grace K. Noonan^2^; Cynthia Gyamfi‐Bannerman^1^

^1^Division of Maternal Fetal Medicine, Department of Obstetrics, Gynecology and Reproductive Sciences, University of California San Diego, San Diego, CA; ^2^Department of Obstetrics, Gynecology and Reproductive Science, University of California San Diego, San Diego, CA

**Objective**: The Great Obstetrical Syndromes (GOS) are designated as specific pregnancy complications (pre‐eclampsia, spontaneous preterm labor or PPROM, IUGR, placental abruption, and stillbirth) that stem from failure to achieve deep placentation. This adverse environment leads to increased placental glucocorticoids, which may promote fetal lung maturity. We assessed whether neonatal respiratory morbidity (NRM) in late preterm pregnancies with GOS was different to those without GOS, and whether betamethasone (BMZ) mitigated the effect.

**Study Design**: Secondary analysis of a multicenter, randomized controlled trial that assessed BMZ in non‐anomalous singleton gestations 34w0d – 36w5d at risk for preterm birth. Individuals with likelihood of utero‐placental pathology, but not designated as GOS (chronic hypertension, gestational diabetes and gestational hypertension) were excluded. The remaining participants were divided into 2 groups: GOS (defined above) and non‐GOS (all others at risk for late preterm delivery). The primary outcome, a composite neonatal respiratory outcome (defined in table 1), and other respiratory outcomes were compared between groups. Logistic regression models adjusted for confounders.

**Results**: Of 2497 participants, 2009 (80.5%) were affected by GOS and 488 (19.5%) were unaffected. GOS pregnancies were more likely nulliparous, while non‐GOS pregnancies were more likely >35 years old and had a previous liveborn with NRM. The primary outcome was significantly less frequent in the GOS group (11.9% vs 17.0%, *p* < 0.01), as was major respiratory morbidity (MRM) (8.8% vs 13.6%, *p* < 0.01, table 1), despite GOS pregnancies having a slightly lower GA at delivery (table 2). These findings remained significant after adjusting for confounders (table 1), and BMZ decreased the risk of the primary outcome (OR 0.79, 90% CI 0.62, 1.00) and MRM (OR 0.67, 95% CI 0.51, 0.88).

**Conclusion**: Neonates from GOS pregnancies were less likely to have NRM compared to those from non‐GOS pregnancies. Nonetheless, BMZ administration further mitigated this risk.

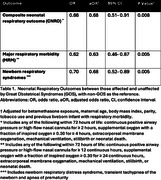

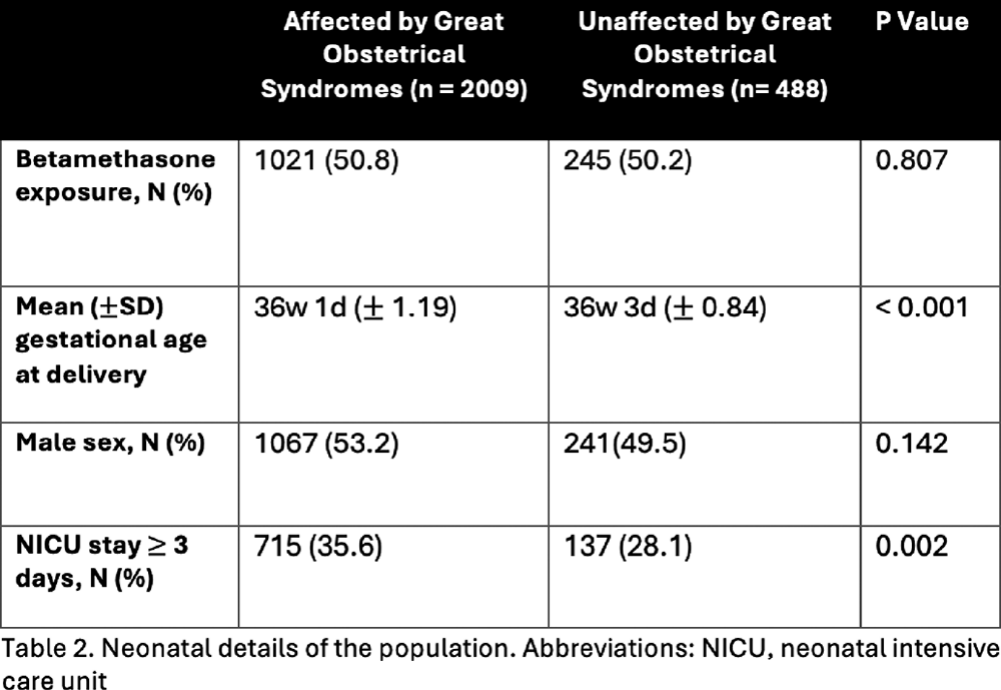

**10:30 AM – 12:00 PM**

## 913 | Consensus **on Vaginal Progesterone Use for Preterm Birth Prevention: The SUSTAIN‐V2 Consensus**

PM Gopinath^1^; Jaishree Gajaraj^2^; Shobha Gudi^3^; Mahesh Koregol^4^; Lavleen Sodhi^5^; Seema Sharma^6^; Ritu Khanna^7^; Seema Pandey^8^; Narahari Agasti^9^; Pradeep Acharya^10^; Hemant Deshpande^11^; Chaitanya Ganpule^12^

^1^Kavery Hospital, Chennai, Tamil Nadu; ^2^Mangai Clinic, Chennai, Tamil Nadu; ^3^Mothers Care Clinic, Mysore, Karnataka; ^4^Nova Fertility Centre, Bangalore, Karnataka; ^5^Dr Sodhi's Healthcare, Mohali, Punjab; ^6^CloudNine Hospital, Chandigarh, Chandigarh; ^7^Khanna Medical Centre, Varanasi, Uttar Pradesh; ^8^Seema Hospital, Khurd, Uttar Pradesh; ^9^Sreema Clinic, Bhubaneshwar, Orissa; ^10^Neha Clinic, Bhaubneshwar, Orissa; ^11^Dr. D. Y. Patil Medical College, Pune, Maharashtra; ^12^Pearl Women's Hospital and Yash IVF, Pune, Maharashtra

**Objective**: The SUSTAIN‐V2 (Safety, Utilization, and Therapeutic Benefits of Vaginal Progesterone) consensus aimed to assess expert agreement on the clinical use, efficacy, and safety of vaginal micronized progesterone for preterm labor prevention in high‐risk obstetric populations. The focus included optimal screening tools, patient selection, and timing of therapy initiation to maximize benefits in singleton and twin pregnancies

**Study Design**: A perception‐mapping consensus was conducted among experienced obstetricians with over 1500 man‐years of cumulative clinical experience. Participants rated 17 evidence‐based statements on a 5‐point Likert scale. A weighted score >100 indicated strong consensus. Data were analyzed using GraphPad 10.4.0.

**Results**: Of 17 statements, 13 reached weighted scores >100, reflecting strong agreement.

- High consensus supported using cervical length assessment with vaginal progesterone to manage high‐risk pregnancies (114.2).
- Routine daily use of 200 mg vaginal progesterone was endorsed for women with short cervix and prior preterm birth, including ART‐conceived pregnancies (119.7, 120.2).
- Early initiation before 20 weeks and combining cervical imaging with uterocervical angle assessment were also supported (114.8).
- Progesterone use in arrested labor and mild cervical shortening achieved strong agreement (116.3, 102.5).
- Use of 400 mg vaginal progesterone in ART pregnancies from mid‐trimester was also well supported (120.2).
- The treatment was considered well‐tolerated and guideline‐aligned across singleton and twin gestations (103.9).

Statements involving biomarkers, PCOS‐related luteal support, and PAPP‐A‐based personalization received lower scores (e.g., 39.7, 54.2), indicating areas requiring further research.

The overall mean response scores were: Agree: 60 ± 19 (95% CI 50 to 70), Strongly agree: 32 ± 22 (95% CI 20 to 43), *p* < 0.0001

**Conclusion**: The panel confirmed the safety and utility of vaginal micronized progesterone, in preterm birth prevention. Strong consensus was reached on early initiation and integration with cervical screening in both spontaneous and ART pregnancies

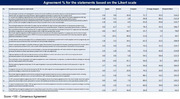

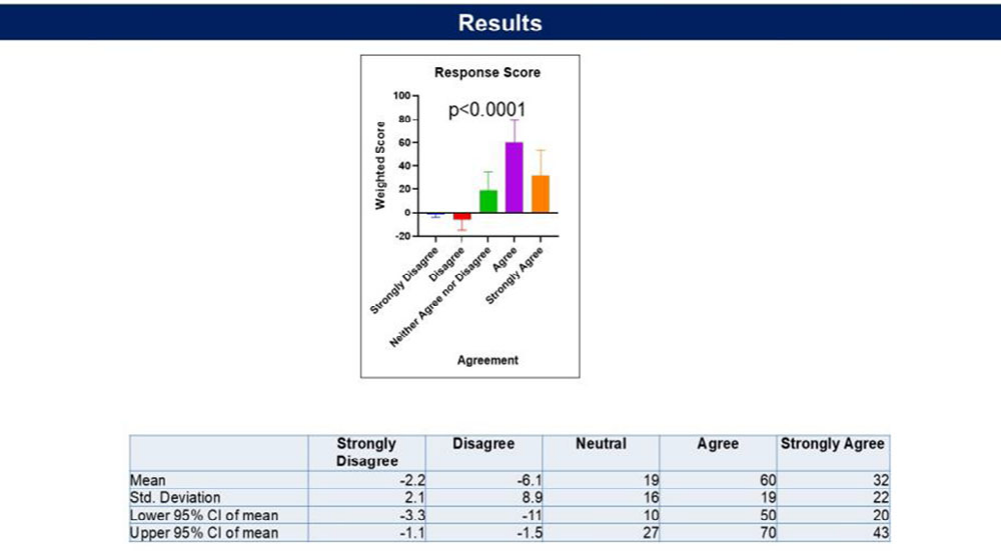

**10:30 AM – 12:00 PM**

## 914 | **Adverse Perinatal Outcomes Among Preterm Pregnancies Complicated by Chorioamnionitis**

Prakrunya Subhasree Badrinarayan^1^; Bharti Garg^2^; Amelia H. Gagliuso^3^; Isha Garg^2^; Irina Cassimatis^2^; Aaron B. Caughey^2^

^1^Oregon Health & Science University, Portland, OR; ^2^Oregon Health & Science University, Oregon Health & Science University, OR; ^3^Oregon Health and Science University, Portland, OR

**Objective**: Chorioamnionitis complicates 1–5% of births annually in the U.S. Studies have found associations between chorioamnionitis and preterm birth, and it has been implicated in the development of neonatal sepsis and perinatal morbidity. These risks have led to widespread use of broad‐spectrum antibiotics, though perinatal complications remain a concern. We evaluated the association between chorioamnionitis and adverse perinatal outcomes among preterm pregnancies in a modern cohort.

**Study Design**: We conducted a retrospective cohort study using California's linked vital statistics and hospital discharge data for deliveries between 2016 and 2020. We included singleton, non‐anomalous births with gestational age of 23–36 weeks without scheduled cesarean. Chorioamnionitis and perinatal outcomes were identified using ICD‐9 and ICD‐10 codes. We used Chi‐square tests and multivariable Poisson regression models.

**Results**: Among 76, 878 preterm births, 2.87% (*n* = 2, 207) had chorioamnionitis. Compared to those without chorioamnionitis, affected individuals had higher risk of severe maternal morbidity (8.3% vs 3.1% [aRR = 2.63; 95% CI 2.26–3.05]), postpartum hemorrhage (8.9% vs 4.1% [aRR = 2.08; 95% CI 1.80–2.40]), sepsis (2.8% vs 0.2% [aRR = 17.94; 95% CI 12.96–24.85]), ICU admission (1.2% vs 0.5% [aRR = 2.64; 95% CI 1.74–4.00]), and placental abruption (7.6% vs 5.8% [aRR = 1.36; 95% CI 1.16–1.59]). Neonates of individuals with chorioamnionitis had higher risk of respiratory distress syndrome (32.1% vs 19.1% [aRR = 1.65; 95% CI 1.54–1.76]), infant death (3.4% vs 0.9% [aRR = 3.23; 95% CI 2.48–4.21]), jaundice (50.8% vs 39.7% [aRR = 1.24; 95% CI 1.19–1.30]), and low Apgar scores (< 3) at 5 minutes (2.2% vs 0.6% [aRR = 2.83; 95% CI 2.00–4.00]).

**Conclusion**: Chorioamnionitis is linked to adverse perinatal outcomes in preterm births, even after adjusting for confounders. As preterm birth is associated with neonatal morbidity, the presence of chorioamnionitis may further increase such risks.

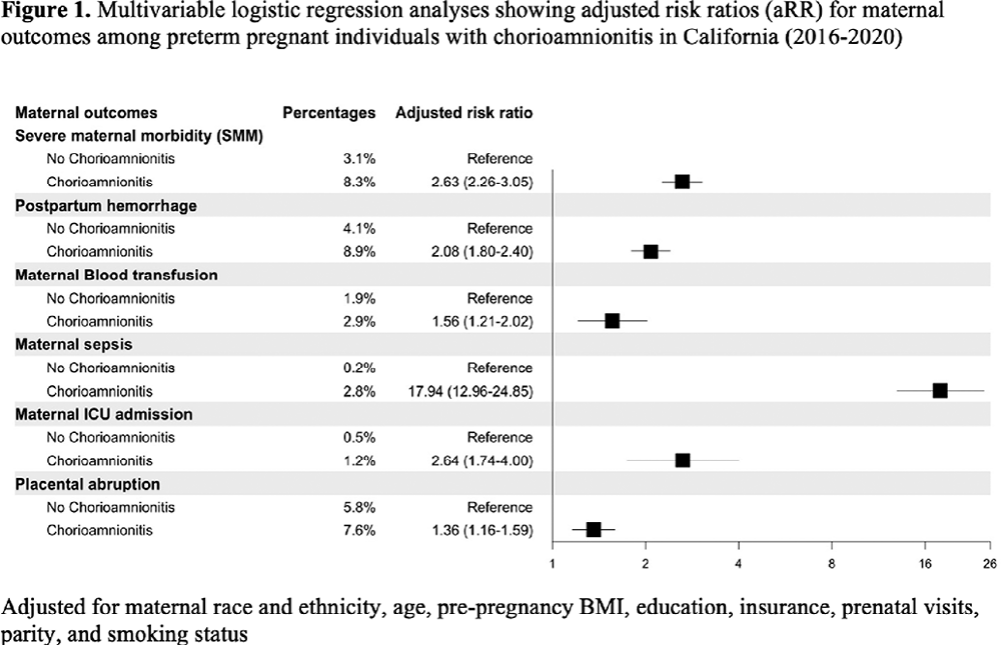

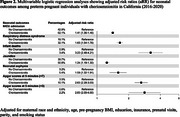

**10:30 AM – 12:00 PM**

## 915 | **Maternal Hyperleptinemia Induces Sex‐Specific Hypertension in Male Mice Offspring**

Puja Punukollu^1^; Padmashree Woodham^1^; Jessica Faulkner^2^; Mona Elgazzaz^2^; Gabrielle Hernandez^3^; Amalia Brawley^1^; Desmond Moronge^2^; Gibson Cooper^2^

^1^Medical College of Georgia, Augusta, GA; ^2^Medical College of Georgia, Medical College of Georgia, GA; ^3^Medical College of Georgia, Johns Creek, GA

**Objective**: Elevated leptin levels in pregnancy is associated with preeclampsia, hypertension and adverse fetal outcomes such as low birth weight. In a novel animal model, our laboratory demonstrated that elevating leptin levels in pregnant mice induces maternal hypertension, vascular endothelial dysfunction and decreases offspring weight. However, whether hyperleptinemia of pregnancy induces long‐term adverse cardiovascular outcomes in offspring is unknown. We hypothesized that leptin infusion in pregnant mice in mid‐late gestation induces hypertension in adult offspring.

**Study Design**: We administered mouse recombinant leptin (LEP, 0.9 mg/kg/day) or saline (XLEP) via subcutaneous mini‐osmotic infusion pumps from gestation day 11 to end of gestation. Offspring were weaned and aged to 6 months, and one female and one male mouse utilized from each litter for blood pressure (BP) measurement. Offspring were implanted with radiotelemeters for conscious BP. After 7 days of baseline on normal salt diet (NSD), mice were transitioned to 4% high salt diet (HSD) for 7 days, followed by HSD + angiotensin II (ANG II, 490 ng/kg/min) for 7 days. Groups included are XLEP+NSD, XLEP+HSD, XLEP+HSD+ANG II, LEP+NSD, LEP+HSD, and LEP+HSD+ANG II for both sexes.

**Results**: At baseline and HSD only conditions, leptin did not produce an effect on BP or HR in offspring. Interestingly, in Ang‐II+HSD, only LEP males showed significant elevation in blood pressure compared to XLEP male offspring (Mean ± SEM; 133 ± 3.9 vs. 101 ± 8.4 respectively, *p* = 0.003), with no difference among the LEP female offspring compared to XLEP females (Mean ± SEM; 124.6 ± 8 vs. 122.4 ± 4.9, respectively, *p* > 0.05)

**Conclusion**: Leptin‐induced preeclampsia in mice causes male‐specific in‐utero programming, resulting in increased Ang‐II sensitivity to hypertensive stimuli – a *nove*l demonstration of sex‐specific fetal cardiovascular programming.

**10:30 AM – 12:00 PM**

## 916 | **Perinatal Outcomes of Transgender Individuals with Prior Testosterone Use: An Epic Cosmos Cohort Analysis**

Rachael E. Jackson^1^; Anne‐Sophie van Wingerden^1^; Rachel Shamoun^1^; Gianna L. Wilkie^2^

^1^University of Massachusetts Chan Medical School, University of Massachusetts Chan Medical School, Worcester, MA; ^2^University of Massachusetts Chan Medical School, Worcester, MA

**Objective**: To evaluate perinatal outcomes among transgender and gender diverse (TGD) birthing persons with testosterone use prior to pregnancy.

**Study Design**: We conducted a retrospective cohort study of TGD individuals with a prior pregnancy stratified by testosterone exposure. Demographic, clinical, and perinatal outcomes were compared using descriptive statistics.

**Results**: Of 755 transgender men, 49.8% reported prior testosterone use. Compared to those with prior testosterone use, individuals without testosterone use were more likely to be younger and of white race. There were no significant differences between groups in initial body mass index, rates of hypertensive disorders, or diabetes. Of 843 pregnancies, 49.7% occurred in individuals with prior testosterone use. Among those without prior testosterone use, pregnancy loss or termination before 20 weeks’ gestation was significantly more common (32% vs 23%, *p* < 0.005). Postpartum depression (PPD) was less common among those without prior testosterone use (6% vs 9%, *p* < 0.05). No other differences in perinatal outcomes were observed, including method of delivery, preterm delivery, pregnancy induced hypertension or gestational diabetes.

**Conclusion**: Prior testosterone use among TGD individuals is not associated with adverse perinatal outcomes. However, pregnancy loss or termination before 20 weeks was more frequent among individuals without prior testosterone use. This finding may reflect differences in pregnancy planning, as individuals using testosterone are recommended to discontinue it prior to conception. Further, prior testosterone use was associated with a higher prevalence of PPD. The underlying cause for this association remains unclear but may reflect needs that are unique to this population. These findings underscore the importance of mental health screening and support for TGD individuals, particularly those with prior testosterone use. Further research is needed to explore the psychosocial and structural factors that influence pregnancy outcomes and postpartum care in TGD populations.

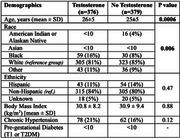

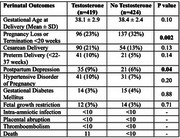

**10:30 AM – 12:00 PM**

## 917 | **The** Added **long‐Term Neurologic Risk of Multiparity: Results from a Population‐Based Cohort Study**

Rachel Rappaport^1^; Gil Gutvirtz^2^; Eyal Sheiner^3^; Tamar Wainstock^4^; Roy Kessous^5^; Ruslan Sergienko^6^

^1^Soroka medical center, Soroka medical center, HaDarom; ^2^Department of Obstetrics and Gynecology, Soroka University Medical Center, Ben‐Gurion University, Metar, HaDarom; ^3^Soroka University Medical Center, Faculty of Health Sciences, Ben‐Gurion University of the Negev, beer sheva, HaDarom; ^4^Department of Epidemiology, Biostatistics and Community Health Sciences, Faculty of Health Sciences, Ben‐Gurion University of the Negev, Beer Sheva, HaDarom; ^5^Soroka University Medical Center, Soroka University Medical Center, HaDarom; ^6^Ben‐Gurion University of the Negev, ben gurion, HaDarom

**Objective**: Multiparity (ε2 deliveries), and particularly grand multiparity (ε5 deliveries), carries several potential risks for maternal short and long‐term health, possibly due to the cumulative physiological stress of repeated pregnancies. These include mainly an increased risk for cardiovascular and metabolic morbidity, while other health aspects are less investigated. In this study we aimed to evaluate a potential association between increasing parity and the long‐term risk of future neurological morbidity in mothers.

**Study Design**: A population‐based cohort study was conducted between the years 1991–2021, and included only women who had concluded childbearing, so parity was definite. Data regarding the incidence of maternal neurological morbidity was extracted from community and hospitalization records. Kaplan‐Meier survival curves were constructed to compare the cumulative incidence over time of maternal neurological morbidity between the study groups. A Cox proportional hazards model was used to control for possible confounders.

**Results**: The study included 54, 369 women with varying parity between 1 to 6 or more births. There was a positive association found between increasing parity and future neurological morbidity (Table). Additionally, the cumulative incidence of neurological morbidity over time (Figure) was significantly higher with increasing parity. After applying the Cox regression model, adjusting for the use of fertility treatments and ethnicity, and comparing to nulliparity as a reference group, every additional birth was found to independently increase the risk for future neurological morbidity of the mother.

**Conclusion**: In our cohort, increasing parity was associated with a significantly higher risk for future neurological morbidity in mothers. Further research is warranted to delineate the cumulative physiological, hormonal, and psychological stressors associated with repeated pregnancies, which may contribute to an increased risk of maternal neurological morbidity.

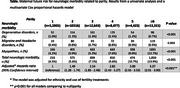

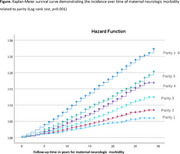

**10:30 AM – 12:00 PM**

## 918 | **Not All** Ripened **Are Delivered Equal: Cesarean Clues Revealed**

Anne‐Sophie van Wingerden^1^; Heidi K. Leftwich^2^; Rachel Shamoun^2^; Vanessa Chin^3^

^1^University of Massachusetts Chan Medical School, University of Massachusetts Chan Medical School, Worcester, MA; ^2^UMass Chan Medical School, Worcester, MA; ^3^UMass Chan Medical School, UMass Chan Medical School, MA

**Objective**: To identify factors associated with cesarean delivery among patients undergoing cervical ripening for labor induction.

**Study Design**: This was a retrospective cohort study of patients undergoing induction of labor with cervical ripening at a single academic institution from 1/1/2020 to 12/31/2021. Inclusion criteria were singleton gestation, gestational age ≥37 weeks, and available pre‐pregnancy BMI. Exclusion criteria included prior cesarean delivery, underweight BMI (< 18.5), pre‐gestational diabetes, significant fetal anomalies, and intrauterine fetal demise. Cervical ripening typically followed institutional guidelines recommending dual‐agent therapy (cervical balloon plus pharmaceutical agent). The primary outcome was mode of delivery. Associations between demographic and clinical factors and cesarean delivery were evaluated using descriptive statistics and multivariable analysis.

**Results**: Of 5712 charts reviewed, 1924 met inclusion criteria. Among this cohort, factors significantly associated with increased risk of cesarean delivery included nulliparity, minimal cervical dilation at initiation of ripening, and absence of cervical balloon use. In contrast, multiparity and the use of dual‐agent cervical ripening were associated with a higher likelihood of vaginal delivery. In both multiparity and nulliparity, total ripening time was longer in those that delivered via cesarean. These findings suggest that both patient characteristics and modifiable clinical practices can impact cesarean risk during induction.

**Conclusion**: Nulliparity, limited cervical dilation, and lack of balloon catheter use were associated with increased cesarean rates in patients undergoing cervical ripening. Notably, absence of cervical balloon use—a modifiable factor—was linked to higher cesarean risk, highlighting the importance of adherence to standardized induction protocols. Findings support continued evaluation of induction practices and patient education to promote evidence‐based decision‐making and reduce primary cesarean births, especially among rising public scrutiny of induction methods via social media platforms.

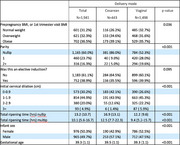

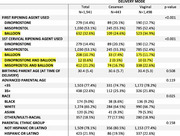

**10:30 AM – 12:00 PM**

## 919 | **Adverse Childhood Experiences and Maternal Infant Bonding in a High‐Risk Obstetrics Population**

Rachel L. Wiley^1^; Dana R. Canfield^2^; Emmanuel O. Elijah^3^; Cynthia Gyamfi‐Bannerman^1^; Minhazur R. Sarker^1^; Marni B. Jacobs^1^

^1^Division of Maternal Fetal Medicine, Department of Obstetrics, Gynecology and Reproductive Sciences, University of California San Diego, San Diego, CA; ^2^Department of Obstetrics & Gynecology, University of Washington, Seattle, WA, Seattle, WA; ^3^Department of Obstetrics, Gynecology and Reproductive Sciences, University of California San Diego, San Diego, CA

**Objective**: Maternal adverse childhood experiences (ACE) are increasingly recognized to have significant impact on parenting outcomes. This study examines the association between maternal ACEs and early maternal infant bonding in a high‐risk obstetric population.

**Study Design**: This is a secondary analysis of prospective cohort of patients recruited from the MFM practice at a single center. Participants were enrolled in the third trimester and followed longitudinally with surveys on health and birth experiences. At enrollment, participants completed the ACE questionnaire and were categorized into exposure groups: low (ACE = 0), moderate (ACE 1–3), and high (ACE > 4) exposure. At six weeks postpartum, participants completed perinatal mental health scales including Edinburg Postnatal Depression Scale (EPDS, positive considered > 10), Generalized Anxiety Disorder‐7 (GAD‐7, positive considered > 4), and the Mother‐Infant Bonding (MIB) Scale, for which a score > 3 indicates impaired bonding.

**Results**: Of 198 enrolled participants, 119 (60.1%) completed both the ACE and MIB scales and were included in the analysis. Of these, 31 (26.1%) were low, 64 (53.8%) were moderate, and 24 (20.2%) were high ACE exposure. Higher ACE exposure group was significantly associated with a history of a mental health diagnosis, and lower household income (all *p* ≤ 0.01, Table 1). Rates of impaired bonding were 19.4% (low), 29.7% (moderate), and 37.5% (high), which was not significantly different between groups (*p* = 0.24). Rates of positive postpartum mental health screen were significantly different (*p* < 0.01).

**Conclusion**: In our high‐risk population, maternal ACEs were not associated with impaired early infant bonding, but were associated with a positive postpartum mental health screen. This is particularly relevant for high risk populations, where both ACEs and perinatal mental health problems are prevalent. This adds to the developing literature that while maternal ACES are associated with perinatal psychopathology, they may not directly be associated with early adverse childhood outcomes.

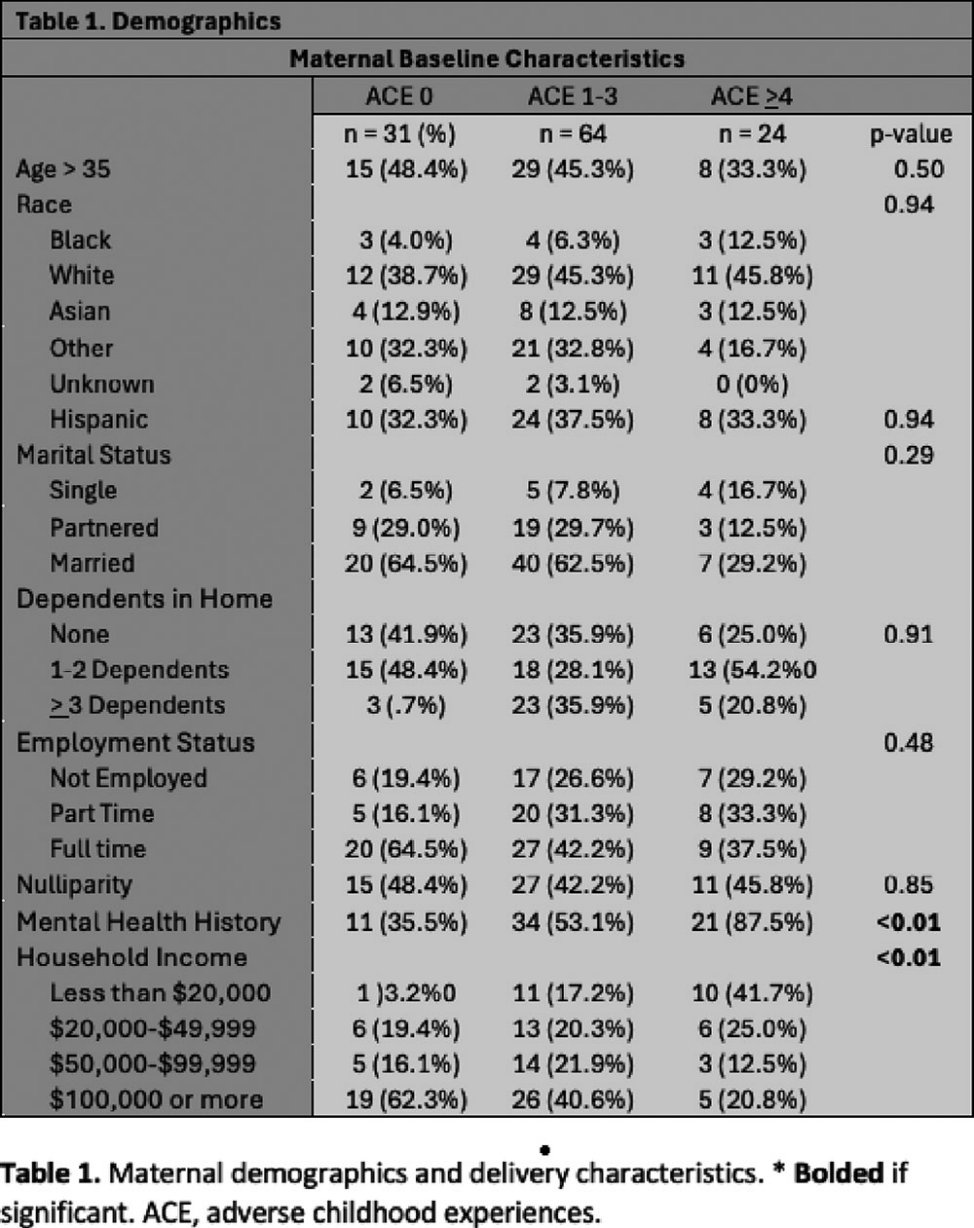

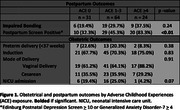

**10:30 AM – 12:00 PM**

## 920 | **Adverse Outcomes in Patients With Early Dysglycemia in Pregnancy Compared With Preexisting Type 2 Diabetes**

Rana K. Fowlkes^1^; Kathryn Anthony^1^; Kim Boggess^2^; Ashley N. Battarbee^3^

^1^University of Alabama at Birmingham, Birmingham, AL; ^2^University of North Carolina at Chapel Hill, Chapel Hill, NC; ^3^Center for Research in Women's Health, University of Alabama at Birmingham, Birmingham, AL

**Objective**: ACOG and ADA recommend screening for undiagnosed pregestational diabetes (DM) at prenatal care onset, but the benefit of screening for early dysglycemia that is not overt DM remains unclear. We aimed to compare outcomes among patients with early dysglycemia to those with preexisting type 2 DM (T2D).

**Study Design**: This was a secondary analysis of a randomized, placebo‐controlled trial of metformin for insulin‐treated T2D in pregnancy. Participants with baseline HbA1c and primary composite outcome data (Table 2) were included. Those diagnosed with DM during pregnancy with HbA1c > 6.5% or missing method of diabetes were excluded. Early dysglycemia was defined as abnormal 2‐step, 1‐step, or 1hr GCT < 23 weeks and HbA1c < 6.5%. We compared baseline characteristics and outcomes between patients with early dysglycemia, T2D with good control (HbA1c < 6.5%) and T2D with poor control (HbA1c ε 6.5%). Multivariable logistic regression estimated the association of early dysglycemia with outcomes, compared to T2D with good control and to T2D with poor control.

**Results**: Of 610 eligible participants, 83 (14%) had early dysglycemia, 150 (24%) T2D with good control, and 377 (62%) T2D with poor control. Groups differed by multiple baseline characteristics (Table). Composite neonatal morbidity differed by maternal DM status (46% vs 58% vs 82%) as did all secondary outcomes (Figure). Compared to T2D with good control, early dysglycemia was not associated with the neonatal composite, but had lower odds of hyperbilirubinemia (Figure). Compared to T2D with poor control, early dysglycemia was associated with lower odds of neonatal composite (aOR 0.33, CI 0.16–0.67) and other secondary outcomes (Figure).

**Conclusion**: In this diverse cohort with detailed data on DM diagnosis and HbA1c in early pregnancy, patients with early dysglycemia appeared to have outcomes similar to those with well‐controlled preexisting T2D, but improved outcomes compared to those with poorly‐controlled T2D. These findings may inform counseling and management of patients diagnosed with early dysglycemia without overt T2D.

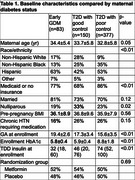

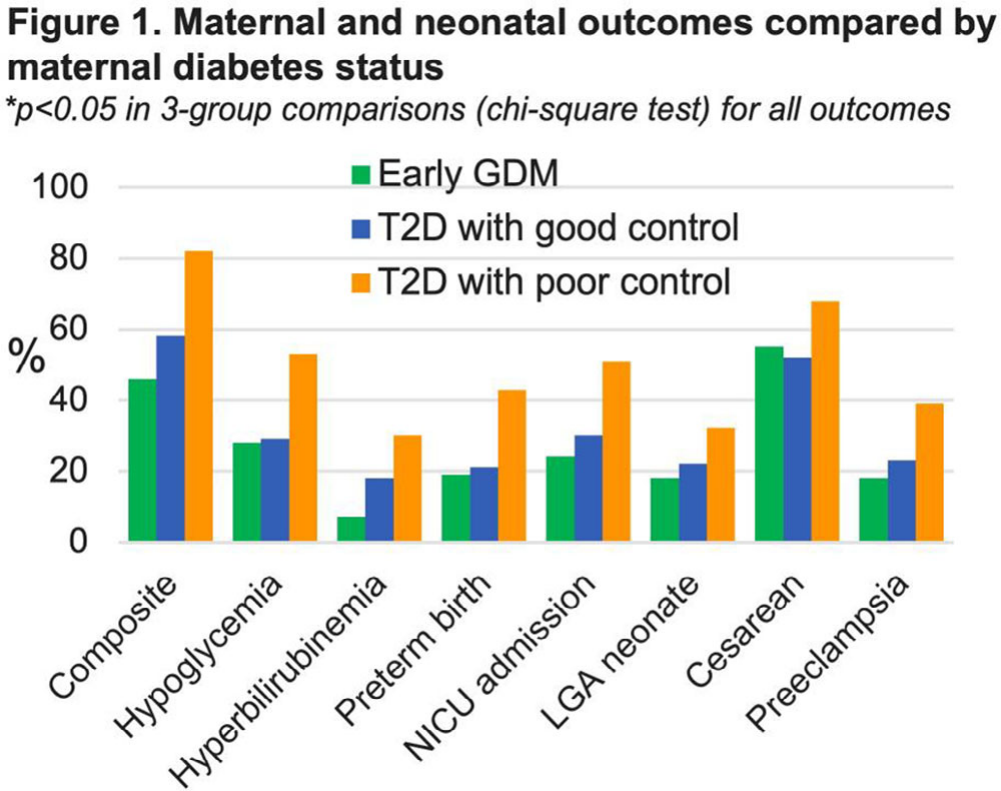

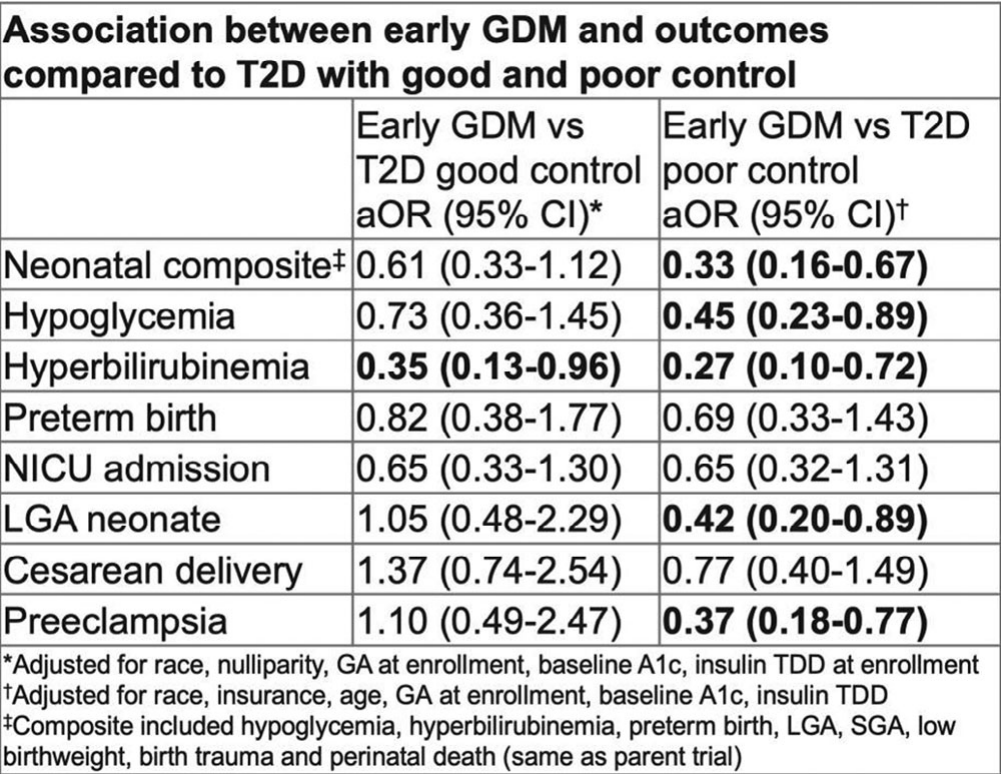

**10:30 AM – 12:00 PM**

## 921 | **Impact of Papaverine on Labor Outcomes Among Primiparous Patients Undergoing Induction of Labor**

Raneen Abu Shqara^1^; Neta Hoffman^2^; Nadir Ganem^3^; Lior Lowenstein^3^; Maya Frank Wolf^3^

^1^Galilee Medical Center, Nahariya, HaZafon; ^2^Galilee Medical Center, Galilee Medical Center, HaZafon; ^3^Galilee Medical Center, Naharyia, HaZafon

**Objective**: Antispasmodics such as papaverine, a musculotropic smooth muscle relaxant, are widely used during labor to facilitate cervical dilatation and to potentially shorten labor duration, though their efficacy and safety are unclear. We aimed to evaluate the impact of papaverine administered during labor induction, on maternal and neonatal outcomes among primiparous women.

**Study Design**: This retrospective cohort study included 1, 889 primiparous women who underwent labor induction with intravenous oxytocin during 2020–2024. The patients were classified according to papaverine administration: none (*n* = 1, 185), one dose (*n* = 618), and two doses (*n* = 86). The primary outcome was the mode of delivery. Secondary maternal outcomes included delivery within 24 hours, postpartum hemorrhage, and puerperal endometritis. Neonatal outcomes included a 5‐minute Apgar score < 7, umbilical cord pH < 7.1, and admission to the neonatal intensive care unit. Multivariate logistic regression analysis was conducted to identify predictors of cesarean delivery.

**Results**: Among women who received one and two doses of papaverine, compared to those who received none, the rates were lower of cesarean delivery due to non‐progressive labor (11.5% and 14.0%, vs. 17.1%, *p* = 0.002) (Table). Vaginal delivery rates were higher among women who received papaverine before rupture of membranes (77.0%) than those who received it after rupture (73.0%) and those who did not receive papaverine (66.1%) (*p* < 0.001) (Figure). In multivariate analysis, papaverine administration was associated with a reduced likelihood of cesarean delivery (odds ratio 0.64, 95% confidence interval 0.50–0.81, *p* < 0.001). Neonatal outcomes, including Apgar scores and admission to the neonatal intensive care unit, were similar across the groups.

**Conclusion**: Among primiparous patients, papaverine administration, particularly before rupture of membranes, was associated with lower cesarean delivery rates due to non‐progressive labor and with shorter labor duration, yet similar adverse maternal and neonatal outcomes. Randomized controlled studies are needed to confirm these findings.

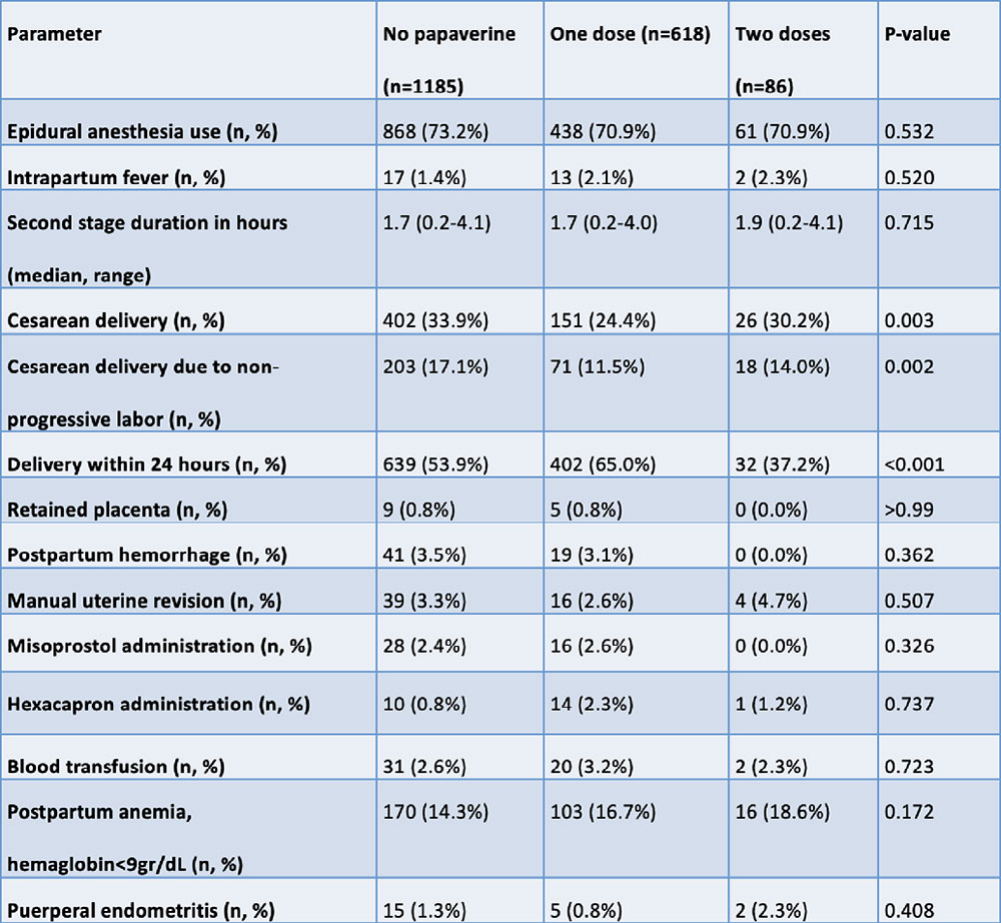

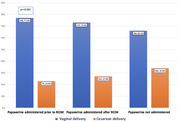

**10:30 AM – 12:00 PM**

## 922 | Outcomes **in Stage II Twin‐Twin Transfusion Syndrome With Donor Intermittent Umbilical Artery Doppler Abnormalities**

Raphael C. Sun^1^; Andrew H. Chon^1^; Lisa M. Korst^2^; Arlyn S. Llanes^3^; Martha A. Monson^4^; Ramen H. Chmait^3^

^1^Oregon Health & Science University, Portland, OR; ^2^Childbirth Research Associates, North Hollywood, CA; ^3^Keck School of Medicine of USC, University of Southern California, Keck School of Medicine of USC, University Of Southern California, CA; ^4^Intermountain Healthcare, Salt Lake City, UT

**Objective**: Per Quintero TTTS staging, donor umbilical artery (UA) intermittent absent or reversed end diastolic flow (A/REDF) is classified as stage I or II depending on donor twin bladder status, whereas UA persistent A/REDF is classified as stage III. However, this distinction between intermittent and persistent UA‐A/REDF has led to ambiguity between centers in regard to staging of TTTS. Here, we compared laser surgery outcomes in stage II patients with intermittent UA‐A/REDF (Stage‐II Intermittent) vs stage II patients with a normal UA waveform (Stage‐II Normal), using stage III patients with persistent donor twin UA‐A/REDF (Stage‐III) for comparison.

**Study Design**: Three groups of monochorionic diamniotic twins with TTTS treated with laser surgery (2006–2023) were analyzed: (1) Stage‐II Normal; (2) Stage‐II Intermittent; and (3) Stage‐III. The groups were compared in bivariate analysis and in a multiple logistic regression model for the primary outcome of donor twin fetal demise (IUFD).

**Results**: The 400 study patients were divided into Stage‐II Normal (*n* = 126), Stage‐II Intermittent (*n* = 62), and Stage‐III (*n* = 212). In bivariate analyses, the rates of donor IUFD were 7.1% vs 8.1% vs 29.2%, *p*< .0001, respectively. Dual survivorship was 84.1% vs 82.3% vs 62.7%, *p*< .0001, respectively. Multiple covariates were eligible for inclusion in the logistic regression model, including gestational age at the procedure, the preoperative estimated fetal weight of the donor, and the presence of arterioarterial anastomoses. Upon adjustment in a multiple logistic regression model, the Stage‐II Intermittent group did not have an increased risk of donor IUFD vs Stage‐II Normal (aOR 0.51, 95% CI 0.15–1.79).

**Conclusion**: TTTS stage II patients with intermittent UA‐A/REDF did not have increased risk of donor IUFD compared to stage II patients with normal UA Dopplers. These findings support the traditional Quintero staging classification, and only persistent UA‐A/REDF should be classified as TTTS stage III.

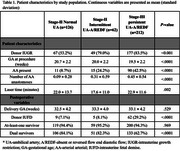

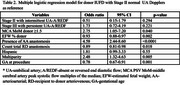

**10:30 AM – 12:00 PM**

## 923 | **The THEA Model: Outcomes of an Innovative Prenatal Care Model Incorporating Home Blood Pressure Monitoring**

Anna S. Graseck; Lisa D. Levine; Rebecca F. Hamm

Perelman School of Medicine, University of Pennsylvania, Philadelphia, PA

**Objective**: The traditional model of prenatal care requires frequent, in‐person prenatal visits, but ACOG recently endorsed care models with decreased visit frequency and consideration of home blood pressure (BP) monitoring. There is concern, however, that important diagnoses, such as hypertensive disorders of pregnancy (HDP), may be missed with fewer in‐person visits. We aimed to evaluate outcomes of an innovative prenatal care model (THEA) that incorporates reduced visits along with home BP monitoring compared to usual in‐office care.

**Study Design**: This retrospective cohort study compared deliveries over a 6‐month period (7/2022–12/2022) at a site utilizing THEA for all patients to a second site in the same health system utilizing traditional prenatal care. Traditional care consists of visits every 4 weeks in the first two trimesters, every 2 weeks beginning at 28 weeks, and weekly at 36 weeks. In THEA, visits were every 4–6 weeks until 36‐weeks, a reduction of 5 in‐person visits. High‐risk patients in THEA had an additional 3 telemedicine visits. BP was monitored weekly via text message. 1:1 propensity score matching balanced parameters associated with delivery site, including age, race, ethnicity, nulliparity, insurance, diabetes, smoking, and body mass index. Patients with chronic hypertension were excluded from analysis. The primary outcome was the rate of HDP. Secondary outcomes are shown in the Table.

**Results**: After propensity score matching, 2, 378 patients were included (1, 189 in each group). The rate of HDP was 26.5% with no difference between groups overall or by severity (Table). Patients in the THEA model had fewer prenatal visits (8 IQR[7–10] vs 11 IQR[9–12], *p* < 0.001). There was no difference in GA at delivery overall or among patients with HDP (39 weeks in both groups, *p* = 0.62). Additionally, there were no differences in severity of HDP, delivery mode, maternal morbidity, or fetal demise.

**Conclusion**: An innovative prenatal care program that allowed for fewer in‐person prenatal visits in conjunction with home BP monitoring did not impact HDP diagnosis, overall morbidity, or other perinatal outcomes.

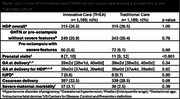

**10:30 AM – 12:00 PM**

## 924 | **Fetal** Aortic **Blood Flow Velocity is Associated with Spontaneous Preterm Birth in Uncomplicated Pregnancies**

Rebecca Horgan^1^; Erkan Kalafat^2^; Elena Sinkovskaya^1^; Alfred Abuhamad^3^; George R. Saade^1^

^1^Eastern Virginia Medical School at Old Dominion University, Norfolk, VA; ^2^Koc University Hospital, Istanbul, Koc University Hospital, Istanbul, Istanbul; ^3^Eastern Virginia Medial School at Old Dominion University, Norfolk, VA

**Objective**: To determine whether fetal cardiac function markers on second‐trimester echocardiogram are associated with adverse pregnancy outcomes.

**Study Design**: We performed a prospective cohort study of non‐anomalous pregnancies at <13 + 6 weeks gestation without umbilical cord or placental abnormalities. Patients underwent research visits at specific gestational ages, including fetal echocardiography evaluation at 20–21 weeks’ gestation. Pregnancy outcomes were classified into four categories: (1) term without complications (reference group), (2) term with hypertensive disorders of pregnancy (HDP) and/or fetal growth restriction (FGR), (3) preterm birth with HDP and/or FGR, and (4) spontaneous preterm birth (sPTB) without HDP/FGR. We used competing risks regression to evaluate associations between fetal cardiac parameters and outcome categories, treating term birth without complications as the censoring event. Key echocardiographic measures included aortic blood flow velocity, mitral and tricuspid E/A ratios, and pulmonary artery flow velocity. Models were adjusted and standardized, and hazard ratios (HR) were reported per standard deviation (SD) increase in each parameter.

**Results**: Among 506 patients, 47 (9.3%) experienced spontaneous preterm birth without HDP and/or FGR. Higher fetal aortic blood flow velocity was significantly associated with increased risk of sPTB (adjusted HR per SD: 1.39, 95% CI: 1.12–1.72, *p* = 0.002). This association was not observed for preterm birth with HDP and/or FGR (HR: 1.03, 95% CI: 0.77–1.39, *p* = 0.83) or term birth with HDP and/or FGR (HR: 0.88, 95% CI: 0.67–1.16, *p* = 0.38) (Figure). Rates of sPTB increased across aortic blood flow velocity categories: 7.0% (≤80 cm/s), 12.5% (81–90 cm/s), and 28.6% (>90 cm/s). No significant associations were found for mitral E/A ratio, tricuspid E/A ratio, or pulmonary artery velocity.

**Conclusion**: Elevated second‐trimester fetal aortic blood flow velocity is associated with subsequent sPTB without HDP or FGR. This may be related to fetal adaptation to utero‐placental insufficiency which in turn prevents HDP and FGR, but not sPTB.

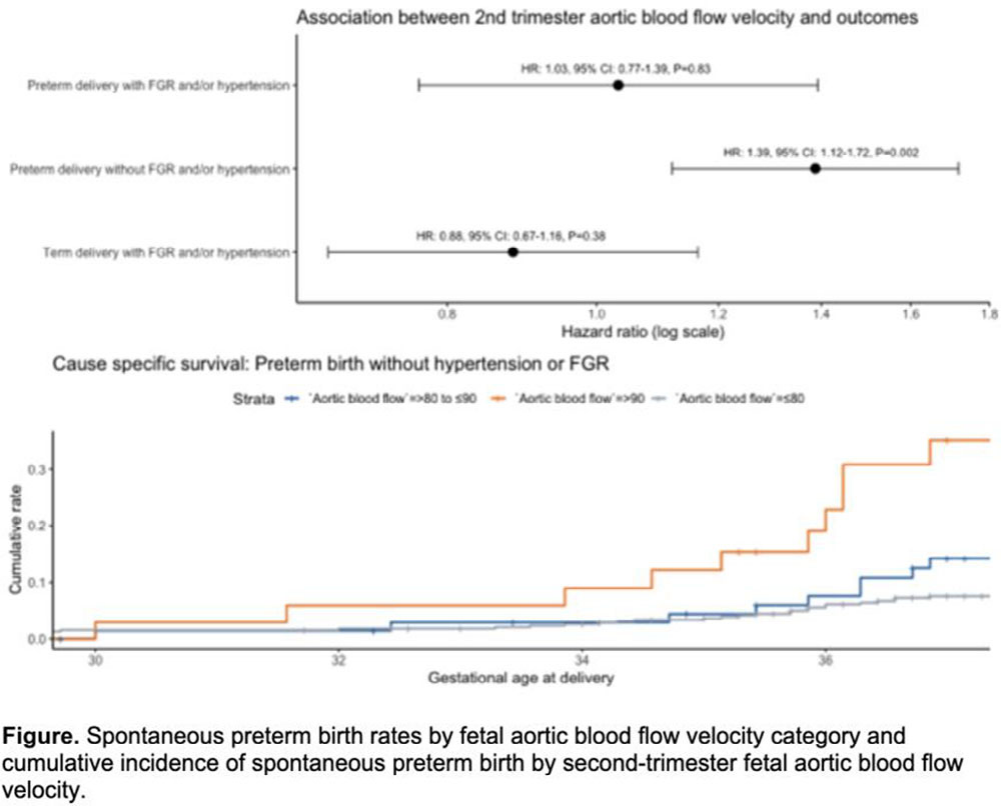

**10:30 AM – 12:00 PM**

## 925 | **Do** Adverse **Perinatal Events Moderate Response to Behavioral Activation Psychotherapy: Findings From the SUMMIT Trial**

Laura La Porte^1^; Richard Silver^1^; Alison M. Stuebe^2^; Aditya Anand^3^; Daisy Singla^4^

^1^Endeavor Health, Evanston, IL; ^2^University of North Carolina at Chapel Hill School of Medicine, Chapel Hill, NC; ^3^Lunenfeld‐Tanenbaum Research Institute, Sinai Health, Sinai Health/Toronto, ON; ^4^Centre for Addiction and Mental Health, Toronto, ON

**Objective**: Adverse perinatal events (APE) are associated with maternal depression, anxiety, and trauma symptoms. However, it is not known whether APE modify the effectiveness of behavioral activation (BA) to treat perinatal mood disorders.

**Study Design**: BA delivered to perinatal participants in the Scaling Up Maternal Mental Healthcare by Increasing Access to Treatment (SUMMIT) trial significantly reduced depression (EPDS), anxiety (GAD‐7) and trauma (PCL‐6) measured at 3‐months post‐treatment. In this secondary analysis, 12‐month mood outcomes were used to compare participants with and without one or more APE (pre‐eclampsia, operative vaginal delivery, unplanned cesarean, emergency cesarean, excessive bleeding, blood transfusion, NICU admission, and fetal loss). Chi‐square and T‐Tests were performed along with MANCOVA to further evaluate APE's influence on BA efficacy after controlling for relevant covariates (age, marital status, education, race/ethnicity, immigration status, treatment dosage, provider type, treatment modality, nulliparous status, perceived social support, participant satisfaction and baseline symptom scores).

**Results**: 519 (46%) of the 1119 participants had >1 APE and their 12‐month mood scores were compared to the 600 non‐APE cases (Table). Clinical symptom scores were statistically significant and worse in all groups (EPDS, GAD‐7 and PCL‐6; *p* < 0.05) among those participants with APE versus non‐APE. However, absolute score differences did not appear to be clinically meaningful (i.e., < 2 units for each APE versus non‐APE comparison). In addition, similar proportions of APE and non‐APE subjects had scores in the normal screening range, respectively (EPDS< 10, 57.4 vs. 62.3; GAD‐7< 10, 77.2 vs.79.3; PCL‐6< 14, 57.3 vs. 60.0).

**Conclusion**: While APE do appear to influence peripartum BA treatment responsiveness for symptoms of depression, anxiety, and trauma, the effect appears modest and should not preclude BA's application in these patients.

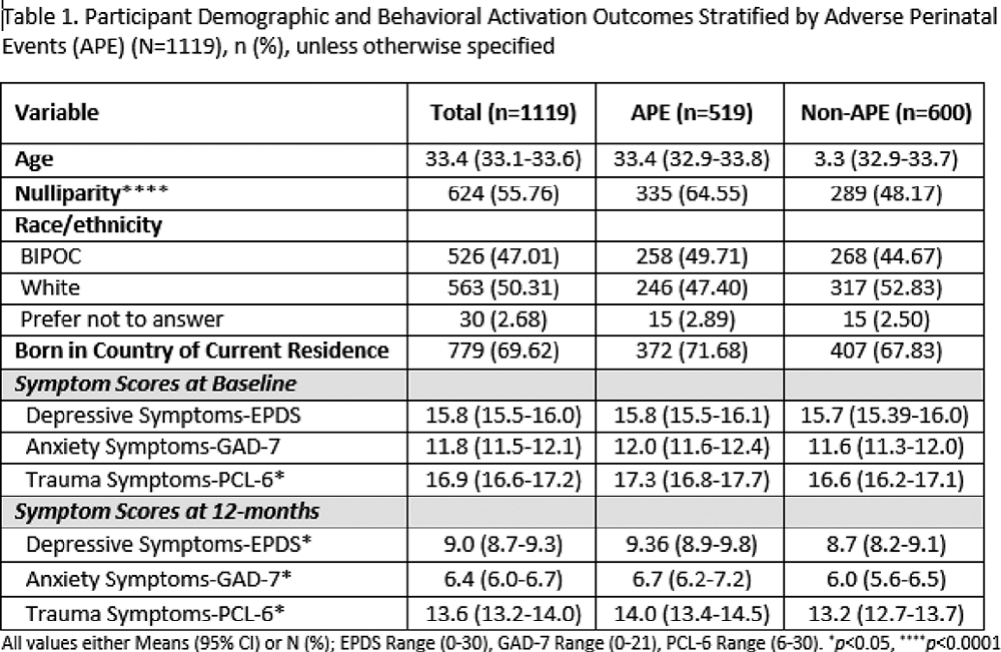

**10:30 AM – 12:00 PM**

## 926 | **Patients’ Expected Versus Experienced Discomfort With Foley Balloon Use for Induction of Labor**

Robert E. Jones^1^; Elizabeth Bloom^2^; Anna M. Modest^1^; Kimberley Campbell^1^; Sarah E. Little^3^

^1^Beth Israel Deaconess Medical Center, Boston, MA; ^2^Beth Israel Deaconess Medical Center, BOSTON, MA; ^3^Beth Israel Deaconess Medical Center, Newton, MA

**Objective**: Patients often defer or decline the use of a foley balloon (FB) for induction of labor (IOL) due to concern for significant discomfort. This study compares patients’ expected versus experienced discomfort with FB use for IOL.

**Study Design**: Patients ≥ 18 years‐old presenting for IOL with intact membranes were surveyed at the start of induction to assess expected discomfort (0–10 pain scale) with FB placement and while the FB remains in place. Patients who underwent FB placement and completed the initial survey completed a follow‐up survey after FB removal or unsuccessful placement to assess experienced discomfort. Discomfort is presented as mean and standard deviation.

**Results**: Over a 2‐month period, there 366 inductions and 183 (50%) completed the initial survey. 92 (50.3%) completed the initial survey underwent FB placement and 76 (82.6%) of these completed the follow‐up survey (Table 1). Four patients received neuraxial anesthesia prior to FB placement and were excluded. There was no significant difference between experienced and expected discomfort during FB placement (5.8 ± 2.7 vs. 6.2 ± 2.2, *p* = 0.22). However, patients experienced less discomfort than expected while the FB remained in place (5.1 ± 2.8 vs. 5.8 ± 2.1, *p* = 0.02). Hydromorphone use (0.5 mg IV and 1.0 mg subcutaneous) prior to FB placement was associated with a significant reduction in the experienced versus expected discomfort while the FB remained in place (4.5 ± 2.6 vs. 6.6 ± 2.2, *p* = 0.01) but not during placement (6.5 ± 2.8 vs. 7.0 ± 2.6, *p* = 0.45). Among those not receiving hydromorphone, no differences were observed between experienced and expected discomfort for FB placement (5.5 ± 2.6 vs. 5.8 ± 1.9, *p* = 0.34) or while in place (5.3 ± 2.9 vs. 5.4 ± 2.0, *p* = 0.45).

**Conclusion**: Patients experience less discomfort than expected while the FB remains in place, but not during placement. This reduction appears to be mediated by hydromorphone. Patients expecting significant discomfort while a FB remains in place benefit from the use of hydromorphone.

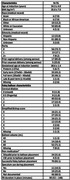

**10:30 AM – 12:00 PM**

## 927 | Predicting **Delivery for Patients in Threatened Preterm Labor by Counting Synchronized Contractions**

Roger C. Young^1^; Perry Barrilleaux^2^; Jennifer B. Gilner^3^

^1^PreTeL, Inc, PORTLAND, OR; ^2^Ochsner LSU Health Schreveport, Schreveport, LA; ^3^Duke University School of Medicine, Durham, NC

**Objective**: For patients in threatened preterm labor, current methods for diagnosing preterm birth (PTB) within 7 days are inadequate. Preterm contractions vary in strength, and predicting PTB based on expression of only strong contractions may improve sensitivity. New analytic methods, combined with recording local uterine wall electromyography (EMG) using directional sensors, can characterize each contraction as synchronized or unsynchronized and differentiate strong versus weak contractions. Additionally, the proportion of synchronized contractions may vary over short periods, so an adequate duration of evaluation is needed to reduce sampling errors. We hypothesize that a high sensitivity for predicting PTB within 7 days can be achieved by counting only synchronized contractions observed over 90 minutes.

**Study Design**: Multicenter, prospective cohort study of pregnancies between 24–36 weeks GA, with cervix ≤ 5 cm and ≥ 1 contraction in 10 min by self‐report or toco. 6‐channel recordings were obtained using PreTeL's directional uterine EMG sensors for 1.5 to 4 hours. EMG signals observed in ≥ 5 channels were classified as synchronized; frequency was the number of synchronized contractions observed over 90 minutes. Outcome was delivery < 7 days of testing. A receiver operator characteristic (ROC) was calculated to evaluate performance.

**Results**: 104 subjects were consented for study; 78 provided EMG data without being lost to follow‐up or clinical interventions such as discharge before completion of data acquisition. The ROC revealed an AUC of 0.77. For a threshold set at 75% specificity (the approximate specificity of current methods), a sensitivity of 80% for predicting PTB within 7 days was observed.

**Conclusion**: Counting synchronized contractions over 90 minutes correctly identifies 4 out of 5 patients in threatened preterm labor who will deliver within 7 days, without increasing the number incorrectly identified above current methods. This technology may improve antenatal corticosteroid administration and guide hospitalization recommendations for this population.

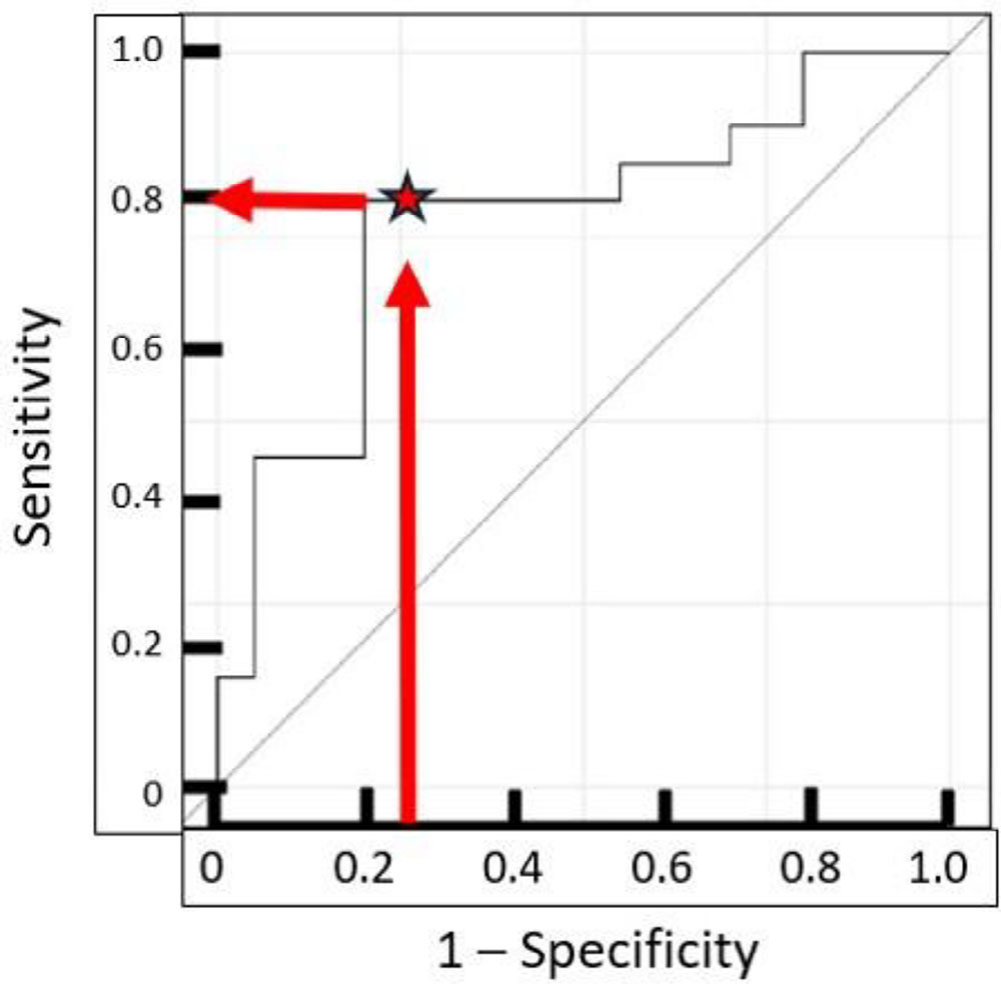

**10:30 AM – 12:00 PM**

## 929 | **Mode of Delivery in United States stillborn Pregnancies‐ a Trend Towards Cesarean Delivery**

Ronald M. Ramus^1^; Alison Verster^2^; Joshua Brunton^2^; Edward H. Springel^2^

^1^Virginia Commonwealth University, VCU Medical Center, VA; ^2^Virginia Commonwealth University, Richmond, VA

**Objective**: In the United States, the rate of Cesarean delivery (CD) has been increasing and is now the mode of delivery for over 30% of all pregnancies. Many of these Cesarean deliveries are performed for fetal indications, which is not a factor when delivering a stillborn fetus. The aim of this study was to determine if this trend is also seen amongst deliveries complicated by stillbirth. This analysis was conducted to understand current trends in stillbirth mode of delivery.

**Study Design**: We conducted a retrospective cohort study using U.S. Vital Statistics Fetal Death data from 2014 to 2023. We included singleton births at >20 weeks' gestation with birthweight >300 grams that had data on mode of delivery. We determined the rate of CD for each year and used multivariate logistic regression to adjust for factors (maternal age, race, prior CD, gestational age, birthweight, hypertension, BMI, diabetes) that may contribute to the change in CD rates in this period.

**Results**: Among the 167, 891 participants that met study criteria, 139, 728 delivered vaginally and 28, 163 delivered via CD (rate of 16.8%). We noted a linear increase in crude CD rate for stillborn pregnancies over the period analyzed (*p* = 0.001). In our unadjusted logistic model, CD was associated with increasing year (OR 1.015, 95% CI 1.01–1.02, *p* < 0.001) with an overall relative increase in CD of 11.8%. In the adjusted model, however, the year of delivery was not significant in predicting mode of delivery (*p* = 0.424). Increasing maternal age, gestational age, birthweight, and BMI were each associated with higher odds of CD, as were race, prior CD, and maternal hypertension or diabetes in the adjusted model (*p* < 0.001).

**Conclusion**: Though vaginal delivery remains the recommended mode of delivery for stillborn pregnancies, the rate of CD for stillborn pregnancies has increased over time. This analysis suggests changes in pregnancy demographics and obstetric complications over time provides a potential explanation for this increase. Further research is needed to determine clinical factors and practice patterns that are contributing to this trend.

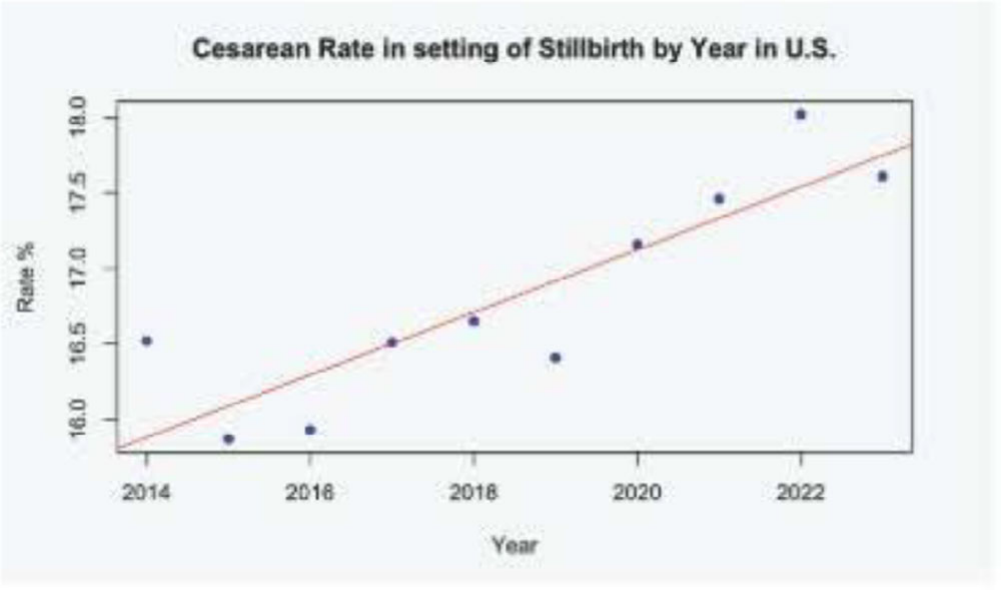

**10:30 AM – 12:00 PM**

## 930 | **Working 9–5: Adverse Perinatal Outcomes by Employment Status in a National Cohort Over 11 Years**

Ronan Daly^1^; Elizabeth Tunney^2^; Zara Molphy^3^; Patrick Dicker^4^; Fergal D. Malone^5^

^1^Royal College of Surgeons Ireland – Rotunda Hospital, Dublin, Dublin; ^2^Royal College of Surgeons in Ireland, Royal College of Surgeons in Ireland, Dublin; ^3^Department of Obstetrics and Gynaecology, Royal College of Surgeons in Ireland, Dublin, Dublin; ^4^Department of Obstetrics and Gynaecology, Royal College of Surgeons in Ireland, Dublin, Ireland., Royal College of Surgeons in Ireland, Dublin; ^5^Department of Obstetrics and Gynaecology, Royal College of Surgeons in Ireland, Dublin, Ireland., Dublin, Dublin

**Objective**: Social determinants of health are widely recognized to influence health outcomes. Our objectives were to analyze the rates of adverse perinatal outcomes and assess the influence of employment status in pregnancy.

**Study Design**: Using data from the Irish National Perinatal Reporting System, key perinatal indicators including stillbirth (death of a fetus weighing ≥500 grams), early neonatal mortality (ENM, death of a liveborn infant during the first week of life) and adjusted perinatal mortality (APM, stillbirths and early neonatal deaths excluding causative congenital anomalies) were assessed in a large retrospective cohort from 2012 to 2022. Outcome rates were analyzed according to maternal employment status: employed outside the home, unemployed, or homemaker. Logistic regression analysis was used to adjust for changes over time.

**Results**: A total of 694, 234 pregnancies were analyzed, representing 100% of births in Ireland over 11 years. Stillbirths occurred in 2, 606 pregnancies (0.004%) and early neonatal deaths occurred in 1, 385 pregnancies (0.002%). Employment data were available for 679, 820 (97.9%) women, with 526, 885 women employed outside the home (76%). 5% of women were unemployed and 17% worked as homemakers (*n* = 31, 860, *n* = 121, 075 respectively). Rates of adverse outcomes were higher in the unemployed and homemaker groups than the employed group (Figure 1). Homemakers had the highest rates of ENM and APM, over unemployed (*p* = 0.0302, *p* = 0.0431 respectively) and employed mothers (*p* < 0.0001 in both). There was no evidence of a difference in the stillbirth rates with greater variability (*p* >0.05).

**Conclusion**: Women working in the home were more likely to experience adverse perinatal outcomes when compared to those working outside the home or those unemployed. Potential contributing factors include logistical barriers such as childcare, transportation and scheduling, in addition to socio‐economic factors. This study highlights the need for further investigation of underlying factors of maternal employment status that may contribute to the adverse pregnancy outcomes in full‐time mothers.

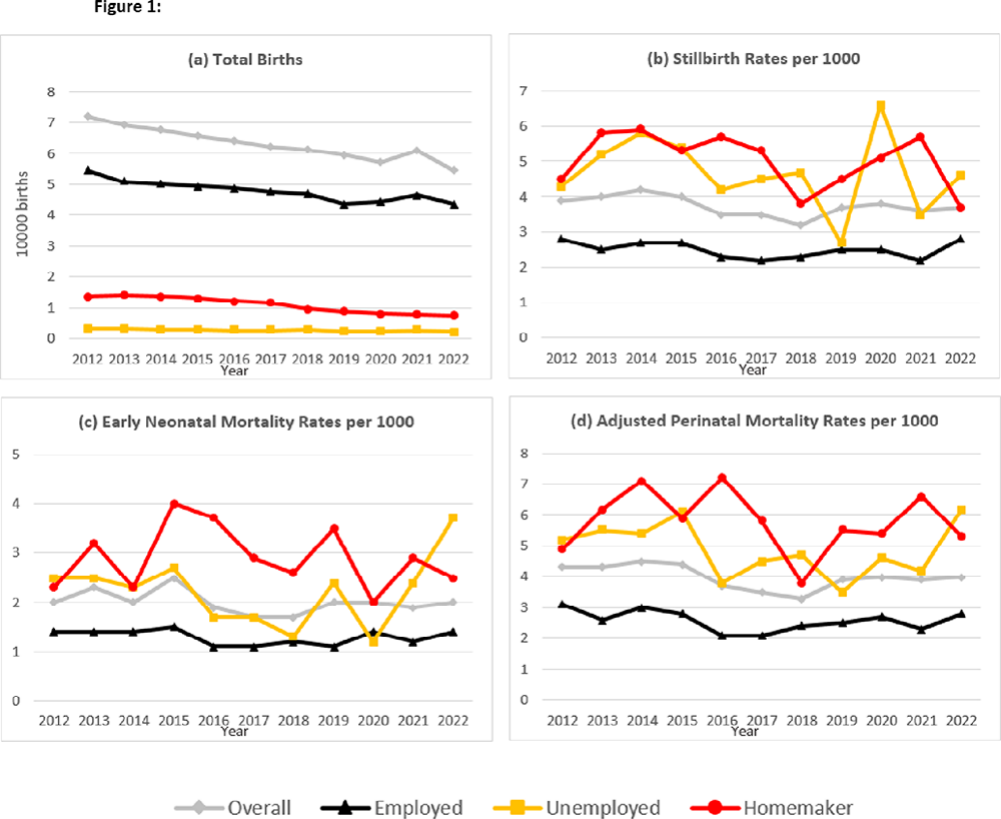

**10:30 AM – 12:00 PM**

## 931 | Preoperative **Predictors and Risk Stratification of Blood Transfusion at Time of Cesarean Delivery**

Rosa Drummond^1^; Jane Quackenbush^2^; Lauren Bernard^2^; Yasmin Hasbini^1^; Katharine Zhu^2^; Allison Lankford^1^

^1^University of Maryland Medical Center, Baltimore, MD; ^2^University of Maryland School of Medicine, Baltimore, MD

**Objective**: To evaluate preoperative factors and develop a risk stratification tool to predict the need for blood transfusion (BT) at time of cesarean delivery (CD.)

**Study Design**: This was a retrospective cohort study of 3, 268 CDs at one university medical center between 2018–2022. Cases were excluded for placenta accreta spectrum (*n* = 146), nonviable fetus (*n* = 43), or delivery outside of L&D (*n* = 46). Variables significant in the univariate analysis were analyzed with multiple logistic regression. Regression coefficients, adjusted odds ratios, and 95% confidence intervals were computed for each variable. A three‐part scoring system was derived by assigning points based on the magnitude of the regression coefficients.

**Results**: There were 114 BTs out of 3, 033 CDs (3.8%.) In the multiple logistic regression model, factors significantly associated with BT included placenta previa (aOR 5.4 [95% CI 2.2–13.2]), history of BT (aOR 4.2 [95% CI 2.6–6.7]), preoperative hemoglobin < 11 (aOR 3.1 [95% CI 2.0–4.7]), suspected intraamniotic infection (aOR 2.4 [95% CI 1.2–5.1]), dilation ≥ 6cm (aOR 2.4 [95% CI 1.5–3.9]), fibroids (aOR 2.2 [95% CI 1.3–3.7]), vaginal bleeding (aOR 2 [95% CI 1.1–3.8]), multifetal gestation (aOR 1.8 [95% CI 1–3.9.]) ROC curve produced an AUC of 0.74, *p* < 0.0001. A risk score created from weighted regression coefficients was categorized into risk groups: low risk (< 5% risk, 0–2 points), medium risk (5–9.99% risk, 3 points), and high risk (>10% risk, ≥4 points.) ROC curve of the risk score produced an AUC of 0.74, *p* < 0.0001, sensitivity 56%, specificity 79%. The actual rate and predicted probabilities of transfusions in the low risk group were 2.1% and 2.1%, in the medium risk group 5.4% and 5.8%, and in the high risk group 15% and 15.1%.

**Conclusion**: Multiple preoperative risk factors are associated with intraoperative BT. This tool can be used to reduce unnecessary preparation of crossmatched blood and improve blood utilization during CD. Recommend crossmatched blood be available in the operating room for high or medium risk patients. A type and screen may be permissible for patients categorized as low risk.

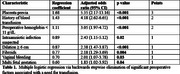

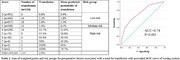

**10:30 AM – 12:00 PM**

## 932 | **Changes** in **Maternal and Infant Healthcare Utilization Following Armed Conflict: A Nationwide Retrospective Cohort Study**

Roy Bitan^1^; Racheli Magnezi^2^; Roni Brin^1^; Uri Amikam^3^

^1^Lis Hospital for Women's Health, Tel Aviv, Tel Aviv; ^2^Department of Management, Health Systems Management Program, Bar Ilan University, Ramat Gan, Israel, Ramat Gan, HaMerkaz; ^3^Lis Hospital for Women's Health, Tel Aviv Sourasky Medical Center, Tel Aviv, Tel Aviv

**Objective**: To assess the impact of armed conflict on maternal and neonatal healthcare utilization in the postpartum period.

**Study Design**: A retrospective cohort study analyzing maternal and neonatal healthcare utilization in the postpartum period was conducted using data from nine university‐affiliated hospitals between October 7, 2022, and April 7, 2024. In October 2023, an armed conflict commenced in Israel. The cohort was divided into two groups: the conflict‐exposed group (women who delivered during the six months following October 7, 2023) and the control group (women who delivered during the same calendar period in the preceding year). Data extracted from electronic medical records included maternal demographics, comorbidities, and detailed information on postpartum healthcare utilization, including hospitalizations, emergency department visits, and outpatient consultations for both mothers and their newborns.

**Results**: The study included 29, 395 deliveries, 14, 616 in the conflict‐exposed group and 14, 779 in the control group. No significant differences were observed between the groups in maternal demographics or preexisting comorbidities. Compared to the control group, women in the conflict‐exposed group had shorter postpartum hospitalizations (mean 3.1 ± 2.2 vs. 3.3 ± 2.2 days, *p* < 0.001), fewer postpartum gynecologic emergency department visits (0.2 ± 0.7 vs. 0.8 ± 0.8, *p* < 0.001), and their infants had shorter neonatal hospitalizations (2.6 ± 2.9 vs. 2.8 ± 3.2 days, *p* < 0.001) as well as fewer pediatric outpatient visits (6.3 ± 8.6 vs. 7.5 ± 9.8, *p* < 0.001). No significant differences were found in maternal or neonatal hospital readmissions.

**Conclusion**: Exposure to armed conflict was associated with decreased utilization of postpartum healthcare services and shorter hospital stays for mothers and newborns. These findings may reflect limited access to care, shifting healthcare‐seeking behavior, or systemic adaptations under emergency conditions. Further investigation is needed to assess the long‐term implications of reduced postnatal follow‐up in conflict settings and to inform future policy planning.

**10:30 AM – 12:00 PM**

## 933 | Obstetrical **Workforce Density and Perinatal Mortality Risks in the United States**

Ruby Lin^1^; Cande V. Ananth^2^; Todd J. Rosen^3^; Rachel Lee^2^

^1^Kaiser Permanente Northern California, Kaiser Permanente Northern California, CA; ^2^Rutgers Robert Wood Johnson Medical School, Rutgers Robert Wood Johnson Medical School, NJ; ^3^Jersey City Medical Center, Jersey City Medical Center, NJ

**Objective**: With the recent focus on maternity care deserts, examining obstetric workforce density is extremely relevant. We hypothesized that a higher density of obstetrics and gynecology (OBGYN) physicians was associated with lower perinatal mortality.

**Study Design**: Using the United States live birth, infant death, and fetal death data (2016 to 2020), this was a cross‐sectional study of full‐term, singleton, cephalic, and non‐anomalous deliveries. County‐level OBGYN physician density was estimated as the number of OBGYN physicians per 1, 000 deliveries using the Area Health Resource Files and categorized into quintiles. Perinatal mortality included term stillbirth and neonatal death at ≤28 days. Confounder‐adjusted relative risks (RR) were calculated using Poisson regression models with robust variance to assess the association between OBGYN density and perinatal mortality. Planned subgroup analyses were conducted to assess the association stratified by race/ethnicity, social vulnerability index, rural‐urban county, county birth volume, and state abortion restrictions.

**Results**: There were 16, 094, 208 full‐term, singleton, cephalic, and non‐anomalous deliveries. Perinatal mortality rates for quintiles 1 (lowest density) through 5 (highest density) were 2.4, 2.5, 2.3, 2.3, and 1.9 per 1000 births, respectively. Compared to quintile 5, the RR of perinatal mortality for quintiles 1 through 4 was 1.15 (95% CI 1.11–1.19), 1.19 (95% CI 1.16–1.23), 1.17 (95% CI 1.13–1.20), and 1.12 (95% CI 1.08–1.16), respectively (Figure). The highest quintile density was associated with the lowest risk of perinatal mortality regardless of determinants of health (Table). The exception was in counties with a high‐risk social vulnerability index‐ increasing OBGYN density was not associated with decreasing perinatal mortality.

**Conclusion**: Higher density of OBGYN physicians was associated with a lower risk of perinatal mortality, except in socially vulnerable counties. In addition to increasing access to obstetric care, this study underscores how social determinants shape perinatal mortality risks.

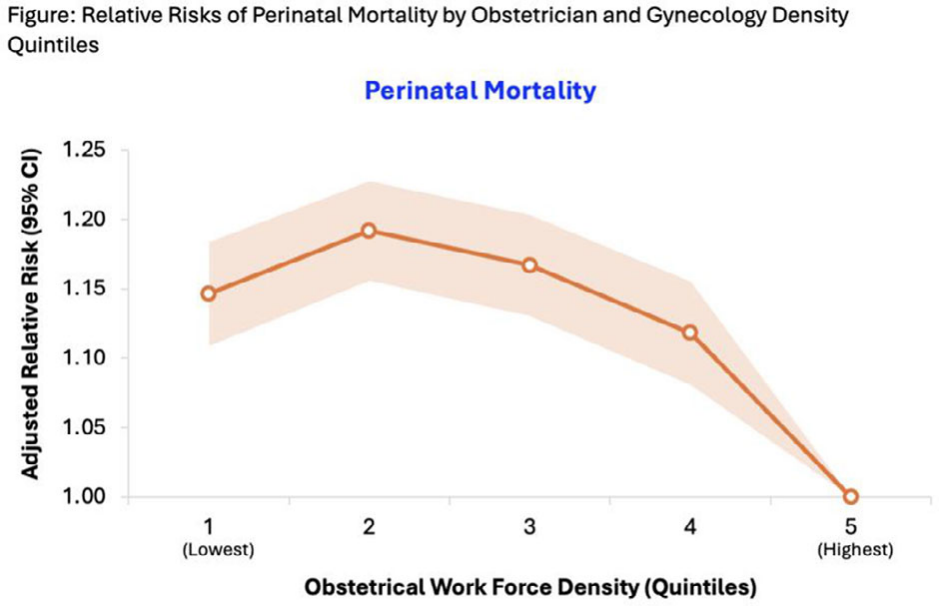

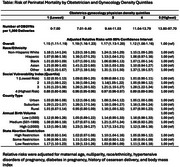

**10:30 AM – 12:00 PM**

## 934 | Association **Between Social Determinants of Health, Neighborhood Indices, and Preterm Birth: A Prospective Study**

Rula Atwani^1^; Alyssa Wilkinson^2^; Katherine Mclaughlan^2^; Danielle Long^2^; Andrea Hooberman‐Pineiro^2^; Tori Johnson^3^; Alyssa Jackson^2^; George R. Saade^1^; Liyun Wu^3^; Grace Spencer^4^; Tetsuya Kawakita^5^

^1^Eastern Virginia Medical School at Old Dominion University, Norfolk, VA; ^2^Macon & Joan Brock Virginia Health Sciences at Old Dominion University, Norfolk, VA; ^3^Norfolk State University, Norfolk, VA; ^4^Eastern Virginia Medical School at Old Dominion University, EVMS OBGYN, Virginia Health Sciences at Old Dominion University, VA; ^5^Eastern Virginia Medical School Department of Obstetrics and Gynecology, Macon & Joan Brock Virginia Health Sciences at Old Dominion University, Norfolk, VA

**Objective**: To evaluate the association between preterm birth and social determinants of health (SDoH) and neighborhood‐level socioeconomic indices.

**Study Design**: This was a nested case‐control prospective study conducted at a tertiary care hospital from February 2024 to July 2025. We limited analysis to individuals aged 18–50 years with singleton pregnancies, gestational age >20 weeks, and livebirth. Participants with preterm births (<37 weeks) and those with term births were enrolled in a 1:3 ratio. Participants completed the Accountable Health Communities Health‐Related Social Needs screening tool, assessing core needs (housing instability, food insecurity, transportation, utility needs, and interpersonal safety) and supplemental needs (financial strain, employment, support, education, physical activity, substance use, mental health, and disability). Neighborhood indices, including Maternal Vulnerability Index (MVI), Area Deprivation Index (ADI), Walkability Index, and Food Desert status, were retrieved using individuals’ home addresses. Relative risks (RR) for SDoH variables were estimated using modified Poisson regression, adjusting for BMI, chronic hypertension (CHTN), and pregestational diabetes (PDM). Neighborhood indices were analyzed using bivariate methods with significance set at *p* < 0.05.

**Results**: We included 280 participants, 209 delivered at term and 71 delivered preterm. Demographic and clinical characteristics were similar between groups except for CHTN (35.2% vs 12.4%, *p* = 0.001), PDM (19.7% vs 6.2%, *p* = 0.001), and gestational diabetes (19.7% vs 9.6%, *p* = 0.024), which were significantly more frequent in the preterm group (Table 1). Compared to the preterm group, the term group had significantly higher substance use (9.9% vs 23.4%, *p* = 0.013). There was no significant difference in core SDoH needs (23.9% vs. 35.9%; *p* = 0.06), supplemental SDoH needs (91.5% vs. 94.7%; *p* = 0.05), or neighborhood indices (Table 2).

**Conclusion**: SDoH and neighborhood indices were not associated with preterm birth at a tertiary care hospital. This may reflect the dominant influence of medical risk factors in this population.

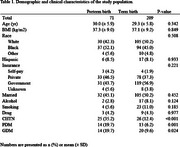

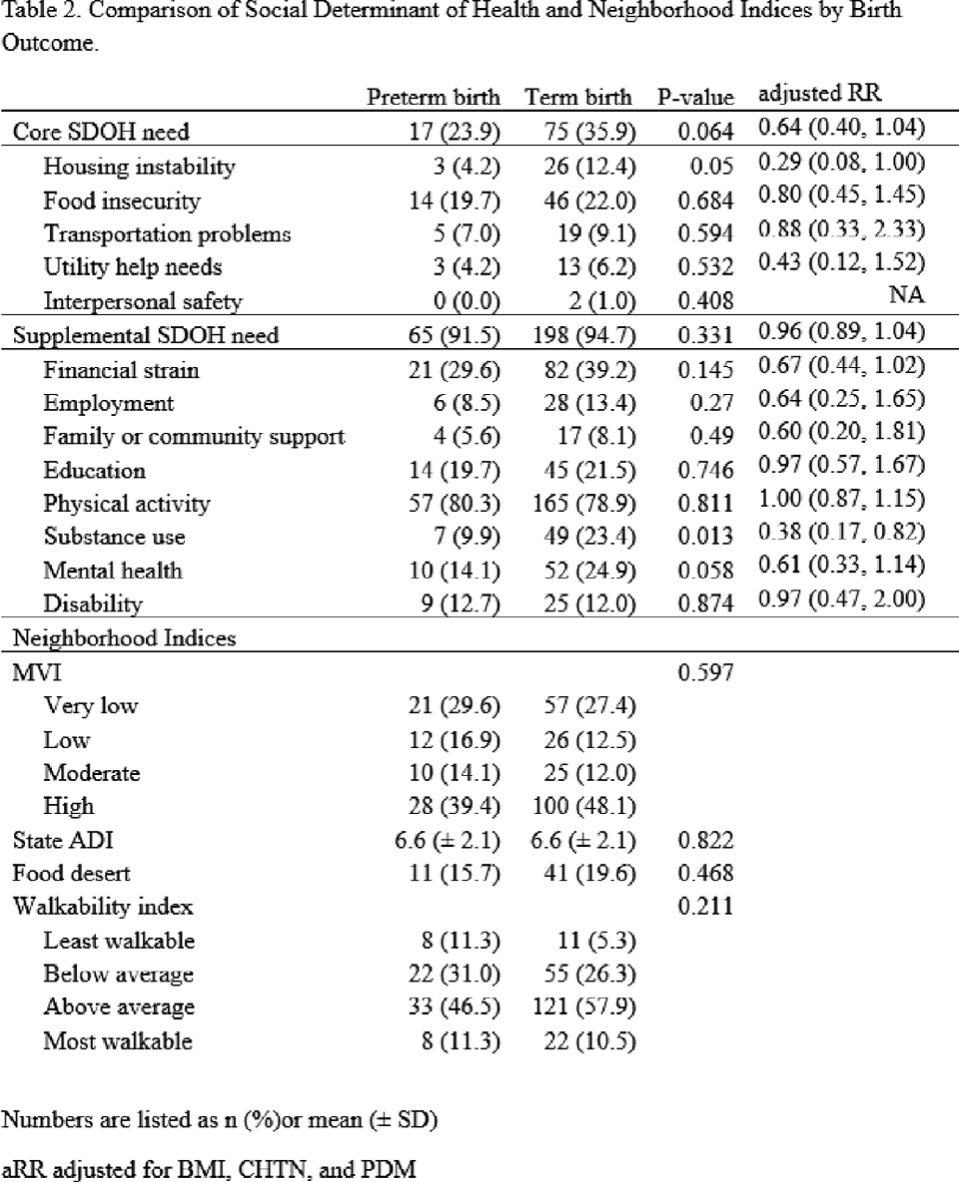

**10:30 AM – 12:00 PM**

## 935 | **Machine Learning Based Risk Stratification for Postpartum Hemorrhage: Insights From a National Dataset**

Sage Smith^1^; Ashley Zimmermann^2^; Mariah Buzzell^3^; Angela Kown^1^; Yuzuru Anzai^2^

^1^University of Chicago, Chicago, IL; ^2^Northwell, New Hyde Park, NY; ^3^Northwell, Northwell Lenox Hill Hospital, NY

**Objective**: The rate of postpartum hemorrhage (PPH), defined as blood loss of ≥1, 000mL, has increased over the last decade and remains the leading cause of maternal mortality. This study aims to identify maternal and obstetric factors associated with increased risk of PPH.

**Study Design**: Maternal and obstetric data from the CDC Natality dataset spanning 2021 to 2023 were analyzed. Deliveries complicated by PPH were identified based on the administration of blood transfusion with or without ICU admission. Clinically relevant variables such as maternal demographic information as well as obstetrical data were selected. Two different models were developed to predict PPH risk – a decision tree classifier model and a logistic regression model. Performance of these two models was evaluated using sensitivity, specificity, and area under the receiver operating curve (AUC).

**Results**: With >95% power from our large cohort, the decision tree classifier model performed with an AUC of 0.83 (sensitivity 72.5%, specificity 77.9%, PPV 76.7%, NPV 73.9%), while logistic regression yielded an AUC of 0.80 (sensitivity 73.6%, specificity 71.5%, PPV 72.1%, NPV 72.9%). In the decision tree, delivery method was one of the strongest predictors of PPH, with risk decreasing in the following order: vaginal delivery after prior cesarean, primary cesarean, repeat cesarean, vaginal delivery without prior cesarean. Labor induction and prior cesarean delivery were also major contributors. Eclampsia was a negligible predictor, likely due to its low prevalence and overlap with other hypertensive variables. Regression analysis highlighted previous cesarean delivery, chorioamnionitis, and pre‐pregnancy diabetes as the strongest predictors.

**Conclusion**: Both models performed similarly and demonstrated good discrimination for identifying individuals at risk of PPH. Early identification of individuals at risk of PPH is critical to improve outcomes and guide preventative interventions.

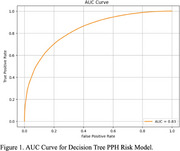

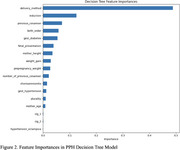

**10:30 AM – 12:00 PM**

## 936 | Intermittent **Subcutaneous Insulin Versus Insulin Pump in Managing Patients With Type 2 Diabetes in Pregnancy**

Salimah Navaz Gangji^1^; Katherine Pepper^2^; Jillian W. Jetmore^2^; Erkan Kalafat^3^; Rebecca Horgan^2^; Jerri Waller^2^; George R. Saade^2^; Marwan Ma'ayeh^2^

^1^Eastern Virginia Medial School at Old Dominion University, Norfolk, VA; ^2^Eastern Virginia Medical School at Old Dominion University, Norfolk, VA; ^3^Koc University Hospital, Istanbul, Koc University Hospital, Istanbul, Istanbul

**Objective**: To compare glycemic control in pregnant patients with Type 2 diabetes (T2DM) managed with intermittent subcutaneous insulin (ISI) therapy versus insulin pump.

**Study Design**: This was a retrospective cohort study of all pregnant individuals with T2DM on insulin therapy and using a continuous glucose monitor (CGM) between 2016–2025. We compared third‐trimester time in range (TIR; % time with a blood sugar between 60–140 mg/dL) and glucose level variance between insulin pump and ISI using the Wilcoxon rank‐sum test. The analysis was stratified by the timing of pump initiation (first, second, or third trimester). A multivariable multinomial logistic regression model was used to assess the association between the timing of pump initiation and third‐trimester TIR categories (≤50%, >50–75%, ≥75%), adjusting for first‐trimester HbA1c, maternal age, BMI, ethnicity, and chronic hypertension.

**Results**: Of the 125 patients in the cohort, 59 were on insulin pump therapy. The median TIR for all pump users was 70.0% (IQR 56.5–80.5) compared to 68.0% (IQR 50.2–79.5) for ISI (*p* = 0.48). This lack of a significant difference persisted when restricting the analysis to those initiating pump therapy in the second or first trimester (*p* = 0.48) and first trimester only (*p* = 0.51). However, individuals who initiated pump therapy in the first trimester had a significantly lower blood glucose level variance compared to ISI (30.5 mg/dL vs. 35.5 mg/dL, *p* = 0.04). In the multivariable analysis, the timing of insulin pump initiation was not significantly associated with better TIR categories. The only significant association with poor glycemic control (TIR ≤50%) was a higher first‐trimester HbA1c (aOR 1.47, 95% CI 1.10–1.98; *p* = 0.01).

**Conclusion**: In pregnant individuals with T2DM, insulin pump use was not associated with better glycemic control compared with ISI. However, initiation of pump therapy in the first trimester was associated with significantly lower glycemic variability. Baseline glycemic status, indicated by first‐trimester HbA1c, remains the most significant determinant of achieving TIR targets.

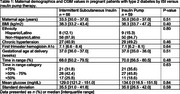

**10:30 AM – 12:00 PM**

## 937 | **Is** Interleukin**‐1 Alpha a Key Mediator of Perinatal Neuroinflammation Associated With Preterm Birth?**

Samantha Ocampo^1^; Morgan E. Wasickanin^2^; Hillary Kinsman^3^; Katherine Free^3^; Jennifer Damicis^3^; Robert Walton^3^; Peter Napolitano^4^; Nicholas Ieronimakis^3^

^1^Oregon Health & Science University, Portland, OR; ^2^Madigan Army Medical Center, Fort Bragg, NC; ^3^Madigan Army Medical Center, Tacoma, WA; ^4^University of Washington, Seattle, WA

**Objective**: Perinatal white matter injury results from neuroinflammation mediated primarily by interleukin 1 (IL‐1) signaling via the cytokines IL‐1a and IL‐1B. The role of IL‐1B in brain injury is extensively characterized yet treatments targeting IL‐1B have proven ineffective. Little is known regarding the function and therapeutic potential of IL‐1a. We aimed to examine the role of IL‐1a in an established modality of perinatal neuroinflammation using a mouse model where *Il1a* is deleted.

**Study Design**: *Il1a* knockout (KO) and wild type (wt) dams received an intrauterine injection of bacterial endotoxin (LPS) to induce fetal neuroinflammation or the vehicle (PBS) as a control, at embryonic day 15 of the 19–21 days gestation. Fetal brains and placentas were isolated at the peak of inflammation, 6‐hours following intrauterine injections (Figure 1A) and analyzed for the expression of inflammatory receptors and cytokines; *Tlr4*, *Il1r1*, *Il1a*, *Il1b*, *Il6* and *Tnf*.

**Results**: Inflammatory receptors were upregulated by LPS exposure mainly in placentas, with exception to *Tlr4* which varied in feta brains by condition and genotype (Figure 1B). Cytokines were significantly upregulated in wild type embryo brains that received LPS vs. PBS (Figure 2). In KO brains, *Il1b* and *Il6* were also upregulated with LPS. With LPS exposure the expression of *Tnf* in KO brains was significantly lower than wild type and similar to PBS controls. In placentas, cytokines were equally upregulated with LPS in both KO and wild type. No *Il1a* transcript was detected in KO tissues.

**Conclusion**: Similarities in *Il1r1* between genotypes suggest that interleukin‐1 receptor signaling was not altered by absence of *Il1a* in KO animals. Results suggest that in fetal brains IL‐1a plays a significant role in Tnf production, a mediator of tissue injury and cell death. This appears specific to neuroinflammation being no differences were observed in placentas. Further research is needed to fully characterize the role and therapeutic potential of targeting IL‐1a within the setting of perinatal neuroinflammation.

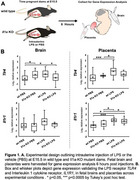

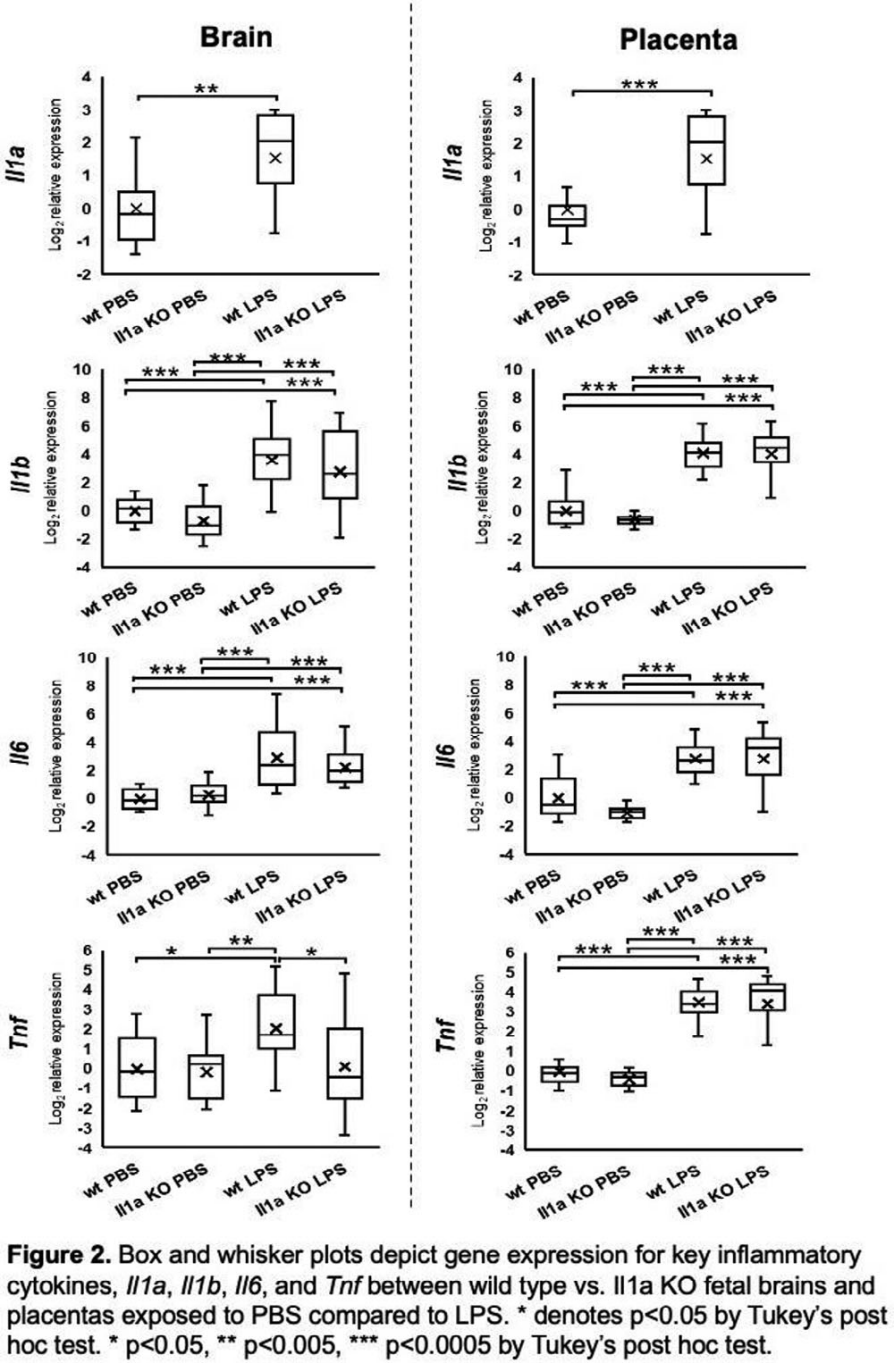

**10:30 AM – 12:00 PM**

## 938 | Ultrasound **to Predict Congenital Syphilis Risk Despite BPG: A Tool, not a Management Strategy**

Sana Jaleel^1^; Heath Yancey^1^; Abigail B. Clark^2^; Shreya Battu^1^; Hiselva Moriyon^3^; Jessica E. Pruszynski^1^; Emily H. Adhikari^1^

^1^University of Texas Southwestern Medical Center, Dallas, TX; ^2^University of Texas Southwestern Medical Center, University of Texas Southwestern Medical Center, TX; ^3^Parkland Health, Dallas, TX

**Objective**: Characterize and compare congenital syphilis (CS) outcomes among pregnancies with and without any abnormal ultrasound (US) findings prior to initiation of benzathine penicillin G (BPG).

**Study Design**: Retrospective chart review of maternal syphilis diagnosed ≥18 weeks at a large public hospital. Among those with US for CS before BPG administration, we documented placentomegaly, polyhydramnios, hepatomegaly, ascites, and elevated MCA systolic velocity (>1.5MoM). We compared syphilis characteristics and US findings among those with and without US abnormalities, and grouped isolated versus combinations of US findings with liveborn congenital syphilis outcomes.

**Results**: From Jan 2010 through June 2025, 307 were diagnosed with syphilis ≥18 weeks; not all had US for CS findings. Of 125(40.7%) with US for CS, 119(95%) received BPG before delivery. Of those with US, 61(48.8%) had at least one finding of CS (hepatomegaly [67%]; placentomegaly [52%], MCA >1.5MoM [25%], polyhydramnios [8%], and ascites [7%]); 64(51.2%) had no abnormal US findings and most were treated in the clinic. There was no difference in maternal syphilis stage or initial RPR among those with vs without abnormal US. CS was higher among fetuses with *at least* hepatomegaly (OR 5.27, 95% CI, 2.05–14.5), but less likely if hepatomegaly was isolated or with one other finding, suggesting BPG was more likely to be effective before delivery. When multiple US abnormalities were identified in the late second or early third trimester, CS was frequent despite BPG. CS occurred in 2/2 stillborn and 2/2 of liveborn with ascites, suggesting this was a late finding and less reversible by BPG before delivery occurred.

**Conclusion**: Most ultrasounds after 18 weeks in gravidas with syphilis are normal, facilitating efficient treatment outpatient. When US demonstrates combinations of multiple CS‐associated abnormalities (particularly ascites) in the late second or early third trimester, BPG may not be successful in preventing a CS outcome before delivery occurs.

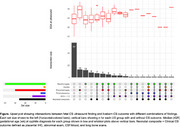

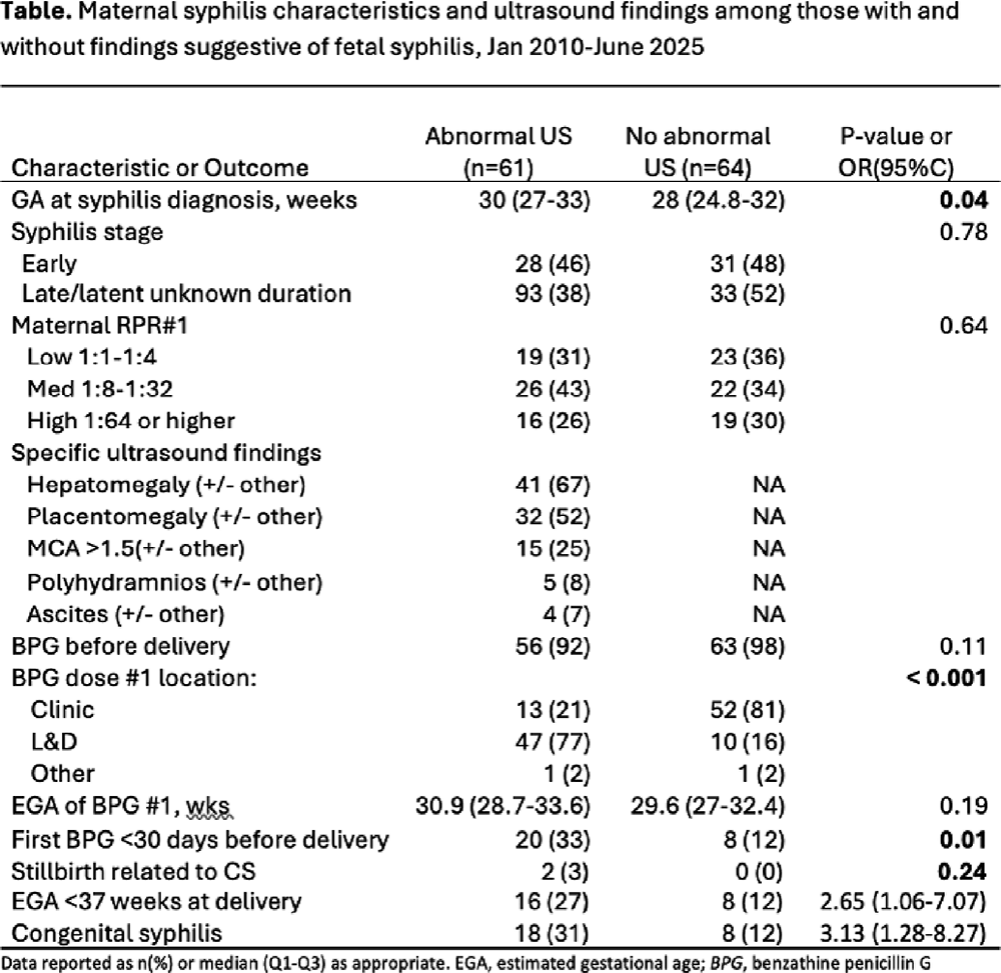

**10:30 AM – 12:00 PM**

## 939 | **Maternal Asthma and the Endocrine Health of Offspring: Insights From a Population‐Based Cohort Study**

Sapir Ellouk^1^; Tamar Wainstock^2^; Eyal Sheiner^3^

^1^Department of Obstetrics and Gynecology, Soroka University Medical Center, Ben‐Gurion University, HaDarom; ^2^Department of Epidemiology, Biostatistics and Community Health Sciences, Faculty of Health Sciences, Ben‐Gurion University of the Negev, Beer Sheva, HaDarom; ^3^Soroka University Medical Center, Faculty of Health Sciences, Ben‐Gurion University of the Negev, beer sheva, HaDarom

**Objective**: Asthma is a prevalent chronic morbidity among pregnant women and is characterized by a variable clinical course during gestation. Maternal asthma has been associated with an increased risk of significant obstetric complications. Emerging evidence suggests that its effects may extend into the postnatal period, raising concerns about potential long‐term health outcomes in offspring. This study evaluated whether maternal asthma is an independent risk factor for long‐term endocrine morbidity in the offspring.

**Study Design**: We conducted a population‐based cohort study of deliveries from 1991 to 2021 using data from community clinics and a tertiary referral hospital. The primary outcome was the incidence of endocrine morbidity in offspring of mothers with vs without asthma. Kaplan–Meier curves assessed cumulative incidence, and a Cox proportional hazards model adjusted for confounders.

**Results**: The analysis included 232, 094 deliveries, 2, 875 (1.2%) of mothers diagnosed with asthma. Offspring born to mothers with asthma had a significantly higher rate of endocrine morbidity compared to those without maternal asthma (11.2% vs. 7.2%; OR = 1.6, 95% CI 1.44–1.83, *p* < 0.001), with notably increased rates of thyroid disorders, hyperlipidemia, and obesity (Table). Kaplan‐Meier survival analysis further demonstrated a significantly greater cumulative incidence of endocrine morbidity among offspring of asthmatic mothers (*log‐rank p* < 0.001; Figure). After adjusting for maternal age, gestational diabetes, cesarean delivery, hypertension, and gestational age using a Cox proportional hazards model, maternal asthma remained an independent predictor of endocrine morbidity in offspring (adjusted HR = 1.4; 95% CI, 1.25–1.56; *p* < 0.001).

**Conclusion**: Maternal asthma is an independent risk factor for long‐term endocrine morbidity in offspring. Further research is warranted to elucidate the underlying mechanisms linking maternal morbidity to fetal programming, the uterine environment, and long‐term offspring health.

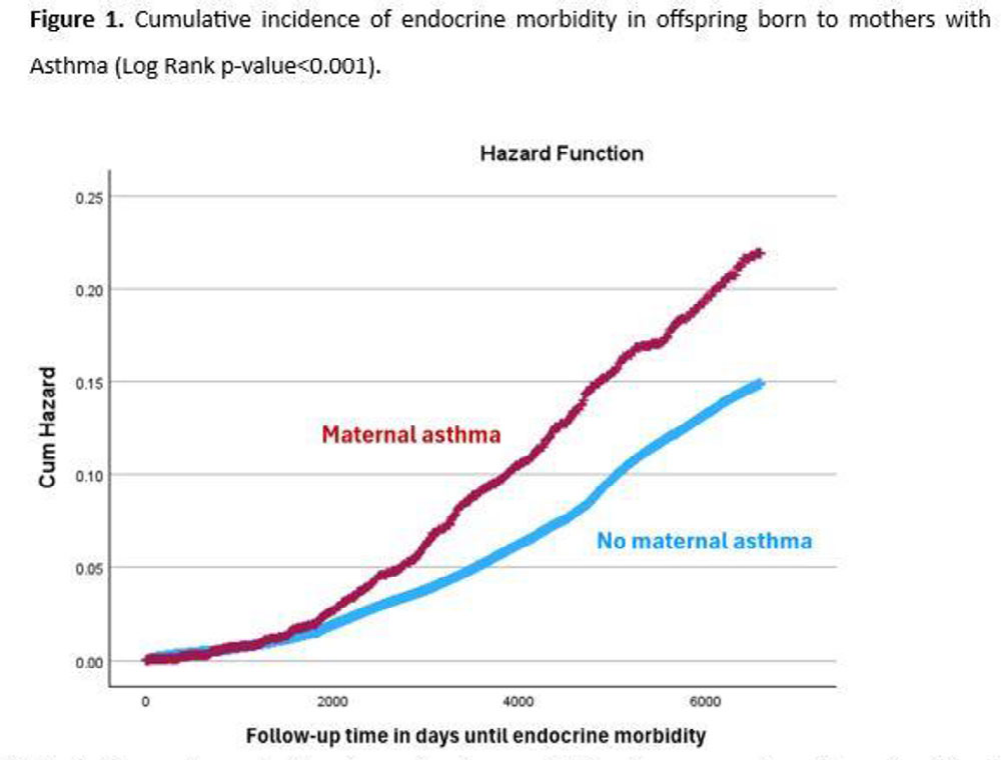

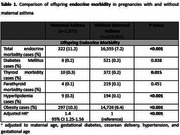

**10:30 AM – 12:00 PM**

## 940 | Neighborhood**‐Level Exposure to Gun Violence and Adverse Pregnancy Outcomes**

Sara E. Edwards^1^; Nicola F. Tavella^2^; Barbara Pereira Vera^3^; Raina Kishan^4^; Nina G. Faynshtayn^3^; Calvin E. Lambert, Jr.^1^; Kimberly Glazer^3^

^1^Division of Maternal‐Fetal Medicine, Icahn School of Medicine at Mount Sinai, New York, NY; ^2^Division of Maternal‐Fetal Medicine, Icahn School of Medicine, Icahn School of Medicine at Mount Sinai Hospital, New York, NY; ^3^Icahn School of Medicine at Mount Sinai, New York, NY; ^4^Mount Sinai Hospital, New York, NY

**Objective**: Adverse pregnancy outcomes are associated with prenatal stress. This study examined whether prenatal exposure to neighborhood‐level gun violence was associated with increased risk of adverse outcomes in a cohort of New York pregnant people.

**Study Design**: This was a retrospective study of all pregnant patients delivering at a single institution in New York City from January 1, 2013 through December 31, 2022. We excluded patients residing outside New York state. Electronic medical records were assessed for clinical and demographic data. Addresses were matched to 2020 US Census tracts and merged with data from The Gun Violence Archive (a validated national repository) to compute the number of gun violence incidents in each tract during each pregnancy. We then transformed these into rates (person‐years). Patients were categorized as 1) any prenatal gun violence exposure, and 2) low, medium, or high exposure using Jenks natural breaks. Multivariable logistic regression models controlling for social indices examined associations of exposure with adverse pregnancy outcomes.

**Results**: We included 52, 906 pregnant patients. 1, 367 (2.58%) patients were exposed to ≥1 gun violence incident during their pregnancy. When classified into risk tertiles, 51, 617 (97.56%) had low exposure, 1, 105 (2.09%) had medium exposure, and 184 (0.35%) had high exposure. Patients with greater gun violence exposure were younger, nulliparous, non‐White, had public insurance, and had an obese pre‐pregnancy BMI (Table 1). In multivariable models, patients with high compared with low gun violence exposure were at greater risk for hypertensive disorders of pregnancy (OR 1.82 [1.15, 2.88]) and placental abruption (OR 4.18 [1.15, 10.75]). Although patients with gun violence exposure delivered at earlier gestational ages (*p* < 0.001, Table 1), preterm birth was not significantly associated with exposure to gun violence (Table 2).

**Conclusion**: Neighborhood‐level gun violence exposure during pregnancy was associated with greater risk of hypertensive disorders of pregnancy and placental abruption.

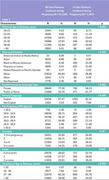

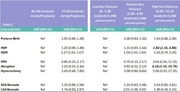

**10:30 AM – 12:00 PM**

## 941 | **Impact of a National Shortage‐Based Intravenous Fluid‐Restrictive Protocol on Delivery Outcomes**

Sara I. Jones^1^; Chun Xu^2^; Tracy Truong^2^; Osinakachukwu Mbata^3^; Lillian Boettcher^4^; Rachel L. Wood^5^; Brenna L. Hughes^6^

^1^Duke University School of Medicine, Durham, NC; ^2^Duke University Medical Center, Durham, NC; ^3^Duke University Health, Durham, NC; ^4^Department of Obstetrics and Gynecology, Duke University School of Medicine, Duke University School of Medicine/Durham, NC; ^5^Division of Maternal Fetal Medicine, Department of Obstetrics and Gynecology, Duke University, Durham, NC; ^6^Department of Obstetrics and Gynecology, Duke University School of Medicine, Duke, NC

**Objective**: Intravenous hydration is a common practice on US L&D units, though general medicine literature recommends limited use due to adverse morbidity. In the fall of 2024, a national intravenous fluid (IVF) shortage led to IVF restriction for laboring and postpartum patients; however, effects of this change are poorly understood. We aimed to assess delivery, obstetric, and neonatal outcomes associated with IVF fluid restriction.

**Study Design**: We performed a pre‐ and post‐ analysis of patients delivering at a single tertiary health system in temporal proximity to the IVF protocol change. Restrictions in IVF use at our institution related to the 2024 Baxter Fluid Shortage began October 3, 2024; this protocol included no continuous IV fluids compared to the prior standard 75ml/hr. Primary exposure was delivery after protocol implementation after a 2‐week washout (October 15 2024‐April 30 2025) compared to deliveries in a pre‐fluid restriction (October 15 2023‐April 30 2024) period. Primary outcome were delivery outcomes. Secondary outcomes were maternal complications and neonatal outcomes. Chi‐square tests and t‐tests were used for analysis.

**Results**: There were 3560 deliveries in the pre‐restriction and 3564 in the IVF‐restriction group following the washout period. There were no differences in maternal age or ethnicity between groups with a statistically but not clinically significant difference in race and payor status between groups (Table 1). There was no difference in cesarean delivery rate (37.4% versus 37.0%), intraamniotic infection (8.9% versus 8.4%), OASIS tear (2.1% vs 1.9%), or pulmonary edema (0.1% vs 0.2%) before versus during the IVF‐restrictive period (Figure 1). No differences were found in neonatal outcomes.

**Conclusion**: An IVF‐restrictive protocol utilized during a national shortage was not associated with a higher rate of cesarean delivery or adverse delivery or neonatal outcomes. This should be considered when developing strategies to minimize unnecessary interventions with potential adverse outcomes and to reduce healthcare waste.

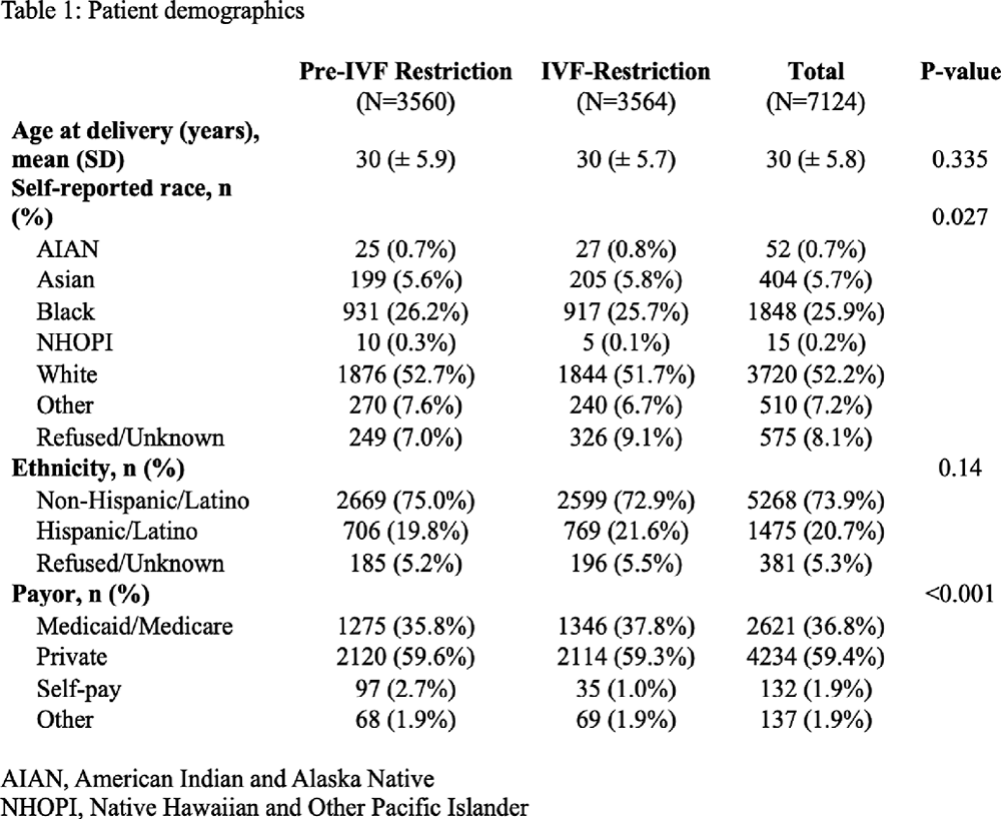

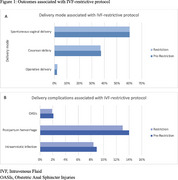

**10:30 AM – 12:00 PM**

## 942 | **Risk** Factors **and Maternal Morbidity Associated With Hysterotomy Extension at Time of Cesarean Delivery**

Sara E. Post; Amanda A. Allshouse; Torri D. Metz; Ann M. Bruno

University of Utah, Salt Lake City, UT

**Objective**: To evaluate the association between hysterotomy extension at time of cesarean delivery in low‐risk nulliparas and maternal morbidity. Secondarily, to identify risk factors for hysterotomy extension in this population.

**Study Design**: This was a secondary analysis of the MFMU ARRIVE trial, a multi‐center randomized trial of induction compared with expectant management among low‐risk nulliparas at term. Those undergoing cesarean delivery with a low transverse hysterotomy were included in this analysis. The exposure was hysterotomy extension (both intentional and unintentional). The primary outcome was a maternal morbidity composite of infection, hemorrhage and intensive care unit (ICU) admission. Secondary outcomes included components of the primary and a neonatal morbidity composite. Multivariable modeling estimated the association between hysterotomy extension and outcomes with odds ratios and 95% confidence intervals reported. Candidate risk factors for hysterotomy extension were also evaluated.

**Results**: Of 1, 234 patients included, hysterotomy extension occurred in 76 (6.2%). Baseline characteristics were similar between those with and without hysterotomy extension (Table 1). Hysterotomy extension compared with no extension was associated with maternal morbidity (34.2% vs. 19.6%, OR 2.13, 95% CI 1.30–3.50; aOR 1.8, 1.08–3.00), and all individual components of the composite. Hysterotomy extension was not associated with neonatal morbidity (23.7% vs. 21.2%, OR 1.15, 95% CI 0.67–1.99; aOR 1.05, 0.60–1.84). No statistically significant risk factors for hysterotomy extension were identified.

**Conclusion**: In a population of low‐risk nulliparas undergoing cesarean delivery, hysterotomy extension was associated with increased maternal morbidity but not neonatal morbidity. There were no statistically significant risk factors for hysterotomy extension identified. It remains unknown whether hysterotomy extension is a consequence of other clinical factors which predispose to these complications, or if it is the hysterotomy extension itself that results in morbidity.

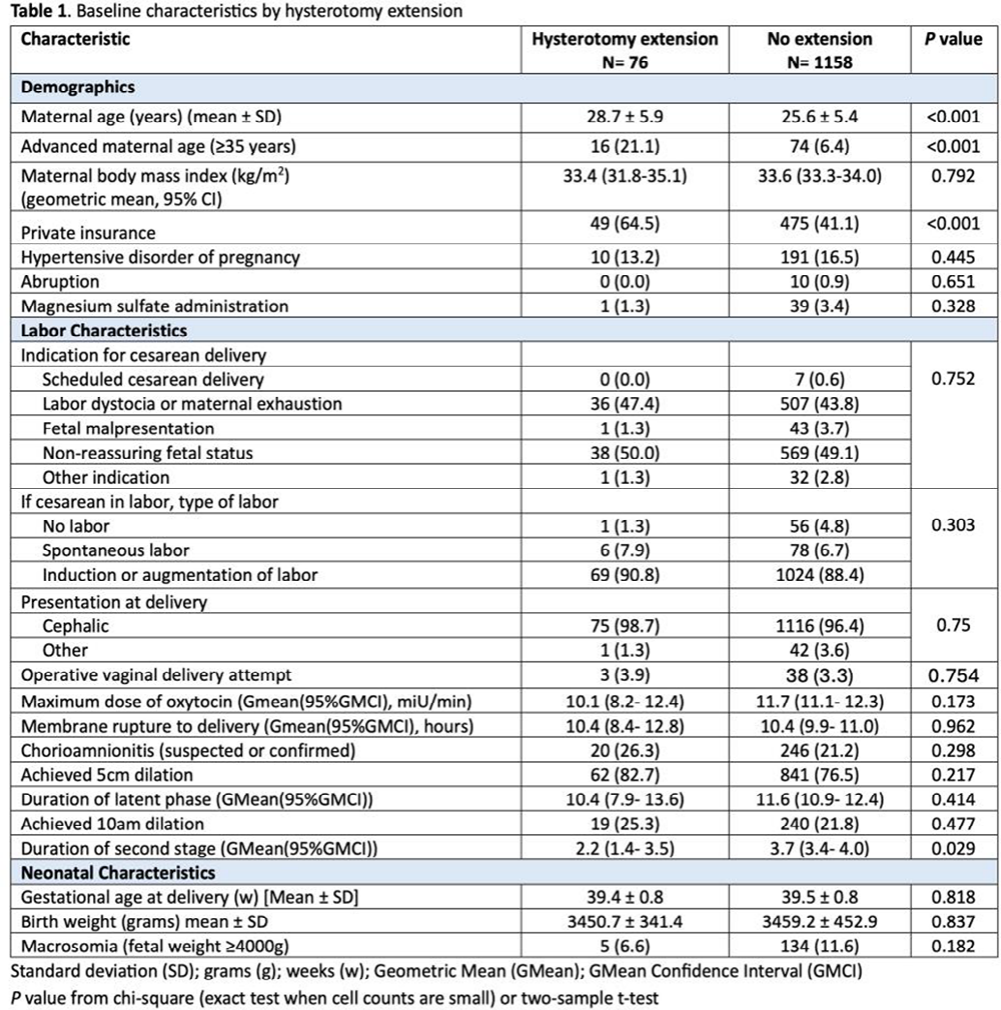

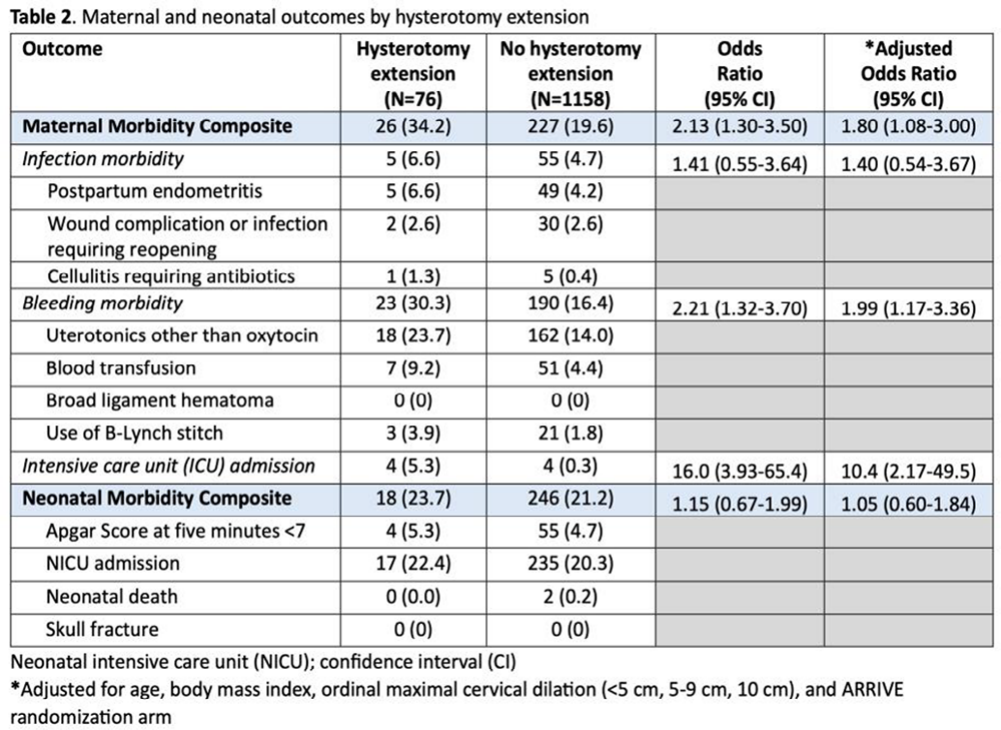

**10:30 AM – 12:00 PM**

## 943 | Association **of Autoimmune Disease With Hypertensive Disorders of Pregnancy**

Sarah H. Abelman; Isabella A. Molina; Sarah Z. Wu; Frank I. Jackson; Oladunni Ogundipe; Luis A. Bracero; Jolene C. Muscat; Matthew J. Blitz

Northwell, New Hyde Park, NY

**Objective**: Certain autoimmune diseases, like systemic lupus erythematosus, are linked with hypertensive disorders of pregnancy (HD), prompting the recommendation for prophylactic low‐dose aspirin. This study examined the association between other autoimmune disorders and HDP.

**Study Design**: This was a retrospective cohort study of all pregnant patients with live births at ≥23 weeks’ gestation from 2019–2024 in a large health system in New York. The primary exposure was autoimmune disease, limited to conditions eligible for TNF‐alpha inhibitor therapy. These conditions are characterized by chronic inflammation and immune dysregulation, which could theoretically contribute to placental dysfunction and HDP. The primary outcome was HDP, and secondary outcome was preeclampsia with severe features. A subgroup analysis was done for pregnancies complicated by inflammatory bowel disease (IBD). Multivariable logistic regression was used to assess the association between the exposure and the outcomes, adjusting for body mass index class, advanced maternal age, multiparity, insurance status, English‐speaking, and race/ethnicity. Analyses were performed using R 4.3.1.

**Results**: Of 139, 671 pregnancies included, 520 (0.4%) were complicated by autoimmune disease and 319 (0.2%) had IBD specifically. Of those with autoimmune disease, 77 (14.8%) were complicated by an HDP diagnosis, compared to 20, 605 (14.8%) without autoimmune diseases. In adjusted and unadjusted analyses (Table 1), there was no increased risk for HDP (aOR 1.00, 95% CI 0.78–1.27) or preeclampsia with severe features. Of those with IBD, 40 (12.5%) were complicated by an HDP diagnosis. In the subgroup analysis (Table 2), those with IBD also had no increased risk of HDP (aOR 0.82, 95% CI 0.58–1.13) or severe preeclampsia.

**Conclusion**: While some autoimmune diseases have known strong associations with preeclampsia, this study suggests that autoimmune diseases overall, including IBD, are not independently associated with HDP. Low dose aspirin use in these patients can be recommended based on other comorbidities in accordance with ACOG guidelines.

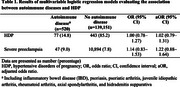

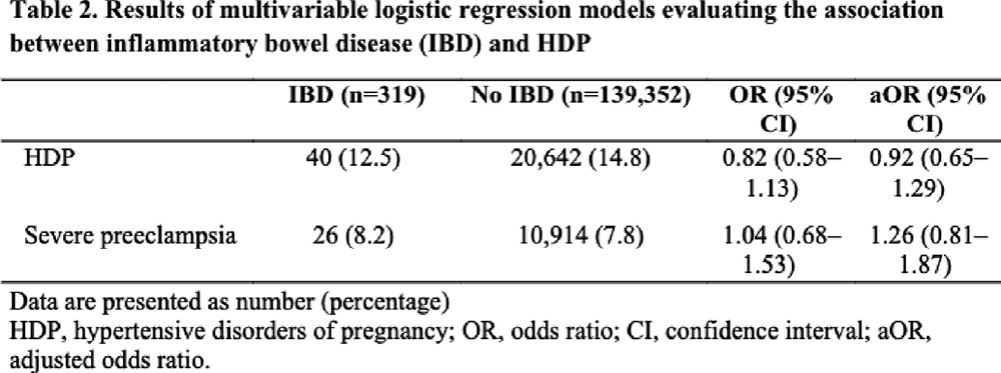

**10:30 AM – 12:00 PM**

## 944 | **Clinical** Exome **and Genome Sequencing Utility in Non‐Isolated Dandy‐Walker Malformation Evaluation: A Retrospective Analysis**

Sarah Araji; Jill Mokry; Seema Lalani; Daryl Scott

Baylor College of Medicine, Houston, TX

**Objective**: Dandy‐Walker malformation (DWM) is a rare congenital abnormality of the posterior fossa and the cerebellum, with an incidence of 1 in 10, 000 to 30, 000 births. DWM is commonly non‐isolated and is associated with other central nervous system (CNS) abnormalities or extra‐CNS anomalies (DWM+). Chromosomal abnormalities are known to be one of the causes of DWM+, however, single nucleotide variant (SNV) testing has not been widely assessed. This work aims to assess the utility of SNV testing in the evaluation of DWM+

**Study Design**: We retrospectively reviewed genetic testing results for individuals with DWM+ undergoing exome or genome sequencing at a tertiary center genetics reference lab between 2012–2025. Age ranged from fetal life – 29 years old. Postnatal median age at diagnosis was 1.19 years old. The primary outcome measure was the rate of diagnosis of exome/genome sequencing in DWM+

**Results**: We analyzed the molecular data of 94 patients with DWM+, and a definitive or possible diagnosis was achieved for 47 individuals, yielding a diagnostic efficacy of 50%. Of these, 23 (48.9%) were a definitive diagnosis and 24 (51%) were possible. We identified several pathogenic variants in genes known to be associated with DWM+, such as CHD7, TUBA1A, TUBB, FKRP, and TMEM138. Our findings are consistent with the previously described association between ciliopathies and DWM; however, we provide substantial new evidence that elucidates the diagnosis of specific ciliopathies, which was previously under‐reported in DWM. We also identified pathogenic variants in other genes that are infrequently considered in the evaluation of DWM+, such as ARID1A, ANKRD11, COL4A1, DDX3X, KMT2D (Kabuki), SMARCB1 (Coffin‐Siris), and KRAS (Noonan).

**Conclusion**: DWM+ is a complex phenotype with various etiologies; these results suggest that the use of exome/genome sequencing can improve the diagnostic rate and shorten the diagnostic odyssey. Furthermore, these findings can also guide providers and expectant parents in making informed decisions about prenatal diagnostic testing.

**10:30 AM – 12:00 PM**

## 945 | **Patient** and **Provider Knowledge and Attitudes Regarding Vaccination with the 9‐valent HPV Vaccine During Pregnancy**

Sarah Boudova^1^; Meghan Gannon^2^; Jennifer Zhan^2^; Valentina Frusone^2^; Aichatou Haidara^2^; Agustina Arce^2^; Kelly McGuigan^2^; Beth Schwartz^2^; Christopher Chambers^2^; Rupsa C. Boelig^3^

^1^University of Illinois Chicago, Chicago, IL; ^2^Thomas Jefferson University, Philadelphia, PA; ^3^Sidney Kimmel Medical College, Thomas Jefferson University, Philadelphia, PA

**Objective**: Cervical cancer is preventable with the HPV vaccine, however vaccination rates remain low. Catch‐up vaccination is currently contraindicated in pregnancy. We aimed to examine patient and provider knowledge and attitudes regarding the 9‐valent HPV vaccine during pregnancy.

**Study Design**: We performed a mixed methods study. Surveys were sent in batches to randomly‐selected providers on the ACOG mailing list until the sample size (*n* = 300) was achieved. Five focus group discussions (FGDs) with 2–5 unvaccinated pregnant individuals/session were conducted until thematic saturation. Thematic analysis was conducted on FGD transcripts. Descriptive statistics were performed on survey data. Data were integrated and interpreted together.

**Results**: We enrolled 18 FGD participants and received 321 provider surveys. Five key themes emerged from survey and FGD data integration: 1) knowledge of the HPV vaccine in pregnancy, 2) perceptions of safety, 3) perceptions of efficacy, 4) the vaccine decision‐making process, and 5) what is needed for comfort with HPV vaccination in pregnancy (Table 1). Patient vaccine knowledge was variable. Patients expressed uncertainty about the safety and efficacy of the vaccine in pregnancy with a primary concern being the baby. Most providers were unsure about the safety (53.9%) and efficacy (52.3%) of the vaccine in pregnancy. Physician recommendation and personal autonomy were key aspects of vaccine decision making. Convergence of findings demonstrate patients and providers desired more research on vaccine safety and efficacy. Provider‐identified barriers to vaccination during pregnancy include the recommendation against vaccination, safety concerns, and patient vaccine hesitancy (Figure 1). The plurality of providers (45.6%) would offer the vaccine in pregnancy if not contraindicated.

**Conclusion**: Patients and providers desire more research on the safety and efficacy of the HPV vaccine in pregnancy. Patients value provider advice on vaccination. Providers adhere to guidelines and report this as the greatest barrier to vaccination in pregnancy.

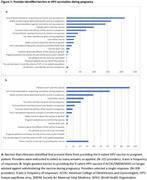

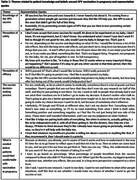

**10:30 AM – 12:00 PM**

## 946 | **Impact of Proximity to Sources of Pollution on Birth Outcomes**

Sarah Y. Cohen^1^; Dong Wang^2^; Laura Brugger^3^; Sarah K. England^2^; Jeannie C. Kelly^4^; Katherine H. Bligard^1^; Antonina I. Frolova^2^; Nandini Raghuraman^2^

^1^Washington University School of Medicine, Saint Louis, MO; ^2^Washington University School of Medicine, St. Louis, MO; ^3^Washington University Brown School of Social Work, Saint Louis, MO; ^4^Washington University School of Medicine, Washington University School of Medicine, MO

**Objective**: Nearly one in four Americans lives near a hazardous pollution site. These sites are disproportionately located in low‐income, Black, and Hispanic communities. This study sought to investigate the association between residential proximity to sites of environmental pollution and pregnancy outcomes.

**Study Design**: This was a secondary analysis of a prospective cohort (2017–2020) of patients with singleton pregnancies delivered at a single Midwestern center. Consenting patients were enrolled before 20 weeks’ gestation and followed through delivery. Pollution sites included solid waste landfills, active industrial sites (per EPA toxic release inventory) and sites containing hazardous waste (EPA Superfund sites). ArcGIS Pro was used to geocode addresses and calculate the distance in miles between maternal residential addresses and pollution sites. Primary outcome was neonatal birthweight. Secondary outcomes were composites of adverse obstetric (OB) and neonatal outcomes. Linear and logistic regression models were used to compare outcomes between close (< 1 mile, commonly used by EPA as toxic waste buffer zone) and distant (≥1 mile) residential proximity to pollution sites.

**Results**: Among 1, 139 patients from the parent study with geocoded addresses, 707 (62%) lived within 1 mile of a pollution site. Patients living close to a pollution site were more likely to be younger, identify as Black, and report tobacco and illicit drug use. After adjusting for age, race, tobacco and drug use, close proximity to a pollution site was not associated with a significant difference in neonatal birthweight, adverse OB outcomes, or adverse neonatal outcomes.

**Conclusion**: In this cohort, close residential proximity to a pollution site was not associated with significant differences in birthweight or adverse pregnancy outcomes after adjusting for key sociodemographic and behavioral factors. While proximity to environmental pollution sites may reflect broader disparities in social determinants of health, these findings suggest that distance alone may not fully capture the risk conferred by environmental exposures during pregnancy.

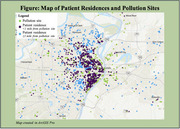

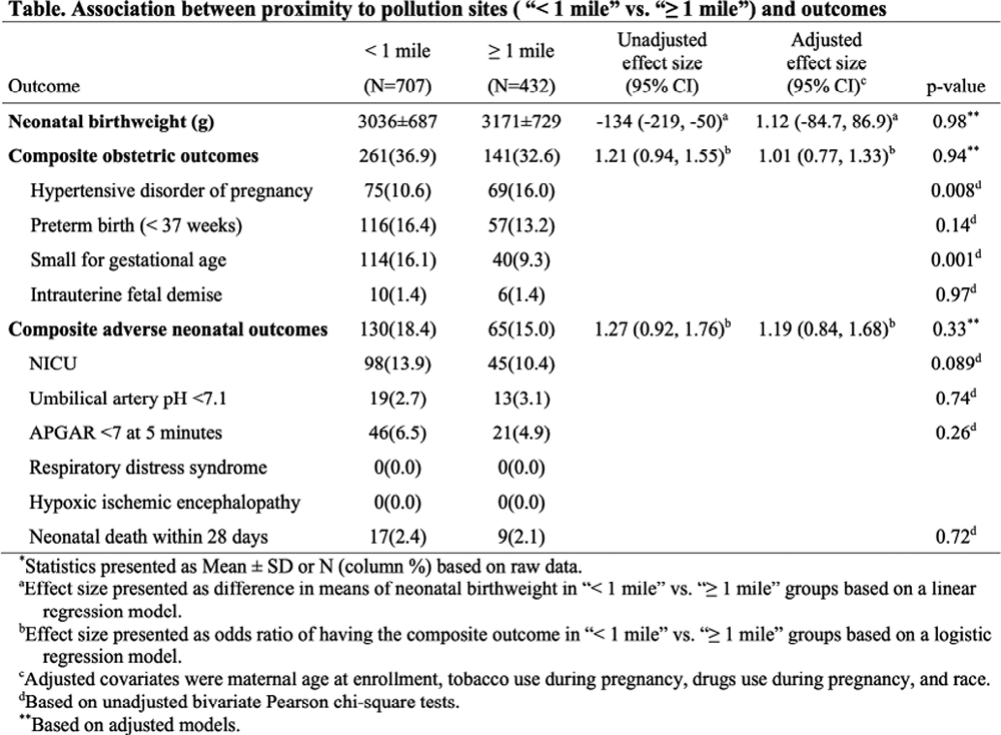

**10:30 AM – 12:00 PM**

## 947 | Optimizing **Post‐Cesarean Opioid Prescriptions Using Inpatient Consumption Data**

Sarah W. Freeman^1^; Mohak Mhatre^2^; Megan J. Li^2^; Meghan E. Beard^3^; Leanne Ludwick^4^; Devika Lekshmi^2^; Sebastian Z. Ramos^2^

^1^Tufts Medical Center, Boston, MA; ^2^Tufts University School of Medicine, Boston, MA; ^3^George Washington School of Medicine, Washington, DC; ^4^Perelman School of Medicine at the University of Pennsylvania, Philadelphia, MA

**Objective**: To evaluate the theoretical impact of three individualized prescribing protocols (based on inpatient opioid consumption) on post‐cesarean discharge prescriptions compared to actual prescribing patterns.

**Study Design**: This retrospective cohort study included all patients 18 and older who underwent cesarean delivery from September 1st, 2023 to December 31st, 2023 at a single academic center. Inpatient opioid use was abstracted from electronic medical records and used to calculate discharge prescriptions based on three previously published protocols: Bleicher et al. (2022), Osmundson et al. (2018), and Smid et al. (2024). These modeled prescriptions were compared to the actual prescriptions received at discharge. Outcomes included total dose prescribed in morphine milligram equivalents (MME) and number of pills prescribed.

**Results**: A total of 208 patients were included who underwent primary (*n* = 131, 63%) or repeat cesarean deliveries (*n* = 77, 37%). Of these, 197 (95%) received an opioid prescription at discharge, and had a median total dose of 75 MME. Both median total dose and number of pills prescribed at discharge were reduced by >70% using the Bleicher or Smid models (Table 1, Figure 1). Compared to actual prescribing, both Bleicher and Smid models reduced the number of prescriptions sent by 37%, while the Osmundson model resulted in a 5% increase in the number of prescriptions issued.

**Conclusion**: Individualized prescribing protocols based on inpatient opioid use after cesarean delivery substantially reduced the total number of prescriptions and opioid dosages sent on discharge, though effectiveness varied by model. Considering inpatient opioid use to inform discharge prescription quantity may enhance opioid stewardship and reduce excess prescribing. Future studies should incorporate patient‐reported outcomes, including satisfaction with pain control, to ensure that reduced prescribing does not compromise adequate postoperative analgesia.

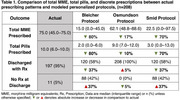

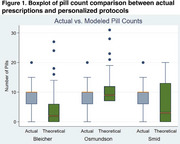

**10:30 AM – 12:00 PM**

## 948 | Longitudinal **Assessment of Prothrombotic and Proatherogenic Gut Microbial Metabolites in Pregnancy**

Sarah C. Graves^1^; Uma Perni^2^; Stanley Hazen^3^; Adina R. Kern‐Goldberger^4^; Jodi Meridieth^2^; Megan R. Ansbro^5^; Xinmin Li^2^; Deepthi Mallela^6^; Madeline Joquico^2^; Cara D. Dolin^7^

^1^VCU School of Medicine, VCU Health, Richmond, VA; ^2^Cleveland Clinic, Cleveland, OH; ^3^Cleveland Clinic, Cleveland, OH; ^4^Cleveland Clinic Foundation, Cleveland Clinic Foundation, OH; ^5^Cleveland Clinic Foundation, Cleveland Clinic Foundation, Cleveland, OH; ^6^Cleveland Clinic Lerner Research Institute, Cleveland Clinic, OH; ^7^Cleveland Clinic, Clevelend, OH

**Objective**: To evaluate trends in maternal serum levels of pro‐thrombotic/atherogenic and cardioprotective gut microbial metabolites and their precursors across pregnancy in a prospective cohort.

**Study Design**: Prospective cohort study enrolling patients ≥ 18 years with singleton pregnancy between 10–14 weeks’ gestation. Maternal blood was collected at 10–14 weeks (visit 1), 24–28 weeks (visit 2), and at time of delivery (visit 3). Assays for predetermined gut microbial metabolites, phenylacetylglutamine (PAG), indole acetic acid (IAA), trimethylamine‐N‐oxide (TMAO), and indole‐3‐propionic acid (I3PA), and their nutrient precursors were performed using isotope dilution LC‐MS/MS methods. Results were summarized and compared across visits.

**Results**: Concentrations for each metabolite across visits are displayed in Figure 1. PAG and IAA increased across gestation, I3PA decreased, and TMAO remained stable. Dietary precursors for TMAO: choline, betaine, γ‐butyrobetaine, crotonobetaine, carnitine, and trimethyl‐lysine all increased from visit 1 to visit 3. Phenylalanine, precursor for PAG, transiently increased at visit 2 but was similar between visit 1 and 3. Tryptophan, precursor for IAA and I3PA, also increased from visit 1 to visit 3 (Table 1).

**Conclusion**: These gut microbial metabolites demonstrated distinct trajectories across pregnancy. Pro‐thrombotic/atherogenic metabolite PAG increased over time, while cardioprotective I3PA decreased. IAA, with likely cardioprotective mechanisms, increased. Despite rising levels of TMAO precursors, TMAO itself remained stable, indicating regulation beyond substrate availability. These findings highlight dynamic changes in gut‐derived metabolites throughout gestation and underscore the interaction of diet, microbial activity, and host metabolism. Longitudinal shifts in these metabolites may have implications for maternal cardiometabolic health and warrant further investigation.

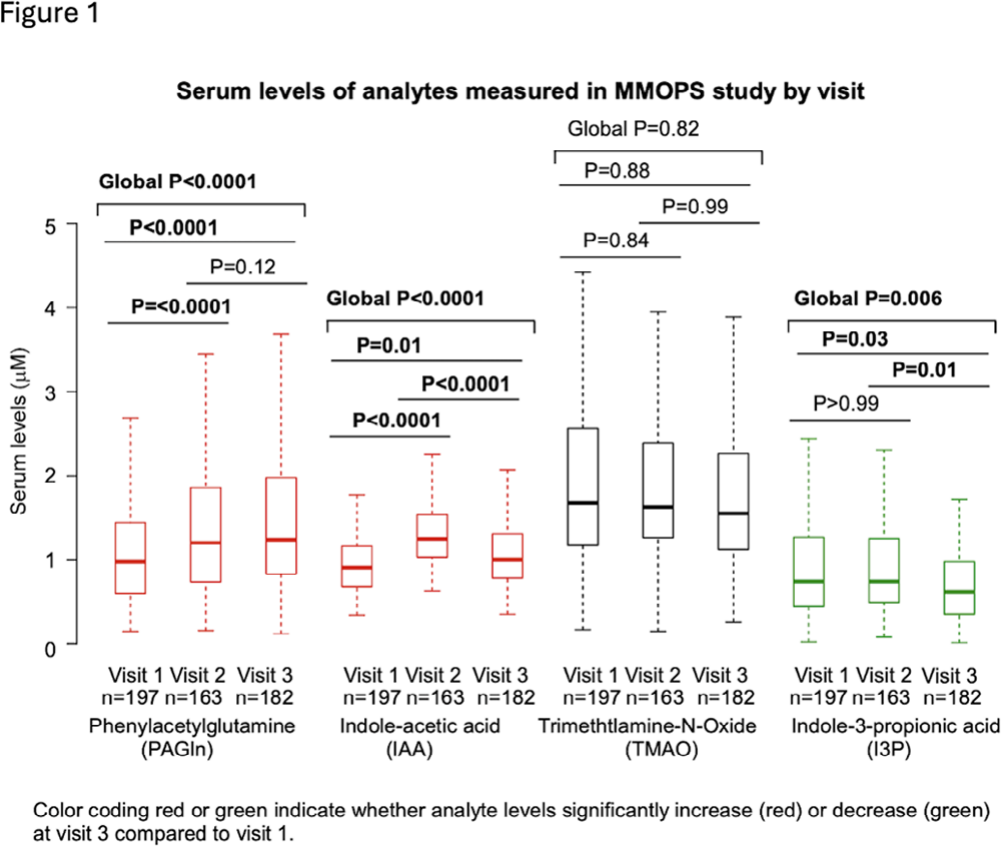

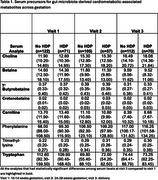

**10:30 AM – 12:00 PM**

## 949 | Developing **a Risk Prediction Model to Understand Cesarean Delivery Rates Within a Large Health System**

Sarah Harris^1^; Sarah White^2^; Asma Ahmed^2^; Robert Phillips Heine^2^

^1^Wake Forest University School of Medicine, Winston‐Salem, NC; ^2^Wake Forest University School of Medicine, Winston Salem, NC

**Objective**: Safely reducing the rate of cesarean birth is an important quality improvement initiative. Current definitions of low‐risk cesarean deliveries do not account for maternal or fetal factors known to increase the risk for cesarean delivery. We aimed to develop and validate a risk prediction model for cesarean delivery among pregnant patients with no prior births.

**Study Design**: We performed a retrospective cohort study within a large health system using Health Records data. All nulliparous patients with a live, term, singleton fetus in the vertex presentation delivered between September 2019 to February 2024 were included. We identified maternal and fetal factors as potential predictors based on literature review and clinical/biological plausibility. Cesarean delivery was defined using diagnostic and procedure codes. We developed a cross‐validated mixed‐effects logistic regression model (GLMM) to predict cesarean delivery using patient and hospital predictors, applying 5‐fold cross‐validation and Platt scaling to align predicted and observed rates. Calibration plots and observed versus expected comparisons by hospital were generated.

**Results**: Our cohort included 23, 724 deliveries occurring at 11 hospitals that had at least 500 deliveries during the study period. The model was well calibrated allowing for accurate site level predictions (Figure 1A). Overall, observed cesarean delivery rates were higher at large, academic hospitals serving higher risk populations and lower for smaller community hospitals (Figure 1B). While observed and predicted rates generally aligned for larger centers, rates differed for smaller hospitals where observed rates tended to be lower than predicted.

**Conclusion**: We developed of a cesarean risk prediction model to improve understanding of cesarean delivery rates. The prediction model can be used to adjust expected cesarean delivery rates at the hospital level, allowing institutions to evaluate their cesarean delivery rates more accurately by accounting for patient level data. Future studies are planned to understand the variance observed within our sample.

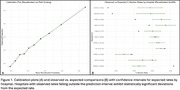

**10:30 AM – 12:00 PM**

## 950 | **Running Dry: Induction of Labor and Obstetric Outcomes during an Intravenous Fluid Shortage**

Sarah Heerboth^1^; Asia Brannon^2^; Katharine Bruce^2^; Sweet Hope Mapatano^2^; Michelle Swanson^2^; Divya Mallampati^3^; Alexandria Kraus^2^; Johanna Quist‐Nelson^3^

^1^University of North Carolina – Chapel Hill, UNC Chapel Hill, Chapel Hill, NC; ^2^University of North Carolina – Chapel Hill, Chapel Hill, NC; ^3^University of North Carolina, Chapel Hill, Chapel Hill, NC

**Objective**: In October 2024, catastrophic flooding caused by Hurricane Helene disrupted the global supply of intravenous fluids (IVF). Our hospital experienced a severe shortage of IVF. We investigated how practice changes in response to the shortage affected obstetric outcomes during induction of labor (IOL).

**Study Design**: We conducted a retrospective cohort study of those with full term, viable pregnancies admitted for IOL between July 1 and December 31, 2024. We excluded those with comorbidities that would independently influence IVF prescribing (e.g. preeclampsia with severe features, pre‐existing cardiac or renal disease). We compared outcomes before and after institutional changes in response to the IVF shortage. (Figure 1) Due to the immediate scarcity of IVF, no washout period was utilized. Statistical analysis was performed using chi square, fisher exact, and t‐tests as appropriate.

**Results**: We included 427 people pre‐implementation and 320 people post‐implementation. Post‐implementation, there were higher rates of nulliparity (43% vs 51%, *p* = 0.04), patients with preeclampsia without severe features (3% vs 8%, *p* = < 0.01), and medically indicated IOL (5% vs 13%, *p* < 0.01). (Table 1) Intrapartum IVF administration was significantly reduced following implementation (2487mL vs 990mL, *p* < 0.01). (Figure 1) There were no differences in mode of delivery or length of labor, but there were more acute kidney injuries in the post‐implementation group. Of the three renal injuries, two were attributed to hypovolemia after postpartum hemorrhage and one to nephrotoxic antibiotic administration. Neonates in the post‐implementation group experienced a smaller reduction in birthweight at 24 hours (3.8% vs 3.0%, *p* < 0.001); there was no change in rate of exclusive breastfeeding.

**Conclusion**: During the IVF shortage, intrapartum IVF use declined significantly. Despite this, we observed no differences in length of labor or mode of delivery. This study highlights opportunities to reevaluate routine intrapartum IVF management and underscores how climate change and natural disasters can affect obstetric practice and maternal health.

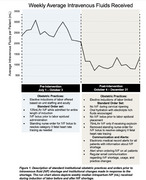

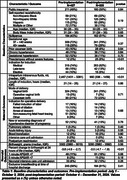

**10:30 AM – 12:00 PM**

## 951 | Prediction **of Spontaneous Labor After 39 Weeks in Low‐Risk Nulliparas: Secondary Analysis of ARRIVE Trial**

Sarah E. Little^1^; Taylor Freret^2^; Akosua Y. Oppong^2^; Anjali J. Kaimal^3^; Mark A. Clapp^4^

^1^Beth Israel Deaconess Medical Center, Newton, MA; ^2^Beth Israel Deaconess Medical Center, Boston, MA; ^3^Department of Obstetrics and Gynecology, University of South Florida, Tampa, FL; ^4^Department of Obstetrics and Gynecology, Mass General Brigham, Massachusetts General Hospital, MA

**Objective**: Individualized predictions of spontaneous labor after 39 weeks could improve informed shared decision‐making around elective induction. Our objective was to understand clinical factors associated with spontaneous labor after 39 weeks in a low‐risk, nulliparous population eligible for elective induction and develop and validate a clinical prediction model.

**Study Design**: Secondary analysis of the ARRIVE trial including participants in the expectant management arm undelivered at 39w0d and excluding those delivered via induction (elective or medically‐indicated) prior to 40w5d (when elective inductions were allowed by study protocol). The primary outcome was spontaneous labor, defined as admission for contractions or augmentation for rupture of membranes. Candidate predictors were identified and the cohort was randomly split into a 70% training and 30% validation set. Discrimination was assessed using AUC and calibration was evaluated using calibration plots.

**Results**: The final cohort included 2231 individuals; 1614 (72.3%) experienced spontaneous labor. In the final model, younger age, lower BMI, less weight gain, non‐smoking status, lower birthweight, higher Bishop score, and any labor‐related triage visit were significantly associated with spontaneous labor (Table 1). The model demonstrated moderate discrimination (AUC = 0.749 training, 0.711 validation; Figure 1). The calibration plot was well calibrated across all predicted probabilities. In the validation set, those in the top quartile (>87% prediction) went into spontaneous labor 90.2% of the time, compared to 65.2% for all others (*p* < 0.01). Results were similar when triage visits for labor‐related complaints were excluded, as this information would not be available prospectively.

**Conclusion**: After 39 weeks, 72% of individuals went into spontaneous labor by 40w5d if not induced prior. Commonly collected clinical variables can be used to refine predictions of spontaneous labor with moderate discrimination. These individualized predictions may better inform patients considering elective induction though need to externally validated.

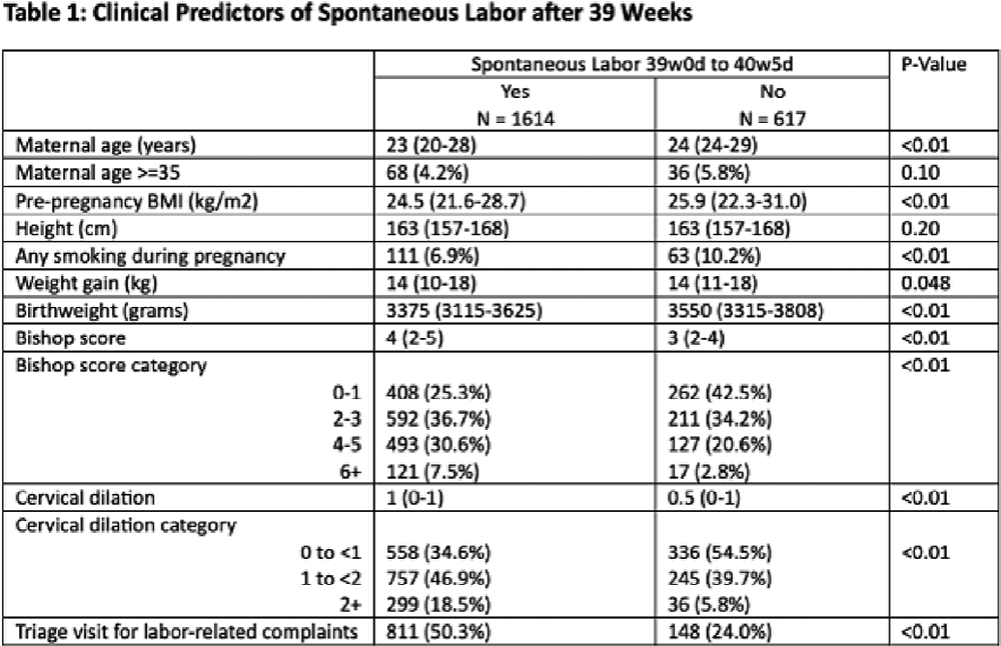

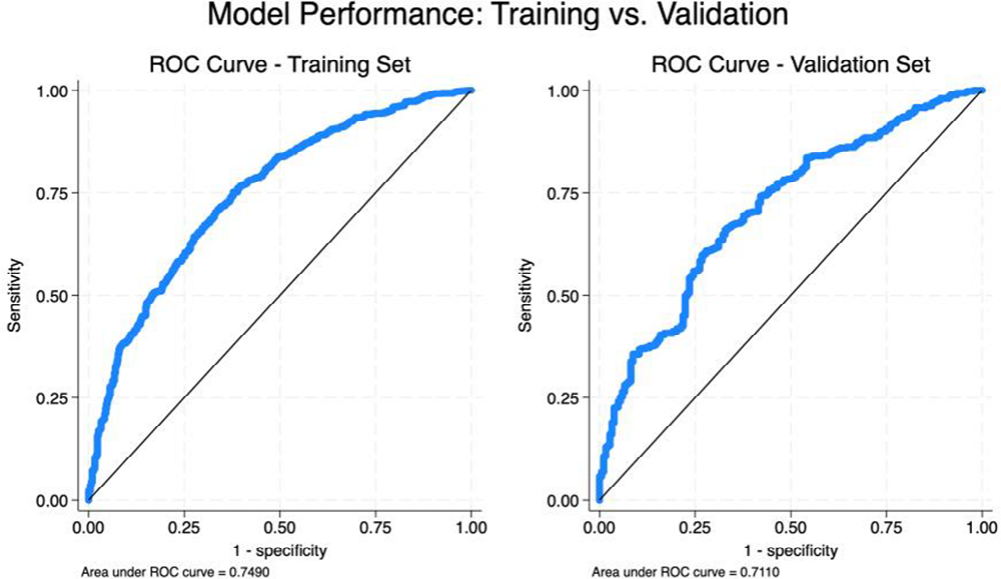

**10:30 AM – 12:00 PM**

## 952 | Comparing **Nifedipine and Labetalol for Blood Pressure Control Using Postpartum Remote Monitoring**

Sarah Shim^1^; Connor Demorest^2^; Katelyn M. Tessier^3^; Martina S. Burn^2^; Tiffany Corlin^2^; Sarah A. Wernimont^2^; Bethany A. Sabol^2^

^1^University of Minnesota Medical Center, Minneapolis, MN; ^2^University of Minnesota, Minneapolis, MN; ^3^University of Minnesota Masonic Cancer Center, University of Minnesota, Minneapolis MN, MN

**Objective**: Postpartum remote blood pressure monitoring (RBPM) programs improve detection and management of hypertensive disorders of pregnancy (HDP), enabling earlier antihypertensive initiation at lower thresholds. While Nifedipine at discharge has been linked to lower readmission risk compared to Labetalol, data on longitudinal postpartum blood pressure (BP) control remain limited. We evaluated BP control, measured as time in target range (TTR), over six weeks of RBPM among individuals discharged on Labetalol compared to Nifedipine.

**Study Design**: This is a retrospective cohort study of postpartum individuals with HDP who delivered between November 2023 and December 2024, were discharged on Labetalol or Nifedipine, and participated in postpartum RBPM. This six week RBPM program utilizes a nurse‐driven standardized protocol for antihypertensive medication initiation and titration to achieve a goal BP of ≤140/ ≤90 mmHg. Based on the standardized protocol utilized, we defined target range as systolic BP 100–139 mmHg and diastolic BP 60–89 mmHg. The primary outcome was BP trend and TTR throughout program participation between the two groups.

**Results**: Of 216 individuals, 40 (18.5%) were prescribed Labetalol monotherapy and 176 (81.5%) Nifedipine monotherapy. While mean blood pressure trends were within goal for both groups (Figure 1), those discharged with Nifedipine spent more time in target range (Labetalol TTR 80.3% vs Nifedipine TTR 87.5%; *p* = 0.03). While there was no significant difference in rates of severe hyper‐ or hypo‐tension (Figure 2), those on Nifedipine experienced fewer elevated BPs (18.3% vs 10.6%; *p* = 0.01). Additionally, the odds of antihypertensive dose escalation were 66% lower for those on Nifedipine compared to Labetalol (OR 0.34, 95% CI 0.13–0.99). Those discharged on Nifedipine were also less likely to remain on medication at 6 weeks postpartum (73.5% vs 36.1%, *p* < 0.001).

**Conclusion**: RBPM is an important tool for postpartum care. This data supports the use of Nifedipine as preferred first‐line antihypertensive agent to achieve rapid and sustained BP control throughout 6 weeks postpartum.

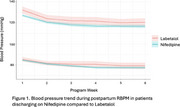

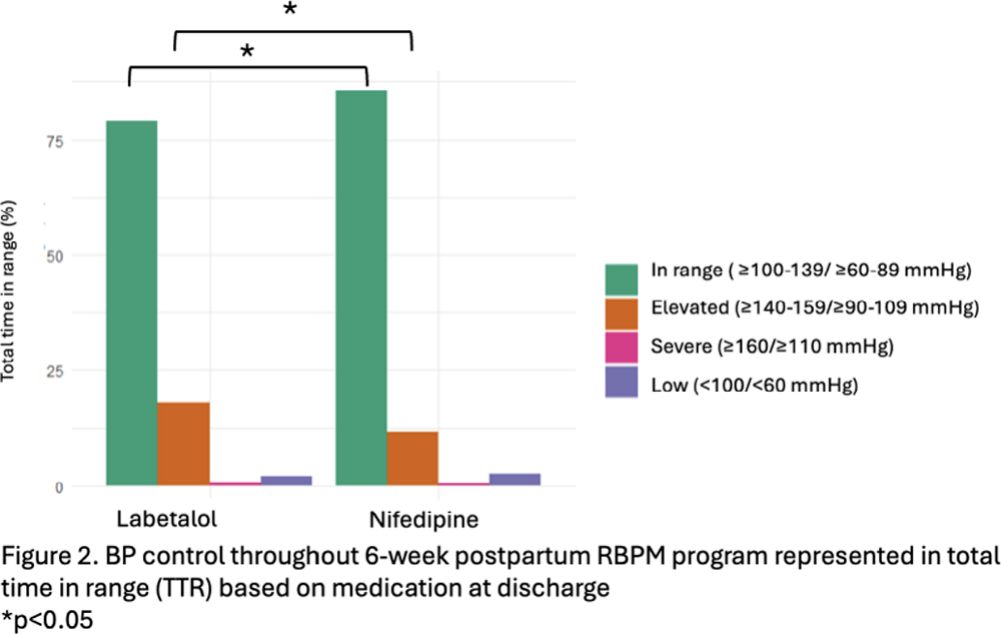

**10:30 AM – 12:00 PM**

## 953 | **Risk** Factors **Associated With the Development of Severe Preeclampsia With or Without Fetal Growth Restriction**

Sascha Wodoslawsky^1^; Mariam K. Ayyash^2^; Rodney A. McLaren, Jr^3^; Taryn Gallagher^4^; Madeline V. Smith^4^; Sam Mauser^5^; Leanna M. Reichert^4^; Huda B. Al‐Kouatly^6^

^1^Department of Obstetrics, Gynecology, and Reproductive Science, Icahn School of Medicine at Mount Sinai, New York, NY; ^2^Columbia University Irving Medical Center, New York, NY; ^3^Maimonides Medical Center, Brooklyn, NY; ^4^Sidney Kimmel Medical College at Thomas Jefferson University, Philadelphia, PA; ^5^Westchester Medical Center, Philadelphia, PA; ^6^Division of Maternal‐Fetal Medicine, Department of Obstetrics and Gynecology, Sidney Kimmel Medical College at Thomas Jefferson University, Philadelphia, PA, USA, Philadelphia, PA

**Objective**: To identify maternal risk factors associated with the development of severe preeclampsia (PE) complicated by fetal growth restriction (FGR) compared with severe PE alone.

**Study Design**: We conducted a retrospective cohort study of singleton pregnancies diagnosed with severe PE between March 2017 and June 2023 at a single health care system. Pregnancies were included if they had a first‐trimester dating ultrasound, a completed anatomy between 18w0d and 24w0d, and were not affected by major structural or chromosomal abnormalities. Cases were stratified by the presence or absence of FGR, defined as an estimated fetal weight or abdominal circumference < 10th percentile for gestational age. Maternal demographic and clinical characteristics evaluated included body mass index (BMI) ≥30 kg/m^2^, race, maternal age ≥35 years, history of chronic hypertension (cHTN), diabetes (pregestational or gestational), autoimmune disease, and renal disease. Comparisons between groups were performed using chi‐square.

**Results**: Out of 9386 maternal and neonatal charts and ultrasounds reviewed, 340 pregnancies had severe PE. Of those pregnancies, 61 (17.9%) were complicated by FGR. There were no significant differences in BMI ≥30 kg/m^2^ (63.9% vs 76.0%, *p* = 0.052), Black race (60.6% vs 58.4%, *p* = 0.75), maternal age ≥35 years (24.6% vs 30.1%, *p* = 0.39), cHTN (36.1% vs 34.8%, *p* = 0.85), and pregestational diabetes (6.6% vs 7.2%, *p* = 0.87) or gestational diabetes (8.2% vs 15.1%, *p* = 0.16) between those with severe PE+FGR and severe PE alone. Autoimmune disease and renal disease were rare and did not differ by group (Table 1).

**Conclusion**: No maternal demographic or clinical risk factors assessed in this study were significantly associated with the development of FGR among pregnancies complicated by severe preeclampsia. Further research is needed to better understand the pathophysiologic and maternal characteristics associated with the development of FGR in pregnancies complicated by severe preeclampsia.

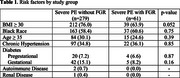

**10:30 AM – 12:00 PM**

## 954 | **Fetal** Doppler **Severity and Outcomes in Pregnancies Complicated by Early Onset Growth Restriction and Preeclampsia**

Scott Infusino^1^; Jeanette M. Larson^1^; Natalia Gontarczyk Uczkowski^2^; Amy Godecker^2^; Jacquelyn H. Adams^3^; J. Igor Iruretagoyena^1^

^1^University of Wisconsin Hospitals and Clinics, Madison, WI; ^2^University of Wisconsin School of Medicine and Public Health, Department of Obstetrics and Gynecology, Madison, WI; ^3^University of Wisconsin School of Medicine and Public Health, Madison, WI

**Objective**: To determine if fetal Doppler severity and progression, as well as pregnancy outcomes, in early onset fetal growth restriction (FGR) are impacted by preeclampsia.

**Study Design**: This was a retrospective cohort study from 2017 to 2023 evaluating singleton, nonanomalous pregnancies with a diagnosis of early onset FGR (< 32 weeks) who underwent serial fetal Doppler evaluation. Doppler evaluation included umbilical artery (UA) pulsatility index (PI), characterization of UA end diastolic flow (forward, absent, or reversed), and characterization of the ductus venosus (DV) a‐wave (forward, absent, or reversed). Pregnancy outcomes were also collected.

**Results**: There were 517 patients that met inclusion criteria. Sixteen percent (*n* = 85) of pregnancies with early onset FGR developed preeclampsia. Patients with a diagnosis of both FGR and preeclampsia were more likely to deliver at an earlier gestational age (32.3 vs 37.6 weeks, *p* < 0.01) and had a shorter interval from diagnosis of FGR to delivery (37.4 vs 80.7 days, *p* < 0.01). Pregnancies with FGR and preeclampsia had a significantly higher Doppler severity at diagnosis of growth restriction and at evaluation before delivery (Table 1). A subgroup of eight patients were identified with severe preeclampsia and growth restriction with reversed umbilical artery Doppler (or late DV changes) that delivered before 28 weeks’ gestation (Table 2). One pregnancy was complicated by stillbirth, while the remaining were delivered for non‐reassuring fetal status or Doppler findings. Five of these pregnancies were complicated by a neonatal or infant death.

**Conclusion**: Pregnancies complicated by both early onset fetal growth restriction and preeclampsia were more likely to have a higher severity of Doppler abnormalities at time of diagnosis and delivery, earlier gestational age at delivery, and shorter interval to delivery. Patients with severe preeclampsia and growth restriction with reversed UA Doppler had particularly poor outcomes. This information can help providers manage these complex pregnancies and adequately counsel patients.

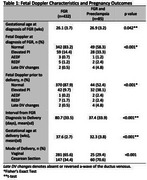

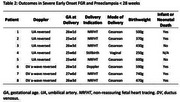

**10:30 AM – 12:00 PM**

## 955 | Association **Between Body Mass Index and Maternal Outcomes Following Uterine Rupture**

Sergio A. Karageuzian^1^; Bo Park^2^; Kevin H. Hu^3^; Ilish Gedestad^1^; Kriti N. Vedhanayagam^3^; Nida Ali^4^; Neville Tritch^5^; Ashra Tugung^4^; Havilah Reimche‐Vu^5^; Ruofan H. Yao^3^

^1^Loma Linda University Health, Loma Linda, CA; ^2^California State University, Fullerton, Fullerton, CA; ^3^Loma Linda University, Loma Linda, CA; ^4^Loma Linda University Children's Health | Perinatal Institute, Loma Linda, CA; ^5^Loma Linda University School of Medicine, Loma Linda, CA

**Objective**: To evaluate the association between maternal body mass index (BMI) and adverse maternal outcomes among individuals with uterine rupture.

**Study Design**: This retrospective cohort study used a national U.S. obstetric database to identify 4, 034, 543 deliveries, of which 5, 253 were complicated by uterine rupture. Patients were stratified by BMI category: underweight, normal weight, overweight, class I, class II, and class III obesity. The primary outcomes were maternal ICU admission, transfusion, and hysterectomy. Multivariable logistic regression was performed to estimate adjusted odds ratios (aOR) for each outcome by BMI category, using normal BMI as the reference and adjusting for confounders including hypertensive disorders, diabetes, maternal age, and number of prior cesareans.

**Results**: Uterine rupture occurred most frequently among underweight individuals (0.19%) compared to other BMI categories (range: 0.11–0.15%). Among patients with uterine rupture, underweight individuals had significantly increased odds of maternal ICU admission (aOR 1.24, 95% CI 1.07–1.44), blood transfusion (aOR 1.24, 95% CI 1.14–1.36), and hysterectomy (aOR 1.31, 95% CI 1.07–1.60). Class III obesity was also associated with increased ICU admission (aOR 1.20, 95% CI 1.12–1.29), but not with increased odds of transfusion or hysterectomy. Overweight and class I obesity were associated with significantly decreased odds of transfusion compared to normal BMI.

**Conclusion**: Underweight BMI is associated with the highest rate of uterine rupture and greater maternal morbidity, including higher rates of ICU admission, transfusion, and hysterectomy. Class III obesity is similarly associated with increased risk for ICU‐level care. These findings highlight the need for vigilant peripartum management of patients at both ends of the BMI spectrum.

**10:30 AM – 12:00 PM**

## 956 | **Dynamics of Immune‐Checkpoint Regulators and Inflammatory Cytokines Associated With Preterm Birth Before and After Cerclage**

Seri Jeong^1^; Keun‐Young Lee^1^; Ga‐Hyun Son^1^; Kyong‐No Lee^2^; Joanne Kwak‐Kim^3^

^1^Hallym University Kangnam Sacred Heart Hospital, Seoul, Seoul‐t'ukpyolsi; ^2^Chungnam National University School of Medicine, Chungnam National University Hospital, Daejeon, Ch'ungch'ong‐namdo; ^3^Rosalind Franklin University of Medicine and Science, Chicago Medical School, North Chicago, IL

**Objective**: This study aimed to investigate the changes in inflammatory cytokines and immune‐checkpoint regulators (ICRs) before and after cervical cerclage, as well as to identify predictors associated with preterm birth in patients with cervical insufficiency (CI).

**Study Design**: A total of 64 women with CI comprised the study group, and 128 endocervical swab samples were collected before and after cervical cerclage. The participants were categorized into two groups based on disease severity, such as membrane bulging vs. non‐bulging, and delivery outcomes, such as preterm vs. full‐term delivery. Seventeen soluble ICRs and 17 inflammatory cytokines were quantified using the Luminex multiplex assay.

**Results**: Our results indicated significant differences in immunologic microenvironments before and after cerclage. Among ICRs, BTLA, CD28, GITR, and CD80/B7–1 were significantly decreased after cerclage compared to before (Figure 1). In contrast, TIM‐3 and PD‐L2 were increased, as well as twelve other cytokines. Pre‐cerclage levels of CCL2/MCP‐1, CXCL10/IP‐10, GM‐CSF, IL‐1alpha, IL‐6, IL‐10, and IL‐17A were significantly elevated in the preterm group compared to the full‐term delivery group. Meanwhile, post‐cerclage levels of LAG‐3, CTLA‐4, CD86/B7‐2, and PD‐L2 showed significantly decreased levels in patients with preterm delivery. Among those, pre‐cerclage CCL2/MCP‐1, GM‐CSF, IL‐1alpha, and IL‐17A levels, and post‐cerclage PD‐L1 level showed significant association with preterm birth according to the univariate analysis (Figure 2). Pre‐cerclage IL‐17A (odds ratio = 2.3, 95% confidence interval = 1.3 to 5.2, and *p*‐value = 0.014) was the most significant predictor in the finally selected model based on the multivariate analysis.

**Conclusion**: Our study revealed the dynamic changes of ICRs and cytokines following cervical cerclage, and identified markers for preterm birth, including pre‐cerclage IL‐17A in patients with CI. By focusing on these components, our work contributes to a better understanding of the local immune landscape and supports the development of targeted strategies to improve pregnancy outcomes.

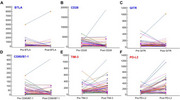

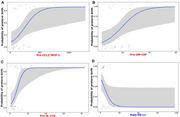

**10:30 AM – 12:00 PM**

## 957 | **Time From Pre‐Labor Rupture of Membrane to Delivery in Grand Multipara Women at Term**

Shai Ram^1^; Roy Bitan^2^; Michael Lavie^1^; Dotan Madar^1^; Anat Lavie^3^; yariv yogev^4^; Roza Shperling‐Berkovitz^5^

^1^Ichilov Tel Aviv Israel, ichilov, HaMerkaz; ^2^Lis Hospital for Women's Health, Tel Aviv, Tel Aviv; ^3^Lis Hospital for Women's Health, Tel Aviv Sourasky Medical Center, Lis Hospital for Women's Health, Tel Aviv Sourasky Medical Center, Tel Aviv; ^4^Lis Hospital for Women's Health, Tel Aviv Sourasky Medical Center, Tel Aviv, Israel, Tel Aviv, Tel Aviv; ^5^Lis Hospital for Women's Health, Tel Aviv Sourasky Medical Center, ichilov, HaMerkaz

**Objective**: To characterize labor progression patterns in grand multiparous women with term pre‐labor rupture of membranes (PROM).

**Study Design**: A retrospective cohort study of all women with singleton term pregnancies (≥37 weeks) and PROM managed expectantly between 2011 and 2023 at a single tertiary medical center. Exclusion criteria were meconium‐stained or bloody fluid, non‐reassuring fetal heart rate, cesarean delivery, labor induction/augmentation, chorioamnionitis, or incomplete documentation. Participants were stratified into three groups by parity: nulliparous, multiparous, and grand multiparous (≥5 prior births). The primary outcome was the time interval from PROM to spontaneous delivery, defined as the birth of the fetus without pharmacologic or mechanical induction or augmentation. Empirical cumulative distribution functions and bootstrap‐generated 95% confidence intervals were used to evaluate labor progression patterns across percentiles.

**Results**: 1. Among 11, 219 eligible women, 4, 347 (38.7%) were nulliparous, 6, 725 (59.9%) multiparous, and 147 (1.3%) grand multiparous.

2. Mean PROM‐to‐delivery intervals were significantly longer in nulliparous women compared to multiparous and grand multiparous women (13.39 ± 11.26 vs 10.22 ± 10.00 and 10.42 ± 11.25 hours, respectively; *p* < .001).

3. Percentile analysis based on the cumulative proportion of women who had delivered following PROM revealed a biphasic pattern among grand multiparas: up to the 60th percentile (i.e., among the earlier 60% of deliveries), PROM‐to‐delivery intervals resembled those of multiparas. However, beyond the 70th percentile – among women who had not yet delivered by that point—labor progression slowed, and their delivery intervals began to approximate those of nulliparas

**Conclusion**: While grand multiparous women generally experience shorter labor following term PROM, prolonged cases demonstrate a shift toward nulliparous‐like patterns. This biphasic progression might inform clinical management.

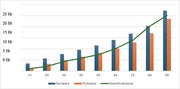

**10:30 AM – 12:00 PM**

## 958 | **Fetal** Deceleration **Features Associated With Acidemia: Is It Type, Depth, Frequency, or Duration That Matters?**

Shannon E. Beermann^1^; Candice L. Woolfolk^2^; Antonina I. Frolova^1^; Jeannie C. Kelly^2^; George A. A. Macones^3^; Alison G. G. Cahill^4^; Nandini Raghuraman^1^

^1^Washington University School of Medicine, St. Louis, MO; ^2^Washington University School of Medicine, Washington University School of Medicine, MO; ^3^Department of Women's Health, Dell Medical School at the University of Texas at Austin, Austin, TX; ^4^Department of Women's Health, Dell Medical School at the University of Texas at Austin, Department of Women's Health, Dell Medical School at the University of Texas at Austin, TX

**Objective**: Total deceleration area (TDA) quantifies the cumulative depth and duration of fetal decelerations over time and is associated with neonatal acidemia. We investigated which specific deceleration features are associated with acidemia among patients with high TDA.

**Study Design**: This is a nested case‐control in a cohort of singleton term pregnancies admitted for induction or spontaneous labor from 2010–2014 at a single center with universal cord gas collection. Fetal heart rate data from the last hour before delivery was extracted by research nurses blinded to outcomes. We included only those with high TDA (≥ 75^th^ percentile). Cases were patients with high TDA and acidemia (umbilical artery pH ≤ 7.10) and controls were patients with high TDA without acidemia. We compared deceleration features including type, depth, duration, and frequency between cases and controls using multivariable logistic regression.

**Results**: Among 1, 958 patients with high TDA in the hour before delivery, 88 (4.5%) had acidemia. Acidemia was significantly associated with a greater number of prolonged decelerations (aOR, 1.18; 95% CI, 1.04–1.33), with duration contributing more to risk than depth. Recurrent late decelerations were also associated with acidemia (*aOR*, 2.54; 95% CI, 1.18–5.28), though neither the depth nor duration of lates were significant. Patients with acidemia were less likely to have recurrent variables, though greater depth of variables was significantly associated with acidemia.

**Conclusion**: Among patients with high TDA, specific decelerations characteristics rather than frequency alone, contribute to acidemia risk. Deceleration depth and duration contribute differently to the risk of acidemia based on the type of deceleration. These findings suggest that more granular, quantitative features of decelerations may improve identification of fetuses at risk.

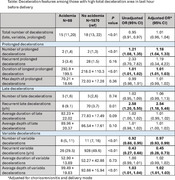

**10:30 AM – 12:00 PM**

## 959 | **The** Protective **Power of Parity: Long‐Term Risk Reduction in Gynecologic Malignancies**

Shayna Miodownik^1^; Gil Gutvirtz^2^; Tamar Wainstock^3^; Eyal Sheiner^4^; Ruslan Sergienko^5^; Roy Kessous^6^

^1^Soroka University Medical Center, Soroka University Medical Center, HaDarom; ^2^Department of Obstetrics and Gynecology, Soroka University Medical Center, Ben‐Gurion University, Metar, HaDarom; ^3^Department of Epidemiology, Biostatistics and Community Health Sciences, Faculty of Health Sciences, Ben‐Gurion University of the Negev, Beer Sheva, HaDarom; ^4^Soroka University Medical Center, Faculty of Health Sciences, Ben‐Gurion University of the Negev, beer sheva, HaDarom; ^5^Ben‐Gurion University of the Negev, ben gurion, HaDarom; ^6^Soroka, BEER SHEVA, HaDarom

**Objective**: Parity has long been recognized as a key factor influencing gynecologic malignancy risk, likely due to hormonal exposures and tissue remodeling due to pregnancy. While prior studies suggest higher parity may be protective, findings vary. We aimed to assess the relationship between maternal parity and the long‐term risk of breast, uterine, and ovarian cancers.

**Study Design**: A retrospective cohort study of women who delivered in a tertiary center between 1991–2021 was conducted. Women were stratified by parity at final pregnancy (from primiparous to parity≥6), and gynecologic cancer diagnoses were assessed using ambulatory and hospitalization records. Kaplan‐Meier survival curves were used to compare cumulative gynecologic malignancy incidence, and Cox regression models were performed to adjust for confounders.

**Results**: Among 28, 642 women included, 810 (2.8%) women subsequently developed a gynecologic malignancy. Among all women in the cohort, multiparous women (≥6 births) had the lowest rates of gynecological cancers (Table). Similarly, women with parity ≥6 presented with the lowest cumulative incidence of gynecological malignancies (Figure). This association remained significant in a Cox proportional hazards model which adjusted for ethnicity and the use of fertility treatments, with parity ≥6 demonstrating a protective effect with significantly lower risk of gynecologic malignancy as compared to primiparous women (Table).

**Conclusion**: Among all the women included in this study, only those with more than 5 deliveries benefited from the protective effect of parity against the development of gynecological malignancy. However, a non‐significant reduction of malignancy risk was also noted for women with more than 3 deliveries, supporting the protective role of recurrent deliveries.

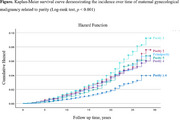

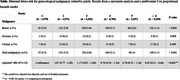

**10:30 AM – 12:00 PM**

## 960 | **Maternal Fetal Medicine Fellow Interest in a Formalized Complex Obstetric Surgery Fellowship Track**

Anna Weinstein^1^; Sheila Moran^1^; Tori Aspir^1^; Arianna Cavalli^2^; Cyrus Jalai^1^; Theofano Orfanelli^1^; Fatima Estrada Trejo^3^; Clara Bertozzi‐Villa^4^; Carly Hirschberg^1^; Pe'er Dar^5^; Georgios Doulaveris^5^

^1^Montefiore Medical Center, Bronx, NY; ^2^Albert Einstein College of Medicine, Bronx, NY; ^3^Montefiore Medical Center, Montefiore Medical Center, NY; ^4^Montefiore‐Einstein, Bronx, NY; ^5^Montefiore Medical Center/Albert Einstein College of Medicine, Montefiore Medical Center/Albert Einstein College of Medicine, NY

**Objective**: To assess Maternal Fetal Medicine (MFM) fellows’ interest in a formalized complex obstetric surgery track within the MFM fellowship, perspectives on training for obstetric surgical emergencies, and comfort with cesarean hysterectomy.

**Study Design**: A cross‐sectional survey of fellows enrolled in ACGME‐accredited MFM fellowships in the U.S. The primary outcome was fellow interest in a formalized surgical training track. Secondary outcomes included training environment, surgical exposure, and comfort with cesarean hysterectomy. Descriptive statistics and chi‐square analysis were used to evaluate associations between training setting and fellow‐reported outcomes.

**Results**: A total of 133 (63.3%) fellows responded to the survey (PGY5: 41%, PGY6: 33%, PGY7: 26%). Most trained at university‐based programs (95%), with representation across all CDC‐defined regions. Overall, 81 (60.9%) expressed interest in a formal MFM surgical track. While 85 (63.9%) felt comfortable independently performing a cesarean hysterectomy, only 53 (39.8%) felt comfortable accepting a post‐fellowship position requiring on‐call surgical backup. Surgical training was rated excellent or good by only 53% of the respondents. When asked who would be called for backup during cesarean hysterectomy, 74 (55.6%) reported non‐MFM (gynecologic oncology or other) while 59 (44.4%) reported MFM faculty. MFM involvement in institutional surgical backup was significantly associated with fellow comfort independently performing hysterectomy (*p* < 0.01), comfort accepting a surgical backup role post‐fellowship (*p* < 0.01), perceived training quality (*p* < 0.01), and interest in a surgical track (*p* = 0.03).

**Conclusion**: A majority of MFM fellows express interest in a formalized surgical training track, with a variable comfort with cesarean hysterectomy. Institutional involvement of MFM in surgical backup is strongly associated with increased confidence and interest in advanced training, supporting the development of a formalized surgical track within MFM fellowship programs.

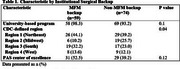

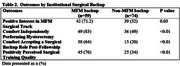

**10:30 AM – 12:00 PM**

## 961 | **Remote** Patient **Monitoring Impact on Short‐Term Outcomes for Individuals With Hypertensive Disorders of Pregnancy**

Shekinah Dosunmu; Casey Lower; Vincent Tang; Nichola Bomani‐Gonzalez; Edwina Iroakazi; Oluwabusola Ola; Sruthi Jayaraman; Joanne N. Quiñones Rivera; Meredith Rochon; Victoria Chestnut; Christopher Nine; Danielle Durie

Lehigh Valley Health Network, Allentown, PA

**Objective**: Remote patient monitoring (RPM) for individuals with postpartum (PP) hypertensive disorders of pregnancy (HDP) has been shown to be an effective alternative to standard follow‐up models. We sought to compare short term PP outcomes between patients enrolled in an RPM program to those who decline enrollment in an academic community health network.

**Study Design**: Retrospective cohort study of patients with HDP referred to a PP RPM program at the time of delivery discharge from 4/13/2020 – 12/31/2022. Demographics, clinical characteristics and postpartum outcomes were compared between those that enrolled and those that declined. The primary outcome was rate of readmission for PP HDP management.

**Results**: Of the 1787 patients during the study period referred for PP RPM at delivery, 1187 (66.4%) enrolled and 597 (33.6%) declined (Table). Patients who enrolled in RPM were more likely to be older, privately insured, nulliparous and married, characteristics often associated with higher healthcare engagement. Enrolled patients were also more likely to have preeclampsia with severe features, deliver preterm and be discharged on antihypertensive medications. Despite evidence of increased disease severity in the enrolled group, readmission rate was similar between groups (5.4% enrolled vs 3.7% declined, *p* = 0.128). Enrolled patients had more frequent PP triage visits, office BP follow‐up and rates of home blood pressure monitoring. Importantly, those enrolled in RPM were more likely to attend their PP visit (89.7% vs 76.6%, *p* < 0.001).

**Conclusion**: Patients who opt to enroll in PP RPM demonstrate a high degree of healthcare engagement, including better compliance with the postpartum visit, an important obstetric quality metric. Despite evidence of more severe disease, there was no increase in PP readmission rates in the enrolled group, suggesting that RPM allows for early identification and outpatient management of HDP complications. Given the possible advantages of RPM, future public health initiatives should focus on barriers to enrollment.

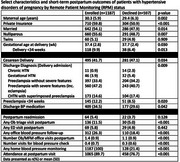

**10:30 AM – 12:00 PM**

## 962 | **The** impact **of 6‐week abortion bans on maternal mental health in Texas**

Shilpi S. Mehta‐Lee^1^; Benjamin Stockton^2^; Ayushi Bommireddipalli^3^; Mia Charifson^4^; Aurora Aurora Scotti^2^; Andrew Fair^2^; Chuan Hong^2^; Linda G. Kahn^2^; Erinn M. Hade^5^

^1^Division of Maternal‐Fetal Medicine, Department of Obstetrics and Gynecology, NYU Grossman School of Medicine, New York, NY; ^2^Department of Population Health, NYU Grossman School of Medicine, New York, NY; ^3^Department of Pediatrics, NYU Grossman School of Medicine, New York, NY; ^4^Department of Population Health, New York University Grossman School of Medicine, New York, NY; ^5^Division of Biostatistics, Department of Population Health, NYU Grossman School of Medicine, New York, NY

**Objective**: Evidence indicates recent abortion bans have led to higher maternal morbidity. Understanding their effect on psychiatric health is crucial. In September 2021, Texas (TX) Senate Bills 4 and 8 (SB4/8), curtailed access to abortion beyond 6‐weeks of gestation. Electronic health records (EHR) provide invaluable data for assessing the consequences of such policies.

**Study Design**: We used Epic Cosmos EHR data to investigate maternal mental health outcomes for pregnancies that began between January 1, 2017, and June 30, 2023, across participating institutions in TX. Those with pregnancies that started before July 1, 2021, were unexposed to SB4/8. There were 600, 536 pregnancies prior to SB4/8 (2017–2021) and 328, 886 following the ban (2021–2023). We assessed rates of depression diagnoses during pregnancy and 60 days postpartum (O99.34, F53, O90.6, F43) through interrupted time series methods based on Bayesian Poisson autoregressive models. Models estimated the cumulative excess diagnoses incidence (95% CI) observed during the ban period compared with the predicted counterfactual diagnosis counts had no ban been implemented, adjusted for demographics and the impact of the COVID‐19 pandemic through model averaging.

**Results**: During the 4.5 years prior to SB4/8, depression rates increased from 18.8 to 32.2 diagnoses per 1000 pregnancies. After the ban rates continued to increase from 32.2 to 39.6 per 1000 pregnancies. There was no evidence of an overall significant increase nor decrease in excess incidence after the ban (‐117.6 cases, 95% CI: ‐1146.0, 883.0). (Figure 1) In subgroup analysis there was some indication of a potential increase in depression diagnoses (Figure 2) in Hispanic women (507.4 excess cases, 95% CI: ‐516.1, 1418.2).

**Conclusion**: EHR data in TX indicate increasing rates of maternal mental health morbidity before and during the COVID‐19 pandemic, which did not revert or stabilize once the crisis was over, and abortion restrictions begun. Further work is necessary to establish the long‐term impacts of abortion bans and the Dobbs decision on maternal mental health.

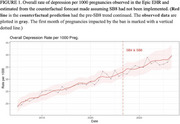

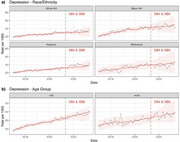

**10:30 AM – 12:00 PM**

## 963 | **Is There a Role for Maternal Inflammatory Markers in Predicting Outcomes After Emergency Cervical Cerclage?**

Shir Zagdon Keidar^1^; Hilit Annie Zucker^1^; Lior Yahav^2^; Tali Silberstein^3^; Maya Maimon Solomom^1^; Tamar Eshkoli^3^; Adi Y. Y.Weintraub^4^

^1^Soroka University Medical Center, Ben‐Gurion University of the Negev, Beer sheva, HaDarom; ^2^Soroka University Medical Center, Ben‐Gurion University of the Negev, beer‐sheva, HaDarom; ^3^Soroka University Medical Center, Faculty of Health Sciences, Ben‐Gurion University of the Negev, Beer sheva, HaDarom; ^4^Soroka University Medical Center, Faculty of Health Sciences, Ben‐Gurion University of the Negev, beer sheba, HaDarom

**Objective**: To assess whether systemic inflammatory markers ‐specifically the neutrophil‐to‐lymphocyte ratio (NLR), platelet‐to‐lymphocyte ratio (PLR), and related indices‐can predict the success of emergency cervical cerclage (ECC) in women with cervical insufficiency diagnosed in the second trimester.

**Study Design**: This retrospective cohort study included 53 women who underwent ECC between 17 and 24 weeks of gestation at a tertiary academic hospital between the years 2015 and 2024. The primary outcome was cerclage efficacy, defined as a prolongation of pregnancy by ≥4 weeks. Demographic, obstetric, and laboratory data‐including NLR, PLR, the systemic immune‐inflammation index (SII), the systemic inflammation response index (SIRI), and the aggregate index of systemic inflammation (AISI), were compared between the effective cerclage and failed cerclage groups.

**Results**: Among the 53 patients, 44 (83%) had a pregnancy prolongation by ≥4 weeks. No significant differences were observed between groups in maternal age, BMI, comorbidities, IVF rates, or hemoglobin levels. The presence of bulging membranes (77.8% vs. 13.6%, *p* < 0.001) and cervical dilation at presentation (77.8% vs. 31.8%, *p* = 0.02) were significantly associated with cerclage failure. However, none of the inflammatory markers demonstrated a statistically significant association with cerclage outcomes. NLR was similar between groups (4.51 ± 2.28 vs. 4.57 ± 2.61, *p* = 0.94). PLR, SII, SIRI, and AISI also failed to distinguish between effective and failed cerclage.

**Conclusion**: Despite a well‐established association between inflammation and obstetric complications such as spontaneous preterm birth, second‐trimester pregnancy loss, and cervical shortening, this study found that commonly used systemic inflammatory markers do not predict ECC effectiveness. These findings highlight the limitations of currently available noninvasive biomarkers in guiding clinical decisions regarding rescue cerclage.

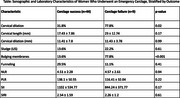

**10:30 AM – 12:00 PM**

## 964 | Effectiveness **of Maternal Antenatal Corticosteroids for Fetal Lung Lesions: Systematic Review and Meta‐Analysis by Lesion**

Shiri R. Warshawsky^1^; Omri Dominsky^2^; Yariv Yogev^3^; Yair J. Blumenfeld^4^

^1^University of Nicosia School of Medicine, Sheba Medical Center, HaMerkaz; ^2^Lis Hospital for Women's Health, Tel Aviv Sourasky Medical Center, Lis Hospital for Women's Health, Tel Aviv Sourasky Medical Center, HaMerkaz; ^3^Lis Hospital for Women's Health, Tel Aviv Sourasky Medical Center Gray Faculty of Medicine, Tel Aviv University, Israel, Lis Hospital for Women's Health, Tel Aviv Sourasky Medical Center, Tel Aviv; ^4^Stanford Medicine, Stanford Medicine, CA

**Objective**: Maternal antenatal corticosteroids (ACS) have been proposed to improve outcomes in fetuses with large congenital lung lesions, though efficacy may vary by lesion type. We systematically evaluated the effectiveness of ACS for large fetal lung lesions, comparing historic Stocker's CPAM‐focused classification (sonographic microcystic vs macrocystic lesions) to contemporary schemes distinguishing CPAM, bronchopulmonary sequestration (BPS), hybrid lesions, bronchogenic cysts, and congenital lobar overinflation (CLE).

**Study Design**: A PRISMA‐compliant systematic review and meta‐analysis was conducted. MEDLINE, Embase, and Scopus were searched through December 2024. Studies reporting outcomes after maternal ACS for mostly large (CPAM volume ratio (CVR) > 1.6) lesions were included. Eight observational cohorts (*n* = 177 fetuses) met criteria; no RCTs were identified. Primary outcomes included: change in CVR, hydrops resolution, and perinatal survival. Subgroup analyses included lesion type and number of ACS courses.

**Results**:

1. Overall, ACS was associated with a significant reduction in CVR (pooled mean decrease ≈1.0; *p* < 0.001), though with considerable heterogeneity (I^2^ ≈ 70%).
2. Among fetuses unresponsive to a single course, additional ACS courses provided a more limited incremental benefit, with lower hydrops resolution rates (56% vs 88%; *p* < 0.01) and similar survival (85–90%).
3. Studies applying the original micro‐ vs macrocystic classification reported greater CVR reductions (40–60%), hydrops resolution (75–80%), and high perinatal survival (85–100%) following a single ACS course compared with more recent lesions‐specific reports.
4. Lesion‐specific analysis confirmed significantly greater CVR reduction and hydrops resolution in CPAM versus non‐CPAM lesions.

**Conclusion**: A single course of maternal ACS is highly effective for reducing lesion size and resolving hydrops in fetuses with microcystic CPAMs. Other lesions demonstrate more limited steroid responsiveness. Additional ACS courses confer diminishing benefit in all lesion types.

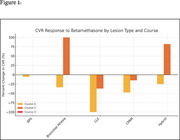

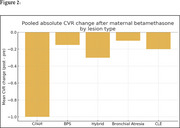

**10:30 AM – 12:00 PM**

## 965 | **Impact of Maternal Substance Use on Infant Birth Weight and Congenital Syphilis Outcomes**

Shreya Battu^1^; Jessica H. Wu^1^; Sophie Gao^1^; Deepa Ravindra^1^; Abigail B. Clark^2^; Heath Yancey^1^; Jessica Sisco^1^; Sophi Farid^1^; Anastasia Kelley^1^; Jessica E. Pruszynski^1^; Emily H. Adhikari^1^

^1^University of Texas Southwestern Medical Center, Dallas, TX; ^2^University of Texas Southwestern Medical Center, University of Texas Southwestern Medical Center, TX

**Objective**: Evaluate the impact of maternal substance on congenital syphilis outcomes.

**Study Design**: This is a retrospective cohort study of pregnant patients with syphilis who delivered at a large, public hospital. We excluded individuals with syphilis treatment prior to pregnancy except in cases of early syphilis. We reviewed medical records for demographics, comorbidities, substances used, STI coinfections, syphilis staging and neonatal outcomes. Maternal substance use was documented in the medical record based on self‐report or indicated maternal urine toxicology testing during pregnancy or delivery. We evaluated the impact of maternal substance use on syphilis‐associated neonatal outcomes and infant birth weight.

**Results**: From January 1, 2010 through June 30, 2025, 581 pregnant patients with syphilis were included, of whom 166 (29%) reported substance use during pregnancy (tobacco [58%], cannabis [54%], methamphetamines [37%], opioids [28%], cocaine [16%] and alcohol [11%]). Compared to patients without substance use, those with substance use were older (27.1 vs. 24.9 years, *p* < 0.001) had higher chronic (13% vs. 5%, *p* = 0.002) and pregnancy‐induced hypertension (40% vs. 26%, *p* < 0.001), and fewer prenatal visits (median 5 vs. 10, *p* < 0.001). Syphilis was also diagnosed later (24 vs. 17 weeks, *p* < 0.001), with initial RPR ≥1:16 more commonly (67% vs. 57%, *p* = 0.026), and less frequent treatment before delivery (73% vs. 93%, *p* < 0.001). Infant birth weight and gestational age at delivery were lower among those with substance use; small for gestational age was more frequent (14 vs 8%, *p* < 0.001). Congenital syphilis was more frequent among substance‐exposed neonates (33[20%] vs. 49[12%], OR 1.83, 95% CI, 1.10–3.02).

**Conclusion**: Maternal substance use in pregnancies affected by syphilis was associated with delayed prenatal care, less adequate treatment, and worse neonatal outcomes, including congenital syphilis, and lower birth weight. Fetal growth concerns in syphilis‐exposed infants may be secondary to superimposed maternal substance use and earlier delivery.

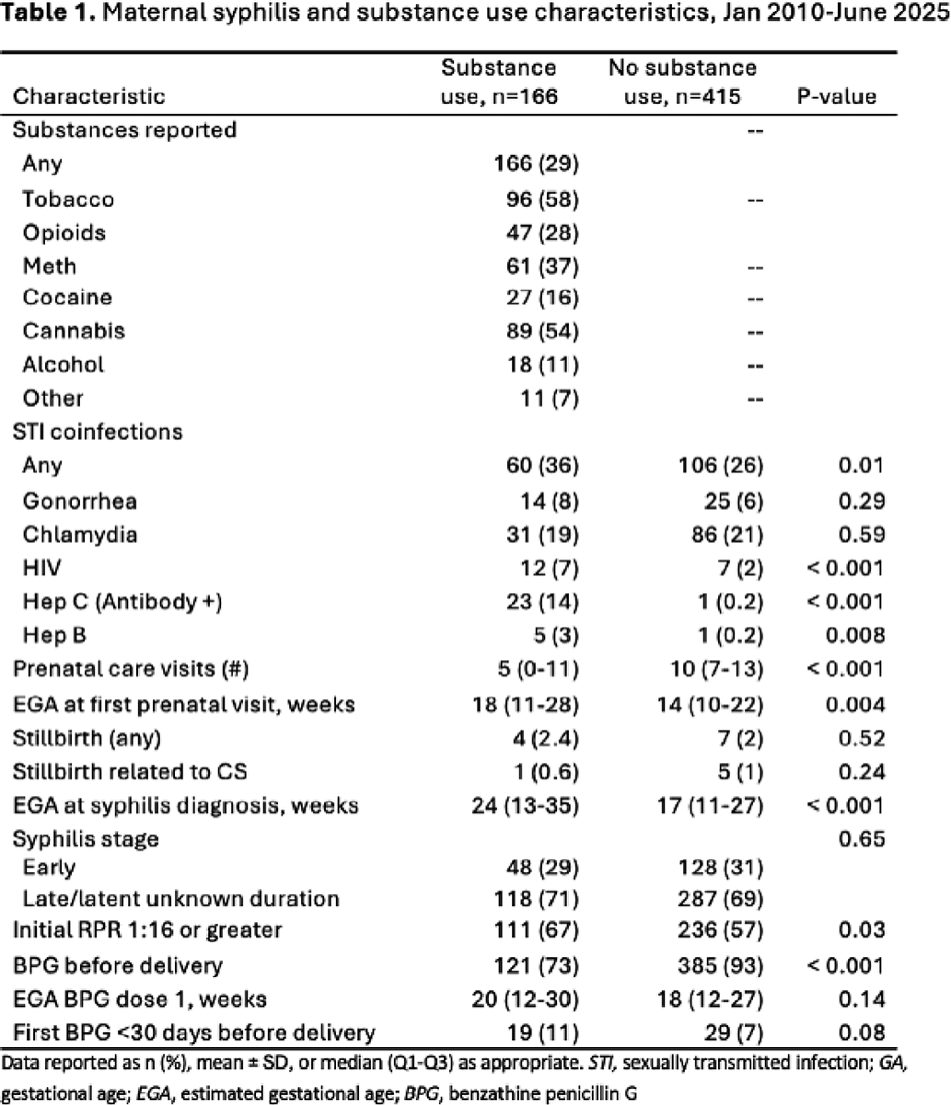

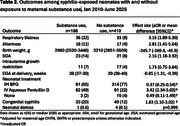

**10:30 AM – 12:00 PM**

## 966 | **Real‐**World **Multi‐center Validation of an Ultrasound Scoring System for Placenta Accreta Spectrum Disorders**

Shuai Zeng; Lian Chen; Yangyu Zhao

Peking University Third Hospital, Beijing, Beijing

**Objective**: Placenta accreta spectrum disorders (PAS), a leading cause of severe obstetric morbidity, require early and accurate diagnosis. While ultrasound remains the first‐line diagnostic tool, existing studies primarily focus on individual sonographic markers, with limited real‐world validation of integrated scoring systems combining clinical and imaging predictors. This study aims to evaluate the diagnostic performance and clinical generalizability of a clinically integrated ultrasound scoring system for PAS, developed in 2016 and widely implemented across Chinese maternity centers.

**Study Design**: A multi‐center retrospective cohort study analyzed 1, 499 high‐risk pregnancies (prior cesarean delivery, placenta previa or uterine curettage) from 14 tertiary hospitals. The scoring system, incorporating eight ultrasound markers (e.g., placental lacunae, bladder‐uterine interface interruption) and clinical risk factors, was applied to antenatal ultrasound records. Diagnostic accuracy was assessed against surgical‐pathological confirmation.

**Results**: The scoring system demonstrated robust diagnostic performance, with an area under the curve (AUC) of 0.785 (95% CI 0.762–0.807, *p* < 0.001) and 0.828 (95% CI 0.804–0.851, *p* < 0.001) for PAS and invasive PAS, respectively in all population. In patients with cesarean section history and placenta previa, the AUCs were the highest among the subgroups, with 0.783 (95% CI 0.735–0.831, *p* < 0.001) and 0.789 (95% CI 0.750–0.828, *p* < 0.001) for PAS and invasive PAS. The best threshold was determined to be 6.5. Nevertheless, in women without CS history and without placenta previa, the AUCs were the lowest, with 0.626 (95% CI 0.526–0.726, *p* < 0.001) and 0.537 (95% CI 0.395–0.678, *p* = 0.279) for PAS and invasive PAS.

**Conclusion**: This large‐scale real‐world validation confirms that the integrated ultrasound scoring system achieves high diagnostic accuracy for PAS across diverse clinical settings. A decade of systematic implementation highlights its practicality as a scalable tool for risk stratification and surgical planning in resource‐varied environments.

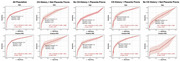

**10:30 AM – 12:00 PM**

## 968 | **Mean** Glucose **and Standard Deviation on Continuous Glucose Monitoring for T1D and Perinatal Outcomes**

Sierra Adkins^1^; Gladys A. Ramos^2^; Ivonne Verduzco^3^; Melissa E. Chambers^4^; Prisca C. Diala^5^; Brianna Dzyuba^6^; Nancy Field^7^; Kaylee Fitzgerald^8^; Christina S. Han^5^; Antoinette Nguyen^9^; Michael Richley^10^; Nasim C. Sobhani^11^

^1^Kirk Kerkorian School of Medicine at the University of Nevada, Las Vegas, Las Vegas, NV; ^2^Division of Maternal Fetal Medicine, Department of Obstetrics, Gynecology and Reproductive Sciences, San Diego, CA; ^3^UCSF, San Francisco, CA; ^4^Division of Maternal Fetal Medicine, Department of Obstetrics and Gynecology, David Geffen School of Medicine at University of California, Los Angeles (UCLA), Los Angeles, CA; ^5^Department of Obstetrics and Gynecology, David Geffen School of Medicine at University of California, Los Angeles (UCLA), Los Angeles, CA; ^6^University of California, San Diego, Division of Maternal Fetal Medicine, Department of Obstetrics, Gynecology and Reproductive Sciences, San Diego, CA; ^7^University of California, Davis, Division of Maternal Fetal Medicine, Department of Obstetrics and Gynecology, Davis, CA; ^8^University of California, Irvine, Irvine, CA; ^9^School of Medicine & Dentistry, University of Rochester, Rochester, NY, Rochester, NY; ^10^University of Washington, Division of Maternal Fetal Medicine, Department of Obstetrics, Gynecology and Reproductive Sciences, Seattle, WA; ^11^UCSF, UCSF/San Francisco, CA

**Objective**: This study examined the association between continuous glucose monitoring (CGM) metrics and perinatal outcomes in pregnant individuals with type 1 diabetes (T1D). We hypothesized that higher mean sensor glucose (MSG) and standard deviation (SD) would be associated with increased risk of adverse outcomes.

**Study Design**: This was a multicenter retrospective cohort study of all gravidas with T1D using CGM who delivered from 2020–2024 at 5 University of California sites. MSG and SD were recorded at 12, 16, 20, 24, 28, and 32 weeks gestational age (GA). The primary outcomes were preeclampsia and large for gestational age (LGA; birthweight ≥90th percentile). Kruskal‐Wallis tests compared the median CGM metrics between those with and without the outcomes. Log binomial regression was used to estimate the relative risk (RR) of the outcomes by CGM metrics, adjusted for years with T1D and diabetic microvascular disease.

**Results**: A total of 254 patients were included. Most used an insulin pump (79%) and did not have diabetic microvascular disease (73%). Median time since T1D diagnosis was 14.9 years, and median periconception hemoglobin A1c was 6.7%. Compared to those without preeclampsia, individuals with preeclampsia had significantly higher MSG and SD at every GA (Figure 1). The comparison between normal‐birthweight infants and LGA infants produced similar results (Figure 2). On adjusted analyses, every 5‐unit increase in MSG was associated with a 9–20% increased risk of preeclampsia at each GA, with highest RR at 12 weeks, while every 5‐unit increase in SD was associated with a 20–29% increased risk of preeclampsia but not at all GA. Similar results were found between MSG and risk of LGA, but SD was not associated with LGA.

**Conclusion**: Increased MSG is associated with higher risk of preeclampsia and LGA, while SD showed weaker associations. These findings highlight the importance of reducing sustained hyperglycemia, particularly early in pregnancy. Further studies are needed to define MSG and SD thresholds to counsel pregnant patients with T1D on minimizing risk.

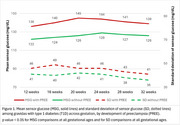

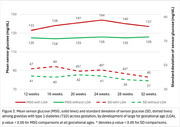

**10:30 AM – 12:00 PM**

## 969 | **Hair** Lipidomics **for Prediction and Pathogenesis of Late‐Onset Fetal Growth Restriction**

Sonia Sajja^1^; Ali Yilmaz^2^; Nadia Ashrafi^2^; Roman Ashrafi^2^; Stewart Graham^2^; Ray Bahado‐Singh^3^

^1^Penn State Health, Penn State Health, PA; ^2^Corewell Health William Beaumont University Hospital, Royal Oak, MI; ^3^Corewell Health William Beaumont University Hospital, Corewell Health WIlliam Beaumont University Hospital, MI

**Objective**: Late‐onset fetal growth restriction (LO‐FGR), or FGR diagnosed ≥ 32 weeks, is associated with increased risk of perinatal asphyxia and death. A meta‐analysis showed that 36‐week biometry has limited accuracy for predicting LO‐FGR (Mustafa 2024). Lipids are critical for fetal growth and placental function. We therefore evaluated maternal hair lipidomics for the prediction of and to elucidate the pathogenesis of LO‐FGR.

**Study Design**: This prospective case‐control study included 29 LO‐FGR cases and 27 appropriately grown controls. Hair samples were profiled using Ultra‐High‐Performance Liquid Chromatography coupled with Mass Spectrometry. Mean lipid concentrations, LO‐FGR vs controls, were compared. Logistic regression models were developed for the detection of LO‐FGR. Areas under the curve (AUC), sensitivity, and specificity values for LO‐FGR prediction based on lipids‐only and lipids combined with 3^rd^ trimester abdominal circumference (AC) measurement. Metabolite set enrichment analysis (MSEA) was conducted to identify the dysregulated biochemical pathways.

**Results**: There was no difference in mean gestational age at sample collection: 35.6 ± 1.9 vs 35.5 ± 2.5 weeks (*p* = 0.482) for cases vs controls. In total, 398 lipids were detected in maternal hair. Overall, statistically significant alterations (*p* < 0.05) in concentrations of 44 lipids were found. The majority of these were triglycerides and diacylglycerols. A lipidomics‐only model achieved excellent diagnostic accuracy: AUC (95% CI) = 0.92 (0.84–0.999) on cross‐validation testing (Figure 1). Lipids plus 3^rd^ trimester AC had a higher predictive accuracy of AUC (95% CI) = 0.932 (0.861–1.00) (Figure 2). MSEA identified dysregulation of important lipid metabolic pathways (*p* < 3x10^−6^), including mitochondrial beta‐oxidation of short‐chain fatty acids and oxidation of branched chain fatty acids in LO‐FGR.

**Conclusion**: Lipid biomarkers in hair achieved high diagnostic accuracy and could represent a novel non‐invasive tool for LO‐FGR detection. Profound lipid disturbance including mitochondrial dysfunction was significantly associated with LO‐FGR development.

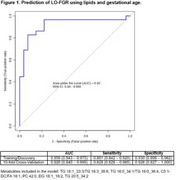

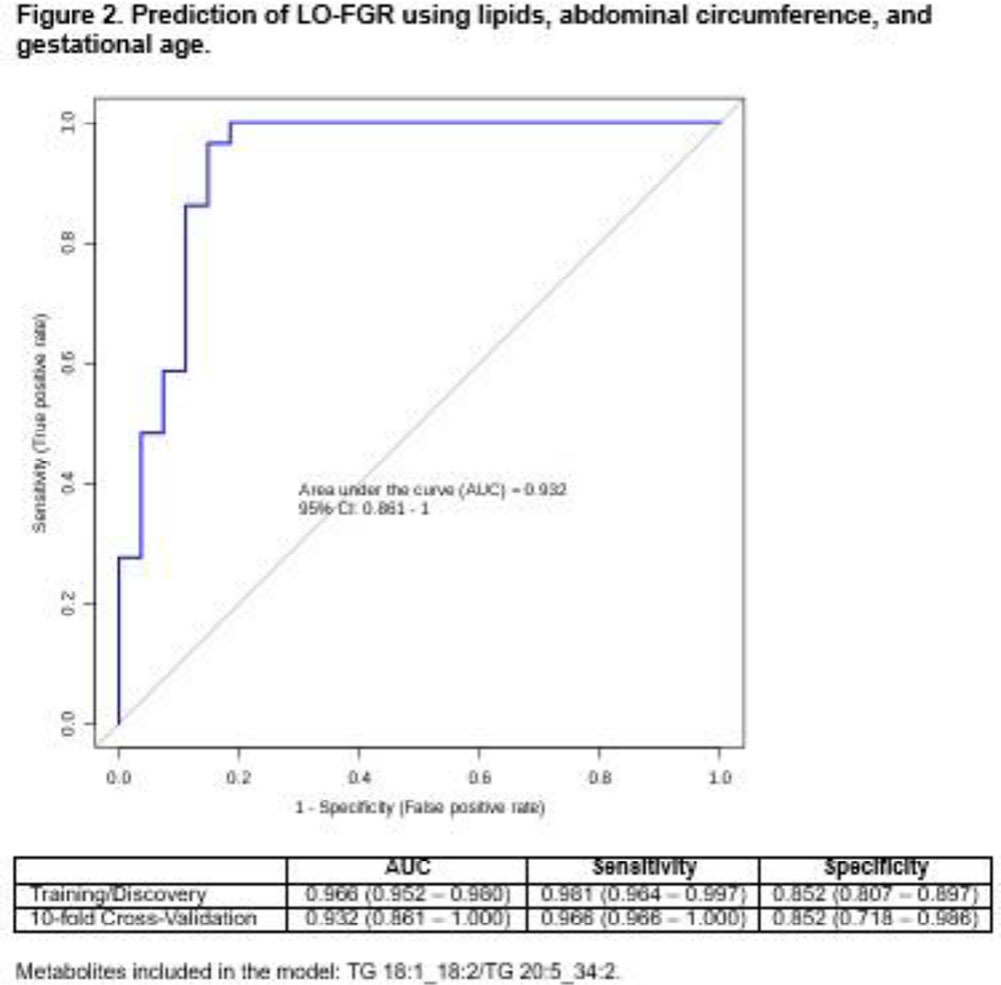

**10:30 AM – 12:00 PM**

## 970 | **Severity of sFlt‐1:PlGF Ratio Elevation and Delivery Within 48 Hours**

Sonya Fabricant; Candace Levian; Tania Esakoff

Cedars‐Sinai Medical Center, Los Angeles, CA

**Objective**: To evaluate the relationship between degree of sFlt‐1:PlGF ratio (SFLT) elevation and need for delivery within 48 hours (48h).

**Study Design**: This was a retrospective cohort study of individuals who delivered at a quaternary care center between 10/13/2023 and 6/21/25. Eligible individuals presented with elevated blood pressure (BP) and had an SFLT performed ≤ 33w4d gestation. SFLT was used to augment clinical evaluation but not to guide delivery timing. Subjects were identified using the Deep6 natural language search engine. Data were abstracted from the medical record. The relationship between time from SFLT collection to delivery was assessed via linear regression (time from SFLT to delivery as a continuous variable) and logistic regression (time from SFLT to delivery < 48h), adjusting for covariates. The diagnostic accuracy of SFLT for delivery < 48h was evaluated using the area under the receiver operating characteristic (ROC) curve (AUC). Diagnostic test statistics for SFLT thresholds were calculated.

**Results**: Of 68 eligible individuals, 12 (18%) delivered within 48h. On linear regression modeling, higher SFLT was independently associated with shorter time to delivery (β = –4.1 days per 100 point increase in the ratio, 95% CI ‐6.0 – ‐2.0, *p* < 0.01). The relationship between SFLT level and time to delivery is illustrated in the Figure. After adjusting for covariates (age, race, BMI, insurance), each 100 point increase in SFLT was associated with a 1.97‐fold increase in odds of delivery < 48h (95% CI 1.28–3.03, *p* < 0.01) (Table). The adjusted AUC for delivery < 48h was 0.85 (95% CI 0.73–0.98). An SFLT threshold ≥ 320 optimized Youden's index (0.56), yielding a 67% sensitivity, 89% specificity, 93% NPV and 57% PPV for delivery < 48h. A threshold ≥ 694 had a 33% sensitivity, 100% specificity, 87% NPV and 100% PPV for delivery < 48h.

**Conclusion**: Among individuals ≤ 33w4d presenting with elevated BP, greater degree of SFLT elevation is associated with increased odds of delivery within 48h. These findings can guide counseling for patients with elevated BP who have an SFLT performed in pregnancy.

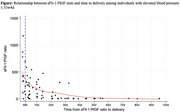

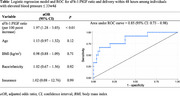

**10:30 AM – 12:00 PM**

## 971 | Abnormal **Umbilical Artery Doppler Studies in Fetal Macrosomia: What do They Mean?**

Stephanie Pham; Megan Geraghty; Sharon A. Fitzgerald Wolff; Megan Thomas

The University of Kansas, Kansas City, KS

**Objective**: Large for gestational age neonates are associated with poorer neonatal outcomes. Umbilical artery Dopplers studies (UADS) are noninvasive tests that evaluate vascular resistance and perfusion which. They are commonly used for pregnancies with fetal growth restriction, however their utility in suspected macrosomic fetuses (SMF) is unknown. We aimed to evaluate the association between abnormal umbilical artery Doppler studies (UADS) and perinatal outcomes in suspected macrosomic fetuses (SMF).

**Study Design**: This retrospective cohort study included singleton pregnancies ≥36 weeks with SMF, defined as EFW ≥90th percentile by Hadlock. Cases with fetal anomalies, aneuploidy, or multiple gestation were excluded. SMFs were categorized by UADS: normal vs abnormal, defined as elevated systolic/diastolic ratio or pulsatility index >95th percentile. The primary outcomes were 1 & 5 minute APGAR scores, NICU admission, arterial cord gas pH and base deficit, hyperbilirubinemia, hypoglycemia, or neonatal death. Multivariable logistic regression analyses were performed using Chi‐Square/Fisher Exact Tests and t‐tests.

**Results**: Of the 447 patients with SMF, 14 (3.1%) had abnormal UADS. More than 70% (*n* = 10, 71.4%) of patients with abnormal UADS experienced any adverse neonatal outcome compared to 45.3% (*n* = 433) of patients with normal UADS (*p* = 0.05). In individual adverse neonatal outcomes, hyperbilirubinemia (50.0% versus 11.0%, *p* = 0.0005), hypoglycemia (28.6% versus 8.6%, *p* = 0.03), and mean cord gas base deficit (5.8 ±  3.3 versus 4.0 ±  3.0, *p* = 0.04) were significantly elevated in those with abnormal UADS. Respiratory distress was also higher in the abnormal UADS group (28.6% versus 15.8%, *p* = 0.26), but not significantly.

**Conclusion**: Among SMF, abnormal UADS are associated with an increased risk of any adverse neonatal outcome by 26%. This may be due to placental insufficiency related to increased demand. Our numbers are small and further studies to assess if UADS may be useful in identifying higher‐risk SMFs are needed to clarify the association.

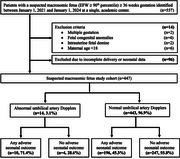

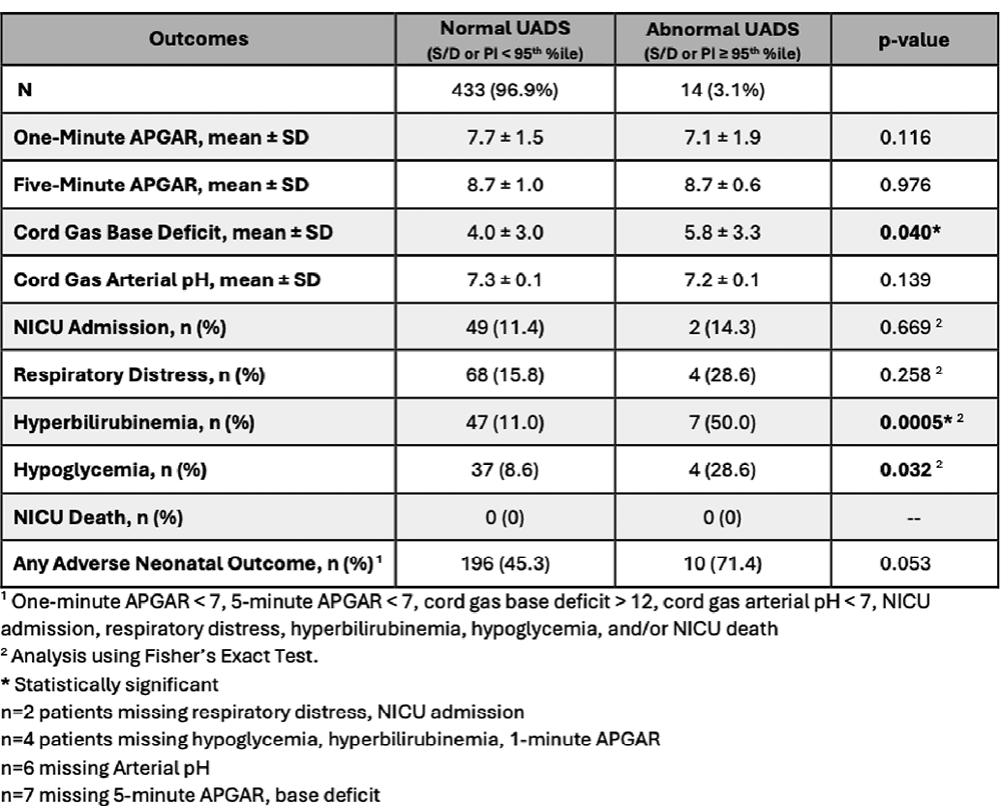

**10:30 AM – 12:00 PM**

## 972 | **Maternal Vaccine Acceptance as a Predictor for RSV Immunization**

Stephanie Schreiber‐Gonzalez^1^; Sandhya Chandrasekaran^2^; Ashish Premkumar^2^; Beth Plunkett^3^; Rachel Ruderman^4^

^1^Ascension Healthcare, Clarendon Hills, IL; ^2^University of Chicago, Chicago, IL; ^3^Endeavor Health Evanston, Evanston, IL; ^4^Endeavor Health, Chicago, IL

**Objective**: To evaluate whether acceptance of influenza, coronavirus‐19 (COVID‐19), and tetanus‐diptheria‐acellular pertussis (Tdap) vaccines are associated with acceptance of the newly‐recommended respiratory syncytial virus (RSV) vaccine in pregnancy.

**Study Design**: We conducted a case‐control study of individuals between 32 and 36 weeks of gestation (the gestational ages at which they would be eligible for RSV vaccination) between 11/2023 and 2/2024 receiving prenatal care at two large urban and suburban medical systems. Exclusion criteria included documented allergy to prior vaccination or pregnancy affected with a lethal fetal anomaly. Covariates included vaccine acceptance defined as receipt of annual influenza and COVID‐19 vaccines as well as the Tdap vaccine during pregnancy. The primary outcome was acceptance of RSV vaccination. Bivariate analyses and a logistic regression were performed to control for confounders including age, race, ethnicity, language, marital status, and insurance status. A *p*‐value of less than 0.05 was used to determine statistical significance.

**Results**: Of 1185 eligible participants, 638 (53.8%) received the RSV vaccine. On bivariate analysis, those who accepted the RSV vaccine were more likely to be older [mean age 33.0 vs 32.7 (*p* < 0.01)], self‐identify as white [263(46.2%), *p* = 0.01], and be married [168 (26.8%), *p* < 0.01](Table 1). When adjusted for confounders, acceptance of other vaccinations in pregnancy were independently associated with RSV vaccination for all three vaccines: Tdap [aOR 4.15, 95% CI (2.25–8.21)], COVID‐19 [aOR 3.30, 95% CI (2.33–4.72)], and influenza [aOR 1.57, 95% CI (1.57–2.82)] (Figure 1).

**Conclusion**: Maternal vaccine acceptance during pregnancy is associated with RSV vaccination, suggesting that patients with established vaccine confidence are more likely to accept new maternal immunizations. Targeted counseling for other vaccines in pregnancy may increase RSV vaccine acceptance and thus improve maternal and neonatal protection.

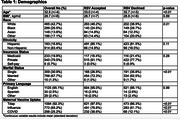

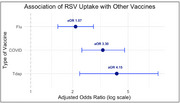

**10:30 AM – 12:00 PM**

## 973 | **Placental Pathology Associated With Preterm Birth in Twin Gestations**

Sunitha Suresh^1^; Penelope Lialios^2^; Alexa A. Freedman^3^; Linda M. Ernst^4^

^1^Endeavor Health, Endeavor Health System, IL; ^2^University of Michigan, University of Michigan, MI; ^3^Northwestern University, Chicago, IL; ^4^Endeavor Health, Evanston, IL

**Objective**: Dichorionic diamniotic (didi) gestation is associated with preterm birth (PTB), without a well described underlying cause. The purpose of this study is to utilize placental pathology to further understand the biologic mechanisms underlying increased risk of PTB in twin gestation.

**Study Design**: This is a retrospective cohort study of live didi twin gestations with placental pathology available delivering at a single health system between 2017 and 2023. Placental pathology was characterized as acute inflammation (AI), fetal vascular malperfusion (FVM), maternal vascular malperfusion (MVM), and chronic inflammation (CI), with presence in **either** twin placenta considered as presence. Prevalence of placental pathology was compared by PTB vs term birth, and subsequently among PTB by spontaneous (sPTB) vs indicated (iPTB) preterm delivery. Frequencies were compared using chi squared tests and logistic regression was used to adjust for covariates‐ IVF pregnancy, maternal age, BMI, preexisting diabetes (DM), and chronic hypertension (CHTN).

**Results**: 373 didi twin pairs were included, of which 49% (184/373) had a PTB. Demographic characteristics were similar by PTB apart from increased DM and CHTN. In the primary analysis, PTB was associated with increased prevalence of MVM (42.4% vs 19.6%, *p* = < . 001, aOR 3.15 95% CI (1.94, 5.11), Table 1). No other pathologies differed by PTB. Among those with a PTB, there was an increased likelihood of AI among those with sPTB vs. iPTB (aOR 4.17, 95% CI 1.88, 9.25) which was attenuated in sensitivity analyses restricted to patients who labored (aOR 1.93, 95% CI 0.71, 5.26). There was no difference in likelihood of MVM by iPTB vs sPTB (aOR 1.01, 95% CI 0.54, 1.89).

**Conclusion**: PTB in twin gestation is associated with MVM. sPTB in twin gestation is associated with a higher odds of AI than iPTB; however, this association was attenuated when restricting to labored patients only. Further research is needed in understanding risk factors underlying MVM as it relates to preterm birth in the didi twin population.

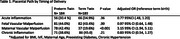

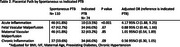

**10:30 AM – 12:00 PM**

## 974 | **RNA‐**Based **Screening for Preterm Preeclampsia Reduces Costs in Pregnancies of Advanced Maternal Age**

Manfred Lee^1^; Brian Nathanson^2^; Avijeet Chopra^2^; Susan Hahn^1^; April Zambelli‐Weiner^2^

^1^Mirvie, South San Francisco, CA; ^2^TTi Health Research & Economics, Hampstead, MD

**Objective**: The objective of this study is to estimate the economic impact of introducing a ribonucleic acid (RNA)‐based risk assessment for preterm preeclampsia (Encompass™, Mirvie) in pregnant individuals of advanced maternal age (AMA; ≥35 years) with no high risk factors for preeclampsia compared to the current United States Preventive Services Task Force (USPSTF) guideline‐based screening.

**Study Design**: Using the Merative^®^ MarketScan Commercial Claims and Encounters Database (01/01/2019 to 12/31/2023), the mean net maternal‐infant payments were quantified in AMA pregnancies with ≥1 moderate preeclampsia risk factor, stratified by preeclampsia status and gestational age (< 28, 28–33, 34–36, and ≥37 weeks of gestational age). Payments included costs incurred from 6 months pre‐delivery to 12 months post‐delivery. A US payer‐focused budget impact analysis incorporated screening and treatment costs (low‐dose aspirin and remote blood pressure monitoring) to compare projected expenditures under RNA‐based risk assessment‐guided care versus USPSTF‐based care. All costs were reported in 2025 US Dollars.

**Results**: Pregnancies with individuals with AMA complicated by preeclampsia incurred substantially higher total costs compared with those without preeclampsia ($2.4 M versus $704 K at < 28 weeks). Additionally, earlier deliveries had a larger cost differential than at‐term deliveries. Incorporating RNA‐based risk assessment into routine care resulted in gross medical cost savings of $5, 678 per pregnancy with AMA screened (or $5.7 M per 1, 000 persons). Cost savings were driven by reductions in severe and early deliveries requiring intensive neonatal care.

**Conclusion**: The implementation of the RNA‐based risk assessment for preterm preeclampsia among individuals with AMA may result in cost savings from the payer perspective. These savings are primarily attributable to targeted interventions and shifts in gestational age at delivery to later stages of pregnancy.

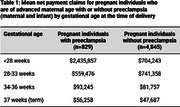

**10:30 AM – 12:00 PM**

## 975 | Organizational **Readiness Factors Predicting Group Prenatal Care Implementation Success: Analysis from a Multi‐Site Randomized Trial**

Sweet Hope Mapatano^1^; Julia Muller^2^; Traci Johnson^3^; Cheron Phillips^4^; Richelle Smith^4^; Kelly McKay‐Gist^4^; Jeannie C. Kelly^5^; Nandini Raghuraman^6^; Melissa Tepe^7^; Candice L. Woolfolk^5^; Shannon Lenze^8^; Danielle M. Vuncannon^9^; Ebony Carter^10^

^1^University of North Carolina – Chapel Hill, Chapel Hill, NC; ^2^University of North Carolina, Chapel Hill, NC; ^3^University of Missouri‐Kansas City, Kansas City, MO; ^4^St. Louis Integrated Health Network, St. Louis, MO; ^5^Washington University School of Medicine, Washington University School of Medicine, MO; ^6^Washington University School of Medicine, St. Louis, MO; ^7^Affinia Healthcare, St. Louis, MO; ^8^Washington University in St. Louis, School of Medicine, Department of Psychiatry, St. Louis, MO; ^9^University of North Carolina, University of North Carolina School of Medicine, NC; ^10^University of North Carolina, University of North Carolina, NC

**Objective**: High group prenatal care (GPC) engagement improves outcomes while low engagement eliminates benefits, potentially explaining recent negative trials. We identified organizational readiness factors that predict high patient engagement in GPC programs.

**Study Design**: Secondary analysis of ongoing multi‐site RCT (2021–2026) examining organizational predictors of GPC implementation success. At the 2.5‐year midpoint, GPC teams from 6‐sites attending a mandatory booster training completed 19‐item organizational readiness assessments. Items were scored as implemented when ≥50% of site team agreed. Pearson correlations examined associations between mid‐study organizational factors and patient engagement: recruitment (% of eligible patients enrolled in study), receipt (≥1 visit), and retention (≥5 visits).

**Results**: Clinical sites enrolled 307 patients total and 26/36 GPC team members (72%) completed readiness assessments. Mean GPC engagement outcomes were 58.4% (307/526) for recruitment (range: 36.2% – 83.1%, SD = 16.5%), 73.2% (150/205) for receipt, (range: 54.5% – 100%, SD = 15.4%), and 48.3% (99/205) for retention (range: 9.1% – 75.0%, SD = 23.6%). Though not statistically significant, the highest predictors of organizational readiness included dedicated facilitator time (r = 0.80; *p* = 0.06), patient incentives (r = 0.69, *p* = 0.13), GPC as the default care (r = 0.55, *p* = 0.26), and staff champions (r = 0.33, *p* = 0.52). Structural, process, and financial factors were implemented across sites.

**Conclusion**: This exploratory analysis suggests operational readiness factors—particularly dedicated facilitator time and patient incentives—show the strongest associations with GPC implementation success. The consistently high implementation of other factors across sites prevented assessment of their predictive value. Given small site sample (*n* = 6), most correlations did not reach statistical significance despite moderate effect sizes. Quality improvement initiatives should target operational readiness gaps since the substantial site variation in retention (9.1%–75.0%) indicates that these barriers may be addressed through implementation.

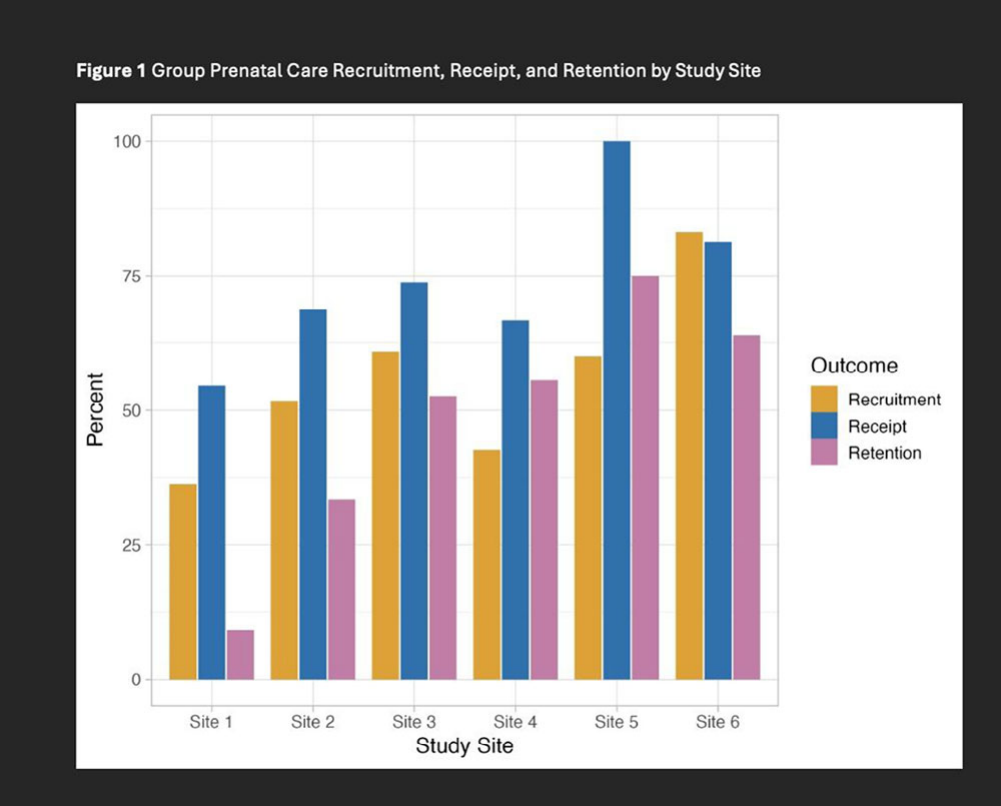

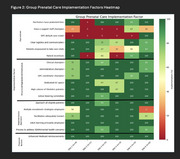

**10:30 AM – 12:00 PM**

## 976 | Association **Between Fertility Treatments and Offspring Risk of Inflammatory Bowel Disease: A Population‐Based Cohort Study**

Tal Czeiger^1^; Gil Gutvirtz^2^; Gali Pariente^3^; Tamar Wainstock^4^; Eyal Sheiner^5^

^1^Soroka Medical Center, Lehavim, HaDarom; ^2^Department of Obstetrics and Gynecology, Soroka University Medical Center, Ben‐Gurion University, Metar, HaDarom; ^3^Department of Obstetrics and Gynecology, Soroka University Medical Center, Ben‐Gurion University, beer sheva, HaDarom; ^4^Department of Epidemiology, Biostatistics and Community Health Sciences, Faculty of Health Sciences, Ben‐Gurion University of the Negev, Beer Sheva, HaDarom; ^5^Soroka University Medical Center, Faculty of Health Sciences, Ben‐Gurion University of the Negev, beer sheva, HaDarom

**Objective**: Fertility treatments are known to modulate maternal hormonal and immunological profiles, which may influence fetal development and long‐term health outcomes. Inflammatory bowel disease (IBD) is a chronic condition with a well‐established immunological etiology. We sought to explore a potential impact of fertility treatments on offspring immune‐mediated conditions and specifically investigated whether offspring of women who conceived using fertility treatments are at an increased risk to develop IBD.

**Study Design**: A population‐based retrospective study was conducted using data from singleton deliveries at a tertiary medical center between 1991 and 2021. The risk of the offspring to develop IBD was compared between children (up to the age of 18 years) born using fertility treatments and children born after spontaneously conceived pregnancies. Offspring were followed up to 18 years of age for the diagnosis of IBD, based on community clinics and/or hospitalization records. The cumulative incidence of IBD was compared between groups using Kaplan–Meier survival analysis, and a Cox proportional hazards model was applied to adjust for potential confounders.

**Results**: A total of 356, 356 singleton deliveries were included in the study, of which 7, 711 (2.2%) deliveries were of pregnancies conceived using fertility treatments. Children born following fertility treatments had a comparable rate of IBD as children born following a spontaneous pregnancy (Table). The Kaplan‐Meier survival curve illustrated comparable cumulative incidence of IBD over time in both groups (Figure). The Cox proportional hazards model, controlling for maternal age and gestational age, reassured that fertility treatments were not a risk factor for the development of IBD in the offspring (Table).

**Conclusion**: In our cohort, children born following fertility treatments are not at an increased risk to develop IBD as compared to children born following spontaneous pregnancies.

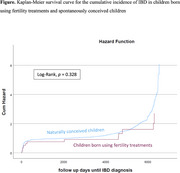

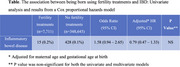

**10:30 AM – 12:00 PM**

## 977 | **Maternal BMI, Mediterranean Diet, and Their Roles in Preterm and Recurrent Preterm Births:Prospective Cohort Study**

Maayan Hagbi Bal^1^; Israel Yoles^2^; Eyal Sheiner^3^; Danit Rivka Shahar^4^; Iris Shoam^5^; Shimrit Yaniv Salem^6^; Neta Benshalom‐Tirosh^4^; Noa Dina Israel‐Tov^5^; Doron Bergman^5^; Ron Rosenbaum^5^; Ifat Baram Goldberg^5^; Ayal Haimov^5^; Noam Tomasis Damri^4^; Ilana Shoham Vardi^4^; Tamar Wainstock^7^

^1^Icahn School of Medicine at Mount Sinai, Beer sheva, HaDarom; ^2^Clalit Health Services, The Central District, Rishon Le Tzion, HaMerkaz; ^3^Soroka University Medical Center, Faculty of Health Sciences, Ben‐Gurion University of the Negev, beer sheva, HaDarom; ^4^Ben‐Gurion University of the Negev, Beer Sheva, HaDarom; ^5^Ben‐Gurion University of the Negev, Ben Gurion, HaDarom; ^6^Faculty of Health Sciences, Joyce & Irving Goldman Medical School at Ben‐Gurion University, Beer sheva, HaDarom; ^7^Ben‐Gurion University of the Negev, Beer sheva, HaDarom

**Objective**: To examine the association between maternal Body Mass Index (BMI), Mediterranean diet adherence, and the risk of PTB and recurrent PTB.

**Study Design**: A prospective cohort of 462 singleton pregnancies at a tertiary center and affiliated clinics (IRB#0390–20‐SOR), including women with and without prior spontaneous PTB, was studied. Maternal BMI was recorded at recruitment and delivery. Mediterranean diet adherence was assessed via a validated short questionnaire. PTB incidence was compared across BMI categories and diet adherence categories, with stratified analysis by PTB history.

**Results**: The study included 462 women: 257 (55.6%) with and 205 (44.4%) without a history of PTB. Of these, 81 (18.1%) had low adherence to the Mediterranean diet (score ≤5/17), 35 (7.6%) were underweight (BMI< 18.5), and 253 (54.8%) had normal weight. Recurrent PTB occurred in 22.5% (*n* = 55), initial PTB in 4.3% (*n* = 8). Underweight women were more likely to have PTB (42.9% [15] vs. 20.6% [88], OR = 2.89, 95% CI: 1.15–7.26). Low diet adherence was associated with increased PTB risk (26% vs. 11.9%, OR = 2.72, 95% CI: 1.5–4.93) and recurrent PTB (42% vs. 18.1%; OR = 3.31, 95% CI: 1.66–6.62; Figure). In adjusted models, low diet adherence increased PTB risk by 19.9% per one‐point decrease (AdjOR = 1.20, 95% CI: 1.02–1.40, *p* = 0.025). Among women with prior PTB, underweight increased recurrent PTB risk (adjOR = 2.69, 95% CI: 1.04–6.93, *p* = 0.040), while low adherence increased it by 25.5% per one‐point decrease (AdjOR = 1.26, 95% CI: 1.05–1.49, *p* = 0.011) (Table).

**Conclusion**: Low adherence to Mediterranean diet is a risk factor for both PTB and recurrent PTB. These findings emphasize the need for tailored nutritional and weight management strategies to mitigate the risk of PTB and recurrent PTB.

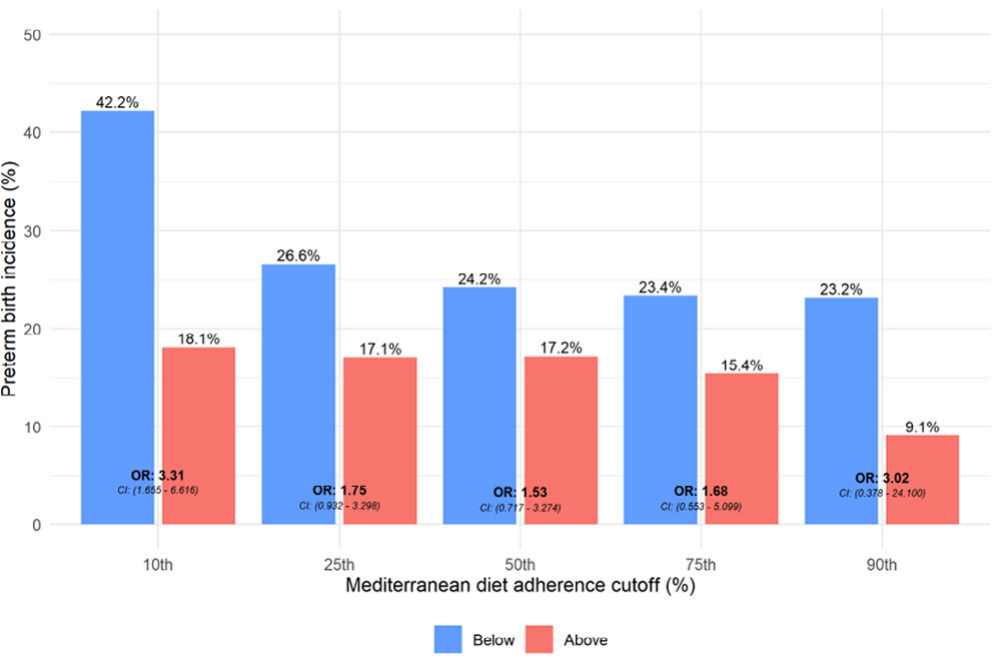

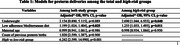

**10:30 AM – 12:00 PM**

## 978 | **The Use** of **Mifepristone During Second Trimester Induction of Labor: A Systematic Review and Meta‐Analysis**

Taytum F. Kahl^1^; Vanya Manthena^2^; Neha Reddy^3^; Preethi Gopal^1^; Courtney N. Sullivan^2^; Jocelyn Wascher^2^; Camille Johnson^2^; Isa Ryan^4^; Hillary McLaren^3^; Shivika Trivedi Kapadia^3^; William A. Grobman^5^; Ashish Premkumar^3^

^1^University of Chicago Pritzker School of Medicine, Chicago, IL; ^2^University of Chicago Medicine, Chicago, IL; ^3^University of Chicago, Chicago, IL; ^4^Endeavor Health Evanston, Evanston, IL; ^5^Brown University, providence, RI

**Objective**: To assess whether providing mifepristone before misoprostol is associated with alterations in the duration of labor (DOL) during second‐trimester induction of labor (2T IOL).

**Study Design**: We performed a systematic review and meta‐analysis of randomized controlled trials (RCT) and cohort studies that included individuals undergoing 2T IOL who received both mifepristone and misoprostol compared to misoprostol between 2000 and 2024 (PROSPERO ID# CRD42024577684). The primary outcome was DOL(in hours) between the first dose of misoprostol to the expulsion of the fetus. Assessment of bias was performed using Cochrane's Risk of Bias tool and Newcastle‐Ottawa Scale for RCTs and cohort studies, respectively. Egger's test was performed for the assessment of publication bias. A secondary analysis evaluated studies based on the indication for IOL (i.e., intrauterine fetal demise [IUFD] or medication abortion [2T MAB]). A leave‐one‐out analysis (LOOA) was performed to assess the impact of individual studies on the overall findings.

**Results**: Of the 513 abstracts identified, 18 met the inclusion criteria (*n* = 1341 in 10 RCTs, *n* = 1867 in 8 cohort studies). 17/18 studies demonstrated a low risk of bias. Among RCTs, the addition of mifepristone reduced the DOL by 5.2 hours (95% CI: ‐6.6 to ‐3.9, Figure 1). A similar trend was seen in the cohort studies (‐4.7 hours, 95% CI: ‐6.5 to ‐2.9, Figure 2). Egger's tests for both RCT and cohort data were non‐significant (*p* = 0.08 & *p* = 0.55, respectively). On secondary analyses, data from RCTs demonstrated that mifepristone had a similar effect on DOL in IUFD (*n* = 276 in 3 studies, ‐4.8, 95% CI: ‐7.1 to ‐2.6) and 2T MAB (*n* = 1065 in 7 studies, ‐5.49, 95% CI: ‐7.3 to ‐3.7). Due to the limited reporting of IUFDs in cohort studies, a secondary analysis could not be performed. The LOOA did not demonstrate any differences in findings.

**Conclusion**: Use of mifepristone, in addition to misoprostol, is associated with a shorter DOL during 2T IOL when compared to misoprostol alone. This effect was similar based on indication for 2T IOL (i.e, IUFD or 2T MAB).

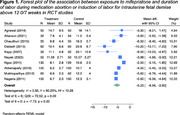

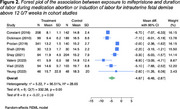

**10:30 AM – 12:00 PM**

## 979 | **Race‐**Specific **Patterns in Discrimination Experiences in Prenatal Care**

Tenisha D. Wilson^1^; Nia Plump^2^; Julia Muller^1^; Traci Johnson^3^; Cheron Phillips^4^; Richelle Smith^4^; Kelly McKay‐Gist^4^; Jeannie C. Kelly^5^; Nandini Raghuraman^6^; Melissa Tepe^7^; Candice L. Woolfolk^5^; Shannon Lenze^8^; Ebony Carter^9^

^1^University of North Carolina, Chapel Hill, NC; ^2^EleVATE Collaborative, St. Louis Integrated Health Network, St. Louis, MO; ^3^University of Missouri‐Kansas City, Kansas City, MO; ^4^St. Louis Integrated Health Network, St. Louis, MO; ^5^Washington University School of Medicine, Washington University School of Medicine, MO; ^6^Washington University School of Medicine, St. Louis, MO; ^7^Affinia Healthcare, St. Louis, MO; ^8^Washington University in St. Louis, School of Medicine, Department of Psychiatry, St. Louis, MO; ^9^University of North Carolina, University of North Carolina, NC

**Objective**: To examine the relationship between perceived everyday discrimination and healthcare discrimination among prenatal patients across racial and ethnic groups using validated instruments.

**Study Design**: Cross‐sectional analysis of baseline data from 159 prenatal patients enrolled in a randomized group prenatal care trial across three Missouri health systems. Perceived discrimination was measured using the Everyday Discrimination (ED) Scale and the healthcare specific Prenatal Interpersonal Processes of Care Discrimination (PD) subscale. Spearman's correlations were conducted overall and within each race/ethnicity group with Benjamini‐Hochberg correction.

**Results**: The sample included 159 patients: 60% Black/non‐Hispanic (BNH) (*n* = 95), 18% Hispanic (*n* = 28), 19% White/non‐Hispanic (WNH) (*n* = 30), and 4% (*n* = 6) another/non‐Hispanic group. All self‐identified Hispanic participants reported Spanish as their primary language. Correlation between everyday and healthcare discrimination varied significantly by race. Hispanic patients showed a moderate positive correlation (ρ = 0.53, p_adj_ = 0.01), while BNH (ρ = ‐0.04, p_adj_ = 0.71) and WNH (ρ = 0.22, p_adj_ = 0.37) patients showed no significant associations.

**Conclusion**: For most patients, experiences of societal discrimination do not predict healthcare‐specific discrimination; however, a significant association was observed among Hispanic patients. Given that all Hispanic participants in our sample were Spanish‐speaking, this link may reflect the compounding impact of language barriers and immigration‐related bias across multiple domains. These findings suggest that healthcare systems maintain significant power to buffer patients from broader societal discrimination and create equitable experiences for most patient groups. However, they also highlight the urgent need for culturally and linguistically tailored interventions to ensure equitable care for Hispanic patients.

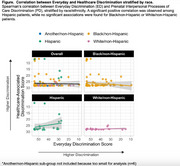

**10:30 AM – 12:00 PM**

## 980 | Recurrent **Neural Network Model for Placenta Accreta Spectrum According to Surgical Technique in Prior Cesarean**

Tess EK Cersonsky^1^; Eugenia Alleva^2^; Isabelle C. Band^2^; Angela T. Bianco^2^; Henri M. Rosenberg^3^

^1^Icahn School of Medicine at Mount Sinai, NY; ^2^Icahn School of Medicine at Mount Sinai, New York, NY; ^3^Icahn School of Medicine at Mount Sinai, Icahn School of Medicine at Mount Sinai, NY

**Objective**: Placenta accreta spectrum (PAS) has risen in incidence with the increase in cesarean delivery rates in the United States. While prior studies have examined uterine closure techniques—such as endometrium‐free closure—and their association with PAS, less is known about how surgical variations at cesarean may influence future PAS risk. We aimed to evaluate if variations in surgical technique, including closure of abdominal layers and suture type, during antecedent cesarean delivery can model PAS risk in a subsequent pregnancy.

**Study Design**: This was a retrospective case‐control study of patients with a history of cesarean delivery and available operative report comparing confirmed PAS cases to propensity‐matched controls. Demographic and medical history were collected for each patient prior to the current pregnancy episode. Data was processed and PAS risk was assessed according to recurrent neural networks (RNN) with K‐fold cross‐validation, creating a model including both static (15 factors) and sequential (variations in 16 surgical steps) data. Feature contribution was evaluated using permutation feature importance.

**Results**: Of 118, 890 records screened, 2, 849 patients (259 PAS cases, 2, 590 controls) were selected; 508 had a prior cesarean with available operative report. The RNN model had a balanced accuracy of 0.78, sensitivity 0.62, specificity 0.95, positive predictive value 0.63, and negative predictive value 0.94. Variation in surgical technique was the most significant contributor to the model, followed by suspected accreta at previous cesarean and pre‐pregnancy BMI. Specific surgical factors associated with PAS included use of cautery for abdominal wall dissection, hysterotomy extension with scissors, and chromic gut uterine closure.

**Conclusion**: Variations in surgical technique at prior cesarean delivery may contribute to PAS risk in subsequent pregnancies. Future studies incorporating complex uterine closure techniques into models, including endometrium‐free closure, may improve risk stratification and inform preventive surgical strategies.

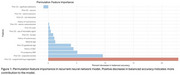

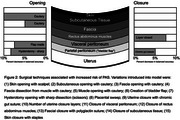

**10:30 AM – 12:00 PM**

## 981 | **Stillbirth at Term: Even Advanced Artificial Intelligence Cannot Predict the Unpredictable**

Martina Benuzzi^1^; Tetsuya Kawakita^2^; Ismail El Moudden^2^; Francesca Monari^1^; Beatrice Melis^1^; Gloria Guariglia^1^; Isabella Neri^1^; Antonio La Marca^1^; Fabio Facchinetti^3^

^1^Obstetrics and Gynecology Unit, Department of Medical and Surgical Sciences for Mothers, Children and Adults, University of Modena and Reggio Emilia, Azienda Ospedaliero‐Universitaria Policlinico, Modena, Italy., Modena, Emilia‐Romagna; ^2^EVMS Department of Obstetrics and Gynecology, Macon and Joan Brock Virginia Health Sciences at Old Dominion University, Norfolk, VA, United States, Norfolk, VA; ^3^University of Modena and Reggio Emilia, Modena, Italy, Modena, Emilia‐Romagna

**Objective**: To assess whether advanced machine learning (ML) models can accurately predict stillbirth at term (≥37 weeks), using clinically derived risk scores and addressing extreme outcome imbalance.

**Study Design**: This was a stratified analysis of 302, 193 term pregnancies (from 325, 860 total), including 322 stillbirths (1.07/1, 000) during the period 2014–2023. Data were obtained from CEDAP (“Certificato di Assistenza al Parto”), the Italian birth registry that collects comprehensive perinatal information on all births occurring in Emilia‐Romagna, a Northern region of Italy. Three domain‐specific risk scores (maternal characteristics, obstetric history, and pregnancy factors) were combined into a composite risk score to train Random Forest and XGBoost models. Class imbalance (936:1) was addressed with cost‐sensitive weighting. Models were trained using 5‐fold cross‐validation and evaluated using AUC‐ROC, sensitivity, specificity, PPV, and NPV.

**Results**: Despite rigorous modeling, both algorithms showed limited performance. Random Forest yielded AUC 0.605, sensitivity 39.1%, PPV 0.24%; XGBoost performed similarly (AUC 0.626, sensitivity 40.6%, PPV 0.23%). NPV was >99.9% but 99.7% of positive predictions were false positives. The top five predictors (gestational age, pre‐pregnancy BMI, maternal age, weight and height) explained 72% of model variance. Gestational age alone contributed 48%, despite the term‐only cohort. Model performance fell below clinical utility thresholds (AUC < 0.70).

**Conclusion**: Stillbirth at term remains largely unpredictable, even with advanced artificial intelligence and rich clinical data. These results highlight a biological knowledge gap, not a technical one—especially regarding placental function in late gestation. In order to meaningfully reduce stillbirth risk, future research must shift from static data modeling to dynamic, physiologically‐based investigation.

**10:30 AM – 12:00 PM**

## 982 | Comparative **Effectiveness of Low‐ vs. Standard‐Dose Misoprostol for Medication Abortion from 24–27 Weeks' Gestation**

Theresa Christensen^1^; Nikita A. Kakkad^2^; Antoinette Oot^3^; Steven Friedman^4^; Justin S. Brandt^5^; Christina Jung^3^

^1^Department of Obstetrics and Gynecology, NYU Grossman School of Medicine, New York, NY; ^2^NYU Grossman School of Medicine, New York, NY; ^3^Division of Family Planning, Department of Obstetrics and Gynecology, NYU Grossman School of Medicine, New York, NY; ^4^Division of Biostatistics, Department of Population Health, NYU Grossman School of Medicine, New York, NY; ^5^Division of Maternal‐Fetal Medicine, Department of Obstetrics and Gynecology, NYU Grossman School of Medicine, New York, NY

**Objective**: Recent guidelines on medication abortion (MAb) from 14 0/7 to 27 0/7 weeks’ gestation recommend reduced misoprostol dosing from 400mcg q3h to 200mcg q3h for cases ≥24 0/7 weeks, though data are limited as few studies include patients beyond 24 weeks. We sought to assess time to delivery and safety of misoprostol 200mcg q3h vs. 400mcg q3h for MAb from 24–27 weeks’ gestation.

**Study Design**: This was a retrospective cohort study of MAbs from 24 0/7 to 27 0/7 weeks’ gestation at an urban quaternary care health network from 7/2022 to 6/2025. All patients received 2 mg intraamniotic digoxin and 200 mg oral mifepristone followed at 24–48 hours by buccal or vaginal misoprostol, either 200mcg q3h or 400mcg q3h. The 400mcg group included some patients who received an 800mcg misoprostol loading dose at start of induction. The primary outcome was time from first misoprostol dose to delivery. Secondary outcomes were delivery complications. We calculated descriptive statistics and performed regression analyses to estimate odds ratios with corresponding 95% CIs.

**Results**: Of 55 patients, 27 received 200mcg q3h and 28 received 400mcg q3h, with demographics balanced between groups [table 1]. Mean time to delivery was 2.6 hours longer in the 200mcg group than the 400mcg group [table 2]. Receiving an 800mcg loading dose was not associated with shorter time to delivery. Receiving 200mcg q3h was associated with lower risk of fever (14.8% vs 25.0%, OR 0.52) but higher risk of retained placenta (14.8% vs 3.6%, OR 4.17) compared to 400mcg q3h. Receiving 200mcg was also associated with increased risk for estimated blood loss ≥500mL, manual placental removal, and dilation and curettage.

**Conclusion**: The results of this exploratory study signal that reducing misoprostol from 400mcg q3h to 200mcg q3h for MAb from 24–27 weeks’ gestation may be associated with longer time to delivery, higher rates of retained placenta, and higher blood loss, but lower rates of fever. Larger cohorts are needed to corroborate our findings and clarify the potential benefits of reduced misoprostol dosing for MAb from 24 0/7 to 27 0/7 weeks’ gestation.

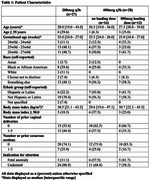

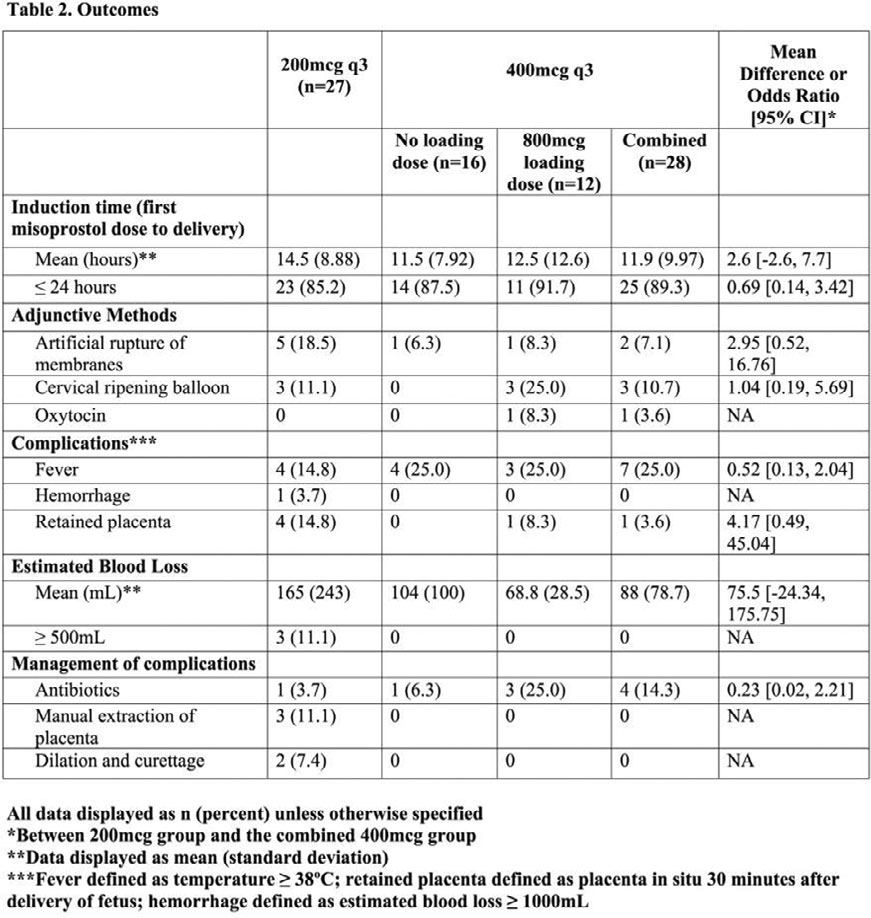

**10:30 AM – 12:00 PM**

## 983 | Postpartum **Mental Health Outcomes in Women Prescribed Tumor Necrosis Factor Alpha Inhibitors**

Audrey Mutoni; Tina Hsieh; Ai‐ris Y. Collier

Beth Israel Deaconess Medical Center, Boston, MA

**Objective**: Several studies explored improved depression symptoms in non‐pregnant individuals using TNFα inhibitors (TNFαi); however, data addressing postpartum mental health outcomes in TNFαi users are limited. We aimed to investigate mental health outcomes in postpartum mothers following exposure to TNFαi from 1 year preconception to early pregnancy compared to non‐users or conventional synthetic disease modifying antirheumatic drugs (csDMARDs) exposure.

**Study Design**: We queried the TriNetX U.S Collaborative network with 71 healthcare organizations for deliveries of women with psoriasis, inflammatory bowel diseases, rheumatoid arthritis, ankylosing spondylitis, or juvenile arthritis aged ≥ 18 years from 11/6/2012 to 12/31/2024. The TNFαi group was defined as those exposed to etanercept, adalimumab, adalimumab, infliximab, certolizumab, or golimumab within 1 year preconception to 20‐week gestation. Two control groups: 1) non‐users; 2) active comparators: sulfasalazine, azathioprine, hydroxychloroquine, and mesalamine, were defined. Patients were followed up from 1 day after delivery (index date) up to 1 year for these outcomes: postpartum depression or mood disturbance, anxiety, bipolar or obsessive‐compulsive disorder, post‐traumatic stress disorder, or suicidal ideation/suicidality. Risk ratios (RR) and 95% confidence intervals (CI) were derived by comparing the outcome risks of TNFαi group that were 1:1 propensity‐score matched (PSM) to controls by demographics, comorbidities, substance use, immunomodulators, healthcare behavior, and BMI. We assessed both total (including recurrent and new‐onset) and new‐onset diagnoses for each outcome.

**Results**: Compared to non‐users (Figure 1) and csDMARDs users (Figure 2), the TNFαi cohort had lower total rates of bipolar disorder diagnoses (RR 0.55, 95% CI 0.31–0.96) but did not show any significant differences in other postpartum mental health outcomes for both total and new onset cases.

**Conclusion**: TNFαi exposure from 1‐year prior pregnancy to early second trimester was not associated with adverse mental health outcomes in the post‐partum period.

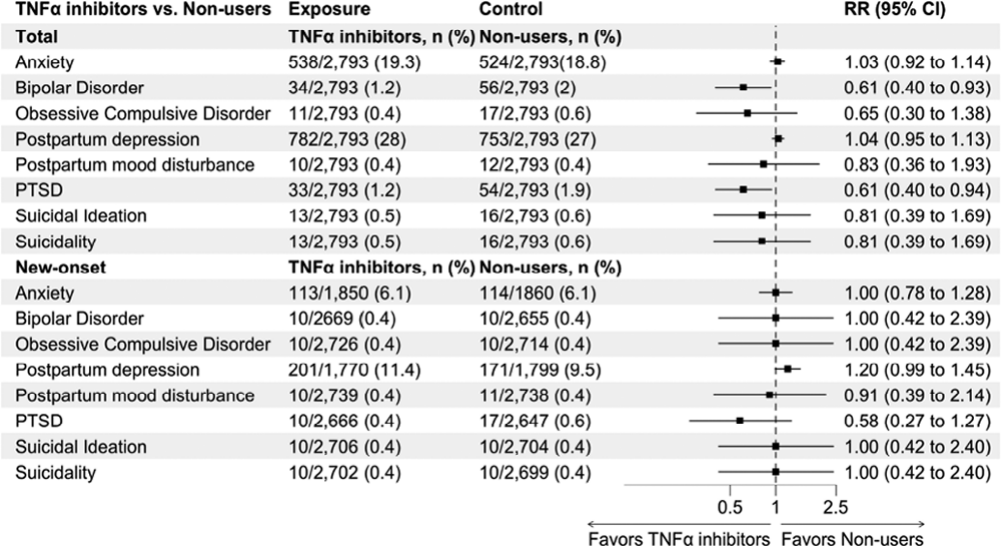

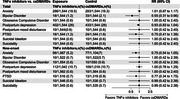

**10:30 AM – 12:00 PM**

## 984 | Discordance **Between Inflammatory Cervicovaginal Cytokine Profiles and Clinical Outcomes Among Individuals With Asymptomatic Short Cervix**

Tracy A. Manuck

On behalf of the Eunice Kennedy Shriver National Institute of Child Health and Human Development Maternal‐Fetal Medicine Units Network, Bethesda, MD

University of North Carolina, Chapel Hill, NC

**Objective**: In a prior analysis of the TOPS RCT of pessary vs. usual care in singleton pregnancies with a short CL (≤20 mm) and no prior PTB, we developed a composite pro‐inflammatory cytokine score from 12 cervicovaginal fluid (CVF) cytokines obtained at enrollment [median 21.8 (IQR 21.4, 22.4) weeks]. Higher scores indicate higher inflammation and associated eventual PTB and maternal & neonatal infections. As all participants were asymptomatic, the cytokine score reflects subclinical inflammation. We sought to identify factors associated with clinical outcome‐cytokine score discordance (e.g., good outcomes despite high cytokine scores or poor outcomes despite low cytokine scores).

**Study Design**: Secondary analysis of TOPS; we included those with low (quartile 1) or high (quartile 4) CVF cytokine scores. The primary outcome was outcome‐score discordance, defined as PTB < 35 weeks ± maternal (chorioamnionitis) or neonatal (sepsis ± pneumonia) infections contrary to cytokine score expectations. We evaluated patient and clinical factors associated with outcome‐score discordance using logistic regression.

**Results**: Of 531/544 RCT participants with a cytokine score, 226 met inclusion criteria; 121 had high scores (of whom 47% had unexpectedly good outcomes); 105 had low scores (of whom 24% had unexpectedly poor outcomes). Randomization CL and antenatal bleeding were associated with clinical outcome‐cytokine score discordance in both those with low and high cytokine scores. Pessary use was associated with lower odds of unexpectedly good outcomes in the high cytokine score group but not with poor outcomes in the low cytokine score group.

**Conclusion**: Quantification of mid‐pregnancy subclinical inflammation by CVF cytokines does not fully explain PTB ± maternal or neonatal infections among individuals with an asymptomatic short CL. The value of adding clinical factors such as vaginal bleeding and the extent of cervical length shortening differs by extent of mid‐pregnancy CVF subclinical inflammation, highlighting the complexity of PTB pathophysiology and emphasizing the importance of integrating clinical and biologic data.

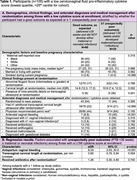

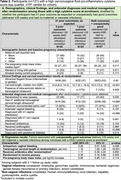

**10:30 AM – 12:00 PM**

## 985 | **Effects of Extracellular Matrix Components on First‐Trimester Trophoblast Stem Cell Growth and Syncytialization**

Uma B. Gaffney^1^; Yalda Afshar^2^; Christina J. Megli^3^

^1^University of Pittsburgh School of Medicine, Pittsburgh, PA; ^2^David Geffen School of Medicine at University of California, Los Angeles (UCLA), Los Angeles, CA; ^3^University of Pittsburgh Magee Women's Hospital, University of Pittsburgh Magee Women's Hospital, PA

**Objective**: Previous studies have demonstrated that components of the extracellular matrix (ECM) can influence cell growth and function. Yet, the effect of ECM on trophoblast cells is poorly described. This is relevant for understanding trophoblast migration and invasion with development of placenta accreta, where trophoblasts come directly into contact with an ECM laden scar. We hypothesize that first‐trimester trophoblast stem cells respond to ECM altering growth, syncytialization and protein expression.

**Study Design**: The CT‐27 placental trophoblast stem cell line was cultured on collagen I, collagen IV, iMatrix, Matrigel, and gels of collagen I and Matrigel. Syncytialization was performed (as described in Okae *et al* 2017). Microscopy was used to monitor cell adhesion and morphology. Supernatant media was collected and analyzed using ELISA for levels of B‐hCG. Bulk RNA sequencing was performed to look for differences in transcripts. CT‐27 cells were cultured on an Advanced Biomatrix ECM Select™ Array Kit Ultra‐36 and imaged to characterize cell adhesion.

**Results**: CT‐27 cells adhered to and grew on collagens I and IV, iMatrix, and Matrigel. Growth kinetics increased with adhesion to collagen IV, iMatrix, and collagen I. On the gel of collagen I, compared to Matrigel, growth was delayed, and cells never invaded into the gel. Syncytialization occurred only on iMatrix and collagen IV. CT‐27 produced b‐HCG on collagens I and IV, iMatrix, or Matrigel, which was enhanced when cells syncytialized. RNA sequencing showed distinct differences in trophoblast transcriptional profiles in response to different collagens. CT‐27 cells adhered to all ECM combinations except for tropoelastin and tropoelastin with collagen VI. However, after 48 hours of growth, cell morphologies were distinct on each surface.

**Conclusion**: ECM exposure alters both the rate and patterns of cell growth, cellular morphology and syncytialization. These data suggest that future studies on the interaction of ECM and trophoblast may highlight molecular mechanisms of trophoblast regulation important for placenta accreta and *in vitro* models of the placenta.

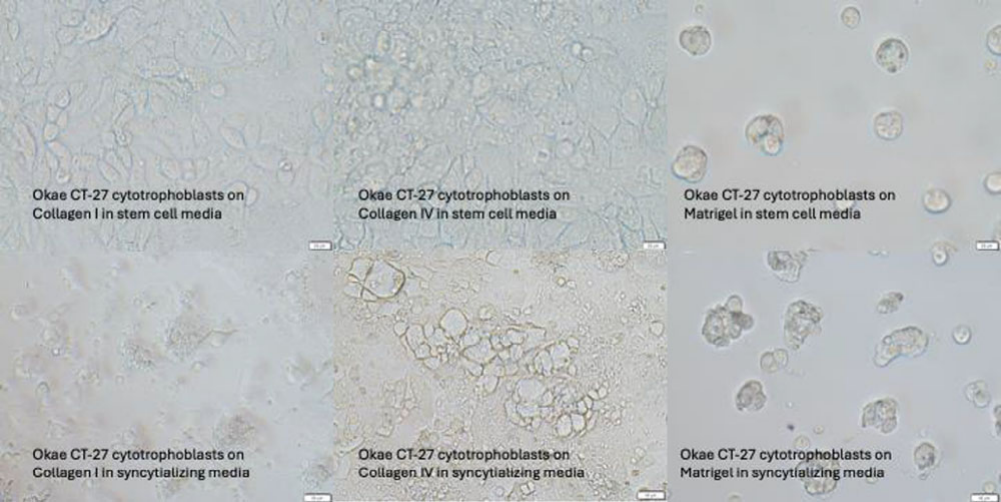

**10:30 AM – 12:00 PM**

## 986 | **The** Impact **of Intimate Partner Substance Use on Relationship Support and Satisfaction in Pregnancy**

Victoria E. Boyd^1^; A. Dhanya Mackeen^2^; Cynthia Drazenovich^1^; Wadia Mulla^3^; Hyagriv Simhan^4^; Richard Legro^5^; Douglas Teti^6^; Wenke Hwang^5^; Tian Guo^7^; Brenda Phillips^5^; Kristin K. Sznajder^5^

^1^Geisinger, Danville, PA; ^2^Geisinger Medical Center, Danville, PA; ^3^Temple, Temple, PA; ^4^University of Pittsburgh Medical Center, Pittsburgh, PA; ^5^Pennsylvania State University, Hershey, PA; ^6^Pennsylvania State University, University Park, PA; ^7^Geisinger, Geisinger, PA

**Objective**: While having a partner may increase social support, the effect of partner substance use on support is unclear. We examined how partner substance use affects perceived social support, resiliency, finances, and relationship quality during pregnancy from the pregnant patient's perspective.

**Study Design**: This is a secondary analysis of the Pennsylvania (PA)‐Maternal and Infant Health in a Pandemic study, a prospective survey study conducted at 4 PA health systems (8/23–5/25). We compared pregnant adults whose partners engaged in recreational substances during pregnancy to those whose did not. The primary outcome was social support (Modified Medical Outcomes Study Social Support Scale). Secondary outcomes included resiliency (Brief Resiliency Scale), financial strain, sexually transmitted infection (STI) exposure, conflict and coparenting dynamics (Coparenting Relationship Scale), birth control use, partner substance use during pregnancy, and relationship satisfaction. Demographics were described using means (+SD) and medians (IQR). The Kruskal Wallis and Chi‐squared tests were used to analyze continuous and categorical bivariate associations, respectively. Generalized linear and logistic regression models were used to adjust for baseline differences; *p*‐values of < 0.05 were considered significant.

**Results**: 2812 pregnancies were included. Partners who took substances were older and had an advanced degree (Table 1). Patients whose partners took substances were older, Caucasian (82.4% vs. 89.5%, *p* < 0.01), took substances, were prescribed opioids, and experienced intimate partner violence. Adjusted analyses found no differences in perceived social support, resilience, financial strain, STI exposure, postpartum contraception, or relationship satisfaction (Table 2). Patients whose partners took substances reported more conflict and lower coparenting scores.

**Conclusion**: Partners use of substances resulted in more reported conflict and worse coparenting dynamics, however, did not affect patients perceived support, resilience, financial strain, STI exposure, birth control use, or relationship happiness.

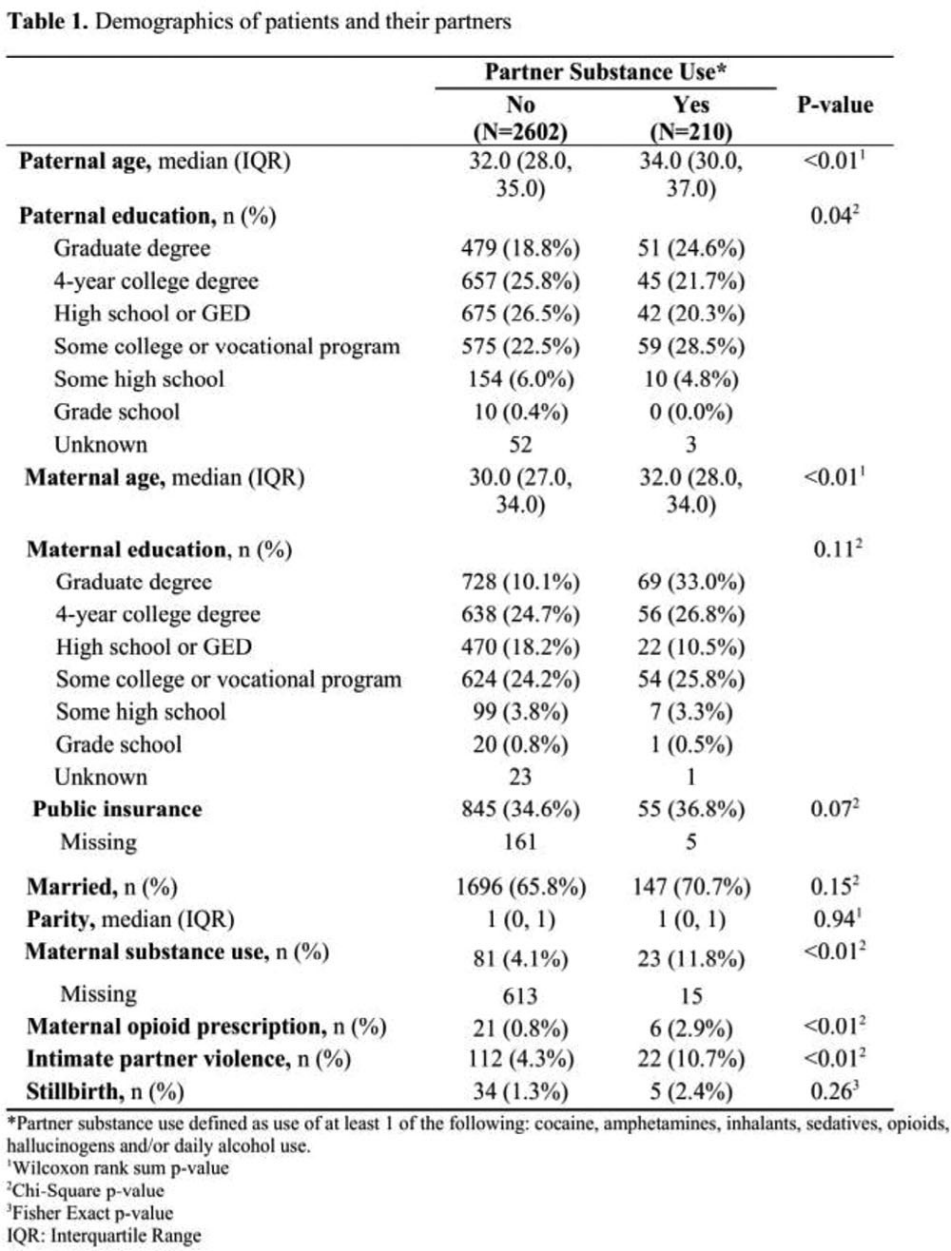

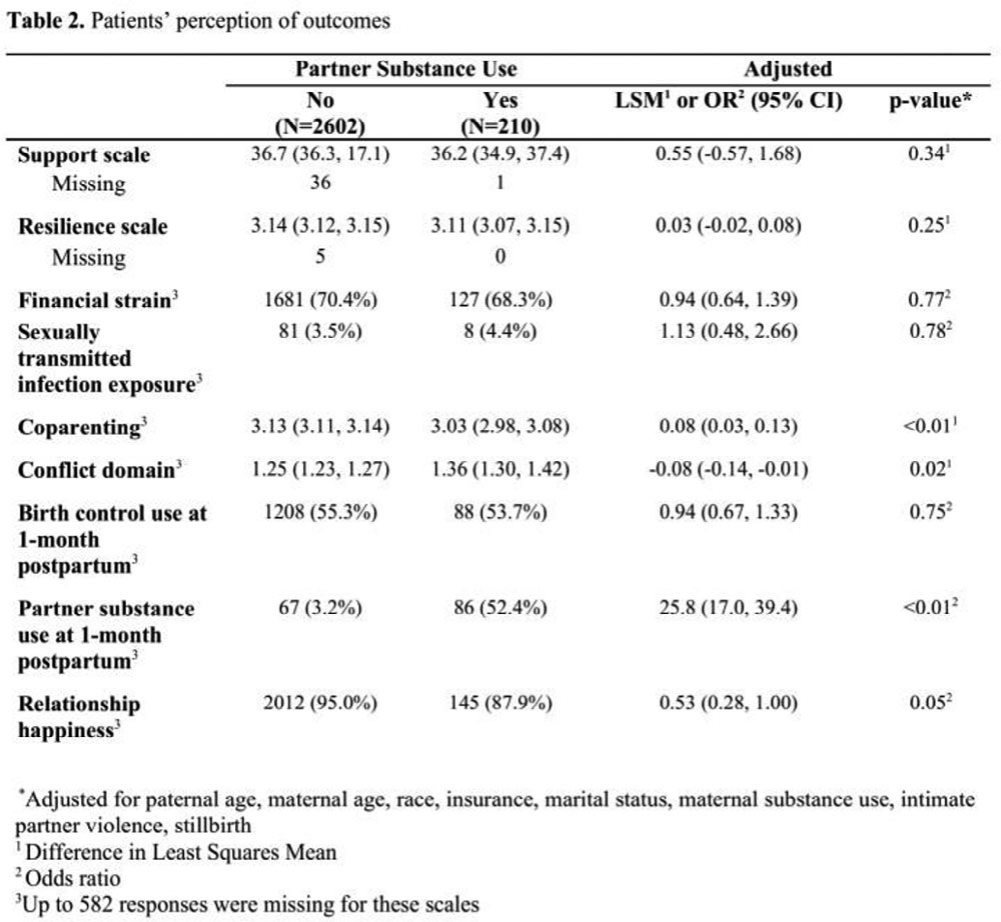

**10:30 AM – 12:00 PM**

## 987 | Optimizing **Prenatal Genetic Risk Assessment: Completion Rates and Sociodemographic Factors Across Carrier Screening Modalities**

Wissam Akkary^1^; Lina A. Fouad^2^; Blair Stevens^3^; Meagan Choates^4^; Luana Goulet^4^; Aranza Gonzalez Cendejas^5^; Irene A. Stafford^1^

^1^Division of Maternal‐Fetal Medicine, Department of Obstetrics, Gynecology and Reproductive Sciences, McGovern Medical School at UTHealth Houston, Houston, TX; ^2^Division of Maternal Fetal Medicine, Department of Obstetrics, Gynecology and Reproductive Sciences, McGovern Medical School at UTHealth Houston, Houston, TX; ^3^Division of Genetics, Department of Obstetrics, Gynecology and Reproductive Sciences, McGovern Medical School at UTHealth Houston, Houston, TX; ^4^UTHealth Houston McGovern Medical School, Houston, TX; ^5^UTHealth, Houston, TX

**Objective**: To compare completion rates of fetal risk assessment (RA) by carrier screening type (core panel, expanded carrier screening [ECS], and single‐gene NIPT [sgNIPT]) and to evaluate sociodemographic predictors of RA completion.

**Study Design**: After institutional approval, we conducted a retrospective cohort study of 1, 153 pregnant patients with abnormal carrier screening results between January 2023 and July 2025 at a single academic center. Completion of RA was defined as follow‐up paternal testing or sgNIPT where applicable. Screening modalities included core (12%, *n* = 142), ECS (81.6%, *n* = 941), and sgNIPT (6.1%, *n* = 70). Sociodemographic variables included age, parity, primary language, insurance status, and father of baby (FOB) presence at counseling visit. Multivariable logistic regression tests were used for analysis and adjusted relative risks (aRR) with 95% CI's were reported.

**Results**: RA completion occurred in 23.9% of core panel patients (34/142), 60.3% of ECS patients (568/941), and 74.3% of sgNIPT patients (52/70), *p* < 0.05. Among ECS patients, 69% had abnormalities that were not eligible for further testing with sgNIPT. Medicaid coverage was significantly associated with a 17% lower probability of RA completion compared to private insurance (aRR = 0.83, 95% CI: 0.73–0.95). Presentation for counseling at 18–24 weeks was associated with 23% lower completion than presentation at ≤12 weeks (aRR = 0.77, 95% CI: 0.61–0.94) and. FOB attendance was associated with a 40% higher likelihood of RA completion (aRR = 1.40, 95% CI: 1.25–1.57).

**Conclusion**: sgNIPT was associated with the highest RA completion. However, disparities by insurance, timing of care, and partner involvement remain significant. While sgNIPT offers a promising tool to streamline genetic risk assessment, it has limitations and may not be appropriate as a standalone strategy for all patients. Thoughtful integration with equitable access and counseling strategies is warranted.

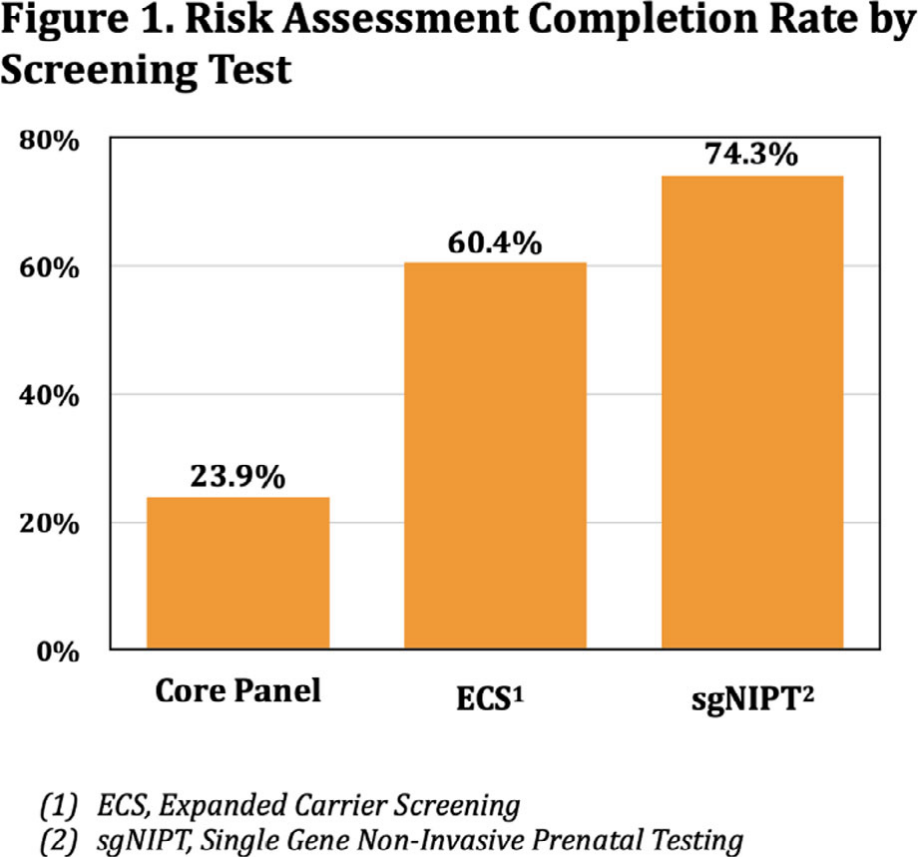

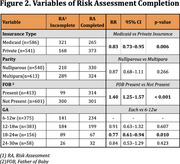

**10:30 AM – 12:00 PM**

## 988 | **Racial** and **Ethnic Differences in Severe Maternal Morbidity Associated with HELLP Syndrome**

Yadira L. Bribiesca Leon^1^; Ariane C. Youssefzadeh^2^; Zaira Chavez Jimenez^1^; Fay Pon^1^; Elizabeth B. Sasso^2^; Gordon J. Brian^2^; Brendan H. Grubbs^2^; Nicole M. Chadwick^3^; Marc H. Incerpi^2^; Joseph G. Ouzounian^2^; Koji Matsuo^4^

^1^Keck School of Medicine of USC, University of Southern California, Los Angeles, CA; ^2^Division of Maternal‐Fetal Medicine, Department of Obstetrics and Gynecology, University of Southern California, Los Angeles, CA; ^3^Department of Obstetrics and Gynecology, Los Angeles General Medical Center, Los Angeles, CA; ^4^Division of Gynecologic Oncology, Department of Obstetrics and Gynecology, University of Southern California, Los Angeles, CA

**Objective**: To assess race/ethnicity‐specific differences in severe maternal morbidity (SMM) associated with HELLP (hemolysis, elevated liver enzymes, and low platelets) syndrome.

**Study Design**: This cross‐sectional study included 62, 715 hospital deliveries with a diagnosis of HELLP syndrome from 2016–2022, obtained from the National Inpatient Sample. Exposure was race/ethnicity, including White (*n* = 36, 060), Hispanic (*n* = 14, 075), Black (*n* = 10, 020), or Asian (*n* = 2, 560). Main outcome measures were SMM defined by the Center for Disease Control and Prevention (20 indicators), assessed with multivariable generalized linear model. Secondary outcomes included hypertensive crisis.

**Results**: Compared to White patients, Black (adjusted‐incidence rate ratio [aIR] 1.80, 95% confidence interval [CI] 1.70–1.91), Asian (aIR 1.85, 95% CI 1.68–2.04), and Hispanic (aIR 1.33, 95% CI 1.25–1.41) patients had higher rates of any SMM. When assessed for the extent of SMM indicators, the likelihood of having triple or more SMM indicators among Asian (aIR 2.82, 95% CI 2.43–3.27), Hispanic (aIR 2.30, 95% CI 2.11–2.51) or Black (aIR 2.28, 95% CI 2.54–3.03) patients, exceeded two‐fold compared to Whites. When comparing individual morbidity to White patients, Black subjects were more likely to have ventilation needs, cardiac morbidity, and cerebrovascular accident; Hispanic subjects were more likely to have cardiac morbidity, shock, and sepsis; and Asian subjects were more likely to have shock, sepsis, cardiac morbidity, and ventilation needs (all, aIR >2.50). In addition, Black (aIR 3.17, 95% CI 2.63–3.82) and Hispanic (aIR 2.74, 95% CI 2.26–3.32) patients had two times higher rates of hypertensive crisis compared to White patients.

**Conclusion**: Among patients who had HELLP syndrome, the extent and severity of SMM was greater for Blacks, Hispanics and Asians compared to Whites. The substantial racial and ethnic disparities in SMM warrant future investigation to understand and mitigate these inequalities.

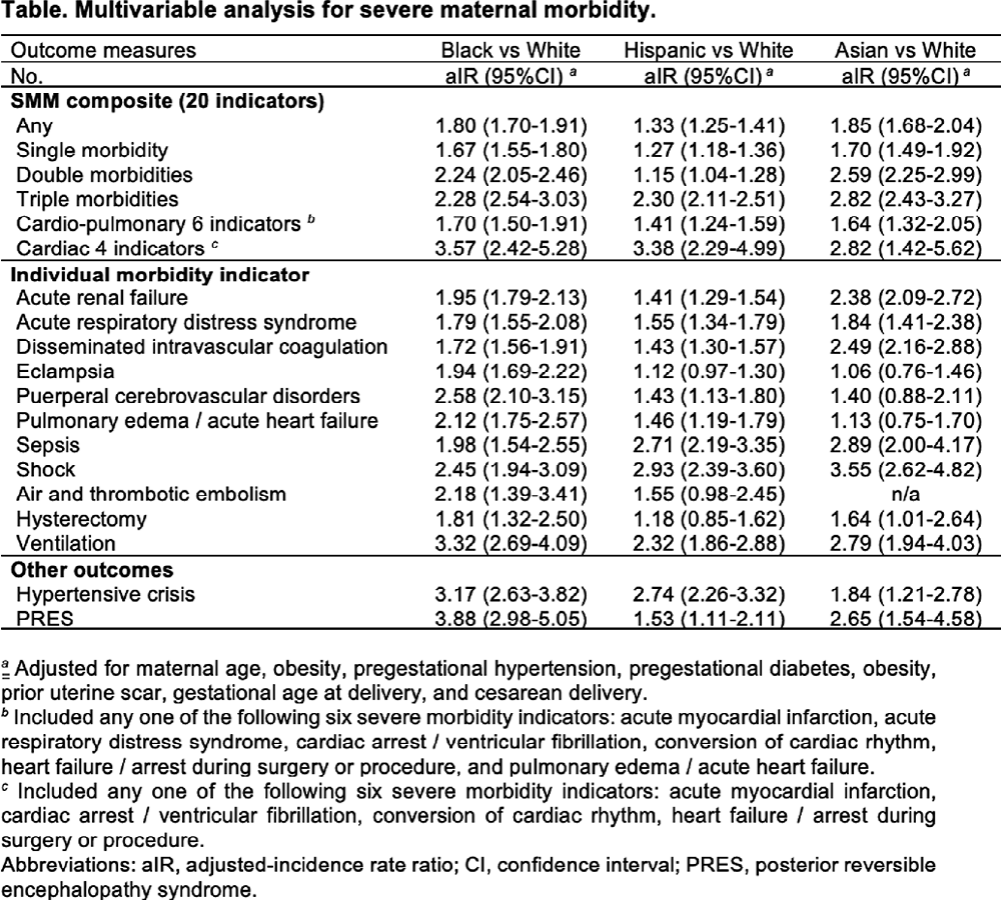

**10:30 AM – 12:00 PM**

## 989 | **When to Give Magnesium: Predicting Early Delivery After PPROM**

Yael Brantz^1^; Michal Fishel Bartal^2^; Michal Axelrod^3^; Lital Shaham^4^; Nimrod Dori‐Dayan^5^

^1^Department of Obstetrics and Gynecology, Sheba Medical Center, Ramat gan, HaMerkaz; ^2^Division of Maternal‐Fetal Medicine, Department of Obstetrics, Gynecology and Reproductive Sciences, Sheba Medical Center, Tel Aviv University, Israel, Ramat Gan, HaMerkaz; ^3^Sheba Medical Center, Sheba Medical Center, HaMerkaz; ^4^Department of Obstetrics and Gynecology, Sheba Medical Center, Sheba Medical Center, HaMerkaz; ^5^Sheba Medical Center, Ramat Gan, HaMerkaz

**Objective**: In premature preterm rupture of membranes (PPROM) between 24+0 and 31+6 weeks, timing magnesium sulfate for neuroprotection is challenging — too early may result in overtreatment, while too late may miss the therapeutic window. We aimed to identify predictors of delivery within 24 hours to guide timely and targeted use.

**Study Design**: We conducted a retrospective cohort study of all individuals with PPROM between 24+0 and 31+6 weeks at a tertiary medical center (2011–2025). Patients were stratified by latency: delivery within ≤24 hours vs. >24 hours. We compared obstetric history, presenting symptoms and outcomes between groups. Multivariable logistic regression was used to determine independent predictors of early delivery.

**Results**: Of 463 patients with PPROM, 169 (36.5%) delivered within 24 hours. Independent risk factors for delivery within 24 hours included multifetal pregnancy (46.2% vs. 25.5%, aOR 2.3, 95% CI 1.5–3.5), premature contractions at presentation (35.5% vs. 13.9%, aOR 2.3, 95% CI 1.4–3.9), cervical length < 25 mm (13.6% vs. 3.4%, aOR 2.7, 95% CI 1.1–6.3), WBC count >11 K/µL (68.7% vs. 55.5%, aOR 1.6, 95% CI 1–2.4) and gestational age >30 weeks’ gestation (55.6% vs. 32.7%, aOR 2.5, 95% CI 1.6–3.7) (Table). The combined presence of at least three risk factors was associated with 70.3% risk for delivery within 24 hours and yielded a positive likelihood ratio of 4.1 (95% CI 3.6–4.7) (Figure).

**Conclusion**: Over one‐third of patients with PPROM between 24 and 32 weeks will deliver within 24 hours. Multifetal gestation, short cervix, contractions at presentation, leukocytosis and gestational age > 30 weeks are risk factors that can help identify those at greatest risk. Targeted magnesium use based on these factors may improve outcomes and avoid overtreatment.

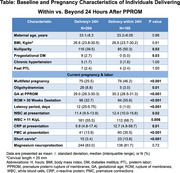

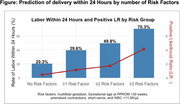

**10:30 AM – 12:00 PM**

## 990 | **Maternal and Neonatal Outcomes in Low‐Risk Women at Term After Waterbirth**

Yael Reichman^1^; Omri Dominsky^2^; Roza Shperling‐Berkovitz^3^; Ayelet Dangot^2^; Yariv Yogev^4^; Anat Lavie^5^

^1^Lis Hospital for Women's Health, Tel Aviv Sourasky Medical Center, Tel Aviv, Tel Aviv; ^2^Lis Hospital for Women's Health, Tel Aviv Sourasky Medical Center, Lis Hospital for Women's Health, Tel Aviv Sourasky Medical Center, HaMerkaz; ^3^Lis Hospital for Women's Health, Tel Aviv Sourasky Medical Center, ichilov, HaMerkaz; ^4^Lis Hospital for Women's Health, Tel Aviv Sourasky Medical Center Gray Faculty of Medicine, Tel Aviv University, Israel, Lis Hospital for Women's Health, Tel Aviv Sourasky Medical Center, Tel Aviv; ^5^Lis Hospital for Women's Health, Tel Aviv Sourasky Medical Center, Lis Hospital for Women's Health, Tel Aviv Sourasky Medical Center, Tel Aviv

**Objective**: To compare maternal and neonatal outcomes after waterbirth versus natural birth in a contemporary low‐risk cohort.

**Study Design**: Retrospective cohort of singleton, cephalic, term (37–42 wk) vaginal deliveries without regional analgesia at a single university hospital (∼13 000 births/yr, June 2024–June 2025). Eligibility: estimated fetal weight 2 500–4 000 g, normal anatomy scan, no uterine scar or major comorbidity, reassuring intrapartum monitoring. Women electing birth in the hospital birthing center were grouped by intended setting (water vs land). The primary outcomes were perineal tears (intact perineum, 1st, 2nd, 3rd/4th), episiotomy, postpartum haemorrhage (defined as estimated blood loss ≥ 1 000 mL), need for manual removal of the placenta and duration of the second and third stage of labor. Secondary outcomes were 5‐min Apgar < 7, neonatal resuscitation, NICU admission and need for sepsis work‐up.

**Results**:

1. Overall, 10, 236 vaginal deliveries occurred during the study period, of which 232 (2.3 %) met inclusion; 87 (37.5 %) delivered in water and 145 (62.5 %) delivered in the natural birth center without water immersion. Among 95 women who planned waterbirth, 87 (91.6%) completed delivery in water.
2. Second‐degree tears were less frequent after waterbirth overall (13.8 % vs 24.8 %; *p* = 0.046) and in primiparas specifically (23.1 % vs 48.7 %; *p* = 0.043).
3. Rates of intact perineum (54 % vs 48.3 %), first‐degree tears (31 % vs 24.1 %), 3rd/4th‐degree tears (0 vs 0.7 %), episiotomy (0 vs 3.4 %), PPH (2.3 % vs 3.4 %), manual removal of the placenta (1.1 % vs 2.8 %), and labor durations were similar between groups (all *p* > 0.095).
4. Neonatal outcomes were comparable between groups (all *p* > 0.30).

**Conclusion**: In our cohort, more than 90 % of women who planned waterbirth achieved it. Waterbirth was associated with a significantly lower rate of second‐degree perineal tears without adversely affecting other maternal or neonatal outcomes or changing labor and delivery duration.

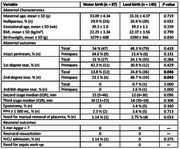

**10:30 AM – 12:00 PM**

## 991 | **Parity‐**Dependent **Outcomes and Procedural Dynamics in Non Reassuring Fetal Heart rate (NRFHR) indicated vacuum‐Assisted Deliveries**

Yael Rosental^1^; Bar Narkis^2^; Anat Pardo^3^; Or Bercovich^4^; Lior Friedrich^5^

^1^'Rabin medical center', Rabin medical center/ Petach tikwa, HaMerkaz; ^2^rabin medical center, Petach Tikwa, Israel, Rabin medical center/ Petach tikwa, HaMerkaz; ^3^Rabin Medical Center, Petach Tikva, HaMerkaz; ^4^Helen Schneider Hospital for Women, Rabin Medical Center, Petach Tikva, Israel, Helen Schneider Hospital for Women, Rabin Medical Center, Petach Tikva, HaMerkaz; ^5^Helen Schneider Hospital for Women, Rabin Medical Center, Petah Tikva, Israel, Petah Tikva, HaMerkaz

**Objective**: To compare maternal and neonatal outcomes, as well as procedural characteristics, of vacuum‐assisted deliveries (VAD) for non‐reassuring fetal heart rate (NRFHR) between primiparous and multiparous individuals.

**Study Design**: This retrospective cohort study included patients who underwent VAD for NRFHR between 2012 and 2024. Participants were stratified by parity—primiparas versus multiparas. Maternal and neonatal outcomes were compared, along with procedural characteristics such as fetal head station, head position at vacuum initiation, and procedure duration. Analyses were performed using univariate comparisons and multivariate logistic regression.

**Results**: Among 3833 individuals, 2908 were primiparas and 925 multiparas. Despite less favorable fetal head conditions in multiparas—higher rates of station S+1 at vacuum application (61.1% vs. 43.4%, *p* < 0.01) and a trend toward more occiput posterior positions (30.9% vs. 19.1%, *p* = 0.078)—procedure duration was shorter in this group (171 vs. 207 sec, *p* < 0.01). Maternal outcomes were significantly better in multiparas, with a lower composite complication rate (adjusted OR 0.37, 95% CI: 0.24–0.59). The maternal composite outcome included postpartum hemorrhage, blood transfusion, and severe perineal tears. Neonatal outcomes—including Apgar scores, cord pH, NICU admission, and skull‐related complications—were comparable between groups.

**Conclusion**: Multiparas undergoing VAD for NRFHR had reduced maternal morbidity and shorter procedures, despite less favorable fetal head conditions. The shorter operative time may reflect better physiological feasibility in multiparous patients. Additionally, the willingness to proceed with VAD under suboptimal conditions may suggest that clinicians feel more confident performing operative deliveries in women with prior vaginal births. These findings underscore the importance of considering both patient‐specific physiological factors and clinician‐related decision‐making behaviors when evaluating the appropriateness and safety of vacuum‐assisted deliveries.

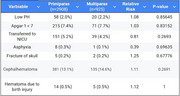

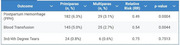

**10:30 AM – 12:00 PM**

## 992 | **Severe** Maternal **Morbidity in Pregnant Individuals With Postural Orthostatic Tachycardia Syndrome: A Population‐Based Analysis**

Yanling Dong; Shriddha Nayak; Alexandra D. Forrest; Arthur Jason Vaught; Marika Toscano

Johns Hopkins University, Baltimore, MD

**Objective**: Postural orthostatic tachycardia syndrome (POTS) is a complex autonomic nervous system disorder that has gained increasing clinical recognition and prevalence, particularly in the post‐COVID era. Its impact on pregnancy and potential association with severe maternal morbidity (SMM) remain poorly understood. This study assessed the association between POTS and SMM during pregnancy.

**Study Design**: This population‐based, retrospective cohort study used TriNetX, a global research network of de‐identified electronic health records from 106 healthcare organizations. Individuals aged 12–55 years with a pregnancy diagnosis between July 2020 and July 2025 were included. Two cohorts were derived: with and without a POTS diagnosis within one year prior to, or one month after, pregnancy diagnosis. The cohorts were propensity score matched 1:1 based on age, race/ethnicity, and comorbidities (diabetes, hypertension, obesity, asthma, tobacco use, autoimmune disease). SMM was defined using the CDC's 21 indicators and corresponding ICD‐10 codes. The primary outcome was adjusted odds ratio (aOR) of composite SMM within one year of pregnancy; secondary outcomes included aORs for individual indicators.

**Results**: Of 2, 718, 856 pregnancies, 5, 058 were affected by POTS. Demographics are shown in Table 1. After matching, 5, 040 individuals remained in each cohort. POTS was associated with higher odds of composite SMM (aOR 2.24, 95% CI 1.85–2.71, *p* < 0.0001) compared to those without POTS. It was also associated with increased odds of six individual SMM indicators, with puerperal cerebrovascular disorders, air and thrombotic embolism, shock and pulmonary edema/acute heart failure showing aORs >3 (Table 2).

**Conclusion**: Pregnant individuals with POTS had over twice the odds of composite SMM and more than threefold odds of thrombotic events, shock and pulmonary edema/acute heart failure. These findings underscore the need for individualized thromboembolism risk assessment, multidisciplinary care, and further research to clarify the pathophysiologic mechanisms linking POTS and SMM.

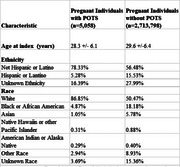

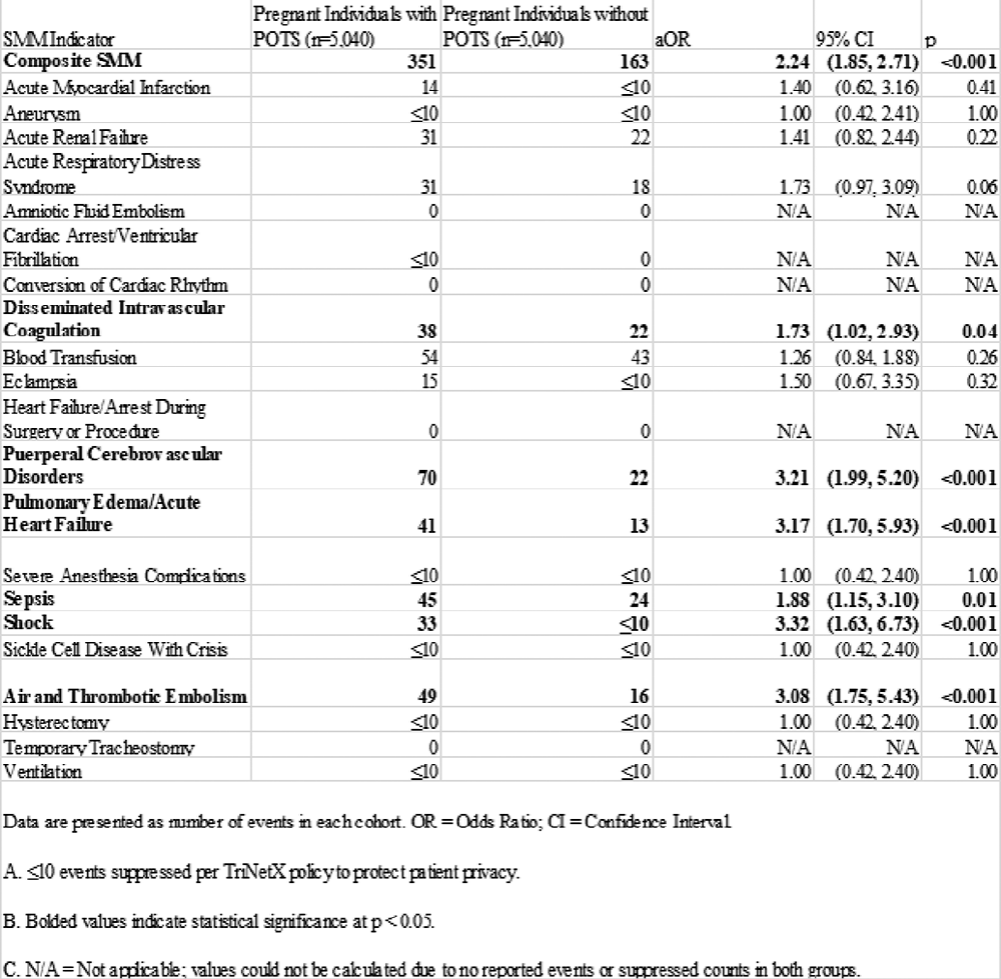

**10:30 AM – 12:00 PM**

## 993 | Admission **Systolic Blood Pressure in Early‐Onset Preeclampsia With Severe Features and Outcomes**

Yossi Bart^1^; Wissam Akkary^1^; Zakaria Doughan^2^; Joe Haydamous^3^; Ahmed Zaki Moustafa^4^; Sean C. Blackwell^1^; Baha M. Sibai^1^

^1^Division of Maternal‐Fetal Medicine, Department of Obstetrics, Gynecology and Reproductive Sciences, McGovern Medical School at UTHealth Houston, Houston, TX; ^2^Department of Obstetrics and Gynecology, McGovern Medical School at UT Health, Houston, Houston, TX; ^3^Department of Obstetrics, Gynecology and Reproductive Sciences, McGovern Medical School at UTHealth Houston, Department of Obstetrics and Gynecology, McGovern Medical School at UT Health, Houston, TX; ^4^Division of Maternal‐Fetal Medicine, Department of Obstetrics, Gynecology and Reproductive Sciences, McGovern Medical School at UTHealth Houston, University of Texas – Houston, TX

**Objective**: This study aimed to assess the association between the highest SBP on the first day of admission and adverse maternal and fetal outcomes among patients with early‐onset preeclampsia with severe features.

**Study Design**: A retrospective cohort study at a Level IV referral center, including patients diagnosed with preeclampsia with severe features between 23w0d and 33w6d (2016–2025). Patients with any component of the primary outcome present at admission were excluded. The primary outcome was a composite adverse outcome (CAO), including placental abruption, fetal death, eclampsia, HELLP syndrome, acute kidney injury, pulmonary edema, heart failure, stroke, and maternal death. Patients were further stratified by diagnostic criteria: those diagnosed based solely on severe‐range blood pressure versus those diagnosed by any other combination of the criteria. Poisson regression was used to calculate adjusted relative risk (aRR) with 95% confidence intervals (CI).

**Results**: Overall, 465 patients met inclusion criteria. Of those, 49 (10%) had SBP < 160 mmHg, 162 (35%) had SBP of 160–179 mmHg, and 254 (55%) had SBP ≥180 mmHg at admission. CAO occurred in 71 (15%) patients, increasing with SBP severity (6%, 11%, and 19%, respectively; *p* = 0.02). After adjustment for statistically confounding variables, SBP ≥180 mmHg was associated with a 3.37‐fold increased risk of CAO compared to SBP < 160 (95% CI 1.10–10.26, Table). Stratified analysis by diagnostic criteria showed a dose‐response relationship between SBP and CAO (Figure). Among those diagnosed by blood pressure alone, SBP ≥180 mmHg was associated with an aRR of 1.85 (95% CI 1.02–3.33) for the CAO compared to SBP < 180. In patients meeting any other diagnostic criteria combination, SBP ≥180 mmHg conferred an aRR of 5.65 (95% CI 1.10–28.97) compared to SBP < 160, with 34% developing CAO.

**Conclusion**: Among patients with early‐onset preeclampsia with severe features, higher admission SBP, particularly ≥180 mmHg, was associated with the CAO rate. This dose‐dependent relationship persisted across diagnostic subgroups.

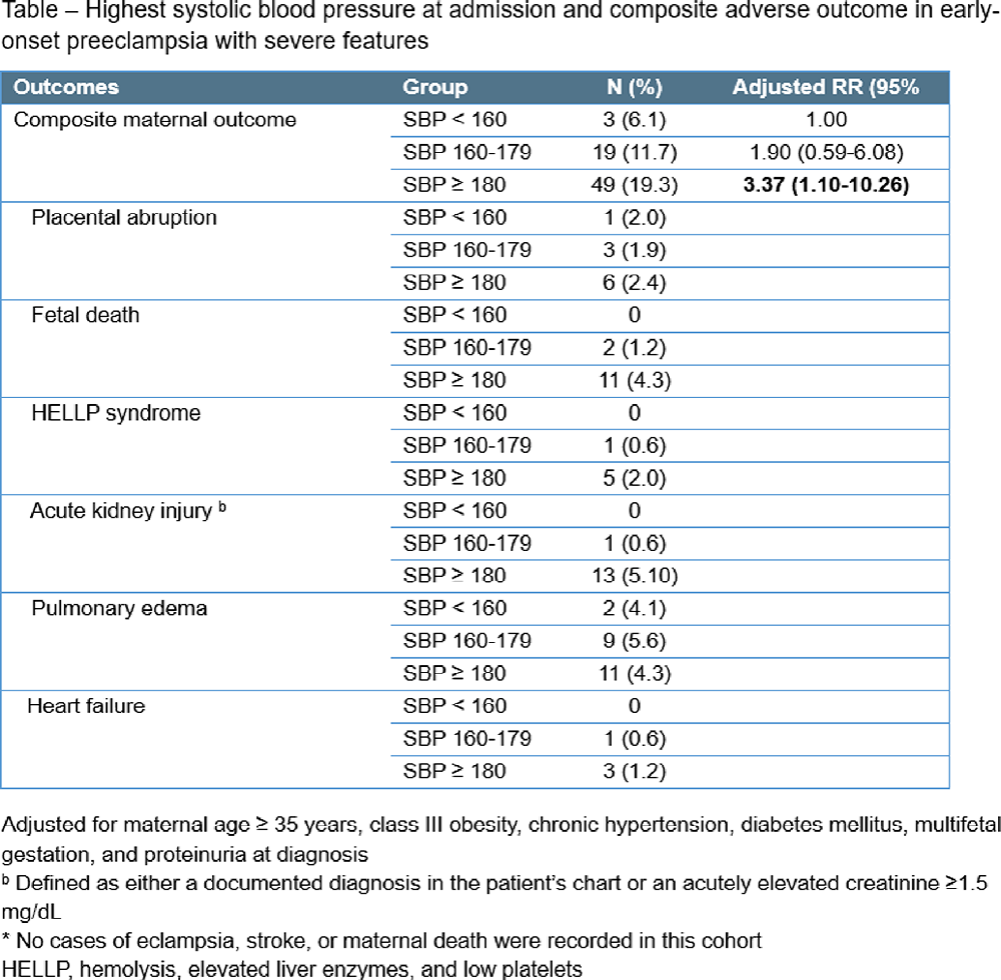

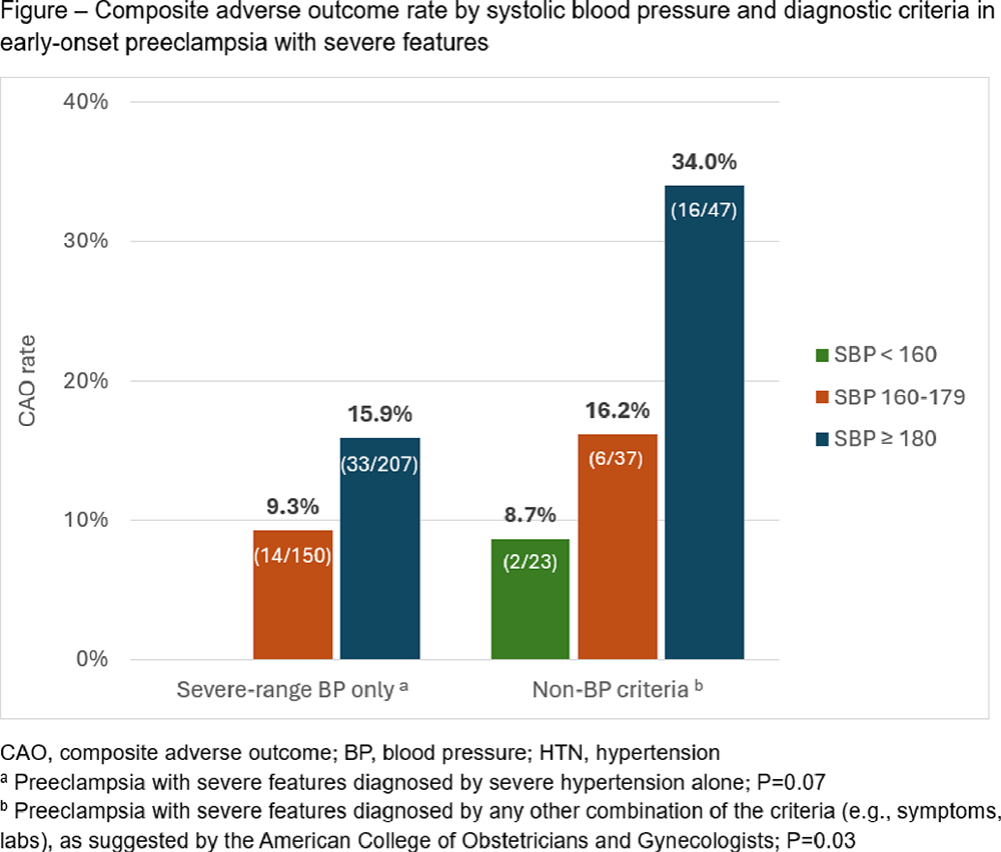

**10:30 AM – 12:00 PM**

## 995 | **Are** Characteristics **in Pregnancies With Opioid Use Disorder (OUD) Associated With Neighborhood Disadvantage?**

Zenobia Gonsalves^1^; Anne Claire Burghard^2^; Rachel Lee^3^; Chaitali Korgaonkar‐cherala^4^; David J. Garry^5^; Kimberly Herrera^3^; Cassandra Heiselman^3^

^1^Stony Brook University Hospital, Great Neck, NY; ^2^Renaissance School of Medicine at Stony Brook University, Stony Brook, NY; ^3^Stony Brook University Hospital, Stony Brook, NY; ^4^Stony Brook Medicine, Stony Brook, NY; ^5^Stony Brook Medicine, Stony Brook Medicine, NY

**Objective**: To determine the relationship between area deprivation index (ADI) and pregnancy characteristics in patients with OUD who are on medications for OUD (MOUD).

**Study Design**: This was a retrospective study of pregnant patients on MOUD who delivered at a single institution between 2017 and 2023. The ADI was determined using the University of Wisconsin's Neighborhood Atlas based on the patient's mailing address at time of delivery admission. ADI was characterized as a state decile ranging from 1 to 10, with 10 representing the highest deprivation areas. Patients were excluded if they provided a P.O. box number as their address or if an ADI was not listed for their address. Charts were abstracted for maternal characteristics and key perinatal outcomes. Statistical analyses were performed using Mann‐Whitney U, Chi‐square, and Spearman's correlation tests, with a *p*‐value < 0.05 deemed statistically significant.

**Results**: 404 patients were included in the analysis. There was found to be a difference in median distributions of ADI scores for patients with white race (*p* = 0.04), government insurance (*p* = 0.04), multiparity (*p* = 0.02), and MOUD started in pregnancy (*p* = 0.02) [Table 1]. When assessing those with higher neighborhood deprivation (ADI > 6) with lower (ADI < 6), a difference was noted in the proportion of patients with government insurance (*p* = 0.02) and on psychiatric medications (*p* = 0.03) [Table 2]. A weak association was noted between ADI score and parity (rs = 0.116, *p* = 0.02).

**Conclusion**: Pregnant MOUD patients residing in more highly disadvantaged neighborhoods were more likely to be multiparous, of white race, have government insurance, have started MOUD in pregnancy, and be on psychiatric medications concomitantly. Additionally, a higher deprivation index was weakly associated with higher parity.

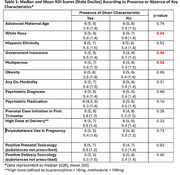

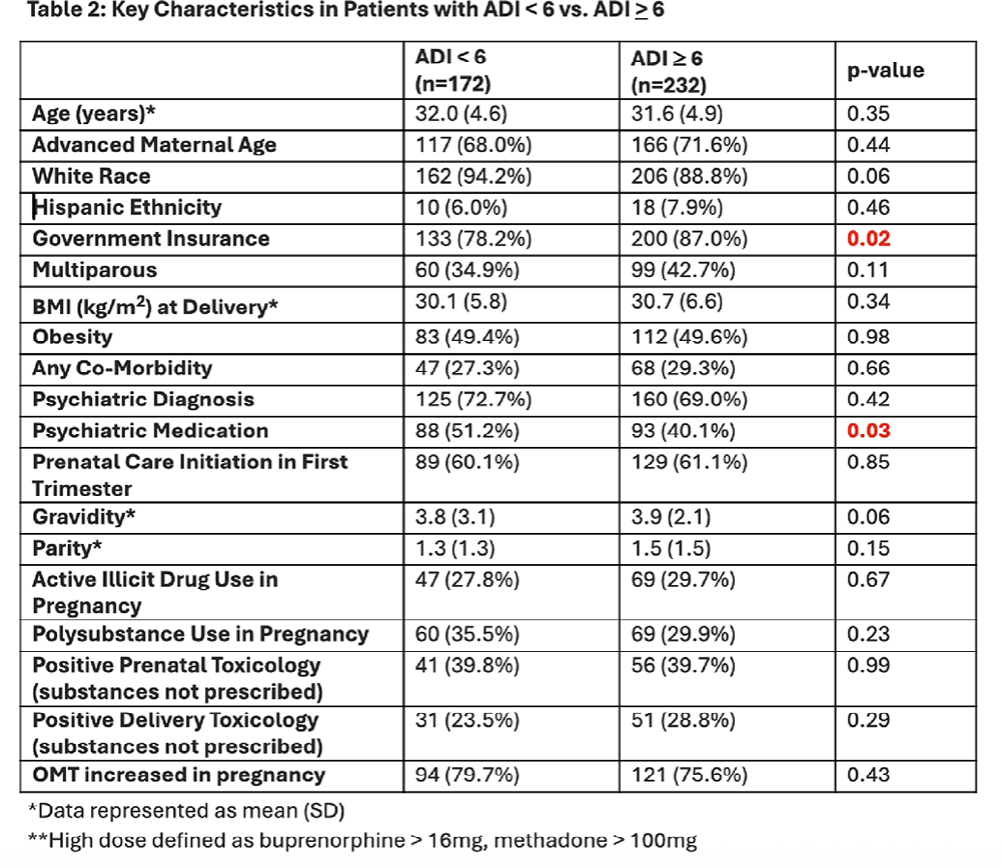

**10:30 AM – 12:00 PM**

## 996 | Longitudinal **MRI Comparison of Placental Immune and Oxygenation Profiles in Opioid‐Exposed Versus Spontaneous Preterm Pregnancies**

Zezhen Xiang^1^; Jeannie C. Kelly^1^; Nardhy Gomez‐Lopez^1^; Sam A. S. Williams^1^; Melissa Mills^2^; Emily Diveley, BSN^1^; Nandini Raghuraman^3^; Antonina I. Frolova^3^; Peinan Zhao^1^; Yong Wang^1^

^1^Washington University School of Medicine, Washington University School of Medicine, MO; ^2^Washington University School of Medicine, Saint Louis, MO; ^3^Washington University School of Medicine, St. Louis, MO

**Objective**: Prenatal opioid exposure (POE) and spontaneous preterm birth (SPTB) are both associated with placental dysregulation, but underlying mechanisms remain unclear. We hypothesized that placental immune activity and oxygenation are similarly disrupted in POE and SPTB pregnancies. Using longitudinal MRI, we compared placental profiles among POE, SPTB, and control pregnancies.

**Study Design**: Pregnant individuals with chronic POE were enrolled and matched to unexposed controls by fetal sex, maternal race, and Area Deprivation Index. Additional matches were identified in a previously imaged cohort of patients who experienced SPTB (spontaneous labor and delivery < 37 weeks). Participants underwent up to one MRI per trimester. Placental immune cell infiltration (“cell ratio”) was assessed using a validated imaging method developed by our team (Figure A), and oxygenation was evaluated via T2* mapping. A fuzzy clustering algorithm segmented placental compartments using T2*, apparent diffusion coefficient, and fractional anisotropy. Exponential regression and likelihood ratio tests were used to evaluate group differences.

**Results**: 21 participants (6 POE; 4 controls; 11 SPTB) with similar demographics were included; The control patients were all term births with no POE. SPTB occurred at a mean gestation of 34 weeks. Immune cell ratios in placental vessels increased over pregnancy in both SPTB (R^2^ = 0.714, *p* < 0.0001) and POE (R^2^ = 0.690, *p* = 0.0055), but not controls (R^2^ = 0.018, *p* = 0.6809); SPTB demonstrated a steeper increase than both POE (F = 3.69, *p* = 0.0389) and controls (F = 10.02, *p* = 0.0005) (Figure B). No longitudinal changes in tissue T2* were observed within groups, but SPTB showed persistently lower oxygenation compared to controls (F = 14.96, *p* < 0.0001), but not POE (F = 0.89, *p* = 0.4196) (Figure C).

**Conclusion**: Both POE and SPTB demonstrated increasing immune cells within placental vessels and persistently lower oxygenation within placental tissue, suggesting shared pathways for placental dysregulation. However, SPTB showed a steeper immune response, potentially distinguishing a threshold for risk of preterm birth.

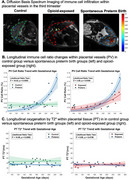

**10:30 AM – 12:00 PM**

## 997 | **Neonatal Outcomes Following Betamethasone Administration in Patients With Obesity**

Zimeng Gao^1^; Alexsaundra Zywicki^2^; Sarah Caveglia^3^; Carrie Irvine^2^; Sarah Crimmins^4^

^1^University of Rochester Medical center, Rochester, NY; ^2^University of Rochester Medical Center, Rochester, NY; ^3^University of Rochester Medical Center, University of Rochester Medical Center, NY; ^4^University of Rochester Medical Center—Roches, University of Rochester, NY

**Objective**: Investigate neonatal outcomes following betamethasone administration in patients with obesity.

**Study Design**: Retrospective case‐control study of preterm deliveries during which each patient obtained at least one dose of betamethasone. The control group included BMIs < 30 and the study group included BMIs ≥ 30. Exclusion included multiple gestations, T1DM, T2DM with pregnancy Hgb A1C > 7.0%, chorioamnionitis, non‐reassuring fetal heart tones requiring emergent delivery, fetal aneuploidy or major structural anomalies, and neonatal abstinence syndrome. Multivariate logistic regressions were performed to compare individual and composite neonatal resuscitation and NICU outcomes by BMI status with adjustments made for gestational age at delivery, neonatal birth weight, diagnosis of preeclampsia, and delivery by cesarean section.

**Results**: Of 1, 240 total patients, 504 (40%) had BMIs < 30, 321 (26%) were class I obese, 200 (16%) were class II obese, and 217 (18%) were class III obese. Relative to the non‐obese group, obese individuals were more likely to be older, of higher gravidity and parity, identify as Black American, deliver by cesarean, and experience preeclampsia. Neonates born to class III obese individuals experienced increased resuscitation with oxygen, PPV, and composite resuscitation (Table 1). No statistical differences in composite or individual neonatal outcomes (RDS, PVL, NEC, ROP, IVH), neonatal death, or length of NICU stay.

**Conclusion**: The data demonstrated differences in the need for neonatal resuscitation in patients with class III obesity, despite adjustments for gestational age, neonatal birth weight, diagnosis of preeclampsia, and delivery by cesarean section. However, these interventions are short term, as longitudinal NICU stays or complications are not statistically different relative to non‐obese individuals. This finding reveals a capacity for future investigations on potential divergent efficacies of pharmacotherapies in the class III obesity population compared to the non‐obese population.